# 2016 AHA/ACC Guideline on the Management of Patients With Lower Extremity Peripheral Artery Disease: A Report of the American College of Cardiology/American Heart Association Task Force on Clinical Practice Guidelines

**DOI:** 10.1161/CIR.0000000000000471

**Published:** 2016-11-13

**Authors:** Writing Committee Members, Marie D. Gerhard-Herman, Heather L. Gornik, Coletta Barrett, Neal R. Barshes, Matthew A. Corriere, Douglas E. Drachman, Lee A. Fleisher, Francis Gerry R. Flowkes, Naomi M. Hamburg, Scott Kinlay, Robert Lookstein, Sanjay Misra, Leila Mureebe, Jeffrey W. Olin, Rajan A.G. Patel, Judith G. Regensteiner, Andres Schanzer, Mehdi H. Shishehbor, Kerry J. Stewart, Diane Treat-Jacobson, M. Eileen Walsh, Jonathan L. Halperin, ACC/AHA Task Force on Clinical Practice Guidelines

**Affiliations:** * Writing committee members are required to recuse themselves from voting on sections to which their specific relationships with industry and other entities may apply; see Appendix 1 for recusal information; † Functioning as the lay volunteer/patient representative; ‡ ACC/AHA Representative; § Vascular and Endovascular Surgery Society Representative; ‖ Society for Cardiovascular Angiography and Interventions Representative; ¶ ACC/AHA Task Force on Clinical Practice Guidelines Liaison; # Inter-Society Consensus for the Management of Peripheral Arterial Disease Representative; ** Society for Vascular Medicine Representative; †† Society of Interventional Radiology Representative; ‡‡ Society for Clinical Vascular Surgery Representative; §§ Society for Vascular Surgery Representative; ‖‖ American Association of Cardiovascular and Pulmonary Rehabilitation Representative; ¶¶ Society for Vascular Nursing RepresentativeChair, ACC/AHA Task Force on Clinical Practice Guidelines

**Keywords:** AHA Scientific Statements, peripheral artery disease, claudication, critical limb ischemia, acute limb ischemia, antiplatelet agents, supervised exercise, endovascular procedures, bypass surgery, limb salvage, smoking cessation

## Abstract

Since 1980, the American College of Cardiology (ACC) and American Heart Association (AHA) have translated scientific evidence into clinical practice guidelines with recommendations to improve cardiovascular health. These guidelines, based on systematic methods to evaluate and classify evidence, provide a cornerstone of quality cardiovascular care.

In response to reports from the Institute of Medicine^1,2^ and a mandate to evaluate new knowledge and maintain relevance at the point of care, the ACC/AHA Task Force on Clinical Practice Guidelines (Task Force) modified its methodology.^3–5^ The relationships among guidelines, data standards, appropriate use criteria, and performance measures are addressed elsewhere.^5^

**Intended Use:**

Practice guidelines provide recommendations applicable to patients with or at risk of developing cardiovascular disease. The focus is on medical practice in the United States, but guidelines developed in collaboration with other organizations may have a broader target. Although guidelines may be used to inform regulatory or payer decisions, the intent is to improve quality of care and align with patients' interests. Guidelines are intended to define practices meeting the needs of patients in most, but not all, circumstances, and should not replace clinical judgment. Guidelines are reviewed annually by the Task Force and are official policy of the ACC and AHA. Each guideline is considered current until it is updated, revised, or superseded by published addenda, statements of clarification, focused updates, or revised full-text guidelines. To ensure that guidelines remain current, new data are reviewed biannually to determine whether recommendations should be modified. In general, full revisions are posted in 5-year cycles.^3–6^

**Modernization:**

Processes have evolved to support the evolution of guidelines as “living documents” that can be dynamically updated. This process delineates a recommendation to address a specific clinical question, followed by concise text (ideally <250 words) and hyperlinked to supportive evidence. This approach accommodates time constraints on busy clinicians and facilitates easier access to recommendations via electronic search engines and other evolving technology.

**Evidence Review:**

Writing committee members review the literature; weigh the quality of evidence for or against particular tests, treatments, or procedures; and estimate expected health outcomes. In developing recommendations, the writing committee uses evidence-based methodologies that are based on all available data.^3–7^ Literature searches focus on randomized controlled trials (RCTs) but also include registries, nonrandomized comparative and descriptive studies, case series, cohort studies, systematic reviews, and expert opinion. Only selected references are cited.

The Task Force recognizes the need for objective, independent Evidence Review Committees (ERCs) that include methodologists, epidemiologists, clinicians, and biostatisticians who systematically survey, abstract, and assess the evidence to address systematic review questions posed in the PICOTS format (P=population, I=intervention, C=comparator, O=outcome, T=timing, S=setting).^2,4–6^ Practical considerations, including time and resource constraints, limit the ERCs to evidence that is relevant to key clinical questions and lends itself to systematic review and analysis that could affect the strength of corresponding recommendations.

**Guideline-Directed Management and Treatment:**

The term “guideline-directed management and therapy” (GDMT) refers to care defined mainly by ACC/AHA Class I recommendations. For these and all recommended drug treatment regimens, the reader should confirm dosage with product insert material and carefully evaluate for contraindications and interactions. Recommendations are limited to treatments, drugs, and devices approved for clinical use in the United States.

**Class of Recommendation and Level of Evidence:**

The Class of Recommendation (COR; ie, the strength of the recommendation) encompasses the anticipated magnitude and certainty of benefit in proportion to risk. The Level of Evidence (LOE) rates evidence supporting the effect of the intervention on the basis of the type, quality, quantity, and consistency of data from clinical trials and other reports (Table 1).^3–5^ Unless otherwise stated, recommendations are sequenced by COR and then by LOE. Where comparative data exist, preferred strategies take precedence. When >1 drug, strategy, or therapy exists within the same COR and LOE and no comparative data are available, options are listed alphabetically.

**Relationships With Industry and Other Entities:**

The ACC and AHA sponsor the guidelines without commercial support, and members volunteer their time. The Task Force zealously avoids actual, potential, or perceived conflicts of interest that might arise through relationships with industry or other entities (RWI). All writing committee members and reviewers are required to disclose current industry relationships or personal interests, from 12 months before initiation of the writing effort. Management of RWI involves selecting a balanced writing committee and assuring that the chair and a majority of committee members have no relevant RWI (Appendix 1). Members are restricted with regard to writing or voting on sections to which their RWI apply. For transparency, members' comprehensive disclosure information is available online. Comprehensive disclosure information for the Task Force is also available online.

The Task Force strives to avoid bias by selecting experts from a broad array of backgrounds representing different geographic regions, sexes, ethnicities, intellectual perspectives/biases, and scopes of clinical practice, and by inviting organizations and professional societies with related interests and expertise to participate as partners or collaborators.

**Individualizing Care in Patients With Associated Conditions and Comorbidities:**

Managing patients with multiple conditions can be complex, especially when recommendations applicable to coexisting illnesses are discordant or interacting.^8^ The guidelines are intended to define practices meeting the needs of patients in most, but not all, circumstances. The recommendations should not replace clinical judgment.

**Clinical Implementation:**

Management in accordance with guideline recommendations is effective only when followed. Adherence to recommendations can be enhanced by shared decision making between clinicians and patients, with patient engagement in selecting interventions on the basis of individual values, preferences, and associated conditions and comorbidities. Consequently, circumstances may arise in which deviations from these guidelines are appropriate.

## 1. Introduction

### 1.1. Methodology and Evidence Review

The recommendations listed in this guideline are, whenever possible, evidence based. An initial extensive evidence review, which included literature derived from research involving human subjects, published in English, and indexed in MEDLINE (through PubMed), EMBASE, the Cochrane Library, the Agency for Healthcare Research and Quality, and other selected databases relevant to this guideline, was conducted from January through September 2015. Key search words included but were not limited to the following: *acute limb ischemia, angioplasty, ankle-brachial index, anticoagulation, antiplatelet therapy, atypical leg symptoms, blood pressure lowering/hypertension, bypass graft/bypass grafting/surgical bypass, cilostazol, claudication/intermittent claudication, critical limb ischemia/severe limb ischemia, diabetes, diagnostic testing, endovascular therapy, exercise rehabilitation/exercise therapy/exercise training/supervised exercise, lower extremity/foot wound/ulcer, peripheral artery disease/peripheral arterial disease/peripheral vascular disease/lower extremity arterial disease, smoking/smoking cessation, statin, stenting, and vascular surgery. Additional relevant studies published through September 2016, during the guideline writing process, were also considered by the writing committee, and added to the evidence tables when appropriate. The final evidence tables included in the* Online Data Supplement *summarize the evidence utilized by the writing committee to formulate recommendations*. Additionally, the writing committee reviewed documents related to lower extremity peripheral artery disease (PAD) previously published by the ACC and AHA.^9,10^ References selected and published in this document are representative and not all-inclusive.

As stated in the Preamble, the ACC/AHA guideline methodology provides for commissioning an independent ERC to address systematic review questions (PI-COTS format) to inform recommendations developed by the writing committee. All other guideline recommendations (not based on the systematic review questions) were also subjected to an extensive evidence review process. For this guideline, the writing committee in conjunction with the Task Force and ERC Chair identified the following systematic review questions: 1) Is antiplatelet therapy beneficial for prevention of cardiovascular events in the patient with symptomatic or asymptomatic lower extremity PAD? 2) What is the effect of revascularization, compared with optimal medical therapy and exercise training, on functional outcome and quality of life (QoL) among patients with claudication? Each question has been the subject of recently published, systematic evidence reviews.^11–13^ The quality of these evidence reviews was appraised by the ACC/AHA methodologist and a vendor contracted to support this process (Doctor Evidence [Santa Monica, CA]). Few substantive randomized or nonrandomized studies had been published after the end date of the literature searches used for the existing evidence reviews, so the ERC concluded that no additional systematic review was necessary to address either of these critical questions.

A third systematic review question was then identified: 3) Is one revascularization strategy (endovascular or surgical) associated with improved cardiovascular and limb-related outcomes in patients with critical limb ischemia (CLI)? This question had also been the subject of a high-quality systematic review that synthesized evidence from observational data and an RCT^14^; additional RCTs addressing this question are ongoing.^15–17^ The writing committee and the Task Force decided to expand the survey to include more relevant randomized and observational studies. Based on evaluation of this additional evidence the ERC decided that further systematic review was not needed to inform the writing committee on this question. Hence, the ERC and writing committee concluded that available systematic reviews could be used to inform the development of recommendations addressing each of the 3 systematic review questions specified above. The members of the Task Force and writing committee thank the members of the ERC that began this process and their willingness to participate in this volunteer effort. They include Aruna Pradhan, MD, MPH (ERC Chair); Natalie Evans, MD; Peter Henke, MD; Dharam J. Kumbhani, MD, SM, FACC; and Tamar Polonsky, MD.

### 1.2. Organization of the Writing Committee

The writing committee consisted of clinicians, including noninvasive and interventional cardiologists, exercise physiologists, internists, interventional radiologists, vascular nurses, vascular medicine specialists, and vascular surgeons, as well as clinical researchers in the field of vascular disease, a nurse (in the role of patient representative), and members with experience in epidemiology and/or health services research. The writing committee included representatives from the ACC and AHA, American Association of Cardiovascular and Pulmonary Rehabilitation, Inter-Society Consensus for the Management of Peripheral Arterial Disease, Society for Cardiovascular Angiography and Interventions, Society for Clinical Vascular Surgery, Society of Interventional Radiology, Society for Vascular Medicine, Society for Vascular Nursing, Society for Vascular Surgery, and Vascular and Endovascular Surgery Society.

### 1.3. Document Review and Approval

This document was reviewed by 2 official reviewers nominated by the ACC and AHA; 1 to 2 reviewers each from the American Association of Cardiovascular and Pulmonary Rehabilitation, Inter-Society Consensus for the Management of Peripheral Arterial Disease, Society for Cardiovascular Angiography and Interventions, Society for Clinical Vascular Surgery, Society of Interventional Radiology, Society for Vascular Medicine, Society for Vascular Nursing, Society for Vascular Surgery, and Vascular and Endovascular Surgery Society; and 16 additional individual content reviewers. Reviewers' RWI information was distributed to the writing committee and is published in this document (Appendix 2).

This document was approved for publication by the governing bodies of the ACC and the AHA and endorsed by the American Association of Cardiovascular and Pulmonary Rehabilitation, Inter-Society Consensus for the Management of Peripheral Arterial Disease, Society for Cardiovascular Angiography and Interventions, Society for Clinical Vascular Surgery, Society of Interventional Radiology, Society for Vascular Medicine, Society for Vascular Nursing, Society for Vascular Surgery, and Vascular and Endovascular Surgery Society.

### 1.4. Scope of Guideline

Lower extremity PAD is a common cardiovascular disease that is estimated to affect approximately 8.5 million Americans above the age of 40 years and is associated with significant morbidity, mortality, and QoL impairment.^18^ It has been estimated that 202 million people worldwide have PAD.^19^ The purpose of this document is to provide a contemporary guideline for diagnosis and management of patients with lower extremity PAD. This document supersedes recommendations related to lower extremity PAD in the “ACC/AHA 2005 Guidelines for the Management of Patients With Peripheral Arterial Disease”^9^ and the “2011 ACCF/AHA Focused Update of the Guideline for the Management of Patients With Peripheral Artery Disease.”^10^ The scope of this guideline is limited to atherosclerotic disease of the lower extremity arteries (PAD) and includes disease of the aortoiliac, femoropopliteal, and infrapopliteal arterial segments. It does not address nonatherosclerotic causes of lower extremity arterial disease, such as vasculitis, fibromuscular dysplasia, physiological entrapment syndromes, cystic adventitial disease, and other entities. Future guidelines will address aneurysmal disease of the abdominal aorta and lower extremity arteries and diseases of the renal and mesenteric arteries.

In developing the “2016 AHA/ACC Guideline on the Management of Patients With Lower Extremity Peripheral Artery Disease,” the writing committee reviewed the evidence to support recommendations in the relevant ACC/AHA guidelines noted in Table 2 and affirms the ongoing validity of the related recommendations, thus obviating the need to repeat existing guideline recommendations in the current guideline. Table 2 also contains a list of other statements that may be of interest to the reader. Table 3 includes definitions for PAD key terms used throughout the guideline.

## 2. Clinical Assessment for PAD

Evaluating the patient for PAD begins with the clinical history, review of symptoms, and physical examination.

### 2.1. History and Physical Examination: Recommendations


**Recommendations for
History and Physical Examination**
**COR****LOE****Recommendations**
**I****B-NR****Patients at increased risk of PAD
(**Table 4**)
should undergo a comprehensive medical history and a review of
symptoms to assess for exertional leg symptoms, including
claudication or other walking impairment, ischemic rest pain,
and nonhealing wounds.**^52–57^
See Online Data Supplement 1.The symptoms and signs of PAD are
variable. Patients with PAD may experience the classic symptom of
claudication or may present with advanced disease, including CLI.
Studies have demonstrated that the majority of patients with
confirmed PAD do not have typical claudication but have other
non–joint-related limb symptoms or are
asymptomatic.^53,55^
Atypical lower extremity symptoms related to PAD may include pain or
discomfort that begins at rest but worsens with exertion, pain or
discomfort that does not stop an individual from walking, and pain
or discomfort that begins with exertion but is not alleviated within
10 minutes of rest.^54^ Patients with PAD who do not have typical
claudication but have other leg symptoms, or who are asymptomatic,
have been shown to have functional impairment comparable to patients
with claudication.^54^ Thus, all patients at increased risk of PAD
should be asked not only about claudication but also about other
exertional non–joint-related limb symptoms and perceived
walking impairment.
**I****B-NR****Patients at increased risk of PAD
(**Table 4**)
should undergo vascular examination, including palpation of
lower extremity pulses (ie, femoral, popliteal, dorsalis pedis,
and posterior tibial), auscultation for femoral bruits, and
inspection of the legs and feet.**^56,58,59^
See Online Data Supplements.A thorough lower extremity vascular
examination and careful inspection of the legs and feet are
important components of the clinical assessment for PAD. To perform
a thorough examination, legs and feet are examined with lower
garments (pants/skirt, shoes, and socks) removed. Examination
findings suggestive of PAD are shown in Table 5. Lower extremity pulses should
be assessed and rated as follows: 0, absent; 1, diminished; 2,
normal; or 3, bounding. Reproducibility of pulse assessment is
better for detection of normal versus absent pulse than for normal
versus diminished pulse.^56^ Absence of the dorsalis pedis pulse is less
accurate for diagnosis of PAD than is absence of the posterior
tibial pulse because the dorsalis pedis pulse can be absent on
examination in a significant percentage of healthy
patients.^56,58^
The presence of multiple abnormal physical findings (ie, multiple
pulse abnormalities, bruits) increases the likelihood of confirmed
PAD.^56,58,59^ Abnormal physical findings,
such as a pulse abnormality, require confirmation with the
ankle-brachial index (ABI) to establish the diagnosis of PAD.
Similarly, an entirely normal pulse examination and absence of
bruits decreases the likelihood of confirmed PAD.^56,58^ The presence of nonhealing
lower extremity wounds may be a sign of CLI. Findings of cool or
discolored skin and delayed capillary refill are not reliable for
PAD diagnosis.^56^
To confirm the diagnosis of PAD, abnormal physical examination
findings must be confirmed with diagnostic testing (Section 3),
generally with the ABI as the initial test.
**I****B-NR****Patients with PAD should undergo
noninvasive blood pressure measurement in both arms at least
once during the initial assessment.**^60–62^
See Online Data Supplement 1.An inter-arm blood pressure difference
of >15 to 20 mm Hg is abnormal and suggestive of subclavian
(or innominate) artery stenosis. Patients with PAD are at increased
risk of subclavian artery stenosis.^60–62^ Measuring blood pressure in
both arms identifies the arm with the highest systolic pressure, a
requirement for accurate measurement of the ABI.^27^ Identification of unequal
blood pressures in the arms also allows for more accurate
measurement of blood pressure in the treatment of hypertension (ie,
blood pressure is taken at the arm with higher measurements).
Although a difference in arm systolic pressures of >15 to 20
mm Hg suggests subclavian (or innominate) artery stenosis, in the
absence of symptoms (eg, arm claudication or symptoms of vertebral
artery steal), no further imaging or intervention is warranted.


## 3. Diagnostic Testing for the Patient with Suspected Lower Extremity PAD (Claudication or CLI)

### 3.1. Resting ABI for Diagnosing PAD: Recommendations


**Figure 1:**
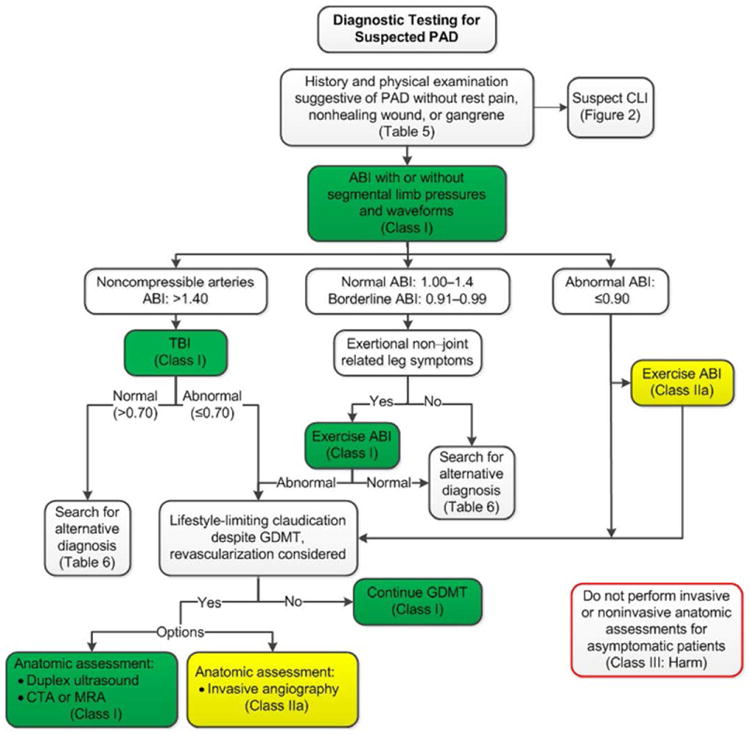
Diagnostic Testing for Suspected PAD Colors correspond to Class of Recommendation in Table 1. ABI indicates ankle-brachial index; CLI, critical limb ischemia; CTA, computed tomography angiography; GDMT, guideline-directed management and therapy; MRA, magnetic resonance angiography; PAD, peripheral artery disease; and TBI, toe-brachial index.

**Figure 2:**
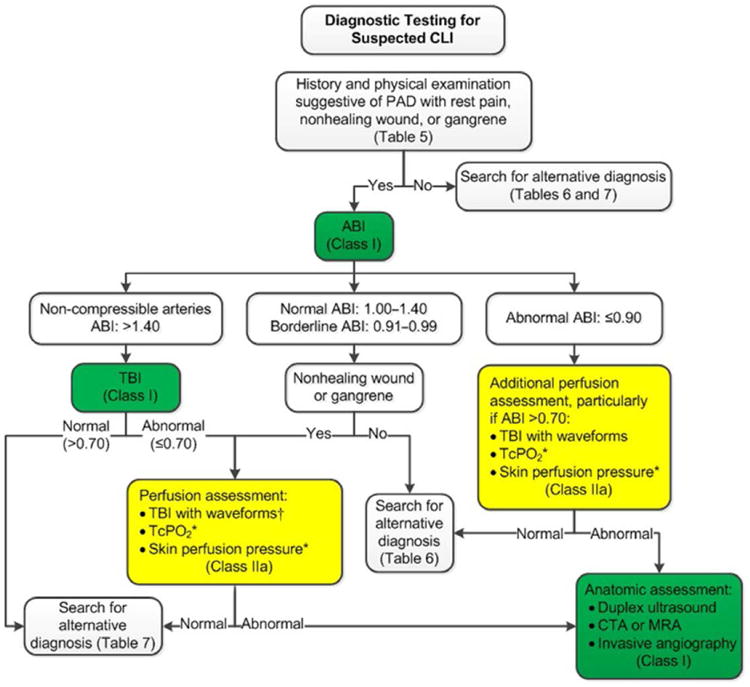
Diagnostic Testing for Suspected CLI Colors correspond to Class of Recommendation in Table 1. *Order based on expert consensus. †TBI with waveforms, if not already performed. ABI indicates ankle-brachial index; CLI, critical limb ischemia; CTA, computed tomography angiography; MRA, magnetic resonance angiography; TcPO_2_, transcutaneous oxygen pressure; and TBI, toe-brachial index.

**Recommendations for
Resting ABI for Diagnosing PAD**
**COR****LOE****Recommendations**
**I****B-NR****In patients with history or
physical examination findings suggestive of PAD (**Table 5**), the resting
ABI, with or without segmental pressures and waveforms, is
recommended to establish the diagnosis.**^64–69^
See Online Data Supplement 4.The resting ABI is obtained by
measuring systolic blood pressures at the arms (brachial arteries)
and ankles (dorsalis pedis and posterior tibial arteries) in the
supine position by using a Doppler device. The ABI of each leg is
calculated by dividing the higher of the dorsalis pedis or posterior
tibial pressure by the higher of the right or left arm blood
pressure.^27^ In patients with a history or physical
examination suggestive of PAD, the ABI has good validity as a
first-line test in the diagnosis of PAD, as shown by vascular
imaging, with sensitivities ranging from 68% to 84%
and specificities from 84% to 99%.^64–69^ Segmental lower extremity
blood pressures and Doppler or plethysmographic waveforms (pulse
volume recordings) can be used to localize anatomic segments of
disease (eg, aortoiliac, femoropopliteal, infrapopliteal).^34,70,71^
**I****C-LD****Resting ABI results should be
reported as abnormal (ABI ≤0.90), borderline (ABI
0.91–0.99), normal (1.00–1.40), or
noncompressible (ABI >1.40).**^27,67–69,72^
See Online Data Supplement 4.Standardized reporting improves
communication among healthcare providers. Calculated ABI values
should be recorded to 2 decimal places. Patients with ABI
≤0.90 are diagnosed with PAD.^67–69^ Those with ABI 0.91 to 0.99
may possibly have PAD and should undergo exercise ABI, if the
clinical suspicion of PAD is significant (Tables 4 and 5).^73,74^
Values >1.40 indicate that the arteries were not able to be
compressed, which is more common among individuals with diabetes
mellitus and/or advanced chronic kidney disease. In the setting of
noncompressible ABI values, additional imaging can be used to
diagnose PAD if the clinical suspicion is significant (Figures 1 and 2).^72^ These cutpoints for ABI interpretation have
been previously proposed and represent a reasonable standardized
categorization.^27^
**IIa****B-NR****In patients at increased risk of
PAD (**Table 4**) but without history or physical examination
findings suggestive of PAD (**Table 5**), measurement of the
resting ABI is reasonable.**^54,55,75–97^
See Online Data Supplements 3 and 4.The ABI test is noninvasive, is simple
to perform, and has minimal risks, making it suitable for use in
asymptomatic individuals. Previous studies have demonstrated a
significant prevalence of abnormal resting ABI among asymptomatic
patients with risk factors for PAD.^55,79,95^ A
significant body of evidence demonstrates that patients with an
abnormal ABI who are asymptomatic have poorer cardiovascular
morbidity and mortality outcomes than do patients with normal
ABI.^79–87^ While there is no conclusive evidence that
aspirin treatment changes cardiovascular or limb outcomes in this
population, in 1 cohort study of 5480 patients with asymptomatic
PAD, statin treatment improved cardiovascular outcomes.^75–78,96^
There is also evidence that
asymptomatic patients with a low resting ABI have a poorer
functional status and a more rapid rate of functional decline than
do patients with a normal ABI.^54,88–92^ Although physical activity has been shown to be
associated with improvement in functional status in patients with
asymptomatic PAD,^93,94^
the benefit of resting ABI testing to identify asymptomatic patients
who are at increased risk of functional decline and may benefit from
structured exercise programs remains to be determined.
**III: No Benefit****B-NR****In patients not at increased risk
of PAD (**Table 4**) and without history or physical examination
findings suggestive of PAD (**Table 5**), the ABI is not
recommended.**^95,98^
See Online Data Supplement 4.The prevalence of PAD among individuals
without risk factors for atherosclerosis and who are <50
years of age is low. Data from population-based cohort studies have
demonstrated a low prevalence (approximately 1%) of abnormal
resting ABI among individuals <50 years of age.^95,98^ In the NHANES (National
Health and Nutrition Study), approximately 95% of
participants with an abnormal resting ABI had at least 1 risk factor
for atherosclerosis.^95^ The yield of ABI testing among younger,
asymptomatic individuals without risk factors for atherosclerosis is
low, and these patients should not be routinely tested for
PAD.^95,98^


### 3.2. Physiological Testing: Recommendations


**Recommendations for
Physiological Testing**
**COR****LOE****Recommendations**
**I****B-NR****Toe-brachial index (TBI) should
be measured to diagnose patients with suspected PAD when the ABI
is greater than 1.40.**^72,99–102^
See Online Data Supplement 5.TBI is a noninvasive test that is
useful to evaluate for PAD in patents with noncompressible arteries,
which cause an artificial elevation of the ABI.^99,100,102,103^ A TBI ≤0.70 is abnormal and diagnostic
of PAD because the digital arteries are rarely
noncompressible.^99–102,104,105^ Patients with longstanding diabetes
mellitus^72,101^ or advanced
chronic kidney disease^106^ have a high incidence of noncompressible
arteries. Therefore, TBI assessment allows for the diagnosis of PAD
in these patients with noncompressible arteries who have history or
physical examination findings suggestive of PAD (Figure 1).
**I****B-NR****Patients with exertional
non–joint-related leg symptoms and normal or borderline
resting ABI (>0.90 and ≤1.40) should undergo
exercise treadmill ABI testing to evaluate for
PAD.**^71,74,107–110^
See Online Data Supplement 5.Exercise treadmill ABI testing is
important to objectively measure symptom limitations and diagnose
PAD.^71,74,107–110^ It is useful in
establishing the diagnosis of lower extremity PAD in the symptomatic
patient when resting ABIs are normal or borderline and to
differentiate claudication from pseudoclaudication in individuals
with exertional leg symptoms. If the post-exercise treadmill ABI is
normal, alternative causes of leg pain are considered (Table 6). If a treadmill is not
available, the pedal plantarflexion ABI test is a reasonable
alternative because the results correlate well with treadmill ABIs
(Figure 1).^111^
**IIa****B-NR****In patients with PAD and an
abnormal resting ABI (≤0.90), exercise treadmill ABI
testing can be useful to objectively assess functional
status.**^71,74,107–110^
See Online Data Supplement 5.In patients with PAD, exercise
treadmill ABI testing can objectively assess symptoms, measure
change in ABI in response to exercise, and assess functional
status^71,74,107–110^ (Figure 1). It can be useful to correlate
exertional lower extremity symptoms to a decline in ABI after
treadmill exercise. Exercise treadmill ABI testing can document the
magnitude of symptom limitation in patients with PAD and provide
objective data that can demonstrate the safety of exercise and help
to individualize exercise prescriptions in patients with PAD before
initiation of a formal program of structured exercise training.
Exercise ABI may also be used to objectively measure the functional
improvement obtained in response to claudication treatment (eg,
structured exercise program or revascularization). Administration of
a 6-minute walk test in a corridor is a reasonable alternative to
treadmill ABI testing for assessment of functional status.^54^
**IIa****B-NR****In patients with normal
(1.00–1.40) or borderline (0.91–0.99) ABI in the
setting of nonhealing wounds or gangrene, it is reasonable to
diagnose CLI by using TBI with waveforms, transcutaneous oxygen
pressure (TcPO_2_), or skin perfusion pressure
(SPP).**^112–116^
See Online Data Supplement 5.The toe pressure and TBI may be
discordant with the ABI 0.90 to 1.40 in some patients with diabetes
mellitus and a nonhealing wound (Figure 2).^115,116^ A TBI ≤0.70 is considered diagnostic
of PAD.^101,104,105^ Doppler or plethysmographic
waveforms taken at the toe supplement the toe pressure and TBI
measurement and may be severely dampened in the setting of CLI. The
likelihood of wound healing decreases with toe pressure <30
mm Hg.^100^
Perfusion assessment measures (ie, TBI with waveforms,
TcPO_2_, SPP) are obtained in a warm room to prevent
arterial vasoconstriction in response to the cold. TcPO_2_
measurements are performed with a standardized protocol and are
taken at multiple sites.^117^ Correlation between TBI, TcPO_2_,
and SPP has been reported.^113^ TcPO_2_ >30 mm Hg has
been used to predict ulcer healing.^118^ SPP ≥30 to 50 mm Hg
is associated with increased likelihood of wound healing.^113^ If perfusion
measures are normal or only mildly impaired, alternative causes of
the nonhealing wounds are considered (Table 7). TcPO_2_ and SPP can
be used in angiosome-targeted assessment for
revascularization.^119^
**IIa****B-NR****In patients with PAD with an
abnormal ABI (≤0.90) or with noncompressible arteries
(ABI >1.40 and TBI ≤0.70) in the setting of
nonhealing wounds or gangrene, TBI with waveforms,
TcPO_2_, or SPP can be useful to evaluate local
perfusion.**^112–116^
See Online Data Supplement 5.Perfusion assessment measures (eg, TBI
with waveforms, TcPO_2_, SPP) can be useful when the ABI is
only mildly reduced (eg, ABI 0.70–0.90) to determine whether
factors other than PAD may be contributing to impaired wound healing
(Figure 2). These perfusion
assessment measures are obtained in a warm room to prevent arterial
vasoconstriction in response to the cold. TcPO_2_
measurements are performed with a standardized protocol and are
taken at multiple sites.^117^ The likelihood of wound healing decreases
with toe pressure <30 mm Hg.^100^ There is correlation
between TBI, TcPO_2_, and SPP. TcPO_2_ >30
mm Hg has been used to predict ulcer healing.^118^ SPP ≥30 to 50 mm Hg
is associated with increased likelihood of wound healing.^113^ TcPO_2_
and SPP can be used in angiosome-targeted assessment for
revascularization.^119^ Additional perfusion assessment may also
be useful for patients with nonhealing wounds or gangrene who have
noncompressible arteries (ABI >1.40) but who have a
diagnosis of PAD that is based on an abnormal TBI (ABI
≤0.70).


### 3.3. Imaging for Anatomic Assessment: Recommendations


**Recommendations for
Imaging for Anatomic Assessment**
**COR****LOE****Recommendations**
**I****B-NR****Duplex ultrasound, computed
tomography angiography (CTA), or magnetic resonance angiography
(MRA) of the lower extremities is useful to diagnose anatomic
location and severity of stenosis for patients with symptomatic
PAD in whom revascularization is considered.**^118,120–122^
See Online Data Supplement 6.For symptomatic patients in whom
ABI/TBI confirms PAD and in whom revascularization is considered,
additional imaging with duplex ultrasonography, CTA, or MRA is
useful to develop an individualized treatment plan, including
assistance in selection of vascular access sites, identification of
significant lesions, and determination of the feasibility of and
modality for invasive treatment. All 3 of these noninvasive imaging
methods have good sensitivity and specificity as compared with
invasive angiography.^118,120–122^ Renal function does not affect the safety
of duplex ultrasonography, although duplex offers lower spatial
resolution than CTA and MRA in the setting of arterial
calcification. The tomographic data from CTA and MRA afford
3-dimensional reconstruction of the vessels examined. The iodinated
contrast used in CTA confers risk of contrast-induced nephropathy
and (rarely) severe allergic reaction^123,124^; CTA uses ionizing radiation. MRA does not
use ionizing radiation; however, gadolinium contrast used frequently
in MRA studies confers risk of nephrogenic systemic sclerosis for
patients with advanced renal dysfunction and is therefore
contraindicated in this population.^125^ The choice of the
examination should be determined in an individualized approach to
the anatomic assessment for each patient, including
risk–benefit assessment of each study type. If these
noninvasive tests are nondiagnostic, then invasive angiography may
be required to delineate anatomy and plan revascularization.
**I****C-EO****Invasive angiography is useful
for patients with CLI in whom revascularization is
considered.**
N/ABy definition, CLI results from
extensive PAD that limits tissue perfusion. Because timely diagnosis
and treatment are essential to preserve tissue viability in CLI, it
is often most effective and expeditious to pursue invasive
angiography with endovascular revascularization directly, without
delay and potential risk of additional noninvasive imaging.
**IIa****C-EO****Invasive angiography is
reasonable for patients with lifestyle-limiting claudication
with an inadequate response to GDMT for whom revascularization
is considered.**
N/AFor patients with lifestyle-limiting
claudication despite GDMT (including structured exercise therapy)
for whom revascularization is being considered, proceeding directly
to invasive angiography for anatomic assessment and to determine
revascularization strategy is reasonable. In certain clinical
settings, noninvasive imaging studies for anatomic assessment (ie,
duplex ultrasound, CTA, or MRA) may not be available because of lack
of local resources or expertise. In addition, there are clinical
scenarios in which noninvasive studies for anatomic assessment may
be perceived to confer greater risk to the patient than invasive
angiography (eg, patient with advanced chronic kidney disease for
whom contrast dose for invasive angiography would be lower than that
required for CTA).
**III: Harm****B-R****Invasive and noninvasive
angiography (ie, CTA, MRA) should not be performed for the
anatomic assessment of patients with asymptomatic
PAD.**^123,124,126^
See Online Data Supplements 6 and 7.Angiography, either noninvasive or
invasive, should not be performed for the anatomic assessment of
patients with PAD without leg symptoms because delineation of
anatomy will not change treatment for this population. This lack of
benefit occurs in the setting of risk of contrast-induced
nephropathy, patient discomfort, and allergic reactions.^123,124,126^ This recommendation does not address
assessment of lower extremity aneurysmal disease or
nonatherosclerotic causes of arterial disease, which is beyond the
scope of this document.


## 4. Screening for Atherosclerotic Disease in Other Vascular Beds for the Patient with PAD

### 4.1. Abdominal Aortic Aneurysm: Recommendation


**Recommendation for
Abdominal Aortic Aneurysm**
**COR****LOE****Recommendation**
**IIa****B-NR****A screening duplex ultrasound for
abdominal aortic aneurysm (AAA) is reasonable in patients with
symptomatic PAD.**^127–129^
See Online Data Supplement 8.PAD has been recognized as a risk
factor for AAA. In observational studies, the prevalence of AAA
(aortic diameter ≥3 cm) was higher in patients with
symptomatic PAD than in the general population^127,129^ and in a population of patients with
atherosclerotic risk factors.^128^ The prevalence of AAA among patients with
PAD increased with age, beginning in patients ≥55 years of
age, and was highest in patients ≥75 years of age.^129^ There are no
data on AAA screening in patients with asymptomatic PAD. This
recommendation refers to screening patients with symptomatic PAD for
AAA regardless of patient age, sex, smoking history, or family
history of AAA. Recommendations for screening the general population
with risk factors for AAA (based on age, sex, smoking history, and
family history) have been previously published.^9^


### 4.2. Screening for Asymptomatic Atherosclerosis in Other Arterial Beds (Coronary, Carotid, and Renal Arteries)

The prevalence of atherosclerosis in the coronary, carotid, and renal arteries is higher in patients with PAD than in those without pad.^128,130–135^ However, intensive atherosclerosis risk factor modification in patients with PAD is justified regardless of the presence of disease in other arterial beds. Thus, the only justification for screening for disease in other arterial beds is if revascularization results in a reduced risk of myocardial infarction (MI), stroke, or death, and this has never been shown. Currently, there is no evidence to demonstrate that screening all patients with PAD for asymptomatic atherosclerosis in other arterial beds improves clinical outcome. Intensive treatment of risk factors through GDMT is the principle method for preventing adverse cardiovascular ischemic events from asymptomatic disease in other arterial beds.

## 5. Medical Therapy for the Patient with PAD

Patients with PAD should receive a comprehensive program of GDMT, including structured exercise and lifestyle modification, to reduce cardiovascular ischemic events and improve functional status. Smoking cessation is a vital component of care for patients with PAD who continue to smoke. A guideline-based program of pharmacotherapy to reduce cardiovascular ischemic events and limb-related events should be prescribed for each patient with PAD and is customized to individual risk factors, such as whether the patient also has diabetes mellitus. Previous studies have demonstrated that patients with PAD are less likely to receive GDMT than are patients with other forms of cardiovascular disease, including coronary artery disease (CAD).^136–138^

### 5.1. Antiplatelet Agents: Recommendations


**Recommendations for
Antiplatelet Agents**
**COR****LOE****Recommendations**
**I****A****Antiplatelet therapy with aspirin
alone (range 75–325 mg per day) or clopidogrel alone (75
mg per day) is recommended to reduce MI, stroke, and vascular
death in patients with symptomatic PAD.**
^139–142^
See Online Data Supplement 13.The effect of antiplatelet therapy on
cardiovascular events has been systematically reviewed by the
Antithrombotic Trialists' Collaboration.^139^ Of note, this
meta-analysis included studies of antiplatelet agents other than
aspirin or clopidogrel. Among patients with symptomatic PAD treated
with antiplatelet therapy, there was a 22% odds reduction
for cardiovascular events, including MI, stroke, or vascular
death.^139^
Symptomatic patients with lower extremity PAD included both those
with claudication and those with prior lower extremity
revascularization. The Antithrombotic Trialists'
Collaboration meta-analysis also compared the efficacy of different
doses of aspirin.^139^ The proportional reduction in vascular events
was 32% with 75 to 150 mg daily, 26% with 160 to 325
mg daily, and 19% with 500 to 1500 mg daily, whereas there
was a significantly smaller (13%) reduction in
cardiovascular events in patients being treated with <75 mg
of aspirin per day.^139^ CLIPS (Critical Leg Ischaemia Prevention
Study) demonstrated a benefit of aspirin (100 mg daily) compared
with placebo in preventing vascular events, but the study was too
small to derive meaningful conclusions.^140^ A meta-analysis of trials
of aspirin (alone or in combination with dipyridamole) for
prevention of cardiovascular events in patients with PAD found a
non–statistically significant reduction in the primary
endpoint of cardiovascular death, MI, and stroke and a statistically
significant reduction in the secondary endpoint of nonfatal stroke
with aspirin versus placebo.^141^ The CAPRIE (Clopidogrel Versus Aspirin in
Patients at Risk of Ischemic Events) trial demonstrated a benefit of
clopidogrel as compared with aspirin in cardiovascular risk
reduction and bleeding events in a population of patients with
symptomatic atherosclerotic vascular disease, including a subgroup
of patients with symptomatic PAD.^142^
**IIa****C-EO****In asymptomatic patients with PAD
(ABI ≤0.90), antiplatelet therapy is reasonable to
reduce the risk of MI, stroke, or vascular death.**
See Online Data Supplement 13.Patients with PAD (ie, ABI
≤0.90) who do not have claudication may have leg symptoms
atypical for claudication or may be too functionally limited to
allow for adequate leg symptom assessment. Patients with PAD without
claudication are at increased cardiovascular risk.^79^ Subgroup analysis
in a trial evaluating asymptomatic patients did not show an effect
of aspirin in patients with an abnormally low ABI (<0.80 or
≤0.90).^76^ However, the trial was not powered to
analyze subgroups, and the uncertainty of the result does not rule
out the possibility that aspirin could provide benefit in such
patients, especially in those at increased risk of cardiovascular
events. Another trial that included asymptomatic patients was too
small to derive meaningful conclusions.^140^
**IIb****B-R****In asymptomatic patients with
borderline ABI (0.91–0.99), the usefulness of
antiplatelet therapy to reduce the risk of MI, stroke, or
vascular death is uncertain.**^75,76^
See Online Data Supplement 13.In asymptomatic patients with an
abnormal or borderline ABI, 2 RCTs found that aspirin had no effect
in reducing cardiovascular events^75,76^ and might increase bleeding.^76^ However, the
trials were not powered to examine patients with borderline ABI
separately. Given that cardiovascular risk is lower in patients with
borderline ABI than in those with abnormal ABI,^80^ it would be unlikely that
aspirin would have a meaningful effect in this subgroup when there
was no evidence of an effect in the total trial populations.
**IIb****B-R****The effectiveness of dual
antiplatelet therapy (DAPT) (aspirin and clopidogrel) to reduce
the risk of cardiovascular ischemic events in patients with
symptomatic PAD is not well established.**^143,144^
See Online Data Supplement 13.Based on findings from a subset of
patients with PAD in the CHARISMA (Clopidogrel for High
Atherothrombotic Risk and Ischemic Stabilization, Management, and
Avoidance) trial, DAPT with aspirin plus clopidogrel may be
considered for patients with PAD at particularly high risk of
cardiovascular ischemic events who are not at high risk of
bleeding.^143,144^ Currently, there are sparse data on newer
P2Y_12_ antagonists for PAD. There is uncertainty about
the net benefit of long-term DAPT for patients with
PAD—specifically the balance of risks of cardiovascular
ischemic events versus major bleeding. Additional clinical trials
are needed in the population with PAD. Refer to the DAPT guideline
focused update for DAPT recommendations specifically for
CAD.^20^
**IIb****C-LD****DAPT (aspirin and clopidogrel)
may be reasonable to reduce the risk of limb-related events in
patients with symptomatic PAD after lower extremity
revascularization.**^145–148^
See Online Data Supplements 13 and 14.There are sparse data on DAPT after
lower extremity revascularization. Still, DAPT is prescribed in up
to 55% of patients after endovascular revascularization for
CLI.^146^
One small RCT of aspirin or aspirin plus clopidogrel in patients
undergoing endovascular revascularization demonstrated that patients
with DAPT had fewer repeat revascularization procedures for clinical
symptoms.^145^ A subsequent small RCT of aspirin plus placebo
or aspirin plus clopidogrel in patients after endovascular
revascularization also showed a decrease in the need for repeat
revascularization at 6 months in patients receiving
clopidogrel.^147^ An RCT of aspirin plus placebo or aspirin plus
clopidogrel in patients who underwent below-knee bypass graft showed
a decrease in limb-related events only in the prespecified subgroup
of patients with prosthetic bypass grafts.^148^ Refer to the DAPT guideline
focused update for DAPT recommendations specifically for
CAD.^20^
**IIb****B-R****The overall clinical benefit of
vorapaxar added to existing antiplatelet therapy in patients
with symptomatic PAD is uncertain.**^149–152^
See Online Data Supplement 13.This novel antagonist of
protease-activated receptor-1 added to existing antiplatelet therapy
reduced the risk of cardiovascular ischemic events in patients with
atherosclerosis who were receiving standard therapy in an
RCT.^150,151^ However, it also
increased the risk of moderate or severe bleeding. Although the
cardiovascular benefit was not demonstrated in the subgroup with
symptomatic PAD, there was a reduction in limb-related events with
vorapaxar, specifically in acute limb ischemia (ALI) and peripheral
revascularization.^149,152^ More than half of ALI events in the PAD subset
were due to thrombosis of lower extremity bypass grafts.^149^ Unfortunately,
the benefit in limb events in patients with PAD was accompanied by
an increased risk of bleeding.^149,152^ Therefore, the overall clinical benefit of
vorapaxar in patients with PAD is uncertain.


### 5.2. Statin Agents: Recommendation


**Recommendation for
Statin Agents**
**COR****LOE****Recommendation**
**I****A****Treatment with a statin
medication is indicated for all patients with
PAD.**^96,153–157^
See Online Data Supplements 15 and 16.Statin therapy improves both
cardiovascular and limb outcomes in patients with PAD.^157^ In a subgroup of
6748 patients with PAD in the HPS (Heart Protection Study),
simvastatin 40 mg daily reduced the rate of first major vascular
event by 22% relative to placebo.^155^
In a multinational registry, statin use
among patients with PAD reduced 4-year adverse limb-related events
(ie, worsening claudication, new CLI, new lower extremity
revascularization, new ischemic amputation) compared with no
statin.^153^ Use of simvastatin in the HPS reduced relative
risk of peripheral vascular events (including noncoronary
revascularization, aneurysm repair, major amputation, or PAD death)
compared with placebo.^155^ In Medicare patients undergoing lower
extremity revascularization, 1-year limb salvage rates were improved
among those receiving statin medication.^154^ In a multicenter RCT, use
of atorvastatin 80 mg daily improved pain-free walking time and
community-based walking at 12 months compared with
placebo.^156^ In 1 cohort study of 5480 patients with
asymptomatic PAD, statin treatment improved cardiovascular
outcomes.^96^ Guidelines for dosing of statin medications
have been previously published.^24^


### 5.3. Antihypertensive Agents: Recommendations


**Recommendations for
Antihypertensive Agents**
**COR****LOE****Recommendations**
**I****A****Antihypertensive therapy should
be administered to patients with hypertension and PAD to reduce
the risk of MI, stroke, heart failure, and cardiovascular
death.**^158–162^
See Online Data Supplements 17 and 18.Treatment of elevated blood pressure is
indicated to lower the risk of cardiovascular events.^162^ Target blood
pressure and selection of antihypertensive therapy should be
consistent with current published guidelines for hypertension
management. Concerns have been raised that antihypertensive therapy
may reduce limb perfusion. However, multiple studies have
demonstrated that blood pressure treatment, including the use of
beta blockers, does not worsen claudication symptoms or impair
functional status in patients with PAD.^163–165^ There is no evidence that
one class of antihypertensive medication or strategy is superior for
blood pressure lowering in PAD.^158,166,167^ An updated multisocietal guideline on the
management of high blood pressure is anticipated in 2017.
**IIa****A****The use of angiotensin-converting
enzyme inhibitors or angiotensin-receptor blockers can be
effective to reduce the risk of cardiovascular ischemic events
in patients with PAD.**^161,168,169^
See Online Data Supplement 17.The effect of ramipril versus placebo
on cardiovascular events was studied in high-risk patients free of
heart failure in the HOPE (Heart Outcomes Prevention Evaluation)
trial.^168,169^ Patients were
normotensive on average at the time of enrollment. In a subgroup of
4051 patients with PAD, ramipril reduced the risk of MI, stroke, or
vascular death by 25%, similar to the efficacy in the entire
study population.^168,169^ The efficacy was similar in patients with PAD
with symptomatic disease and asymptomatic low ABI.^168^ ONTARGET
(Ongoing Telmisartan Alone and in Combination With Ramipril Global
Endpoint Trial) compared telmisartan, ramipril, and combination
therapy in patients with cardiovascular disease, including PAD,
and/or diabetes mellitus.^161^ All 3 treatments had similar
cardiovascular event rates with higher rates of adverse events
(including hypotension, syncope, and renal failure) in the
combination-therapy group. The efficacy of telmisartan was similar
in the subgroup of 3468 patients with PAD, which supports the use of
angiotensin-receptor blockers as an alternative to
angiotensin-converting enzyme inhibitors.^161^ The effect of
angiotensin-receptor blockers in asymptomatic PAD has not been
studied.


### 5.4. Smoking Cessation: Recommendations


**Recommendations for
Smoking Cessation**
**COR****LOE****Recommendations**
**I****A****Patients with PAD who smoke
cigarettes or use other forms of tobacco should be advised at
every visit to quit.**^170–172^
See Online Data Supplements 19 and 20.Tobacco use is a strong risk factor for
the development and progression of PAD.^173,174^ Sparse evidence exists with regard to the
association of novel tobacco product use, including electronic
cigarettes, and PAD.^175^ Observational studies suggest that smoking
cessation is associated with lower rates of cardiovascular ischemic
events, limb-related events, bypass graft failure, amputation, and
death in patients with PAD.^172,176–178^ Clinician advice increases quit rates,
which supports simple provider-based measures as a component of
smoking cessation programs.^22,171,179^
**I****A****Patients with PAD who smoke
cigarettes should be assisted in developing a plan for quitting
that includes pharmacotherapy (ie, varenicline, bupropion,
and/or nicotine replacement therapy) and/or referral to a
smoking cessation program.**^170,180–182^
See Online Data Supplements 19 and 20.Coordinated smoking cessation
interventions that include nonpharmacological and pharmacological
approaches have the greatest efficacy. An RCT of a follow-up program
and smoking cessation medications provided to hospitalized patients,
including those with PAD, demonstrated a modest increase in quit
rates.^181^
In an RCT of patients with PAD specifically, a comprehensive smoking
cessation program combining counseling and pharmacological agents
increased the rates of smoking cessation to 21.3%, compared
with 6.8% with standard advice.^170^ Three pharmacological
approaches (ie, varenicline, bupropion, and nicotine replacement
therapy) used alone or in combination all increase smoking cessation
rates.^179,180,182^ Two meta-analyses of RCTs
of smoking cessation medications showed no evidence of increased
cardiovascular event rates with nicotine replacement, bupropion, or
varenicline.^183,184^ Sparse data suggest that electronic cigarettes
have no benefit on smoking cessation rates.^179^
**I****B-NR****Patients with PAD should avoid
exposure to environmental tobacco smoke at work, at home, and in
public places.**^185,186^
See Online Data Supplement 20.Passive smoke exposure has been
associated with the development of PAD.^186^ Observational studies have
shown lower cardiovascular and cerebrovascular event rates in the
general population after enactment of smoke-free
legislation.^185^ The effects of avoidance of passive smoke
exposure on limb-related events are not known.


### 5.5. Glycemic Control: Recommendations


**Recommendations for
Glycemic Control**
**COR****LOE****Recommendations**
**I****C-EO****Management of diabetes mellitus
in the patient with PAD should be coordinated between members of
the healthcare team.**
N/ADiabetes mellitus is an important risk
factor for the development of PAD.^187^ Furthermore, the presence
of diabetes mellitus increases the risk of adverse outcomes among
patients with PAD, including progression to CLI, amputation, and
death.^188,189^ A comprehensive
care plan for patients with PAD and diabetes mellitus is important
and may include diet and weight management, pharmacotherapy for
glycemic control and management of other cardiovascular risk
factors, and foot care and ulcer prevention.^25,190^ Guidelines for glycemic control among
patients with diabetes mellitus and atherosclerotic vascular disease
have been previously published.^25,29^
Regular follow-up with and communication among the patient's
healthcare providers, including vascular specialists and diabetes
care providers (eg, primary care physicians, endocrinologists)
constitute an important component of care for patients with PAD and
diabetes mellitus.
**IIa****B-NR****Glycemic control can be
beneficial for patients with CLI to reduce limb-related
outcomes.**^191,192^
See Online Data Supplement 22.In a cohort of 1974 participants with
diabetes mellitus from the Strong Heart Study, compared with
patients without PAD, patients with PAD and a Hg A1c level
<6.5% had lower age-adjusted odds of major
amputation compared to patients with PAD and hemoglobin A1c
6.5% to 9.5% and hemoglobin A1c
>9.5%.^188^ Glycemic control is particularly important
for patients with PAD and diabetes mellitus who have CLI.
Single-center observational studies have demonstrated improved
limb-related outcomes, including lower rates of major amputation and
improved patency after infrapopliteal intervention, among patients
with CLI who have more optimized glycemic control parameters
compared with patients with inferior glycemic control.^191,192^


### 5.6. Oral Anticoagulation: Recommendations


**Recommendations for
Oral Anticoagulation**
**COR****LOE****Recommendations**
**IIb****B-R****The usefulness of anticoagulation
to improve patency after lower extremity autogenous vein or
prosthetic bypass is uncertain.**^193–195^
See Online Data Supplements 23 and 24.Two RCTs evaluating the effectiveness
of oral anticoagulation (warfarin) in improving lower extremity
bypass patency demonstrated improved patency among the subgroup of
patients with autogenous vein bypass grafts.^193,194^ However, a Cochrane systematic review
showed no patency benefit with the use of anticoagulation compared
with antiplatelet therapy.^195^ All RCTs and observational studies
evaluating the effect of anticoagulants on bypass patency
demonstrated increased bleeding complications associated with
anticoagulant use. One RCT evaluating the effectiveness of oral
anticoagulation (warfarin) in addition to aspirin in improving lower
extremity bypass patency demonstrated improved patency in a subgroup
of patients with 6-mm polytetrafluoroethylene (known as PTFE) bypass
graft.^196^
Randomization to anticoagulation plus aspirin was associated with
increased risk of death and major hemorrhage versus aspirin
alone.
**III: Harm****A****Anticoagulation should not be
used to reduce the risk of cardiovascular ischemic events in
patients with PAD.**^194,196–198^
See Online Data Supplements 23 and 24RCTs and observational studies have
uniformly demonstrated that oral anticoagulation therapy aimed at
decreasing major cardiovascular ischemic events provided no benefit
and resulted in increased morbidity.^194,196–198^ In the WAVE (Warfarin Antiplatelet
Vascular Evaluation) trial of patients with atherosclerotic vascular
disease, including PAD, there was no difference in cardiovascular
ischemic events among patients randomized to oral anticoagulation
and antiplatelet therapy versus antiplatelet therapy
alone.^198^
In addition, there was an increase in bleeding endpoints including
life-threatening and intracranial bleeding.^198^ One RCT demonstrated
increased death rate among patients randomized to warfarin plus
aspirin versus aspirin alone after lower extremity bypass
grafting.^196^


### 5.7. Cilostazol: Recommendation


**Recommendation for
Cilostazol**
**COR****LOE****Recommendation**
**I****A****Cilostazol is an effective
therapy to improve symptoms and increase walking distance in
patients with claudication.**^199,200^
See Online Data Supplement 25.In a Cochrane review including 15
double-blind RCTs with a total of 3718 participants, cilostazol was
associated with improvement in claudication symptoms but no changes
in cardiovascular deaths or QoL when compared with
placebo.^199^ In 1 RCT, cilostazol was more effective than
pentoxifylline or placebo.^200^ Side effects include headache, abnormal
stool (diarrhea), dizziness, and palpitations. Cilostazol is
contraindicated in patients with congestive heart failure.^201^ In 1 trial,
20% of patients discontinued cilostazol within 3
months.^202^


### 5.8. Pentoxifylline: Recommendation


**Recommendation for
Pentoxifylline**
**COR****LOE****Recommendation**
**III: No Benefit****B-R****Pentoxifylline is not effective
for treatment of claudication.**^200,203^
See Online Data Supplement 26.In a Cochrane review of 24 studies with
3377 participants, there was large variability in study design and
results between individual studies, and therefore the
review's effectiveness was unclear.^203^ Pentoxifylline was shown to
be generally well tolerated.^203^ In a multicenter RCT of pentoxifylline,
cilostazol, or placebo for patients with moderate-to-severe
claudication, there was no difference between pentoxifylline and
placebo in the primary endpoint of maximal walking
distance.^200^ Therefore, pentoxifylline is not recommended
as treatment for claudication.


### 5.9. Chelation Therapy: Recommendation


**Recommendation for
Chelation Therapy**
**COR****LOE****Recommendation**
**III: No Benefit****B-R****Chelation therapy (eg,
ethylenediaminetetraacetic acid) is not beneficial for treatment
of claudication.**^204^
See Online Data Supplement 27.In a Cochrane review of 5 studies with
260 participants, chelation therapy showed no significant difference
in symptoms (maximal and pain-free walking distance) compared with
placebo.^204^


### 5.10. Homocysteine Lowering: Recommendation


**Recommendation for
Homocysteine Lowering**
**COR****LOE****Recommendation**
**III: No Benefit****B-R****B-complex vitamin supplementation
to lower homocysteine levels for prevention of cardiovascular
events in patients with PAD is not recommended.**^205–207^
See Online Data Supplements 28 and 29.Although patients with PAD have been
shown to have increased plasma homocysteine levels compared with
patients without PAD, there is no evidence that B-complex vitamin
supplementation improves clinical outcomes in patients with
PAD.^207^
The HOPE-2 trial randomized 5522 patients with atherosclerotic
vascular disease, including symptomatic PAD, or diabetes mellitus
with additional risk factors to receive folic acid/vitamin
B6/vitamin B12 or placebo.^205,206^ Despite lowering of homocysteine levels in the
vitamin supplementation arm, there was no improvement in the primary
endpoint of cardiovascular death, MI, or stroke.


### 5.11. Influenza Vaccination: Recommendation


**Recommendation for
Influenza Vaccination**
**COR****LOE****Recommendation**
**I****C-EO****Patients with PAD should have an
annual influenza vaccination.**
See Online Data Supplements 30 and 31.Observational studies have demonstrated
reduced cardiovascular event rates among patients with
cardiovascular disease who have received an influenza
vaccination.^30^ Two RCTs that enrolled patients with CAD
demonstrated a benefit of an influenza vaccination on the prevention
of cardiovascular events, particularly coronary ischemic
events.^208,209^ Although these trials did not specifically
enroll participants with PAD, a majority of patients with PAD also
have CAD.^30^ On the
basis of this evidence, an annual influenza vaccination is
recommended as a component of medical therapy for patients with
PAD.


## 6. Structured Exercise Therapy: Recommendations

Structured exercise therapy is an important element of care for the patient with PAD. Components of structured exercise programs for PAD are outlined in Table 8.


**Recommendations for
Structured Exercise Therapy**
**COR****LOE****Recommendations**
**I****A****In patients with claudication, a
supervised exercise program is recommended to improve functional
status and QoL and to reduce leg symptoms.**^36–38,40–46,48,210,211^
See Online Data Supplement 32.The data supporting the efficacy of
supervised exercise training as an initial treatment for claudication
continue to develop and remain convincing, building on many earlier
RCTs.^40–46,48,210,211^ Trials with long-term follow-up
from 18 months^37,38^ to 7 years^36^ have demonstrated a
persistent benefit of supervised exercise in patients with claudication.
Data also support a benefit of supervised exercise for patients with
symptomatic PAD and diabetes mellitus.^212^ The risk–benefit ratio
for supervised exercise in PAD is favorable, with an excellent safety
profile in patients screened for absolute contraindications to exercise
such as exercise-limiting cardiovascular disease, amputation or
wheelchair confinement, and other major comorbidities that would
preclude exercise.^36,39,49,213–216^ Despite the health benefits associated with
supervised exercise in patients with PAD, initiating and maintaining a
high level of adherence remain challenging. Frequent contact with
patients both when performing exercise in the supervised setting and at
home has been somewhat effective in promoting retention.^37,38^
**I****B-R****A supervised exercise program should
be discussed as a treatment option for claudication before possible
revascularization.**^36–38^
See Online Data Supplement 32.The CLEVER (Claudication: Exercise Versus
Endoluminal Revascularization) trial randomized patients with
symptomatic aortoiliac PAD and showed comparable benefits for supervised
exercise and stent revascularization at 6 and 18 months, with each
therapy being superior to optimal medical care.^37,38^ Overall, the safety profile for supervised
exercise was excellent. An RCT that compared 7-year effectiveness of
supervised exercise or endovascular revascularization in patients with
stable claudication with iliac or femoropopliteal disease found no
differences in improved walking and QoL outcomes.^36^ Although more secondary
interventions occurred in the exercise group, the total number of
interventions was greater in the endovascular revascularization group.
Collectively, these studies provide strong support for offering patients
a supervised exercise program for reducing claudication symptoms and for
improving functional status and QoL.
A 3-month RCT that compared percutaneous
transluminal angioplasty (PTA), supervised exercise, and combined
treatment for claudication found that both supervised exercise and PTA
improved clinical and QoL outcomes, whereas PTA plus supervised exercise
produced greater benefits than either therapy alone.^217^ The ERASE
(Endovascular Revascularization and Supervised Exercise) study
randomized participants with claudication to endovascular
revascularization plus supervised exercise or supervised exercise alone.
After 1 year, patients in both groups had significant improvements in
walking distances and health-related QoL, with greater improvements in
the combined-therapy group.^218^ Collectively, these studies support the
continued provision of supervised exercise to patients with
claudication, whether as a monotherapy or combined with
revascularization.
**IIa****A****In patients with PAD, a structured
community- or home-based exercise program with behavioral change
techniques can be beneficial to improve walking ability and
functional status.**^49,88,94,213^
See Online Data Supplement 32.Unstructured community-based or home-based
walking programs that consist of providing general recommendations to
patients with claudication to simply walk more are not
efficacious.^50^
Studies supporting structured community- or home-based programs for
patients with symptomatic PAD (claudication and/or leg symptoms atypical
for claudication) are more recent than studies supporting supervised
exercise programs, and have provided strong evidence in support of the
community- or home-based approach.^47,49,51,88,94,213^ For
example, the GOALS (Group Oriented Arterial Leg Study) trial^94^ included patients with
confirmed PAD with and without claudication (atypical lower extremity
symptoms or no symptoms) and showed increases in several parameters of
functional status for both of these patient cohort subgroups, versus
nonexercising controls, after 6 months,^88^ with improvement maintained at
12 months.^94^
As with supervised exercise programs,
despite proven benefit, initiating and maintaining a high level of
adherence to community- or home-based exercise programs remains
challenging. Studies that have incorporated behavioral change
techniques, such as health coaching and activity tracking used in
supervised settings, appear to reduce attrition and promote higher
levels of adherence, thereby improving functional and QoL outcomes, both
short term and long term.^49,88,94^
**IIa****A****In patients with claudication,
alternative strategies of exercise therapy, including upper-body
ergometry, cycling, and pain-free or low-intensity walking that
avoids moderate-to-maximum claudication while walking, can be
beneficial to improve walking ability and functional
status.**^39,215,219,220^
See Online Data Supplements 32 and 33.Protocols for exercise therapy for PAD
traditionally have recommended intermittent walking bouts to moderate or
higher pain levels interspersed with short periods of rest. Although
these protocols are efficacious, intolerance of pain may lead to poor
exercise adherence. An increasing number of studies have shown that
modalities of exercise that avoid claudication or walking performed at
intensities that are pain free or produce only mild levels of
claudication can achieve health benefits comparable to walking at
moderate or higher levels of claudication pain.^39,41,215,219–221^


## 7. Minimizing Tissue Loss in Patients with PAD: Recommendations


**Recommendations for
Minimizing Tissue Loss in Patients With PAD**
**COR****LOE****Recommendations**
**I****C-LD****Patients with PAD and diabetes
mellitus should be counseled about self–foot examination and
healthy foot behaviors.**^222,223^
See Online Data Supplement 34.Some RCTs have suggested that patient
education may help reduce the incidence of serious foot ulcers and lower
extremity amputations, but the quality of evidence supporting patient
education is low.^222^
Educational efforts generally include teaching patients about healthy
foot behaviors (eg, daily inspection of feet, wearing of shoes and
socks; avoidance of barefoot walking), the selection of proper footwear,
and the importance of seeking medical attention for new foot
problems.^223^
Educational efforts are especially important for patients with PAD who
have diabetes mellitus with peripheral neuropathy.
**I****C-LD****In patients with PAD, prompt
diagnosis and treatment of foot infection are recommended to avoid
amputation.**^224–228^
See Online Data Supplement 34.Foot infections (infection of any of the
structures distal to the malleoli) may include cellulitis, abscess,
fasciitis, tenosynovitis, septic joint space infection, and
osteomyelitis. Studies have investigated the accuracy of physical
findings for identification of infection and determining infection
severity and risk of amputation.^224–226^ Because of the consequences associated with
untreated foot infection—especially in the presence of
PAD—clinicians should maintain a high index of
suspicion.^228^
It is also recognized that the presence of diabetes mellitus with
peripheral neuropathy and PAD may make the presentation of foot
infection more subtle than in patients without these problems. Foot
infection should be suspected if the patient presents with local pain or
tenderness; periwound erythema; periwound edema, induration or
fluctuance; pretibial edema; any discharge (especially purulent); foul
odor; visible bone or a wound that probes-to-bone; or signs of a
systemic inflammatory response (including temperature
>38°C or <36°C, heart rate
>90/min, respiratory rate >20/min or Paco_2_
<32 mm Hg, white blood cell count >12 000 or
<4000/mcL or >10% immature forms).^226^ Probe-to-bone test
is moderately predictive for osteomyelitis but is not
pathognomonic.^227^
**IIa****C-LD****In patients with PAD and signs of
foot infection, prompt referral to an interdisciplinary care team
(**Table 9**) can
be beneficial.**^228–230^
See Online Data Supplement 34.The EuroDIALE (European Study Group on
Diabetes and the Lower Extremity) study demonstrated that the presence
of both PAD and foot infection conferred a nearly 3-fold higher risk of
leg amputation than either infection or PAD alone.^228^ The treatment of deep
soft-tissue infection typically requires prompt surgical drainage;
vascular imaging and expeditious revascularization generally follow.
Experienced clinical teams have reported very good outcomes when this is
performed in a coordinated and timely fashion.^229,230^ Previous groups have described various
combinations of functions of interdisciplinary care teams (See Online Data Supplement 34a for a complete list of functions). See Section 9.2 for
recommendations related to the role of the interdisciplinary care team
in wound healing therapies for CLI.
**IIa****C-EO****It is reasonable to counsel patients
with PAD without diabetes mellitus about self–foot
examination and healthy foot behaviors.**
N/AAlthough there are limited data to support
patient education about self–foot examination and foot care for
patients with diabetes mellitus, there are no data that have evaluated
this practice in a population of patients with PAD but without diabetes
mellitus. Nonetheless, this is a very low-risk intervention with
potential for benefit. Educational efforts generally include teaching
patients about healthy foot behaviors (eg, daily inspection of feet;
foot care and hygiene, including appropriate toenail cutting strategies;
avoidance of barefoot walking), the selection of appropriately fitting
shoes, and the importance of seeking medical attention for new foot
problems.^223^
**IIa****C-EO****Biannual foot examination by a
clinician is reasonable for patients with PAD and diabetes
mellitus.**
N/AA history of foot ulcers, foot infections,
or amputation identifies patients with a very high
(>10%) yearly incidence of recurrent ulcers.^231^ Examination includes
a visual inspection for foot ulcers (full-thickness epithelial defects)
and structural (bony) deformities, monofilament testing for sensory
neuropathy, and palpation for pedal pulses.


## 8. Revascularization for Claudication

An individualized approach to revascularization for claudication is recommended for each patient to optimize outcome. Revascularization is but one component of care for the patient with claudication, as each patient should have a customized care plan that also includes medical therapy (Section 5), structured exercise therapy (Section 6), and care to minimize tissue loss (Section 7). If a strategy of revascularization for claudication is undertaken, the revascularization strategy should be evidence based and can include endovascular revascularization, surgery, or both.

Because of the variability of ischemic limb symptoms and impact of these symptoms on functional status and QoL, patients should be selected for revascularization on the basis of severity of their symptoms. Factors to consider include a significant disability as assessed by the patient, adequacy of response to medical and structured exercise therapy, status of comorbid conditions, and a favorable risk–benefit ratio. Patient preferences and goals of care are important considerations in the evaluation for revascularization. The revascularization strategy should have a reasonable likelihood of providing durable relief of symptoms. A general recommendation for revascularization as a treatment option for claudication is provided below followed by specific recommendations for endovascular (Section 8.1.1) and surgical (Section 8.1.2) procedures if a revascularization strategy is undertaken.

### 8.1. Revascularization for Claudication: Recommendation


**Recommendation for
Revascularization for Claudication**
**COR****LOE****Recommendation**
**IIa****A****Revascularization is a reasonable
treatment option for the patient with lifestyle-limiting
claudication with an inadequate response to
GDMT.**^12,37,38,232,233^
See Online Data Supplements 35 and 36.A minority of patients with
claudication (estimated at <10% to 15% over
5 years or more) will progress to CLI.^234–237^ Therefore, the role of
revascularization in claudication is improvement in claudication
symptoms and functional status, and consequently in QoL, rather than
limb salvage. Revascularization is reasonable when the patient who
is being treated with GDMT (including structured exercise therapy)
presents with persistent lifestyle-limiting claudication.^12,37,38,232,233^
Lifestyle-limiting claudication is defined by the patient rather
than by any test. It includes impairment of activities of daily
living and/ or vocational and/or recreational activities due to
claudication. There should be clear discussion with the patient
about expected risks and benefits of revascularization, as well as
discussion of the durability of proposed procedures.


#### 8.1.1. Endovascular Revascularization for Claudication: Recommendations

Endovascular techniques to treat claudication include balloon dilation (angioplasty), stents, and atherectomy. These techniques continue to involve and now include covered stents, drug-eluting stents (DES), cutting balloons, and drug-coated balloons. The technique chosen for endovascular treatment is related to lesion characteristics (eg, anatomic location, lesion length, degree of calcification) and operator experience. Assessment of the appropriateness of specific endovascular techniques for specific lesions for the treatment of claudication is beyond the scope of this document.

Revascularization is performed on lesions that are deemed to be hemodynamically significant, and stenoses selected for endovascular treatment should have a reasonable likelihood of limiting perfusion to the distal limb. Stenoses of 50% to 75% diameter by angiography may not be hemodynamically significant, and resting or provoked intravascular pressure measurements may be used to determine whether lesions are significant.^238,239^ Multiple RCTs have compared endovascular procedures to various combinations of medical treatment with or without supervised or unsupervised exercise programs.^12,37,38,217,232,233,240–251^ These trials have used different endpoints and enrolled patients with anatomic disease distribution at different levels.


**Recommendations
for Endovascular Revascularization for
Claudication**
**COR****LOE****Recommendations**
**I****A****Endovascular procedures are
effective as a revascularization option for patients with
lifestyle-limiting claudication and hemodynamically
significant aortoiliac occlusive disease.**^12,37,38,232,240,242,246^
See Online Data Supplements 35 and 36.Two separate systematic analyses
that included RCTs that enrolled patients with aortoiliac
disease reported that endovascular treatment of claudication
improved walking parameters and QoL.^11,12,233^ The CLEVER trial
enrolled only patients with aortoiliac disease and compared
endovascular therapy to supervised exercise therapy and to
medications alone.^37,38^ At 6-month follow-up, both the endovascular
therapy and supervised exercise groups had improved peak walking
time compared with medication alone, with a greater improvement
in the supervised exercise group.^37^ By 18 months, there was
no significant difference between the endovascular therapy and
supervised exercise groups, with a sustained benefit versus
medication alone.^38^ Other RCTs that included patients with
aortoiliac disease have shown QoL, as assessed by questionnaires
and time to onset of claudication, may be superior with
endovascular treatment in combination with a medical and an
exercise treatment plan, compared versus medical treatment
alone.^232,233,246^ The ERASE trial randomized patients with
claudication and aortoiliac (as well as femoropopliteal) disease
to endovascular revascularization plus supervised exercise or
supervised exercise alone. After 1 year, patients in both groups
had significant improvements in walking distances and
health-related QoL, with greater improvements in the
combined-therapy group.^218^ The long-term comparative efficacy of
endovascular revascularization versus supervised exercise
therapy and medical therapy compared to supervised exercise
therapy and medical therapy without revascularization for
aortoiliac disease is unknown.
**IIa****B-R****Endovascular procedures are
reasonable as a revascularization option for patients with
lifestyle-limiting claudication and hemodynamically
significant femoropopliteal disease.**^217,232,243–245,250,251^
See Online Data Supplement 35.Multiple RCTs have demonstrated
short-term efficacy with endovascular treatment of
femoropopliteal disease for claudication versus supervised
exercise training or medical therapy, with benefit that
diminishes by 1 year.^217,232,240–246,250,251^ Two separate systematic reviews that
included RCTs that enrolled patients with femoropopliteal
disease, reported that endovascular treatment of claudication
improved walking parameters and QoL.^11,12,233^ The durability of
endovascular treatment for claudication is directly related to
vessel patency. Long-term patency is greater in the iliac artery
than in the femoropopliteal segment. Furthermore, durability is
diminished with greater lesion length, occlusion rather than
stenosis, the presence of multiple and diffuse lesions,
poor-quality runoff, diabetes mellitus, chronic kidney disease,
renal failure, and smoking.^252–255^ The choice of endovascular therapy as
a revascularization approach for claudication due to
femoropopliteal disease therefore should include a discussion of
outcomes, addressing the risk of restenosis and repeat
intervention, particularly for lesions with poor likelihood of
long-term durability.
**IIb****C-LD****The usefulness of
endovascular procedures as a revascularization option for
patients with claudication due to isolated infrapopliteal
artery disease is unknown**.^256–258^
See Online Data Supplement 35.Isolated infrapopliteal disease is
unlikely to cause claudication. Incidence of in-stent restenosis
is high and long-term benefit lacking with bare-metal stenting
of the infrapopliteal arteries.^256^ Studies that have
enrolled patients with claudication as well as CLI have
demonstrated a benefit of DES versus bare-metal stents or versus
drug-coated balloons for revascularization of infrapopliteal
lesions.^257,258^ However, these differences were mainly for
patency and restenosis endpoints, and neither of these studies
included patient-oriented outcomes, such as walking function or
QoL parameters. Additional efficacy data on the use of
infrapopliteal drug-coated balloon or DES for the treatment of
claudication are likely to be published in the near future.
**III: Harm****B-NR****Endovascular procedures
should not be performed in patients with PAD solely to
prevent progression to CLI.**^234–237,259–261^
See Online Data Supplements 36 and 38.There are no data to support a
practice paradigm of performing endovascular procedures on
patients with PAD for the purpose of preventing progression of
claudication symptoms to CLI. Reported rates of amputation or
progression to CLI from prospective cohort studies of patients
with claudication are <10% to 15% over 5
years or more, and increased mortality rate associated with
claudication is usually the result of cardiovascular events
rather than limb-related events.^234–237,262^ Similarly, there are no
data to support revascularization in patients with asymptomatic
PAD. Procedural risks include bleeding, renal failure from
contrast-induced nephropathy, and the possibility of adverse
limb outcomes.^259–261^ Therefore, the known risks of
endovascular procedures outweigh any hypothetical benefit of
preventing progression from asymptomatic PAD or claudication to
CLI.


#### 8.1.2. Surgical Revascularization for Claudication: Recommendations


**Recommendations
for Surgical Revascularization for Claudication**
**COR****LOE****Recommendations**
**I****A****When surgical
revascularization is performed, bypass to the popliteal
artery with autogenous vein is recommended in preference to
prosthetic graft material.**^263–271^
See Online Data Supplements 37 and 38.The superficial femoral and
proximal popliteal arteries are the most common anatomic sites
of stenosis or occlusion among individuals with claudication.
Femoral-popliteal bypass is therefore one of the most common
surgical procedures for claudication and may be performed under
general or regional anesthesia. The type of conduit and site of
popliteal artery anastomosis (above versus below knee) are major
determinants of outcomes associated with femoral-popliteal
bypass. Systematic reviews and meta-analyses have identified a
clear and consistent primary patency benefit for autogenous vein
versus to prosthetic grafts for popliteal artery
bypass.^270,271^ Prosthetic grafts to the popliteal artery
above the knee have reduced patency rates and increased rates of
repeat intervention.^263,266,269,272^ Sparse evidence suggests a long-term
patency advantage for Dacron over polytetrafluoroethylene (known
as PTFE) graft for above-knee bypass,^270^ although this finding
has not been consistently demonstrated in all RCTs.^266,273,274^
**IIa****B-NR****Surgical procedures are
reasonable as a revascularization option for patients with
lifestyle-limiting claudication with inadequate response to
GDMT, acceptable perioperative risk, and technical factors
suggesting advantages over endovascular
procedures.**^232,265,275–277^
See Online Data Supplements 37 and 38.Systematic reviews have concluded
that surgical procedures are an effective treatment for
claudication and have a positive impact on QoL and walking
parameters but have identified sparse evidence supporting the
effectiveness of surgery compared with other
treatments.^11,233,278,279^ Although symptom and patency outcomes for
surgical interventions may be superior versus less invasive
endovascular treatments for specific patients, surgical
interventions are also associated with greater risk of adverse
perioperative events.^280–286^ Treatment selection should therefore
be individualized on the basis of the patient's goals,
perioperative risk, and anticipated benefit. Surgical procedures
for claudication are usually reserved for individuals who a) do
not derive adequate benefit from nonsurgical therapy, b) have
arterial anatomy favorable to obtaining a durable result with
surgery, and c) have acceptable risk of perioperative adverse
events. Acceptable risk is defined by the individual patient and
provider on the basis of symptom severity, comorbid conditions,
and appropriate GDMT risk evaluation. Guidelines for the
evaluation and management of patients undergoing noncardiac
surgery, including vascular surgical procedures, have been
previously published.^21^
**III: Harm****B-R****Femoral-tibial artery
bypasses with prosthetic graft material should not be used
for the treatment of claudication.**^287–289^
See Online Data Supplement 37.Bypasses to the tibial arteries
with prosthetic material for treatment of claudication should be
avoided because of very high rates of graft failure and
amputation.^287–289^
**III: Harm****B-NR****Surgical procedures should
not be performed in patients with PAD solely to prevent
progression to CLI.**^234–237,262^
See Online Data Supplements 37 and 38.Claudication does not commonly
progress to CLI. Reported rates of amputation or progression to
CLI from prospective cohort studies of patients with
claudication are <10% to 15% for 5 years
or more, and increased mortality rate associated with
claudication is usually the result of cardiovascular events
rather than limb-related events.^234–237,262^ Surgical intervention
should not be performed primarily to prevent disease
progression, given the risk of adverse perioperative events
without potential for significant benefit. Similarly, there are
no data to support surgical revascularization in patients with
asymptomatic PAD to prevent progression to CLI.


## 9. Management of CLI

Patients with CLI are at increased risk of amputation and major cardiovascular ischemic events. Care of the patient with CLI includes evaluation for revascularization and wound healing therapies, with the objective to minimize tissue loss, completely heal wounds, and preserve a functional foot. Medical therapy to prevent cardiovascular ischemic events is also an important component of care for the patient with CLI (Section 5).

### 9.1. Revascularization for CLI: Recommendations


**Recommendation for
Revascularization for CLI**
**COR****LOE****Recommendation**
**I****B-NR****In patients with CLI,
revascularization should be performed when possible to minimize
tissue loss.**^290^
See Online Data Supplement 39.Patients with CLI are at high risk of
major cardiovascular ischemic events, as well as nonhealing wounds
and major amputation. In a systematic review of 13 studies of
patients with CLI who did not receive revascularization, which
included patients enrolled in medical and angiogenic therapy trials,
there was a 22% all-cause mortality rate and a 22%
rate of major amputation at a median follow-up of 12
months.^290^ The goal of surgical or endovascular
revascularization is to provide in-line blood flow to the foot
through at least 1 patent artery, which will help decrease ischemic
pain and allow healing of any wounds, while preserving a functional
limb. Multiple RCTs comparing contemporary surgical and endovascular
treatment for patients with CLI are ongoing.^15–17^ Revascularization is not
warranted in the setting of a nonviable limb.
**I****C-EO****An evaluation for
revascularization options should be performed by an
interdisciplinary care team (**Table 9**) before amputation in the
patient with CLI.**
N/APatients with CLI should be evaluated
by an interdisciplinary care team. Before amputation, evaluation
generally includes imaging for assessment of revascularization
options (eg, duplex ultrasound, CTA, MRA, or catheter-based
angiogram). The objective of this strategy is to minimize tissue
loss and preserve a functional limb with revascularization.


#### 9.1.1. Endovascular Revascularization for CLI: Recommendations


**Recommendations
for Endovascular Revascularization for CLI**
**COR****LOE****Recommendations**
**I****B-R****Endovascular procedures are
recommended to establish in-line blood flow to the foot in
patients with nonhealing wounds or
gangrene.**^292,293^
See Online Data Supplement 39.The technique chosen for
endovascular treatment of CLI is related to anatomic location of
lesions, lesion characteristics, and operator experience.
Revascularization is performed on hemodynamically significant
stenoses that are likely to be limiting blood flow to the limb.
For stenoses of 50% to 75%, where the
hemodynamic significance is unclear, intravascular pressure
measurements may be used to determine hemodynamic
significance.^294^ The BASIL (Bypass versus Angioplasty
in Severe Ischemia of the Leg) RCT demonstrated that
endovascular revascularization is an effective option for
patients with CLI as compared with open surgery.^292,293^ The primary endpoint of
amputation-free survival was the same in the endovascular and
surgical arms. Of note, the endovascular arm used only
PTA.^292,293^ Multiple RCTs comparing contemporary
surgical and endovascular treatment for patients with CLI are
ongoing.^15–17^
Table 10 addresses
factors that may prompt an endovascular versus surgical approach
to the patient with CLI.
**IIa****C-LD****A staged approach to
endovascular procedures is reasonable in patients with
ischemic rest pain.**^295,296^
N/AFor patients with multilevel
disease who suffer from ischemic rest pain, in-flow lesions are
generally addressed first.^295,296^ Depending on procedural characteristics,
including contrast volume used, radiation exposure, and
procedure time, out-flow lesions can be addressed in the same
setting or at a later time if symptoms persist. This strategy
for ischemic rest pain is distinct from the strategy recommended
for CLI in the patient with a nonhealing wound or gangrene. In
that scenario, restoration of direct in-line flow to the foot is
essential for wound healing.
**IIa****B-R****Evaluation of lesion
characteristics can be useful in selecting the endovascular
approach for CLI.**^297,298^
See Online Data Supplement 39.The lesion characteristics to
consider include length, anatomic location, and extent of
occlusive disease. For example, if an adequate angioplasty
result can be achieved with PTA alone for short (<10 cm)
stenoses in the femoropopliteal segment, then stent placement is
not necessary.^297,298^ Presence of thrombosis or calcification at
the lesion site will also affect the endovascular approach. In
general, the advantages of DES and drug-coated balloons over PTA
alone or bare-metal stents are more consistent in the
femoropopliteal segment than for infrapopliteal
interventions.^257,258,299–309^ However, these differences are mainly
for patency, restenosis, and repeat-revascularization endpoints.
Most studies were underpowered or did not examine other
patient-oriented outcomes, such as amputation or wound healing
in CLI. Endovascular techniques continue to evolve rapidly, and
there has been limited literature comparing techniques with
regard to clinically significant outcomes, such as amputation or
wound healing.
**IIb****B-NR****Use of angiosome-directed
endovascular therapy may be reasonable for patients with CLI
and nonhealing wounds or gangrene.**^310–319^
See Online Data Supplements 39 and 40.During the past decade, the goal of
care with regard to endovascular therapy for the treatment of
nonhealing wounds due to CLI has been establishment of direct
in-line blood flow to the affected limb. The angiosome concept
has also been described in the literature in relation to the
treatment of nonhealing wounds. Angiosome-directed treatment
entails establishing direct blood flow to the infrapopliteal
artery directly responsible for perfusing the region of the leg
or foot with the nonhealing wound. Multiple retrospective
studies and 1 small nonrandomized prospective study assessing
the efficacy of this concept have been published.^119,310–321^ Meta-analyses of these
studies found improved wound healing and limb salvage with
angiosome-guided therapy but cautioned that the quality of the
evidence was low.^322,323^ Although the angiosome concept is
theoretically satisfying, randomized data comparing the
establishment of in-line flow versus angiosome-guided therapy
have yet to be published. Furthermore, there is no evidence yet
to demonstrate the potential benefit of treating additional
infrapopliteal arteries once in-line flow has been established
in one artery, regardless of angiosome. Important considerations
with regard to angiosome-guided therapy include the potential
for longer procedural times, more contrast exposure, and more
technically complex procedures. The impact of all these factors
needs to be weighed against the likelihood of a technically
successful procedure providing hypothetical added benefit over
the establishment of in-line blood flow.


#### 9.1.2. Surgical Revascularization for CLI: Recommendations


**Recommendations
for Surgical Revascularization for CLI**
**COR****LOE****Recommendations**
**I****A****When surgery is performed for
CLI, bypass to the popliteal or infrapopliteal arteries (ie,
tibial, pedal) should be constructed with suitable
autogenous vein.**^263,266,269,272^
See Online Data Supplement 37.Many large RCTs have demonstrated
that bypasses above the knee should be autogenous vein either
reversed or in situ vein.^263,266,269,272^ There are large single-center trials
showing the efficacy of autogenous vein to distal tibial
vessels.^324,325^ In addition, composite sequential
femoropopliteal-tibial bypass and bypass to an isolated
popliteal arterial segment that has collateral out flow to the
foot are both acceptable methods of revascularization and should
be considered when no other form of bypass with adequate
autogenous conduit is possible.^326,327^
**I****C-LD****Surgical procedures are
recommended to establish in-line blood flow to the foot in
patients with nonhealing wounds or
gangrene.**^328–330^
See Online Data Supplement 42.In patients presenting with
nonhealing ulcers or gangrene, surgical procedures should be
performed to establish in-line blood flow to the foot.^328–330^
Table 10 addresses
factors that may prompt a surgical approach to the patient with
CLI.
**IIa****B-NR****In patients with CLI for whom
endovascular revascularization has failed and a suitable
autogenous vein is not available, prosthetic material can be
effective for bypass to the below-knee popliteal and tibial
arteries.**^331–333^
See Online Data Supplement 42.There are studies demonstrating
that patients for whom endovascular treatment for CLI has failed
can be treated successfully with autogenous vein bypass
graft^332,333^ or prosthetic material.^331^ Although
autogenous vein is the preferred conduit for surgical
revascularization, prosthetic conduit is a secondary option for
patients with CLI without suitable saphenous vein who require
surgical revascularization.
**IIa****C-LD****A staged approach to surgical
procedures is reasonable in patients with ischemic rest
pain.**^334–336^
N/AIt is reasonable to perform a
staged approach to revascularization in patients with ischemic
rest pain with multilevel disease. For example, aortoiliac
(inflow) disease may be treated first with endovascular
treatment or by surgical reconstruction, depending on lesion
characteristics, patient comorbidities, and patient
preference.^337,338^ Combined percutaneous and surgical
revascularization may require separate interventions, typically
with the most proximal procedure performed first.


### 9.2. Wound Healing Therapies for CLI: Recommendations


**Recommendations for
Wound Healing Therapies for CLI**
**COR****LOE****Recommendations**
**I****B-NR****An interdisciplinary care team
should evaluate and provide comprehensive care for patients with
CLI and tissue loss to achieve complete wound healing and a
functional foot.**^229,339–341^
See Online Data Supplement 44.The management of patients with CLI and
nonhealing wounds should include coordinated efforts for both
revascularization and wound healing, because the risk of
limb-threatening infections remains until complete wound healing is
achieved. The structure and activities of interdisciplinary care
teams for CLI may vary according to several factors, including the
local availability of resources. Previous groups have described
various combinations of activities of this team, which are in
addition to revascularization and include functions such as wound
care, infection management, orthotics, and prosthetics (see Online Data Supplement 34a for a complete list of functions).
Coordination of these activities and some degree of organized team
structure are recommended, as opposed to ad hoc or unstructured
referrals among various specialty clinicians not involved in
interdisciplinary care.
Ambulatory patients with PAD and
nonhealing foot ulcers should be considered for efforts to prevent
amputation. The components of this effort may include
revascularization, offloading, treatment of infection, and wound
care. The long-term outcome of the limb is excellent when complete
wound healing can be achieved.^339^Revascularization should be coordinated
with the efforts of clinicians who manage foot infections, provide
offloading, and achieve complete wound healing, either through
medical therapy, surgical options, or a combination thereof.
Coordinated and timely interdisciplinary care can achieve excellent
limb outcomes for patients with PAD and nonhealing foot
wounds.^229,339–341^
**I****C-LD****In patients with CLI, wound care
after revascularization should be performed with the goal of
complete wound healing.**^339^
See Online Data Supplement 44.A comprehensive plan for treatment of
CLI must include a plan for achieving an intact skin surface on a
functional foot. One study demonstrated a limb salvage rate of
100% at 3 years in a cohort of patients with CLI who
achieved complete wound healing with endovascular revascularization
and dedicated wound care.^339^ Before revascularization, the
interdisciplinary care team should devise a plan to achieve the goal
of complete wound healing. After successful revascularization, most
patients with gangrene of the foot are evaluated for minor
amputation with staged/delayed primary closure or surgical
reconstruction when feasible.^342–344^ Negative-pressure wound therapy dressings
are helpful to achieve wound healing after revascularization and
minor (ie, digit or partial foot) amputation when primary or delayed
secondary closure is not feasible.^345,346^ Spontaneous amputation, or autoamputation,
of gangrenous digits should be reserved for palliation in patients
without options for revascularization.^345,347,348^
Other evidence-based guidelines
relevant to those with nonhealing foot wounds following
revascularization cover the full spectrum of diabetic foot
problems^349^ or separately consider the management of
infection,^225,350^ offloading,^351^ and wound care.^352^ To date, there
are no RCTs or high-quality studies that have focused on wound
healing adjuncts in limbs with severe PAD (eg, topical cytokine
ointments, skin substitutes, cell-based therapies intended to
optimize wound healing).
**IIb****B-NR****In patients with CLI,
intermittent pneumatic compression (arterial pump) devices may
be considered to augment wound healing and/or ameliorate severe
ischemic rest pain.**^353^
See Online Data Supplement 44.A systematic review of studies that
used intermittent pneumatic compression devices specifically
designed to augment arterial perfusion of the lower extremities
suggests that these may provide modest clinical benefit
(specifically, decreased amputation rates and improved QoL) in
patients with CLI who were ineligible for
revascularization.^353^ The potential benefit appears to outweigh
the low risk associated with the use of these devices.
**IIb****C-LD****In patients with CLI, the
effectiveness of hyperbaric oxygen therapy for wound healing is
unknown.**^354^
See Online Data Supplement 44.The literature evaluating the utility
of hyperbaric oxygen therapy has focused on patients without severe
PAD and has not demonstrated a long-term benefit on wound healing or
improving amputation-free survival when compared with sham
treatment.^355^ There are no published studies evaluating the
role of hyperbaric oxygen therapy for patients with
nonreconstructible PAD. One small RCT that focused on patients with
foot ulcers and PAD (ABI <0.80 or TBI <0.70) for
whom no revascularization was planned demonstrated a significant
decrease in ulcer area at 6 weeks, but no significant differences in
ulcer size at 6 months, complete ulcer healing at 6 weeks or 6
months, and major or minor amputations.^354^ Further research on the
utility of hyperbaric oxygen therapy in this context is needed.
**III: No Benefit****B-R****Prostanoids are not indicated in
patients with CLI.**^356^
See Online Data Supplement 43.A systematic review and meta-analysis
concluded that RCTs have not demonstrated meaningful long-term
clinical benefit from the administration of prostanoids to patients
with CLI attributable to nonreconstructible PAD.^356^


## 10. Management of ALI

ALI is one of the most treatable and potentially devastating presentations of PAD. Timely recognition of arterial occlusion as the cause of an ischemic, cold, painful leg is crucial to successful treatment. The writing committee has used a standard definition of ALI in which symptom duration is <2 weeks (Table 3).^33,34^ Category I refers to viable limbs that are not immediately threatened. Category II refers to threatened limbs. Category IIa limbs are marginally threatened and salvageable, if promptly treated. Category IIb are immediately threatened limbs that require immediate revascularization if salvage is to be accomplished. Category III are irreversibly damaged limbs, in which case resultant major tissue loss or permanent nerve damage is inevitable.^34^

### 10.1. Clinical Presentation of ALI: Recommendations


**Figure 3:**
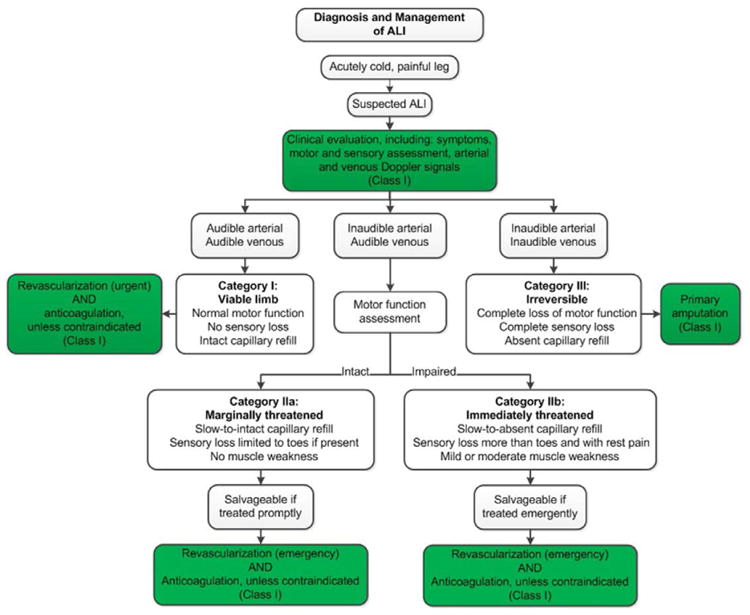
Diagnosis and Management of ALI ^33,34^, Colors correspond to Class of Recommendation in Table 1. ALI indicates acute limb ischemia.

**Recommendations for
Clinical Presentation of ALI**
**COR****LOE****Recommendations**
**I****C-EO****Patients with ALI should be
emergently evaluated by a clinician with sufficient experience
to assess limb viability and implement appropriate
therapy.**
N/APatients with ALI should be rapidly
evaluated by a vascular specialist if one is available. Depending on
local clinical expertise, the vascular specialist may be a vascular
surgeon, interventional radiologist, cardiologist, or a general
surgeon with specialized training and experience in treating PAD. If
such expertise is not locally or rapidly available, there should be
strong consideration of transfer of the patient to a facility with
such resources. The more advanced the degree of ischemia, the more
rapidly the communication (including communication about potential
patient transfer) needs to occur.
**I****C-LD****In patients with suspected ALI,
initial clinical evaluation should rapidly assess limb viability
and potential for salvage and does not require
imaging.**^357–361^
See Online Data Supplements 45 and 46.ALI is a medical emergency and must be
recognized rapidly. The time constraint is due to the period that
skeletal muscle will tolerate ischemia—roughly 4 to 6
hours.^362^
A rapid assessment of limb viability and ability to restore arterial
blood flow should be performed by a clinician able to either
complete the revascularization or triage the patient.^358^ Lower extremity
symptoms in ALI can include both pain and loss of function. The
longer these symptoms are present, the less likely the possibility
of limb salvage.^360,361^ Clinical assessment must include symptom
duration, pain intensity, and motor and sensory deficit severity to
distinguish a threatened from a nonviable extremity (Figure 3). The bedside assessment should
include arterial and venous examination with a handheld
continuous-wave Doppler because of the inaccuracy of pulse
palpation.^34^ The loss of dopplerable arterial signal
indicates that the limb is threatened. The absence of both arterial
and venous Doppler signal indicates that the limb may be
irreversibly damaged (nonsalvageable). Comorbidities should be
investigated and managed aggressively, but this must not delay
therapy. Even in the setting of rapid and effective
revascularization, the 1-year morbidity and mortality rates
associated with ALI are high.^360,363^


### 10.2. Medical Therapy for ALI: Recommendations


**Recommendation for
ALI Medical Therapy**
**COR****LOE****Recommendation**
**I****C-EO****In patients with ALI, systemic
anticoagulation with heparin should be administered unless
contraindicated.**
N/AHeparin (generally intravenous
unfractionated heparin) is given to all patients acutely.^35,364^ This can stop thrombus
propagation and may provide an anti-inflammatory effect that lessens
the ischemia. Patients who have received heparin before the onset of
ALI and have a decrease in platelet count may have heparin-induced
thrombocytopenia.^365,366^ In this situation, a direct thrombin inhibitor
is given, rather than heparin, if heparin-induced thrombocytopenia
with thrombosis is suspected.


### 10.3. Revascularization for ALI: Recommendations


**Recommendations for
Revascularization for ALI**
**COR****LOE****Recommendations**
**I****C-LD****In patients with ALI, the
revascularization strategy should be determined by local
resources and patient factors (eg, etiology and degree of
ischemia).**^367–369^
See Online Data Supplement 47.For marginally or immediately
threatened limbs (Category IIa and IIb ALI [Figure 3]), revascularization
should be performed emergently (within 6 hours). For viable limbs
(Category I ALI [Figure 3]), revascularization should be performed an on
urgent basis (within 6–24 hours). The revascularization
strategy can range from catheter-directed thrombolysis to surgical
thromboembolectomy. Available facilities and clinical expertise are
factors that should be considered when determining the
revascularization strategy. The technique that will provide the most
rapid restoration of arterial flow with the least risk to the
patient should be selected. For example, catheter-directed
thrombolysis can provide rapid restoration of arterial flow to a
viable or marginally threatened limb, particularly in the setting of
recent occlusion, thrombosis of synthetic grafts, and stent
thrombosis.^367^ If this is not available locally, surgical
options for timely revascularization should be considered, along
with the feasibility of timely transfer to a facility with the
necessary expertise.
**I****A****Catheter-based thrombolysis is
effective for patients with ALI and a salvageable
limb.**^367–371^
See Online Data Supplement 47.Assessment of the comparative
effectiveness of catheter-based thrombolysis versus open surgery is
complicated by variable definitions of ALI in this literature. Four
RCTs comparing catheter-based thrombolysis to surgery,^367,369–371^ as well as a
meta-analysis,^368^ have demonstrated similar limb salvage
rates between the 2 approaches but better survival with
catheter-based therapy. The survival advantage of catheter-based
therapy may be at least in part attributable to multiple
comorbidities found among the population of patients who present
with ALI. Increased comorbidities are likely to contribute to
increased perioperative risk. Several of the RCTs included patients
with relatively chronic ischemia. Acuity and severity are both
factors in the decision to consider thrombolysis.^367,369–371^
**I****C-LD****Amputation should be performed as
the first procedure in patients with a nonsalvageable
limb.**^372,373^
See Online Data Supplement 48.For patients with Category III ALI
(Figure 3), amputation
should be performed as the index procedure. Prolonged duration of
ischemia is the most common factor in patients requiring amputation
for treatment of ALI. The risks associated with reconstruction
outweigh the potential benefit in a limb that is already insensate
or immobile because of prolonged ischemia. Patients who have an
insensate and immobile limb in the setting of prolonged ischemia
(>6 to 8 hours) are unlikely to have potential for limb
salvage.^34,362^ In addition, in
this setting the reperfusion and circulation of ischemic metabolites
can result in multiorgan failure and cardiovascular collapse.
However, if pain can be controlled and there is no evidence of
infection, amputation may be deferred if this meets with the
patient's goals.
**I****C-LD****Patients with ALI should be
monitored and treated (eg, fasciotomy) for compartment syndrome
after revascularization.**^372,373^
See Online Data Supplement 48.The lower extremity muscles reside in
compartments, surrounded by fascia and bones. Reperfusion to
ischemic muscles can cause cellular edema, resulting in increased
compartment pressure. When compartment pressure is >30 mm
Hg, there is capillary and venule compression that leads to
malperfusion of the muscle; this is compartment syndrome. Fasciotomy
is indicated when the compartment pressure increases. Measurement of
intracompartment pressure is not always easily accessible. In such
cases, evaluation for fasciotomy is prompted by development of
increased pain, tense muscle, or nerve injury. Fasciotomy should be
considered for patients with Category IIb ischemia for whom the time
to revascularization is >4 hours.
**IIa****B-NR****In patients with ALI with a
salvageable limb, percutaneous mechanical thrombectomy can be
useful as adjunctive therapy to thrombolysis.**^374–378^
See Online Data Supplements 49 and 50.Multiple nonrandomized studies have
suggested that percutaneous mechanical thrombectomy in combination
with pharmacological therapy can be beneficial in the treatment of
threatened limbs.^374–378^
**IIa****C-LD****In patients with ALI due to
embolism and with a salvageable limb, surgical
thromboembolectomy can be effective.**^379–381^
See Online Data Supplements 49 and 50.Patients with arterial embolism and an
absent pulse ipsilateral to the ischemic limb can be treated by
exposure of an artery in the affected limb and balloon-catheter
thromboembolectomy. These patients may benefit from adjunctive
intraoperative fibrinolytics. In the event that thromboembolectomy
does not restore arterial flow, bypass can be performed.^381–383^
**IIb****C-LD****The usefulness of
ultrasound-accelerated catheter-based thrombolysis for patients
with ALI with a salvageable limb is unknown.**^384–386^
See Online Data Supplements 47 and 50.The use of ultrasound-accelerated
catheter delivery of thrombolytic agents has been published in case
series^384^
and retrospective analyses.^385^ However, the single RCT comparing this
technique to standard catheter-based thrombolytic therapy failed to
demonstrate a difference in outcomes, including bleeding, despite a
lower total amount of lytic delivered.^386^


### 10.4. Diagnostic Evaluation of the Cause of ALI: Recommendations


**Recommendations for
Diagnostic Evaluation of the Cause of ALI**
**COR****LOE****Recommendations**
**I****C-EO****In the patient with ALI, a
comprehensive history should be obtained to determine the cause
of thrombosis and/or embolization.**
N/AIn addition to identifying a known
history of PAD, the history should focus on uncovering clinical
evidence of other conditions that can result in ALI through either
embolic or thrombotic mechanisms. These conditions include atrial
fibrillation, left ventricular thrombus, aortic dissection, trauma,
hypercoagulable state, and presence of a limb artery bypass graft.
The clinical history should identify the presence or absence of a
history of MI, symptoms and signs of left ventricular dysfunction
resulting in congestive heart failure, or possible endocarditis. The
history should evaluate for possibility of deep vein thrombosis with
intracardiac shunt (eg, patent foramen ovale or other that may
result in paradoxical arterial embolism), hypercoagulable state, and
family history of thrombosis.
**IIa****C-EO****In the patient with a history of
ALI, testing for a cardiovascular cause of thromboembolism can
be useful.**
N/ATreatment of ALI should not be delayed
for testing for the underlying cause of the limb ischemia. Delay
from symptom onset to revascularization is a major determinant of
outcome.^360,361^ The evaluation of a cardiovascular cause of
ALI is most useful in the patient without underlying PAD. Evaluation
for cardiovascular cause includes electrocardiogram or additional
heart rhythm monitoring to detect atrial fibrillation,
electrocardiogram to detect evidence of MI, and echocardiography to
further determine whether there is a cardiac etiology for
thromboembolism, such as valvular vegetation, left atrial or left
ventricular thrombus, or intracardiac shunt.^387^


## 11. Longitudinal Follow-Up: Recommendations

PAD is a lifelong chronic medical condition. Ongoing care focuses on cardiovascular risk reduction with medical therapy, optimizing functional status with structured exercise and, when indicated, revascularization.


**Recommendations for
Longitudinal Follow-Up**
**COR****LOE****Recommendations**
**I****C-EO****Patients with PAD should be followed
up with periodic clinical evaluation, including assessment of
cardiovascular risk factors, limb symptoms, and functional
status.**
N/AA comprehensive care plan for patients with
PAD includes periodic clinical evaluation by a healthcare provider with
experience in the care of vascular patients. Clinical evaluation should
include assessment of cardiovascular risk factors, assessment of
adherence to medical therapy, and re-evaluation of smoking cessation
efforts. Comprehensive lifestyle modification, including heart-healthy
nutrition, is encouraged.^22^ Patients with PAD should also undergo periodic
assessment of limb symptoms, functional status, and their ability to
participate in vocational and recreational activities. Ongoing
participation in a structured exercise program should be facilitated.
Foot examination and patient counseling about healthy foot behaviors in
PAD are addressed in Section 7.
**I****C-EO****Patients with PAD who have undergone
lower extremity revascularization (surgical and/or endovascular)
should be followed up with periodic clinical evaluation and ABI
measurement.**
N/AIn addition to the clinical evaluation of
cardiovascular risk factors, functional status, and adherence to medical
therapy and smoking cessation, patients with PAD who have previously
undergone lower extremity revascularization (surgical and/or
endovascular) require additional ongoing assessment and care. Follow-up
visits after revascularization should include reassessment of the
patient's limb symptoms and interval change in functional
status, as well as participation in a structured exercise program. Pulse
examination and ABI are included in the assessment. A change in ABI of
0.15 is considered clinically significant.^388^
**IIa****B-R****Duplex ultrasound can be beneficial
for routine surveillance of infrainguinal, autogenous vein bypass
grafts in patients with PAD.**^389–395^
See Online Data Supplements 51 and 52.A general surveillance schedule may be at 4
to 6 weeks, 6 months, and 12 months in the first year and yearly
thereafter. It is important that testing frequency is individualized to
the patient, type of arterial bypass, and any prior duplex scan
findings. Duplex graft surveillance focuses on the identification of
high-grade stenosis (eg, peak systolic velocity >300 cm/s and
peak systolic velocity ratio across the stenosis >3.5) or
impending graft failure (eg, PSV <40 cm/s).^392,395^ Detection of a graft stenosis prompts the
consideration of further revascularization to treat the stenosis and
maintain graft patency. Duplex may detect significant stenoses that may
not be detected by a decline in ABI.^394^ Although case series have
demonstrated high rates of primary assisted patency with a duplex
ultrasound-surveillance strategy, RCTs of duplex surveillance versus
clinical surveillance with the ABI have demonstrated mixed results in
terms of a benefit on patency and limb outcomes.^391,393,396^
**IIa****C-LD****Duplex ultrasound is reasonable for
routine surveillance after endovascular procedures in patients with
PAD.**^397–399^
See Online Data Supplement 52.Studies have developed duplex ultrasound
diagnostic criteria for diagnosing restenosis at the site of
endovascular revascularization. Diagnostic criteria need to be
customized to the location (eg, iliac or superficial femoral artery) and
type of intervention (eg, angioplasty, uncovered stent, or covered
stent). The optimal timing for surveillance after endovascular
procedures is unclear.^397–399^ There are limited outcome data on routine duplex
surveillance versus clinical surveillance plus the ABI after
endovascular revascularization.^397–399^ The value of duplex ultrasound may be greater in
cases with higher rates of restenosis, such as after interventions to
treat very long lesions or occlusions.^400^
**IIb****B-R****The effectiveness of duplex
ultrasound for routine surveillance of infrainguinal prosthetic
bypass grafts in patients with PAD is uncertain.**^393,401–403^
See Online Data Supplements 51 and 52.Duplex ultrasound of prosthetic bypass
grafts may be used to characterize mid-graft velocity, because low
velocities can predict impending graft failure.^401–403^ Outcome studies of duplex
surveillance of prosthetic grafts have not shown consistent
benefit.^393,401–403^ One RCT of duplex versus
clinical surveillance with the ABI for femoropopliteal grafts did not
show a benefit of duplex on outcome in the subset of patients with
prosthetic grafts, though there was a benefit of duplex surveillance for
vein bypass grafts.^393^


## 12. Evidence Gaps and Future Research Directions

In performing the evidence review and in developing the present guidelines, the writing committee identified the following critical evidence gaps and future directions for PAD-related research:

- Basic science and translational studies to better understand the vascular biology of endovascular therapies and bypass grafting and to develop new methods for preventing restenosis after revascularization.
- Determination of risk factors for progression from asymptomatic PAD to symptomatic disease, including CLI.
- RCTs needed to determine the value of using the ABI to identify asymptomatic patients with PAD for therapies to reduce cardiovascular risk (eg, antiplatelet agents, statins, and other therapies).
- Advancement in PAD diagnostics, such as technologies for simplified yet highly accurate measurement of the ABI and tools for more reliable noninvasive perfusion assessment in CLI.
- Comparative-effectiveness studies to determine the optimal antiplatelet therapy (drug or drugs and dosage) for prevention of cardiovascular and limb-related events in patients with PAD.
- Development of additional medical therapies for claudication–an area of unmet medical need with a currently limited research pipeline.^404^
- Studies to investigate the role of dietary intervention, in addition to statin therapy, to improve outcome and modify the natural history of PAD.
- Additional research to identify the best community-or home-based exercise programs for patients with PAD to maximize functional status and improve QoL, as well as the role of such exercise programs before or in addition to revascularization.
- Development and validation of improved clinical classification systems for PAD that incorporate symptoms, anatomic factors, and patient-specific risk factors and can be used to predict clinical outcome and optimize treatment approach. An example of a recently developed classification system is the Society for Vascular Surgery limb classification system, based on wound, ischemia, and foot infection (WIfI), which has been validated in different populations and may permit more meaningful prognosis in patients with CLI.^405–409^
- Comparative- and cost-effectiveness studies of the different endovascular technologies for treatment of claudication and CLI, including drug-coated balloons and DES. Studies should include patient-centered end-points, such as functional parameters, time to wound healing, and QoL, in addition to standard patency-focused outcomes. These studies could then be incorporated into value-based clinical algorithms for approach to revascularization for claudication and CLI.
- Additional studies to demonstrate the impact of multisocietal registries on clinical outcomes and appropriate use. At present, these include the Vascular Quality Initiative (VQI), the National Cardiovascular Data Registry Peripheral Vascular Intervention Registry™ (PVI Registry™), and the National Radiology Data Registry for Interventional Radiology (NRDR). These registries provide an opportunity to obtain “real-world” data on surgical and endovascular procedures for PAD and to improve quality by providing feedback to participating centers. Future efforts should incorporate these registries into interventional RCTs and postmarketing studies of PAD-related devices.

## 13. Advocacy Priorities

The writing committee identified 3 priorities for multi-societal advocacy initiatives to improve health care for patients with PAD. First, the writing committee supports the availability of the ABI as the initial diagnostic test to establish the diagnosis of PAD in patients with history or physical examination findings suggestive of PAD (Table 5). Although the ABI test is generally reimbursed by third-party payers for patients with classic claudication or lower extremity wounds, payers may not provide reimbursement for the ABI with other findings suggestive of PAD, such as lower extremity pulse abnormalities or femoral bruits. The writing committee affirms the importance of confirming the diagnosis of PAD in such patients to allow for GDMT as delineated in this document. Second, the writing committee supports the vital importance of insuring access to supervised exercise programs for patients with PAD. Although extensive high-quality evidence supports supervised exercise programs to improve functional status and QoL, only a minority of patients with PAD participate in such programs because of lack of reimbursement by third-party payers. Third, the writing committee recognizes the need for incorporation of patient-centered outcomes into the process of regulatory approval of new medical therapies and revascularization technologies. For revascularization technologies, regulatory approval is driven primarily by data on angiographic efficacy (ie, target lesion patency) and safety endpoints. The nature of the functional limitation associated with PAD warrants the incorporation of patient-centered outcomes, such as functional parameters and QoL, into the efficacy outcomes for the approval process.

## Supplementary Material

Evidence Table 1. Nonrandomized Trials, Observational Studies, and/or Registries of History for Clinical Assessment for PAD–Section 2.1.
Evidence Table 2. Nonrandomized Trials, Observational Studies, and/or Registries of Physical Examination for Clinical Assessment for PAD–Section 2.1.
Evidence Table 3. RCTs of Resting ABI for Diagnosing PAD–Section 3.1.
Evidence Table 4. Nonrandomized Trials, Observational Studies, and/or Registries of Resting ABI for Diagnosing PAD–Section 3.1.
Evidence Table 5. Nonrandomized Trials, Observational Studies, and/or Registries of Physiological Testing–Section 3.2.
Evidence Table 6. Nonrandomized Trials, Observational Studies, and/or Registries of Imaging for Anatomic Assessment (Ultrasound, CTA, MRA, Angiography)–Section 3.3.
Evidence Table 7. RCTs of Imaging for Anatomic Assessment (Ultrasound, CTA, MRA, Angiography)–Section 3.3.
Evidence Table 8. Nonrandomized Trials, Observational Studies, and/or Registries for Abdominal Aortic Aneurysm–Section 4.1.
Evidence Table 9. Nonrandomized Trials, Observational Studies, and/or Registries of Coronary Artery Disease Screening in PAD–Section 4.2.
Evidence Table 10. RCTs for CAD Screening in PAD–Section 4.2.
Evidence Table 11. Nonrandomized Trials, Observational Studies, and/or Registries of Screening in Carotid Artery Disease–Section 4.3.
Evidence Table 12. Nonrandomized Trials, Observational Studies, and/or Registries for Renal Artery Disease–Section 4.4.
Evidence Table 13. RCTs Evaluating Antiplatelet Agents– Section 5.1.
Evidence Table 14. Nonrandomized Trials, Observational Studies, and/or Registries of Antiplatelet Agents–Section 5.2.
Evidence Table 15. Randomized Trials Comparing Statin Agents–Section 5.2.
Evidence Table 16. Nonrandomized Trials, Observational Studies, and/or Registries of Statin Agents–Section 5.2.
Evidence Table 17. RCTs for Antihypertensive Agents– Section 5.3.
Evidence Table 18. Nonrandomized Trials, Observational Studies, and/or Registries of Antihypertensive Agents–Section 5.3.
Evidence Table 19. RCTs for Smoking Cessation–Section 5.4.
Evidence Table 20. Nonrandomized Trials, Observational Studies, and/or Registries of Smoking Cessation–Section 5.4.
Evidence Table 21. RCTs Evaluating Glycemic Control in Patients with PAD and Diabetes Mellitus–Section 5.5.
Evidence Table 22. Nonrandomized Trials, Observational Studies, and/or Registries of Glycemic Control–Section 5.5.
Evidence Table 23. RCTs Evaluating Oral Anticoagulation–Section 5.6.
Evidence Table 24. Nonrandomized Trials, Observational Studies, and/or Registries of Oral Anticoagulation–Section 5.6.
Evidence Table 25. RCTs and Observational Studies of Cilostazol–Section 5.7.
Evidence Table 26. Nonrandomized Trials, Observational Studies, and/or Registries of Pentoxifylline–Section 5.8.
Evidence Table 27. Systematic Review of Chelation Therapy–Section 5.9.
Evidence Table 28. Nonrandomized Trials, Observational Studies, and/or Registries of Homocysteine Lowering Therapy for Lower Extremity PAD in Patients with Diabetes Mellitus–Section 5.10.1.
Evidence Table 29. RCTs Comparing Additional Medical Therapies of Homocysteine Lowering Therapy for Lower Extremity PAD–Section 5.10.1.
Evidence Table 30. RCTs for Influenza Vaccination–Section 5.10.2.
Evidence Table 31. Nonrandomized Trials for Influenza Vaccination–Section 5.10.2.
Evidence Table 32. RCTs for Exercise Therapy–Section 6.
Evidence Table 33. Nonrandomized Trials, Observational Studies, and/or Registries for Exercise Therapy–Section 6.
Evidence Table 34. Nonrandomized Trials and Observational Studies of Minimizing Tissue Loss in Patients with PAD–Section 7.
Data Supplement 34a. Functions of a Multidisciplinary Foot Care / Amputation Prevention Team–Section 7.
Evidence Table 35. RCTs Comparing Endovascular Treatment and Endovascular Versus Noninvasive Treatment of Claudication–Section 8.1.
Evidence Table 36. Nonrandomized Trials, Observational Studies, and/or Registries of Endovascular and Endovascular Versus Noninvasive Treatment of Claudication–Section 8.1.
Evidence Table 37. RCTs Evaluating Surgical Treatment for Claudication–Section 8.1.2.
Evidence Table 38. Nonrandomized Trials, Observational Studies, and/or Registries of Surgical Treatment for Claudication–Section 8.1.2.
Evidence Table 39. RCTs Comparing Endovascular Revascularization for Chronic CLI–Section 8.2.
Evidence Table 40. Nonrandomized Trials, Observational Studies, and/or Registries of Endovascular Revascularization for Chromic CLI–Section 8.2.1.
Evidence Table 41. RCTs of Surgical Revascularization for Chronic CLI–Section 8.2.
Evidence Table 42. Nonrandomized Trials, Observational Studies, and/or Registries for Surgical Revascularization for Chronic CLI–Section 8.2.
Evidence Table 43. RCT Comparing Prostanoids for End-Stage Peripheral Artery Disease–Section 8.2.3.
Evidence Table 44. Nonrandomized Trials, Observational Studies, and/or Registries for Would Healing Therapies for CLI–Section 8.2.3.
Evidence Table 45. Nonrandomized Trials, Observational Studies, and/or Registries of Acute Limb Ischemia–Section 9.1.
Evidence Table 46. Nonrandomized Trials, Observational studies, and/or Registries Comparing Evaluating Noninvasive Testing and Angiography for ALI–Section 9.1.
Evidence Table 47. RCTs of Revascularization Strategy for ALI–Section 9.2.2.
Evidence Table 48. Nonrandomized Trials, Observational Studies, and/or Registries of Clinical Presentation of ALI–Section 9.2.2.
Evidence Table 49. Nonrandomized Trials, Observational Studies, and/or Registries of Diagnostic Evaluation of the Cause of ALI–Section 9.2.2.
Evidence Table 50. Nonrandomized Trials, Observational Studies, and/or Registries of Revascularization Strategy for ALI–Section 9.2.2.
Evidence Table 51. RCTs for Longitudinal Follow-Up–Section 10.
Evidence Table 52. Nonrandomized Trials, Observational Studies, and/or Registries for Longitudinal Follow-Up–Section 10.
References.

## Figures and Tables

**Table 1:** ACC/AHA Recommendation System: Applying Class of Recommendation and Level of Evidence to Clinical Strategies, Interventions, Treatments, or Diagnostic Testing in Patient Care* (Updated August 2015)

CLASS (STRENGTH) OF RECOMMENDATION	
**CLASS 1 (STRONG)**	**Benefit >>> Risk**
Suggested phrases for writing recommendations: ■ Is recommended ■ Is indicated/useful/effective/beneficial ■ Should be performed/administered/other ■ Comparative-Effectiveness Phrases†: ○ Treatment/strategy A is recommended/indicated in preference to treatment B ○ Treatment A should be chosen over treatment B	
**CLASS IIa (MODERATE)**	**Benefit >> Risk**
Suggested phrases for writing recommendations; ■ Is reasonable ■ Can be useful/effective/beneficial ■ Comparative-Effectiveness Phrases†: ○ Treatment/strategy A is probably recommended/indicated in preference to treatment B ○ It is reasonable to choose treatment A over treatment B	
**CLASS IIb (WEAK)**	**Benefit ≥ Risk**
Suggested phrases for writing recommendations: ■ May/might be reasonable ■ May/might be considered ■ Usefulness/effectiveness is unknown/unclear/uncertain or not well established	
**CLASS III: No Benefit (MODERATE) (*Generally, LOE A or B use only*)**	**Benefit = Risk**
Suggested phrases for writing recommendations: ■ Is not recommended ■ Is not indicated/useful/effective/beneficial ■ Should not be performed/administered/other	
**CLASS III: Harm (STRONG)**	**Risk > Benefit**
Suggested phrases for writing recommendations: ■ Potentially harmful ■ Causes harm ■ Associated with excess morbidity/mortality ■ Should not be performed/administered/other	
**LEVEL (QUALITY) OF EVIDENCE**‡	
**LEVEL A**	
■ High-quality evidence‡ from more than 1 RCT ■ Meta-analyses of high-quality RCTs ■ One or more RCTs corroborated by high-quality registry studies	
**LEVEL B-R**	**(Randomized)**
■ Moderate-quality evidence‡ from 1 or more RCTs ■ Meta-analyses of moderate-quality RCTs	
**LEVEL B-NR**	**(Nonrandomized)**
■ Moderate-quality evidence‡ from 1 or more well-designed, well-executed nonrandomized studies, observational studies, or registry studies ■ Meta-analyses of such studies	
**LEVEL C-LD**	**(Limited Data)**
■ Randomized or nonrandomized observational or registry studies with limitations of design or execution ■ Meta-analyses of such studies ■ Physiological or mechanistic studies in human subjects	
**LEVEL C-EO**	**(Expert Opinion)**
Consensus of expert opinion based on clinical experience	

COR and LOE are determined independently (any COR may be paired with any LOE), A recommendation with LOE C does not imply that the recommendation is weak. Many important clinical questions addressed in guidelines do not lend themselves to clinical trials. Although RCTs are unavailable, there may be a very dear clinical consensus that a particular test or therapy is useful or effective.

* The outcome or result of the intervention should be specified (an improved clinical outcome or increased diagnostic accuracy or incremental prognostic information).

† For comparative-effectiveness recommendations (COR I and lla; LOE A and B only), studies that support the use of comparator verbs should involve direct comparisons of the treatments or strategies being evaluated.

‡ The method of assessing quality is evolving, including the application of standardized, widely used, and preferably validated evidence grading tools; and for systematic reviews, the incorporation of an Evidence Review Committee.

COR indicates Class of Recommendation; EO, expert opinion; LD, limited data; LOE, Level of Evidence; NR, nonrandomized; R, randomized; and RCT randomized controlled trial.

**Table 2:** Important Guideline Policy

Title	Organization	Publication Year (Reference)
ACC/AHA Guideline policy relevant to the management of lower extremity PAD		
Duration of dual antiplatelet therapy in patients with coronary artery disease	ACC/AHA	2016^20^
Perioperative cardiovascular evaluation and management of patients undergoing noncardiac surgery	ACC/AHA	2014^21^
Lifestyle management to reduce cardiovascular risk	AHA/ACC	2013^22^
Assessment of cardiovascular risk	ACC/AHA	2013^23^
Blood cholesterol to reduce atherosclerotic cardiovascular risk in adults	ACC/AHA	2013^24^
PAD (lower extremity, renal, mesenteric, and abdominal aortic)	ACC/AHA	2005^9^ and 2011^10^
Secondary prevention and risk-reduction therapy for patients with coronary and other atherosclerotic vascular disease	AHA/ACC	2011^25^
Other related publications		
Atherosclerotic occlusive disease of the lower extremities guideline	SVS	2015^26^
Measurement and interpretation of the ankle-brachial index	AHA	2012^27^
Cardiac disease evaluation and management among kidney and liver transplantation candidates	AHA/ACC	2012^28^
Intensive glycemic control and the prevention of cardiovascular events	ADA/ACC/AHA	2009^29^
Influenza vaccination as secondary prevention for cardiovascular disease	AHA/ACC	2006^30^
Indications for renal arteriography at the time of coronary arteriography	AHA/CLCD/CVRI/KCVD	2006^31^
Seventh Report of the Joint National Committee on Prevention, Detection, Evaluation, and Treatment of High Blood Pressure (JNC 7)*	NHLBI	2003^32^

* A revision to the current document is being prepared, with publication expected in 2017. The new title is expected to be “ACC/AHA/AAPA/ABC/ACPM/AGS/APhA/ASH/ASPC/NMA/PCNA Guideline for the Detection, Evaluation, Prevention and Management of High Blood Pressure.”

AAPA indicates American Academy of Physician Assistants; ABC, Association of Black Cardiologists; ACC, American College of Cardiology; ACPM, American College of Preventive Medicine; ADA, American Diabetes Association; AGS, American Geriatrics Society; AHA, American Heart Association; APhA, American Pharmacists Association; ASH, American Society of Hypertension; ASPC, American Society for Preventive Cardiology; CLCD, Council on Clinical Cardiology; CVRI, Council on Cardiovascular Radiology and Intervention; KCVD, Council on the Kidney in Cardiovascular Disease; NHLBI, National Heart, Lung, and Blood Institute; NMA, National Medical Association; PAD, peripheral artery disease; PCNA, Preventive Cardiovascular Nurses Association; and SVS, Society for Vascular Surgery.

**Table 3:** Definition of PAD Key Terms

Term	Definition
Claudication	Fatigue, discomfort, cramping, or pain of vascular origin in the muscles of the lower extremities that is consistently induced by exercise and consistently relieved by rest (within 10 min).
Acute limb ischemia (ALI)	Acute (<2 wk), severe hypoperfusion of the limb characterized by these features: pain, pallor, pulselessness, poikilothermia (cold), paresthesias, and paralysis. One of these categories of ALI is assigned (Section 10): Viable—Limb is not immediately threatened; no sensory loss; no muscle weakness; audible arterial and venous Doppler. Threatened—Mild-to-moderate sensory or motor loss; inaudible arterial Doppler; audible venous Doppler; may be further divided into IIa (marginally threatened) or IIb (immediately threatened). Irreversible—Major tissue loss or permanent nerve damage inevitable; profound sensory loss, anesthetic; profound muscle weakness or paralysis (rigor); inaudible arterial and venous Doppler.^33,34^
Tissue loss	Type of tissue loss: Minor—nonhealing ulcer, focal gangrene with diffuse pedal ischemia. Major—extending above transmetatarsal level; functional foot no longer salvageable.^33^
Critical limb ischemia (CLI)	A condition characterized by chronic (≥2 wk) ischemic rest pain, nonhealing wound/ulcers, or gangrene in 1 or both legs attributable to objectively proven arterial occlusive disease. The diagnosis of CLI is a constellation of both symptoms and signs. Arterial disease can be proved objectively with ABI, TBI, TcPO_2_, or skin perfusion pressure. Supplementary parameters, such as absolute ankle and toe pressures and pulse volume recordings, may also be used to assess for significant arterial occlusive disease. However, a very low ABI or TBI does not necessarily mean the patient has CLI. The term CLI implies chronicity and is to be distinguished from ALI.^35^
In-line blood flow	Direct arterial flow to the foot, excluding collaterals.
Functional status	Patient's ability to perform normal daily activities required to meet basic needs, fulfill usual roles, and maintain health and well-being. Walking ability is a component of functional status.
Nonviable limb	Condition of extremity (or portion of extremity) in which loss of motor function, neurological function, and tissue integrity cannot be restored with treatment.
Salvageable limb	Condition of extremity with potential to secure viability and preserve motor function to the weight-bearing portion of the foot if treated.
Structured exercise program	Planned program that provides individualized recommendations for type, frequency, intensity, and duration of exercise. Program provides recommendations for exercise progression to assure that the body is consistently challenged to increase exercise intensity and levels as functional status improves over time. There are 2 types of structured exercise program for patients with PAD: Supervised exercise program Structured community- or home-based exercise program
Supervised exercise program	Structured exercise program that takes place in a hospital or outpatient facility in which intermittent walking exercise is used as the treatment modality. Program can be standalone or can be made available within a cardiac rehabilitation program. Program is directly supervised by qualified healthcare provider(s). Training is performed for a minimum of 30 to 45 min per session, in sessions performed at least 3 times/wk for a minimum of 12 wk.^36–46^ Patients may not initially achieve these targets, and a treatment goal is to progress to these levels over time. Training involves intermittent bouts of walking to moderate-to-maximum claudication, alternating with periods of rest. Warm-up and cool-down periods precede and follow each session of walking.
Structured community- or home-based exercise program	Structured exercise program that takes place in the personal setting of the patient rather than in a clinical setting.^41,47–51^ Program is self-directed with the guidance of healthcare providers who prescribe an exercise regimen similar to that of a supervised program. Patient counseling ensures that patients understand how to begin the program, how to maintain the program, and how to progress the difficulty of the walking (by increasing distance or speed). Program may incorporate behavioral change techniques, such as health coaching and/or use of activity monitors.
Emergency versus urgent	An *emergency* procedure is one in which life or limb is threatened if the patient is not in the operating room or interventional suite and/or where there is time for no or very limited clinical evaluation, typically within <6 h. An *urgent* procedure is one in which there may be time for a limited clinical evaluation, usually when life or limb is threatened if the patient is not in the operating room or interventional suite, typically between 6 and 24 h.
Interdisciplinary care team	A team of professionals representing different disciplines to assist in the evaluation and management of the patient with PAD. For the care of patients with CLI, the interdisciplinary care team should include individuals who are skilled in endovascular revascularization, surgical revascularization, wound healing therapies and foot surgery, and medical evaluation and care. Interdisciplinary care team members may include: Vascular medical and surgical specialists (ie, vascular medicine, vascular surgery, interventional radiology, interventional cardiology) Nurses Orthopedic surgeons and podiatrists Endocrinologists Internal medicine specialists Infectious disease specialists Radiology and vascular imaging specialists Physical medicine and rehabilitation clinicians Orthotics and prosthetics specialists Social workers Exercise physiologists Physical and occupational therapists Nutritionists/dieticians
Cardiovascular ischemic events	Acute coronary syndrome (acute MI, unstable angina), stroke, or cardiovascular death.
Limb-related events	Worsening claudication, new CLI, new lower extremity revascularization, or new ischemic amputation.

ABI indicates ankle-brachial index; ALI, acute limb ischemia; CLI, critical limb ischemia; MI, myocardial infarction; PAD, peripheral artery disease; TBI, toe-brachial index; and TcPO_2_, transcutaneous oxygen pressure.

**Table 4:** Patients at Increased Risk of PAD

Age ≥65 y
Age 50–64 y, with risk factors for atherosclerosis (eg, diabetes mellitus, history of smoking, hyperlipidemia, hypertension) or family history of PAD^63^
Age <50 y, with diabetes mellitus and 1 additional risk factor for atherosclerosis
Individuals with known atherosclerotic disease in another vascular bed (eg, coronary, carotid, subclavian, renal, mesenteric artery stenosis, or AAA)

AAA indicates abdominal aortic aneurysm; PAD, peripheral artery disease.

**Table 5:** History and/or Physical Examination Findings Suggestive of PAD

History
Claudication
Other non–joint-related exertional lower extremity symptoms (not typical of claudication)
Impaired walking function
Ischemic rest pain
Physical Examination
Abnormal lower extremity pulse examination
Vascular bruit
Nonhealing lower extremity wound
Lower extremity gangrene
Other suggestive lower extremity physical findings (eg, elevation pallor/dependent rubor)

PAD indicates peripheral artery disease.

**Table 6:** Alternative Diagnoses for Leg Pain or Claudication With Normal Physiological Testing (Not PAD-Related)

Condition	Location	Characteristic	Effect of Exercise	Effect of Rest	Effect of Position	Other Characteristics
Symptomatic Baker's cyst	Behind knee, down calf	Swelling, tenderness	With exercise	Also present at rest	None	Not intermittent
Venous claudication	Entire leg, worse in calf	Tight, bursting pain	After walking	Subsides slowly	Relief speeded by elevation	History of iliofemoral deep vein thrombosis; edema; signs of venous stasis
Chronic compartment syndrome	Calf muscles	Tight, bursting pain	After much exercise (jogging)	Subsides very slowly	Relief with rest	Typically heavy muscled athletes
Spinal stenosis	Often bilateral buttocks, posterior leg	Pain and weakness	May mimic claudication	Variable relief but can take a long time to recover	Relief by lumbar spine flexion	Worse with standing and extending spine
Nerve root compression	Radiates down leg	Sharp lancinating pain	Induced by sitting, standing, or walking	Often present at rest	Improved by change in position	History of back problems; worse with sitting; relief when supine or sitting
Hip arthritis	Lateral hip, thigh	Aching discomfort	After variable degree of exercise	Not quickly relieved	Improved when not weight bearing	Symptoms variable; history of degenerative arthritis
Foot/ankle arthritis	Ankle, foot, arch	Aching pain	After variable degree of exercise	Not quickly relieved	May be relieved by not bearing weight	Symptoms variable; may be related to activity level or present at rest

Modified from Norgren L et al.^35^ PAD indicates peripheral artery disease.

**Table 7:** Alternative Diagnoses for Nonhealing Wounds With Normal Physiological Testing (Not PAD-Related)

Condition	Location	Characteristics and Causes
Venous ulcer	Distal leg, especially above medial mellolus	Develops in regions of skin changes due to chronic venous disease and local venous hypertension Typically wet (ie, wound drainage) rather than dry lesion
Distal small arterial occlusion (microangiopathy)	Toes, foot, leg	Diabetic microangiopathy End-stage renal disease Thromboangiitis obliterans (Buerger's) Sickle cell anemia Vasculitis (eg, Churg-Strauss, Henoch-Schonlein purpura, leukocytoclastic vasculitis, microscopic polyangiitis, polyarteritis nodosa) Scleroderma Cryoagglutination Embolic (eg, cholesterol emboli, thromboemboli, endocarditis) Thrombotic (eg, antiphospholipid antibody syndrome, Sneddon's syndrome, warfarin skin necrosis, disseminated intravascular coagulation, livedoid vasculitis, protein C or S deficiency, prolonged vasospasm)
Local injury	Toes, foot, leg	Trauma Insect or animal bite Burn
Medication related	Toes, foot, leg	Drug reactions (eg, erythema multiforme) Medication direct toxicity (eg, doxorubicin, hydroxyurea, some tyrosine kinase inhibitors)
Neuropathic	Pressure zones of foot	Hyperkeratosis surrounds the ulcer Diabetes mellitus with peripheral neuropathy Peripheral neuropathy without diabetes mellitus Leprosy
Autoimmune injury	Toes, foot, leg	With blisters (eg, pemphigoid, pemphigus, epidermolysis bullosa) Without blisters (eg, dermatomyositis, lupus, scleroderma)
Infection	Toes, foot, leg	Bacterial (eg, pseudomonas, necrotizing streptococcus) Fungal (eg, blastomycosis, Madura foot, chromomycosis) Mycobacterial Parasitic (eg, Chagas, leishmaniasis) Viral (eg, herpes)
Malignancy	Toes, foot, leg	Primary skin malignancy Metastatic malignancy Malignant transformation of ulcer
Inflammatory	Toes, foot, leg	Necrobiosis lipoidica Pyoderma gangrenosum Granuloma annulare

PAD indicates peripheral artery disease.

**Table 8:** Structured Exercise Programs for PAD: Definitions

Supervised exercise program (COR I, LOE A)
Program takes place in a hospital or outpatient facility.
Program uses intermittent walking exercise as the treatment modality.
Program can be standalone or within a cardiac rehabilitation program.
Program is directly supervised by qualified healthcare provider(s).
Training is performed for a minimum of 30–45 min/session; sessions are performed at least 3 times/wk for a minimum of 12 wk.^36–46^
Training involves intermittent bouts of walking to moderate-to-maximum claudication, alternating with periods of rest.
Warm-up and cool-down periods precede and follow each session of walking.
Structured community- or home-based exercise program (COR IIa, LOE A)
Program takes place in the personal setting of the patient rather than in a clinical setting.^41,47–51^
Program is self-directed with guidance of healthcare providers.
Healthcare providers prescribe an exercise regimen similar to that of a supervised program.
Patient counseling ensures understanding of how to begin and maintain the program and how to progress the difficulty of the walking (by increasing distance or speed).
Program may incorporate behavioral change techniques, such as health coaching or use of activity monitors.

COR indicates Class of Recommendation; LOE, Level of Evidence; and PAD, peripheral artery disease.

**Table 9:** Interdisciplinary Care Team for PAD

A team of professionals representing different disciplines to assist in the evaluation and management of the patient with PAD. For the care of patients with CLI, the interdisciplinary care team should include individuals who are skilled in endovascular revascularization, surgical revascularization, wound healing therapies and foot surgery, and medical evaluation and care.
Interdisciplinary care team members may include:
Vascular medical and surgical specialists (ie, vascular medicine, vascular surgery, interventional radiology, interventional cardiology)
Nurses
Orthopedic surgeons and podiatrists
Endocrinologists
Internal medicine specialists
Infectious disease specialists
Radiology and vascular imaging specialists
Physical medicine and rehabilitation clinicians
Orthotics and prosthetics specialists
Social workers
Exercise physiologists
Physical and occupational therapists
Nutritionists/dieticians

CLI indicates critical limb ischemia; and PAD, peripheral artery disease.

**Table 10:** Therapy for CLI: Findings That Prompt Consideration of Surgical or Endovascular Revascularization

Findings That Favor Consideration of Surgical Revascularization	Examples
Factors associated with technical failure or poor durability with endovascular treatment	Lesion involving common femoral artery, including origin of deep femoral artery
	Long segment lesion involving the below-knee popliteal and/or infrapopliteal arteries in a patient with suitable single-segment autogenous vein conduit
	Diffuse multilevel disease that would require endovascular revascularization at multiple anatomic levels
	Small-diameter target artery proximal to site of stenosis or densely calcified lesion at location of endovascular treatment
Endovascular treatment likely to preclude or complicate subsequent achievement of in-line blood flow through surgical revascularization	Single-vessel runoff distal to ankle
Findings That Favor Consideration of Endovascular Revascularization	Examples
The presence of patient comorbidities may place patients at increased risk of perioperative complications from surgical revascularization. In these patients, an endovascular-first approach should be used regardless of anatomy	Patient comorbidities, including coronary ischemia, cardiomyopathy, congestive heart failure, severe lung disease, and chronic kidney disease
Patients with rest pain and disease at multiple levels may undergo a staged approach as part of endovascular-first approach	In-flow disease can be addressed first, and out-flow disease can be addressed in a staged manner, when required, if clinical factors or patient safety prevent addressing all diseased segments at one setting
Patients without suitable autologous vein for bypass grafts	Some patients have had veins harvested for previous coronary artery bypass surgery and do not have adequate remaining veins for use as conduits. Similarly, patients may not have undergone prior saphenous vein harvest, but available vein is of inadequate diameter

CLI indicates critical limb ischemia.

**Appendix 1:** Author Relationships With Industry and Other Entities (Relevant)—2016 AHA/ACC Guideline on the Management of Patients With Lower Extremity Peripheral Artery Disease (March 2014)

Committee Member	Employment	Consultant	Speakers Bureau	Ownership/Partnership /Principal	Personal Research	Institutional, Organizational, or Other Financial Benefit	Expert Witness	Voting Recusals by Section*
Marie D. Gerhard-Herman, Chair	Harvard Medical School—Associate Professor	None	None	None	None	None	None	None
Heather L. Gornik, Vice Chair	Cleveland Clinic Foundation, Cardiovascular Medicine—Medical Director, Noninvasive Vascular Laboratory	None	None	Summit Doppler Systems Zin Medical	Astra Zeneca Theravasc	None	None	3.1, 3.2, 5.1–5.3, and 5.6.
Coletta Barrett	Our Lady of the Lake Regional Medical Center—Vice President	None	None	None	None	None	None	None
Neal R. Barshes	Baylor College of Medicine, Division of Vascular Surgery and Endovascular Therapy Michael E. DeBakey Department of Surgery—Assistant Professor	None	None	None	None	None	None	None
Matthew A. Corriere	University of Michigan—Frankel Professor of Cardiovascular Surgery, Associate Professor of Surgery	None	None	None	None	None	None	None
Douglas E. Drachman	Massachusetts General Hospital—Training Director	Abbott Vascular St. Jude Medical	None	None	Atrium Medical Bard Lutonix	None	None	4, 8.1.1–9.1.2, and 10.2.2.
Lee A. Fleisher	University of Pennsylvania Health System Department of Anesthesiology and Critical Care—Chair	None	None	None	None	None	None	None
Francis Gerry R. Fowkes	University of Edinburgh—Emeritus Professor of Epidemiology	AstraZeneca† Bayer Merck	None	None	None	None	None	5.1–5.3, 5.6, 5.10, 7, and 9.2.
Naomi M. Hamburg	Boston University School of Medicine, Cardiovascular Medicine Section—Associate Professor of Medicine	None	None	None	None	None	None	None
Scott Kinlay	VA Boston Healthcare System—Associate Chief, Cardiology Director, Cardiac Catheterization Laboratory & Vascular Medicine	None	None	None	Medtronic† The Medicines Company†	None	None	4, 5.6, 8.1.1, 9.1.1, 10.2.1, and 10.2.2.
Robert Lookstein	Mount Sinai Medical Center—Chief, Interventional Radiology; Professor of Radiology and Surgery; Vice Chair, Department of Radiology	Boston Scientific Medrad Interventional Possis The Medicines Company	Cordis‡	None	Shockwave (DSMB)	None	None	4, 5.6, 8.1.1, 9.1.1, 10.2.1, and 10.2.2.
Sanjay Misra	Mayo Clinic, Division of Vascular and Interventional Radiology—Professor; Department of Radiology— Interventional Radiologist	None	None	None	Johnson & Johnson (DSMB)	None	None	4, 7, 8, and 10.2.2.
Leila Mureebe	Duke University Medical Center—Associate Professor of Surgery, Division of Vascular Surgery	None	None	None	None	None	None	None
Jeffrey W. Olin	Ichan School of Medicine at Mount Sinai, Zena and Michael A. Wiener Cardiovascular Institute and Marie-Josée and Henry R. Kravis Center for Cardiovascular Health—Professor of Medicine, Cardiology; Director, Vascular Medicine	AstraZeneca Merck Novartis Plurestem	None	Northwind†	AstraZeneca†	None	None	5.1–5.3, 5.6, 5.10, and 12.
Rajan A.G. Patel	John Ochsner Heart & Vascular Center, Ochsner Clinical School, University of Queensland School of Medicine— Senior Lecturer	None	None	None	None	None	None	None
Judith G. Regensteiner	University of Colorado, Health Sciences Center, Division of Cardiology—Associate Professor of Medicine	None	None	None	None	None	None	None
Andres Schanzer	University of Massachusetts Medical School—Professor of Surgery and Quantitative Health Sciences; Program Director, Vascular Surgery Residency	Cook Medical	None	None	None	None	None	4, 8.1.1, 9.1.1, and 10.2.2.
Mehdi H. Shishehbor	Cleveland Clinic, Interventional Cardiology and Vascular Medicine— Director, Endovascular Services	Boston Scientific‡ Medtronic‡	None	None	None	Atrium Medical AstraZeneca†	None	4, 8.1.1– 9.1.2, and 10.2.2.
Kerry J. Stewart	Johns Hopkins University, School of Medicine; Johns Hopkins Bayview Medical Center— Professor of Medicine; Director, Clinical and Research Exercise Physiology	None	None	None	None	None	None	None
Diane Treat-Jacobson	University of Minnesota, School of Nursing— Professor	None	None	None	None	None	None	None
M. Eileen Walsh	University of Toledo, College of Nursing— Professor	None	None	None	None	None	None	None

This table represents the relationships of committee members with industry and other entities that were determined to be relevant to this document. These relationships were reviewed and updated in conjunction with all meetings and/or conference calls of the writing committee during the document development process. The table does not necessarily reflect relationships with industry at the time of publication. A person is deemed to have a significant interest in a business if the interest represents ownership of ≥5% of the voting stock or share of the business entity, or ownership of ≥$5000 of the fair market value of the business entity; or if funds received by the person from the business entity exceed 5% of the person's gross income for the previous year. Relationships that exist with no financial benefit are also included for the purpose of transparency. Relationships in this table are modest unless otherwise noted. According to the ACC/AHA, a person has a relevant relationship IF: a) the relationship or interest relates to the same or similar subject matter, intellectual property or asset, topic, or issue addressed in the document; or b) the company/entity (with whom the relationship exists) makes a drug, drug class, or device addressed in the document, or makes a competing drug or device addressed in the document; or c) the person or a member of the person's household, has a reasonable potential for financial, professional, or other personal gain or loss as a result of the issues/content addressed in the document.

* Writing committee members are required to recuse themselves from voting on sections to which their specific relationships with industry and other entities may apply.

† Significant relationship.

‡ No financial benefit.

ACC indicates American College of Cardiology; AHA, American Heart Association; DSMB, data safety monitoring board; and VA, Veterans Affairs.

**Appendix 2:** Reviewer Relationships With Industry and Other Entities (Comprehensive)—2016 AHA/ACC Guideline on the Management of Patients With Lower Extremity Peripheral Artery Disease (March 2016)

Reviewer	Representation	Employment	Consultant	Speakers Bureau	Ownership/Partnership /Principal	Personal Research	Institutional,Organizational, or Other FinancialBenefit	Expert Witness
Deepak L. Bhatt	Official Reviewer—ACC Board of Trustees	Brigham and Women's Hospital— Executive Director of Interventional Cardiovascular Programs; Harvard Medical School—Professor of Medicine	Elsevier	None	None	Amarin* Amgen* AstraZeneca* Bristol-Myers Squibb* Cardax† Eisai* Ethicon* FlowCo† Forest Laboratories* Ischemix* Mayo Clinic Medtronic* Merck† Pfzer* PLx Pharma† Regado Biosciences† Roche* Sanof-aventis* St. Jude Medical Takeda† The Medicines Company* WebMD*	Belvoir Publications (Editor)* Biotronik Boston Scientific Clinical Cardiology(Deputy Editor)† Harvard Clinical Research Institute HMP Communications (Editor)* Duke Clinical Research Institute* Journal of Invasive Cardiology (Editor)* Medscape Cardiology Slack Publications (Editor)* St. Jude Medical VA Healthcare System†	None
Mark A. Creager	Official Reviewer—AHA	Dartmouth-Hitchcock Medical Center— Director	None	None	None	None	AHA (Past President)†	None
Philip Goodney	Official Reviewer—AHA	Dartmouth-Hitchcock—Associate Professor of Surgery and The Dartmouth Institute Director	None	None	None	NIH*	NIH	None
John S. Ikonomidis	Official Reviewer—ACC/ AHA Task Force on Clinical Practice Guidelines	Medical University of South Carolina—Chief	None	None	None	None	None	None
Amy W. Pollak	Official Reviewer—AHA	Mayo Clinic—Cardiovascular Medicine Physician	None	None	None	None	None	None
Michael D. White	Official Reviewer—ACC Board of Governors	Catholic Health Initiatives—Chief Academic Officer	Anthera Pharmaceuticals†	None	None	AstraZeneca†	None	None
Ehrin J. Armstrong	Organizational Reviewer—SVM	University of Colorado—Director, Interventional Cardiology	Abbott Medtronic Merck Spectranetics	None	None	None	None	None
Bernadette Aulivola	Organizational Reviewer—VESS	Loyola University medical Center, Stritch School of Medicine—Director, Division of Vascular Surgery and Endovascular Therapy; Associate Professor, Department of Surgery; Program Director, Vascular Surgery Fellowship; Medical Director, Vascular Noninvasive lab	None	None	None	None	None	None
Alison Bailey	Organizational Reviewer—AACVPR	University of Tennessee Chattanooga—Cardiologist	None	None	None	CSL Behring	AACVPR† ZOLL Medical	None
Todd Brown	Organizational Reviewer—AACVPR	University of Alabama at Birmingham—Associate Professor	None	None	None	Amgen* Omthera† NIH*	None	None
Kristen Columbia	Organizational Reviewer—SVN	University of Maryland Baltimore Washington Medical Center, Maryland Vascular Center—Nurse practitioner	None	None	None	None	None	None
Michael S. Conte	Organizational Reviewer—SVS	University of California San Francisco—Professor and Chief	Cook Medical Medtronic	None	None	Bard	University of California Department of Surgery	None
Alik Farber	Organizational Reviewer—SCVS	Boston Medical Center—Chief, Division of Vascular Surgery	Bard†	None	None	None	None	None
Robert Feezor	Organizational Reviewer—VESS	University of Florida—Associate Professor of Surgery, Division of Vascular Surgery and Endovascular Therapy	Cook Medical* Medtronic Terumo	None	None	Cook Medical	Cook Medical Novate	Defendant, peripheral angioplasty, 2015
Dmitriy N. Feldman	Organizational Reviewer—SCAI	Weill Cornell Medical College, New York Presbyterian Hospital—Associate Professor of Medicine	AstraZeneca	Abbott Bristol-Myers Squibb† Daiichi-Sankyo Eli Lilly Medtronic Pfizer The Medicines Company	None	None	Biotronic The Medicines Company	None
Jonathan Golledge	Organizational Reviewer—TASC	James Cook University—Professor, Department of Surgery, Head of Vascular Biology Unit	None	None	None	James Cook University*	None	None
Bruce H. Gray	Organizational Reviewer—SCAI	Greenville Health System—Director of Clinical Trials, Department of Surgery	None	Medtronic†	None	Abbott† W.L. Gore†	NCDR† ACC†	None
William R. Hiatt	Organizational Reviewer—TASC	Colorado Prevention Center—Professor of Medicine	None	None	None	AstraZeneca* Bayer* CSI Kowa Kyushu University Merck Pluristem* ReNeuron	CPC Clinical Research* NIH*	None
Joseph Mills	Organizational Reviewer—SVS	Baylor College of Medicine—Professor and Chief, Division of Vascular surgery and Endovascular Therapy	None	None	None	None	AnGes Bayer Cesca	None
Mohammad Reza Rajebi	Organizational Reviewer—SIR	University of Colorado Denver— Assistant Professor	None	None	None	None	None	None
Mitchell J. Silver	Organizational Reviewer—SVM	McConnell Heart Hospital for Critical Limb Care—Director of Vascular Imaging	Boston Scientific W.L. Gore Medtronic	Bristol-Myers Squibb* Pfzer*	Contego Medical*	None	W.L. Gore Medtronic NIH	None
Lily Thomson	Organizational Reviewer—SVN	Hôpital St-Boniface Hospital—Clinical Research Coordinator, Vascular Surgery Nurse, Section of Vascular Surgery, Health Sciences Centre	None	None	None	None	None	None
Sana M. Al-Khatib	Content Reviewer—ACC/ AHA Task Force on Clinical Practice Guidelines	Duke Clinical Research Institute—Associate Professor of Medicine	None	None	None	FDA* NHLBI* PCORI* VA (DSMB)	HRS (Board of Trustees)† Elsevier*	None
Herbert Aronow	Content Reviewer—ACC Peripheral Vascular Disease Member Section	Rhode Island Hospital—Director of Cardiac Catheterization Laboratories	None	None	None	Silk Road Medical† Saint Luke's Health System The Medicines Company†	Bard NIH PCORI† SVM† W.L. Gore	
Joshua A. Beckman	Content Reviewer	Vanderbilt University Medical Center— Director	AstraZeneca* Merck* Sanof*	None	EMX† JanaCare†	Bristol-Myers Squibb* Merck* NIH	Vascular Interventional Advances	Defendant, venous thrombo-embolism, 2015*
James C. Blankenship	Content Reviewer	Geisinger Medical Center—Staff Physician; Director, Cardiac Catheterization Laboratory	None	None	None	Abbott† AstraZeneca† Boston Scientific† GlaxoSmithKline† Hamilton Health Sciences† Medinal LTD† Orexigen Therapeutics† St. Jude Medical† Stentys† Takeda Pharmaceuticals†	SCAI (Past President)† AMA†	None
Biykem Bozkurt	Content Reviewer—ACC/AHA Task Force on Clinical Practice Guidelines	Michael E. DeBakey VA Medical Center—The Mary and Gordon Cain Chair and Professor of Medicine	None	None	None	Novartis	None	None
Joaquin E. Cigarroa	Content Reviewer—ACC/AHA Task Force on Clinical Practice Guidelines	Oregon Health and Science University—Clinical Professor of Medicine	None	None	None	None	ACC/AHA† AHA† ASA† Catheterization and Cardiovascular Intervention† Portland Metro Area AHA(President)† SCAI Quality Interventional Council† NIH	None
Federico Gentile	Content Reviewer—ACC/AHA Task Force on Clinical Practice Guidelines	Centro Medico Diagnostico—Director, Cardiovascular Disease	None	None	None	None	None	None
Anuj Gupta	Content Reviewer—ACC Peripheral Vascular Disease Member Section	University of Maryland—Assistant Professor of Medicine	None	None	None	Seimens* Medtronic†	Direct Flow Medical† Edwards†	None
John Jeb Hallett	Content Reviewer	Medical University of South Carolina—Clinical Professor of Surgery	None	None	None	None	None	None
Alan Hirsch	Content Reviewer	University of Minnesota Medical School—Professor of Medicine, Epidemiology and Community Health, and Director Vascular Medicine Program	Merck* Novartis†	None	None	Bayer* Pluristem (PLX-PAD trial-PI)† AstraZeneca (EUCLID trial–PI)† Pluristem*	AHA† Tactile Medical*	None
Mark A. Hlatky	Content Reviewer—ACC/AHA Task Force on Clinical Practice Guidelines	Stanford University School of Medicine—Professor of Health Research and Policy, Professor of Medicine	Acumen* Genentech	None	None	Blue Cross/Blue Shield Center for Effectiveness Evaluation* George Institute HeartFlow* NHLBI Sanofi-aventis	ACC (Associate Editor)*	None
Michael R. Jaff	Content Reviewer	Newton-Wellesley Hospital; Harvard Medical School— Professor of Medicine	AOPA Cardinal Health Covidien† Micell Vascular Therapies	None	MC10† Janacare† Northwind PQ Bypass Primacea SanoV Valiant Medical	Abbott† Boston Scientific† Cordis† IC Sciences Medtronic† Novello	CBSET Intersocietal Accreditation Commission SCAI† VIVA Physicians Group*	None
José A. Joglar	Content Reviewer—ACC/AHA Task Force on Clinical Practice Guidelines	UT Southwestern Medical Center—Professor of Internal Medicine; Clinical Cardiac Electrophysiology—Fellowship Program Director	None	None	None	None	None	None
Glenn N. Levine	Content Reviewer—ACC/AHA Task Force on Clinical Practice Guidelines	Baylor College of Medicine—Professor of Medicine; Director, Cardiac Care Unit	None	None	None	None	None	None
Khusrow Niazi	Content Reviewer—ACC Peripheral Vascular Disease Member Section	Emory University Department of Medicine—Associate Professor of Medicine	None	Medtronic*	None	Bard Impeto Terumo	None	Plaintiff, MI resulting in death, 2015*
Paul D. Varosy	Content Reviewer—Task Force on Performance Measures	VA Eastern Colorado Health Care System—Associate Professor	None	None	None	VA Health Services Research and Development (PI)*	AHA (Guest Editor)†	None
Christopher J. White	Content Reviewer	Ochsner Clinical School, University of Queensland—Chairman, Department of Cardiology	Neovasc	None	None	AstraZeneca Pharmaceuticals NIH Neovasc Surmodics	ACE (Board of Directors)†	None

This table represents all relationships of reviewers with industry and other entities that were reported by authors, including those not deemed to be relevant to this document, at the time this document was under development. The table does not necessarily reflect relationships with industry at the time of publication. A person is deemed to have a significant interest in a business if the interest represents ownership of ≥5% of the voting stock or share of the business entity, or ownership of ≥$5000 of the fair market value of the business entity; or if funds received by the person from the business entity exceed 5% of the person's gross income for the previous year. Relationships that exist with no financial benefit are also included for the purpose of transparency. Relationships in this table are modest unless otherwise noted. Please refer to http://www.acc.org/guidelines/about-guidelines-and-clinical-documents/ relationships-with-industry-policy for definitions of disclosure categories or additional information about the ACC/AHA Disclosure Policy for Writing Committees.

* Significant relationship.

† No financial benefit.

AACVPR indicates American Association of Cardiovascular and Pulmonary Rehabilitation; ACC, American College of Cardiology; ACE, Accreditation for Cardiovascular Excellence; AHA, American Heart Association; AMA, American Medical Association; DSMB, data and safety monitoring board; EUCLID, Effects of Ticagrelor and Clopidogrel in Patients with Peripheral Artery Disease; FDA, US Food and Drug Administration; HRS, Heart Rhythm Society; MI, myocardial infarction; NCDR, National Cardiovascular Data Registry; NIH, National Institutes of Health; NHLBI, National Heart, Lung, and Blood Institute; PCORI, Patient-Centered Outcomes Research Institute; PI, primary investigator; PLX-PAD, placental-derived adherent stromal cell; SCAI, Society for Cardiovascular Angiography and Interventions; SCVS, Society for Clinical Vascular Surgery; SIR, Society of Interventional Radiology; SVM, Society for Vascular Medicine; SVN, Society for Vascular Nursing; SVS, Society for Vascular Surgery; TASC, Trans-Atlantic Inter-Society Consensus for the Management of Peripheral Arterial Disease; VA, Veterans Affairs; VESS, Vascular and Endovascular Surgery Society; and VIVA, Vascular Intervention Advances.

**Appendix 3:** Abbreviations

AAA = abdominal aortic aneurysm
ABI = ankle-brachial index
ALI = acute limb ischemia
CAD = coronary artery disease
CLI = critical limb ischemia
CTA = computed tomography angiography
DAPT = dual antiplatelet therapy
DES = drug-eluting stent(s)
GDMT = guideline-directed management and therapy
MI = myocardial infarction
MRA = magnetic resonance angiography
PAD = peripheral artery disease
PTA = percutaneous transluminal angioplasty
RCT = randomized controlled trial
SPP = skin perfusion pressure
TBI = toe-brachial index
TcPO_2_ = transcutaneous oxygen pressure
QoL = quality of life

## Appendix

The American Heart Association requests that this document be cited as follows: Gerhard-Herman MD, Gornik HL, Barrett C, Barshes NR, Corriere MA, Drachman DE, Fleisher LA, Fowkes FGR, Hamburg NM, Kinlay S, Lookstein R, Misra S, Mureebe L, Olin JW, Patel RAG, Regensteiner JG, Schanzer A, Shishehbor MH, Stewart KJ, Treat-Jacobson D, Walsh ME. 2016 AHA/ACC guideline on the management of patients with lower extremity peripheral artery disease: a report of the American College of Cardiology/American Heart Association Task Force on Clinical Practice Guidelines.

## ACC/AHA Task Force Members

Jonathan L. Halperin, MD, FACC, FAHA, Chair; Glenn N. Levine, MD, FACC, FAHA, Chair-Elect; Sana M. Al-Khatib, MD, MHS,FACC, FAHA; Kim K. Birtcher, PharmD, MS, AACC; Biykem Bozkurt, MD, PhD, FACC, FAHA; Ralph G. Brindis, MD, MPH, MACC; Joaquin E. Cigarroa, MD, FACC; Lesley H. Curtis, PhD, FAHA; Lee A. Fleisher, MD, FACC, FAHA; Federico Gentile, MD, FACC; Samuel Gidding, MD, FAHA; Mark A. Hlatky, MD, FACC; John Ikonomidis, MD, PhD, FAHA; José Joglar, MD, FACC, FAHA; Susan J. Pressler, PhD, RN, FAHA; Duminda N. Wijeysundera, MD, PhD

## Presidents and Staff

**American College of Cardiology**: Richard A. Chazal, MD, FACC, President

Shalom Jacobovitz, Chief Executive Officer

William J. Oetgen, MD, MBA, FACC, Executive Vice President, Science, Education, Quality, and Publishing

Amelia Scholtz, PhD, Publications Manager, Science, Education, Quality, and Publishing

**American College of Cardiology/American Heart Association**: Katherine Sheehan, PhD, Director, Guideline Strategy and Operations

Lisa Bradfield, CAE, Director, Guideline Methodology and Policy

Abdul R. Abdullah, MD, Associate Science and Medicine Advisor

Allison Rabinowitz, MPH, Project Manager, Clinical Practice Guidelines

**American Heart Association**: Steven R. Houser, PhD, FAHA, President

Nancy Brown, Chief Executive Officer

Rose Marie Robertson, MD, FAHA, Chief Science and Medicine Officer

Gayle R. Whitman, PhD, RN, FAHA, FAAN, Senior Vice President, Office of Science Operations

Comilla Sasson, MD, PhD, FACEP, Vice President, Science and Medicine

Jody Hundley, Production Manager, Scientific Publications, Office of Science Operations

## Appendix

The Data Supplement is available with this article at http://circ.ahajournals.org/lookup/suppl/doi:10.1161/CIR.0000000000000471/-/DC2.

## Appendix

Copies: This document is available on the World Wide Web sites of the American College of Cardiology (www.acc.org) and the American Heart Association (professional.heart.org). A copy of the document is available at http://professional.heart. org/statements by selecting either the “By Topic” link or the “By Publication Date” link. To purchase additional reprints, call 843-216-2533 or kelle.ramsay@wolterskluwer.com.

## Appendix

Expert peer review of AHA Scientific Statements is conducted by the AHA Office of Science Operations. For more on AHA statements and guidelines development, visit http://professional.heart.org/statements and select the “Policies and Development” link.
