# Supplementary material for: 2016 AHA/ACC Guideline on the Management of Patients With Lower Extremity Peripheral Artery Disease: A Report of the American College of Cardiology/American Heart Association Task Force on Clinical Practice Guidelines
Source: Circulation. Author manuscript; Available in PMC 2017 Sep 21. (PMC5477786; doi:10.1161/CIR.0000000000000471)
Supplement: Supplementary file 1 — Evidence Table 1. Nonrandomized Trials, Observational Studies, and/or Registries of History for Clinical Assessment for PAD–Section 2.1. Evidence Table 2. Nonrandomized Trials, Observational Studies, and/or Registries of Physical Examination for Clinical Assessment for PAD–Section 2.1. Evidence Table 3. RCTs of Resting ABI for Diagnosing PAD–Section 3.1. Evidence Table 4. Nonrandomized Trials, Observational Studies, and/or Registries of Resting ABI for Diagnosing PAD–Section 3.1. Evidence Table 5. Nonrandomized Trials, Observational Studies, and/or Registries of Physiological Testing–Section 3.2. Evidence Table 6. Nonrandomized Trials, Observational Studies, and/or Registries of Imaging for Anatomic Assessment (Ultrasound, CTA, MRA, Angiography)–Section 3.3. Evidence Table 7. RCTs of Imaging for Anatomic Assessment (Ultrasound, CTA, MRA, Angiography)–Section 3.3. Evidence Table 8. Nonrandomized Trials, Observational Studies, and/or Registries for Abdominal Aortic Aneurysm–Section 4.1. Evidence Table 9. Nonrandomized Trials, Observational Studies, and/or Registries of Coronary Artery Disease Screening in PAD–Section 4.2. Evidence Table 10. RCTs for CAD Screening in PAD–Section 4.2. Evidence Table 11. Nonrandomized Trials, Observational Studies, and/or Registries of Screening in Carotid Artery Disease–Section 4.3. Evidence Table 12. Nonrandomized Trials, Observational Studies, and/or Registries for Renal Artery Disease–Section 4.4. Evidence Table 13. RCTs Evaluating Antiplatelet Agents– Section 5.1. Evidence Table 14. Nonrandomized Trials, Observational Studies, and/or Registries of Antiplatelet Agents–Section 5.2. Evidence Table 15. Randomized Trials Comparing Statin Agents–Section 5.2. Evidence Table 16. Nonrandomized Trials, Observational Studies, and/or Registries of Statin Agents–Section 5.2. Evidence Table 17. RCTs for Antihypertensive Agents– Section 5.3. Evidence Table 18. Nonrandomized Trials, Observational Studies, and/or Registries of Antihypertensive Agent [file NIHMS867255-supplement-supplement_1.pdf]

Author Relationships With Industry and Other Entities (Comprehensive)— 2016 AHA/ACC Guideline on the Management of Patients With Lower Extremity Peripheral Artery Disease (March 2015)

Committee Member	Employment	Consultant	Speakers Bureau	Ownership/ Partnership/ Principal	Personal Research	Institutional, Organizational, or Other Financial Benefit	Expert Witness
Marie D. Gerhard-Herman *(Chair)*	Harvard Medical School—Associate Professor	None	None	None	• Progeria Research Foundation*	• American Board of Internal Medicine Cardiovascular Exam Committee	None
Heather L. Gornik *(Vice Chair)*	Cleveland Clinic Foundation, Cardiovascular Medicine—Medical Director, Noninvasive Vascular Laboratory	None	None	• Summit Doppler Systems<br>• Zin Medical	• AstraZeneca<br>• Theravasc	• Fibromuscular Dysplasia Society of America†<br>• IAC Vascular Testing<br>• Intersocietal Commission for the Accreditation of Vascular Laboratories†<br>• PCORI<br>• Society of Vascular Medicine†	None
Coletta Barrett	Our Lady of the Lake Regional Medical Center—Vice President	None	None	None	None	None	None
Neil R. Barshes	Baylor College of Medicine, Division of Vascular Surgery and Endovascular Therapy Michael E. DeBakey Department of Surgery—Assistant Professor	None	None	None	None	None	None
Matthew A. Corriere	University of Michigan—Frankel Professor of Cardiovascular Surgery, Associate Professor of Surgery	None	None	None	None	• Vascular Cures and Society for Vascular Surgery Foundation*	None
Douglas E. Drachman	Massachusetts General Hospital—Training Director	• Abbott Vascular<br>• Corindus Vascular Robotics<br>• St. Jude Medical	None	None	• Atrium Medical<br>• Bard<br>• iDEV Technologies<br>• Lutonix<br>• PLC Medical Systems<br>• Prairie Education and Research Cooperative	None	None
Lee Fleisher	University of Pennsylvania Health System Department of Anesthesiology and Critical Care—Chair	• Blue Cross/Blue Shield Association-Medical Advisory Panel to the Technology Evaluation Center	None	None	• Johns Hopkins Medical Institutions	• Association of University Anesthesiologists†<br>• Foundation for Anesthesia Education and Research†	None

Committee Member	Employment	Consultant	Speakers Bureau	Ownership/ Partnership/ Principal	Personal Research	Institutional, Organizational, or Other Financial Benefit	Expert Witness
		• National Quality Forum					
Francis Gerry R. Fowkes	University of Edinburgh—Emeritus Professor of Epidemiology	• AstraZeneca*<br>• Bayer<br>• Merck	None	None	None	None	None
Naomi M. Hamburg	Boston University School of Medicine, Cardiovascular Medicine Section—Associate Professor of Medicine	• Acceleron Pharma*	None	None	• Everest Genomics*<br>• Hershey's*<br>• Unex*<br>• Welch's	• Intersocietal Commission for the Accreditation of Vascular Laboratories‡	None
Scott Kinlay	VA Boston Healthcare System—Associate Chief, Cardiology Director, Cardiac Catheterization Laboratory & Vascular Medicine	None	None	None	• Medtronic*<br>• The Medicines Company*	None	None
Robert Lookstein	Mount Sinai Medical Center—Chief, Interventional Radiology; Professor of Radiology and Surgery; Vice Chair, Department of Radiology	• Boston Scientific<br>• Medrad Interventional<br>• Possis<br>• The Medicines Company	• Cordis†	None	• Veniti<br>• Shockwave (DSMB)	None	None
Sanjay Misra	Mayo Clinic, Division of Vascular and Interventional Radiology—Professor; Department of Radiology—Interventional Radiologist	None	None	None	• Johnson & Johnson (DSMB)	None	None
Leila Mureebe	Duke University Medical Center—Associate Professor of Surgery, Division of Vascular Surgery	None	None	None	None	None	None
Jeffrey W. Olin	Ichan School of Medicine at Mount Sinai, Zena and Michael A. Wiener Cardiovascular Institute and Marie-Josée and Henry R. Kravis Center for Cardiovascular Health—Professor of Medicine, Cardiology; Director, Vascular Medicine	• AstraZeneca<br>• Fibromuscular Dysplasia Society of America†<br>• Merck<br>• Novartis<br>• Pluremstem	None	• Northwind*	• AstraZeneca*	None	None
Rajan Patel	John Ochsner Heart & Vascular Center, Ochsner Clinical School, University of Queensland School of Medicine—Senior Lecturer	None	None	None	• Aastrom<br>• Abbott<br>• NHLBI	• Abiomed	None

Committee Member	Employment	Consultant	Speakers Bureau	Ownership/ Partnership/ Principal	Personal Research	Institutional, Organizational, or Other Financial Benefit	Expert Witness
Judith G. Regensteiner	University of Colorado, Health Sciences Center, Division of Cardiology—Associate Professor of Medicine	None	None	None	None	None	None
Andres Schanzer	University of Massachusetts Medical School—Professor of Surgery and Quantitative Health Sciences; Program Director, Vascular Surgery Residency	• Cook Medical	None	None	None	None	None
Mehdi H. Shishehbor	Cleveland Clinic, Interventional Cardiology and Vascular Medicine—Director, Endovascular Services	• Boston Scientific† • Medtronic†	None	None	None	• Astra Zeneca • NIH • Volcano/Philips	None
Kerry J. Stewart	Johns Hopkins University, School of Medicine; Johns Hopkins Bayview Medical Center—Professor of Medicine; Director, Clinical and Research Exercise Physiology	None	None	None	• Society of Women's Health Research*	None	None
Diane Treat-Jacobson	University of Minnesota, School of Nursing—Professor	None	None	None	• NHLBI*	None	None
M. Eileen Walsh	University of Toledo, College of Nursing—Professor	None	None	None	None	• American Nurses Credentialing Center† • Journal of Vascular Nursing (Editorial Board)†	None

This table represents all relationships of committee members with industry and other entities that were reported by authors, including those not deemed to be relevant to this document, at the time this document was under development. The table does not necessarily reflect relationships with industry at the time of publication. A person is deemed to have a significant interest in a business if the interest represents ownership of ≥5% of the voting stock or share of the business entity, or ownership of ≥$5,000 of the fair market value of the business entity; or if funds received by the person from the business entity exceed 5% of the person's gross income for the previous year. Relationships that exist with no financial benefit are also included for the purpose of transparency. Relationships in this table are modest unless otherwise noted. Please refer to http://www.acc.org/guidelines/about-guidelines-and-clinical-documents/guidelines-and-documents-task-forces for definitions of disclosure categories or additional information about the ACC/AHA Disclosure Policy for Writing Committees.

*Significant relationship.
†No financial benefit.

DSMB indicates data safety monitoring board; IAC, Intersocietal Accreditation Commission; NHLBI, National Heart, Lung, and Blood Institute; NIH, National Institute of Health; and PCORI, Patient-Centered Outcomes Research Institute.

2016 AHA/ACC Guideline on the Management of Patients With Lower Extremity Peripheral Artery Disease Data Supplements

(Section numbers correspond to the full-text guideline.)

Table of Contents

Evidence Table 1. Nonrandomized Trials, Observational Studies, and/or Registries of History for Clinical Assessment for PAD–Section 2.1. ... 3
Evidence Table 2. Nonrandomized Trials, Observational Studies, and/or Registries of Physical Examination for Clinical Assessment for PAD–Section 2.1. ... 9
Evidence Table 3. RCTs of Resting ABI for Diagnosing PAD–Section 3.1. ... 12
Evidence Table 4. Nonrandomized Trials, Observational Studies, and/or Registries of Resting ABI for Diagnosing PAD–Section 3.1. ... 13
Evidence Table 5. Nonrandomized Trials, Observational Studies, and/or Registries of Physiological Testing–Section 3.2. ... 23
Evidence Table 6. Nonrandomized Trials, Observational Studies, and/or Registries of Imaging for Anatomic Assessment (Ultrasound, CTA, MRA, Angiography)–Section 3.3. ... 28
Evidence Table 7. RCTs of Imaging for Anatomic Assessment (Ultrasound, CTA, MRA, Angiography)–Section 3.3. ... 34
Evidence Table 8. Nonrandomized Trials, Observational Studies, and/or Registries for Abdominal Aortic Aneurysm–Section 4.1. ... 37
Evidence Table 9. Nonrandomized Trials, Observational Studies, and/or Registries of Coronary Artery Disease Screening in PAD–Section 4.2. ... 38
Evidence Table 10. RCTs for CAD Screening in PAD–Section 4.2. ... 39
Evidence Table 11. Nonrandomized Trials, Observational Studies, and/or Registries of Screening in Carotid Artery Disease–Section 4.3. ... 39
Evidence Table 12. Nonrandomized Trials, Observational Studies, and/or Registries for Renal Artery Disease–Section 4.4. ... 40
Evidence Table 13. RCTs Evaluating Antiplatelet Agents– Section 5.1. ... 41
Evidence Table 14. Nonrandomized Trials, Observational Studies, and/or Registries of Antiplatelet Agents–Section 5.2. ... 56
Evidence Table 15. Randomized Trials Comparing Statin Agents–Section 5.2. ... 56
Evidence Table 16. Nonrandomized Trials, Observational Studies, and/or Registries of Statin Agents–Section 5.2. ... 59
Evidence Table 17. RCTs for Antihypertensive Agents– Section 5.3. ... 61
Evidence Table 18. Nonrandomized Trials, Observational Studies, and/or Registries of Antihypertensive Agents–Section 5.3. ... 64
Evidence Table 19. RCTs for Smoking Cessation–Section 5.4. ... 64
Evidence Table 20. Nonrandomized Trials, Observational Studies, and/or Registries of Smoking Cessation–Section 5.4. ... 67
Evidence Table 21. RCTs Evaluating Glycemic Control in Patients with PAD and Diabetes Mellitus–Section 5.5. ... 68
Evidence Table 22. Nonrandomized Trials, Observational Studies, and/or Registries of Glycemic Control–Section 5.5. ... 69
Evidence Table 23. RCTs Evaluating Oral Anticoagulation–Section 5.6. ... 71
Evidence Table 24. Nonrandomized Trials, Observational Studies, and/or Registries of Oral Anticoagulation–Section 5.6. ... 73
Evidence Table 25. RCTs and Observational Studies of Cilostazol–Section 5.7. ... 74
Evidence Table 26. Nonrandomized Trials, Observational Studies, and/or Registries of Pentoxifylline–Section 5.8. ... 77
Evidence Table 27. Systematic Review of Chelation Therapy–Section 5.9. ... 79
Evidence Table 28. Nonrandomized Trials, Observational Studies, and/or Registries of Homocysteine Lowering Therapy for Lower Extremity PAD in Patients with Diabetes Mellitus–Section 5.10.1. ... 79
Evidence Table 29. RCTs Comparing Additional Medical Therapies of Homocysteine Lowering Therapy for Lower Extremity PAD–Section 5.10.1. ... 80
Evidence Table 30. RCTs for Influenza Vaccination–Section 5.10.2. ... 81
Evidence Table 31. Nonrandomized Trials for Influenza Vaccination–Section 5.10.2. ... 82
Evidence Table 32. RCTs for Exercise Therapy–Section 6. ... 82
Evidence Table 33. Nonrandomized Trials, Observational Studies, and/or Registries for Exercise Therapy–Section 6. ... 91
Evidence Table 34. Nonrandomized Trials and Observational Studies of Minimizing Tissue Loss in Patients with PAD–Section 7. ... 92

© American Heart Association, Inc. and American College of Cardiology Foundation

Data Supplement 34a. Functions of a Multidisciplinary Foot Care / Amputation Prevention Team–Section 7. 96
Evidence Table 35. RCTs Comparing Endovascular Treatment and Endovascular Versus Noninvasive Treatment of Claudication–Section 8.1. 97
Evidence Table 36. Nonrandomized Trials, Observational Studies, and/or Registries of Endovascular and Endovascular Versus Noninvasive Treatment of Claudication–Section 8.1. 120
Evidence Table 37. RCTs Evaluating Surgical Treatment for Claudication–Section 8.1.2. 123
Evidence Table 38. Nonrandomized Trials, Observational Studies, and/or Registries of Surgical Treatment for Claudication–Section 8.1.2. 129
Evidence Table 39. RCTs Comparing Endovascular Revascularization for Chronic CLI–Section 8.2. 143
Evidence Table 40. Nonrandomized Trials, Observational Studies, and/or Registries of Endovascular Revascularization for Chromic CLI–Section 8.2.1 151
Evidence Table 41. RCTs of Surgical Revascularization for Chronic CLI–Section 8.2. 157
Evidence Table 42. Nonrandomized Trials, Observational Studies, and/or Registries for Surgical Revascularization for Chronic CLI–Section 8.2. 162
Evidence Table 43. RCT Comparing Prostanoids for End-Stage Peripheral Artery Disease–Section 8.2.3. 167
Evidence Table 44. Nonrandomized Trials, Observational Studies, and/or Registries for Would Healing Therapies for CLI–Section 8.2.3 167
Evidence Table 45. Nonrandomized Trials, Observational Studies, and/or Registries of Acute Limb Ischemia–Section 9.1 169
Evidence Table 46. Nonrandomized Trials, Observational studies, and/or Registries Comparing Evaluating Noninvasive Testing and Angiography for ALI–Section 9.1. 171
Evidence Table 47. RCTs of Revascularization Strategy for ALI–Section 9.2.2. 172
Evidence Table 48. Nonrandomized Trials, Observational Studies, and/or Registries of Clinical Presentation of ALI–Section 9.2.2. 175
Evidence Table 49. Nonrandomized Trials, Observational Studies, and/or Registries of Diagnostic Evaluation of the Cause of ALI–Section 9.2.2 177
Evidence Table 50. Nonrandomized Trials, Observational Studies, and/or Registries of Revascularization Strategy for ALI–Section 9.2.2 178
Evidence Table 51. RCTs for Longitudinal Follow-Up–Section 10. 181
Evidence Table 52. Nonrandomized Trials, Observational Studies, and/or Registries for Longitudinal Follow-Up–Section 10. 182
References .... **Error! Bookmark not defined.**

Methodology and Evidence Review

The recommendations listed in this guideline are, whenever possible, evidence based. An initial extensive evidence review, which included literature derived from research involving human subjects, published in English, and indexed in MEDLINE (through PubMed), EMBASE, the Cochrane Library, the Agency for Healthcare Research and Quality, and other selected databases relevant to this guideline, was conducted from January through September 2015. Key search words included but were not limited to the following: *acute limb ischemia, angioplasty, ankle-brachial index, anticoagulation, antiplatelet therapy, atypical leg symptoms, blood pressure lowering/hypertension, bypass graft/bypass grafting/surgical bypass, cilostazol, claudication/intermittent claudication, critical limb ischemia/severe limb ischemia, diabetes, diagnostic testing, endovascular therapy, exercise rehabilitation/exercise therapy/exercise training/supervised exercise, lower extremity/foot wound/ulcer, peripheral artery disease/peripheral arterial disease/peripheral vascular disease/lower extremity arterial disease, smoking/smoking cessation, statin, stenting,* and *vascular surgery*. Additional relevant studies published through September 2016, during the guideline writing process, were also considered by the writing committee, and added to the evidence tables when appropriate.

© American Heart Association, Inc. and American College of Cardiology Foundation

Evidence Table 1. Nonrandomized Trials, Observational Studies, and/or Registries of History for Clinical Assessment for PAD–Section 2.1.

Study Acronym; Author; Year Published	Study Type/Design; Study Size	Patient Population	Primary Endpoint and Results (include P value; OR or RR; & 95% CI)	Summary/Conclusion Comment(s)
Rose GA 1962(1) 13974778	**Study type:** Cross-sectional study pts with and without claudication given claudication questionnaire; validated to clinical Dx of IC. Study also validated a questionnaire for angina pectoris.<br><br>**Size:** n=37 pts with "undoubted" IC; n=18 controls; total n=55 pts<br><br>**Questionnaire**: IC defined as leg pain that met all of the following elements:<br>• Site must include 1 or both calves<br>• Must be provoked by either hurrying or walking up hill (or by walking on level for those who never walk uphill)<br>• Must never start at rest<br>• Must make the pt stop or slacken pace<br>• Must disappear on a majority of occasions in ≤10 min<br>• Must never disappear while walking continues	**Inclusion criteria:**<br>• "Most" IC/PAD pts had angiograms; non-PAD pts had other causes of leg pain;<br>• IC group mean age 57.1 y; other leg pain group mean age 48.2 y.<br><br>**Exclusion criteria:** N/A	**Results:**<br>• 34/37 claudicants met criteria for IC by questionnaire (92% sensitive)<br>• Of 18 other leg pain controls none met criteria for IC by questionnaire (100% specific)	• Put forth a concept of classic IC<br>• Very small sample size for validation of questionnaire. Highly restrictive definition of IC (will exclude pts with atypical leg symptoms).<br>• High specificity for IC/PAD.<br>• Later studies reported much lower sensitivity of this questionnaire (68%), specificity (100%)<br>*Richard JL, Ducimetiere P, Elgrishi I, et al. Rev Epidemiol Med Sci Sante Publ 1972 (French)*
Leng GC, Fowkes FG 1992(2) 1474406	**Study type:** Cross-sectional study of questionnaire vs. MD clinical assessment/ABI±exercise. Study developed modification of Rose/WHO Questionnaire (phase I/development) and validated the subsequent Edinburgh Claudication Questionnaire (phase II/validation).<br><br>**Size:** Phase I (development) n=647; 586 with claudication/PAD and 61 with other leg pain. Phase II (validation)	**Inclusion criteria:**<br>• Pts with leg symptoms seen in Vascular Clinic who had undergone ABI (Phase I/development).<br>• Vascular clinic pts with leg pain and community pts seeing a GP (Phase II/validation).<br><br>**Exclusion criteria:** N/A	**Results:**<br>• Performance of WHO/Rose in the dataset—Sensitivity 60%; specificity of 91%<br>• Does the pain every disappear while still walking, poorest performing element of WHO/Rose<br>• Edinburgh Claudication Questionnaire performance vs. ABI/clinical assessment by clinician:<br>• Sensitivity: 91.3% community, 82.8% vascular clinic<br>• Specificity: 99.3% community, 100%	• Identified key issues with WHO/Rose Questionnaire to develop Edinburgh Claudication Questionnaire. Maintained 5 questions from WHO/Rose (or with minor modification), removed 2 questions, diagram included for pts to localize site of pain (front and back of both legs)

© American Heart Association, Inc. and American College of Cardiology Foundation

	n=350; 50 vascular clinic pts and 300 community pts—also did a reproducibility study		vascular clinic<br>• PPV: 91% community, 100% vascular clinic<br>• NPV: 99% community, 81% vascular clinic	
Criqui MH, et al.<br>1996(3)<br>9546918	**Study type:** Cross-sectional study of modified WHO/ROSE questionnaire (San Diego Claudication Questionnaire) vs. ABI/TBI/posterior tibial flow velocity<br><br>**Size:** n=508 pts (980 limbs for analysis)	**Inclusion criteria:**<br>• Pts seen during preceding 10 y at San Diego VA Hospital or UCSD Medical Center vascular labs invited to participate<br>• Mean age 68 y<br>• Vascular lab studies used to characterize pts as:<br>Optimal (no disease)<br>Borderline Normal<br>Isolated small vessel<br>Isolated posterior tibial<br>Moderate PAD (ABI 0.61–0.9)<br>Severe PAD (ABI <0.6)<br><br>**Exclusion criteria:** N/A	**Results:** Questionnaire identified wide spectrum of clinical sx in pts with documented PAD, including no sx, pain at rest, noncalf pain, nonRose calf claudication, Rose calf claudication	• San Diego Claudication Questionnaire accounts for right and left leg symptoms separately (as well as both legs) and included buttock and thigh pain.<br>• Questionnaire allows for more variation of sx and pts leg symptoms can be categorized as: No pain, pain at rest, non-calf, non-Rose calf and Rose (calf).<br>• Study recognized wider spectrum of leg sx in PAD including leg sx not c/w WHO/Rose and also non-calf symptoms—early concept of "atypical" leg sx in PAD
McDermott MM, et al.<br>1999(4)<br>10030313	**Study type:** Cross-sectional study of pts with and without PAD administered San Diego Claudication questionnaire, ABI assessment<br><br>**Size:** n=268 pts (137 known PAD from vascular lab; 26 known PAD from general medical practice; 105 pts without PAD)	**Inclusion criteria:**<br>• Pts with and without PAD identified from (vascular. lab, general medical clinics)<br>• PAD defined as ABI <0.9<br><br>**Exclusion criteria:** Low MMSE, nursing home residents, wheel-chair bound, pts with major lower extremity amputation, non-English speakers, life expectancy <6 mo, noncompressible ABI >1.50	**Results:**<br>• Grouped pts according to 4 categories based on San Diego Claudication Questionnaire:<br>1. No exertional leg symptoms<br>2. IC (classic)<br>3. Atypical exertional leg symptoms<br>4. Pain at rest<br>• Among N=137 PAD pts identified from vascular lab:<br>15.3% had no exertional leg symptoms;<br>28.5% had IC (classic);<br>25.5% atypical exertional leg symptoms;<br>30.7% pain at rest.<br>• Among PAD pts (n=163), factors significantly associated absence of exertional leg sx: older age, male sex, DM, PAD pt recruited from general medicine clinic rather than vascular lab<br>• Among PAD pts (N=163). factors	• Further validated wider spectrum of lower extremity sx among pts with confirmed PAD

© American Heart Association, Inc. and American College of Cardiology Foundation

			significantly associated with classical IC lower ABI, PAD recruited from vascular lab rather than general medicine clinic	
McDermott MM, et al. 2001(5) 11585483	**Study type**: Cross-sectional study of pts with and without PAD identified from 3 medical centers in same city. Pts underwent functional capacity assessments (6min walk, 4 M walk, chair raises), assessment of physical activity, ABI, questionnaires<br><br>**Size:** n=590 pts (460 with PAD; 130 without PAD)	**Inclusion criteria:**<br>• Pts with and without PAD identified from 3 medical centers (vascular lab, general medical practice)<br>• PAD confirmed with study ABI (average leg pressure method) and required ABI <0.9<br><br>**Exclusion criteria:**<br>• "PAD" pts with normal ABI at study visit<br>• Dementia<br>• Nursing home residents<br>• Wheelchair bound<br>• Pts with major lower extremity amputation<br>• Recent major surgery<br>• Non-English speakers	**Results:**<br>Grouped pts according to 6 types of leg symptoms in 4 overall categories:<br>1. IC (classic)<br>2. Atypical exertional leg pain (carry on/stop)<br>3. No exertional leg pain (active/inactive walk >6 blocks/wk Yes/No)<br>4. Leg pain on exertion and at rest<br>• Among confirmed PAD pts:<br>32% had IC;<br>19% leg pain on exertion and at rest;<br>29% atypical exertional leg pain (9% carry on; 20% stop);<br>20% no exertional leg pain.<br>• PAD pts in the non-IC groups also demonstrated functional impairment in terms of 6 min walk, 4 meter walk.<br>• No exertional leg pain/inactive and exertional and rest pain groups with worse functional capacity than IC group.<br>• Atypical exertional leg pain/carry on group with better outcomes on 6 min walk than IC group.	• More data on wide spectrum of leg sx among pts with PAD and demonstration that functional impairment is common regardless of type of leg symptoms.
Hirsch AT, et al. 2001(6) 11560536	**Study type**: Multi-center cross-sectional study conducted at 350 primary care practices in the US.<br><br>Pts enrolled underwent San Diego Claudication Questionnaire, medical and CV Hx/risk factor assessment, BP, anthropomorphics, and ABI assessment.<br><br>Pts. identified as having PAD (and their providers) further asked about awareness of the PAD Dx.	**Inclusion criteria:**<br>• Age ≥70 y; Age 50–69 y with DM or at least 10 pack-year tobacco Hx<br>• PAD (lower leg pressure method) defined as ABI ≤0.9 in either leg<br><br>**Exclusion criteria**: N/A	**Results:**<br>• Prevalence of PAD in this cohort was 29%<br>• Among 1865 pts with PAD (mean ABI 0.78):<br>5.5%–15.3% Rose claudication;<br>46.3%–61.7% atypical leg sx;<br>23.3%–48.3% no pain;<br>**rates reported for new Dx/prior Dx and for PAD only and PAD+CVD	• More data on wide spectrum of leg sx among pts with PAD; only approximately 5%–15% of ABI confirmed PAD pts have classic Rose claudication. Majority have atypical non-Rose leg sx or no leg pain.

© American Heart Association, Inc. and American College of Cardiology Foundation

	**Size:** n=6,979 (1865 had PAD)			
Khan NA, et al. 2006(7) 16449619	**Study type:** Systematic review of studies that evaluated element of Hx and/or physical examination for Dx of PAD in pts with and without disease<br><br>**Size:** Total of 6,272 pts in 11 diagnostic accuracy studies	**Inclusion criteria:**<br>• Studies published from 1/1966–3/2005<br>• 51 potential articles identified from MEDLINE and Cochrane databases<br>• Exam maneuvers had to be described clearly<br>• PAD Dx confirmed by reference standard: ABI, duplex, or angiogram<br>• Data could be extracted into a 2 x 2 table<br>• 17 studies met inclusion criteria (11 on diagnostic accuracy)<br><br>**Exclusion criteria:** N/A	**Results:**<br>***Hx – Symptoms of claudication***<br>• Presence of claudication ↑ likelihood PAD (LR PAD: 3.30; 95% CI: 2.30–4.80)<br>• Absence of claudication did not lower likelihood of any PAD, but lowered likelihood of moderate to severe PAD (ABI <0.70) (LR: 0.57; 95% CI: 0.43–0.76))	• Presence of claudication increases likelihood of PAD. Absence of claudication does not lower likelihood of PAD, but lowers likelihood of moderate to severe PAD.
Grøndal N, et al. 2015(8) 25923784	**Study type:** Danish intervention arm of screening trial<br><br>**Size:** n=25,083 men who were screened for AAA. 18,749 attended the screening (uptake 74.7%).	**Inclusion criteria:** Men age 65–74 y who were screened for AAA.<br><br>**Exclusion criteria:** N/A	**1° endpoint:** Prevalence of PAD in pts screened for AAA.<br><br>**Results:** AAA was diagnosed in 3.3% and PAD in 10.9%.	• The prevalence of AAA in Denmark has declined in the past decade from 4.0% to 3.3%.<br>• 10.9% of men undergoing screening for AAA also had PAD.
Wassel et al. 2011(9) 21920269	**Study type:** Observational population-based study of current or former employees of the University of California, San Diego, and their significant others, as well as 193 other volunteers and their significant others.<br><br>**Size:** n=2,404 pts	**Inclusion criteria:** Men and women age 19–91 y who completed the baseline visit in the San Diego Population Study<br><br>**Exclusion criteria:** N/A	**1° endpoint:** Prevalence of PAD in the study population<br><br>**Results:**<br>• Family hx of PAD was significant, when adjusting for SBP, DBP, and dyslipidemia (OR: 1.83; 95% CI: 1.03–3.26; p=0.04)<br>• Family hx of PAD was strongly associated with severe prevalent PAD (OR: 2.42; 95% CI: 1.13–5.23; p=0.02).<br>• Parental hx of PAD was significant when adjusting for SBP, DBP, and dyslipidemia (OR: 1.83; 95% CI: 1.00–3.41; p=0.05)<br>• Parental hx of PAD was strongly associated with severe prevalent PAD (OR: 2.91; 95%CI: 1.33–6.40; p=0.008).	N/A

© American Heart Association, Inc. and American College of Cardiology Foundation

Clark CE et al., 2012(10) 22293369	**Study type:** Meta-analysis<br><br>**Size:** n=20 studies	**Inclusion criteria:**<br>• Cohort or cross-sectional studies of differences in BP between arms<br>• Age ≥18 y<br>• Data for central vascular disease, PVD, or death<br><br>**Exclusion criteria:**<br>• Case reports	**1° endpoint:** PVD<br><br>**Results:**<br>• Significant association of a difference of ≥10 mmHg and SS (risk ratio: 8.8; 95% CI: 3·6–21.2)<br>• Significant association in noninvasive studies of a difference of ≥15 mmHg and PVD (risk ratio: 2.5, 95% CI: 1.6–3.8) (sensitivity: 15%; 95% CI: 9–23) (specificity: 96%; 95% CI: 94–98)<br>• Significant association in noninvasive studies of a difference of ≥15 mmHg and pre-existing cerebrovascular disease (risk ratio: 1.6, 95% CI: 1.1–2.48) (sensitivity: 8%; 95% CI: 2–26) (specificity: 93%; 95% CI: 86–97)<br>• Significant association in noninvasive studies of a difference of ≥15 mmHg and cardiovascular mortality (HR: 1.7, 95% CI: 1.1–2.5)<br>• Significant association in noninvasive studies of a difference of ≥15 mmHg and all-cause mortality (HR: 1.6; 95% CI: 1.1–2.3<br>• Significant association of ≥10 mmHg and PVD (RR: 2.4; 95% CI: 1.5–3.9) (sensitivity: 32%; 95%CI: 23–41) (specificity: 91%, 95% CI: 86–94)	• A difference in SBP of ≥10 mm Hg or of ≥15 mm Hg, between arms might help to identify pts who need further vascular assessment.<br>• A difference of ≥15 mm Hg could be a useful indicator of risk of vascular disease and death.
Singh S et al., 2015(11) 26160261	**Study type:** Meta-analysis of cohort studies<br><br>**Size:** n=18 cohorts	**Inclusion criteria:**<br>• Studies measuring BP simultaneously in arms or legs<br>• Studies reporting CAD, cerebrovascular disease, PAD, subclavian stenosis, survival or mortality, and other relevant CV indices or outcomes.<br><br>**Exclusion criteria:**<br>• Studies that did not report a dichotomous outcome defined by a	**1° endpoint:** Prevelance of PAD, CAD, cerebrovascular disease, subclavian stenosis, all-cause, and CV mortality<br><br>**Results:**<br>• Significant association between IASBPD of ≥10 mmHg and PAD (RR: 2.22; 95% CI: 1.41–3.5; p=0.0006) (sensitivity: 16.6%; 95% CI: 6.7–35.4) (specificity: 91.9%; 95% CI: 83.1–96.3)<br>• Significant association of PAD at cutoff of 15 mmHg (RR: 1.91; 95% CI: 1.28–2.84;	• Inter-arm and leg BP differences are predictors of PAD. The IASBPD may be associated subclavian stenosis, high left ventricular mass effect, and higher brachial–ankle PWVs.

© American Heart Association, Inc. and American College of Cardiology Foundation

		specific BP difference cutoff	p=0.001) (sensitivity: 25.1%; 95% CI 7.9–56.7) (specificity: 88.2%; 95% CI: 71.7–95.7).<br>• Significant association between inter-leg BP difference of ≥15 mmHg and PAD (RR: 11.87; 95% CI: 7.64–18.44).<br>• IASBPD of ≥10 mmHg was not associated with carotid-femoral PWV (standardized mean difference: 0.26; 95% CI: 0.15–0.68; p=0.21). One study demonstrated positive association between IASBPD of ≥10 mmHg and brachial ankle PWV (adjusted OR from multivariate model: 1.001; 95% CI: 1.000–1.001; p=0.022).<br>• Significant association of inter-leg BP difference of ≥15 mm Hg or more and brachial–ankle PWV (standardized mean difference: 0.68; 95% CI: 0.37–0.99; p=0.0001).	
Shadman R et al., 2004(12) 15358030	**Study type**: Review of cohort studies<br><br>**Size**: n=4 cohorts with 4,223 pts (2,975 from 2 free-living cohorts and 1,248 from 2 clinical cohorts)	**Inclusion criteria**:<br>Cohort A:<br>• Geographic defined population study<br>• Part of the Lipid Research Clinics protocol study<br><br>Cohort B:<br>• Randomly selected from a database of UCSD employees and spouses<br><br>Cohort C:<br>• Pt population in Chicago<br><br>Cohort D:<br>• Pts who visited the San Diego Vererans Administration Medical Center or UCSD Medical Center vascular laboratories between 1990–1994	**1° endpoint**: Prevelance of SS<br><br>**Results**:<br>• SS was significantly (p<0.05) associated with past smoking (OR: 1.80), current smoking (OR: 2.61), and higher levels of SBP (OR:1.90 per 20 mm Hg)<br>• Significant association between higher levels of HDL and SS (OR: 0.87 per 10 mg/dl)<br>• Significant association of SS and PAD (OR: 5.11, p<0.001)	•SS is correlated with current and past smoking histories, SBP, HDL levels (inversely), and the presence of PAD<br>• bilateral brachial BP measurements should routinely be performed in pts with an elevated risk profile, both to screen for SS, and to avoid missing a hypertension or PAD diagnosis because of unilateral pressure measurement in an obstructed arm

© American Heart Association, Inc. and American College of Cardiology Foundation

		**Exclusion criteria:**<br>Cohort A: Missing data<br><br>Cohort B: N/A<br><br>Cohort C:<br>• Wheelchair bound<br>• Hx Foot or leg amputations<br>• Nursing home residents<br>• Non-English speaking<br>• Hx dementia<br><br>Cohort D: N/A		

ABI indicates ankle-brachial index; BP, blood pressure; CI, confidence interval; CV, cardiovascularular; DBP, diastolic blood pressure; GP, general practitioner; HR, hazard ratio; IASBPD, inter-arm systolic blood pressure; IC, intermittent claudication; LR, likelihood ratio; MMSE, Mini-Mental State Examination; N/A, not applicable; NPV, negative predictive value; OR, odds ratio; PAD, peripheral artery disease; PPV, positive predictive value; pt, patient; PVD, peripheral vascular disease; PWV, pulse wave velocity; RR, relative risk; SBP, systolic blood pressure; SS, subclavian artery stenosis; TBI, toe-brachial index; UCSD, University of California, San Diego; VA, veterans affairs; and WHO, World Health Organization.

Evidence Table 2. Nonrandomized Trials, Observational Studies, and/or Registries of Physical Examination for Clinical Assessment for PAD–Section 2.1.

Study Acronym; Author; Year Published	Study Type/Design; Study Size	Patient Population	Primary Endpoint and Results (include P value; OR or RR; & 95% CI)	Summary/Conclusion Comment(s)
Khan NA et al.<br>2006(7)<br>16449619	**Study type:**<br>Systematic review of studies that evaluated element of Hx and/or physical examination for Dx of PAD in pts with and without disease<br><br>**Study size:** n=6,272 pts in 11 diagnostic accuracy studies	**Inclusion criteria:**<br>• Studies published from 1/1966–3/2005<br>• 51 potential articles identified from MEDLINE and Cochrane databases<br>• Exam maneuvers had to be described clearly<br>• PAD Dx confirmed by reference standard: ABI, duplex, or angiogram<br>• Data could be extracted into a 2 x 2 table<br>• 17 studies met inclusion criteria	**Results:**<br>***Physical Examination***<br>**Skin changes**<br>Skin cool to touch in affected leg:<br>• LR PAD: 5.90; 95% CI 4.10–8.60<br>Leg wound/sore:<br>• LR PAD: 5.90; 95% CI: 2.60–13.40<br>Discolored skin:<br>• LR PAD: 2.80; 95% CI: 2.40–3.30<br>Absence of cool skin, wound/sore did not lower likelihood of PAD<br>**Bruits**<br>Presence of ≥1 bruit	• In general, presence of physical findings increases likelihood of PAD<br>• Entirely normal pulse exam and absence of any bruits decrease likelihood of PAD<br>• Sensitivities/specificities not reported in this review

© American Heart Association, Inc. and American College of Cardiology Foundation

		(11 on diagnostic accuracy)<br><br>**Exclusion criteria:** N/A	• LR PAD: 5.60; 95% CI: 4.70–6.70<br>Over iliac, femoral, popliteal artery<br>Absence of a bruit over all 3 arteries<br>• LR PAD: 0.39; 95% CI: 0.34–0.45<br>**Pulse Palpation**<br>Any* pulse abnormality<br>• LR PAD: 4.70; 95% CI: 2.20–9.90<br>Absent/reduced<br>*any=femoral/popliteal/DP/PT<br>Absence of any pulse abnormality:<br>• LR PAD: 0.38; 95% CI: 0.23–0.64<br>Abnormal dorsalis pedis pulse less diagnostically useful than abnormal femoral or PT pulse<br>• DP not palpable in 8.1% of healthy pts<br>• PT not palpable in 2.9% of healthy pts<br><br>**Capillary Refill**<br>Abnormal capillary refill time<br>LR PAD: 1.90; 95% CI: 1.20–3.20<br>Prolonged venous refill<br>LR mod/sev PAD: 3.60; 95% CI: 1.90–6.80<br><br>Normal venous refill time not informative to r/o PAD	
Cournot M et al.<br>2007(13)<br>18154997	**Study type:**<br>• Part of the EVADEC, prospective cohort study (cross-sectional analysis). Pts with no known vascularular disease underwent physical examination followed by vascularular studies (carotid, femoral ultrasound, ABI)<br>• Physical examination included pulse assessment (present/absent), bruit assessment using the	**Inclusion criteria:**<br>• 18–90 y (mean age 52 y)<br>• No known CVD<br>• Asx<br><br>**Exclusion criteria:** CV disease identified by medical record review	**Results**<br>14.5% of pts had any bruit or absent PT/DP pulse<br>*Femoral bruit*<br>• +LR ipsilateral ABI <0.9: 2.90; 95% CI: 1.63–5.16<br>• -LR ipsialteral ABI <0.9: 0.93; 95% CI: 0.88–0.98<br>*Absent PT pulse*<br>• +LR ipsilateral ABI <0.9: 1.80; 95% CI: 1.08–3.01<br>• -LR ipsilateral ABI <0.9: 0.94; 95% CI: 0.88–1.01<br>*Absent DP pulse*<br>• +LR ipsilateral ABI <0.9: 2.01; 95% CI: 1.17–3.45<br>• -LR ipsilateral ABI <0.9: 0.94; 95% CI: 0.88–1.00<br>*Absent DP+PT*<br>• +LR ipsilateral ABI <0.9: 3.57; 95% CI: 1.93–6.60<br>• -LR ipsilateral ABI <0.9: 0.93; 95% CI: 0.97–1.00<br>Interaction term for DM not significant<br>Interobserver agreement 97% for femoral bruit; 92% PT palpation; 92% DP palpation	Both presence of femoral bruit and absent pulses increase likelihood of PAD in asx pts without known PAD/CVD

© American Heart Association, Inc. and American College of Cardiology Foundation

	bell of stethoscope<br><br>**Size:** n=2,736 eligible pts<br><br>**Interobserver variability substudy size:** 500 pts		Also reported on carotid bruit for Dx of carotid stenosis/plaque/increased IMT (did not affect LR)	
Armstrong DW et al. 2010(14) 21165366	**Study type:** Retrospective database analysis of pts who underwent ABI and had a physical examination documented in the CARDIOfile database between 12.2005–2.2010 at a single clinic<br><br>**Size:** n=1,236 eligible pts with complete data	**Inclusion criteria:** Pts who had ABI performed for suspected PAD or risk factors for PAD (Age >70 y, DM or smokers ages 50–69 y, intermediate Framingham Risk score)<br><br>**Exclusion criteria:** Pts with ABI >1.30 in either leg; incomplete physical examination in the databse<br><br>**Definitions**<br>• PAD defined as ABI ≤0.9<br>• Pulses rated 0-3 scale; analysis absent vs. present<br>• Femoral bruits present/absent<br>• Claudication=leg sx with exercise gone within 5 min of rest.	**Results:**<br>28.1% of pts had an abnormal ABI in at least 1 leg (PAD)<br>*Femoral bruit*<br>• Sens 36.1%, Spec 92.0%<br>• PPV 51.1%, NPV 86.2%, Accuracy 81.6%<br>• +LR PAD 4.5<br>• -LR PAD 0.69<br>*PT pulse abnl*<br>• Sens 70.0%, Spec 83.4%<br>• PPV 49.3%, NPV 92.3%, Accuracy 80.9%<br>• +LR PAD 4.2<br>• -LR PAD 0.36<br>*DP pulse abnl*<br>• Sens 63.9%, Spec 80.6%<br>• PPV 43.2%, NPV 90.7%, Accuracy 77.5%<br>• +LR PAD 3.3<br>• -LR PAD 0.45<br>*Absent DP and PT pulses+femoral bruit either side (vs. normal pulses, no femoral bruits)*<br>• Sens 58.2%, Spec 98.3%<br>• PPV 81%, NPV 94.9%, Accuracy 93.8%<br>• +LR PAD 34.2<br>• -LR PAD 0.43	• Completely normal exam (all ankle pulses present and no femoral bruits) has high accuracy for normal ABI/no PAD.<br>• Pulse abnormalities+femoral bruits makes Dx of PAD likely.<br>• Single abnormal physical findings increased likelihood of abnormal ABI (specific findings)<br>• Sensitivity of single abnormal physical examination findings lower; not as "reassuring" to rule out PAD/abnormal ABI

ABI indicates ankle-brachial index; CI indicates confidence interval; CVD, cardiovascularular disease; CV, cardiovascularular; DP, dorsalis pedis; Hx, history; IMT, intima-media thickness; LR, likelihood ratio; PPV, positive predictive value; PAD, peripheral artery disease; PT, posterior tibial; pt, patient; OR, odds ratio; RR, relative risk; sens, sensitivity; and spec, specificity.

© American Heart Association, Inc. and American College of Cardiology Foundation

Evidence Table 3. RCTs of Resting ABI for Diagnosing PAD–Section 3.1.

Study Acronym; Author; Year Published	Aim of Study; Study Type; Study Size (N)	Patient Population	Study Intervention (# patients) / Study Comparator (# patients)	Endpoint Results (Absolute Event Rates, P value; OR or RR; & 95% CI)	Relevant 2° Endpoint (if any); Study Limitations; Adverse Events
Fowkes FG et al. 2010(15)<br>20197530	**Aim:** To determine the effectiveness of ASA in preventing events in people with a low ABI identified on screening the general population<br><br>**Study type:** RCT<br><br>**Size:** n=3,350 pts	**Inclusion criteria:** Men and women age 50–75 y<br><br>**Exclusion criteria:**<br>• Previous Hx of vascular disease, MI, or stroke;<br>• Currently taking ASA or warfarin.	**Intervention:** 100 mg enteric coated ASA<br><br>**Comparator:** Placebo	**1° endpoint:** Composite of initial fatal or nonfatal coronary event, stroke or revascularization. (ASA: 13.7; 95% CI: 11.8–15.9 vs. placebo: 13.3; 95% CI: 11.4–15.4, events per 1,000 person-y; HR: 1.03; 95% CI: 0.84–1.27<br><br>**1° Safety endpoint:**<br>Major Hemorrhage: ASA: 2.5; 95% CI: 1.7–3.5 vs. placebo: 1.5; 95% CI: 0.9–2.3 per 1,000 person-y; HR: 1.71; 95% CI: 0.99–2.97	• Initial vascular events defined as a composite of a 1° endpoint event or angina, IC, orTIA.<br>ASA: 22.8; 95% CI: 20.2–25.6 vs. placebo: 22.9; 95% CI: 20.3–25.7 events per 1,000 person-y;<br>HR: 1.00; 95% CI: 0.85–1.17<br>• All-cause mortality<br>ASA group, 176 deaths (12.8; 95% CI: 11.0–14.8 per 1,000 person-y); placebo group, 186 deaths (13.5; 95% CI: 11.6–15.6 per 1,000 person-y; HR: 0.95; 95% CI: 0.77–1.16)<br>• Limitations: higher proportion of women, inclusion of pts with DM could have influenced results
**POPADAD**<br>Belch J et al. 2008(16)<br>18927173	**Aim**: To determine whether ASA and antioxidant therapy, combined or alone, are more effective than placebo in reducing CVD events in pts with DM and Asx PAD.<br><br>**Study type:** Multicenter, randomized, double blind, 2×2 factorial, placebo controlled trial.<br><br>**Size:** n=1,276 pts	**Inclusion criteria**: Age ≥40 y with type 1 or type 2 DM and ABI of ≤0.99 but no Sx CVD.<br><br>**Exclusion criteria**: People with: evidence of Sx vascular CVD; ASA or antioxidant therapy use on a regular basis; peptic ulceration, severe dyspepsia, a bleeding disorder, or intolerance to ASA; suspected serious physical illness (e.g., cancer), which could curtail life expectancy; psychiatric illness (reported by GP); pts with congenital heart disease; and pts unable to give informed consent	**Intervention and comparator**: Daily, 100 mg ASA tablet + antioxidant capsule (n=320); ASA + placebo capsule (n=318); placebo tablet + antioxidant capsule (n=320); or placebo tablet + placebo capsule (n=318).	**1° endpoint**:<br>• Death from CHD or stroke, nonfatal MI or stroke, or amputation above the ankle for CLI; and death from CHD or stroke<br>• 116 of 638 1° events in the ASA groups compared with 117 of 638 in the no ASA groups (18.2% vs. 18.3%) HR: 0.98; 95% CI: 0.76–1.26. 43 deaths from CHD or stroke occurred in the ASA groups compared with 35 in the no ASA groups (6.7% vs. 5.5%): HR: 1.23; 95% CI: 0.79–1.93).<br>• No difference in treatment for ABI <0.90	**Adverse effect (effect estimates):**<br>• Malignancy 0.76 (0.52–1.11),<br>• GI bleeding, 0.90 (0.53–1.52)<br>• Dyspepsia 0.77 (0.55–1.08),<br>• Allergy 1.14 (0.80–1.63)

© American Heart Association, Inc. and American College of Cardiology Foundation

McDermott, MM et al. 2013(17) 23821089	**Study type**: RCT testing efficacy of a home-based walking exercise intervention vs. control in pts with PAD with and without claudication<br><br>**Size**: n=194 pts; 72.2% without claudication	**Inclusion criteria**:<br>• Age ≥65 y<br>• ABI ≤0.9 or 20% post exercise drop in ABI<br><br>**Exclusion criteria**:<br>• Lower extremity amputation<br>• Inability to walk ≥50 ft without stopping<br>• Inability to attend weekly sessions<br>• Walking impairment not from PAD<br>• CLI	**Intervention**: Home-based group-mediated cognitive behavioral walking group<br><br>**Comparator**: Health education	**1° endpoint**: Change in 6-MWT between baseline and 6 mo<br><br>**Secondary outcomes**: Change in treadmill MWT; PFWT; physical activity; WIQ scores; PCS and MCS of SF-36<br><br>**Results**:<br>6-MWT:<br>• Control: 347 m BL vs. 329 m 6mo<br>• Intervention: 372 m BL vs. 386 m 6 mo	• Modest improvement in 6-MWT distance after 6 mo of home-based exercise in pts with Asx PAD

1° indicates primary; ABI, ankle-brachial index; ASA, aspirin; Asx, asymptomatic; CI, confidence interval; BL, baseline; CVD, cardiovascular disease; CHD, coronary heart disease; GI, gastrointestinal; HR, hazard ratio, Hx, history; IC, intermittent claudication; MCS, mental component summary score; MWT, mean walking time; PAD, peripheral artery disease; PCS, physical component summary score; PFWT, pain-free walking time; pt, patient; Sx, symptomatic; RCT, randomized controlled trial; and TIA, transient ischemic attack

Evidence Table 4. Nonrandomized Trials, Observational Studies, and/or Registries of Resting ABI for Diagnosing PAD–Section 3.1.

Study Acronym; Author; Year Published	Study Type/Design; Study Size	Patient Population	Primary Endpoint and Results (include P value; OR or RR; & 95% CI)	Summary/Conclusion Comment(s)
Criqui MH, et al. 2005(18) 16246968	**Study type**: Cross-sectional study<br><br>**Size**: 2,343 pts	**Inclusion criteria**:<br>• Age 29–91 y<br>• 1 of the following ethnicities: Non-Hispanic Whites, blacks, Hispanics, Asian<br><br>**Exclusion criteria**: N/A	**1° endpoint**: PAD prevalence<br><br>**Results**:<br>• 104 PAD cases (4.4%)<br>• Blacks had a higher PAD prevalence than Non-Hispanic Whites (OR: 2.30; p>0.024)<br>• Hispanics and Asians has a lower but nonsignificant lower PAD prevalence than Whites	• Suggests black ethnicity is a risk factor for PAD<br>• No evidence of blacks being of higher susceptibility to CV risk factors to explain increased risk for PAD<br>• Low prevalence of PAD (4.4%)
Selvin E, et al. 2004(19) 15262830	**Study type**: Cross-sectional survey<br><br>**Size**: n=2,174 pts	**Inclusion criteria**:<br>• Age ≥40 y<br>• Participants of 1999–2000 NHANES<br>• Participants with valid mean ABI blood pressure index	**1° endpoint**: Frequency of detection, pt and physician awareness of diagnosis, and treatment intensity<br><br>**Results**:<br>• Prevalence of PAD in adults ≥40 y in U.S. was 4.3% (95% CI: 3.1%–5.5%)<br>• Prevalence of PAD in adults ≥70 y in U.S. was 14.5% (95% CI: 10.8%–18.2%)	• PAD defined as ABI <0.90 in either leg<br>• In the U.S., PAD affects >5 million adults.<br>• PAD prevalence increases with age and disproportionately affects blacks.<br>• Majority of pt with PAD have ≥1

© American Heart Association, Inc. and American College of Cardiology Foundation

		**Exclusion criteria**:<br>• ABI values >1.5<br>• Participants with missing variables of interest	• Black race/ethnicity (OR: 2.83; 95% CI: 1.48–5.42); current smoking (OR: 4.46; 95% CI: 2.25–8.84), DM (OR: 2.27; 95% CI: 1.03–7.12), hypertension (OR: 1.74; 95% CI: 0.97–3.13), hypercholesterdemia (OR: 1.68; 95% CI: 1.09–2.57) and low kidney function (OR: 2.00; 95% CI: 1.08–3.70) were positively associated with PAD prevalence.	CVD risk factor.<br>• Low Prevalence of PAD: 4.3%; 95% CI: 3.1%–5.5%
Hirsch AT, et al. 2001(6)<br>11560536	**Study type**:<br>• Multi-center cross-sectional study conducted at 350 primary care practices in the US.<br>• Pts enrolled underwent San Diego Claudication Questionnaire, medical and CV Hx/risk factor assessment, BP, anthropomorphics, and ABI assessment.<br>• Pts. identified as having PAD (and their providers) further asked about awareness of the PAD Dx.<br><br>**Size**: n=6,979 pts (1,865 had PAD)	**Inclusion criteria**:<br>• Age ≥70 y or age 50–69 y with DM or Hx of ≥10 pack-year tobacco<br>• PAD (lower leg pressure method) defined as ABI ≤0.9 in either leg<br><br>**Exclusion criteria**: N/A	**Results:**<br>• Prevalence of PAD in this cohort was 29%<br>• Among 1,865 pts with PAD (mean ABI 0.78):<br>5.5–15.3% Rose claudication;<br>46.3–61.7% atypical leg sx;<br>23.3–48.3% no pain<br>**Rates reported for new Dx/prior Dx and for PAD only and PAD+CVD	• More data on wide spectrum of leg sx among pts with PAD; only about 5-15% of ABI confirmed PAD pts have classic Rose claudication. Many majority have atypical non-Rose leg sx or no leg pain.
Guo X, et al. 2008(20)<br>18362433	**Study type**:<br>Observational test comparison<br><br>**Size**: n=298 pts	**Inclusion criteria**:<br>• Age ≥35 y<br>• Cardiology clinic: referrals for DSA & ABI<br><br>**Exclusion criteria**: Severe DM & hypertension<br><br>**Gold standard**:<br>• DSA.<br>• Stenosis ≥50%<br><br>**ABI method**: Oscillometry	**1° endpoint**: Presence of stenosis below aorto-iliac bifurcation in leg with lower ABI<br><br>**Results**:<br>• Sensitivity: 76 (N/A)<br>• Specificity: 90 (N/A)<br>• PPV: 36 (N/A)<br>• NPV: 98 (N/A)	• Moderate sensitivity and good specificity. No indication of % with PAD symptoms but low prevalence of PAD on DSA (7%) suggests it was negligible.<br>• 53% had coronary heart disease and 13% stroke.
Aboyans V, et al.	**Study type**: Scientific	**Inclusion criteria**: N/A	**1° endpoint**: N/A	• AHA Scientific Statement on the

© American Heart Association, Inc. and American College of Cardiology Foundation

2012(21)<br>23159553	statement<br><br>**Size**: N/A	**Exclusion criteria**: N/A	**Results**: N/A	measurement and interpretation of the ABI
Aboyans V, et al. 2008(22)<br>18692981	**Study type**: Cross-sectional<br><br>**Size**: n=510 pts	**Inclusion criteria**: ambulatory pts presenting to vascular lab<br><br>**Exclusion criteria**: N/A	**1° endpoint**: Association of risk factors with ABI >1.4 and ABI <0.9 and disease presence by TBI<br><br>**Results**: In 84.2% of cases, diabetic limbs with ABI ≥1.40 had abnormal results in at least 1 of the 2 noninvasive vascular indicators, suggestive of concomitant occlusive disease.	• 50% with DM<br>• No angiographic correlations
Schröder F, et al. 2006(23)<br>16950430	**Study type**: Observational test comparison<br><br>**Size**: n=216 pts	**Inclusion criteria**: Attending a vascular medicine clinic “suspected of having a vascular disease. Age >40 y<br><br>**Exclusion criteria**: Previous evidence of PAD, obesity, atrial fibrillation, ABI >1.3<br><br>**Gold standard**: Duplex ultrasound	**1° endpoint**: Stenosis >70%<br><br>**Results**: High;Low of post/ant tibial arteries<br>• Sensitivity: 0.68;0.89<br>• Specificity: 0.99;0.93<br>• PPV 0.99;0.93<br>• NPV: 0.74;0.88	ABI had good sensitivity and very high specificity and PPV. Using lower ankle pressure improved sensitivity.
Premalatha G, et al.<br>2002(24)<br>12568206	**Study type**: Observational test comparison<br><br>**Size**: n=100 pts	**Inclusion criteria**: Pts with DM with foot lesions<br><br>**Exclusion criteria**: Calcification of peripheral arteries<br><br>**Gold standard**: Duplex ultrasound	**1° endpoint**: Precise criteria for PAD not stated.<br><br>**Results:**<br>• Sensitivity: 0.71<br>• Specificity: 0.89	Study in pts with DM with clinical suggestion of PAD showing good sensitivity and high specificity.
Allen J, et al. 1996(25)<br>8638864	**Study type**: Observational test comparison<br><br>**Size**: n=200 pts	**Inclusion criteria**: Consecutive referrals to a vascular laboratory.<br><br>**Exclusion criteria**: Previous vascular surgery. DM<br><br>**Gold standard**: Duplex	**1° endpoint**: Stenosis >50%<br><br>**Results**:<br>• Sensitivity: 0.82<br>• Specificity: 0.84<br>• PPV: 1.0<br>• NPV: 0.83	• Pt symptoms not presented in detail but it would appear that most were sx pts referred for investigation.<br>• ABI had good sensitivity and specificity and excellent PPV.

© American Heart Association, Inc. and American College of Cardiology Foundation

		ultrasound		
Lijmer JG, et al. 1996(26) 8795165	**Study type:** Observational test comparison<br><br>**Size:** n=53 pts	**Inclusion criteria:** Claudication symptoms or signs of CLI in referrals to vascular laboratory<br><br>**Exclusion criteria:** N/A<br><br>**Gold standard:** Digital subtraction angiography	**1° endpoint:** Stenosis >50%<br><br>**Results:**<br>• Sensitivity: 0.84<br>• Specificity: 0.88	• Small study but merits include some correction for "verification bias" in selection of pts having angiography and thus included in the study.<br>• ABI had good sensitivity and specificity.
Ankle Brachial Index Collaboration 2008(27) 18612117	**Study type:** Meta-Analysis<br><br>**Size:** n=16 population cohort studies, n=57,294 pts	**Inclusion criteria:** Availability of demographic and medical characteristics, baseline ABI measurement, follow-up data with h information on fatal and nonfatal events<br><br>**Exclusion criteria:** Previous Hx of CHD	**1° endpoint:** Change in FRS CV risk prediction with addition of ABI<br><br>**Results:**<br>• Follow-up ranged from 3–6.7 y; 9924 (25% CVD) deaths during 480,325 person-years of follow-up.<br>• CV mortality HR for different ABI levels: Reference=1.11–1.20; ABI ≤0.60=5.58 for men; 7.04 for women. 19% of men and 36 % of women would change risk category with ABI added to FRS.	• ABI provided independent risk information and almost doubled risk of total mortality CV mortality and major coronary events when combined with FRS.<br>• Many men would move to a lower risk category, while more women would move from a lower to a higher risk category.
Fowkes FG, et al. 2014(28) 24367001	**Study type:** Prospective<br><br>**Size:** n=18 cohorts, n=44,752 pts	**Inclusion criteria:** Dataset including ABI measurement and FRS data points, follow-up for mortality and CV events.<br><br>**Exclusion criteria:** Hx CHD, invalid ABI, not vital status follow-up.	**1° endpoint:** C index (fraction of occasions where the predictor score correctly predicts the earlier event for a pair of individuals) and NRI score<br><br>**Results:**<br>• C index for major coronary events, FRS only:<br>Men: 0.67; 95% CI: 0.6–0.74;<br>Women: 0.58; 95% CI: 0.49–0.66<br>• CV mortality:<br>Men: 0.68; 95% CI: 0.63–0.74;<br>Women: 0.45; 95% CI: 0.38–0.52.<br>• Adding ABI to FRS improves men's scores modestly and women's scores substantially. Major coronary events:<br>Men: 0.69; 95% CI: 0.61–0.76;<br>Women: 0.069; 95% CI: 0.61–0.076.<br>• CV mortality:<br>Men: 0.71; 95% CI: 0.65–0.76;<br>Women: 0.65; 95% CI: 0.58–0.72<br>Prediction NRI scores:<br>• Major coronary events:	• ABI+FRS model led to improved performance mainly in women.<br>• Restricting to those at intermediate risk resulted in higher NRIs in both men and women

© American Heart Association, Inc. and American College of Cardiology Foundation

			Men: 4.3%; 95% CI: 0.0–7.6%; p=0.050;<br>Women: 9.6%; 95% CI: 6.1%–16.4%; p<0.001<br>• CV mortality:<br>Men: 5.7%; 95% CI: 2.7%–7.9%; p<0.001);<br>Women: 15.7%; CI: 11.3–20.2%; p<0.001).<br>• Restricting use of prediction model to those at intermediate risk resulted in greater effect (15.9% in men and 23.3% in women)	
**GETABI study**<br>Diehm C, et al.<br>2009(29)<br>19901192	**Study type:** Prospective cohort study<br><br>**Size:** n=6,880 pts;<br>5,392 pts=no PAD;<br>836 pts=asx PAD;<br>593 pts=sx PAD	**Inclusion criteria:** Age ≥65 y, 5 y follow-up data, mentally competent to cooperate and sign consent<br><br>**Exclusion criteria:** Life expectancy <6 mo	**1° endpoint:** Severe vascular events, CV and all-cause mortality.<br><br>**Results:**<br>• Mortality (pts /1000):<br>No PAD: 19.5;<br>Asx PAD:41.7;<br>• HR vs. no PAD: 1.66; 95% CI: 1.38–2.0;<br>Sx PAD: 53.0;<br>• HR vs. no PAD: 1.89; 95% CI; 1.55–2.30.<br>• No significant differences between asx and sx PAD groups in all-cause mortality.<br>• Composite outcome All-cause mortality and Vascular events (pts/1000):<br>No PAD: 27.2, Asx PAD: 60.4;<br>HR vs. no PAD: 1.81; CI: 1,53–2.14;<br>Sx PAD 104.7; HR compared to no PAD: 2.66; 95% CI: 2.25–3.15.<br>• Difference between PAD groups also significant (HR: 1.48; 95% CI: 1.21–1.80.<br>• No differences between PAD groups in MI, stroke, peripheral amputation. Sig differences in myocardial and peripheral revascularualrizations.	• 1 in 5 elderly pts visiting primary care clinician had PAD.<br>• Pts with PD regardless of severity had increased risk of CV events and death compared to those without PAD<br>• Sx PAD had greater risk of composite outcome of all-cause death or vascular event than asx PAD pts but no greater risk of all-cause mortality alone, MI, or stroke
**USPSTF Review**<br>Lin JS, et al.<br>2013(30)<br>24156115	**Study type:** Systematic Evidence Review<br><br>**Size:** n=1 meta-analysis, 18 population-based cohorts (52,510 pts)	**Inclusion criteria:** 3 mo follow-up; designed to evaluate treatment benefit in screen-detected persons or populations who had Asx or unrecognized PAD<br><br>**Exclusion criteria:** Pts with DM	**Results:**<br>• ABI added to other risk predictors increases but questions clinical utility or significance.<br>• No randomized studies showing improved outcomes in response to detection of Asx disease.<br>• Benefit of reclassification including ABI may be higher and clinically important in older populations at higher risk. May be most useful for pts near the thresholds of risk categories.<br>• Acknowledge the evidence demonstrating increased morbidity and mortality in Asx pts.	• Several studies currently ongoing that could give more definitive answers in the future.

© American Heart Association, Inc. and American College of Cardiology Foundation

Alahdab F, et al. 2015(31)<br>25721066	**Study type:** Systematic Review<br><br>**Size:** n=40 individual studies, 2 systematic reviews, 1 meta-analysis	**Inclusion criteria:** Studies reporting results of screening for asx pts<br><br>**Exclusion criteria:** Not original data, did not report on asx pts	**1° endpoint:** Multiple that would justify screening for asx pts: Accurate test available; disease sufficiently prevalent and mortal; screening leads to reduced morbidity and mortality; screening is not harmful<br><br>**Results:**<br>• ABI is adequate test (diagnostic accuracy=0.87; diagnostic OR: 15.33; 95% CI: 9.39–25.02; pooled sensitivity=75%; specificity=86%);<br>• PAD is prevalent (average screening yield=17.2%) and mortal (pooled HR=2.99 for all-cause mortality and 2.35 for CV mortality).<br>• No studies compared screened vs. non screened populations for mortality outcomes.<br>• ABI screening can improve FRS in risk prediction.<br>• Some evidence that screening can lead to improved morbidity<br>• Little evidence about potential harm or cost-effectiveness. Discussed potential bleeding risk of ASA with no proven benefit	• Yield of ABI screening text in asx pts depends on prevalence of traditional risk factors<br>• No high quality evidence supports 'pt-important' benefits from screening low-risk individuals<br>• High-risk individuals may not need screening since there is already indication to treat their risk
**Health ABC Study**<br>Hiramoto JS, et al. 2014(32)<br>23512905	**Study type:** Prospective<br><br>**Size:** n=2,797 pts	**Inclusion criteria:**<br>• Age 70–79 y<br>• No disability<br>• No functional limitation<br>• Baseline ABI measurement<br><br>**Exclusion criteria:**<br>• Self-reported Hx of claudication<br>• LEX revascularization	**1° endpoint:** Development of CV events/mortality, clinical PAD (assessed every 6 mo). Median follow-up 9.37 y.<br><br>**Results:** Baseline low ABI associated with black race, elevated SBP, prevalent CVD, and DM. Men had higher incident clinical PAD compared to women across all categories of ABI. Men had higher rates of CHD death and incident MI except in the 1.3 category, where women had higher rates of MI and CHD death. Women had higher rates of incident stroke.<br>ABI <0.90<br>• **CHD Death:**<br>Men: HR: 4.38; 95% CI: 1.8–10.6;<br>Women HR: 4.96; 95% CI: 1.53–16.01.<br>• **Incident PAD**:<br>Men: HR:7.85; 95% CI: 4.44–13.90;<br>Women: HR: 5.56; 95% CI: 2.44–12.67.<br>• **Stroke**:<br>Men: HR: 1.17; 95% CI: 0.56–2.47;<br>Women: HR:2.58; 95% CI: 1.35–4.92;<br>• **Incident MI:**<br>Men: HR:2.26; 95% CI: 1.19–4.30;<br>Women: HR: 2.55; 95% CI: 1.13–5.72<br>• **Other points:**	• Subclinical PAD seems to affect women disproportionately compared to men<br>• Higher prevalence of borderline ABI in women; associated with poor outcomes<br>• Category of ABI >1.3; associated with poorer CV outcomes in women

© American Heart Association, Inc. and American College of Cardiology Foundation

			In women with ABI >1.3, Incident MI HR: 9.31; 95% CI: 4.01–21.63; Incident stroke HR: 4.81; 95% CI: 2.27–10.30	
Bundó M, et al. 2010(33) 21035692	**Study type**: Follow-up observational study (10 y, mean 7.7 y)<br><br>**Size**: n=262 pts	**Inclusion criteria**: Type 2 DM<br><br>**Exclusion criteria**: Sx PAD or previously diagnosed	**1° endpoint**: Mortality (cause of death), CVD, CHD, Disease progression (from normal to abnormal, or 15% decrease in ABI)<br><br>**Results:**<br>• Normal vs. abnormal baseline ABI:<br>• Mortality: 16.8% vs. 52.8%<br>• Nonfatal CV Events: 19.4% vs. 38.9%<br>• CVD: 8.2% vs. 30.6%	• Small sample size<br>• Significant differences between groups in CV outcomes
TsivgoulisF, et al. 2012(34) 22138142	**Study type**: Prospective longitudinal cohort study<br><br>**Size**: n=176 pts	**Inclusion criteria**:<br>• Asx PAD<br>• Acute ischemic stroke or TIA<br><br>**Exclusion criteria**: Sx PAD	**1° endpoint**: 30 d recurrence of stroke<br><br>**Results:** PAD pts had higher 30 d recurrence of stroke (19.2%; 95% CI: 4.1–34.3; vs. 3.3%: 95% CI: 0.4–6.2. Final multivariate analysis HR: 12.46; 95% CI: 2.22–70.0; p=0.004	• Very small numbers of PAD pts<br>• Asx PAD pts have higher short term risk of recurrent stroke
Bouisset, F. et al 2012(35) 22513182	**Study type**: Prospective, longitudinal cohort study (median follow-up 7.2 y; range 5.7–8.6 y).<br><br>**Size**: n=710 in final analysis	**Inclusion criteria**:<br>• Nonconsecutive male pts age 45–74 y, with stable CHD.<br>• ABI measured; classified as no PAD (n=446) or subclinical PAD (n=181), sx PAD (n=83)<br><br>**Exclusion criteria**:<br>• Acute coronary episode within past 7 d<br>• Hx cancer	**1° endpoint**: All-cause mortality; prognostic effect of PAD status on all-cause death assessed by Cox regression analysis.<br><br>**Results:**<br>• Median 7.2 y survival rates No PAD=87.4%; Subclinical PAD=78.5%; clinical PAD=70.1%<br>• Cox regression analysis: Unadjusted model:<br>• HR for subclinical PAD vs. no PAD: 1.88; 95% CI: 1.27–2.78; p=0.001.<br>• HR for clinical PAD vs. no PAD: 2.57; 95% CI: 1.62–4.07; p<0.001.<br><br>Adjusted model:<br>• HR for subclinical PAD vs. no PAD: 1.65; 95% CI: 1.11–2.44; p=0.01.<br>• HR for clinical PAD vs. no PAD: 2.11; 95% CI: 1.28–3.47.	• PAD common in this population<br>• Detection of subclinical PAD in pts with known coronary disease provides additional information for long-term mortality risk evaluation<br>• Limitation: Studied only men
Sen S, et al. 2009(36) 19713540	**Study type**: Prospective longitudinal hospital-based cohort<br><br>**Size**: n=102 pts	**Inclusion criteria**:<br>• Stroke<br>• TIA<br>• Asx PAD vs. normal ABI<br><br>**Exclusion criteria**:	**1° endpoint**: Composite vascular events including stroke, TIA, MI and vascular death median 2.1 y<br><br>**Results:**<br>• Asx PAD (26%) vs. no PAD (74%)<br>Composite vascular events: 50% vs. 16%	• Small sample, single site<br>• Pts with stroke or TIA and Asx PAD have worse outcomes than those without Asx PAD.

© American Heart Association, Inc. and American College of Cardiology Foundation

		• <18 y<br>• Intercerebral hemorrhage<br>• Coma<br>• Conditions limiting life expectancy to <12 mo<br>• Sx PAD	• Cumulative event-free survival: 1.6; 95% CI: 1.2–1.9 y vs. 2.5 y; 95% CI: 2.4–2.6 y; p=0.0001	
Ratanakorn D, et al.<br>2012(37)<br>21236702	**Study type:** Cross-sectional<br><br>**Size:** n=747 Thai pts	**Inclusion criteria:** Consecutive stroke registry pts with ischemic stroke or TIA within 7 d confirmed by CT or MRA; age ≥18 y,<br><br>**Exclusion criteria:** Hx of previous or current Sx PAD; severe disabling stroke; ET intubation and mechanical ventilation; incomplete ABI data.	**1° endpoint:** Prevalence of PAD among total population and subgroups<br><br>**Results:**<br>• Prevalence of abnormal ABI=18/1%; Multivariate analysis abnormal ABI related to female sex (OR: 1.61; 95% CI: 1.09–2.40; p=0.017); Age ≥60 y (OR: 3.54; 95% CI: 2.14–5.85; p<0.001); Previous ischemic events including CAD (OR: 2.55; 95% CI: 1.47–4.43; p=0.001); CVD (OR: 2.15; 95% CI: 1.37–3.55; p=0.002).<br>• Prevalence in pts ≥60 y =25%; ≥70 y =30%. No significant relationship with atherosclerotic risk factors. Strongest prevalence of abnormal ABI in large artery disease and cardioembolic stroke subtypes.	• Early detection of PAD may facilitate treatment and identify excess risk of subsequent stroke or other CV events.
Ramos R, et al<br>2016(38)<br>26868687	**Study Type:** Cohort design for matched pair analysis on the basis of study inclusion date and propensity for statin treatment<br><br>**Size:** n=5,480 Spanish pts from the Information System for Development of Research in Primary Care database.	**Inclusion Criteria:**<br>• 35–85 y<br>• ABI measurement documented<br>• ABI<0.95;<br><br>**Exclusion Criteria:** Previously hx of sx PAD, CHD, stroke or revascularization procedure.	**1°endpoint:** HR of absolute risk reduction in MACE and all-cause mortality and 1-year number needed to treat for 'new' statin users vs. non-statin users followed 2–7 y.<br><br>**Results:**<br>• MACE rates<br>New users: 19.7 (95% CI:17.2 to 22.5)<br>Non-users: 24.7 (95% CI: 21.8 to 27.8)<br>(20% RRR)<br>1 y NNT: 200<br>• All-cause mortality rates<br>New users: 24.8 (95% CI: 22.0 to 27.8)<br>Non-users: 30.3 (95% CI: 27.2 to 33.6)<br>(19% RRR)<br>1 y NNT 239<br>• NNT decreased with ABI cutpoint	• First study to report the association between statins and both MACE and mortality reduction among individuals free of clinical CVD, but with asx PAD identified by ABI.<br>• Reduction observed regardless of CVD risk scores at baseline<br>• Absolute reduction in MACE and all-cause mortality similar to that seen in secondary prevention studies.
Jiménez M, et al.<br>2014(39)<br>24529125	**Study type:** Cross-sectional<br><br>**Size:** Random population sample, n=933 pts	**Inclusion criteria:** Moderate to high vascular risk (REGICOR score >5%<br><br>**Exclusion criteria:** Hx	**1° endpoint:** Presence of carotid stenosis<br><br>**Results:** Prevalence of SCCA higher in those with REGICOR score >10% and in pts with asx PAD. Asx PAD increased risk of SCCA by more than 5-fold. ABI diagnosing SCCA: Sensitivity=0.3;	• ABI emerged as tool to identify pts with high risk of having subclinical carotid or intracranial atherosclerosis<br>• Helps target screening,

© American Heart Association, Inc. and American College of Cardiology Foundation

		stroke, PAD, CAD	95% CI: 0.18–0.42; specificity=0.95 (95% CI: 0.93-0.96); PPV=0.26 (95% CI: 0.15–0.37), NPV= 0.95 (95% CI: 0.94–0.97).	increasing cost-effectiveness
McDermott MM, et al.<br>2000(40)<br>10704168	**Study type**: Cross-sectional<br><br>**Size**:<br>• Stratified random sampling of 32,538<br>• Final sample n=574 asx pts	**Inclusion criteria**: Community dwelling disabled women ≥65 y participating in Women's Health and Aging Study<br><br>**Exclusion criteria**: Mini-mental score <18	**1° endpoint**: Prevalence of Asx PAD; relationship between physical functioning and Asx PAD.<br><br>**Results**:<br>• ABI<0.90=198 (34.5%)<br>• ABI<0.50=48 (8.4%)<br>• Subjective and objective measures of mobility and lower extremity function, all statistically lower in Asx PAD compared to non-PAD.	• Asx PAD is independently associated with impaired lower extremity functioning.
**WALCS Study**<br>McDermott MM, et al.<br>2001(5)<br>11585483	**Study type**: Cross-sectional, new pts consecutively identified and pts already identified with PAD from large general medicine practice.<br><br>**Size**:<br>• n=430 men and women with PAD<br>• n=130 without PAD.<br>ASX active=63<br>ASX inactive=28	**Inclusion criteria**<br>Diagnosed with PAD (ABI<0.90); ≥55 y<br><br>**Exclusion criteria**:<br>• ABI >1.5;<br>• Normal ABI,<br>• Dementia<br>• Amputation<br>• Non-English speaking<br>• Wheelchair bound<br>• Nursing home resident<br>• Recent surgery	**1° endpoint**: 6 MWT scores, 7 d physical activity, SPPB, Questionnaires<br><br>**Results**:<br>• PAD sj. Divided into 6 categories. asx 2 categories: active vs. inactive<br>• 33.3% active and 53.6% inactive PAD pts reported sx during 6MWT<br>• All PAD groups had worse functioning that non-PAD group<br>• Asx inactive functioning similar to claudication group<br>• Asx inactive functioning poorer than claudication group	N/A
**WALCS Study**<br>McDermott MM et al.,<br>2004(41)<br>15280343	**Study type**: Prospective cohort study of PAD pts with differing types of leg symptoms (same cohort as above) 2 yr follow-up<br><br>**Size**:<br>• n=417 pts with PAD<br>• n=259 pts without PAD	**Inclusion criteria**<br>• ABI <0.90<br>• ≥55 y<br>• Non-PAD group identified from internal medicine practice<br><br>**Exclusion criteria**:<br>• ABI >1.5<br>• Normal ABI<br>• Dementia<br>• Amputation<br>• Non-English speaking<br>• Wheelchair bound	**1° endpoint**: Decline in 6 MWT, Usual pace and fastest-pace 4-Meter velocity, summary performance score<br><br>**Results**: Baseline physical functioning poorer in asx PAD than non-PAD; decline greater on all measures. asx PAD has greater decline in 6 MWT than pts with claudication	• Asx pts have >2 y decline in physical functioning compared to asx non-PAD pts. 6 MWT decline greater in asx pts that IC group.

© American Heart Association, Inc. and American College of Cardiology Foundation

		• Nursing home resident<br>• Recent surgery		
**WALCS Study**<br>McDermott MM, et al.<br>2006(42)<br>16389250	**Study type**: Prospective cohort study with median follow-up of 36 mo<br><br>**Size**: n=417 men and women with PAD	**Inclusion criteria:**<br>• Age ≥55 y<br>• ABI <0.90<br>• Non-PAD group identified from internal medicine practice<br><br>**Exclusion criteria**: ABI >1.5; Normal ABI, dementia, amputation, nonEnglish speaking, wheelchair bound, nursing home resident	**1° endpoint:** Rate of decline in 6 MWT, Usual pace and fastest-pace 4-Meter velocity, summary performance score<br><br>**Results:**<br>• Pts separated into groups based on physical activity level (walk 3 or more times per wk vs. less frequently).<br>• Asx PAD pts who walked for exercise 3 or more times per wk had less functional decline than those who walked for exercise less frequently	• Greater physical activity associated with less decline in physical functioning in ASX PAD pts.
**WALCS study**<br>McDermott MM, et al.<br>2010(43)<br>20550604	**Study type**: Prospective observational study<br><br>**Size**: n=415 pts followed up to 7 y	**Inclusion criteria**: See above<br><br>**Exclusion criteria**: See above	**1° endpoint**: 6 MWT, becoming unable to walk up and down a flight of stairs or walk ¼ mile without assistance in pts without mobility loss at baseline<br><br>**Results**: Always asx pts had greater mobility loss than pts with claudication (HR: 2.94; 95% CI: 1.39–6.19; p=0.005).<br>Asx pts did not demonstrate as much decline in 6MWT as pts with claudication.	N/A
**LIFE study**<br>McDermott MM, et al.<br>2013(44)<br>24222666	**Study type**: Cross-sectional study in community-dwelling sedentary older adults<br><br>**Size**: n=1,566 pts categorized into categories of:<br>Definite PAD , borderline PAD, low normal ABI, no PAD	**Inclusion criteria**:<br>• Age 70–89 y<br>• Community-dwelling<br>• Sedentary (<125 min of physical activity/wk<br>• Functional limitations<br><br>**Exclusion criteria**: N/A	**1° endpoint**: Physical function measures<br><br>**Results:**<br>• 65% of definite PAD pts asx.<br>• In asx pts lower ABI values associated with longer 4 meter walk time and slower walking velocity	• Lower extremity atherosclerosis may be common preventable cause of functional limitations in older persons.<br>• Even in individuals who are considered functionally impaired, low ABI is associated with greater functional impairment.
Niazi K, et al.<br>2006(45)<br>17039537	**Study type**: Cross-sectional study<br><br>**Size**: n=107 pts, 208 limbs	**Inclusion criteria**:<br>• ABI performed within 30 d prior to DSA<br><br>**Exclusion criteria:**<br>• Pts with noncompressible	**1° endpoint**: N/A<br><br>**Results:**<br>• Sensitivity of the HAP and LAP ABI for diagnosis of PAD was 69% and 84%, respectively<br>• Overall accuracy of HAP and LAP ABI was 72% and 80%,	• LAP ABI has better sensitifvity and overall accuracy in comparison to the HAP ABI in diagnosing PAD

© American Heart Association, Inc. and American College of Cardiology Foundation

		vessels<br>• ABI >1.40	respectively	

ABI indicates ankle-brachial index; ASA, acetylsalicylic acid; asx, asymptomatic; BL, baseline; CAD, coronary artery disease; CHD, coronary heart disease; CI indicates confidence interval; CLI, critical limb ischemia; CT, computed tomography; CVD, cardiovascular disease; CV, cardiovascular; DM, diabetes mellitus; DSA, digital subtraction angiography; ET, endotracheal; FRS, Framingham risk score; HAP, high ankle pressure; HR, hazard ratio; HTN, hypertension; IC, intermittent claudication; LAP, low ankle pressure; MACE, major adverse cardiovascular event; LEX, lower extremity; MCS, mental health composite score; MI, myocardial infarction; MRI, magnetic resonance imaging; MWT, mean walking time; N/A, not applicable; NHANES, National Health and Nutrition Examination Survey; NPV, negative predictive value; NRI, net reclassification improvement; NNT, number needed to treat OR, odds ratio; PAD, peripheral artery disease; PCS, physical composite score, PFWT, pain free walking time; PPV, positive predictive value; pt, patient; RR, relative risk; SBP, systolic blood pressure; SCCA, significant stenosis >50%; SF, Short Form; Sx, symptomatic; TIA, transient ischemic attack; US, United States; and WIQ, Walking Impairment Questionnaire.

Evidence Table 5. Nonrandomized Trials, Observational Studies, and/or Registries of Physiological Testing–Section 3.2.

Study Acronym; Author; Year Published	Study Type/Design; Study Size	Patient Population	Primary Endpoint and Results (include P value; OR or RR; & 95% CI)	Summary/Conclusion Comment(s)
Rutherford RB, et al.<br>1997(46)<br>9308598	**Study type:** Observational study of SDP/PVR compared to the gold standard of angiography for Dx of PAD<br><br>**Size:** n=114 pts undergoing SDP/PVR and angiography	**Inclusion criteria:** 11 normal volunteers and 103 pts having had angiography<br><br>**Exclusion criteria:** No angiography	**1° endpoint:** Correct classification of PAD<br><br>**Results:** 97% of normal limbs were correctly classified by SDP/PVR, 86% correct classification using either SDP or PVR	N/A
Eslahpazir BA, et al.<br>2014(47)<br>24200144	**Study type:** Single healthcare system, retrospective cohort of all pts with SDP/PVR /DWand angiography 2009–2011 (blinded readers for each technique)<br><br>**Size:** n=89 limbs	**Inclusion criteria:** Having both SDP/PVR and angiography<br><br>**Exclusion criteria:** Those with incomplete reports	**1° endpoint:** Determination of the most accurate diagnostic value<br><br>**Results:** 66% diagnostic accuracy (presence and level of PAD), less variability in interpretation using pressure than in waveform interpretation	• Readings reflecting incompressibility were not utilized
Ouriel K, et al.<br>1982(48)<br>7079971	**Study type:** Observational<br><br>**Size:** n=218 pts (372 limbs) and 25 normal pts	**Inclusion criteria:** Able to have ABI, treadmill ABI and reactive hyperemia<br><br>**Exclusion criteria:** N/A	**1° endpoint:** Sensitivity and specificity of exercise ABI to detect PAD<br><br>**Results:** 97% and 96% stress testing value is in pts with symptoms and normal	N/A

© American Heart Association, Inc. and American College of Cardiology Foundation

	and 10 stable claudicants		ABI	
Aerden D, et al. 2011(49) 21514102	**Study type**: Prospective study<br><br>**Size**: n=187 lower extremities	**Inclusion criteria**: Pts in diabetic foot clinic with angiography and ABI. All with nonhealing foot ulcer and/ or absent pulse<br><br>**Exclusion criteria**: Distal arterial bypass	**1° endpoint**: Correlation of ABI and angiography in pts seen in diabetic foot clinic<br><br>**Results**: Correlation between ABI and angiographic disease was weak (<0.48). ABI could not be determined in 34%. In those with calcifications, correlation with angiographic severity was worse.	• Arterial calcification evaluated using plain X-ray<br>• Biphasic Doppler signals useful, monophasic not useful
Park SC, et al. 2012(50) 922783531	**Study type**: Retrospective analysis of angiography, ABI, TBI (many with ulcers)<br><br>**Size**: n=30 limbs	**Inclusion criteria**: TBI <0.6 or ABI < 0.9, diabetic gangrene)<br><br>**Exclusion criteria**: N/A	**1° endpoint**: ABI or TBI correlation with angiographic disease<br><br>**Results**: 13 of 30 limbs with abnormal TBI, 100% specificity and sensitivity	• Studies with normal population and TBI had sparse arterial imaging (did not meet QUADAS standards)
Weinberg I, et al. 2013(51) 22899598	**Study type**: Retrospective study<br><br>**Size**: n=116 limbs	**Inclusion criteria**: Pts with ABI >1.4, angiography and TBI<br><br>**Exclusion criteria**: N/A	**1° endpoint**: Angiographic evidence of PAD with TBI <0.7<br><br>**Results**: 92% of pts with TBI <0.7 had angiographic evidence of PAD	• 67% DM and 19% on hemodialysis
Suominen V, et al. 2008(52) 18313338	**Study type**: Retrospective ABI >1.3 and angiography<br><br>**Size**: n=69 pts of the total 1,762 pts seen in the vascular lab	**Inclusion criteria**: TBI, ABI and angiography<br><br>**Exclusion criteria**: N/A	**1° endpoint**: Presence of abnormal ABI >1.3, TBI <0.6 and angiographic evidence of disease<br><br>**Results**: High sensitivity and specificity	• Larger population with normal ABI and abnormal TBI
Aboyans V, et al. 2008(22) 18692981	**Study type**: Cross-sectional<br><br>**Size**: n=510 pts	**Inclusion criteria**: ambulatory pts presenting to vascular lab<br>**Exclusion criteria**: N/A	**1° endpoint**: Association of risk factors with ABI >1.4 and ABI <0.9 and disease presence by TBI<br><br>**Results**: In 84.2% of cases, diabetic limbs with ABI ≥1.40 had abnormal results in at least 1 of the 2 noninvasive vascular indicators, suggestive of concomitant occlusive disease.	• 50% with DM<br>• No angiographic correlations
Wagener JS and Hendricker C 1987 (53)	**Study type**: Prospective study of repeated measurements of $TcPO_2$	**Inclusion criteria**: Healthy nonsmoking adults	**1° endpoint**: Variability of repeat measures	• Mornings and afternoons over 7 d to 7 mo with variable inspired oxygen

© American Heart Association, Inc. and American College of Cardiology Foundation

3677809	**Size**: n=10 pts	**Exclusion criteria**: Respiratory symptoms	**Results**: Higher for $TcPO_2$ than Sa O_2 pulse oximetry	
Tsai FW, et al. 2000(54) 10876204	**Study type**: prospective vascular lab pts with SPP and toe pressures<br><br>**Size**: n=85 limbs, 43 of 53 pts with DM	**Inclusion criteria**: SPP and TBI in the vascular lab<br><br>**Exclusion criteria**: N/A	**1° endpoint**: Correlation of TBI and SPP<br><br>**Results**: Correlation 0.87 ($p<0.01$) for all	• Laser Doppler SPP<br>do not know if any had ulcers or rest pain
Yamada T, et al. 2008 (55) 18241755	**Study type**: retrospective<br><br>**Size**: n=211 pts (50% with DM or hemodialysis)	**Inclusion criteria**: vascular lab referral for arterial insufficiency due to arteriosclerosis obliterans<br>ABP, TBP, $TcOO_2$ and SPP<br><br>**Exclusion criteria**: N/A	**1° endpoint**: Ability of test to predict wound healing<br><br>**Results**: Healing more likely at TBP >30 and SPP >40 mm Hg,<br>Best prediction SPP + TBP	• 26 with ulcer or gangrene leading to amputation<br>• 13% with high ABI<br>• SPP correlates with ABP, TBP and $TcPO_2$<br>• $TcPO_2$ did not work well to predict healing
Bosanquet DC, et al. 2014 (56) 24841052	**Study type**: Meta-analysis<br><br>**Size**: n=15 cohort studies with 1,868 individual limbs	**Inclusion criteria**: direct (to angiosome) vs. indirect infrapop revascularization<br><br>**Exclusion criteria**: N/A	**1° endpoint**: Wound healing and limb salvage, mortality<br><br>**Results**: Direct revascularization of the tibial vessels appears to result in improved wound healing and limb salvage rates compared with indirect revascularization, with no effect on mortality or reintervention rates.	• Marginal quality
Carter SA 1969 (57) 5818299	**Study type**: Technique to measure systolic pressures in the lower extremities<br><br>**Size**: n=288 limbs	**Inclusion criteria**: 202 limbs with disease and 86 limbs without angiographically documented disease<br><br>**Exclusion criteria**: Inability to tolerate cuff inflation	**1° endpoint**: Ability to determine PAD with systolic pressure assessment<br><br>**Results**: Well tolerated and excellent correlation with angiography	• Description of case detail included
Carter SA and Tate RB 1996 (58) 8752037	**Study type**: Toe pressures in consecutive pts referred to 1 vascular lab<br><br>**Size**: n=182 pts, 352 limbs	**Inclusion criteria**: Referral to lab for segmental pressures<br><br>**Exclusion criteria**: N/A	**1° endpoint**: Clinical correlation<br><br>**Results**: Low toe PW amplitude is significantly related to the occurrence of rest pain, skin breakdown, or both after controlling is done for the value of the toe pressure and ABI or ankle pressure	• Aim: to test whether addition of the measurements of toe PW, which depend on distal perfusion, to pressure measurements could improve the determination of the severity of arterial disease and the presence of CLI.
Ramsey DE, et al. 1983 (59) 6833352	**Study type**: Toe pressures were correlated with ankle pressures, clinical symptoms, and the	**Inclusion criteria**: Pts with ulcers presenting to the vascular lab<br><br>**Exclusion criteria**: Absence of ulcer	**1° endpoint**: Relationship of toe pressure to healing<br><br>**Results**: The TBI, arm minus toe	Toe pressure >30 mm Hg associated with good healing potential

© American Heart Association, Inc. and American College of Cardiology Foundation

	presence or absence of diabetes in 294 limbs<br><br>**Size:** n=294 limbs		pressure, and the absolute toe pressure had an average sensitivity and specificity of 85% and 88% for asx limbs and 89% and 86% for ischemic limbs.	
Biancari F and Juvonen T 2014 (60) 24491282	**Study type:** Meta-analysis<br><br>**Size:** n=9 studies (no RCT)	**Inclusion criteria:** 715 legs treated by direct revascularization according to the angiosome principle and 575 legs treated by indirect revascularization<br><br>**Exclusion criteria:** N/A	**1° endpoint:** Wound healing<br><br>**Results:** Direct revascularization of the foot angiosome affected by ischemic tissue lesions may improve wound healing and limb salvage rates compared with indirect revascularization	•Aim: The efficacy of angiosome-targeted revascularization to achieve healing of ischemic tissue lesions of the foot and limb salvage is controversial.
Vincent DG, et al. 1983 (61) 6833348	**Study type:** Observational<br><br>**Size:** n=219 limbs	**Inclusion criteria:**<br>• Presence of limb<br>• Both asx volunteers and pts with PAD presenting to the vascular lab were studied	**1° endpoint:** Diagnostic accuracy toe pressure and ABI<br><br>**Results:** Toe pressure was the most reliable indicator of occlusive disease, and was able to assess disease distal to the ankle	• 5 groups were separated using the ankle-brachial and the toe-ankle systolic pressure ratios: normal, claudication, limb salvage, claudication/incompressible arteries, and limb salvage/incompressible arteries.
Mahe G, et al. 2015 (62) 26252297	**Study type:** Retrospective analysis of clinical results<br><br>**Size:** n=12,312 consecutive pts	**Inclusion criteria:** Consecutive pts underwent exercise ABI<br><br>**Exclusion criteria**: Inability to exercise	**1° endpoint:** Diagnosis of PAD using the 2 criteria<br><br>**Results:** Only small overlap between the 2 populations of PAD identified	• To determine whether postexercise criteria for PAD diagnosis recommended by the AHA identifies the same group of PAD pts.
Nicolaï SP, et al. 1990 (63) 19631868	**Study type:** Meta regression analysis<br><br>**Size:** n=8 studies, 658 pts	**Inclusion criteria:** Trials assessing reliability oftreadmill testing were identified. Inclusion criteria were the use of a C- or G-protocol, repetition of this protocol, and a retrievable ICC.	**1° endpoint:** Reliability of treadmill testing<br><br>**Results:** For ICD, the estimated reliabilities of the C- and G-protocol (as assessed by the ICC) were 0.85 (95% confidence interval [CI]: 0.82-0.88) and 0.83 (95% CI: 0.80-0.85), respectively, without dependency of the reliability on velocity or grade.	For ACD, the reliability was significantly better for the G-protocol (0.95, 95% CI: 0.94-0.96) than for the C-protocol. Moreover, the reliability of the C-protocol was dependent on grade of the treadmill (0%, 10%, and 12%) with a mean ICC of 0.76 (95% CI: 0.54-0.88), 0.89 (95% CI: 0.86-0.91), and 0.91 (95% CI 0.88-0.92), respectively
Laing SP and Greenhalgh RM 1980 (64) 7357254	**Study type:** Observational<br><br>**Size:** n=26 pts	**Inclusion criteria:** Presentation with claudication	**1° endpoint:** Comparison of 2 protocols<br><br>**Results:** The pts walked for 1 or 2 min at 4 km/h and 1 or 2 min at 6 km/h, and the fall in pressure was the same when measured immediately after exercise.	N/A

© American Heart Association, Inc. and American College of Cardiology Foundation

Raines JK, et al. 1976 (65) 1246689	**Study type**: Observation<br><br>**Size**: n=4,500 procedures	**Inclusion criteria**: Pts in the vascular lab	**1° endpoint**: Criteria for management<br><br>**Results:** Excellent reproducibility for physiologic testing including pulse volume recording and segmental pressures	N/A
Sumner DS and Strandness DE 1969 (66) 5777227	**Study type**: Observation	**Inclusion criteria**: Pts presenting to the vascular lab with claudication	**1° endpoint**: Relationship between calf blood flow and ankle blood pressure in pts with claudication<br><br>**Results:** Close correlation	N/A
Castronuovo JJ, et al. 1997(67) 9357464	**Study type**: Prospective double blind study<br><br>**Size**: n=53 pts	**Inclusion criteria**: Vascular lab referrals for CLI<br><br>**Exclusion criteria**: Sepsis or need for guillotine amputation	**1° endpoint**: Prediction of wound healing by SPP<br><br>**Results:** SPP measurements identified 31 of 32 limbs diagnosed as having CLI by clinical evaluation (i.e., group I, those limbs that required vascular reconstruction or major amputation)	• DM and wound size similar in 2 groups<br>• The sensitivity of SPP <30 mm Hg as a diagnostic test of CLI was 85%, and the specificity was 73%. The overall diagnostic accuracy of SPP less than 30 mm Hg as a diagnostic test of CLI was 79.3% ($p<0.002$, Fischer's exact test).
Biotteau E, et al. 2009(68) 20087286	**Study type**: Retrospective matched paired study<br><br>**Size**: n=120 pts	**Inclusion criteria**: Pts presenting to the vascular lab with suspected CLI	**1° endpoint**: Whether a difference can be found for chest and foot $TcPo_2$ respectively between pts with and without DM referred for clinically suspected CLI.<br><br>**Results:** TcPo2 is lower at the chest but not at the foot level in diabetic than in non-diabetic pts with suspected CLI.	• Evenly matched DM and non-DM<br>• 30 mm Hg threshold applicable to both populations
Bunte MC, et al. 2015(69) 26892836	**Study type**: Observational<br><br>**Size**: n=89 consecutive pts	**Inclusion criteria**: CLI and presentation with rest pain	**Results**: Among 31 CLI pts with available ABI and TBI results, 19 (61%) had a TBI <0.7 *and* a non-compressible or resting ABI <0.9. Conversely, no pts with a borderline or normal ABI (0.9–1.4) had a normal TBI (≥0.7)	• Among a contemporary, real-world CLI population, 29% had near-normal or normal ABI, despite having significant infragenicular arterial disease.
Stein R, et al. 2006(70) 16669410	**Study type**: Retrospective review<br><br>**Size**: n=396 pts	**Inclusion criteria**: Sx outpatients referred for measurement of segmental blood pressure, the ABI or pulse volume recordings by physicians not specialized in the evaluation and management of pts with PVD	**1° endpoint**: Diagnostic utility of measuring the ABI at rest in pts referred to the vascular laboratory for evaluation of suspected PAD<br><br>**Results:** Nearly half of pts referred to the outpatient vascular laboratory because of	• Diagnostic accuracy was improved with pulse volume recordings and exercise ABI

© American Heart Association, Inc. and American College of Cardiology Foundation

			suspected arterial disease had a normal resting ABI	
Shishehbor MH, et al. 2016(71) 26860642	**Study type:** Observational<br><br>**Size:** n=237 pts; 40 pts with available TBI	**Inclusion criteria:**<br>• Pts in the IN.PACT DEEP Trial<br>• Isolated infrapopliteal disease<br>• Available ABI	**1° endpoint:** Diagnostic measurement of ABI and TBI to diagnose lower extremity ulcers and severe disease<br><br>**Results:** 1/3 of pts with CLI and severe isolated infrapopliteal disease have normal or incompressible ABIs. Only a few pts met the hemodynamic criteria for CLI according to cutoffs suggested for ABI (6%) and ankle pressure (16%) defined by multiple guidelines.	• Current recommended hemodynamic pressures to diagnose CLI are insensitive and failed to identify a significant portion of pts with lower extremity ulcers and angiographically proven severe disease. Toe pressure is more sensitive in pts with CLI.

ABI indicates ankle-brachial index; AHA, American Heart Association; asx, asymptomatic; CLI, critical limb ischemia; DM, diabetes mellitus; ICC, intraclass correlation coefficient; ICD, International Classification of Disease; N/A, not applicable; PAD, peripheral artery disease; PVD, peripheral vascular disease; PVR, pulse volume recordings; PW, pulse wave; RCT, randomized controlled trial; Sa O_2, oxygen saturation; SDP, segmental Doppler pressure; SPP, skin perfusion pressure; sx, symptomatic; TBI, toe-brachial index; TBP, toe blood pressure; and $TcPO_2$, transcutaneous oxygen pressure.

Evidence Table 6. Nonrandomized Trials, Observational Studies, and/or Registries of Imaging for Anatomic Assessment (Ultrasound, CTA, MRA, Angiography)–Section 3.3.

Study Acronym; Author; Year Published	Study Type/Design; Study Size	Patient Population	Primary Endpoint and Results (include P value; OR or RR; & 95% CI)	Summary/Conclusion Comment(s)
**PIVUS study**<br>Wilkström J, et al. 2008(72) 18300136<br><br>Wilkström J, et al. 2009(73) 19446989	**Study type:** Observational test comparison<br><br>**Size:** n=306 pts	**Inclusion criteria:**<br>• General population register Sweden<br>• Age 70 y<br><br>**Exclusion criteria:** Unable to have WBMRA<br><br>**Gold standard:** WBMRA. Stenosis ≥50%<br><br>**ABI method:** Doppler	**1° endpoint:** Presence of stenosis in pelvic or leg arteries in right or left legs<br><br>**Results:**<br>Sensitivity:<br>• Right: 20 (10, 34)<br>• Left: 15 (7, 27)<br>Specificity:<br>• 99 (96, 100)<br>• 99 (96, 100)<br>PPV:<br>• 83 (51, 97)<br>• 82 (48, 97)<br>NPV:<br>• 84 (79, 88)	• Low sensitivity but good PPV.<br>• High specificity. Similar results (not shown) to detect occlusion, except lower PPV

© American Heart Association, Inc. and American College of Cardiology Foundation

			• 80 (74, 84)	
Guo X, et al. 2008(20) 18362433	**Study type:** Observational test comparison<br><br>**Size:** n=298 pts	**Inclusion criteria:**<br>• Age ≥35 y<br>• Cardiology clinic: referrals for DSA & ABI<br><br>**Exclusion criteria:** Severe DM & hypertension<br><br>**Gold standard:**<br>• DSA.<br>• Stenosis ≥50%<br><br>**ABI method:**<br>Oscillometry	**1° endpoint:** Presence of stenosis below aorto-iliac bifurcation in leg with lower ABI<br><br>**Results:**<br>Sensitivity: 76 (N/A)<br>Specificity: 90 (N/A)<br>PPV: 36 (N/A)<br>NPV: 98 (N/A)	• Moderate sensitivity and good specificity. No indication of % with PAD symptoms but low prevalence of PAD on DSA (7%) suggests it was negligible.<br>• However 53% had coronary heart disease and 13% stroke.
Clairotte R, et al. 2009(74) 19366974	**Study type:** Observational test comparison<br><br>**Size:** n=63 pts	**Inclusion criteria:** Referrals to clinic for duplex<br><br>**Exclusion criteria:** DM<br><br>**Gold standard:**<br>• Duplex ultrasound<br>• Velocity ratio ≥2 for stenotic:proximal segments<br><br>**ABI method:**<br>Doppler	**1° endpoint:** Presence of stenosis in iliac to ankle arteries<br><br>**Results:**<br>Sensitivity: 73 (N/A)<br>Specificity: 98 (N/A)<br>PPV: 98 (N/A)<br>NPV: 78 (N/A)	• Moderate sensitivity & very good specificity. No indication of % pts with PAD symptoms but only 14% had “clinical PAD”.<br>• Duplex ultrasound not ideal gold standard.<br>• Small study.
Burbelko M, et al. 2013(75) 23188773	**Study type:** Observational<br><br>**Size:** n=152 pts	**Inclusion criteria:** Underwent MRA and DSA of the lower extremities within 30 d.<br><br>**Exclusion criteria:** N/A	**1° endpoint:** Evaluation of stenosis grade and image quality<br><br>**Results:**<br>Sensitivity: 73–93<br>Specificity: 64–89	• CE-MRA demonstrates good sensitivity and specificity<br>• CE-MRA is standardizable and shows good inter-observer agreement<br>• Use of CE-MRA as alternative to intra-arterial DSA is well justified
Shareghi S, et al. 2010(76) 19753637	**Study type:** Observational<br><br>**Size:** n=28 pts	**Inclusion criteria:** consecutive pts with sx lower extremity IC and an abnormal ABI (ABI<0.9)<br><br>**Exclusion criteria:** N/A	**1° endpoint:** N/A<br><br>**Results:**<br>Sensitivity: 99<br>Specificity: 98	• MDCT demonstrated accurate detection of hemodynamically significant disease of the lower extremities

© American Heart Association, Inc. and American College of Cardiology Foundation

De Vries SO, et al.<br>1996(77)<br>8796687	**Study type:** Meta-analysis<br><br>**Size:** n=14 reports	**Inclusion criteria**:<br>• Medline, English-language studies published between January 1984 and June 1994.<br>• Additional references from bibliographies of review articles and original papers.<br>• Studies pertaining to diagnostic performance of duplex or color-guided duplex ultrasonography in PAD of the lower extremities<br>• Contrast angiography was used as the gold standard<br>Significant lesion defined as an arterial diameter reduction on angiography of 50%–100%<br>• The absolute numbers of True-positive, false-negative, true-negative, and false-positive observations were available or derivable.<br><br>**Exclusion criteria:** N/A	**1° endpoint:** N/A<br><br>**Results:**<br>Sensitivity:<br>• 83 (Duplex)<br>• 93 Color guided Duplex<br>Specificity:<br>• 95	N/A
Ota H, et al.<br>2004(78)<br>14684540	**Study type:** Observational<br><br>**Size:** n=27 cases in 24 pts	**Inclusion criteria**:<br>• Sx lower extremity peripheral arterial occlusive disease<br>• Underwent both MDCT angiography and digital subtraction angiography of the aortoiliac and lower extremity arteries<br><br>**Exclusion criteria:** N/A	**1° endpoint:** N/A<br><br>**Results:**<br>Sensitivity:<br>• 99.2<br>Specificity:<br>• 99.1	• MDCT angiography is a reliable method for evaluation the aortoiliac and lower extremity arteries
He C, et al.<br>2014(79)<br>25252783	**Study type:** NR (retrospective cohort study)<br><br>**Size:** n=161 pts	**Inclusion criteria:** Consecutive pts with DM (13 women; mean age, 69.42±11.04 y) and 101 pts without DM (23 women; mean age, 68.50±13.59 y) who underwent DSCT and 320-MDCTA of the arteries in both legs.<br><br>**Exclusion criteria:** Allergy to the iodine	**1° endpoint:** Plaque type, distribution, shape and obstructive natures were compared between pts with and without DM<br><br>**Results:** Total of 2898 vascular segments were included in the analysis. Plaque and stenosis were detected in 681 segments in 60 pts with DM (63.1%) and 854 segments in 101 pts without DM (46.9%;	• DM is associated with a higher incidence of plaque, increased incidence of mixed plaques, moderate stenosis and localization primarily in the distal lower leg segments.<br>• The advanced and noninvasive MDCT could be used for routine preoperative evaluations of LEA.

© American Heart Association, Inc. and American College of Cardiology Foundation

		contrast agent, liver, kidney or HF (Creatinine level ≥120 mol/L), pregnancy and leg amputation. The vascular exclusion criteria included vascular malformations, poor imaging and a lumen diameter <1.5 mm.	p<0.05). Regarding these plaques, pts with DM had a higher incidence of mixed plaques (34.2% vs. 27.1% for pts without DM). An increased moderate stenosis rate and decreased occlusion rate were observed in pts with DM relative to pts without DM (35.8% vs. 28.3%; and 6.6% vs. 11.4%; respectively). In pts with DM, 362 (53.2%) plaques were detected in the distal lower leg segments, whereas in pts without DM, 551 (64.5%) plaques were found in the proximal upper leg segments. The type IV plaque shape, in which the full lumen was involved, was detected more frequently in pts with DM than in pts without DM (13.1% vs. 8.2%).	
Philip F, et al. 2013(80) 23553996	**Study type:** NR (retrospective cohort study)<br><br>**Size:** n=83 pts	**Inclusion criteria:** MDCT and aortography of the pelvic vascularulature prior to consideration for transcatheter aortic valve replacement<br><br>**Exclusion criteria**: N/A	**1° endpoint:** Localize the IPA origin, degree of stenosis (normal: <50% stenosis or abnormal: >50% stenosis or occlusion), normal= and extent of calcification, quantified using a nominal scale (0=no calcification, 1 ≤25%, 2=25%–50%, 3 ≥50% of the IPA length).<br><br>**Results:** In a pt-based analysis, the sensitivity of MDCT for detecting significant proximal IPA disease was 100% and, specificity 74%, positive predictive valve was 66%, and negative predictive value was 100%. In assessing the distal IPA and cavernosal arteries, the sensitivity was 100%, specificity was 64%, positive predictive value 89%, and negative predictive value of 100%. MDCT used significantly more contrast and more radiation than aortography.	• Studies were read independently and blinded
Kayhan A 2012(81) 21345629	**Study type:** NR (prospective)<br><br>**Size:** n=43 pts	**Inclusion criteria:** pts with IC and leg pain, diagnosed as mild PAOD,<br><br>**Exclusion criteria**: N/A	**1° endpoint:** Stenotic lesions<br><br>**Results:** MDCTA detected obstructed or stenotic lesions in 16.8% of arteries, vs. 11.1% compared to DUS. When suprapopliteal arteries alone were considered, MDCTA detected lesions in 15.0% of arteries vs. 11.0% with DUS. When infrapopliteal arteries only were considered, MDCTA detected lesions in 19.6% of arteries, vs. 11.3% with DUS. MDCTA showed 5.7% (95% CI: 3.5%–7.9%) more lesions than DUS when all arteries were considered together, 8.3% (95% CI: 4.6%–12.0%) more lesions	• 40-row MDCTA may be used as a screening tool in pts with mild lower extremity PAOD as it is a noninvasive and more accurate modality when compared to DUS.

© American Heart Association, Inc. and American College of Cardiology Foundation

			when only the infrapopliteal arteries were compared, and 4.0% (95% CI: 1.3%–6.8%) more lesions when only suprapopliteal arteries were compared (p<0.01 for all comparisons).	
Joshi SB, et al. 2009(82) 20083076	**Study type:** NR (retrospective)<br><br>**Size:** n=37 pts	**Inclusion criteria:** Consecutive pts requiring evaluation of aortoiliofemoral anatomy prior to cardiovascular procedures (pts being considered for percutaneous aortic valve intervention.)<br><br>**Exclusion criteria:** N/A	**1° endpoint:** Conventional angiographic and CT images were analyzed independently to assess suitability for large bore (7 mm diameter) intra-arterial catheter access.<br><br>**Results:** Excellent CT image quality was achieved in 34 of 37 pts (92%). The mean contrast dose for CT was 12±2 mL. In 9 pts (24%), CT changed the assessment of femoral access feasibility. Furthermore, in another 7 pts (19%), unfavorable anatomy as shown by CT directed the avoidance of a particular side. Overall, CT findings altered the interventional approach in 16 pts (43%).	• Purpose was to evaluate the feasibility of using ultra-low-dose intra-arterial contrast injection for iliofemoral CT angiography to follow diagnostic cardiac catheterization.<br>• 0 to 15 mL of contrast diluted with normal saline was injected intra-arterially via the pigtail catheter while a spiral CT of the abdomen and pelvis was acquired<br>• There was no significant deterioration detected in renal function after coronary and CT angiography (estimated glomerular filtration rate 54.8±3.8 mL/min before 53.3±3.9 mL/min after, p=0.55).
Mesurolle B, et al. 2004(83) 15246474	**Study type:** NR (prospective)<br><br>**Size:** n=16 pts	**Inclusion criteria:** In the assessment of occlusive arterial disease of abdominal aorta and the lower extremities.<br><br>**Exclusion criteria:** N/A	**1° endpoint:** Sensitivity and specificity vs. catheter angiography<br><br>**Results:** Overall sensitivity of helical CT was 91% and specificity 93%. Segmental analysis found a sensitivity of 43% in infrapopliteal arteries, and a specificity of 86%. elical CT was inconclusive in 6.2% of segments whereas angiography was inconclusive in 5%. Overall sensitivity of helical CT was 91% and specificity 93%. Segmental analysis found a sensitivity of 43% in infrapopliteal arteries, and a specificity of 86%.	• 16 pts underwent both transcatheter angiography and helical CT
Romano M, et al. 2004(84) 15145492	**Study type:** NR (prospective)<br><br>**Size:** n=42 pts	**Inclusion criteria:** Untreated pts with peripheral vascular occlusive disease<br><br>**Exclusion criteria:** Pts with previous radiological interventions or surgery for their peripheral vascular occlusive disease	**1° endpoint:** Sensitivity and specificity of 4 channel MDCTA of the abdominal aorta and lower extremities arteries compared with DSA.<br><br>**Results:** Overall sensitivity and specificity of MDCTA were 93 and 95%, respectively, with positive and negative predictive values of 90 and 97%. Overall diagnostic accuracy was 94%. Normal arterial	N/A

© American Heart Association, Inc. and American College of Cardiology Foundation

			segments and 100% occlusions were correctly identified in all cases by MDCTA. Moderately stenotic segments interpretation in the calves appeared to be more controversial, but no statistical difference in accuracy of MDCTA in the infrapopliteal district arteries was noted with respect to accuracy in the more proximal arterial bed. Good to excellent interobserver and intraobserver agreement were observed, with k values greater than 0.80.	
Martin ML, et al. 2003(85) 12646460	**Study type**: NR (prospective)<br><br>**Size**: n=41 pts	**Inclusion criteria**: Pts referred for DSA of the lower extremities for investigation of sx atherosclerotic disease of the legs<br><br>**Exclusion criteria**: Elevated serum creatinine (>120 micro mol/L) levels, allergy to contrast material, or acute limb-threatening ischemia were excluded. Because pts under- went MDCT angiography and DSA on different days, potential candidates who lived more than 1 H from our hospital were not asked to enroll.	**1° endpoint**: Sensitivity and specificity of MDCT angiography in showing arterial occlusions and stenoses of ≥75%. Intertechnique agreement was measured for each anatomic segment, and interobserver agreement was calculated for both techniques. Agreement was quantified using the kappa statistic.<br><br>**Results:** The sensitivity and specificity of MDCT angiography for depicting arterial occlusions and stenoses of at least 75% were 88.6% and 97.7%, and 92.2% and 96.8%, respectively. Substantial intertechnique agreement (kappa >0.4) was present in 102 (97.1%) of 105 arterial segments. Substantial interobserver agreement was present in 104 (99.0%) of 105 comparisons for both MDCT angiography and DSA with an average kappa value of 0.84 for CT and 0.78 for DSA. MDCT angiography showed more patent segments than DSA (1,192 vs. 1,091). All 9 segments seen on DSA and not seen on MDCT angiography were in the calves. Of 110 segments seen on MDCT angiography and not seen on DSA, 100 (90.9%) were in the calves.	• MDCT angiography was accurate in showing arterial atheroocclusive disease with reliability similar to DSA. MDCT angiography showed more vascular segments than DSA, particularly within calf vessels.
Andreucci M, et al. 2014(86) 24895606	**Study type**: A review of the evidence base for the adverse effects associated with radiographic contrast drugs.<br><br>**Size**: N/A	**Inclusion criteria**: N/A<br><br>**Exclusion criteria**: N/A	**1° endpoint**: N/A<br><br>**Results:**<br>• Monitor renal functions for contrast-induced nephropathy<br>• Nephrotoxic meds should be discontinued before contrast administration<br>• Either nonionic iso-osmolar contrast media or	• Important side effects include hypersensitivity reactions, thyroid dysfunction and contrast-induced nephropathy<br>• The knowledge and screening of side effects can allow appreciation and then prompt management.

© American Heart Association, Inc. and American College of Cardiology Foundation

			nonionic low-osmolar contrast media use to be favored<br>• Lowest dose to be used<br>• Fluid intake to be encouraged.<br>• In high-risk pts N-acetylcysteine may be administered.	
Stacul F, et al. 2011(87)<br>21866433	**Study type**:<br><br>**Size**: N/A	**Inclusion criteria**: N/A<br><br>**Exclusion criteria**: N/A	**1° endpoint**: N/A<br><br>**Results:**<br>• N/A	• Topics reviewed include the definition of CIN, the choice of contrast medium, the prophylactic measures used to reduce the incidence of CIN, and the management of pts receiving metformin

ABI indicates ankle-brachial index; CE-MRA, contrast-enhanced MRA; CI, confidence interval; CT, computed tomography; DM, diabetes mellitus; DSA, digital subtraction angiography; DSCT, dual source computed tomography; DUS, duplex ultrasonography; IC, intermittent claudication; IPA, internal pudendal artery; LEA, lower extremity atherosclerosis; MDCTA, multidetector computed tomography angiography; MDCT, multidetector computed tomography; N/A, not applicable; NR, nonrandomized; NPV, negative predictive value; PAD, peripheral artery disease; PAOD, peripheral arterial occlusive disease; PPV, positive predictive value; pt, patient; and WBMRA, whole-body magnetic resonance angiography.

Evidence Table 7. RCTs of Imaging for Anatomic Assessment (Ultrasound, CTA, MRA, Angiography)–Section 3.3.

Study Acronym; Author; Year Published	Aim of Study; Study Type; Study Size (N)	Patient Population	Study Intervention (# patients) / Study Comparator (# patients)	Endpoint Results (Absolute Event Rates, P value; OR or RR; & 95% CI)	Relevant 2° Endpoint (if any); Study Limitations; Adverse Events
Meyer BC, et al. 2012 (88)<br>22473508	**Aim**: Compare a CB injection protocol using high-iodine concentration contrast medium with a SB injection protocol at equi-iodine doses for run-off CTA.<br><br>**Study type**: prospective RCT<br><br>**Size**: n=83 pts	**Inclusion criteria**: 64 pts with suspected PAD who underwent 40 or 64-slice run-off CTA<br><br>**Exclusion criteria**: N/A	**Intervention**: The CB protocol (32 pts, iomeprol 400mgI/mL, 100 mL, 4 mL/sec)<br><br>**Comparator**: The SB protocol (32 pts, iomeprol 300 mgI/mL, 134 mL, 4 mL/sec).	**1° endpoint**: Luminal CD values were measured and AO was scored (5-point scale). Overall arterial CD was significantly higher with the compact bolus (CB: 279±57HU, SB: 234±32HU, p=0.0017). Segmental CD was significantly higher (p<0.05) in 7 of 16 evaluated segments. Patency-based comparison revealed superior AO in vessels with relevant (50%–99%) stenoses (CB: 4.54 vs. SB: 4.18; p=0.04). Contrast bolus overriding without pathological reasons, i.e., acute occlusions, was noted in 1 pt in each group. Venous overlay was observed less frequently in the CB group (CB vs. SB: 12 vs. 19 pts, NS;	• At equi-iodine doses, the CB protocol led to a quantitatively and qualitatively higher AO compared to the SB protocol. Therefore, a CB protocol should be favored for run-off CTA.

© American Heart Association, Inc. and American College of Cardiology Foundation

				29 of 64 legs [45%] vs. 44 of 64 legs [69%]; p=0.01).	
Fraioli F, et al. 2006(89) 15988586	**Aim:** Compare the influence of radiation dose on image quality and diagnostic accuracy of low dose MDCT with DSA for the detection of aortoiliac and PAD.<br><br>**Study type:** RCT<br><br>**Size:** n=75 pts	**Inclusion criteria:** Onsecutive pts, with a clinical Dx of obstructive arterial disease of the extremities underwent MDCT angiography of the aorta and peripheral vessels.<br><br>**Exclusion criteria:** Renal insufficiency (serum creatinine >2 mg/dl), contra-indication to iodinated contrast, respiratory failure, congestive heart failure and poor general condition of the pt.	**Intervention:** Pt population was randomly divided into three groups of 25 pts. In each group, MDCT scanning parameters were kept constant, except for the mAs.<br><br>**Comparator:** 50 mAs vs. 100 mAs vs. 130 mAs	**1° endpoint:**<br>• The dose reduction was 74% for group A and 40% for group B.<br>• The evaluation of the presence and degree of stenoses revealed a sensitivity, specificity, accuracy, PPV and NPV of 96%, 94%, 95%, 83% and 99% for Group A (50 mAs), 96%, 96%, 96%, 89% and 99% for Group B (100 mAs) and 98%, 96%, 97%, 91% and 100% for the standard dose protocol, Group C (130 mAs).	• Low-dose scanning is thus a feasible and accurate option for 4-row CT angiography of the peripheral vessels.<br>• This technique provides substantial reduction of the radiation dose delivered to the pt while maintaining optimal diagnostic accuracy.
Met R, et al. 2009(90) 19176443	**Aim:** To determine the accuracy of CTA compared with intra-arterial DSA in differentiating extent of disease in pts with PAD<br><br>**Study type:** Meta-analysis<br>CTA vs. DSA<br><br>**Size:** n=909 studies	**Inclusion criteria:**<br>• Reviews of effectiveness for studies comparing CTA with intra-arterial DSA for PAD<br>• Compared multidetector CTA with intra-arterial DSA Included at least 10 pts with IC or CLI<br>• Aimed to detect >50% stenosis or arterial occlusion<br>• Presented either 2 x 2 or 3 x 3 contingency tables (≤50% stenosis vs. >50% stenosis or occlusion), or provided data allowing their construction<br><br>**Exclusion criteria:** N/A	**1° endpoint:** Sensitivity of CTA for detecting PAD (>50% stenosis)<br><br>**Results:** Sensitivity stenosis >50% (95%CI: 92–9); specificity 96%(95% CI: 93–97)	CTA had adequate sensitivity for detecting PAD	N/A
Favaretto E, et al. 2007(91) 17443099	**Aim:** Investigate the agreement between DSA in the diagnosis of stenosis<br><br>**Study type:** Prospective series	**Inclusion criteria:** Lower limb artery disease (claudication, critical ischemia, or skin lesions)<br><br>**Exclusion criteria:** N/A	**1° endpoint:** Diagnostic accuracy of duplex for detected lesion severity of LE PAD<br><br>**Results:** Kappa=0.70; 95% CI: 0.588–0.825 for the whole arterial axis. Agreement was	The sensitivity and specificity of duplex compared to angiography is modest	N/A

© American Heart Association, Inc. and American College of Cardiology Foundation

	Duplex vs. angio<br><br>**Size:** n=49 pts		good for the aorto-iliac district (kappa=0.63) with a sensitivity of 63% and a specificity of 96%, and for the femoro-popliteal district (kappa=0.70) with a sensitivity of 74% and a specificity of 83%. In infrapopliteal arteries, kappa showed a poor agreement.		
Kau T, et al. 2011 (92)<br>21365195	**Aim:** Evaluate the accuracy of DE-CTA maximum intensity projections<br><br>**Study type:** Prospective series DE-CTA vs. angio<br><br>**Size:** n=58	**Inclusion criteria:** Pts with sx peripheral arterial occlusive disease<br><br>**Exclusion criteria:** in ability to get CTA	**1° endpoint:** Diagnostic accuracy of DE-CTA to detect stenosis severity<br><br>**Results:** In DSA, 52.3% of segments were significantly stenosed or occluded. Agreement of DE-CTA MIPs with DSA was good in the aorto-iliac and femoro-popliteal regions (kappa=0.72; kappa=0.66), moderate in the crural region (kappa=0.55), slight in pedal arteries (kappa=0.10) and very good in bypass segments (kappa=0.81). Accuracy was 88%, 78%, 74%, 55% and 82% for the respective territories and moderate (75%) overall, with good sensitivity (84%) and moderate specificity (67%). Sensitivity and specificity was 82% and 76% in claudicants and 84% and 61% in pts with CLI.	DE-CTA had good diagnostic accuracy above the knee. Below the knee the diagnostic accuracy was modest at best and worse when arteries were calcified.	N/A
McCullough PA, 2011(93)<br>21609484	**Aim:** To compare discomfort rates in pt-reported outcomes related to IOCM with LOCM	**Inclusion criteria:** Studies with intra-arterial administration of CM.<br><br>**Exclusion criteria:** Studies with intravenous	**Intervention:** IOCM (Iodixanol) (3,385)<br><br>**Comparator:** LOCM (4,796)	**1° endpoint:**<br>• Pain:<br>Pts receiving IOCM vs. various LOCMs (RD: -0.049; 95% CI: -0.076 – -0.021; p=0.001). IOCM was favored over all LOCMs combined with a summary RD:	• Cold sensation: NS difference<br>• IOCM was found to have less frequent and severe pain and warmth during administration as

© American Heart Association, Inc. and American College of Cardiology Foundation

	**Study type**: Meta-analysis of pooled pt outcomes from 22 RCTs<br><br>**Size**: n=8,087 (discomfort, n=3,567)	administration of contrast media, reviews, meta analyses		-0.188; 95% CI: 0.265 – -0.112; p<0.001) for incidence.<br>• Warmth:<br>IOCM favored over LOCMs, RD: -0.043; 95% CI: -0.074 – -0.011; p=0.008)	compared to LOCM

AO indicates Arterial opacification; CB, compact bolus; CD, contrast density; CI, confidence interval; CLI, critical limb ischemia; CTA, computed tomographic angiography; CT, computed tomography; DE-CTA, dual-energy computed tomographic angiography; DSA, digital subtraction angiography; IC, intermittent claudication; IOCM, iso-osmolar contrast media; LOCM, low-osmolar contrast media; mAs, milliamperage second value; MDCT, multiple detector computed tomography; MIPs, maximum intensity projections; NS, not significant; pt, patient; RD, risk difference; and SB, standard bolus.

Evidence Table 8. Nonrandomized Trials, Observational Studies, and/or Registries for Abdominal Aortic Aneurysm–Section 4.1.

Study Acronym; Author; Year Published	Study Type/Design; Study Size	Patient Population	Primary Endpoint and Results (include P value; OR or RR; & 95% CI)	Summary/Conclusion Comment(s)
Sultan S, et al. 2013(94)<br>23711680	**Study type**: Cross-sectional single-center study<br><br>**Size**: 328 pts having a vascular intervention for PAD, AAA, or carotid disease	**Inclusion criteria**: Intervention for 1 of the PVD territories. Poly vascular disease defined as disease in ≥2 territories.<br><br>**Exclusion criteria**: N/A	**1° endpoint**: Prevalence of AAA, CAD, and carotid disease in PAD pts receiving revascularization<br><br>**Results**: Poly-vascular bed pts had about 8X the risk of carotid disease or AAA.	• Looks at the risk according to multiple vascular beds not just PAD<br>• Can't discern the risk of AAA or CVD with PAD alone
Kurvers HA, et al. 2003(95)<br>12764269	**Study type**: Cross-sectional single center study<br><br>**Size**: n=2,274 vascular pts	**Inclusion criteria**: Enrolled in SMART study referred to a vascular center with sx peripheral atherosclerosis in some arterial territory or elevated risk factors (e.g. DM)<br><br>**Exclusion criteria**: N/A	**1° endpoint**: Prevalence of AAA >3cm diameter<br><br>**Results**: Prevalence 6.5% in PAD pts vs. ~1% for risk factor only pts. Age >54 y and PAD increased prevalence to 9.6%. Prevalence of AAA >5cm low in all groups	• Select sx atherosclerosis population
Grøndal N, et al. 2015(8)<br>25923784	**Study type**: Danish intervention arm of screening trial<br><br>**Size**: n=25,083 men who were screened for AAA.	**Inclusion criteria**: Men age 65–74 y who were screened for AAA.<br><br>**Exclusion criteria**: N/A	**1° endpoint**: Prevalence of PAD in pts screened for AAA.<br><br>**Results**: AAA was diagnosed in 3.3% and PAD in 10.9%.	• The prevalence of AAA has declined in the past decade from 4.0% to 3.3%.<br>• 10.9% of men undergoing screening for AAA also had PAD.

© American Heart Association, Inc. and American College of Cardiology Foundation

	1,8749 attended the screening (uptake 74.7%).			
Giugliano G, et al. 2012(96) 23173942	**Study type:** Prospective case series<br><br>**Size:** n=213 consecutive pts	**Inclusion criteria:** 213 consecutive pts with PAD screened for AAA<br><br>**Exclusion criteria:** N/A	**1° endpoint:** Prevalence of AAA in pts with PAD<br><br>**Results:** AAA was present in 19 pts (9%) with similar prevalence in men and women.	• Small study showed that prevalence of AAA in pts with PAD is much higher than in the general population.<br>• Prevalence related to age:<br><55 y: 0<br>55-64 y: 5.1%<br>65-74 y: 11.4%<br>>75 y: 15.8%
Barba A 2005(97) 15963741	**Study type:** Observational descriptive study<br><br>**Size:** n=1,166 pts with PAD	**Inclusion criteria:** 1,166 consecutive pts with PAD had AAA screening<br><br>**Exclusion criteria:** None	**1° endpoint:** Prevalence of AAA in pts with PAD<br><br>**Results:** Prevalence of AAA in men was 13.6% and in women 4.1% but there were only 73 women.	• Prevalence of AAA in pts with PAD is higher than in the general population.<br>• As in other studies, the prevalence of AAA in pts with PAD increased with age.<br>• The prevalence was much higher in men than women.

AAA indicates abdominal aortic aneurysm; CAD, coronary artery disease; CVD, cardiovascular disease; N/A, not applicable; PAD, peripheral artery disease; and PVD, peripheral vascular disease.

Evidence Table 9. Nonrandomized Trials, Observational Studies, and/or Registries of Coronary Artery Disease Screening in PAD–Section 4.2.

Study Acronym; Author; Year Published	Study Type/Design; Study Size	Patient Population	Primary Endpoint and Results (include P value; OR or RR; & 95% CI)	Summary/Conclusion Comment(s)
Lee JY, et al. 2013(98) 24355120	**Study type:** Cohort<br><br>**Size:** n=2,424 pts with CAD and 119 pts without significant CAD on cath	**Inclusion criteria:** Pts having coronary angiography<br><br>**Exclusion criteria:** Pts with known PAD or prior ABI	**1° endpoint:** Prevalence of abnormal ABI <0.9 or >1.4 and MACE over 3 y.<br><br>**Results:**<br>• In CAD pts: 14% had ABI <0.9, vs. 4% in pts without CAD. Of the 390 pts with abnormal ABI, 130 (33%) had coronary revascularization at time of cath. 3 y MACE significantly higher with abnormal ABI (15.7% vs. 3.3%; p<0.001).<br>• Abnormal ABI HR: 1.87 or 2.40 on propensity matched analysis.	• Doesn't really say the prevalence of CAD in all pts with abnormal PAD. It looks at a select group who had cath and then looks at the impact of PAD on outcomes over 3 y.<br>• Shows prognostic value of low ABI for MACE but does not provide information on the value of screening for CAD in pts with low ABI
Moyer VA and U.S. Preventative Services Task Force	**Study type:** Review of studies assessing ABI and CAD	**Inclusion criteria:** All studies examining the prognostic value of	**1° endpoint:** N/A<br><br>**Results:** See box to right. More useful for	• USPSTF summary statement concluding that screening for PAD using the ABI in asx individuals is not of benefit.

© American Heart Association, Inc. and American College of Cardiology Foundation

2013(99) 24026320	**Size:** N/A	screening ABI in asx pts.<br><br>**Exclusion criteria:** N/A	question addressing asx screening with an ABI	• They find several studies showing a relationship of low ABI to CAD events, but that the NRI is often not reported or indicates a change that may not be clinically significant<br>• This is more useful for the assessment of the value of screening ABI in asx individuals
Lin JS, et al. 2013(30) 24156115	**Study type:** Review of studies assessing value of ABI in addition to Framingham risk score.<br><br>**Size:** n=52,510	**Inclusion criteria:** Studies assessing the value of ABI as a predictor of CAD events<br><br>**Exclusion criteria:** N/A	**1° endpoint:** Test characteristics and NRI<br><br>**Results:** NRI small when adding ABI to FRS	• USPSTF analysis supporting the summary statement above (99)<br><br>• NRI small when adding ABI to FRS<br>• This is more useful for the assessment of the value of screening ABI in asx individuals

ABI indicates ankle-brachial index; asx, asymptomatic; CAD, coronary artery disease; CTA, computed tomographic angiography; CT, computed tomography; FRS, Framingham risk score; HR, hazard ratio; MACE, major adverse cardiovascular events; N/A, not applicable; NRI, net reclassification improvement; PAD, peripheral artery disease, pt, patient; and USPSTF, United States Preventative Services Task Force.

Evidence Table 10. RCTs for CAD Screening in PAD–Section 4.2.

Study Acronym; Author; Year Published	Aim of Study; Study Type; Study Size (N)	Patient Population	Study Intervention (# patients) / Study Comparator (# patients)	Endpoint Results (Absolute Event Rates, P value; OR or RR; & 95% CI)	Relevant 2° Endpoint (if any); Study Limitations; Adverse Events
McFalls EO, et al. 2004(100) 15625331	**Study type:** RCT of cardiac catheterization and coronary revascularization for CAD in high-risk pts scheduled for vascular surgery<br><br>**Size:** n=5,859 pts	**Inclusion criteria:** Pts scheduled for major vascular surgery (AAA repair or lower extremity operation) who were considered at increased risk of cardiovascular events according to a risk score and the myocardial ischemia on noninvasive testing<br><br>**Exclusion criteria:** Left main stenosis >50%, LVEF <20%, severe aortic stenosis	**Intervention:** Revascularization before elective major vascular surgery<br><br>**Comparator:** No revascularization before elective major vascular surgery	**1° endpoint:** Long-term mortality<br><br>**Results:** No difference in outcomes. Mortality at 2.7 y was 22% in the no-CAD revascularization group and 23% in the CAD revascularization group. 30 d postoperative MI=12% in the CAD revascularization group and 14% in the no-CAD revascularization group.	• No difference in 30 d postoperative MI=12% in the CAD revascularization group and 14% in the no-CAD revascularization group.<br>• Excludes left main disease<br>• No advantage to screening for CAD in pts having elective major vascular surgery on mortality or perioperative rates of MI.

AAA indicates abdominal aortic aneurysm; CAD, coronary artery disease; LVEF, left ventricular ejection fraction; MI, myocardial infarction; pt, patient; and RCT, randomized controlled trial.

Evidence Table 11. Nonrandomized Trials, Observational Studies, and/or Registries of Screening in Carotid Artery Disease–Section 4.3.

Study Acronym; Author;	Study Type/Design; Study Size	Patient Population	Primary Endpoint and Results (include P value; OR or RR;	Summary/Conclusion Comment(s)

© American Heart Association, Inc. and American College of Cardiology Foundation

Year Published			& 95% CI)	
Sultan S, et al.<br>2013(94)<br>23711680	**Study type**: Cross-sectional single-center study<br><br>**Size**: n=328 pts having a vascular intervention for PAD, AAA, or carotid disease	**Inclusion criteria**: Intervention for 1 of the PVD territories. Poly vascular disease defined as disease in ≥2 territories.<br><br>**Exclusion criteria**: N/A	**1° endpoint**: Prevalence of AAA, CAD, and carotid disease in PAD pts receiving revascularization<br><br>**Results:** Poly-vascular bed pts had about 8X the risk of carotid disease or AAA.	• Looks at the risk according to multiple vascular beds not just PAD<br>• Can't discern the risk of AAA or CVD with PAD alone
Kurvers HA, et al.<br>2003(95)<br>12764269	**Study type**: Cross-sectional single center study<br><br>**Size**: n=2,274 vascular pts	**Inclusion criteria**: Enrolled in SMART study referred to a vascular center with sx peripheral atherosclerosis in some arterial territory or elevated risk factors (e.g. DM)<br><br>**Exclusion criteria**: N/A	**1° endpoint**: Prevalence of carotid stenosis<br><br>**Results:** Prevalence 12.5% in PAD pts vs. ~2% for risk factor only pts. Age >54 y and PAD increased prevalence to 22%.	• Select sx atherosclerosis population

AAA indicates abdominal aortic aneurysm; ABI, ankle-brachial index; CAD, coronary artery disease; CVD, cardiovascular disease; DM, diabetes mellitus; PAD, peripheral artery disease; pt, patient; PVD, peripheral vascular disease; sx, symp.

Evidence Table 12. Nonrandomized Trials, Observational Studies, and/or Registries for Renal Artery Disease–Section 4.4.

Study Acronym; Author; Year Published	Study Type/Design; Study Size	Patient Population	Primary Endpoint and Results (include P value; OR or RR; & 95% CI)	Summary/Conclusion Comment(s)
Olin JW, et al.<br>1990(101)<br>2368764	**Study type**: Single center, retrospective cohort study<br><br>**Size**: n=395 consecutive pts	**Inclusion criteria**: Pts who underwent catheter angiography for evaluation of AAA, Aortoiliac Occlusive Disease and PAD.<br><br>**Exclusion criteria**: N/A	**1° endpoint**: Prevalence of >50% renal artery stenosis<br><br>**Results**: Prevalence was 38% in pts with AAA, 33% with AOD and 39% with PAD.	• There is a high prevalence of incidental renal artery stenosis in pts with atherosclerosis in other locations, even in the absence of clinical clues to suspect RAS.
Leertouwer TC, et al.<br>2001 (102)<br>11260411	**Study type**: Single center, retrospective cohort study<br><br>**Size**: n=386 consecutive pts	**Inclusion criteria**: Pts who underwent catheter based angiography for evaluation of PAD<br><br>**Exclusion criteria**: N/A	**1° endpoint**: Prevalence of >50% renal artery stenosis<br><br>**Results**: 126 (33%) had >50% stenosis.	• Incidental renal artery stenosis is common in pts with PAD<br>• Renal replacement therapy did not occur in any of these pts thus revascularization to prevent ESRD is not indicated in most pts.
**CHS**<br>Hansen KJ, et al.<br>2002(103)	**Study Type**: Multicenter, longitudinal cohort study	**Inclusion criteria**: Free living pts age >65 y were invited to undergo renal artery duplex	**1° endpoint**:<br>Prevalence of RAS in a free standing elderly population	• This is the 1st population based estimate of the prevalence of RVD among free living, elderly black and

© American Heart Association, Inc. and American College of Cardiology Foundation

12218965	**Size:** n=870 pts	ultrasound<br><br>**Exclusion criteria:** N/A	**Results:**<br>• 834 (96%) were technically adequate to define the presence or absence of RVD<br>• Prevalence of RAS was 6.8%.<br>• No difference in prevalence between white and black pts.	white Americans

AAA indicates; AOD, arterial occlusive disease; ESRD, end-stage renal disease; N/A, not applicable; PAD, peripheral artery disease; pt, patient; RAS, renal artery stenosis; and RVD, renal vascular disease.

Evidence Table 13. RCTs Evaluating Antiplatelet Agents– Section 5.1.

Study Acronym; Author; Year Published	Aim of Study; Study Type; Study Size (N)	Patient Population	Study Intervention (# patients) / Study Comparator (# patients)	Endpoint Results (Absolute Event Rates, P value; OR or RR; & 95% CI)	Relevant 2° Endpoint (if any); Study Limitations; Adverse Events
**POPADAD**<br>Belch J, et al. 2008(16)<br>18927173	**Aim**: To determine whether ASA and antioxidant therapy, combined or alone, are more effective than placebo in reducing the development of cardiovascular events in pts with DM and asx PAD.<br><br>**Study type:** Multicenter, randomized, double blind, 2×2 factorial, placebo controlled trial.<br><br>**Size:** n=1,276 pts	**Inclusion criteria**: Aged ≥40 y with type 1 or type 2 DM and an ABI of ≤0.99 but no sx cardiovascular disease<br><br>**Exclusion criteria**: People with evidence of sx CV disease; those who use ASA or antioxidant therapy on a regular basis; those with peptic ulceration, severe dyspepsia, a bleeding disorder, or intolerance to ASA; those with suspected serious physical illness (such as cancer), which might have been expected to curtail life expectancy; those with psychiatric illness (reported by their GP); those with congenital heart disease; and those unable to give informed consent	**Intervention and comparator**: Daily, 100 mg ASA tablet + antioxidant capsule (n=320), ASA tablet + placebo capsule (n=318), placebo tablet + antioxidant capsule (n=320), or placebo tablet + placebo capsule (n=318)	**1° endpoint**:<br>• Death from coronary heart disease or stroke, nonfatal MI or stroke, or amputation above the ankle for CLI; and death from CHD or stroke<br>• 116 of 638 primary events occurred in the ASA groups compared with 117 of 638 in the no ASA groups (18.2% vs. 18.3%) HR: 0.98; 95% CI: 0.76–1.26. 43 deaths from coronary heart disease or stroke occurred in the ASA groups compared with 35 in the no ASA groups (6.7% vs. 5.5%): HR: 1.23; 95% CI: 0.79–1.93).<br>• No difference in treatment for ABI <0.90	**Adverse effect (effect estimates):**<br>• Malignancy 0.76 (0.52–1.11),<br>• Gastrointestinal bleeding, 0.90 (0.53–1.52)<br>• Dyspepsia 0.77 (0.55–1.08),<br>• Allergy 1.14 (0.80–1.63)

© American Heart Association, Inc. and American College of Cardiology Foundation

Fowkes FG, et al.<br>2010(15)<br>20197530	**Aim:** To determine the effectiveness of ASA in preventing events in people with a low ABI identified on screening the general population.<br><br>**Study type**:<br>Randomized Controlled Trial<br><br>**Size:** n=3,350 pts	**Inclusion criteria:**<br>Age 50 to 75 with no Hx of vascular disease and ABI <0.95<br>**Exclusion criteria:**<br>Hx of MI, stroke, angina, or PAD; currently used ASA, other antiplatelet or anticoagulant agents; had severe indigestion; had chronic liver or kidney disease; were receiving chemotherapy; had contraindications to ASA; and had an abnormally high or low hematocrit value (measured after the screening)	**Intervention:** 100 mg enteric coated ASA<br><br>**Comparator:**<br>Placebo	**1° endpoint**: Composite of initial (earliest) fatal or nonfatal coronary event or stroke or revascularization<br>No statistically significant difference was found between groups (13.7 events per 1000 person-years in the ASA group vs. 13.3 in the placebo group; HR: 1.03; 95% CI; 0.84–1.27)<br><br>**Safety endpoint:**<br>• Major hemorrhage<br>• Initial event of major hemorrhage requiring admission to hospital occurred in 34 pts (2.5 per 1000 person-years) in the ASA group and 20 (1.5 per 1000 person-years) in the placebo group (HR: 1.71; 95% CI: 0.99–2.97).	• All initial vascular events, defined as a composite of a primary endpoint event or angina, IC or transient ischemic attack; no statistically significant difference between groups (22.8 events per 1000 person-years in the ASA group vs. 22.9 in the placebo group; HR: 1.00; 95% CI: 0.85–1.17)<br>• All-cause mortality no significant difference in all-cause mortality between groups (176 vs. 186 deaths, respectively; HR: 0.95; 95% CI: 0.77–1.16)
**CLIPS**<br>Catalano M, et al.<br>2007(104)<br>17305650	**Aim:** To assess the prophylactic efficacy of ASA and a high-dose antioxidant vitamin combination in pts with PAD in terms of reduction of the risk of a first vascular event (MI, stroke, vascular death) and CLI.<br><br>**Study type:**<br>Randomized, placebo-controlled, double-blind clinical trial with 2x2 factorial designs.<br><br>**Size:** n=366 pts	**Inclusion criteria:** stage I–II PAD documented by angiography or ultrasound, with ankle/brachial index <0.85 or toe index <0.6<br><br>**Exclusion criteria:**<br>• Fontaine stage III or IV PVD; life<br>• Expectancy <24 mo; vascular surgery or angioplasty in the last 3 mo;<br>• Pregnancy or lactation;<br>• Contraindication to ASA;<br>• Major cardiovascular events requiring antiplatelet therapy;<br>• Participation in another clinical trial;<br>• Uncooperative pts;<br>• Treatment with drugs that interfere with hemostasis, such as anticoagulants, antiplatelet agents and prostanoids, peripheral vasodilators, ASA and/or	**Intervention and Comparator:** Oral ASA (100 mg daily), oral antioxidant vitamins (600 mg vitamin E, 250 mg vitamin C and 20 mg beta-carotene daily), both or neither	**1° endpoints:**<br>• Incidence of fatal and nonfatal vascular events (MI, stroke and pulmonary embolism) and critical leg ischemia<br>• 7 of 185 ASA and 20 of 181 placebo pts suffered a major vascular event (risk reduction 64%, p=0.022)<br>• 5 ASA and 8 placebo pts, respectively, suffered critical leg ischemia (total 12 vs. 28, p=0.014)<br><br>**Safety endpoint:** Incidence of bleeding 4 in ASA and 0 in placebo (p=0.99)	• 76% with type 2 DM

© American Heart Association, Inc. and American College of Cardiology Foundation

		supplementary vitamins that could not be discontinued or had to be started.			
Horrocks M, et al. 1997(105) 9257670	**Aim:** To investigate the effects of 2 platelet inhibitors, ASA and iloprost, on platelet uptake and restenosis at the site of angioplasty in pts undergoing femoral or popliteal angioplasty.<br><br>**Study type:** Prospective, randomized<br><br>**Size:** n=43 pts	**Inclusion criteria:** Pts undergoing femoral or popliteal angioplasty<br><br>**Exclusion criteria:** Bleeding disorder, ulcer disease	**Intervention:** ASA (300 mg/d), iloprost (8 H/d IV infusion) or no antiplatelet medication during angioplasty and on the subsequent 2 d.	**1° endpoint:**<br>• Platelet uptake was measured using 111 Indium-labelled platelets. Restenosis was assessed by repeat angiography at 3 mo and clinical symptoms up to 12 mo.<br>• Median changes in platelet uptake were similar in the 3 treatment groups, but all platelet radioactivity ratios >2.0 occurred in the control group. Restenosis at 3 mo was observed in 3 control, 5 ASA and 1 iloprost pt.<br>• Further surgical intervention was performed in 3 control and 3 ASA pts, but in none of the iloprost pts up to 12 mo after angioplasty	• Limited utility as iloprost also utilized
Minar E, et al. 1995(106) 7697845	**Aim:** To compare the effects of high-dose (1000 mg/d) and low-dose (100 mg/d) ASA on long-term patency after femoropopliteal angioplasty.<br><br>**Study type:** Randomized<br><br>**Size:** n=216 pts	**Inclusion criteria:** Pts treated successfully by percutaneous transluminal angioplasty for femoropopliteal lesions<br><br>**Exclusion criteria**: Failed PTA, recent gastroduodenal ulcer, life expectancy <2 y, severe renal insufficiency, need for ongoing nonsteroidal, unable to consent	**Intervention and Comparator**: 1000 or 100 mg ASA daily.	**1° endpoint:** Long-term (24 mo) patency 36 pts in the high-dose and 36 in the low-dose ASA group, developed angiographically verified reobstruction within the recanalized segment. By intention-to-treat analysis, the cumulative patency rates at 24 mo were 62.5% in the high-dose and 62.6% in the low-dose ASA group (Wilcoxon, p=0.97; log-rank, p=0.97). The cumulative survival at 24 mo of follow-up was 86.6% in the high-dose and 87.7% in the low-dose ASA group.<br><br>**Safety endpoint:** Discontinued therapy for gastrointestinal symptoms, 4 in high dose and 0 in low dose<br>Discontinued therapy 30 high dose and 11 low dose (p<0.01)	• 100 mg as effective as 1000 mg<br>• Treatment started 3 d after PTA

© American Heart Association, Inc. and American College of Cardiology Foundation

**CAPRIE** 1996 (107) 8918275	**Aim**: To assess the relative efficacy of clopidogrel (75 mg once daily) and ASA (325 mg once daily) in reducing the risk of a composite outcome cluster of ischemic stroke, MI, or vascular death<br><br>**Study type**: Randomized, blinded<br><br>**Size**: n=19,185 pts	**Inclusion criteria**: Pts with atherosclerotic vascular disease manifested as either recent ischemic stroke, recent MI, or sx PAD<br><br>**Exclusion criteria**:<br>• Age <21 y<br>• Severe cerebral deficit likely to lead to pt being bedridden or demented Carotid endarterectomy after qualifying stroke<br>• Qualifying stroke induced by carotid endarterectomy or angiography<br>• Pt unlikely to be discharged alone after qualifying event<br>• Severe comorbidity likely to limit pt's life expectancy to less than 3 y Uncontrolled hypertension<br>• Scheduled for major surgery<br>• Contraindications to study drugs:<br>• Severe renal or hepatic insufficiency<br>• Hemostatic disorder or systemic bleeding<br>• Hx of haemostatic disorder or systemic bleeding<br>• Hx of thrombocytopenia or neutropenia<br>• Hx of drug-induced hematologic or hepatic abnormalities<br>• Known to have abnormal WBC, differential, or platelet count<br>• Anticipated requirement for long-term anticoagulants, non-study antiplatelet drugs or NSAIDs affecting platelet function<br>• Hx of ASA sensitivity<br>Women of childbearing age not	**Intervention**: Clopidigrel 75 mg per d<br><br>**Comparator**: ASA 325 mg per d	**1° endpoint**:<br>• Composite outcome cluster of ischemic stroke, MI, or vascular death<br>• 1960 first events included in the outcome cluster on which an intention-to-treat analysis showed that pts treated with clopidogrel had an annual 5.32% risk of ischemic stroke, MI, or vascular death lower than 5.83% with ASA (p=0.043). A relative-risk reduction of 8.7% in favor of clopidogrel (95% CI: 0.3–16.5)<br><br>**Safety endpoint**: Bleeding similar in the 2 groups	• Reported adverse experiences in the clopidogrel and ASA groups judged to be severe included rash (0.26% vs. 0.10%), diarrhea (0.23% vs. 0.11%), upper gastrointestinal discomfort (0.97% vs. 1.22%), intracranial hemorrhage (0.33% vs. 0·47%), and gastrointestinal hemorrhage (0.52% vs. 0.72%), respectively. There were 10 (0.10%) pts in the clopidogrel group with significant reductions in neutrophils (<1.2 × 10^9/L) and 16 (0.17%) in the ASA group.<br>• Marginally statistically significant result (p=0.043) was observed for the primary endpoint, with statistical heterogeneity of treatment effect (p=0.042) being observed between the 3 predefined subgroups of pts with recent stroke, MI, or PVD. Only the PVD subgroup clearly benefited from clopidogrel over ASA the use of clopidogrel vs. ASA.

© American Heart Association, Inc. and American College of Cardiology Foundation

		using reliable contraception<br>Currently receiving investigation drug<br>• Previously entered in other clopidogrel studies<br>Geographic or other factors making study participation impractical			
**CHARISMA**<br>Cacoub PP, et al.<br>2009(108)<br>19136484	**Aim:** To determine whether clopidogrel + ASA provides greater protection against major cardiovascular events than ASA alone in pts with PAD.<br><br>**Study type:**<br>Substudy of Bhatt et al., 2007.<br>Post hoc analysis of pt subgroup from a larger randomized trial<br><br>**Size:** n=3,096 pts	**Inclusion criteria:** Sx (2,838) current IC together with an ABI ≤0.85, or a Hx of IC together with a previous related intervention (amputation, surgical or catheter-based peripheral revascularization) or asx (258) PAD ABI, 0.90 were identified among those with multiple risk factors<br><br>**Exclusion criteria:** Taking oral antithrombotic medications or NSAIDs on a long-term basis (although cyclooxygenase-2 inhibitors were permitted). Pts were also excluded if, in the judgment of the investigator, they had established indications for clopidogrel therapy (such as a recent acute coronary syndrome). Pts who were scheduled to undergo a revascularization were not allowed to enroll until the procedure had been completed; such pts were excluded if they were considered to require clopidogrel after revascularization.	**Intervention:**<br>Clopidogrel + ASA<br><br>**Comparator:**<br>Placebo + ASA	**1° endpoint:** Among the pts with PAD, the primary endpoint occurred in 7.6% in the clopidogrel + ASA group and 8.9% in the placebo + ASA group (HR: 0.85; 95% CI: 0.66–1.08; p=0.18). In these pts, the rate of MI was lower in the dual antiplatelet arm than the ASA alone arm: 2.3% vs. 3.7% (HR: 0.63; 95% CI: 0.42–0.96; p=0.029), as was the rate of hospitalization for ischemic events: 16.5% vs. 20.1% (HR: 0.81; 95% CI: 0.68–0.95; p=0.011).<br><br>**Safety endpoint:** The rates of severe, fatal, or moderate bleeding did not differ between the groups, whereas minor bleeding was increased with clopidogrel: 34.4% vs. 20.8% (OR: 1.99; 95% CI: 1.69–2.34; p<0.001)	• Positive subgroups within negative trials are often the result of confounding or bias, especially post-hoc defined subgroups.<br>•The rate of the primary safety endpoint (severe bleeding) was 1.7% in each treatment group (p ¼ 0.90).
**CHARISMA**<br>Bhatt DL, et al.<br>2007(109)<br>17498584	**Aim:** To determine whether there is benefit of clopidoprel + ASA in a subpopulation of CHARISMA	**Inclusion criteria:** "CAPRIE-like" if they were enrolled with a documented prior MI, documented prior ischemic stroke, or sx PAD<br><br>**Exclusion criteria:**	**Intervention:**<br>Clopidogrel + ASA<br><br>**Comparator:**<br>Placebo + ASA	**1° endpoint:** The rate of cardiovascular death, MI, or stroke was significantly lower in the clopidogrel + ASA arm than in the placebo + ASA arm: 7.3% vs. 8.8% (HR 0.83; 95% CI: 0.72–0.96; p=0.01)	• Positive subgroups within negative trials are often the result of confounding or bias, especially post hoc defined subgroups<br>• Hospitalizations for

© American Heart Association, Inc. and American College of Cardiology Foundation

	(Clopidogrel for High Atherothrombotic Risk and Ischemic Stabilization, Management, and Avoidance) trial, where no statistically significant benefit was found in the overall broad population of stable pts studied.<br><br>**Study type:** Post hoc analysis of pt subgroup from a larger randomized trial<br><br>**Size:** n=9,478 pts	• Taking oral antithrombotic medications or NSAIDs on a long-term basis (although cyclooxygenase-2 inhibitors were permitted).<br>• In the judgment of the investigator, pts had established indications for clopidogrel therapy (such as a recent acute coronary syndrome).<br>• Pts who were scheduled to undergo a revascularization were not allowed to enroll until the procedure had been completed; such pts were excluded if they were considered to require clopidogrel after revascularization.		**Safety endpoint:**<br>• Moderate bleeding was significantly in-creased: 2.0% vs. 1.3% (HR: 1.60; 95% CI: 1.16–2.20, p=0.004).<br>• No significant difference in the rate of severe bleeding: 1.7% vs. 1.5% (HR: 1.12; 95% CI: 0.81–1.53; p=0.50)	ischemia were significantly decreased in the clopidogrel group, 11.4% vs. 13.2% (HR: 0.86; 95% CI: 0.76–0.96; p=0.008)
**CHARISMA**<br>Berger PB, et al. 2010(110)<br>20516378	**Aim:** To determine the frequency and time course of bleeding with DAPT in pts with established vascular disease or risk factors only; identify correlates of bleeding; and determine whether bleeding is associated with mortality.<br><br>**Study type:** Post hoc analysis of double-blind, placebo-controlled, randomized trial	**Inclusion criteria:** Pts had either established stable vascular disease or multiple risk factors for vascular disease without established disease<br><br>**Exclusion criteria:**<br>• Taking oral antithrombotic medications or NSAIDs on a long-term basis (although cyclooxygenase-2 inhibitors were permitted).<br>• In the judgment of the investigator, pts had established indications for clopidogrel therapy (such as a recent acute coronary syndrome).<br>• Pts who were scheduled to undergo a revascularization were not allowed to enroll until the	**Intervention:** Clopdiogrel + ASA<br><br>**Comparator:** Placebo + ASA	**1° endpoint:**<br>• Bleeding was assessed with the use of the Global Utilization of Streptokinase and t-PA for Occluded Coronary Arteries (GUSTO) criteria.<br>• Severe bleeding occurred in 1.7% of the clopidogrel group vs. 1.3% on placebo (p=0.087); moderate bleeding occurred in 2.1% vs. 1.3%, respectively (p<0.001).<br>• Moderate bleeding was strongly associated with increased mortality on multivariable analysis<br>(HR: 2.55; 95% CI: 1.71–3.80; p<0.0001)	• ASA 75 mg to 162 mg

© American Heart Association, Inc. and American College of Cardiology Foundation

	**Size**: n=15,603 pts	procedure had been completed; such pts were excluded if they were considered to require clopidogrel after revascularization.			
Cassar K, et al. 2005(111) 15609386	**Aim**: To investigate the antiplatelet effect of a combination of ASA and clopidogrel compared with ASA alone in pts with claudication undergoing endovascular revascularization<br><br>**Study type**: Double-blind randomized placebo-controlled<br><br>**Size**: n=132 pts	**Inclusion criteria**:<br>• Pts undergoing lower limb angioplasty<br>• Hemoglobin >10 g/L<br>• Platelet count >150 × 10^9 g/L<br>• Aspartate aminotransferase, alkaline phosphatase, γ-glutamyltransferase <3 times upper normal limit<br>• Creatinine <2 times upper normal limit<br>• Body mass index <33<br>• Age 18–80 y<br>• No contraindication to either ASA or clopidogrel<br><br>**Exclusion criteria**:<br>• Hx of hematological malignancy<br>• Acute illness within 14 d of randomization<br>• Transfusion of whole blood or red cells within 14 d or randomization Known or suspected drug or alcohol abuse On steroids On warfarin or heparin Hx of bleeding diathesis or coagulopathy Hx of severe neutropenia (neutrophil count <1.8 × 10^9LI) Hx of thrombocytopenia (platelet count <150 × 10^9/L)	**Intervention**: Clopidogrel 75 mg and ASA 75 mg<br><br>**Comparator**: Placebo and ASA 75 mg	**1° endpoint**: Flow cytometric measurements of platelet fibrinogen binding and P-selectin expression were taken as measures of platelet function at baseline, 12 h after the loading dose, and 1 h, 24 h and 30 d after intervention. Within 12 h of the loading dose, platelet activation in the clopidogrel group had decreased (P-selectin by 27.3%, p=0.017; fibrinogen binding by 34.7%, p=0.024; stimulated fibrinogen binding by 49.2%, p<0.001). No change was observed in the placebo group. Platelet function in the clopidogrel group was significantly suppressed compared with baseline at 1 hr, 24 hr and 30 d after endovascular intervention (stimulated fibrinogen binding by 53.9%, 51.7%, and 57.2% respectively; all p<0.001).<br><br>**Safety endpoint**: 2 pts in each group developed a skin rash and 2 in each group developed a hematoma at the site of radiological access that did not require intervention. The number of pts who developed bruising at and around the site of access was slightly higher in the clopidogrel group (25 vs. 16) but the difference between the 2 groups was not statistically significant. 2 pts in the clopidogrel group had an ischemic stroke at d 7 and d 12 after angioplasty. 1 of these pts, however, had stopped taking all medication immediately after intervention. Another pt developed melena secondary to bleeding from multiple small gastric ulcers. Further investigation revealed that the pt had metastatic colonic cancer. 1 pt in the clopidogrel group became hypotensive	• Limited to post PTA platelet function

© American Heart Association, Inc. and American College of Cardiology Foundation

				immediately after intervention and was found to have a retroperitoneal hematoma. This resulted in a delay in discharge from hospital of 7 d but no surgical intervention was necessary	
**CASPAR**<br>BelchJJ, et al. 2010(112)<br>20678878	**Aim:** To determine whether clopidogrel + ASA conferred benefit on limb outcomes over ASA alone in pts undergoing below-knee bypass grafting<br><br>**Study type:** Prospective, multicenter, randomized, double-blind, placebo-controlled<br><br>**Size:** n=851 pts	**Inclusion criteria:** Pts undergoing vascular grafting as a treatment for PAD were eligible for recruitment to the trial 2–4 d after bypass surgery. Between 40–80 yr .<br><br>**Exclusion criteria:**<br>• Onset of PAD symptoms before the age of 40 y;<br>• Nonatherosclerotic vascular disease;<br>• Pts receiving aortobifemoral, iliac-femoral, or crossover (femoral-femoral) grafts, or undergoing peripheral transcutaneous angioplasty during the same surgery;<br>• Significant bleeding risk, such as current active bleeding at the surgical site;<br>• Withdrawal of an epidural catheter less than 12 hr before randomization;<br>• Peptic ulceration within 12 mo of randomization;<br>• Previous or current intracranial hemorrhage or hemorrhagic stroke;<br>• Any Hx of severe spontaneous bleeding;<br>• Current warfarin therapy or anticipated need for warfarin;<br>• Concomitant additional antiplatelet agents or thrombolytic agents	**Intervention:** Clopidogrel 75 mg/d + ASA 75 to 100 mg/d<br><br>**Comparator:** Placebo + ASA 75 to 100 mg/d	**1° endpoint:**<br>• Composite of index-graft occlusion or revascularization, above-ankle amputation of the affected limb, or death<br>• In the overall population, the primary endpoint occurred in 149 of 425 pts in the clopidogrel group vs. 151 of 426 pts in the placebo (+ ASA) group (HR: 0.98; 95% CI: 0.78–1.23). In a prespecified subgroup analysis, the primary endpoint was significantly reduced by clopidogrel in prosthetic graft pts (HR: 0.65; 95% CI: 0.45–0.95; p=0.025) but not in venous graft pts (HR: 1.25; 95% CI: 0.94–1.67; NS). A significant statistical interaction between treatment effect and graft type observed (p=0.008).<br><br>**Safety endpoint:**<br>• Severe bleeding (GUSTO)<br>• Although total bleeds were more frequent with clopidogrel, there was no significant difference between the rates of severe bleeding in the clopidogrel and placebo (+ ASA) groups (2.1% vs. 1.2%).	• Benefit only in prosthetic graft group

© American Heart Association, Inc. and American College of Cardiology Foundation

**MIRROR**<br>Tepe F, et al. 2012 (113)<br>22569995	**Aim**: To investigate the influence of dual antiplatelet therapy vs. ASA alone on local platelet activation and clinical endpoints in pts with PAD treated with endovascular therapy<br><br>**Study type**: Randomized, double-blind, placebo-controlled<br><br>**Size**: n=80 pts	**Inclusion criteria**:<br>• Age >18 y and <90 y.<br>• Chronic PAD in an artery of the upper leg (superficial femoral artery and/or popliteal artery) Stage Rutherford 3–5<br><br>**Exclusion criteria**: Acute limb-threatening ischemia requiring immediate action and restoration of flow within less than 1 hr.<br>• Recent major trauma including resuscitation, or active internal bleeding (e.g. gastrointestinal, genitourinary)<br>• Known severe hepatic or renal disorder (liver cirrhosis, stage B, C or serum creatinine >2.5 mg)<br>• Hx of bleeding diathesis of platelet count <100,000/mm^3.<br>• Cerebrovascular accident within 2 yr (thrombolysis only).<br>• Recent (within 2 mo) intracranial or intraspinal surgery or trauma (thrombolysis only).<br>• Recent (within 2 mo) major surgery (thrombolysis only)<br>• Intracranial neoplasms<br>• Arteriovenous malformations or aneurysms Severe uncontrolled hypertension (systolic blood pressure >220 mm hg, diastolic blood pressure >100 mm hg)<br>• Hypertensive or diabetic retinopathy<br>• Other disease with severe life limitation (e.g., advanced cancer, NYHA IV)<br>• Known autoimmune disorders.<br>• Known allergy against ASA	**Intervention**: 500 mg ASA and 300 mg clopidogrel before intervention followed by a daily dose of 100 mg ASA and 75 mg clopidogrel for 6 mo<br><br>**Comparator**: Clopidogrel replaced by placebo	**1° endpoint**:<br>• Local concentrations of platelet activation markers β-thromboglobulin and CD40L, and the rate of pt's resistant to clopidogrel<br>• The median peri-interventional concentration of β-TG was 224.5 vs. 365.5 (p=0 0.03) in the clopidogrel and placebo group. The concentration of CD40L was 127 and 206.5 (p=0 0.05). 30% of pts who had clopidogrel were resistant. 2 clopidogrel and 8 placebo pts required TLR (p=0.04). The clopidogrel pts who needed revascularisation were both resistant to clopidogrel.<br><br>**Safety endpoint**: Minor bleeding complications occurred in 1 clopidogrel and 2 placebo pts.	N/A

© American Heart Association, Inc. and American College of Cardiology Foundation

		and/or clopidogrel.<br>• Childbearing potential or existing pregnancy.<br>• Contraindications to urokinase, reteplase, clopidogrel, heparin and acetylsalicylic acid.<br>• Pt who has previously been included in this trial.<br>• Pt who requires long-term Cox2 inhibition.<br>• Pt who is not able to sign the informed consent form			
Bonaca MP, et al. 2013(114) 23501976	**Aim:** The effect of vorapaxar on cardiovascular and peripheral vascular outcomes in pts who qualified for TRA2°P-TIMI 50 with sx PAD.<br><br>**Study type:** Randomized, double-blind, placebo-controlled trial<br><br>**Size:** n=3,787 pts	**Inclusion criteria:** Hx of IC in conjunction with an ABI <0.85 or previous revascularization for limb ischemia<br><br>**Exclusion criteria:**<br>• A planned revascularization that had not yet been performed;<br>• Hx of a bleeding diathesis<br>• Were receiving vitamin K antagonist therapy<br>• Had active hepatobiliary disease	**Intervention:** Vorapaxar<br><br>**Comparator:** Placebo	**1° endpoint:** Primary efficacy endpoint was cardiovascular death, MI, or stroke. The primary endpoint did not differ significantly with vorapaxar (11.3% vs. 11.9%; HR: 0.94; 95% CI: 0.78–1.14; p=0.53)<br><br>**Safety endpoint:** Principal safety endpoint was Global Utilization of Streptokinase and t-PA for Occluded Coronary Arteries (GUSTO) bleeding. Bleeding occurred more frequently with vorapaxar compared with placebo (7.4% vs. 4.5%; HR: 1.62; 95% CI: 1.21–2.18; p=0.001).	• Rates of hospitalization for ALI (2.3% vs. 3.9%; HR: 0.58; 95% CI: 0.39–0.86; p=0.006) and peripheral artery revascularization (18.4% vs. 22.2%; HR: 0.84; 95% CI: 0.73–0.97; p=0.017) were significantly lower in pts randomized to vorapaxar.
Strobl FF, et al. 2013(115) 24093324	**Aim:** Investigating the effects of dual antiplatelet therapy on TLR after balloon angioplasty ± stenting in the femoropopliteal segment<br><br>**Study type:** Prospective, randomized, single-center, double-blinded and placebo-	**Inclusion criteria:** PAD pts with TLR after femoropopliteal endovascular intervention<br><br>**Exclusion criteria:** N/A	**Intervention:** ASA and clopidogrel<br><br>**Comparator:** ASA	**1° endpoint:** At 6 mo, clopidogrel pts had significantly lower rates of TLR compared to placebo pts [2 (5%) vs. 8 (20%); p=0.04]. After stopping clopidogrel/placebo after 6 mo, there was no significant difference in TLR at 12 mo after treatment [9 (25%) clopidogrel vs. 12 (32.4%) placebo; p=0.35]. Mortality was 0 vs. 1 in the placebo group at 6 mo (p=0.32) and 0 vs. 3 at 12 mo (p=0.08).	N/A

© American Heart Association, Inc. and American College of Cardiology Foundation

	controlled clinical trial<br><br>**Size**: n=73 pts				
Antiplatelet Trialists Collaboration (graft arterial patency) 1994 (116) 8312766	**Aim**: To determine the efficacy of antiplatelet therapy in maintaining vascular patency in various categories of pts.<br><br>**Study type**: Overviews of 46 RCTs of antiplatelet therapy vs. control and 14 RCTs comparing one antiplatelet regimen with another.<br><br>**Size**: n=12,000 pts	**Inclusion criteria**: Pts at varying degrees of risk of vascular occlusion (by virtue of disease or of having some vascular procedure) were in trials of antiplatelet therapy vs. control or trials comparing different antiplatelet regimens<br><br>**Exclusion criteria**: 39 trials of antiplatelet therapy vs. control were identified among pts having peripheral vascular procedures or with PVD (see part I) but vascular occlusion was monitored systematically in only 14 of them	**Intervention**: Antiplatelt therapy<br><br>**Comparator**: No antiplatelet therapy	**1° endpoint**: Antiplatelet therapy produced a highly significant (2p <0.0001) reduction in vascular occlusion, with similar proportional reductions in several different types of pts As well as preventing subclinical occlusion, antiplatelet therapy produced a significant (2p=0.002) reduction of about one quarter in the odds of suffering a "vascular event" (nonfatal MI, nonfatal stroke, or vascular death).<br><br>**Safety endpoint**: No clear excess bleeding	• Allocation to antiplatelet therapy in the 14 trials with pts with PAD was associated with a proportional reduction of 43% (SD 8%) in vascular occlusion, which was highly significant. Studies of pts with saphenous vein grafts or prosthetic implants for lower limb disease contributed most of the data; of the 3 other studies, 1 assessed the patency of native vessels in pts with IC and 2 concerned pts who had had peripheral angioplasty.<br>• allocation to a mean scheduled duration of 19 mo of antiplatelet therapy produced a substantial absolute reduction of 92 (SD 15) per 1,000 in the risk of peripheral artery occlusion (15.7% of antiplatelet allocated pts vs. 24.9% of corresponding controls
Antiplatelet Trialists 2002(117) 11786451	**Aim**: To determine the effects of antiplatelet therapy among tps at high risk of occlusive vascular events.<br><br>**Study type**: Meta-	**Inclusion criteria**: PAD includes those with claudication and/or peripheral revascularization<br><br>**Exclusion criteria**: N/A	**Intervention**: Antiplatelet therapy<br><br>**Comparator**: Control	**1° endpoint**: Allocation to antiplatelet therapy reduced the combined outcome of any serious vascular event by about one quarter; nonfatal MI was reduced by one third, nonfatal stroke by one quarter, and vascular mortality by one sixth (with no apparent adverse effect on other deaths)	• Among 9,214 pts with PAD in 42 trials (compared with 4,939 such pts in 33 trials previously evaluated there was a proportional reduction of 23% (8%) in serious vascular events (p=0.004), with similar

© American Heart Association, Inc. and American College of Cardiology Foundation

	analysis of RCTs of antiplatelet therapy for prevention of death, MI, and stroke in high risk pts<br><br>**Size**: n=287 studies involving 135,000 pts in comparisons of antiplatelet therapy vs. control and 77,000 in comparisons of different antiplatelet regimens			**Safety endpoint:** The proportional increase in risk of a major extracranial bleed with antiplatelet therapy was about one half (OR: 1.6; 95% CI: 1.4–1.8), with no significant difference between the proportional increases observed in each of the 5 high risk categories of pts	benefits among pts with IC, those having peripheral grafting, and those having peripheral angioplasty<br>• Much of the data was from the picotamide trial
Morrow DA, et al.<br>2012(118)<br>22443427	**Aim**: Determine the impact of vorapaxar on secondry prevention of atherothrombotic events<br><br>**Study type**: RCT<br><br>**Size**: n=26,449 pts	**Inclusion criteria**: Pts who had a hx of MI, ischemic stroke, or PAD<br><br>**Exclusion criteria**: Pts were ineligible if they were planning to undergo a revascularization procedure, had a hx of bleeding diathesis, had recent active abnormal bleeding, were receiving ongoing treatment with warfarin, or had active hepatobiliary disease.	**Intervention**: Vorapaxar<br><br>**Comparator**: Placebo	**1° endpoint:** Composite of death from cardiovascular causes, MI, or stroke in 1,028 pts (9.3%) in the vorapaxar group and in 1,176 pts (10.5%) in the placebo group (HR for the vorapaxar group: 0.87; 95% CI: 0.80–0.94; p<0.001).<br><br>**Safety endpoint:** There was an increase in the rate of intracranial hemorrhage in the vorapaxar group (1.0%, vs. 0.5% in the placebo group; P<0.001).	•3,787 PAD pts
Bonaca MP, et al.<br>2013<br>23501976	**Aim**: Determine the effect of vorapaxar on CV and peripheral vascular outcomes<br><br>**Study type**: RCT<br><br>**Size**: n=26,449 pts	**Inclusion criteria**: Pts who qualified for TRA 2°P-TIMI 50 pts with a with stable atherosclerotic vascular disease and a prior MI, ischemic stroke, or PAD<br><br>**Exclusion criteria**: N/A	**Intervention**: Vorapaxar**.** Thienopyridine was planned at randomization in 12,410 pts<br><br>**Comparator**: Placebo	**1° endpoint**: CV death, MI, or stroke<br><br>**Safety endpoint**: Global Utilization of Streptokinase and t-PA for Occluded Coronary Arteries bleeding.	In the PAD Cohort:<br>• No significant difference between vorapaxar and comparator for CV death, MI, or stroke (11.3% vs. 11.9%; HR: 0.94; 95% CI: 0.78–1.14; p=0.53)<br>• Significantly lower rates of hospitalization for ALI for vorapaxar group (2.3% vs. 3.9%; HR: 0.58; 95% CI: 0.39–0.86; p=0.006)<br>• Significant increase in bleeding in vorapaxar group

© American Heart Association, Inc. and American College of Cardiology Foundation

					compared with placebo (.4% vs. 4.5%; HR: 1.63; 95% CI: 1.21–2.18; p=0.001).
Bohula EA, et al. 2015(119) 26338971	**Aim:** To determine whether the efficacy and safety of antiplatelet therapy with vorapaxar was modified by concurrent thienopyridine use.<br><br>**Study type:** Randomized, double-blind, placebo-controlled trial<br><br>**Size:** n=16,897 pts	**Inclusion criteria:** TRA 2°P-TIMI 50 pts who qualified with a MI in the preceding 2 weeks to 12 months and was restricted to.<br><br>**Exclusion criteria:** Pts without a hx of stroke or transient ischemic attack given its contraindication in that population	**Intervention:** Vorapaxar. Thienopyridine was planned at randomization in 12,410 pts<br><br>**Comparator:** Placebo	**1° endpoint:** Vorapaxar significantly reduced the composite of cardiovascular death, MI, and stroke in comparison with placebo regardless of planned thienopyridine therapy (planned thienopyridine, HR: 0.80; 95% CI: 0.70–0.91; p<0.001; no planned thienopyridine, HR: 0.75; 95% CI: 0.60–0.94; p=0.011; p-interaction=0.67).<br><br>**Safety endpoint:** Consistent with the findings in the overall cohort, these rates reveal an increased RR of GUSTO moderate to severe bleeding in pts treated with vorapaxar in comparison with placebo; however, there was no significant modification by planned thienopyridine use (planned thienopyridine HR: 1.50; 95% CI: 1.18–1.89, p<0.001; no planned thienopyridine HR: 1.90; 95% CI: 1.17–3.07; p=0.009; p-interaction=0.37	N/A
Bonaca MP, et al. 2016(120) 26826179	**Aim:** Evaluate the causes, sequelae and predictors of ALI in a contemporary population with sx PAD and whether PAR-1 antagonism with vorapaxar reduced ALI overall and by etiology.<br><br>**Study type:** Subgroup of a randomized trial<br><br>**Size:** n=3,787 pts	**Inclusion criteria:** TRA 2°P-TIMI 50 pts with PAD<br><br>**Exclusion criteria:** AF and absence of PAD	**Intervention:** Vorapaxar<br><br>**Comparator:** Placebo	**1° endpoint:** ALII<br>Vorapaxar reduced first ALI events by 41% (HR: 0.58; 95%CI: 0.39–0.86; p=0.006), as well as total ALI events by 41% (94 events vs. 56 events, risk ratio: 0.59; 95% CI: 0.38–0.93,p=0.022)<br><br>**Safety endpoint:** Bleeding (see TRA 2°P-TIMI 50)	• Most ALI events were graft thrombosis or in situ native vessel thrombosis<br>• Effect consistent across all etiologies

© American Heart Association, Inc. and American College of Cardiology Foundation

**PAD from TRACER**<br>Jones WS, et al. 2014(121)<br>25262270	**Aim**: Investigate the efficacy and safety of vorapaxar in NSTE ACS pts with documented PAD<br><br>**Study type**: Subgroup of large randomized trial<br><br>**Size**: n=936 pts	**Inclusion criteria**: TRACER pts with a hx of PAD<br><br>**Exclusion criteria**: TRACER pts without PAD	**Intervention**: Vorapxar<br><br>**Comparator**: Placebo	**1° endpoint**: Lower rates of ischemic end points, peripheral revascularization, and amputation with vorapaxar did not reach statistical significance.*<br><br>**Safety endpoint**: Vorapaxar increased bleeding in both pts with and without PAD at a similar magnitude of risk.	N/A
Katsanos K, et al.<br>2015 (122)<br>26274912	**Aim**: Comparative Efficacy and Safety of Different Antiplatelet Agents for Prevention of Major Cardiovascular Events and Leg Amputations in pts with PAD<br><br>**Study type**: Meta-analysis<br><br>**Size**: n=34,518 pts	**Inclusion criteria**: RCT using antiplatelet drugs in pts with PAD<br><br>**Exclusion criteria**: N/A	**Intervention**: Antiplatelet therapy<br><br>**Comparator**: Placebo	**1° endpoint**: MACE and leg amputations<br>A significant MACE reduction was noted with Ticagrelor plus aspirin (RR: 0.67; 95%CrI: 0.46–0.96; NNT=66), Clopidogrel (RR: 0.72; 95%CrI: 0.58–0.91; NNT=80), Ticlopidine (RR: 0.75; 95%CrI: 0.58–0.96; NN =87), and Clopidogrel plus aspirin (RR: 0.78; 95%CrI: 0.61–0.99; NNT=98).<br><br>Dual antiplatelet therapy with Clopidogrel plus aspirin significantly reduced major amputations following leg revascularization (RR: 0.68; 95%CrI: 0.46–0.99 compared to ASA, NNT=94)<br><br>**Safety endpoint**: The risk of severe bleeding was significantly higher with Ticlopidine (RR: 5.03; 95%CrI: 1.23–39.6; NNH=25), Vorapaxar (RR: 1.80; 95%CrI: 1.22–2.69; NNH=130), and Clopidogrel plus ASA (RR: 1.48; 95%CrI: 1.05-2.10; NNT=215)	N/A
Magnani G, et al.<br>2015(123)<br>25792124	**Aim**: To observe the safety and efficacy of vorapaxar<br><br>**Study type**: Multinational, double-blinded, placebo-controlled TRA 2°P-TIMI 50 trial	**Inclusion criteria**:<br>• Met TRA 2°P-TIMI 50 inclusion criteria<br>• Hx of spontaneous MI within prior 2 wk to 12 mo<br>• Those with symptomatic PAD had hx of IC in conjunction with either an ABI <0.85 or previous revascularization for limb ischemia	**Intervention**: Vorapaxar sulfate 2.5 mg (vorapaxar 2.08 mg) daily<br><br>**Comparator**: Placebo	**1° endpoint**: Composite endpoints of CV death, MI, or stroke, and CV death, MI, stroke, or recurrent ischemia leading to urgent coronary revascularization<br>• 3 y KM event rate of CV death, MI, or stroke was 7.9% in vorapaxar compared with 9.5% in placebo (HR: 0.80; 95% CI: 0.73–0.89; p<0.001).<br>• 3 y KM event rate of CV death, MI, stroke, or	• Vorapaxar was shown to reduce CV death, MI, or stroke in the intended use and FDA approved population (not those with a hx of stroke).

© American Heart Association, Inc. and American College of Cardiology Foundation

	**Size:** n=16,897 pts	**Exclusion criteria:** N/A		urgent coronary revascularization was 10.1% in vorapaxar and 11.8% in placebo (HR: 0.83; 95% CI: 0.76–0.90; p<0.001).<br>• 3 y KM event rate of CV death or MI was 7.2% in vorapaxar and 8.3% in placebo; HR: 0.83; 95% CI: 0.75–0.93, p<0.001).<br>• 3 y KM event rate of MI was 5.4% in vorapaxar and 6.4% in placebo (p<0.001)<br>• 3 y KM event rate of stroke was 1.2% in vorapaxar and 1.6% in placebo (p=0.002) individually.<br><br>**Safety endpoint:** GUSTO moderate or severe bleeding:<br>• Combined bleeding criteria was 3.7% with vorapaxar and 2.4% in placebo (HR, 1.55; 95% CI: 1.30–1.86, p<0.001).<br>• Severe bleeding was 1.3% with vorapaxar vs. 1.0% with placebo (HR 1.24; 95% CI: 0.92–1.66, P=0.16	
Berger JS et al, 2009<br>(124)	**Aim:** To determine the effect of ASA on CV event rates in pts with PAD<br><br>**Study type:** Meta-analysis of prospective RCTs<br><br>**Size:** n=18 trials, 5,269 pts	**Inclusion criteria:**<br>• Prospective RCTs<br>• PAD pts assigned to aspirin or placebo/control group<br>• Data on all-cause mortality, CV death, MI, stroke, and major bleeding<br><br>**Exclusion criteria:** N/A	**Intervention:** ASA<br><br>**Comparator:** Placebo/control	**1° endpoint:**<br>• Nonfatal MI, nonfatal stroke, CV death<br>• Secondary outcomes were all-cause mortality<br><br>**Safety endpoint:** Major bleeding	• ASA therapy, alone or in combination with dipyridomole, had no significant effect on CV events<br>• ASA did have significant reduction in nonfatal stroke<br>• No significant outcome for MI, CV mortality, or all-cause mortality

ABI indicates ankle-brachial index; ACS, acute coronary syndrome; ALI, acute limb ischemia; ASA, aspirin; CHD, coronary heart disease; CI indicates confidence interval; CLI, critical limb ischemia; CV, cardiovascular; GP, general practitioner; GUSTO, Global Utilization of Streptokinase and t-PA for Occluded; Coronary Arteries HR, hazard ratio; IC, intermittent claudication; IV, intravenous; KM, Kaplan-Meier; MACE, major adverse cardiac event; MI, myocardial infarction; N/A, not applicable; NNT, number needed to treat; NS, not significant; NYHA, New York Heart Association; NSAIDs, nonsteroidal anti-inflammatory drugs; OR, odds ratio; PAD, peripheral artery disease; PTA, percutaneous transluminal angioplasty; pt, patient; PVD, peripheral vascular disease; RCT, randomized controlled trial; RR, relative risk; and TLR, target lesion revascularization.

© American Heart Association, Inc. and American College of Cardiology Foundation

Evidence Table 14. Nonrandomized Trials, Observational Studies, and/or Registries of Antiplatelet Agents–Section 5.2.

Study Acronym Author Year	Aim of Study; Study Type; Study Size (N)	Patient Population	Study Intervention (include # patients) / Study Comparator (include # patients)	Endpoint Results (include Absolute Event Rates, P value; OR or RR; and 95% CI)	Relevant 2° Endpoint (if any); Study Limitations; Adverse Events
Armstrong EJ et al. 2015(125) 25864042	**Aim:** This study was conducted to determine whether there is additive benefit of DAPT with ASA and clopidogrel compared with ASA monotherapy among pts with sx peripheral arterial disease.<br><br>**Study type:** Observational cohort<br><br>**Size:** n=629 pts	**Inclusion criteria:**<br>• UC Davis PAD registry<br>• Claudication or CLI<br>• All had angiography<br><br>**Exclusion criteria:**<br>• Warfarin use (96 pts)<br>• No antiplatelet therapy (28)<br>• In registry for ALI, carotid artery stenosis, subclavian artery stenosis, or renal artery stenosis	**Groups:** 348 with DAPT, 281 with ASA only<br><br>Record review with median follow 3.2 y	**1° endpoint:** During 3 y of follow-up, 50 events (20%) occurred in the DAPT group vs. 59 (29%) in the ASA monotherapy group. After propensity weighting, DAPT use was associated with a decreased risk of MACEs (adjusted HR: 0.65; 95% CI: 0.44–0.96) and overall mortality (adjusted HR: 0.55; 95% CI: 0.35–0.89). No association was found between DAPT use and the risk of major amputation (adjusted HR: 0.69; 95% CI: 0.37–1.29). In a subgroup of 94 pts who underwent point-of-care platelet function testing, 21% had decreased response to ASA and 55% had a decreased response to clopidogrel. No association was found between a reduced response to ASA or clopidogrel and adverse events at 1 y.	N/A

ALI indicates acute limb ischemia; ASA, acetylsalicylic acid; CI, confidence interval; CLI, critical limb ischemia; DAPT, dual antiplatelet therapy; HR, hazard ratio; MACE, major adverse cardiac event; PAD, peripheral artery disease; and pt, patient.

Evidence Table 15. Randomized Trials Comparing Statin Agents–Section 5.2.

Study Acronym Author Year	Aim of Study; Study Type; Study Size (N)	Patient Population	Study Intervention (include # patients) / Study Comparator (include # patients)	Endpoint Results (include Absolute Event Rates, P value; OR or RR; and 95% CI)	Relevant 2° Endpoint (if any); Study Limitations; Adverse Events
**HPS**<br>HPS Collaborative Group 2007(126) 17398372	**Aim:** Assess impact of cholesterol-lowering therapy on major adverse vascular events in pts with PAD<br><br>**Study type:** Prospective, blinded, RCT.<br><br>**Size:** n=20,536 pts	**Inclusion criteria:**<br>• Age 40–80 y<br>• Chol >135mg/dL<br>• PAD, CVD, DM, or HTN (if male and >65)<br><br>**Exclusion criteria:** If PCP feels statin clearly indicated or contraindicated; prior MI, stroke, or admission with angina in previous 6 mo; liver dysfunction; renal dysfunction;	**Intervention:** Simvastatin 40 mg (10,269)<br><br>**Comparator:** Placebo (10,267)	**1° endpoint:** 24% (95% CI: 19–28; p<0.0001) proportional reduction in the first occurrence of a major vascular event Those with LEPAD: 22% (95% CI: 15–29; p<0.0001) proportional reduction<br><br>**1° Safety endpoint (if relevant):**<br>• CPK elevation >10x ULN in 1 out of 10,000 pts/y.	• Comparable proportional reduction in first major coronary event, stroke, and revascularization (considered separately)<br>• 16% reduction in peripheral vascular events (5%–25%; p=0.006), primarily through reduction in noncoronary revascularizations<br>• Statin group: 85% compliant with statin<br>• Non-statin group: 17% non-study statin

© American Heart Association, Inc. and American College of Cardiology Foundation

		muscle disease; concurrent Rx (cyclosporine, fibrates, niacin); child bearing; severe CHF; limitations to compliance.		• Mean follow-up 5.0 y	
Mohler ER, et al. 2003(127) [12952839]	**Aim**: Determine whether cholesterol lowering with atorvastatin improves walking performance in pts with IC<br><br>**Study type**: Prospective, blinded, RCT<br><br>**Size**: n=354 pts	**Inclusion criteria**:<br>• Age >25 y<br>• Stable IC for 6 mo<br>• ABI ≤0.90<br>• 20% reduction in ABI post exercise (Gardner)<br>• LDL ≤160.<br><br>**Exclusion criteria**:<br>• MI, coronary revascularization, peripheral revascularization within 6 mo.<br>• USA within 3 mo.<br>• Stroke or TIA within 6 mo.<br>• DVT/PE within 3 mo.<br>• Current engagement in exercise rehab program.	**Intervention**: Atorvastatin 10 mg daily (120 pts) or atorvastatin 80 g daily (120 pts)<br><br>**Comparator**: Placebo (114 pts)	**1° endpoint**: Change in MWT at 12 mo.<br>• Placebo: 50±12 s<br>• Atorva 10: 90±18<br>• Atorva 80: 90±18 (p=0.37)	• Change in PFWT at 12 mo<br>• Placebo: 39±8<br>• Atorva 10: 74±14 (p=0.13)<br>• Atorva 80: 81±15 (p=0.025)
**ICPOP**<br>Hiatt WR, et al. 2010(128) [20212073]	**Aim**: Test the hypothesis that ER Niacin plus lovastatin would improve exercise performance in pts with PAD and claudication compared with diet intervention.<br><br>**Study type**: RCT<br><br>**Size**: n=387	**Inclusion criteria**:<br>• Age >40 y<br>• Stable IC<br>• ABI ≤0.90<br>• 20% reduction in ABI post-exercise (Gardner)<br>• LDL ≤160<br>• PWT 1–20 min<br>• <20% variability in 2 assessments.<br><br>**Exclusion criteria**: Pts with CAD or other indication for lipid lowering therapy.	**Intervention**: Low-dose Niacin 1000 mg plus lovastatin 40 mg or high-dose Niacin 2000 mg plus lovastatin 40 mg<br><br>**Comparator**: Diet	**1° endpoint**:<br>• Change from baseline in PWT and in claudication onset time at 28 wk<br>• Diet: 26.5%; 95% CI: 16.4%–37.6%<br>L• ow Niacin/Lova: 38.6%; 95% CI: 27.6%–50.6%, p=0.096<br>• High Niacin/Lova: 37.8%; 95% CI: 26.6%–50.1%, p=0.137<br><br>**Safety endpoint**: 2/3 of pts in each treatment group reported drug-related adverse event (pruritis, diarrhea, elevated blood sugar). Flushing in 54%. Serious adverse events were	• Change in ABI<br>• Walking Impairment Questionnaire<br>• Composite of CV events

© American Heart Association, Inc. and American College of Cardiology Foundation

				similar in all 3 groups (11.2%, 11.2%, 10.3%)	
Giri J, et al. 2006(129) 16516084	**Aim:** To determine whether statin use is associated with less annual decline in LE functioning with/without LEPAD<br><br>**Study type:** Prospective cohort study (identified in noninvasive vascular lab between 1998-2000 at 3 Chicago institutions).<br><br>**Size:** n=544	**Inclusion criteria:**<br>• PAD group: ABI <0.90.<br>• Non-PAD: 1.50 ≥ABI ≥0.90<br><br>**Exclusion criteria:**<br>• SNF resident<br>• Wheelchair bound<br>• Foot or leg amputation<br>• Non-English speaking<br>• Recent major surgery<br>• Prior vasc surgery<br>• Normal ABI	**Intervention:** On statin<br><br>**Comparator:** Not on statin	**1° endpoint:**<br>• Pts with PAD using statins had less annual decline in:<br>• Usual-pace walking velocity (0.002 vs. -0.024 m/s/y; p=0.013)<br>• Rapid-pace walking velocity (-0.006 vs. -0.042 m/s/y; p=0.006)<br>• 6 min walk performance (-34.5 vs. -57.9 ft/y; p=0.088)<br>• Summary performance score (-0.152 vs. -0.376; p=0.067) compared with non-users.<br>• Among pts without-PAD, there were no significant associations between statin use and functional decline.	N/A
West AM, et al. 2011(130) 21570685	**Aim:** LDL-C cholesterol by adding ezetimibe to statin therapy would regress atherosclerosis measured by MRI in the SFA in PAD.<br><br>**Study type:** Single center, prospective, RCT, double-blinded<br><br>**Size:** n=87 pts	**Inclusion criteria:** 30–85 y, PAD (ABI 0.4–0.9)<br><br>**Exclusion criteria:** Rest pain, CLI, contraindication to MRI, pregnancy.	**Intervention:** Statin-naive (randomized to simvastatin or simvastatin plus ezetimibe) or previously on statin given open label ezetimibe<br><br>**Comparator:** Simvastatin alone	**1° endpoint:**<br>• Atherosclerotic plaque volume in the proximal 15–20 cm of SFA at baseline and annually x 2.<br>• Baseline and y 2 volumes:<br>• S + E (11.5 ± 1.4 vs.10.5 ± 1.3 cm^3; p=NS) or<br>• S (11.0 ± 1.5 vs.10.5 ± 1.4 cm^3, p=NS)<br>• E (10.0 ± 0.8–10.8 ± 0.9; p<0.01)	• Only 72 pts at follow-up (2 died, 11 lost to follow-up, 2 withdrew prior to baseline imaging)<br>• Statin initiation with or without ezetimibe in statin-naive pts halted plaque progression<br>• Ezetimibe added to existing statin still resulted in progression of plaque volume; ezetimibe's effect on PAD may depend on relative timing of statin therapy.<br>• LDL-C was lowered by the addition of ezetimibe in both groups, but did not translate to change in plaque volume. Study was underpowered to detect a difference between S and S + E
Stoekenbroek RM, et al. 2015(131) 25595417	**Aim:** Determine whether high-dose statin vs. usual dose statin reduces incidence of PAD and CAD outcomes in pts	**Inclusion criteria:**<br>• Age ≤80 y<br>• Confirmed prior MI<br><br>**Exclusion criteria:** N/A	**Intervention:** Atorvastatin 80mg<br><br>**Comparator:** Simvastatin 20–40mg	**1° endpoint:**<br>• No PAD at baseline: new clinical Dx of PAD requiring diagnostic procedures or interventions.<br>• 2.2% in atorvastatin	• Post-hoc evaluation of CAD outcomes in pts with PAD at baseline<br>• Baseline PAD in 374 pts (4.2%)<br>• Major coronary events nonsignificantly lower in the atorvastatin group (14.4%) compared with the simvastatin group

© American Heart Association, Inc. and American College of Cardiology Foundation

	with PAD<br><br>**Study type:** Multi-center, RCT, open-label, blinded outcome assessment<br><br>**Size:** n=8,888 pts			• 3.2% in simvastatin<br>• (HR: 0.70; 95% CI: 0.53–0.91; p=0.007)<br>• Known PAD at baseline: new hospitalization for treatment for PAD<br>• No significant difference (18.3% vs. 16.5%)	(20.1%) (HR: 0.68; 95% CI: 0.41–1.11; p=0.13).<br>• Atorvastatin reduced overall CV (p=0.046) and coronary events (p=0.004) and coronary revascularization (p=0.007)
Aung PP, et al. 2007(132)<br>17943736	**Aim:** Assess outcomes with statin vs. placebo in individuals with LEPAD<br><br>**Study type:** Meta-analysis of 18 RCT.<br><br>**Size:** n=10, 049	**Inclusion criteria:** RCTs of lipid-lowering therapy in PAD of the lower limb<br><br>**Exclusion criteria:** N/A	**Intervention:** Lipid-lowering therapies<br><br>**Comparator:** Placebo	**1° endpoint:**<br>• Overall mortality: no significant difference (OR: 0.86; 95% CI: 0.49–1.50)<br>• Total Cardiovascular events: no significant difference (OR: 0.8; 95% CI: 0.59–1.09)	Subgroup analysis (exclusion of PQRST):<br>• Significant reduction of total cardiovascular events (OR: 0.74; 95% CI: 0.55–0.98)<br>• Significant reduction of total coronary events (OR: 0.76; 95% CI: 0.67–0.87)<br>• Greatest effectiveness in statin use for individuals with LDL ≥3.5 mmol/L

ABI indicates ankle-brachial index; CAD, coronary artery disease; CHF, congestive heart failure; CI indicates confidence interval; CLI, critical limb ischemia; CPK, creatine phosphokinase; CVD, cardiovascular disease; CV, cardiovascular; DVT/PE, deep vein thrombosis/pulmonary embolism; HR, hazard ratio; HTN, hypertension; IC, intermittent claudication; LDL-C, low-density lipoprotein; LE, lower extremity; LEPAD, lower extremity peripheral artery disease; MI, myocardial infarction; MRI, magnetic resonance imaging; MWT, maximal walking time; N/A, not applicable; PAD, peripheral artery disease; PCP, primary care physician; PFWT, pain-free walking time; pt, patient; PWT, peak treadmill walking time; RCT, randomized controlled trial; RR, relative risk; SFA, superficial femoral artery; SNF, skilled nursing facility; TIA, transient ischemic attack; ULN, upper limit normal; and USA, unstable angina.

Evidence Table 16. Nonrandomized Trials, Observational Studies, and/or Registries of Statin Agents–Section 5.2.

Study Acronym<br>Author<br>Year	Aim of Study;<br>Study Type;<br>Study Size (N)	Patient Population	Study Intervention (include # patients) / Study Comparator (include # patients)	Endpoint Results (include Absolute Event Rates, P value; OR or RR; and 95% CI)	Relevant 2° Endpoint (if any);<br>Study Limitations;<br>Adverse Events
**REACH Registry**<br>Kumbhani DJ, et al. 2014(133)<br>24585266	**Aim:** Assess impact of statin use on primary adverse limb outcomes at 4 y and composite CV death, MI, stroke.<br><br>**Study type:** Registry<br><br>**Size:** n=5,861 pts	**Inclusion criteria:** Documented sx PAD with complete 4 y follow-up.<br><br>**Exclusion criteria:** Not meeting inclusion criteria; no follow-up data for primary endpoint; no documented Hx of PAD; no information regarding statin use at enrollment	**Intervention:** Statin use (62%)<br><br>**Comparator:** No statin use (38%)	**1° endpoint:** Primary adverse limb outcomes (worsening claudication, new CLI, new LE revascularization, new ischemic amputation) at 4 y<br>- 22% in statin<br>- 26.2% in no statin (HR: 0.82; 95% CI: 0.72–0.92; p=0.0013)	• Registry data (undefined confounders)<br>• Need for revascularization, worsening claudication may be subjectively determined by observer<br>• More likely on statin if enrolled by cardiologist than by provider of other specialty (vascular surgery)

© American Heart Association, Inc. and American College of Cardiology Foundation

Vogel TR, et al. 2013(134) 24300135	**Aim**: To evaluate preoperative administration of statins and longitudinal limb salvage after LE endovascular revascularization and LE open surgery.<br><br>**Study type**: Medicare Claims Database Review<br><br>**Size**: n=22,954	**Inclusion criteria**: Age ≥65 y with a diagnosis of atherosclerosis of LE arteries who were hospitalized during 2007–2008 for LE revascularization<br><br>**Exclusion criteria**: N/A	**Intervention**: On statin at time of revascularization (11,687)<br><br>**Comparator**: No statin	**1° endpoint**: 1 y limb salvage rates<br>Statin: RR=0.82; 95% CI: 0.78–0.86; p<0.0001	N/A
Westin GG, et al. 2014(135) 24315911	**Aim**: To determine the associations between statin use and MACCE and amputation-free survival in CLI pts.<br><br>**Study type**: Single center registry (retrospective cohort)<br><br>**Size**: n=380 (between 2006–2012)	**Inclusion criteria**: ≥1 presentation with CLI (Rutherford 4–6). "On statin" if hospitalization data or most recent pre-procedure clinic note had statin listed (65% of pts enrolled)<br><br>**Exclusion criteria**: N/A	**Intervention**: On statin (246 or 65%)<br><br>**Comparator**: No statin	**1° endpoint**: Composite MACCE (death, MI, stroke) within 1 y of procedure.<br><br>**Results:** Statin: 18%, no statin: 23% (HR: 0.53; 95% CI: 0.28–0.99; p=0.048)<br>Propensity score to control for confounding variables	• Secondary outcomes (1 y): death, MI, stroke, ipsilateral LE bypass, ipsilateral major amputation, amputation-free survival, vessel patency (primary, primary assisted, secondary)<br>Amputation-free survival HR: 0.59; 95% CI: 0.35–0.98; p=0.04<br>Improved vessel patency<br>• Pts on statin had higher rates of DM, HTN, CAD, CVD, prior MI
Feringa HH, et al. 2007(136) 17360142	**Aim**: To determine whether higher-dose statins and lower dose LDL are independently associated with better outcomes in PAD<br><br>**Study type**: Single center, prospective, observational, cohort study<br><br>**Size**: n=1,374 pts	**Inclusion criteria:**<br>• Age ≥18<br>• ABI ≤0.90<br><br>**Exclusion criteria:**<br>• MI or coronary revascularization in past 6 mo<br>• Liver disease (Cirrhosis or hepatitis)	**Intervention**: Statin therapy (propensity analysis applied to control for confounders)	**1° endpoint:** All-cause mortality and cardiac death<br><br>**Results:**<br>• 6 mo LDL:<br><100 in 30.8%<br><70 in 9.7%<br>• Lowest all-cause and cardiac mortality (18% and 13%) in pts with lowest cholesterol (<70), p<0.001; gradually increasing with increasing cholesterol levels	Secondary endpoint: progression to kidney failure<br>Conclude: pts with ABI <0.90 benefit from LDL <70<br>Mean follow-up 6 y

© American Heart Association, Inc. and American College of Cardiology Foundation

CAD indicates coronary artery disease; CLI, critical limb ischemia; CVD, cardiovascular disease; CV, cardiovascular; DM, diabetes mellitus; HR, hazard ratio; HTN, hypertension; LDL, low-density lipoprotein; LE, lower extremity; MACCE, major adverse cardiovascular and cerebrovascular events; MI, myocardial infarction; N/A, not applicable; pt, patient; and RR, relative risk.

Evidence Table 17. RCTs for Antihypertensive Agents– Section 5.3.

Study Acronym; Author; Year Published	Aim of Study; Study Type; Study Size (N)	Patient Population	Study Intervention (# patients) / Study Comparator (# patients)	Endpoint Results (Absolute Event Rates, P value; OR or RR; & 95% CI)	Relevant 2° Endpoint (if any); Study Limitations; Adverse Events
**HOPE Study**<br>ABI subgroup<br>Ostergren J, et al.<br>2004(137)<br>14683738	**Aim**: Impact of ramipril on CVD events<br><br>**Study type**: RCT<br><br>**Size**: n=9,297 pts overall, 4,051 with PAD<br>8,986 pts with ABI measured. 3,099 pts with PAD	**Inclusion criteria**: Age ≥55 y with CVD (CAD, stroke, PAD) or DM+RF<br><br>**Exclusion criteria**:<br>• HF or LV dysfunction (EF <0.4)	**Intervention**: Ramipril vs. placebo<br>PAD group (N=1996 ramipril vs. N=2085 placebo)	**1° endpoint**:<br>• MACE<br>• Asx PAD: ABI 0.6–0.9 15.7 vs. 21.6 0.72 (0.56, 0.92)<br><0.6 16.4 vs. 22.0 0.77 (0.55, 1.09)<br>• Clinical PAD 20.1 vs. 25.8 0.75 (0.61, 0.92)	N/A
**HOPE**<br>Yusuf S, et al.<br>2000(138)<br>10639539	**Aim**: To investigate effect of ACEI (Ramipril-10mg) on CV events in high risk pts ≥55 y with a mean entry BP of 139/79 mmHg in both groups<br><br>**Study type**: RCT, 2x2 factorial design<br><br>**Size**: n=9,297 pts	**Inclusion criteria**: Pts ≥55 y with hx of CAD, stroke, PVD or DM with either hypertension, elevated total cholesterol, low LDL, smoking, or micro albuminuria.<br><br>**Exclusion criteria**:<br>• HF<br>• <0.40 EF<br>• On ACE-I or Vitamin E<br>• Uncontrolled hypertension or overt nephropathy<br>• Had MI or stroke<4 wk	**Intervention**: Ramipril (10mg) (4,645)<br><br>**Comparator**: Placebo (4,652)	**1° endpoint**: Composite of MI, stroke, or mortality from CV causes.<br><br>**Results**: Endpoint reduction Ramipril group vs. Placebo (14% vs. 17.8%; RR: 0.78; CI: 0.70–0.86; p<0.001)	• Death from cardiac causes reduced (6.1% vs. 8.1%; p<0.001)<br>• Death from MI reduced (9.9% vs. 12.3%; p<0.001)<br>• Death from any cause (10.4 % vs. 12.2%; p=0.005)<br>• Ramipril was found to be beneficial in the PVD subgroup
**ONTARGET**<br>Yusuf S, et al.<br>2008(139)<br>18378520	**Aim**: Impact of telmisartan vs. ramipril vs. combination on CVD events in pts with vascular disease or high-risk DM	**Inclusion criteria**:<br>• Vascular disease (CAD, cerebrovascular disease, PAD) or DM+end-organ damage<br><br>**Exclusion criteria**:<br>• HF or LV dysfunction	**Intervention**: Telmisartan 80mg vs. Ramipril 10 vs. combo<br>PAD group (N=1136 ramipril vs. N=1161 telmisartan vs. N=1171 combo)	**1° endpoint**:<br>• MACE:<br>• Overall trial 16.5% in Ramipril, 16.7% telmisartan, 16.3% combination group.<br>• Ramipril vs. telmisartan	• Increased risk of hypotension, syncope, renal dysfunction in combination group

© American Heart Association, Inc. and American College of Cardiology Foundation

	**Study type**: RCT<br><br>**Size**: n=8,576 pts overall, 3,468 with PAD			RR: 1.01; 95% CI: 0.94–1.09)<br>• Combo vs. Ramipril RR: 0.99; 95% CI: 0.92–1.07	
**INVEST**<br>PAD subgroup<br>Bavry AA, et al. 2010(140)<br>19996066	**Aim**: Compare CCB vs. BB based treatment regimens for HTN in older with CAD<br><br>**Study type**: Prespecified post hoc analysis of RCT<br><br>**Size**: n=2,699 pts (total trial: 22,576) pts. Mean follow-up 2.7 y Primary outcome: death, MI, stroke.	**Inclusion criteria**:<br>• PAD+CAD pts (clinician defined)<br>• Age ≥50 ywith HTN+stable CAD<br><br>**Exclusion criteria**: Unstable angina, angioplasty, CABG, stroke within 1 mo<br>Sinus bradycardia, sick sinus syndrome, AVB >1st degree<br>Class IV HF<br>Creatinine ≥4<br>Liver failure	**Intervention**: Intensive therapy with verapamil±trandolapril vs. atenolol±hctz	**1° endpoint**:<br>• 16.2% in PAD pts<br>• Least frequently SBP 135-145 with j-shaped relationship<br>• No difference between 2 types of medication strategies (HR: 0.89; 95% CI: 0.74–1.07; p=0.21)	• No difference in vascular procedures (HR: 0.94; 95% CI: 0.77–1.13; p=0.5)<br>• Poor/Fair QoL (HR: 0.87; 95% CI: 0.77–0.99; p=0.03)
Zanchetti A, et al. 2006(141)<br>17053536	**Aim**: Valsartan vs. amlodipine<br><br>**Study type**: Subgroup analysis of PAD<br><br>**Size**: n=15,245 pts CVD events: cardiac death, HF hospitalization, MI, emergency cardiac procedure. Mean follow-up 4.2 y.	**Inclusion criteria**: Overall trial:<br>• Age ≥50 y<br>• HTN, CVDRF or CVD.<br>Clinical PAD=2114<br><br>**Exclusion criteria**:<br>• Renal artery stenosis<br>• Coronary revascularization or stroke within 3 mo<br>• Valvular heart disease<br>• Severe liver or kidney disease<br>• HF<br>• Requiring BB use	**Intervention**:<br>• Valsartan vs. amlodipine<br>I• n PAD subgroup N=1052 valsartan, N=1062 amlodipine	**1° endpoint**: In PAD subgroup: Event rates 13.4 vs. 13.6 p=0.63	• Amlodipine with greater BP decrease.
Diehm C, et al. 2011(142)<br>21602713	**Aim**: Nebivolol vs. hctz on walking capacity in IC<br><br>**Study type**: RCT<br><br>**Size**: n=Parallel in 177 pts with 127	**Inclusion criteria**: PAD with IC with HTN<br><br>**Exclusion criteria**:<br>Inability to exercise<br>Poorly controlled DM	**Intervention**: Nebivolol 5 mg vs. hctz 25 mg	**1° endpoint**: Initial claudication distance: Increase 28% vs. 26%.	• No difference in ABI change between groups.<br>• No adverse effects BB

© American Heart Association, Inc. and American College of Cardiology Foundation

	completers				
**NORMA trial** Espinola-Klein C, et al. 2011(143) [21646599]	**Aim:** Compare BB on walking parameters<br><br>**Study type:** RCT<br><br>**Size:** n=128 pts	**Inclusion criteria:** IC+HTN<br><br>**Exclusion criteria:**<br>• CLI<br>• Inability to exercise<br>• Contraindications BB<br>• MI within 6 mo<br>• Uncontrolled DM	**Intervention:** Nebivolol 5mg vs. metoprolol 95mg	**1° endpoint:** ICD and ACD increased in both groups. No difference between groups.	• No difference in ABI change between treatments.<br>• 7 pts with AE bradycardia Re-enforces safety BB in IC
Paravastu SC, et al. Cochrane Review 2013(144) [24027118]	**Aim:** BB Safety in PAD<br><br>**Study type:** Update of a review<br><br>**Size:** n=119 pts	**Inclusion criteria:** 6 RCT comparing BB to placebo.	**Intervention:** BB vs. placebo	**1° endpoint:** None of the trials showed worsening of walking measures with BB	• No evidence that BB adversely affect walking parameters in IC
**ALLHAT** 2002(145) [12479763]	**Aim:** Comparison of an alpha blocker, ACE inhibitor, or CCB, each compared to a thiazide-type diuretic on non-fatal or fatal CHD<br><br>**Study type:** RCT<br><br>**Size:** n=33,357 pts	**Inclusion Criteria:**<br>• Age >50 y<br>• African American15,085 (35.5)<br>• White 19,977 (47.0)<br>• Hispanics 5,299 (12.5)<br><br>**Exclusion criteria:** N/A	Intervention: Chlorthalidone vs. Doxazosin, Amlopdipine, or Lisinopril	**1° endpoint:** Nonfatal MI and fatal CHD	• No difference in primary outcome (nonfatal MI and fatal CHD)

ABI indicates ankle-brachial index; ACEI, angiotensin converting enzyme inhibitor; AE, adverse event; AVB, atrioventricular block; ACD, absolute claudication distance; ACEi, angiotensin-converting-enzyme inhibitor; AE, adverse event; BB, beta blockers; BP, blood pressure; CABG, coronary artery bypass grafting; CAD, coronary arterial disease; CCB, calcium channel blockers; CI, confidence interval; CLI, critical limb ischemia; CVD, cardiovascular disease; CVDRF, cardiovascular disease risk factors; CV, cardiovascular; DM, diabetes mellitus; EF, ejection fraction; hctz, hydrochlorothiazide; HF, heart failure; HR, hazard ratio; HTN, hypertension; IC, intermittent claudication; LV, left ventricular; MACE, major adverse cardiovascular events; MI, myocardial infarction; PAD, peripheral artery disease; PVD, peripheral vascular disease; QoL, quality of life; RCT, randomized controlled trial; RR, relative risk; and SBP, systolic blood pressure.

© American Heart Association, Inc. and American College of Cardiology Foundation

Evidence Table 18. Nonrandomized Trials, Observational Studies, and/or Registries of Antihypertensive Agents–Section 5.3.

Study Acronym; Author; Year Published	Study Type/Design; Study Size	Patient Population	Primary Endpoint and Results (include P value; OR or RR; & 95% CI)	Summary/Conclusion Comment(s)
Feringa HH, et al.<br>2006(146)<br>16545650	**Study type**:<br>Observation Cohort<br><br>**Size**: 2,420 PAD pts	**Inclusion criteria**:<br>• Referred for Evaluation of PAD<br>• ABI ≤0.9<br>• 77% with ABI ≤0.7<br><br>**Exclusion criteria**: N/A	All-cause mortality: 44% at median follow-up time of 8 y. MV and propensity score adjusted<br>BB HR: 0.68; 95% CI: 0.58–0.80; p<0.001<br>ACEi HR: 0.80; 95% CI: 0.69–0.94; p=0.005<br>Nonsignificant: diuretics, CCB	• Potential for residual confounding<br>• Supports use of BB, ACEi in clinical PAD
**HOPE**<br>Sleight P, et al.<br>2000(147)<br>11967789	**Study type**:<br>Editorial review<br><br>**Size**: n=9,297 pts	**Inclusion criteria**: N/A<br><br>**Exclusion criteria**: N/A	**1° endpoint**: N/A<br><br>**Results:** N/A	• Significant benefits in mortality and morbidity from use of Ramipril in subjects at high risk of future CV events (ACEi could be offered to wider group of pts. including those on Aspirin prophylaxis).<br>• ACEi found to be highly cost effective in a preliminary analysis

ACEi indicates angiotensin-converting-enzyme inhibitor; BB, beta blocker; CCB, calcium channel blockers; CI, confidence interval; HR, hazard ratio; N/A, not applicable; OR, odds ratio; pt, patient; and RR, relative risk.

Evidence Table 19. RCTs for Smoking Cessation–Section 5.4.

Study Acronym; Author; Year Published	Aim of Study; Study Type; Study Size (N)	Patient Population	Study Intervention (# patients) / Study Comparator (# patients)	Endpoint Results (Absolute Event Rates, P value; OR or RR; & 95% CI)	Relevant 2° Endpoint (if any); Study Limitations; Adverse Events
Rigotti NA, et al.<br>Helping HAND Trial<br>2014(148)<br>25138333	**Aim**: To compare post discharge tobacco cessation intervention with standard care in hospitalized adult smokers who want to quit<br><br>**Study type**:<br>single-center RCT<br><br>**Size**: n=397 hospitalized adult	**Inclusion criteria**:<br>• Age >18 y<br>• Current smoker<br>• Plan to quit<br>• Agree to accept medication<br>• 38% (N=151) with Circulatory Dx: cardiovascular, peripheral vascular, cerebrovascular<br><br>**Exclusion criteria**: LOS <24 H, no telephone, substance use (other than tobacco, alcohol, marijuana), admitted for alcohol or drug overdose, medical	**Intervention**: Automated voice response calls, free smoking cessation medication for 90 d<br><br>**Comparator**: Printed recommendations	**1° endpoint**:<br>• Biochemically confirmed tobacco abstinence at 6 mo<br>• 26% vs. 15% (RR: 1.71; 95% CI: 1.14–2.56; p=0.009) NNT 9.4<br>• Subgroup analysis in Circulatory disorders showed similar results	• Single-center<br>• 20% lost to follow-up at 6 mo

© American Heart Association, Inc. and American College of Cardiology Foundation

	smokers	instability, admitted to obstetric or psychiatric units, life expectancy <12 mo			
Rigotti NA, et al. 2010(149)<br>20048210	**Aim:** To evaluate effect of varenicline on smoking cessation rates in pts with stable cardiovascular disease.<br><br>**Study type:** Multi-center RCT<br><br>**Size:** n=714 pts	**Inclusion criteria:**<br>• Age 35–75 y<br>• Want to quit smoking but had not tried in past 3 mo<br>• Stable CVD (CAD, PAD, Cerebrovascular disease). PAD=179, 25%<br><br>**Exclusion criteria:**<br>• Cardiovascular intervention within 2 mo<br>• Uncontrolled hypertension<br>• Prior amputation<br>• Class III/IV CHF<br>• Moderate/severe COPD<br>• Uncontrolled GI/hepatic/endocrine disease<br>• Severe renal impairment<br>• Cancer, depression, psychosis, drug or alcohol use/abuse	**Intervention:** Varenicline (0.5 once daily for 3 d, 0.5 twice a day for 4 d, 1 mg twice a day for 12 wk)<br><br>**Comparator:** Placebo	**1° endpoint:**<br>• 4 wk continuous abstinence rate<br>• 9–12 wk CAR:<br>• 47% vs. 13.9% (OR: 6.11; 95% CI: 4.18–8.93; p<0.0001)<br><br>**Safety endpoint:**<br>• SAE 6.5% varenicline vs. 6.0 placebo<br>• No difference in psychiatric AEs<br>• Non-statistically different but higher rate CV events in varenicline 25 vs. 20	• 9–52 wk abstinence rate: 19.2 vs. 7.2% (OR: 3.14; 95% CI: 1.93–5.11; p<0.0001)<br>• FDA advisory: may increase risk of adverse cardiovascular events
Hennrikus D, et al. 2010(150)<br>21144971	**Aim:** To evaluate intensive tailored counseling intervention for smoking cessation in PAD pts<br><br>**Study type:** RCT<br><br>**Size:** n=124 pts	**Inclusion criteria:**<br>• Primary inclusion criteria were a Dx of lower extremity PAD (defined as at least 1 of the following:<br>• An ABI of <0.90 in at least 1 lower extremity;<br>• A TBI of <0.60.<br>• Objective evidence of arterial occlusive disease in 1 lower extremity by duplex ultrasonography, MRA, or CTA<br>• Prior leg arterial revascularization or amputation due to PAD<br>• Current smoking (defined as smoking ≥1 cigarette a day ≥6 d per wk).<br>• Additional inclusion criteria included a desire to quit within the next 30 d	**Intervention:** Clinician advice, smoking counselor, individualized letter, motivational interview, info about pharmacologic intervention<br><br>**Comparator:** Verbal advice, list of programs	**1° endpoint:** 6 mo biologically confirmed smoking cessation 21.3% vs. 6.8%; chi-square: 5.21; p=0.023	N/A

© American Heart Association, Inc. and American College of Cardiology Foundation

		• Age ≥18 y<br>• Ability to speak and write English<br>• No participation in a smoking cessation program in the past 30 d<br>• Consumption of <21 alcoholic drinks/wk.<br><br>**Exclusion criteria:** N/A			
Tonstad S et al. 2003(151)<br>12714026	**Aim:** Buproprion SR in established CVD<br><br>**Study type:** RCT<br><br>**Size:** n=629 pts	**Inclusion criteria:**<br>• CAD<br>• PAD (33%)<br>• HF (Class I or II)<br>• Adults who smoke average ≥10 cigarettes/d during previous 12 mo without quit attempt in previous 3 mo.<br><br>**Exclusion criteria:**<br>• Seizure<br>• Renal/hepatic/heme/pulmonary neurologic disease<br>• Psychosis<br>• Depression	**Intervention:** 7 wk buproprion 150/d 1–2, then 150bid<br><br>**Comparator:** Placebo	**1° endpoint:** 4 wk smoking cessation<br>43% vs. 19% (OR: 3.27; 95% CI: 2.24–4.84)	N/A
Stead LF, et al. 2013(152)<br>23728631	**Study type:** Meta-analysis<br><br>**Size:** n=42 trials; 31,000 pts	**Inclusion criteria:**<br>• Trials between 1972–2012<br>• Trials of smoking interventions involving clinicians<br><br>**Exclusion criteria:** N/A	**Intervention:** Smoking cessation advice<br><br>**Comparator:** N/A	**1° endpoint:**<br>• Brief advice RR: 1.66; 95% CI: 1.42–1.94<br>• Intensive RR: 1.84; 95% CI: 1.60–2.13	• Simple advice has a small effect on cessation rates
Prochaska JJ and Hilton JF 2012(153)<br>22563098	**Study type:** Meta-analysis<br><br>**Size:** n=22 trials	**Inclusion criteria:**<br>• RCT adults with varenicline vs. placebo<br>• 2 with active CVD, 11 with Hx CVD<br><br>**Exclusion criteria:** N/A	**Intervention:** Varenicline<br><br>**Comparator:** Placebo	**1° endpoint:** CV events during drug treatment or within 30 d of discontinuation<br><br>**Results:** RR: 1.40; 95% CI: 0.82–2.39; p=0.22	• Risk of cardiovascular SAE with varenicline use: meta-analysis
Mills EJ et al. 2014(154)<br>24323793	**Study type:** Meta-analysis<br><br>**Size:** n=63 RCT	**Inclusion criteria:** RCT of NRT, bupropion, and varenicline that reported CVD outcome<br><br>**Exclusion criteria:** N/A	**Intervention:** NRT, bupropion, or varenicline<br><br>**Comparator:** N/A	**1° endpoint:**<br>• All CVD and MACE<br>• NRT: RR 1.81; 95% CI: 1.35–2.43<br>• Buproprion: RR: 1.03; 95% CI: 0.71–1.50<br>• Varenicline: RR: 1.24; 95% CI:	N/A

© American Heart Association, Inc. and American College of Cardiology Foundation

				0.85–1.81	

AE indicates adverse event; CAD, coronary arterial disease; CAR, continuous abstinence rate; CHF, congestive heart failure; CI, confidence interval; COPD, chronic obstructive pulmonary disease; CTA, computed tomography angiography; CVD, cardiovascular disease; CV, cardiovascular; FDA, Food and Drug Administration; GI, gastrointestinal; LOS, length of stay; MACE, major adverse cardiovascular event; MRA, magnetic resonance angiogram; N/A, not applicable; NNT, number needed to treat; NRT, nicotine replacement therapy; OR, odds ratio; PAD, peripheral artery disease; pt, patient; RCT, randomized controlled trial; RR, relative risk; and SAE, serious adverse event.

Evidence Table 20. Nonrandomized Trials, Observational Studies, and/or Registries of Smoking Cessation–Section 5.4.

Study Acronym; Author; Year Published	Study Type/Design; Study Size	Patient Population	Primary Endpoint and Results (include P value; OR or RR; & 95% CI)	Summary/Conclusion Comment(s)
Clair C, et al.<br>2013(155)<br>23483176	**Study type**: Prospective cohort. To investigate the impact of weight gain on the effect of smoking cessation on cardiovascular events<br><br>**Size**: n=3,251 pts, mean follow-up 25 y, 631 CVD events.	**Inclusion criteria**:<br>• Longitudinal cohort study 1984–2011.<br>• Self-reported smoking status: smoker, recent quitter (<4 y), long-term quitter >4 y, nonsmoker<br>• Stratified by DM<br><br>**Exclusion criteria**: Established CVD.	**1° endpoint**:<br>• CVD events (coronary heart disease, cerebrovascular disease, PAD, congestive heart failure).<br>• PAD events=73<br><br>**Results**:<br>No DM:<br>• Recent Quitters RR: 0.61; 95% CI: 0.21–1.78<br>• Long-term Quitters RR: 0.29; 95% CI: 0.16–0.52<br>DM:<br>• Recent Quitters RR: 0.36; 95% CI: 0.04–2.97<br>• Long-term Quitters RR: 0.42; 95% CI: 0.16–1.10	•Smoking cessation associated with lower CVD rates (including PAD) even when adjusting for weight gain.
**VSGNE**<br>Hoel AW, et al.<br>2013(156)<br>23375433	**Study type**: Registry<br><br>**Size**: n=7,807 pts	**Inclusion criteria**:<br>• CEA<br>• Carotid stent<br>• LE bypass<br>• AAA repair<br><br>**Exclusion criteria**:<br>• Lost to follow-up at 1 y<br>• Lack of smoking status at 1 y	**1° endpoint**: Self-reported smoking cessation at 1 y<br><br>**Results**:<br>• 46% pts post LE bypass quit at 1 y<br>Variability across treatment center in smoking cessation rates 28%–62%<br>• 78% of surgeons offered pharmacologic therapy or referral to smoking cessation program. Rates of cessation higher in these surgeons 48% vs. 33%	• Systems of care promote smoking cessation in pts with vascular disease<br>• High rates of smoking cessation after surgical procedures
**ACS/NSQIP**<br>Selvarajah S, et al.<br>2014(157)<br>24502815	**Study type**: Registry<br><br>**Size**: n=16,534 pts	**Inclusion criteria**:<br>• Infrainguinal bypass surgery<br>• Pre-operative smoking status	**1° endpoint**: 30 d graft failure<br><br>**Results**: Higher early graft failure in active smokers (OR: 1.21; 95% CI: 1.02–1.43; p=0.03)	• Active smoking associated with early graft failure.

© American Heart Association, Inc. and American College of Cardiology Foundation

		**Exclusion criteria**: N/A		
**UCSD**<br>Armstrong EJ, et al.<br>2014(158)<br>25282696	**Study type**: Retrospective cohort<br><br>**Size**: n=204 pts	**Inclusion criteria:**<br>• Peripheral angiography for claudication or CLI<br>• Active smoking at time of angiography<br>30% quit for 1 y<br><br>**Exclusion criteria**: N/A	**1° endpoint**: Amputation-free survival<br><br>**Results:**<br>• Smoking cessation associated with lower mortality 14% vs. 31% (HR: 0.40; 95% CI: 0.18–0.90<br>• Higher amputation-free survival 81% vs. 60% (HR: 0.43; 95% CI: 0.2–0.86)	• Smoking cessation associated with better outcomes in PAD.
**Scottish Family Health Study**<br>Lu L, et al<br>2013(159)<br>23880175	**Study Type**: Cross-sectional cohort study<br><br>**Size**: n=5,686 pts, 134 (2.4% with PAD defined by ABI)	**Inclusion criteria:**<br>• Never smokers<br>• Age ≥18 y<br><br>**Exclusion criteria**: N/A	**Results**: Second-hand smoke exposure (≥40 hrs/wk) higher prevalence PAD (OR: 5.56; 95% CI: 1.82–17.06; p=0.003)	No cotinine levels available, cross-sectional
Tan CE and Glantz SA<br>2012(160)<br>23109514	**Study Type**: Meta-analysis of impact of smoke-free laws with coronary, heart disease, cerebrovascular events<br><br>**Size**: n=45 studies of 33 smoke-free laws	**Inclusion criteria**: Studies published before November 30, 2011<br><br>**Exclusion criteria**: N/A	**Results**: Smoke-free legislation associated with lower hospital admission or death for: coronary events (RR: 0.84; 95% CI: 0.82–0.88), other heart disease (RR: 0.61; 95% CI: 0.44–0.85), cerebrovascular events (RR: 0.84; 95% CI: 0.75–0.94)	Did not ascertain PAD events

AAA indicates abdominal aortic aneurysm; ABI, ankle-brachial index; CEA, carotid endarterectomy; CLI, critical limb ischemia; CI, confidence interval; CVD, cardiovascular disease; DM, diabetes mellitus; HR, hazard ratio; LE, lower extremity; N/A, not applicable; OR, odds ratio; PAD, peripheral artery disease; and RR, relative risk.

Evidence Table 21. RCTs Evaluating Glycemic Control in Patients with PAD and Diabetes Mellitus–Section 5.5.

Study Acronym; Author; Year Published	Aim of Study; Study Type; Study Size (N)	Patient Population	Study Intervention (# patients) / Study Comparator (# patients)	Endpoint Results (Absolute Event Rates, P value; OR or RR; & 95% CI)	Relevant 2° Endpoint (if any); Study Limitations; Adverse Events

© American Heart Association, Inc. and American College of Cardiology Foundation

**PROACTIVE**<br>Dormandy JA et al.<br>2005(161)<br>16214598	**Aim**: To ascertain whether pioglitazone reduces macrovascular morbidty and mortality in high-risk pts with type 2 DM<br><br>**Study type**: Double blind, placebo controlled randomized trial<br><br>**Size**:<br>• n=5,238 pts<br>• PAD subgroup ~20% n=1,043 (reported as 1,274 in 2009 PAD subset publication)	**Inclusion criteria**:<br>• Pts with DM<br>• Age 35–75 y<br>• HgB A1c >6.5% despite treatment with diet or oral agents (with or without insulin).<br>• Evidence of "extensive macrovascular disease" CAD or stroke or "objective arterial disease in the leg" (PAD)<br>• PAD defined as major amputation or claudication+ABI <0.9<br><br>**Exclusion criteria**:<br>• Type I DM<br>• Pt only on insulin<br>• Planned coronary/peripheral revascularization<br>• NYHA CHF class II or above<br>• CLI excluded (rest pain, ischemic ulcer, gangrene)<br>• CKD on dialysis<br>• Abnormal ALT (> 2.5 x ULN)	**Intervention**: Oral pioglitazone (15 mg qd mo 1; 30 mg mo 2; 45 qd mo 3-end; medication could be adjusted if needed)<br><br>**Comparator**: Placebo	**1° endpoint**: Composite all-cause mortality, nonfatal MI, stroke, ACS, coronary or peripheral revascularization, major amputation<br><br>Average follow-up 34.5 mo.<br><br>**1° endpoint**: HR: 0.90; 95% CI: 0.80–1.02; p=0.095<br><br>**Safety endpoint**: No difference in CHF admissions or death due to CHF between pioglitazone and placebo groups	**2° endpoint**:<br>• All-cause mortality, non–fatal MI, stroke HR: 0.84; 95% CI: 0.72–0.98; p=0.027<br>• Subgroup analysis for PAD not reported.<br><br>**Summary**:<br>• Primary endpoint was negative, but secondary endpoint (primary for most studies of MACE) positive for reduction in events with pioglitazone vs. placebo; no PAD specific data presented, though 20% of pt population had sx PAD<br>• PAD substudy (2009 publication): PAD subset had higher event rates than non-PAD subset. In subset of pts enrolled with PAD (N=1,274 reported), there was no benefit of pioglitazone on the primary or secondary endpoint with increased rate of LE revascularization in the pioglitazone vs. placebo groups (p=0.0077). In the subgroup of pts randomized WITHOUT PAD, there was a beneficial effect of pioglitazone seen.

ABI indicates ankle-brachial index; ACS, acute coronary syndrome; ALT, alanine aminotransferase; CAD, coronary artery disease; CHF, congestive heart failure; CI, confidence interval; CKD, chronic kidney disease; CLI, critical limb ischemia; DM, diabetes mellitus; HR, hazard ratio; HgB, hemoglobin; LE, lower extremity; MACE, medical adverse cardiac events; MI, myocardial infarction; NYHA, New York Heart Association; PAD, peripheral artery disease; pt, patient; RCT, randomized controlled trial, and ULN, upper limit of normal.

Evidence Table 22. Nonrandomized Trials, Observational Studies, and/or Registries of Glycemic Control–Section 5.5.

Study Acronym; Author; Year Published	Study Type/Design; Study Size	Patient Population	Primary Endpoint and Results (include P value; OR or RR; & 95% CI)	Summary/Conclusion Comment(s)
**PAD-UCD**<br>Singh S, et al.<br>2014(162)<br>24939930	**Study type**: Observational registry of pts undergoing interventional procedures for CLI or ALI at a single center	**Inclusion criteria**: Pts with PAD within a peripheral interventional registry with DM with CLI or ALI who underwent infrapopliteal intervention	**1° endpoint**: Patency of the target lesion<br><br>**Results**: Pts with peri-procedural FBG values below the median value of 144 mg/dL had improved primary patency at 1 yr (46% vs. 16%; HR: 1.82; p=0.005); association robust after adjustment for insulin use and	• Observational study provides some support for adequate peri-procedural glycemic control with revascularization for infrapopliteal lesions in pts with DM with ALI/CLI to prevent MALE, possibly patency

© American Heart Association, Inc. and American College of Cardiology Foundation

	**Size**: n=149 pts, 309 PTA procedures	during the study period<br><br>**Exclusion criteria**: No FBG on day of angiogram procedure or within 2 d of the procedure	lesion characteristics<br><br>One yr major adverse limb events lower for pts with FBG below median (23% vs. 35%; p=0.05)	of PTA sites
Takahara M, et al. 2010(163) 20843974	**Study type**: Observational cohort study vs. retrospective chart review (study design not clear) at a single center<br><br>**Size**: n=278 pts; 197 pts with DM	**Inclusion criteria**: Pts with PAD undergoing PTA for CLI including pts with and without DMs<br><br>**Exclusion criteria**: Pts with CLI who were not candidates for PTA and treated by other means	**1° endpoint**: Major amputation, mortality (all-cause)<br><br>**Results**: Average follow-up 90±72 wk.<br><br>Among 287 CLI pts with DM:<br>HgB A1c level not associated with increased mortality<br><br>HgBA1c level associated with major amputation, adjusted HR: 1.349 per 1% increment; 95% CI: 1.103–1.650; p=0.004)<br><br>Association was robust after MV adjustment for other factors.<br><br>Increased quartiles of HgB A1C had stepwise increase in risk for major amputation, adjust HRs (for Fontaine Stage IV, dialysis, infection)<br>Quartile Adjusted HR<br>Q1 ≤5.9% -<br>Q2 6–6.7% 2.030 (0.657-6.266, p NS)<br>Q3 6.8–7.6% 3.398 (1.227-9.412, p=0.019)<br>Q4 ≥7.7% 3.983 (1.398-11.35, p=0.010)	• Another observational study providing some support for adequate glycemic control among PAD pts with DM with CLI who will undergo revascularization (pre-procedural HgB A1c) to reduce risk of amputation---association more pronounced for highest quartile of HgB A1c vs. lowest quartile.<br>• No mortality benefit seen over a relatively short period of follow-up
**Strong Heart Study** Resnick HE, et al. 2004(164) 14970108	**Study type**: Observational cohort study<br><br>**Size**: n=4,549 in entire cohort; 1,974 with DM without prior lower extremity amputation	**Inclusion criteria**: Native Americans age 45–74 y seen for baseline examination 1989–1992 and subsequent follow-up visits<br><br>**Exclusion criteria**: Pts without DM; those with prior LE amputation excluded	**1° endpoint**: Incident lower extremity amputation<br><br>**Results**: After average 8 yr follow-up. Among pts with PAD (ABI <0.9), higher HgB A1c increased odds of lower extremity amputation. Relationship also seen among pts with normal ABI and those with non-compressible vessels (ABI >1.4).<br><br>Odds of incident LE amputation among pts with DM and PAD (ABI <0.9) or non-compressible vessels (ABI ≤1.4); reference pts with DM with normal ABI and HgB A1c <6.5%* (OR=1)	• Epidemiological cohort study providing evidence of an association between HgBA1c/glycemic control and risk of LE amputation among pts with DM with PAD and also those with non compressible vessels (most of whom have PAD when assessed by other means)

© American Heart Association, Inc. and American College of Cardiology Foundation

			Pts with DM with PAD ABI <0.9<br>HgB A1c Age adjusted OR LE amp<br><6.5% 1.7<br>6.5-9.5% 5.6 (p<0.05)<br>>9.5% 8.7 (p<0.05)<br><br>Pts with DM with n/c vessels ABI >1.4<br>HgB A1c Age adjusted OR LE amp<br><6.5% 2.6<br>6.5-9.5% 7.5 (p<0.05)<br>>9.5% 10.4 (p<0.05)	

ABI indicates ankle-brachial index; ALI, acute limb ischemia; CI indicates confidence interval; CLI, critical limb ischemia; DM, diabetes mellitus; FBG, fasting blood glucose; HgbA1c, hemoglobin A1c; HR, hazard ratio; LE, lower extremity; MALE, major adverse limb event; MV, multivariate; NS, non-significant; OR, odds ratio; PAD, peripheral artery disease; PTA, percutaneous transluminal angioplasty; pt, patient; and RR, relative risk.

Evidence Table 23. RCTs Evaluating Oral Anticoagulation–Section 5.6.

Study Acronym;<br>Author;<br>Year Published	Aim of Study;<br>Study Type;<br>Study Size (N)	Patient Population	Study Intervention (# patients) /<br>Study Comparator (# patients)	Endpoint Results (Absolute Event Rates, P value; OR or RR; & 95% CI)	Relevant 2° Endpoint (if any);<br>Study Limitations;<br>Adverse Events
**WAVE TRIAL**<br>Anand S, et al.<br>2007(165)<br>17634457	**Aim**: Evaluate anticoagulant agents in prevention of cardiovascular complications in pts with PAD<br><br>**Study type**: RCT<br><br>**Size**: n=2,161 pts	**Inclusion criteria**:<br>• Age 35–85 y<br>• PAD defined as atherosclerosis of the arteries of the lower extremities, the carotid arteries, or the subclavian arteries<br><br>**Exclusion criteria**:<br>• Indication for oral anticoagulant treatment<br>• Actively bleeding or at high risk for bleeding<br>• Stroke within 6 mo before enrollment<br>• Dialysis	**Intervention**: Anticoagulation and antiplatelet<br><br>**Comparator**: Antiplatelet alone	**1° endpoint**: MI, stroke, or death no difference (12.2% vs. 13.3%, p=0.48)<br><br>**1° Safety endpoint**: Life threatening bleeding significantly increased (4.0% vs. 1.2%, p<0.0001)	• Mean follow-up 35 mo<br><br>**Summary**:<br>• Combination of an anticoagulant and antiplatelet therapy not more effective than antiplatelet therapy alone in preventing major cardiovascular complications and associated with increase in life-threatening bleeding

© American Heart Association, Inc. and American College of Cardiology Foundation

**BOA TRIAL** 2000(166) 10665553	**Aim**: Compare effectiveness of oral anticoagulants with ASA in prevention infrainguinal bypass-graft occlusion and clinical events<br><br>**Study type**: RCT<br><br>**Size**: n=2,690 pts	**Inclusion criteria**: Infrainguinal bypass for PAD<br><br>**Exclusion criteria**:<br>• Contraindication to trial medications<br>• Shortened life expectancy<br>• MI or stroke 1 mo before surgery<br>• Abnormalities of platelets<br>• Anemia	**Intervention**: Warfarin<br><br>**Comparator**: ASA	**1° endpoint**:<br>• Graft occlusion no difference<br>• Vascular death, MI, stroke, or amputation no difference<br><br>**Safety endpoint**: Bleeding increased (HR: 1.96; 95% CI: 1.42–2.71)	• Mean follow-up 21 mo<br>• Vein graft subset-benefit to anticoagulation<br><br>**Summary**:<br>• No difference other than in vein graft subgroup analysis and increased bleeding complications
Johnson WC and Williford WO 2002(167) 11877686	**Aim**: Evaluate warfarin + ASA therapy) vs. ASA alone on mortality, morbidity and bypass patency<br><br>**Study type**: RCT<br><br>**Size**: n=831 pts	**Inclusion criteria**: Any bypass for PAD<br><br>**Exclusion criteria**: Contraindication to ASA or warfarin	**Intervention**: Anticoagulation and antiplatelet<br><br>**Comparator**: Antiplatelet alone	**1° endpoint**:<br>• Bypass patency no significant difference<br>• 6 mm PTFE bypass subgroup analysis significant benefit (71% vs. 58%; p=0.02)<br><br>**Safety endpoint**:<br>• Mortality increased (32% vs. 23%; p=0.0001)<br>• Major hemorrhage increased (p=0.02)	•1/3 of anticoagulation pts stopped anticoagulation<br><br>**Summary**:<br>• Anticoagulation + ASA compared to ASA no difference in overall patency but increased mortality and major hemorrhage.<br>• Benefit in subgroup analysis of patency for 6 mm PTFE.
Sarac TP, et al. 1998(168) 9737454	**Aim**: Effects of anticoagulation therapy after autogenous vein bypass on duration of patency, limb salvage rates, and complication rates<br><br>**Study type**: RCT<br><br>**Size**: n=64 pts	**Inclusion criteria**: Infrainguinal vein bypass high risk for graft occlusion<br><br>**Exclusion criteria**: N/A	**Intervention**: Warfarin and ASA<br><br>**Comparator**: ASA alone	**1° endpoint**:<br>• 3 y patency improved (PP: 74% vs. 51%, p=0.04; PAP: 77% vs. 56%, p=0.5; SP: 81% vs. 56%, p=0.2)<br>• 3 y limb salvage improved (81% vs. 31%; p=0.01)<br>• Survival no difference<br><br>**Safety endpoint**:<br>• Postop hematoma increased (32% vs. 3.7%, p=0.004)<br>• No difference in RBC transfusions	•Small study<br>•Definition of high risk for bypass failure unclear<br>•Did not evaluate stroke, MI<br><br>**Summary**:<br>• Anticoagulation after vein bypass increases the incidence of wound hematomas, but improves patency rate and limb salvage.

© American Heart Association, Inc. and American College of Cardiology Foundation

Antonicelli R, et al. 1999(169) 10492316	**Aim**: Evaluate the efficacy of low-dose, subcutaneous calcium-heparin in comparison with placebo in pts with IC<br><br>**Study type**: RCT<br><br>**Size**: n=201 pts	**Inclusion criteria**:<br>• Willingness to use parenteral therapy<br>• ≥6 mo Hx of IC who had PAD confirmed by Doppler examination<br><br>**Exclusion criteria**: N/A	**Intervention**: Subcutaneous heparin and ASA<br><br>**Comparator**: ASA alone	**1° endpoint**:<br>• Maximum walking time 40% in heparin group and 16% in placebo group (p=0.05)<br>• Pain-free walking time 39% in heparin group and 23% in placebo group (p=0.09).	• 132 of 201 randomized pts completed the study<br><br>**Summary**:<br>• Treatment with low-dose subcutaneous heparin is safe and effective in improving walking performance

ASA indicates acetylsalicylic acid; CI, confidence interval; HR, hazard ratio; IC, intermittent claudication; MI, myocardial infarction; N/A, not applicable; PAD, peripheral artery disease; PTFE, polytetrafluoroethylene; pt, patient; and RCT, randomized controlled trial.

Evidence Table 24. Nonrandomized Trials, Observational Studies, and/or Registries of Oral Anticoagulation–Section 5.6.

Study Acronym; Author; Year Published	Study Type/Design; Study Size	Patient Population	Primary Endpoint and Results (include P value; OR or RR; & 95% CI)	Summary/Conclusion Comment(s)
Alonso-Coello P, et al. 2012(170) 22315275	**Study type**: Clinical practice guidelines based on meta-analysis of 3 RCTs evaluating warfarin + ASA vs. ASA alone.<br><br>**Size**: n=3,048 pts	**Inclusion criteria**:<br>• Asx PAD<br>• Sx PAD<br>• ALI<br>• Post peripheral arterial revascularization<br>• Carotid stenosis<br><br>**Exclusion criteria**: N/A	**1° endpoint**:<br>• Prevention of cardiovascular disease<br>• Relief of lower extremity symptoms and critical ischemia<br><br>**Results:** Results failed to demonstrate or exclude an effect of warfarin + ASA vs. ASA alone on mortality, nonfatal MI, or nonfatal stroke. However, there was a significant increase in major bleeding events with warfarin.	• Recommend against the use of warfarin + ASA in pts with asx or sx PAD (Grade 1B)
Bedenis R, et al. 2015(171) 25695213	**Study type**: Cochrane Review<br><br>**Size**: n=1,381 pts in the 3 studies included for the analysis of anticoagulants.	**Inclusion criteria**: Lower extremity bypass for PAD<br><br>**Exclusion criteria**: N/A	**1° endpoint**: Bypass primary patency<br><br>**Results:** No difference in primary graft patency when ASA or ASA with dipyridamole was compared to a vitamin K antagonist	• No patency benefit with use of anticoagulation
Cosmi B, et al. 2001(172) 11687006	**Study type**: Cochrane Review<br><br>**Size**: n=3 studies in the primary analysis; 4	**Inclusion criteria**: IC, RCT data<br><br>**Exclusion criteria**: N/A	**1° endpoint**:<br>• Maximum walking distance<br>• Pain-free walking distance<br><br>**Results:** No benefit of heparin, LMWHs or oral	• No significant difference was observed between heparin treatment and control groups for pain-free walking distance or maximum walking distance at the end of treatment<br>• Major and minor bleeding events were

© American Heart Association, Inc. and American College of Cardiology Foundation

	additional studies were included in the sensitivity analysis		anticoagulants has been established for IC. An increased risk of major bleeding events has been observed especially with oral anticoagulants. The use of anticoagulants for IC cannot be recommended at this stage.	significantly more frequent in the group treated with oral anticoagulants compared to control, with a nonsignificant increase in fatal bleeding events.

ALI indicates acute limb ischemia; ASA, aspirin; IC, intermittent claudication; LMWH, low molecular weight heparin; N/A, not applicable; PAD, peripheral arterial disease; pt, patient; and RCT, randomized controlled trial.

Evidence Table 25. RCTs and Observational Studies of Cilostazol–Section 5.7.

Study Author; Year Published	Aim of Study; Study Type; Study Size (N)	Patient Population	Study Intervention (# patients) / Study Comparator (# patients)	Endpoint Results (Absolute Event Rates, P value; OR or RR; & 95% CI)	Relevant 2° Endpoint (if any); Study Limitations; Adverse Events
Bedenis R, et al. 2014 (173) 25358850	**Aim:** To determine Cilostazol's impact on claudication walking distances, mortality, and vascular events in pts with stable IC.<br><br>**Study type:** Meta-analysis: Double-blind, RCTs of cilostazol vs. placebo, or vs. other antiplatelet agents in pts with stable IC.<br><br>**Size:**<br>• n=15 studies.<br>• n=3,718 pts	**Inclusion criteria:** Cilostazol with placebo, or medications currently known to increase walking distance e.g. pentoxifylline. All pts had IC secondary to PAD.	All included studies compared cilostazol 100mg 2x/d with placebo. In addition, 2 studies compared cilostazol 50 mg 2x/d with placebo, and 1 study compared cilostazol 150 mg 2x/d with placebo. 3 studies compared cilostazol 100 mg 2x/d with pentoxifylline 400 mg 3x/d. 1 study compared cilostazol 100 mg 2x/d with pentoxifylline 600 mg 2x/d and 1 study compared cilostazol 100 mg 2x/d with the antiplateletK-134 50 mg and 100mg 2x/d	For 8 studies data were compatible for comparison by meta-analysis, but data for 7 studies were too heterogeneous to be pooled. For the studies included in the meta-analysis, for ICD there was an improvement in the cilostazol group for the 100 mg and 50 mg 2x/d, compared with placebo (WMD: 31.41 meters; 95% CI: 22.38–40.45 meters; p<0.00001) and (WMD: 19.89 meters; 95% CI: 9.44–30.34 meters; p=0.0002), respectively. ICD was improved in the cilostazol group for the comparison of cilostazol 150 mg vs. placebo and cilostazol 100 mg vs. pentoxifylline, but only single studies were used for these analyses. ACD was significantly increased in pts taking cilostazol 100 mg and 50 mg 2x/d, compared with placebo (WMD: 43.12 meters; 95% CI: 18.28–67.96 meters; p=0.0007) and (WMD: 32.00 meters; 95% CI: 14.17–49.83 meters; p=0.0004), respectively. As with ICD, ACD was increased in pts taking cilostazol 150 mg vs. placebo, but with only 1 study an association cannot be clearly determined. 2 studies comparing cilostazol to pentoxifylline had opposing findings, resulting in an imprecise CI (WMD: 13.42 meters (95% CI: -43.51 – 70.35 meters; p=0.64). ABI was lowered in the cilostazol 100 mg group compared with placebo (WMD: 0.06; 95% CI: 0.04–0.08; p<0.00001). The single study evaluating ABI	There was no association between treatment type and all-cause mortality for any of the treatment comparisons, but there were very few events, and therefore inadequately powered. In general cilostazol was associated with a higher odds of headache, diarrhea, abnormal stool, dizziness and palpitations

© American Heart Association, Inc. and American College of Cardiology Foundation

				for the comparison of cilostazol vs. pentoxifylline found no change in ABI.	
Dawson DL, et al.<br>2000 (174)<br>11063952	**Aim:** To determine evaluate the relative efficacy and safety of cilostazol and pentoxifylline.<br><br>**Study type:** Randomized, double-blind, placebo-controlled, multicenter trial.<br><br>**Size:** n=698 pts	**Inclusion criteria:**<br>• Moderate-to-severe claudication<br>• Baseline pain-free walking distance ≥53.6 m<br>• Baseline maximal walking distance ≤537.6 m<br><br>**Exclusion criteria:**<br>• Buerger's disease<br>• Critial ischemia (category II or III chronis lower extremity ischemia)<br>• Lower extremity arterial reconstruction (surgical or endovascular) or sympathectomy within 3 mo<br>• Prior use of cilostrazol	**Study intervention:** Pentoxifylline or cilostazol<br><br>**Comparator:** Placebo	**Primary endpoint:** Walking ability, measured by MWD.<br>• Cilostazol treatment resulted in greater MWD than both pentoxifylline and placebo at 24 wk (p<0.001).<br>• Pentoxifylline treatment resulted in no improvement in MWD compared to placebo<br><br>**Secondary endpoints:** PFWD and resting Doppler limb pressures<br>• At wk 4 and after, there was a greater improvement in PFWD with cilostazol treatment than placebo (p<0.01)<br>• There was no difference in PFWD with pentoxifylline treatment compared with placebo (p<0.05).	• Withdrawal rates due to adverse effects were similar among the cilostazol (16%) and the pentoxifylline treatments (19%)<br>• Adverse events were higher in the active treatment groups than in placebo (27% for cilostazol; 26% for pentoxifylline; 16% for placebo; p=0.006)<br>• Overall results have not shown clear evidence of an improvement in walking performance with pentoxifylline treatment.
Goldenberger NA, et al.<br>2012 (175)<br>22615190	**Aim:** To investigate the effect of cilostazol + l-carnitine vs. cilostazol alone on treadmill performance in IC.<br><br>**Secondary objectives:** To evaluate QoL measures and safety indices with the drug	**Inclusion criteria:** PAD pts with stable IC were randomized to either l-carnitine 1 g or matching placebo 2x/d, on a background of cilostazol.	145 pts met criteria for the mITT population and 120 pts for the per-protocol population. 74 L-carnitine/71 placebo.	In the mITT (n=145), the mean ln ratio in PWT was 0.241 for cilostazol/l-carnitine vs. 0.134 for cilostazol/placebo (p=0.076), corresponding to mean increases of 1.99 and 1.36 min, respectively. In the per-protocol population (n=120), the mean *ln* ratio in PWT was 0.267 for cilostazol/l-carnitine vs. 0.145 for cilostazol/placebo (p=0.048).	The per-protocol population, the mean *ln* ratio in PWT was significantly increased in the cilostazol/l-carnitine group vs. the cilostazol/placebo group (0.267 vs. 0.145, respectively; p=0.048). This represented an arithmetic mean increase in PWT of 39.2% from baseline to d 180 for cilostazol/l-carnitine, as compared to 21.5% for cilostazol/placebo.

© American Heart Association, Inc. and American College of Cardiology Foundation

	combination.<br><br>**Study type**: A multicenter, randomized, double-blind, placebo-controlled trial<br><br>**Size**: n=164 pts				In the cilostazol/l-carnitine group, the mean increase in physical functioning on the SF-36v2 was also nearly double that of the cilostazol/placebo group (6.77 [16.379] vs. 3.73 [17.566], respectively; p=0.066).
Warner CJ, et al.<br>2014 (176)<br>24468286	**Aim**: MEDLINE (1946-2012), and Cochrane CENTRAL (1996-2012), and trial registries searched for studies comparing cilostazol in combination with antiplatelet therapy to antiplatelet therapy alone after PVI.<br><br>**Study type**: Meta-analysis:<br><br>**Size**: n=1,522 pts	**Inclusion criteria**:<br>• Pts undergoing endovascular treatment (angioplasty or stenting) for infrainguinal LE PVD.<br>• The intervention must be cilostazol in the periprocedural setting.<br>• The comparison group may be no cilostazol, an antiplatelet medication, or placebo.<br>• ≥6 mo follow-up<br>• The study reported at ≥1 pre-specified outcome of interest (restenosis, freedom from amputation, mortality).	2 RCTs and 4 retrospective cohorts met inclusion criteria. 1,522 pts included in the review.<br>A majority (87%) were from retrospective cohort studies.<br>All studies were conducted in Japan and published between 2008–2012.<br>All compared cilostazol with either no cilostazol (n=4) or an alternative antiplatelet medication (n=2), with both groups receiving various co-interventions (ASA with or without an adjunct antiplatelet medication).	The addition of cilostazol was associated with decreased restenosis (RR: 0.71; 95% CI: 0.60–0.84; p<0.001), improved amputation-free survival (HR: 0.63; 95% CI: 0.47–0.85; p=0.002), improved limb salvage (HR: 0.42; 95% CI: 0.27–0.66; p<0.001), and improved freedom from target lesion revascularization (RR: 1.36; 95% CI: 1.14–1.61; p<0.001).	There was no significant reduction in mortality among those receiving cilostazol (RR: 0.73; 95% CI: 0.45–1.19; p=0.21).

© American Heart Association, Inc. and American College of Cardiology Foundation

**STOP-IC**<br>Iida O, et al.<br>2013 (177)<br>23652861	**Aim**: To determine by angiographic follow-up whether treatment with cilostazol reduces restenosis at 12 mo after PTA with provisional nitinol stenting for femoropopliteal disease<br><br>**Study type**:<br><br>**Size**: n=152 pts: 75 in cilostazol/77 placebo	**Inclusion criteria**: Within 1 wk after randomization, each pt was admitted and underwent PTA with provisional nitinol stenting.	**Study intervention**: 75 in cilostazol<br><br>**Study comparator**: 77 placebo	**Results**: During the12 mo follow-up period, 11 pts died and 152 pts (80%) had evaluable angiographic data at 12 mo. The angiographic restenosis rate at 12 mo was 20% (15/75) in the cilostazol group vs. 49% (38/77) in the noncilostazol group (p=0.0001) by ITT analysis.	The cilostazol group also had a significantly higher event-free survival at 12 mo (83% vs. 71%, p=0.02), although cardiovascular event rates were similar in both groups.

ABI indicates ankle-brachial index; ACD, absolute claudication distance; CI, confidence interval; HR, hazard ratio; IC, intermittent claudication; ICD, initial claudication distance; ITT, intent-to-treat; LE, lower extremity; mITT, modified intent-to-treat; MWD, maximal walking distance; PAD, peripheral artery disease; PFWD, pain free walking distance; PTA, percutaneous transluminal angioplasty; PVD, peripheral vascular disease; PVI, peripheral vascular intervention; PWT, peak walking time; RCT, randomized controlled trial; and PTFE, polytetrafluoroethylene; pt, patient; QoL, quality of life; RCT, randomized controlled trial; RR, relative risk; and WMD, walking maximal distance.

Evidence Table 26. Nonrandomized Trials, Observational Studies, and/or Registries of Pentoxifylline–Section 5.8.

Study Acronym; Author; Year Published	Aim of Study; Study Type; Study Size (N)	Patient Population	Study Intervention (# patients) / Study Comparator (# patients)	Endpoint Results (Absolute Event Rates, P value; OR or RR; & 95% CI)	Relevant 2° Endpoint (if any); Study Limitations; Adverse Events

© American Heart Association, Inc. and American College of Cardiology Foundation

Salhiyyah K, et al.<br>2015 (178)<br>22258961	**Aim:** To determine the efficacy of pentoxifylline in improving the walking capacity of pts with stable IC<br><br>**Study type:** Cochrane review<br><br>**Size:** n=24 studies with 3,377 pts (Current until April 2015)	**Inclusion criteria:**<br>• Double blind RCTs comparing pentoxifylline vs. placebo or any other pharmacological intervention<br>• Symptoms of stable IC<br>• Fontaine stage II due to PAD<br><br>**Exclusion criteria:** Pts with critical ischemia or had previously undergone surgical or percutaneous procedures	• 17 studies compared pentoxifylline with placebo<br>• 1 study compared pentoxifylline with flunarizine<br>• 1 study compared pentoxifylline with aspirin<br>• 1 study compared pentoxifylline with GBE<br>• 1 study compared pentoxifylline with nylidrin hydrochloride<br>• 2 studies compared pentoxifylline with PGE1<br>• 1 study compared pentoxifylline with nifedipine<br>• 2 studies compared pentoxifylline with cilostazol and placebo<br>• 1 study compared pentoxifylline with iloprost and placebo	The difference in percentage improvement in TWD for pentoxifylline over placebo ranged from 1.2%–155.9%, and for PFWD the difference ranged from -33.8% – 73.9%Testing for statistical significance of these results was generally not possible due to the lack of data.	• There was no statistically significant difference in ABI between the pentoxifylline and placebo groups.<br>• Pentoxifylline was generally well tolerated.
			**Study intervention:** Pentoxifylline<br><br>**Comparator:** Placebo	• Large variability in results. Unable to perform meta-analysis because of variability.<br>• PFWD (11 studies): -33.8%– 73.9% with pentoxifylline<br>• TWD (14 studies): 1%–155.9% with pentoxifylline<br>• QoL – SF-36 (3 studies): 2 studies showed not difference, one study showed a significant improvement in QoL.	N/A
			**Study intervention:** Pentoxifylline<br><br>**Comparator**: Active agents	• Pentoxifylline showed a larger improvement in PFWD when compared with GBE (1 study), buflomedil (1 study) and iloprost (1 study).<br>• Cilostazol (2 studies) and PGE1 (2 study) showed a larger improvement in PFWD compared with pentoxifylline.<br>• For TWD a larger improvement was shown for Pentoxifylline showed a larger improvement in TWD when compared with nylidrin, GBE and ASA. Cilostazol, PGE1 and flunarizine showed larger improvements in TWD compared with pentoxifylline.<br>• Pentoxifylline appeared to be well tolerated in most	N/A

© American Heart Association, Inc. and American College of Cardiology Foundation

ABI indicates ankle-brachial index; GBE, ginkgo biloba extract; IC, intermittent claudication; PAD, peripheral artery disease; PFWD, pain free walking distance; PGE1, prostaglandin E1; pt, patient; QoL, quality of life; and TWD, total walking distance.

Evidence Table 27. Systematic Review of Chelation Therapy–Section 5.9.

Study Acronym; Author; Year Published	Aim of Study; Study Type; Study Size (N)	Patient Population	Study Intervention (# patients) / Study Comparator (# patients)	Endpoint Results (Absolute Event Rates, P value; OR or RR; & 95% CI)	Relevant 2° Endpoint (if any); Study Limitations; Adverse Events
Villarruz MV, et al. 2008(179) [12519577]	**Aim**: To assess the effects of EDTA chelation on clinical outcomes among people with atherosclerotic CV disease:<br><br>**Study type**: Systematic review	**Inclusion criteria**: Pts with PVD, particularly those with IC	7 publications representing 5 trials.	• WMD in ABI: 0.01; 95% CI: -0.03 – 0.06.<br>• WMD for walking distance: -37.93; 95% CI: -90.32 – 0.06<br>• WMD for PFWD post-treatment: -7.73; 95% CI: -22.59 – 7.13	• Side effects: Faintness: RR: 11.44; 95% CI: 1.51–86.45<br>• Gastrointestinal symptoms RR: 1.63; 95% CI: 0.67–3.99<br>• Proteinuria RR: 2.60; 95% CI: 0.85–7.93<br>• Hypocalcemia RR: 3.12; 95% CI: 0.65–14.98

ABI indicates ankle-brachial index; EDTA, ethylene diamine tetraacetic acid; CI, confidence interval; HR, hazard ratio; IC, intermittent claudication; N/A, not applicable; PFWD, pain free walking distance; pt, patient; PVD, peripheral vascular disease; RR, relative risk; and WMD, weighted mean difference.

Evidence Table 28. Nonrandomized Trials, Observational Studies, and/or Registries of Homocysteine Lowering Therapy for Lower Extremity PAD in Patients with Diabetes Mellitus–Section 5.10.1.

Study Acronym; Author; Year Published	Study Type/Design; Study Size	Patient Population	Primary Endpoint and Results (include P value; OR or RR; & 95% CI)	Summary/Conclusion Comment(s)
Khandanpour N, et al. 2009 (180) [19560951]	**Study type**: Meta-analysis of observational studies and clinical trials<br><br>**Size**:<br>• n=14 studies included in meta-analysis (of 214 retrieved from databases)	**Inclusion criteria**:<br>• Reviewed MEDLINE, EMBASE, and Cochrane databases for studies published between 1950—December, 2007<br>• Observational meta-analysis: studies with measurement of plasma homocysteine levels in PAD pts and non-PAD controls<br>• Clinical trial meta-analysis: Trials for which PAD pts with treated with single or combined vitamin therapy (folate, vitamin B6 and/or vitamin B12)<br>• PAD defined as ABI <0.9, IC, diminished	**1° endpoint**: Homocysteine levels in PAD pts vs. controls<br><br>**Results:**<br>• PAD pts had higher homocysteine levels than non-PAD controls<br>• Pooled mean difference vs. controls +4.31 micromol/L (95% CI: 1.71–6.31; p<0.0001)<br>• Mean plasma homocysteine levels higher in PAD pts than in controls in all 14 studies include in meta-analysis, though magnitude of difference varied across studies<br>• Clinical trial meta-analysis unable to be performed due to limited study quality and diverse outcomes reported. Among	• Homocysteine levels are elevated among PAD pts as compared to non-PAD controls<br>• Data lacking to make statement regarding benefit of homocysteine lowering therapy for clinical benefit in PAD

© American Heart Association, Inc. and American College of Cardiology Foundation

		pedal pulses + angiographically demonstrated PAD (obstruction of one at least major leg artery)<br><br>**Exclusion criteria:** Lack of non PAD control group, non-English studies, case reports, homocysteine levels not extractable, non-fasting or post-methionine loading homocysteine levels reported	8 clinical trials, 3 nonrandomized.<br>• All 8 studies demonstrated reduction in plasma homocysteine in folate/vitamin intervention groups<br>• One study in meta-analysis which reported on ABI and walking distance studied other nutritional supplements not homocysteine lowering vitamins alone.<br>• Studies reported other endpoints including endothelial function testing, inflammatory and other biomarkers	

ABI indicates ankle-brachial index; CI, confidence interval; IC, intermittent claudication; PAD, peripheral artery disease; and pt, patient.

Evidence Table 29. RCTs Comparing Additional Medical Therapies of Homocysteine Lowering Therapy for Lower Extremity PAD–Section 5.10.1.

Study Acronym; Author; Year Published	Aim of Study; Study Type; Study Size (N)	Patient Population	Study Intervention (# patients) / Study Comparator (# patients)	Endpoint Results (Absolute Event Rates, P value; OR or RR; & 95% CI)	Relevant 2° Endpoint (if any); Study Limitations; Adverse Events
**HOPE-2**<br>Lonn E, et al. 2006 (181)<br><br>16531613<br><br>**HOPE-2 Investigators**<br>Lonn E, et al. 2006(182)<br>16450017	**Aim:** Study effect of vitamin supplementation to lower homocysteine levels on risk of major CV events among pts with vascular disease<br><br>**Study type:** Double blind, placebo controlled randomized trial<br><br>**Size:**<br>• n=5,522 randomized pts with PAD<br>• n=133 claudication (2.4%)<br>• n=276 with PAD revascularization (5.0%)	**Inclusion criteria:**<br>• Age ≥55 y with documented CAD, PAD, cerebrovascular disease, or DM + at least 1 additional risk factor.<br>• PAD enrollment criteria were prior lower extremity revascularization (bypass or PTA), claudication with ABI ≤0.8, documented (leg) arterial stenosis ≥50% on angiography, prior ischemic limb or foot amputation<br><br>**Exclusion criteria:**<br>• Use of vitamin supplements with significant folic acid content<br>• Prior adverse reactions to folate/B6/B12<br>• Planned cardiac/peripheral vascular revascularization within 6 mo<br>• Significant non-atherosclerotic/athero-thrombotic cardiovascular disease<br>• Other non-cardiovascular comorbidities expected to limit	**Intervention:** Folic acid 2.5 mg/vitamin B6 50 mg/vitamin B12 1 mg in a combined pill<br><br>**Comparator:** Placebo	**1° endpoint:**<br>• No improvement in composite of death from CV cause, MI, and stroke with intervention<br>• Event rates 18.8% (intervention) vs. 19.8% (placebo); RR: 0.95; 95% CI: 0.84–1.07; p=0.41.<br>• "Average follow-up" 5 y<br><br>**Safety endpoint:** No SAEs related to study treatment.	• Homocysteine decreased in interventional arm and increased in placebo arm (-2.4 micromol/L vs. +0.8 micromol/L)<br>• No difference in risk of death between groups (RR: 0.96; 95% CI: 0.81–1.13)<br>• No difference in risk of MI between groups (RR: 0.989; 95% CI: 0.85–1.14)<br>• Decreased RR stroke among those randomized to intervention (RR: 0.75; 95% CI: 0.59–0.97).<br>• Increased RR risk of hospitalization with unstable angina among those randomized to intervention (RR: 1.24; 95% CI: 1.04–1.49)<br>• All other secondary outcomes with no difference in groups (including VTE, cancer)<br><br>**Summary:** Negative study; no overall CV benefit of homocysteine lowering therapy in this Westernized population study (US, Canada, Brazil, and Europe) which included a small subset of PAD pts.

© American Heart Association, Inc. and American College of Cardiology Foundation

		compliance or ability to complete study			

CAD indicates coronary artery disease; CI, confidence interval; CV, cardiovascular; HOPE, Heart Outcomes Prevention Evaluation; PAD, periphery artery disease; PTA, percutaneous transluminal angioplasty; pt, patient; RCT, randomized controlled trial; RR, relative risk; SAE, serious adverse event; US, United States; and VTE, venous thromboembolism.

Evidence Table 30. RCTs for Influenza Vaccination–Section 5.10.2.

Study Acronym; Author; Year Published	Aim of Study; Study Type; Study Size (N)	Patient Population	Study Intervention (# patients) / Study Comparator (# patients)	Endpoint Results (Absolute Event Rates, P value; OR or RR; & 95% CI)	Relevant 2° Endpoint (if any); Study Limitations; Adverse Events
**FLUVACS** Gurfinkel EP, et al. 2004 (183) 14683739	**Aim**: To test the effect of 1 yr benefit of influenza vaccination in pts with MI and planned PCI<br><br>**Study type**: RCT<br><br>**Size**: n=301 (200 MI pts and 101 PCI pts)	**Inclusion criteria**: MI pts or PCI pts<br><br>**Exclusion criteria**: Unstable CAD, prior by-bass surgery, angioplasty, or tissue necrosis	**Intervention**: Influenza vaccine (151)<br><br>**Comparator**: No vaccination ontop of standard medication (150)	**1° endpoint**: Time to first CVD<br>• At 6 mo: 2% in vaccinated intervention group vs. 8% CVD in unvaccinated controls (RR: 0.25; 95% CI: 0.07–0.86; p=0.01)<br>• At 1 yr: 6% in vaccinated intervention group vs. 17% CVD in unvaccinated controls. (RR: 0.34; 95% CI: 0.17–0.71; p=0.002)	Time to first composite triple endpoint of CVD, MI, and rehospitalization for severe recurrent ischemia at 1 yr was significantly decreased in the intervention group compared to control group (22% in vaccinated intervention group vs. 37% in unvaccinated control group; RR: 0.59; 95% CI: 0.4–0.86; p=0.004)<br>• Reduction in RR of CVD in vaccinated group at 1 y.<br>• No PAD specific evidence identified
**FLUCAD** Ciszewski A, et al. 2008 (184) 18187561	**Aim**: Determine effects of influenza vaccination on coronary events in pts with CAD<br><br>**Study type**: RCT<br><br>**Size**: n=658 treated CAD pts (477 men)	**Inclusion criteria**:<br>• Age 30–80 y<br>• CAD confirmed by angiography with ≥50% stenosis of ≥1 large epicardial coronary artery<br><br>**Exclusion criteria**: Congestive heart failure NYHA III/IV<br>• Planned CV surgery within 6mo<br>• Evolving renal failure<br>• Neoplastic disease<br>• Psycho-organic disorder or any factor impeding follow-up	**Intervention**: Influenza vaccine (325)<br><br>**Comparator**: Placebo (333)	**1° endpoint**: 1 yr CVD<br>• At 1 y: HR: 1.06; 95% CI: 0.15–7.56; p=0.95	**2° endpoint**:<br>• No difference between two groups for CVD, acute MI, or coronary revascularization<br>• At 1 y coronary ischemic events was decreased in intervention group compared to placebo control group (HR: 0.54; 95% CI: 0.29–0.99; p=0.047)<br><br>**Limitations**: Small sample size, effect of flu vaccination on restenosis is unknown, pt selection bias<br><br>• No PAD specific evidence identified

© American Heart Association, Inc. and American College of Cardiology Foundation

		• Contraindication to vaccination			

CAD indicates coronary artery disease; CI, confidence interval; CVD, cardiovascular death; CVD, cardiovascular disease; HR, hazard ratio; ITT, intention to treat; MI, myocardial infarction; NYHA, New York Heart Association; PCI, percutaneous intervention; pt, patient; RCT, randomized controlled trial; and RR, relative risk.

Evidence Table 31. Nonrandomized Trials for Influenza Vaccination–Section 5.10.2.

Study Acronym; Author; Year Published	Study Type/Design; Study Size	Patient Population	Primary Endpoint and Results (include P value; OR or RR; & 95% CI)	Summary/Conclusion Comment(s)
Davis MM, et al. 2006 (185) 17010820	**Study type**: Science Advisory Statement<br><br>**Size**: N/A	**Inclusion criteria**: Cohort , case control studies and RCTs<br><br>**Exclusion criteria**: N/A	• COR I LOE B recommendation to immunize with inactivated vaccine as part of comprehensive secondary prevention in persons with coronary and other atherosclerotic vascular disease.<br>• 1 RCT (FLUVACS) included<br>• Summary of observational cohort and case control studies demonstrating reduced CV event rates among pts with cardiovascular disease who received influenza vaccination	• Not recommended for persons with CV conditions to be immunized with live, attenuated vaccine.<br>• Immunization coverage levels are below national goals

COR indicates class of recommendation; CV, cardiovascular; LOE, level of evidence; N/A, not applicable; PCI, percutaneous coronary intervention; pt, patient; and RCT, randomized controlled trial.

Evidence Table 32. RCTs for Exercise Therapy–Section 6.

Study Acronym; Author; Year Published	Aim of Study; Study Type; Study Size (N)	Patient Population	Study Intervention (# patients) / Study Comparator (# patients)	Endpoint Results (Absolute Event Rates, P value; OR or RR; & 95% CI)	Relevant 2° Endpoint (if any); Study Limitations; Adverse Events
**CLEVER 18 mo F/U**<br>Murphy TP, et al. 2015 (186) 25766947	**Aim:** Report the longer-term (18 mo) efficacy of SE compared with ST and OMC included printed advice about exercise and diet. SE and ST pts also received	**Inclusion criteria:**<br>• Age >40 y<br>• oderate to severe IC due to aortoiliac PAD. IC defined as ability to walk ≥2 min on TM at 2 miles/hr at 0% grade but <11 min (about 5.5 METS maximum). ≥50%	**Intervention:** OMC, n=22; SE, n=44; ST, n=46. SE was supervised for 26 wk, 3 times/wk, 1 h for 6 mo followed by a telephone maintenance program through 18 mo during home-based exercise.	**1° endpoint:** PWT improved from baseline to 18 mo for both SE (5±5.4 min) and ST (3.2±4.7 min) more than OMC (0.2±2.1 min); p<0.001 and p<0.04, respectively. SE and ST did not differ.<br><br>**1° Safety endpoint:** All major	• At 18 mo, improvement in disease-specific scales (WIQ, PAQ) was statistically superior for ST and SE compared with OMC, but ST and SE differed significantly from each other (favoring ST) only for PAQ symptoms, PAQ treatment satisfaction, PAQ QoL, and PAQ summary<br>• Mean ABI values were normalized in

© American Heart Association, Inc. and American College of Cardiology Foundation

	OMC.<br><br>**Study type**: Long-term follow-up of RCT<br><br>**Size**: n=79 of 111 pts initially enrolled completed assessments at 18 mo.	• Stenosis of distal aorta or iliac arteries.<br><br>**Exclusion criteria**: CLI or 2 comorbid conditions that limited walking ability.	**Comparator**: N/A	adverse events occurred in first 6 mo and not in the follow-up. These included an MI in the OMC group; 1 death in SE group; and 1 target limb revascularization in the ST group.	the stented pts and changed by 0.00±0.1 for OMC, 0.2±0.2 for ST, and 0.00±0.1 for SE (p=0.002 for ST vs. OMC and p<0.001 for ST vs. SE)<br>• SE had the advantage of improved limb muscle strength, walking efficiency, and performance.
**CLEVER**<br>Murphy TP, et al. 2012 (187)<br>22090168	**Aim**: Compare the benefits OMC, SE, and ST on both walking outcomes and measures of QoL in pts with claudication due to aortoiliac PAD.<br><br>**Study type**: RCT<br><br>**Size**: n=111 pts	**Inclusion criteria**:<br>• Age >40 y<br>• Moderate to severe IC due to aortoiliac PAD. IC defined as ability to walk at least 2 min on TM at 2 miles/hr at 0% grade but <11 min (about 5.5 METS maximum).<br>• ≥50% stenosis of distal aorta or iliac arteries.<br><br>**Exclusion criteria**: CLI or 2 comorbid conditions that limited walking ability.	**Intervention**: OMC, n=22; SE, n=44; ST, n=46. SE was supervised for 26 wk, 3 times/wk, 1 hr for 6 mo. A ST/SE group was dropped after 8 pt to enhance enrollment in the other groups. Randomization ratio was 2:2:1 (ST:SE:OMC).<br><br>**Comparator**: N/A	**1° endpoint**: Compared with baseline, PWT improved by 1.2±2.6 min with OMC alone, 5.8±4.6 min with SE, and 3.7±4.9 min with ST. Compared with OMC alone, SE led to a greater mean improvement in PWT by 4.6 min (95% CI: 2.7–6.5; p<0.001), whereas ST had a somewhat smaller relative improvement in PWT of 2.5 min (95% CI: 0.6–4.4; p=0.022). A direct comparison of SE and ST showed a greater improvement in PWT with SE by a mean of 2.1 min (95% CI: 0.0–4.2; p=0.04)<br><br>**Safety endpoint**: 4 SAEs within 30 d of ST. SAEs noted in the 18 mo follow-up report that said they occurred in the first 6 mo were not mentioned.	• ABI improved by 0.29±0.33 in the ST group (p<0.0001) only.<br>• The greatest improvements in self-reported QoL were observed in the ST cohort despite greater increases in PWT in the SE group.
**GOALS**<br>McDermott MM, et al.<br>2013 (17)<br>23821089	**Aim**: Determine whether a home-based walking exercise program using a group-mediated cognitive	**Inclusion criteria**: Resting ABI ≤0.9 or ABI between 0.91–1 with a 20% drop after a heel-rise test or medical evidence of LE revascularization or	**Intervention**: Walking on-ground (not TM) progressing to 50 min 5 times/wk for 6 mo. For pts with IC, walk to pain level 4 of 5, rest, and resume. For	**1° endpoint**: Exercisers increased their 6 min walk distance (357.4–399.8 meters vs. 353.3–342.2 meters for those in the control group; mean difference: 53.5; 95% CI: 33.2–	• Maximal TM walking time (intervention, 7.91–9.44 min vs. control, 7.56–8.09; mean difference: 1.01 min; 95% CI: 0.07–1.95; p=0.04), accelerometer-measured physical activity over 7 ds (intervention, 778.0–866.1 vs. control,

© American Heart Association, Inc. and American College of Cardiology Foundation

	behavioral approach, can improve functional performance compared with a control group in pts with PAD with and without IC.<br><br>**Study type**: RCT<br><br>**Size**: n=194 pts	evidence of PAD.<br><br>**Exclusion criteria**: LE amputation, wheelchair confinement, inability to walk 50 ft, walking aid except cane, walking impairment other than PAD, surgery within past 3 mo, other major comorbidities that would preclude unsupervised exercise	pts without IC, walk at 12–14 on Borg RPE scale. Using a group-mediated cognitive behavioral approach, exercisers also met once a wk for 90 min.<br><br>**Comparator**: Health education control group that met weekly for 60 min to discuss general health topics.	73.8; p<0.001.<br><br>**Safety endpoint**: 1 exerciser developed dyspnea on exertion and subsequently required CABG and completed study after recovery.	671.6–645.0; mean difference: 114.7 activity units; 95% CI: 12.82–216.5; p=0.03), WIQ distance score (intervention, 35.3–47.4 vs. control, 33.3–34.4; mean difference: 11.1; 95% CI: 3.9–18.1; p=0.003), and WIQ speed score (intervention: 36.1–47.7 vs. control: 35.3–36.6; mean difference: 10.4; 95% CI: 3.4–17.4; p=0.004).<br>• 1 death from cancer among exercisers and 2 deaths from hypertensive CVD and CVD with pneumonia, all considered not study related.
**GOALS**<br>McDermott MM, et al.<br>2014 (188)<br>24850615	**Aim**: 6 mo intervention of walking vs. controls in pts with PAD with and without IC. This is a follow-up study at 12 mo, 6 mo after completing the 6 mo intervention<br><br>**Study type**: RCT<br><br>**Size**: Initial study enrolled 194 pts, of which 178 completed testing at 6 mo. At 12 mo, 168 completed follow-up testing	**Inclusion criteria**: Resting ABI ≤0.9 or ABI between 0.91–1 with a 20% drop after a heel-rise test or medical evidence of LE revascularization or evidence of PAD.<br><br>**Exclusion criteria**:<br>• LE amputation<br>• Wheelchair confinement<br>• Inability to walk 50 ft<br>• Walking aid except cane<br>• Walking impairment other than PAD<br>• Surgery within past 3 mo<br>• Other major comorbidities that would preclude unsupervised exercise	**Intervention**: During 6 mo phase, exercisers attended weekly group sessions, which included group-mediated cognitive behavioral techniques. During the next 6 mo, exercisers received call from their group facilitator and were encouraged to exercise and keep logs, which were sent back to study team.<br><br>**Comparator**: Controls received calls related to general health topics.	**1° endpoint**: Compared to controls, exercisers increased their 6 min walk distance from baseline to 12 mo follow-up, (from 355.4–381.9 m in the intervention vs. 353.1–345.6 m in the control group; mean difference: +34.1 m; 95% CI: 14.6–53.5; p<0.001)<br><br>**Safety endpoint**: No adverse events reported	• WIQ speed score increased (from 36.1–46.5 in exercisers vs. 34.9–36.5 in the control group; mean difference: +8.8; 95% CI: +1.6 – +16.1; p=0.018). Change in the WIQ distance score was not different between groups at 12 mo (p=0.139).<br>• No adverse events reported
Collins TC, et al.<br>2011 (189)<br>21873560	**Aim**: Determine the efficacy of a home-based walking intervention to improve walking ability and QoL in	**Inclusion criteria**:<br>• Age ≥40 y<br>• With PAD or prior surgery for PAD with continued IC<br>• Type 1 or 2 DM<br><br>**Exclusion criteria**:	**Intervention**: All pts in both groups received education about PAD and self-management behaviors for DM and CVD risk factors. Exercisers participated in a home-based routine	**1° endpoint**: The groups did not differ in 6 mo change in maximal treadmill walking distance average: 24.5; SE: 19.6 meters vs. maximal treadmill walking distance average: 39.2; SE: 19.6 meters; p=0.60.	• For the exercise and control groups, respectively, average walking speed scores increased by 5.7 (standard error: 2.2) units and decreased by 1.9 (standard error: 2.8) units (p=0.03); the mental health QoL subscale score of the SF-36 increased by 3.2 (standard error:

© American Heart Association, Inc. and American College of Cardiology Foundation

	people with DM and PAD<br><br>**Study type**: RCT<br><br>**Size**: n=145 pts	• No intention to exercise<br>• No telephone<br>• LE amputation<br>• CLI<br>• LE revascularization in past 6 mo<br>• MI within past 3 mo<br>• Comorbidities that would preclude participation in unsupervised exercise program	walking program for 3 d and 1 group exercise session per wk for 6 mo.<br><br>**Comparator**: Controls received twice monthly calls to discuss their health behaviors	**Safety endpoint**: No unanticipated adverse events in either group. Some events included general health issues, leg bypass surgery, broken hip, foot problems, and unable to complete treadmill testing but these were too few to ascertain group effects.	1.5) and decreased by 2.4 (standard error: 1.5) units (p=0.01).
Gardner AW, et al. 2011 (190)<br>21262997	**Aim**: Compare changes in exercise performance and daily ambulatory activity in PAD with IC after a home-based exercise program, a supervised exercise program, and usual-care control.<br><br>**Study type**: RCT<br><br>**Size**: n=119 pts	**Inclusion criteria**: Exertional leg pain, resting ABI ≤0.9 or ABI ≤.73 after exercise<br><br>**Exclusion criteria**: Inability to obtain ABI due to noncompressible vessels, asx PAD, use of cliostazol or pentoxifylline initiated within 3 mo before study, exercise limited by other causes, major comorbidities (active cancer, renal, or liver disease	**Intervention**: 12 wk. Home-based exercise of intermittent walking to near-maximal pain 3 d/wk at self-selected pace. Walking duration progressed from 20 min initially to 45 min during final 2 wk of program. Supervised program was performed on a treadmill with durations 5 min shorter than home-based program. Intensity set at 40% of peak workload from baseline exercise test, to near-maximal pain, rest, and resume exercise. Both groups used step activity monitors to measure walking.<br><br>**Comparator**: Non-exercise, usual care control	**1° endpoint**: Both exercise programs increased claudication onset time (p<0.001) and peak walking time (p<0.01). Controls did not change.<br><br>**Safety endpoint**: Not specified but though no unanticipated adverse events in either group. Events included stroke (2), leg revascularization (2), MI (1), and hernia surgery (1). These were too few to ascertain group effects.	• Home group only increased daily average cadence (p<0.01)
Saxton JM, et al. 2011 (191)<br>21215558	**Aim**: Compare the effects of upper- and lower-limb aerobic exercise training on	**Inclusion criteria**:<br>• PAD with IC by Hx<br>• ABI ≤0.9<br>• Symptoms 12 mo	**Intervention**: Arm cranking at 85%–90% of limb-specific maximal oxygen uptake, 2 d/wk for 24 wk, total time exercise time of	**1° endpoint**: After 6 wk, improvements in the perceived severity of claudication (p=0.023) and stair climbing ability (p=0.011) vs. controls	• At 48 and 72 wk, improvement in perceptions of walking distance were better maintained in upper limb group. Improvements in walking speed and stair climbing ability were similarly maintained

© American Heart Association, Inc. and American College of Cardiology Foundation

	disease-specific functional status and generic health-related QoL in pts with IC<br><br>**Study type**: RCT<br><br>**Size**: n=104 pts	**Exclusion criteria**:<br>• Revascularization with past 12 mo<br>• Exercise limiting angina<br>• SOB<br>• Severe arthritis<br>• Medications for IC except if using long term	20 min in 40 min session. 2 min bouts intermittent with 2 min rest<br><br>**Comparator**: Leg cycling using same parameters as for arm exercise and a non-exercise control group	were observed in the upper limb group, and an improvement in the general health domain of the SF-36v2 vs. controls was observed in the lower limb group (p=0.010). After 24 wk, all 4 WIQ domains were improved in the upper limb group vs. controls (p≤0.05), and 3 of the 4 WIQ domains were improved in the lower limb group (p<0.05).<br><br>**Safety endpoint**: Not specified but though no unanticipated adverse events in either group. These were too few to ascertain group effects.	in both exercise groups vs. controls. Sustained improvements were also seen in both exercise groups vs. control.
Treat-Jacobson D, et al.<br>2009 (192)<br>19651669	**Aim**: Compare effects of aerobic arm-ergometry vs. treadmill walking or usual care in PAD with IC<br><br>**Study type**: RCT<br><br>**Size**: n=41 pts	**Inclusion criteria**: Lifestyle-limiting claudication, ABI ≤0.9, drop in ABI of ≥10% after treadmill walking,<br><br>**Exclusion criteria**: Uncontrolled HBP, CLI, exercise limited by other health conditions, coronary or LE revascularization past 3 mo	**Intervention**: Arm-ergometry at one work level below maximal during baseline test. 3d/wk, exercise for 2 min, rest for 2 min for 60 min. Progressive increase of exercise over 12 wk by increasing workload and exercise bouts<br><br>**Comparator**: TM walking to 4/5 claudication, rest, exercise. Workload increased when pt could walk 8 min without having to stop due to IC. A combination group performed both arm ergometry and walking. A usual care group did not receive participate in supervised exercise but given usual care walking	**1° endpoint**: 12 wk maximal walking distance increased in the arm-ergometry (+53%), treadmill (+69%), and combination (+68%) groups (p<0.002 vs. control). The 12 wk pain free walking distance increased in the arm-ergometry group (+82%; p=0.025 vs. control). Change in PFWD in treadmill (+54%; p=0.196 vs. control) and combination (+60%; p=0.107 vs. control) groups did not reach statistical significance.<br><br>**Safety endpoint**: Not specified with 1 study unrelated injury.	• 24 wk MWD was maintained in the arm-ergometry (p=0.009) and treadmill (p=0.019) groups, whereas the combination group declined (p=0.751) vs. control. PFWD improvement was maintained in the arm-ergometry group after a 12 wk follow-up (+123%; p=0.011 vs. control)<br>• Resting SBP was lower after 12 wk on in arm group (-17 mm Hg) vs. controls. This was maintained at 24 wk (-11 mm Hg).

© American Heart Association, Inc. and American College of Cardiology Foundation

			guidelines.		
Mika P, et al.<br>2013 (193)<br>23117015	**Aim**: To compare 3 mo of SET performed to moderate claudication pain vs. pain-free walking in pts with IC<br><br>**Study type**: RCT<br><br>**Size**: n=60 pts	**Inclusion Criteria**: Age 50–75 y with IC, stable medical therapy for 6 mo, not taking medications for IC pain.<br><br>**Exclusion criteria**: CVD event in prior 1 y, unstable angina, impaired function status due to cardiac, lung, kidney, liver, or joint disease, unable to walk at 3.2 km/hr.	**Intervention**: Titled MT. SET, 3 times/wk at 3.2 km/hr and grade that induced IC within 3–5 min. Walking done with intermittent bouts of walking to moderate pain and rest until pain abated. The session was done initially for 35 min and progressed by 5 min each 2 wk until a total of 60 min was accomplished.<br><br>**Comparator**: Titled PFT. The PFT walked until initial onset of pain, stopped to rest, and then resumed walking following the same pattern as the MT group.	**1° endpoint**: Post-training MWD was prolonged by 100% (p<0.001) vs. 98% (p<0.001), and PFWT by 120% (p<0.001) vs. 93% (p<0.001) in the MT group as compared to the PFT, respectively.<br><br>Endothelial function assessed by flow-mediated dilation increased by 56% (p<0.001) in the MT group and by 36% (p<0.01) in the PFT group.<br><br>**Safety endpoint**: Not specified. Among 8 dropouts/withdrawal, none were reported as being related to SET in either group.	• No significant changes in the levels of hs-CRP and fibrinogen were seen after SET in either group. The smoking status and BMI did not change significantly after the program in both groups (p>0.05).
**CETAC**<br>Fakhry F, et al.<br>2013 (194)<br>23842830	**Aim**: Compare the long-term clinical effectiveness of a SET-first or an ER-first treatment strategy in pts with IC.<br><br>**Study type**: RCT<br><br>**Size**: n=151 pts	**Inclusion criteria**: Stable IC with iliac and femoropopliteal disease.<br><br>**Exclusion criteria**: N/A	**Intervention**: 24 wk of supervised TM exercise, 30 min, 2 d/wk, and 3 d/wk walk at home.<br><br>**Comparator**: Endovascular revascularization with initial angioplasty and stenting as needed	**1° endpoint**: After 7 y, functional performance consisting of maximal walking distance and pain free walking distance (p<0.001) and QoL (p≤0.005) had improved after both SET and ER. Long-term comparison showed no differences between the two treatments. Except in the secondary intervention rate, which was significantly higher after SET (p=0.001). Yet, the total number of endovascular and surgical interventions	• The portion of pts not needing secondary intervention rate, was significantly lower after SET, 47% vs. 73% with ER (p=0.001). Yet, the total number of endovascular and surgical interventions (primary and secondary) remained higher after ER, 121 vs. 64 (p<0.001)<br>• The cumulative survival probability for 7 y was 68% with SET and 74% with ER, (HR: 1.35; 95 % CI: 0.67–2.70; p=0.402)

© American Heart Association, Inc. and American College of Cardiology Foundation

				(primary and secondary) remained higher after ER (p<0.001)<br><br>**Safety endpoint:** See secondary outcomes	
Mazari FA, et al. 2010 (195) 19762206	**Aim:** To compare the 3 mo effects of PTA, SET, and PTA + SET for the treatment of femoropopliteal disease in pts with IC<br><br>**Study type:** RCT<br><br>**Size:** n=178 pts	**Inclusion criteria:** Stable IC and suitable for PTA for femoropopliteal lesions after 3 mo of optimal medical therapy for CVD risk factors and DM.<br><br>**Exclusion criteria:** CLI, severe systemic disease, inability to tolerate treadmill testing, significant cardiac ischemia; revascularization in prior 6 mo	**Intervention:** SET, 3 times/wk for 12 wk, consisting of circuit training that included stepping, heel raises, leg press, exercise cycle, knee extension, and elbow flexion. PTA consisting of balloon angioplasty and no stenting.<br><br>**Comparator:** Combined PTA + SET.	**1° endpoint:** All groups demonstrated significant clinical (pt reported walking distance, MWD, PFWD, rest and post-exercise ABI) and QoL improvements (p<0.05). Combined therapy produced greater improvement in clinical outcomes than PTA or SET alone (p<0.05) but not in QoL measures.<br><br>**Safety endpoint:** See secondary outcomes. No study specific adverse events reported.	• 21 pts (7%) withdrew, of whom 8 were in the SET group, 3 were in the PTA group, and 10 were in the combined group. 11 pts who had PTA had restenosis but none required revascularization.
**ERASE** Fakhry F, et al. 2015 (196) 26547465	**Aim:** To assess the 1 y effectiveness of combination therapy of ER + SET or SET alone in pts with IC<br><br>**Study type:** RCT<br><br>**Size:** n=212 pts	**Inclusion criteria:** ABI <0.9 or decrease >0.15 with exercise, 1 or more vascular stenosis at the aortoiliac or femropoliteral level or both, and impaired MWD.<br><br>**Exclusion Criteria:** Not a candidate for revascularization or prior treatment for the target lesions, limited life expectancy; limited ambulation due to causes other than IC.	**Intervention:** Combination therapy of ER + SET. For ER, a stent was used only if the initial balloon angioplasty was not successful. SET was started 2–4 wk after ER. SET consisted primarily of intermittent bouts of treadmill walking to near-maximum claudication pain. Frequency of 2–3 sessions for 30–45 min for initial 3 mo followed by at least 1 session per wk between mo 3–6 and then 1 session per 4 wk until 1 y.	**1° endpoint:** After 1 y, MWD increased in both groups (p<0.001) with a greater improvement in the combined therapy group (p<0.001)<br><br>**Safety endpoint:** See secondary outcomes. No study specific AE's discussed.	• After 1 y, PFWD increased in both groups (p<0.001) with a greater improvement in the combined therapy groups (p<0.001). Similarly, ABI at rest and after exercise showed significantly greater improvement in the combination therapy group. Also, measures of health-related QoL improved in both groups with greater improvements with combined therapy.<br><br>• A higher proportion of pts without an additional intervention in the combination group (92%) vs. the SET alone (77%), HR: 3.2; 95% CI: 1.1–9.2; p=0.005.

© American Heart Association, Inc. and American College of Cardiology Foundation

			**Comparator**: SET alone.		
Guidon M and McGee H 2013 (197) 22804715	**Aim**: To assess the 1 y effects of participation in a 12 wk supervised exercise program on functional capacity and QoL for PAD pts<br><br>**Study type**: RCT<br><br>**Size**: n=44 pts	**Inclusion criteria**: Fontaine Stage II, ABI <0.9 at rest or a decrease of ankle pressure by ≥15 mm Hg post-exercise<br><br>**Exclusion criteria**: Comorbidities which precluded participation in exercise, MI past 6 mo, acute onset or within one mo of IC, lower limb revascularization past 6 mo	**Intervention**: 2 d/wk supervised exercise for 12 wk. 30–40 min of aerobic exercise using a range of equipment including treadmill, stepper, elliptical trainer, recumbent cycle, and arm cycle. Intensity of 70%–80% of exercise test maximum HR. On treadmill, walking to leg pain of 3 of 4, rest, and resume walking. Exercise intensity progressed by increasing resistance or time.<br><br>**Comparator**: Usual care, general advice about exercise and smoking cessation, ABI measurement	**1° endpoint**: At 12 wk, there was a trend towards improved QoL in both groups, with a tendency for greater improvement in the exercise group (p=0.066) and a trend towards improved functional capacity (WIQ Stair-climbing p=0.093) in the exercise group, with an increase of 8.55 points in the exercise group and a decrease of 13.42 points in the control group. At 1 y, IC Questionnaire scores in the exercise group were considerably better than those in the control group, 27.94±19.83 vs. 38.54±24.26 (p=0.058), reflecting improved QoL and maintenance of benefits.<br><br>**Safety endpoint**: Not specified. 2 exercisers and 1 control dropped for progression of PAD, 3 exercisers dropped for non-specified medical reasons in first 12 wk.	N/A
Gardner AW, et al. 2014 (198) 25237048	**Aim**: To compare the 12 wk effects of exercise training using a step watch home-exercise program, a supervised exercise program,	**Inclusion criteria**: Sx PAD by Hx of ambulatory leg pain or pain confirmed by treadmill exercise or ABI ≤0.90 at rest or ≤0.73 after exercise.<br><br>**Exclusion criteria**: ABI	**Intervention**: Home-based 3 mo of intermittent walking (NEXT STEP) o mild-to-moderate claudication pain 3 d/wk, progressing from 20–45 min/session. Pts used step monitor during each session. Exercise logs	**1° endpoint**: At 12 wk, change scores for COT (p<0.001), PWT (p<0.001), 6 min walk distance (p=0.028), daily average cadence (p=0.011) were different among the 3 groups, with both walking programs showing changes in these	• Time to minimum calf muscle StO2 during exercise (p=0.025), large-artery elasticity index (p=0.012), and high-sensitivity C-reactive protein (p=0.041) were also significantly different among the 3 groups. Both walking groups improved time to minimum StO_2. Only the NEXT Step home group had

© American Heart Association, Inc. and American College of Cardiology Foundation

	and an attention control group on walking time and selected physiological outcomes.<br><br>**Study type**: RCT<br><br>**Size**: n=180 pts	≥1.40; asx PAD; medications for PAD symptoms, other serious comorbidities.	were reviewed by study staff, and feedback was given to guide subsequent exercise sessions.<br><br>**Comparator**: Supervised exercise while wearing step activity monitor following similar workout protocol as home-based group. There was also an attention-control, light resistance exercise group that did not walk but performed various resistance exercise. These pts also wore a step monitor to quantify time of their visits.	walking parameters from baseline. The change for PWT in the supervised exercise group was greater than the home-based group (p<0.05).<br><br>**Safety endpoint**: Not specified. 1 stroke and 1 MI in attention control group; 1 stroke in supervised exercise group; 1 leg revascularization in home-based walking group.	improvements from baseline in LAEI, and hs-CRP (p<0.05).
Langbein WE, et al. 2002 (199) 12021703	**Aim**: To determine if polestriding exercise increases exercise tolerance of persons with IC pain caused by PAD.<br><br>**Study type**: RCT<br><br>**Size**: n=52 pts	**Inclusion criteria**: Pain from claudication primary limiting factor to maximal exercise<br><br>**Exclusion criteria**: Severe leg pain at rest, ischemic ulceration, resting ABI <0.4, revascularization in past y, current use of vitamin E, warafin sodium, or pentoxifylline, other factors limiting exercise	**Intervention**: Polestriding exercise 3 times/wk for 4 wk, twice per wk for 8 wk, once per wk for 4 wk.<br><br>**Comparator**: Nonexercise control	**1° endpoint**: Polestriding improved exercise tolerance on the constant work-rate and incremental treadmill tests (p<0.001). Perceived claudication pain were significantly less after polestriding training program. pt perceived distance (p<0.001) and walking speed scores (p<0.022) on the Walking Impairment Questionair improved in the polestriding trained group only.<br><br>**Safety endpoint**: N/A	**2° endpoint:** No changes in resting or postexercise ABI
Walker RD, et al. 2000 (200) 10753273	**Aim**: To compare effects of upper limb (arm cranking) and lower-limb (leg cranking) exercise training on walking	**Inclusion criteria**: Moderate to severe IC<br><br>**Exclusion criteria**: Claudication of >12 mo or revascularization in	**Intervention**: An upper-limb and lower limb training groups 2 d/wk for 6 wk. Each group performed intermittent 2 min bouts of exercise followed by 2 min	**1° endpoint**: Both training groups improved the maximum power generated during the incremental upper- and lower-limb ergometry tests (p<0.001). PFWD and MWD improved in	• Improvements in physical function and role-limitation-physical domains of the SF-36 QoL questionnaire.<br>• No exercise-related adverse events.

© American Heart Association, Inc. and American College of Cardiology Foundation

	distances in pts with claudication.<br><br>**Study type**: RCT<br><br>**Size**: n=76 pts	previous 12 mo; other exercise-limiting comorbidities such as angina, shortness of breath, severe arthritis.	of rest; total exercise of 20 min during a 40 min session<br><br>**Comparator**: Untrained group	both groups (p<0.001). Improvements were similar between the 2 training groups, while there was no change in the untrained control group.<br><br>**Safety endpoint**: N/A	

ABI indicates ankle-brachial index; ACC, Journal of American College of Cardiology; BMI, body mass index; CABG, coronary artery bypass graft; CI, confidence interval; CLI, critical limb ischemia; COT, claudication onset time; CV, cardiovascular; CVD, cardiovascular disease; ER, endovascular revascularization; HR: hazard ratio; HBP, high blood pressure; HR, hazard ratio; hs-CRP, high-sensitivity C-reactive Protein; IC, intermittent claudication; JAMA, Journal of American Medical Association; LAEI, large artery elasticity; LDL, low density lipoproteins; LE, lower extremity; METs, metabolic equivalent; MI, myocardial infarction; MWD, maximal walking distance; N/A, not applicable; OMC, optimal medical care; OR, odds ratio; PAD, periphery artery disease; PAQ, personal attributes questionnaire; PFT, pain free time; PFWD, pain free walking distance; PFWT, pain free walking time; PWT, peak walking time; QoL, quality of life; RCT, randomized controlled trial; RR, relative risk; RPE, ratings of perceived exertions; SBP, systolic blood pressure; SE, supervised exercise; SET, supervised exercise training; SOB, shortness of breath; StO_2, tissue oxygen saturation; ST, stent revascularization; TM, treadmill; and WIQ, walking impairment questionnaire.

Evidence Table 33. Nonrandomized Trials, Observational Studies, and/or Registries for Exercise Therapy–Section 6.

Study Acronym; Author; Year Published	Study Type/Design; Study Size	Patient Population	Primary Endpoint and Results (include P value; OR or RR; & 95% CI)	Summary/Conclusion Comment(s)
Pilz M, et al.<br>2014(201)<br>24825596	**Study type**: Nonrandomized intervention consisting of combined aerobic and strength training lasting for 6 or 12 mo in pts with IC.<br><br>**Size**: n=94 pts (n=42 for 6 mo, n=52 for 12 mo)	**Inclusion criteria**: PAD Rutherford stage 1–3, ABI ≤0.9,<br><br>**Exclusion criteria**: Rutherford stage 0 or 4–6, exercise limiting CVD or orthopedic conditions, only aorto-illiac stenosis	**1° endpoint**: Maximal walking distance, walking speed, muscle strength<br><br>**Results**: Significant increases in all parameters evaluated, but greater benefit was found in the 12 mo training group. The absolute claudication distance increased similarly by 27.5% and 29.5%, respectively, at 6 and 12 mo a greater increase in walking speed (12.1% vs. 5.3%;, p<0.001) was seen at 12 vs. 6 mo. All strength parameters increased significantly in both the groups showing an increase for "pushing" by 90.0% (6 mo) and 90.2% (12 mo), for "pulling" by 64.2% (6 mo) and 75.3% (12 mo), and for "tiptoe standing" by 70.5% (group A) and 113.7% (12 mo; p<0.05).	• Combined exercise increased walking speed, MWD, and muscle strength parameters.<br>• Greater improvements resulted from the 12 mo program<br>• No changes in weight, total cholesterol, or blood sugar in the 6 mo group. Total cholesterol decreased by -9.4 mg/dL in 12 mo group (p=0.0053)<br>• Strength exercise involved lower extremity exercise<br>• Though the program was supervised, walking was done on a track in a gym rather than treadmill to mimic walking in a community setting. Pts were also instructed to walk on the weekends on their own.
Mays RJ, et al.<br>2013(202)<br>24103409	**Study type**: Literature review<br><br>**Size**: n=10 RCTs	**Inclusion criteria**:<br>• PubMed/MEDLINE and Cochrane databases<br>• English language	**1° endpoint**: Peak walking performance on the treadmill.<br><br>**Results**: Supervised exercise programs were	• Unstructured recommendations for pts with sx PAD to exercise in the community are not efficacious.<br>• Community walking programs may improve with

© American Heart Association, Inc. and American College of Cardiology Foundation

		• Used community walking programs to treat PAD pts with IC<br><br>**Exclusion criteria**: N/A	more effective than community walking studies with general recommendations to walk at home. Community trials that incorporated more advice and feedback for PAD pts in general resulted in similar outcomes with no differences in peak walking time compared to supervised walking exercise groups.	more feedback and monitoring

CVD indicates cardiovascular disease; IC, intermittent claudication; MWD, maximum walking distance; PAD, peripheral artery disease; and pt, patient.

Evidence Table 34. Nonrandomized Trials and Observational Studies of Minimizing Tissue Loss in Patients with PAD–Section 7.

Study Acronym; Author; Year Published	Study Type/Design; Study Size	Patient Population	Primary Endpoint and Results (include P value; OR or RR; & 95% CI)	Summary/Conclusion Comment(s)
Crane M and Werber B 1999(203)<br>10028467	**Study type**: NR, retrospective cohort<br><br>**Size**: n=115 pts (55 nonpathway, 60 pathway)	**Inclusion criteria**: All diabetic foot infections 1993 and 1995–1996	**1° endpoint**: Prevalence of major (leg) amputation among those admitted with infection<br><br>**Results:** 23% nonpathway vs. 7% pathway	Established pathway allows "earlier recognition, evaluation and expedient treatment of potentially limb-threatening infections"
Larsson J, et al. 1995(204)<br>8542736	**Study type**: NR, retrospective cohort<br><br>**Size**: n=200,000 pt population with 2.4% prevalence of DM (~4,800)	**Inclusion criteria**: "All DM related primary amputations from toe to hip" between 1982 and 1993	**1° endpoint**: Annual incidence (per inhabitant) of major and minor amputation<br><br>**Results:** All amputations=19.1 vs. 9.4 per 100K; major amputations=16 vs. 3.6 per 100K	"Multidisciplinary approach plays an important role to reduce and maintain a low incidence of major amputations in diabetic pts"
Armstrong DG, et al. 2012(205)<br>22431496	**Study type**: NR, retrospective cohort<br><br>**Size**: n=790 diabetic foot operations	**Inclusion criteria**: All diabetic foot operations 2006–2008 vs. 2008-2010	**1° endpoint**: Amputation level, case mix<br><br>**Results**: 37.5% reduction in transtibial amputations; 44% increase in vascular interventions	Interdisciplinary care as a "rapid and sustained impact in changing surgery type from reactive to proactive" and reduces major amputations
Chung J, et al. 2015(206)<br>25073577	**Study type**: NR, retrospective cohort<br><br>**Size**: 85 pts	**Inclusion criteria**: "All consecutive pts" with R5/6 CLI at a single hospital 8/2010–6/2012	**1° endpoint**: 1 y amputation-free survival<br><br>**Results**: 67 vs. 42% at 1 y; also higher mean limb salvage times. Multidisciplinary care remained significant on multivariate analysis	Multidisciplinary care improves amputation-free survival in pts with R5/6 CLI
Canavan RJ, et al. 2008(207)	**Study type**: NR<br><br>**Size**: n=273,987 population	**Inclusion criteria**: All LE amputations from 7/1995–6/2000	**1° endpoint**: Incidence of major and minor amputations	Reduction in major amputations "a result of better organized diabetes care"

© American Heart Association, Inc. and American College of Cardiology Foundation

18071005	with 1.94% prevalence of DM		**Results:** Decrease in incidence from 564–176/100K pts with DM between first and fifth y after change; increase in angioplasty prevalence	
Williams DT, et al. 2012(208) 22503433	**Study type:** NR, retrospective & prospective cohorts<br><br>**Size:** n=220,000 pts with 4.2% prevalence of DM (9,328)	**Inclusion criteria:** All DM or PAD pts receiving in pt treatment 1/2004–12/2005 (before service) vs. 1/2006–12/2009 (after service)	**1° endpoint:** Incidence of major and minor amputation<br><br>**Results:** Fewer major amputations among DM pts (peak of 24.7 to nadir of 1.07 per 10,000); decrease in minor amputations	"Formation of a well-defined [multidisciplinary] service … has been associated with further demonstrable reductions in limb loss caused by diabetic foot disease."
Driver VR, et al. 2005(209) 15677774	**Study type:** NR<br><br>**Size:** n=About 350,000 population (4,940 with DM)	**Inclusion criteria:** All in pt LEA between 1999–2003	**1° endpoint:** Incidence of LEA (all levels)<br><br>**Results:** Decreased amputation incidence from 9.9–1.6 per 1K (71% of which were minor)	Multidisciplinary care improves outcomes, decreases amputation rates
Wrobel JS, et al. 2003 (210) 14578237	**Study type:** Cross-sectional<br><br>**Size:** n=10 Veterans Affairs medical centers	**Inclusion criteria:** Surveys of general, vascular, and orthopedic surgeons; rehabilitation specialists; podiatrists; physical therapists; pedorthists; orthotists; DM care specialists; DM educators; dermatologists; wound care specialists; and infectious disease clinicians; and 10 randomly-selected primary care providers	**1° endpoint:** Correlation between lower extremity amputation rates and<br><br>**Results:** Significant negative correlation between programming coordination and total and minor amputations	Improved programming coordination more influential than feedback coordination or site rankings on decreasing amputation rates
Vartanian et al. 2015 (211) 25596408	**Study type:** NR, retrospective review<br><br>**Size:** n=91 limbs from 89 pts	**Inclusion criteria:** Pts with neuroischemic wounds treated at a signle institutional amputation prevention clinic from March 2012–July 2013. Pts at highest risk for limb loss, defined as ischemic wounds (ischemic ulcer or gangrene) or diabetic foot ulcers.<br><br>**Exclusion criteria:** New pts evaluated for benign conditions (e.g., arthritis, overuse injuries, simple infections in nondiabetics, venous ulcers, minor trauma, radiculopathy).	**1° endpoint:** Time to wound healing, reulceration rate, and ambulatory status.<br><br>**Results:** 67% of wounds were present >6 wks before referral. A total of 151 podiatric and 86 vascular interventions were prformed, with an equal distribution of endovascular and open revascularizations. Complete wound healing observed in 59% of wounds, and average time to full healing was 12 wk. Hindfood wounds predictive of failure to heal (OR: 0.21; p <0.01; 95% CI: 0.06–0.68).	Multidisciplinary care can help effectively heal wounds and maintain ambulatory status in pts with limb threatening neuroischemic wounds. Hindfoot or ankle wounds can adversely influence the outcome. Healing can be prolonged and a substancial proportion of pts can be expected to have a recurrence, therefore surveillance is mandatory. A coordinated amputation prevention program may help to minimize hospital readmissions in the high-risk population.

© American Heart Association, Inc. and American College of Cardiology Foundation

Gardner SE, et al. 2009(212) 19147524	**Study type:** Cross sectional study<br><br>**Size:** n=64 pts	**Inclusion criteria:**<br>• Age ≥18 y of age<br>• Pts with ≥1 full-thickness, nonarterial diabetic foot ulcers from a Department of Veterans Affairs Medical Center and an academic-affiliated hospital<br><br>**Exclusion criteria:**<br>• White blood cell count <1500 cells/mm^3<br>• Patelet count <125,000/mm^3<br>• Coagulopathies<br>• Receiving anticoagulation therapy	**1° endpoint:**<br>• Sensitivity, specificity, and concordance probability of each sign as compared to microbial load (reference standard),<br>• Sensitivity, specificity, and concordance probability of the IDSA combination of signs as compared to microbial load, and<br>• discriminatory accuracy of a composite predictor computed from the classic and signs specific to secondary wounds as compared to microbial load.<br><br>**Results:**<br>• No signs were significant predictors, although increasing pain was marginally insignificant (c=0.56; p=0.055)<br>IDSA combination of signs were not significant.<br>Composite predictor c=0.783; $c_{overfitting\ corrected}$=0.645; SE=0.0483; 95% CI: 0.559–0.732.	Individual signs of infection do not perform well nor does the IDSA combination of signs<br>A composite predictor based on all signs provides a moderate level of discrimination
Lipsky BA, et al. 2012(213) 22619242	**Study type:** Summary of new guidelines for diabetic foot infections<br><br>**Size:** N/A	**Inclusion criteria:** N/A<br><br>**Exclusion criteria:** N/A	**1° endpoint:** N/A<br><br>**Results:** N/A	N/A
Pickwell K, et al. 2015(214)	**Study type:** Prospective study<br><br>**Size:** n=575 pts	**Inclusion criteria:** Part of the Eurodiale study.<br><br>**Exclusion criteria:** Pts treated in the participating centers for an ulcer of the ipsilateral foot during the previous 12 mo and those with life expectancy <1 y	**1° endpoint:** Healing of the foot ulcer, major amputation, or death<br><br>**Results:** 159 (28%) pts (126 minor and 33 major) within 1 y follow-up; 103 pts (18%) underwent amputations proximal to and including the hallux<br>Incidence of amputation increased with redness, periwound or pretibial edema, the presence of pus, lymphadenitis/lymphangitis, fever (all p<0.01) and elevated CRP (p=0.01).	Positive probe-to-bone test, deep ulcer, elevated CRP levels, and the presence of periwound or pretibial edema. The presence of increased (non)purulent exudate, foul smell, and fever independently predicted any amputation but not amputations excluding the lesser toes are risk factos for lower extremity amputation in pts with diabetic foot ulcers.
Dinh MT, et al. 2008(215)	**Study type:** Meta-analysis<br><br>**Size:** n=9 articles from the	**Inclusion criteria:** studies that assess the accuracy of clinical or imaging diagnostic modalities for	**1° endpoint:**<br><br>**Results:**	Among the imaging tests that we evaluated, MRI was the most accurate. However, MRI is costly and may not be

© American Heart Association, Inc. and American College of Cardiology Foundation

	literature search and 59 studies identified by perusing reference lists of potentially relevant articles	diagnosis of osteomyelitis in pts with diabetes and foot ulcer, and studies that used histopathologic examination and/or microbiologic culture of bone specimens as the reference test for diagnosis of osteomyelitis. All pts had to participate in the test being studied as well as the reference test<br><br>**Exclusion criteria: N/A**	• A positive probe-to-bone test result in had a sensitivity of 0.87 (95% CI: 0.71–0.96) for diagnosis of osteomyelitis and a specificity of 0.91 (95% CI: 0.89–0.92). The likelihood ratio for a positive test result was 9.40, and the likelihood ratio for a negative test result was 0.14,<br>• The pooled diagnostic OR for exposed bone or a positive probe-to-bone test result was 49.45<br>• Sensitivity of plain radiography for diagnosis of osteomyelitis was highly variable, ranging from 0.28–0.75	readily available. Nuclear medicine bone scan and indium-labeled leukocyte scans had low-to-moderate accuracy for detection of osteomyelitis. Plain radiographs provided limited information
Prompers L, et al. 2008(216) 18297261	**Study type:** Prospective cohort study within the EURODIALE Study<br><br>**Size:** n=1,088 pts	**Inclusion criteria:** Part of the EURODIALE Study<br><br>**Exclusion criteria: N/A**	**1° endpoint:** Wound healing<br><br>**Results:** At 1-y follow up, 23% of pts had not healed. Predictors of nonhealing are older age, male sex, HF, inability to stand or walk without help, ESRD, larger ulcer size, peripheral neuropathy, and PAD. Infection is a predictor of nonhealing in PAD pts only.	Predictors of healing differ between pts with and without PAD, suggesting that diabetic foot ulcers with or without concomitant PAD should be defined as two separate disease states

AFS indicates amputation-free survival; CLI, critical or chronic limb ischemia; DM, diabetes mellitus; DR, diabetes-related; ESRD, end stage renal disease; HF, heart failure; IDSA, Infectious Disease Society of America; LEA, lower extremity amputation; LPS, Limb Prevention Service; MDC, multidisciplinary care; NR, nonrandomized; OR, odds ratio; pt, patient; and RR, relative risk.

© American Heart Association, Inc. and American College of Cardiology Foundation

Data Supplement 34a. Functions of a Multidisciplinary Foot Care / Amputation Prevention Team–Section 7.

Study Name	Patient Education	Risk Stratification, Testing for Neuropathy and/or PAD	Prophylactic Podiatric Surgery	Protocols, Algorithms, Referral Pathways	Wound Care, Including Debridement in Clinic	Infection Management	Close Post-Operative Monitoring	Orthotics and Prosthetics	Other
Crane 1999 10028467 (203)				X					
Driver 2005 15677774 (209)	X	X			X	X	X	X	Research; community outreach/education
Williams 2012 22503433 (208)	X			X	X				Admission to vascular inpatient service for infection; multidisciplinary clinics
Rogers 2010 20804929 (217)		X	X		X	X	X	X	Gait analysis; medical management of PAD
Sumpio 2010 20488327 (218)		X	X		X	X	X	X	
Fitzgerald 2009 19436764 (219)		X			X	X	X		
Wrobel 2006 16649651 (220)	X			X					Ease in recruiting staff; confidence in staff; clinician attendance at diabetic foot care education program in past 3 yrs

PAD indicates peripheral artery disease.

© American Heart Association, Inc. and American College of Cardiology Foundation

Evidence Table 35. RCTs Comparing Endovascular Treatment and Endovascular Versus Noninvasive Treatment of Claudication–Section 8.1.

Study Acronym; Author; Year Published	Aim of Study; Study Type; Study Size (N)	Patient Population	Study Intervention (# patients) / Study Comparator (# patients)	Endpoint Results (Absolute Event Rates, P value; OR or RR; & 95% CI)	Relevant 2° Endpoint (if any); Study Limitations; Adverse Events
Tetteroo E, et al. 1998(221) 9643685	**Aim**: Determine superiority of iliac PTAS vs. PTA<br><br>**Study type**: RCT<br><br>**Size**: n=279 pts	**Inclusion criteria**:<br>• Claudication<br>• Iliac artery stenosis <5cm<br><br>**Exclusion criteria**:<br>• Stenosis >10 cm in length<br>• Arterial occlusion >5 cm in length, or ≤5 cm not allowing the passage of a guide wire<br>• Stenosis involving the distal aorta; severe comorbidity (e.g., severe cardiac or cerebrovascular abnormality, malignant disease)	**Intervention**: PTAS<br><br>**Comparator**: PTA	**1° endpoint**: Reduction in symptoms; QoL	• No difference between groups at 2 y<br>• Group I=PTAS. Group II=PTA. The mean follow-up was 9.3 mo (range 3–24). Initial hemodynamic success and complication rates were 119 (81%) of 149 limbs and 6 (4%) of 143 limbs (group I) vs. 103 (82%) of 126 limbs and 10 (7%) of 136 limbs (group II), respectively. Clinical success rates at 2 y were 29 (78%) of 37 pts and 26 (77%) of 34 pts in groups I and II, respectively (p=0.6); however, 43% and 35% of the pts, respectively, still had symptoms. QoL improved significantly after intervention (p<0.05) but no difference between the groups during follow-up. 2 y cumulative patency rates were similar at 71% vs. 70% (p=0.2), respectively, as were reintervention rates at 7% vs. 4%, respectively (95% CI: 2%–9%).
Klein WM, et al. 2004(222) 15286319	**Aim**: Determine superiority of iliac PTAS vs. PTA<br><br>**Study type**:<br><br>**Size**: n=279 pts	**Inclusion criteria**:<br>• Claudication<br>• Iliac artery stenosis <5cm<br><br>**Exclusion criteria**:<br>• Stenosis of >10 cm in length<br>• Occlusion of >5 cm in length, or of ≤5 cm if it did not allow the passage of a guidewire; stenosis involving the distal aorta<br>• Or severe comorbidity (e.g., severe cardiac or cerebrovascular abnormality, malignant disease)	**Intervention**: PTAS<br><br>**Comparator**: PTA	**1° endpoint**: Technical success and incidence of reintervention	• No difference between groups<br>• Long-term follow-up from above study. The mean follow-up period was 5.6 y±1.3 (±standard deviation). There were no significant differences between primary stent placement and primary angioplasty treatment groups in regard to number of reinterventions in the treated iliac arteries (33 [18%] of 187 segments and 33 [20%] of 169 segments, respectively) or in the ipsilateral legs (45 [25%] of 181 legs and 50 [30%] of 164 legs, respectively). Sex, presence of critical ischemia, and length of stenosis were predictors of whether a pt would require iliac reintervention.

© American Heart Association, Inc. and American College of Cardiology Foundation

Bosch JL and Hunink MG 1997(223) 9205227	**Aim**: Determine superiority of iliac PTAS vs. PTA<br><br>**Study type**: Meta-analysis<br><br>**Size**: n=301 pts	**Inclusion criteria**:<br>• Claudication of CLI<br>• Iliac artery involvement<br><br>**Exclusion criteria**: Studies without specified endpoints	**Intervention**: PTAS<br><br>**Comparator**: PTA	**1° endpoint**: Technical success; primary patency<br><br>**Safety endpoint**: Mortality and MACE	• No difference between groups<br>• The immediate technical success rate in the PTA group was 91%; the rate was higher in the stent group (96%), but the difference was not statistically significant [corrected]. Complication and mortality rates were not statistically significantly different. Analyzed data included technical failures and were adjusted for lesion type and disease severity. 4 y primary patency rates were 65% for stenoses vs. 54% for occlusions after PTA to treat claudication and were 53% for stenoses vs. 44% for occlusions after PTA to treat critical ischemia. These rates were 77% for stenoses vs. 61% for occlusions after stent placement to treat claudication and 67% for stenoses vs. 53% for occlusions after stent placement to treat critical ischemia. The risk of long-term failure was reduced by 39% after stent placement compared with PTA.
Kashyap VS, et al. 2008(224) 18804943	**Aim**: Iliac occlusive disease. PTAS vs. aorto-bifem<br><br>**Study type**: Retrospective<br><br>**Size**: PTAS n=83 pts vs. ABF n=86 pts	**Inclusion criteria**: Sx aorto-iliac occlusive disease (claudication 53% rest pain, 28%; tissue loss, 12%; ALI, 7%)<br><br>**Exclusion criteria**:<br>• Pts undergoing endovascular treatment such as PTA or stenting for iliac stenoses.<br>• Pts with iliac dissection, an associated AAA, or iliac recanalization before or during AAA endograft placement.	**Intervention**: PTAS<br><br>**Comparator**: ABF	**1° endpoint**: Technical success; primary patency; secondary patency; survival	• Primary patency at 3 y was significantly higher for ABF than for R/PTAS (93% vs. 74%, p=0.002)<br>• Secondary patency rates (97% vs. 95%), limb salvage (98% vs. 98%), and long-term survival (80% vs. 80%) were similar
**ABSOLUTE** Schillinger M, et al. 2007(225) 17502568	**Aim**: SFA PTAS vs. PTA<br><br>**Study type**: RCT<br><br>**Size**: n=104 pts	**Inclusion criteria**: Rutherford 3–5 and SFA stenosis<br><br>**Exclusion criteria**:<br>• Acute CLI, previous bypass surgery, or stenting of the	**Intervention**: PTAS<br><br>**Comparator**: PTA	**1° endpoint**: Restenosis by duplex at 2 y	• PTAS is superior to PTA for long lesions (lesion length 112 mm PTAS and 93 mm PTA)<br>• Of 104 pts with chronic limb ischemia and superficial femoral artery obstructions, 98 (94%) could be followed up until 2 y after intervention for occurrence of restenosis (>50%) by duplex

© American Heart Association, Inc. and American College of Cardiology Foundation

		SFA<br>• Untreated inflow disease of the ipsilateral pelvic arteries (>50% stenosis or occlusions)			ultrasound and for clinical and hemodynamic outcome by treadmill walking distance and ABI. Restenosis rates at 2 y were 45.7% (21 of 46) vs. 69.2% (36 of 52) in favor of primary stenting compared with balloon angioplasty with optional secondary stenting by an ITT analysis (p=0.031). Consistently, stenting (whether primary or secondary; n=63) was superior to plain balloon angioplasty (n=35) with respect to the occurrence of restenosis (49.2% vs. 74.3%; p=0.028) by a treatment-received analysis. Clinically, pts in the primary stent group showed a trend toward better treadmill walking capacity (average, 302 vs. 196 m; p=0.12) and better ABI values (average, 0.88 vs. 0.78; p=0.09) at 2 y, respectively. Reintervention rates tended to be lower after primary stenting (17 of 46 [37.0%] vs. 28 of 52 [53.8%]; p=0.14)
**FAST**<br>Krankenberg H, et al.<br>2007 (226)<br>17592075	**Aim**: SFA PTA vs. PTAS<br><br>**Study type**: RCT<br><br>**Size**: n= 244 pts	**Inclusion criteria**: SFA stenosis and claudication or CLI<br><br>**Exclusion criteria**:<br>• TL that required pretreatment with adjunctive devices, e.g., lasers or debulking catheters<br>• A TL that extended into the popliteal artery; previous stent implantation in the targeted SFA<br>• Multiple lesions >10 cm in length<br>• Acute or subacute (≤4 wk) thrombotic occlusion<br>• An untreated ipsilateral iliac artery stenosis<br>• Ongoing dialysis treatment<br>• Treatment with oral anticoagulants other than antiplatelet agents.	**Intervention**: PTAS<br><br>**Comparator**: PTA	**1° endpoint**: Technical success, 1 y duplex restenosis	• For short lesions mean length 45 mm, no difference between PTAS and PTA<br>• Overall, stent fractures were detected in 45 of 121 treated legs (37.2%). In a stent-based analysis, 64 of 261 stents (24.5%) showed fractures, which were classified as minor (single strut fracture) in 31 cases (48.4%), moderate (fracture of >1 strut) in 17 cases (26.6%), and severe (complete separation of stent segments) in 16 cases (25.0%). Fracture rates were 13.2% for stented length ≤8 cm, 42.4% for stented length >8–16 cm, and 52.0% for stented length >16 cm. In 21 cases (32.8%) there was a restenosis of >50% diameter reduction at the site of stent fracture. In 22 cases (34.4%) with stent fracture there was a total stent reocclusion. According to Kaplan Meier estimates, the primary patency rate at 12 mo was significantly lower for pts with stent fractures (41.1% vs. 84.3%, p<0.0001).

© American Heart Association, Inc. and American College of Cardiology Foundation

Laird JR, et al. 2010(227) 20484101	**Aim**: SFA SES vs. PTA<br><br>**Study type**: RCT<br><br>**Size**: n= 206 pts	**Inclusion criteria**: Fem/pop artery stenosis<br><br>**Exclusion criteria**:<br>• Pts with CLI (Rutherford categories 4–6)<br>• Sensitivity to contrast media that was not amenable to pretreatment with steroids, antihistamines, or both<br>• Known allergies to study medications or materials<br>• Renal failure (serum creatinine >2.0 mg/dL) or hepatic insufficiency<br>• Previous bypass surgery of the target limb<br>• Extensive PVD that precluded safe insertion of an introducer sheath<br>• Aneurysmal disease in the vessel segment to be treated<br>• Thrombus in the area to be treated that could not be resolved<br>• Angiographic evidence of poor inflow that was inadequate to support vascular bypass or who were receiving dialysis or immunosuppressive therapy were ineligible	**Intervention**: PTAS<br><br>**Comparator**: PTA	**1° endpoint**: 1 y duplex derived patency	• Mean lesion length 71 mm; PTAS superior<br>• A total of 206 pts from 24 centers in the United States and Europe with obstructive lesions of the superficial femoral artery and proximal popliteal artery and IC were randomized to implantation of nitinol stents or percutaneous transluminal angioplasty. The mean total lesion length was 71 mm for the stent group and 64 mm for the angioplasty group. Acute lesion success (<30% residual stenosis) was superior for the stent group compared with the angioplasty group (95.8% vs. 83.9%; p<0.01). 29 (40.3%) pts in the angioplasty group underwent bailout stenting because of a suboptimal angiographic result or flow-limiting dissection. Bailout stenting was treated as a TL revascularization and loss of primary patency in the final analysis. At 12 mo, freedom from TL revascularization was 87.3% for the stent group compared with 45.1% for the angioplasty group (p<0.0001). Duplex ultrasound-derived primary patency at 12 mo was better for the stent group (81.3% vs. 36.7%; p<0.0001). Through 12 mo, fractures occurred in 3.1% of stents implanted. No stent fractures resulted in loss of patency or TL revascularization.
Dick P, et al. 2009(228) 19859954	**Aim**: SFA SES vs. PTA<br><br>**Study type**: RCT<br><br>**Size**: n=73 pts	**Inclusion criteria**: SFA stenosis and claudication<br><br>**Exclusion criteria**:<br>• Acute CLI<br>• Previous bypass surgery or stenting of the SFA<br>• Untreated inflow disease of	**Intervention**: PTAS<br><br>**Comparator**: PTA	**1° endpoint**: Primary patency	• PTAS is superior to PTA<br>• Average length of the treated segments was 98±54 mm and 71±43 mm in the stent and PTA groups (p=0.011), respectively. In the PTA group, secondary stenting was performed in 10 of 39 pts (26%) due to a suboptimal result after balloon dilation. Restenosis rates in the stent and PTA groups were 21.9% vs. 55.6% (p=0.005) at 6 mo by

© American Heart Association, Inc. and American College of Cardiology Foundation

		the ipsilateral pelvic arteries (>50% stenosis or occlusion)<br>• Known intolerance of study medications or contrast agent.			CTA, and 2.9% vs. 18.9% (p=0.033), 18.2% vs. 50.0% (p=0.006), and 34.4% vs. 61.1% (p=0.028) at 3, 6, and 12 mo by sonography, respectively. Clinically, pts in the stent group reported a significantly higher maximum walking capacity compared with the PTA group at 6 and 12 mo.
**IN.PACT**<br>Tepe G, et al. 2015(229)<br>25472980	**Aim**: SFA DCB vs. PTA<br><br>**Study type**: RCT<br><br>**Size**: n= 331 pts	**Inclusion criteria**: IC or ischemic rest pain attributable to superficial femoral and popliteal PAD<br><br>**Exclusion criteria**:<br>• Lesion and/or occlusions located in or extending to the popliteal artery or below the ankle joint space<br>• Inflow lesion or occlusion in the ipsilateral iliac, SFA, or popliteal arteries with length ≥15 cm<br>• Significant (≥50% DS) inflow lesion or occlusion in the ipsilateral iliac, SFA, or popliteal arteries left untreated<br>• Previously implanted stent in the TL(s). Aneurysm in the target vessel<br>• Acute thrombus in the TL	**Intervention**: DCB<br><br>**Comparator**: PTA	**1° endpoint**: 12 mo primary patency	• DCB superior to PTA<br>• The IN.PACT SFA Trial is a prospective, multicenter, single-blinded, randomized trial in which 331 pts with IC or ischemic rest pain attributable to superficial femoral and popliteal PAD were randomly assigned in a 2:1 ratio to treatment with DCB or PTA. The primary efficacy endpoint was primary patency, defined as freedom from restenosis or clinically driven TL revascularization at 12 mo. Baseline characteristics were similar between the 2 groups. Mean lesion length and the percentage of total occlusions for the DCB and PTA arms were 8.94±4.89 and 8.81±5.12 cm (p=0.82) and 25.8% and 19.5% (p=0.22), respectively. DCB resulted in higher primary patency vs. PTA (82.2% vs. 52.4%; p<0.001). The rate of clinically driven TL revascularization was 2.4% in the DCB arm in comparison with 20.6% in the PTA arm (p<0.001). There was a low rate of vessel thrombosis in both arms (1.4% after DCB and 3.7% after PTA [p=0.10]). There were no device- or procedure-related deaths and no major amputations
**DEBATE-SFA**<br>Liistro F, et al. 2013(230)<br>24239203	**Aim**: PEB+BMS vs. PTA+BMS<br><br>**Study type**: RCT<br><br>**Size**: n=104 pts	**Inclusion criteria**: Claudication and SFA stenosis<br><br>**Exclusion criteria**:<br>• Life expectancy <1 y<br>• Contraindication for combined antiplatelet therapy<br>• Known allergy to nickel or paclitaxel<br>• Need for major amputation	**Intervention**: PEB+BMS<br><br>**Comparator**: PTA+BMS	**1° endpoint**: 12 mo binary restenosis	• PEB+BMS is superior to PTA+BMS<br>• Mean lesion length was 94±60 vs. 96±69 mm in the PEB+BMS and PTA+BMS groups (p=0.8), respectively. The primary endpoint occurred in 9 (17%) vs. 26 (47.3%) of lesions in the PEB+BMS and PTA+BMS groups (p=0.008), respectively. A near-significant (p=0.07) 1 y freedom from TL revascularization advantage was observed in the PEB+BMS group. No major amputation occurred. No significant difference was observed according to lesion characteristics or technical approach.

© American Heart Association, Inc. and American College of Cardiology Foundation

		at the time of enrollment<br>• Failure to recanalize intended below-the-knee arteries in CLI pts at risk of major amputation			
Scheinert D, et al. 2014(231)<br>24456716	**Aim**: SFA DCB vs. PTA<br><br>**Study type**: RCT<br><br>**Size**: n=101 pts	**Inclusion criteria**: Rutherford class 2–5 femoropopliteal lesions<br><br>**Exclusion criteria**:<br>• Life expectancy ≤2 y<br>• Creatinine >2.5 mg/dL or Hx of hemorrhagic stroke ≤3 mo<br>• Previous surgery of the TL<br>• Previous or planned intervention ≤30 d<br>• Use of adjunctive therapies (including glycoprotein IIb/IIIa inhibitors)<br>• Severe lesion calcification<br>• Sudden symptom onset<br>• Acute or subacute target vessel thrombus or occlusion<br>• Absence of ≥1 patent untreated runoff vessel<br>• Significant inflow disease	**Intervention**: DCB<br><br>**Comparator**: PTA	**1° endpoint**: The primary endpoint was angiographic late lumen loss at 6 mo. Secondary outcomes included adjudicated major adverse events (death, amputation, TL thrombosis, reintervention), functional outcomes, and pharmacokinetics.	• DCB superior to PTA<br>• Demographic, PVD, and lesion characteristics were matched, with mean lesion length of 8.1 3.8 cm and 42% total occlusions. At 6 mo, late lumen loss was 58% lower for the Lutonix DCB group (0.46 1.13 mm) than for the control group (1.09 1.07 mm; p=0.016). Composite 24 mo major adverse events were 39% for the DCB group, including 15 TL revascularizations, 1 amputation, and 4 deaths vs. 46% for uncoated balloon group, with 20 TL revascularizations, 1 thrombosis, and 5 deaths. Pharmacokinetics showed biexponential decay with peak concentration (Cmax) of 59 ng/mL and total observed exposure (AUC(all)) of 73 ng h/ml. For successful DCB deployment excluding 8 malfunctions, 6 mo late lumen loss was 0.39 mm and the 24 mo TL revascularization rate was 24%.
Werk M, et al. 2012(232)<br>23192918	**Aim**: SFA DCB vs. PTA<br><br>**Study type**: RCT<br><br>**Size**: n=85 pts	**Inclusion criteria**: Sx femoro-popliteal atherosclerotic disease<br><br>**Exclusion criteria**: Key exclusion criteria were:<br>• Acute thrombus or aneurysm in the target vessel<br>• Failure to cross the TL with a guidewire<br>• Inflow lesions that cannot be successfully pretreated<br>• Significant disease of all 3	**Intervention**: DCB<br><br>**Comparator**: PTA	**1° endpoint**: The primary endpoint was late lumen loss at 6 mo assessed by blinded angiographic corelab quantitative analyses	• DEB is superior to PTA<br>• Pts with sx femoro-popliteal atherosclerotic disease undergoing percutaneous transluminal angioplasty were randomized to paclitaxel-coated IN.PACT Pacific or uncoated Pacific balloons. The primary endpoint was late lumen loss at 6 mo assessed by blinded angiographic corelab quantitative analyses. Secondary endpoints were binary restenosis and Rutherford class change at 6 mo, and TL revascularization + major adverse clinical events (major adverse events=death, target limb amputation, or TL revascularization) at 6 and 12 mo. 85 pts (91 cases=interventional procedures) were randomized in 3 hospitals (44 to DEB and 47

© American Heart Association, Inc. and American College of Cardiology Foundation

		infrapopliteal vessels<br>• Renal failure (serum creatinine >2.0 mg/dL)<br>• Known intolerance or allergy to study medications<br>• Life expectancy <2 y			to uncoated balloons). Average lesion length was 7.0±5.3 and 6.6±5.5 cm for DEB and control arm, respectively. Procedural success was obtained in all cases. 6 mo quantitative angiography showed that DEB were associated with significantly lower late lumen loss (-0.01 mm; 95% CI: -0.29–0.26 vs. 0.65 mm; 95% CI: 0.37–0.93; p=0.001) and fewer binary restenoses (3 [8.6%] vs. 11 [32.4%]; p=0.01). This translated into a clinically relevant benefit with significantly fewer major adverse events for DEB vs. uncoated balloons up to 12 mo (3 [7.1%] vs. 15 [34.9%]; p<0.01) as well as TL revascularizations (3 [7.1%] vs. 12 [27.9%]; p=0.02).
**VIASTAR**<br>Lammer J, et al. 2013(233)<br>23831445	**Aim**: SFA Viabahn vs. nitinol SES<br><br>**Study type**: RCT<br><br>**Size**: n=141 pts	**Inclusion criteria**: Sx SFA stenosis<br><br>**Exclusion criteria**: The major exclusion criteria were:<br>• Untreated inflow lesions<br>• Any previous stenting or surgery in the target artery, serum creatinine level >2.5 mg/dL<br>• Septicemia<br>• Known intolerance to heparin, antithrombotic study medications, or contrast agents	**Intervention**: Viabahn (heparin coated)<br><br>**Comparator**: SES	**1° endpoint**: 6 and 12 mo primary patency	• No significant difference<br>• Mean±SD lesion length was 19.0±6.3 cm in the Viabahn group and 17.3±6.6 cm in the BMS group. Major complications within 30 d were observed in 1.4%. The 12 mo primary patency rates in the Viabahn and BMS groups were: ITT 70.9% (95% CI: 0.58–0.80) and 55.1% (95% CI: 0.41–0.67) (log-rank test p=0.11); TPP 78.1% (95% CI: 0.65–0.86) and 53.5% (95% CI: 0.39–0.65) (HR: 2.23; 95% CI: 1.14–4.34) (log-rank test p=0.009). In lesions ≥20 cm, (TASC class D), the 12 mo patency rate was significantly longer in VIA pts in the ITT analysis (VIA 71.3% vs. BMS 36.8%; p=0.01) and the TPP analysis (VIA 73.3% vs. BMS 33.3%; p=0.004). Freedom from TL revascularization was 84.6% for Viabahn (95% CI: 0.72–0.91) vs. 77.0% for BMS (95% CI: 0.63–0.85; p=0.37). The ABI in the Viabahn group significantly increased to 0.94±0.23 compared with the BMS group (0.85±0.23; p<0.05) at 12 mo.
**VIBRANT**<br>Geraghty PJ, et al.<br>2013(234)<br>23676191	**Aim**: Viabahn vs. SES<br><br>**Study type**: RCT<br><br>**Size**: n=184 pts	**Inclusion criteria**: Sx complex superficial femoral artery disease (TASC I class C and D lesions, accompanied by IC or ischemic rest pain)	**Intervention**: Viabahn (non-heparin coated)<br><br>**Comparator**: SES	**1° endpoint**: Patency, limb hemodynamics, and QoL were evaluated at 1, 6, 12, 24, and 36 mo following intervention.	• No significant difference<br>• The average treated lesion measured 18±8 cm in length, and 58.8% of lesions displayed segmental or complete occlusion. At 3 y, primary patency rates (defined by peak systolic velocity ratio ≤2.0 and no TL revascularization) did not significantly

© American Heart Association, Inc. and American College of Cardiology Foundation

		**Exclusion criteria**: Occluded popliteal artery of <1 infrapop artery patent to the ankle			differ between pts treated with the VIABAHN stent graft and those who received a bare nitinol stent (24.2% vs. 25.9%; p=0.392). Stent fractures were significantly more common in bare nitinol stents (50.0%) than in the VIABAHN endoprostheses (2.6%). Primary-assisted patency rates were higher in those receiving bare nitinol stents than the VIABAHN stent graft (88.8% vs. 69.8%; p=0.04), although secondary patency rates did not differ between bare nitinol stent and stent graft recipients (89.3% vs. 79.5%; p=0.304). There were no instances of procedure-related mortality or amputation. The hemodynamic improvement and quality measures improved equally in both groups.
Saxon RR, et al. 2008(235) 18503895	**Aim**: SFA: Viabahn vs. PTA<br><br>**Study type**: RCT<br><br>**Size**: n=197 pts	**Inclusion criteria**: Sx SFA PAD<br><br>**Exclusion criteria**: Occluded popliteal artery of <1 infrapop artery patent to the ankle	**Intervention**: Viabahn<br><br>**Comparator**: PTA	**1° endpoint**: 12 mo duplex primary patency	• Viabahn superior to PTA alone<br>• The stent-graft group had a significantly higher technical success rate (95% vs. 66%, p<0.0001) and 1 y primary vessel patency rate at duplex ultrasonography (65% vs. 40%, p=0.0003). A patency benefit was seen for lesions at least 3 cm long. At 12 mo, chronic limb ischemia status was 15% further improved for the stent-graft group (p=0.003). There were no significant differences between treatment groups with regard to the occurrence of early or late major adverse events.
Kedora J, et al. 2007(236) 17126520	**Aim**: SFA: Viabhan vs. synthetic fem-pop bypass<br><br>**Study type**: RCT<br><br>**Size**: n=86 pts	**Inclusion criteria**: Sx femoral-popliteal arterial occlusive disease<br><br>**Exclusion criteria**:<br>• No aorto-iliac disease<br>• <1 infrapop artery patent to ankle	**Intervention**: Viabahn<br><br>**Comparator**: Synthetic fem-pop bypass	**1° endpoint**: 12 mo duplex primary patency	• No difference<br>• Pts were monitored for a median of 18 mo. No statistical difference was found in the primary patency (p=0.895) or secondary patency (p=0.861) between the 2 treatment groups. Primary patency at 3, 6, 9, and 12 mo of follow-up was 84%, 82%, 75.6%, and 73.5% for the stent graft group and 90%, 81.8%, 79.7%, and 74.2% for the femoral-popliteal surgical group. 13 pts in the stent graft group had 14 reinterventions, and 12 reinterventions occurred in the surgical group. This resulted in secondary patency rates of 83.9% for the stent graft group and 83.7% for the surgical group at the 12 mo follow-up.
**Zilver PTX**	**Aim**: SFA DES vs.	**Inclusion criteria**: Sx	**Intervention**: DES	**1° endpoint**: 2 mo rates of	• DES is superior to PTA±BMS

© American Heart Association, Inc. and American College of Cardiology Foundation

Dake MD, et al. 2011(237) 21953370	PTA w provisional BMS<br><br>**Study type**: RCT<br><br>**Size**: n=474 pts	fem/pop PAD<br><br>**Exclusion criteria**: Major exclusion criteria included:<br>• Utreated >50% DS of the inflow tract<br>• Lesion pretreatment with adjunctive devices<br>• Previous target vessel stenting	(no polymer)<br><br>**Comparator**: PTA w provisional BMS	event-free survival and patency	• Pts were randomly assigned to primary DES implantation (n=236) or PTA (n=238). Demographics and lesion characteristics were similar between groups (eg, average lesion length, approximately 65±40 mm). 120 pts had acute PTA failure and underwent secondary random assignment to provisional DES (n=61) or BMS (n=59). Primary endpoints were the 12 mo rates of event free survival and patency in the primary DES and PTA groups. Compared with the PTA group, the primary DES group exhibited superior 12 mo event free survival (90.4% vs. 82.6%; p=0.004) and primary patency (83.1% vs. 32.8%; p<0.001), satisfying the primary hypotheses. In the secondary evaluations, (1) the primary DES group exhibited superior clinical benefit compared with the PTA group (88.3% vs. 75.8%; p<0.001), (2) the provisional DES group exhibited superior primary patency (89.9% vs. 73.0%; p=0.01) and superior clinical benefit (90.5% and 72.3%; p=0.009) compared with the provisional BMS group, and (3) the stent fracture rate (both DES and BMS) was 0.9% (4/457).
Dake MD, et al. 2015(238) PMC4823823	**Aim**: SFA DES vs. PTA w provisional BMS<br><br>**Study type**: RCT<br><br>**Size**: n=474 pts	**Inclusion criteria**: Sx fem/pop PAD<br><br>**Exclusion criteria**: Major exclusion criteria included:<br>• Utreated >50% DS of the inflow tract<br>• Lesion pretreatment with adjunctive devices<br>• Previous target vessel stenting	**Intervention**: DES (no polymer)<br><br>**Comparator**: PTA w provisional BMS	**1° endpoint**: 2 mo rates of event-free survival and patency	• 5-y results from Zilver PTX study show long-term information previously unavailable.<br>• Zilver PTX DES provided sustained safety and clinical durability in comparison with standard endovascular treatments
**SIROCCO** Duda SH, et al. 2006(239) 17154704	**Aim**: SFA: DES vs. BMS<br><br>**Study type**: RCT<br><br>**Size**: n=93 pts	**Inclusion criteria**: Chronic limb ischemia and SFA occlusions or stenoses TASC C<br><br>**Exclusion criteria**: Lesions	**Intervention**: DES<br><br>**Comparator**: BMS	**1° endpoint**: Freedom from restenosis	• No meaningful difference between sirolimus DES vs. BMS<br>• Both the sirolimus-eluting and the bare SMART stents were effective in revascularizing the diseased SFA and in sustaining freedom from restenosis. For both types of stents, improvements

© American Heart Association, Inc. and American College of Cardiology Foundation

		>20 cm			in ABI and symptoms of claudication were maintained over 24 mo (median 24 mo ABI 0.96 for the sirolimus group vs. 0.87 for the bare stent group, p>0.05). At 24 mo, the restenosis rate in the sirolimus group was 22.9% vs. 21.1% in the bare stent group (p>0.05). The cumulative in-stent restenosis rates according to duplex ultrasound were 4.7%, 9.0%, 15.6%, and 21.9%, respectively, at 6, 9, 18, and 24 mo; the rates did not differ significantly between the treatment groups. The TLR rate for the sirolimus group was 6% and for the bare stent group 13%; the TVR rates were somewhat higher: 13% and 22%, respectively. Mortality rates did not differ significantly between the groups.
Tepe G, et al. 2008(240) 18272892	**Aim**: SFA: PTA vs. PTA with balloon dipped in paclitaxel<br><br>**Study type**: RCT<br><br>**Size**: n=154 pts	**Inclusion criteria**: Pts with Rutherford stages 1–5 sx & stenosis or occlusion of a femoropopliteal artery<br><br>**Exclusion criteria**:<br>• Poor inflow; absence of a patent crural artery<br>• Acute onset of symptoms<br>• Pregnancy<br>• Life expectancy of >1 y<br>• Contraindications to required medication	**Intervention**: Paclitaxel dipped balloon<br><br>**Comparator**: PTA	**1° endpoint**: Angiographic restenosis at 6 mo and TVR	• DCB superior<br>• The mean (±SD) age of the pts was 68±8 y, 24% were smokers, and 49% had DM. 27% of the lesions were total occlusions, and 36% were restenotic lesions. The mean lesion length was 7.4±6.5 cm. There were no significant differences in baseline characteristics between the groups. There were no adverse events attributable to the paclitaxel-coated balloons. At 6 mo, the mean late lumen loss was 1.7±1.8 mm in the control group, as compared with 0.4±1.2 mm (p<0.001) in the group treated with paclitaxel-coated balloons and 2.2±1.6 mm (p=0.11) in the group treated with paclitaxel in the contrast medium. The rate of revascularization of TLs at 6 mo was 20 of 54 (37%) in the control group, 2 of 48 (4%) in the group treated with paclitaxel-coated balloons (p<0.001 vs. control), and 15 of 52 (29%) in the group treated with paclitaxel in the contrast medium (p=0.41 vs. control); at 24 mo, the rates increased to 28 of 54 (52%), 7 of 48 (15%), and 21 of 52 (40%)

© American Heart Association, Inc. and American College of Cardiology Foundation

**EXCITE ISR**<br>Dippel EJ, et al. 2015(241)<br>25499305	**Aim**: SFA ISR: ELA+PTA vs. PTA<br><br>**Study type**: RCT<br><br>**Size**: n=250 pts	**Inclusion criteria**: Rutherford Class 1–4 SFA ISR<br><br>**Exclusion criteria**:<br>• Pregnancy<br>• ALI<br>• Life expectancy <12 mo<br>• Cerebrovascular accidents or MI 60 d prior to procedure<br>• Contraindications or allergies that could affect the procedure<br>• Uncontrolled hypercoagulability<br>• Systemic infection in TL<br>• Previous treatment to the target vessel within 3 mo prior to study procedure<br>• Serum creatinine ≥2.5 mg/dL unless dialysis-dependent<br>• Aneurysm within TL<br>• DES or covered stents in the TL<br>• Planned or predicted cardiac surgery or interventions prior to completion of 30 d follow-up<br>• Grade 4/5 stent fracture affecting target stent or proximal to the target stent.	**Intervention**: ELA+PTA<br><br>**Comparator**: PTA	**1° endpoint**: 6 mo TLR<br><br>**Safety endpoint**: 30 d MACE	• ELA+PTA superior to PTA alone for SFA ISR<br>• Study enrollment was stopped at 250 pts due to early efficacy demonstrated at a prospectively-specified interim analysis. A total of 169 ELA+PTA pts (62.7% male; mean age 68.5±9.8 y) and 81 PTA pts (61.7% male; mean age 67.8±10.3 y) were enrolled. Mean lesion length was 19.6±12.0 cm vs. 19.3±11.9 cm, and 30.5% vs. 36.8% of pts exhibited total occlusion. ELA+PTA pts demonstrated superior procedural success (93.5% vs. 82.7%; p=0.01) with significantly fewer procedural complications. ELA+PTA and PTA pt 6-mo freedom from TLR was 73.5% vs. 51.8% (p<0.005), and 30 d major adverse event rates were 5.8% vs. 20.5% (p<0.001), respectively. ELA+PTA was associated with a 52% reduction in TLR (HR: 0.48; 95% CI: 0.31–0.74).
**COBRA**<br>Banerjee S, et al.<br>2012(242)<br>22981558	**Aim**: SFA: PTAS vs. PTAS with Cryo PTA<br><br>**Study type**: RCT<br><br>**Size**: n=74 pts	**Inclusion criteria**:<br>• DM<br>• Sx PAD<br>• Superficial femoral artery lesions requiring implantation of stents >5 mm in diameter and >60 mm in length.	**Intervention**: Cryoplasty PTA<br><br>**Comparator**: PTA	**1° endpoint**: 12 mo binary restenosis	• Post-dilation with cryoplasty balloon reduced binary restenosis compared to conventional balloon angioplasty<br>• 74 pts, with 90 stented superficial femoral artery lesions, were randomly assigned to post-dilation using cryoplasty (n=45 lesions) or conventional balloons (n=45 lesions). Mean lesion length was 148±98 mm, mean stented length was 190±116 mm, mean stent diameter was 6.1±0.4 mm, and

© American Heart Association, Inc. and American College of Cardiology Foundation

		**Exclusion criteria**:<br>• Allergic to ASA, clopidogrel, or iodine-based radiographic contrast<br>• Had obstructive (≥50% diameter stenosis) iliofemoral artery disease<br>• Absence ≥1 vessel infrapopliteal run-off. All pts had radio-opaque tape in the imaging field as a reference for determining vessel dimensions.			50% of the lesions were total occlusions. Post-dilation balloon diameters were 5.23±0.51 mm vs. 5.51±0.72 mm in the cryoplasty and conventional balloon angioplasty groups, respectively (p=0.02). At 12 mo, binary restenosis was significantly lower in the cryoplasty group (29.3% vs. 55.8%; p=0.01; OR: 0.36; 95% CI: 0.15–0.89).
Whyman MR, et al.<br>1996(243)<br>8760978	**Aim**: Compare PTA vs. Med Tx for treadmill distance until onset of claudication, treadmill MWD, pt reported MWD, ABI, QoL (NHP) and Duplex measured extent of occlusive disease.<br><br>**Study type**: RCT<br><br>**Size**: n=62 pts (30 PTA+Meds, 32 Med Tx) 47 femoral; 15 iliac	**Inclusion criteria**:<br>• Unilateral IC<br>• Short stenoses<br><br>**Exclusion criteria**:<br>• Previous angioplasty or arterial surgery to the sx leg<br>• MI within 6 mo<br>• Pts taking oral anticoagulants<br>• Duration of symptoms <1 mo<br>• Inability to manage the treadmill examination<br>• Any psychiatric illness or other reason making follow-up difficult	**Intervention**: PTA+medical therapy<br><br>**Comparator**: Medical therapy (Medical therapy=ASA+advise on smoking and exercise)	**1° endpoint**: Max treadmill time to onset of claudication at 6 mo follow-up p<0.01	• More PTA pt were asx on treadmill at 6 mo (p≤0.01)<br>• More PTA pt had no claudication at 6 mo (p≤0.05)<br>• ABI higher in PTA group at 6 mo (p≤0.05)<br>• Lower Nottingham Health Score pain scores at 6 mo in PTA group (p≤0.05)
Whyman MR, et al.<br>1997(244)<br>9357454	**Aim**: 2 y follow-up of above study<br><br>**Study type**: RCT<br><br>**Size**: n=62 pts (30 PTA+Meds, 32 Med Tx) 47 femoral; 15 iliac	**Inclusion criteria**:<br>• Unilateral IC<br>• Short stenoses<br><br>**Exclusion criteria**:<br>• Previous angioplasty or arterial surgery to the sx leg<br>• MI within 6 mo<br>• Pts taking oral	**Intervention**: PTA+medical therapy<br><br>**Comparator**: Medical therapy (Medical therapy=ASA+advise on smoking and exercise)	**1° endpoint**: Max treadmill time to onset of claudication at 2 y follow-up<br><br>**Safety endpoint**: Non-reported	• No difference in pt reported MWD, treadmill onset to claudication, treadmill MWD, or ABPI (p>0.05)<br>• No difference in NHP QoL

© American Heart Association, Inc. and American College of Cardiology Foundation

		anticoagulants<br>• Duration of symptoms <1 mo<br>• Inability to manage the treadmill examination<br>• Any psychiatric illness or other reason making follow-up difficult			
Perkins, JM, et al.<br>2011(245)<br>21855020	**Aim**: Compare ABI and Walking distance in PAD pts treated with PTA vs. exercise training<br><br>**Study type**: RCT<br><br>**Size**: n=56 pts	**Inclusion criteria**: Unilateral claudication lesion(s) on angiography suitable for angioplasty, as agreed by surgeons and radiologists<br><br>**Exclusion criteria**: Not specified in article	**Intervention**: PTA<br><br>**Comparator:** Exercise training (Supervised exercise classes 2x/wk for the first 6 mo. After this, attendance was required on a regular basis according to the pt's progress. Each class lasted 30 min. Dynamic leg exercises were performed, with the intensity of exercise increasing as the pt's exercise tolerance improved. Pts were also encouraged to perform the same exercises at home on a regular basis)	**1° endpoint**: Better ABI in PTA group at 15 mo; no difference in ABI, distance to claudication or MWD at 6 y follow-up	• Small study<br>• No difference in endpoints at 6 y follow-up (only 37 pts followed to 6 y<br>• PTA only (no stents or med Tx)
Spronk S, et al.<br>2009(246)<br>19188327	**Aim**: To compare clinical success, functional capacity, and QoL during 12 mo after revascularization or supervised exercise training in pts with IC	**Inclusion criteria**:<br>• IC<br>• Max PFWD <350 m<br>• ABI <0.9<br><br>**Exclusion criteria**:<br>• AAA<br>• Life incapacitating cardiac disease (≥NYHA class III)	**Intervention**: PTA with provisional stent<br><br>**Comparator**: Hospital based supervised exercise training	**1° endpoint**: Improvement in one Rutherford category<br><br>**Safety endpoint**: Functional capacity defined in terms of ABI, maximum PFD, and MWD SF-36 QoL	• At 1 wk endo superior<br>• By 12 mo no difference<br>• 2010 correction of statistical methods—better for exercise group—still no difference at 12 mo

© American Heart Association, Inc. and American College of Cardiology Foundation

	**Study type**: RCT<br><br>**Size**: n=76 endo; n=75 hospital based supervised exercise	• Multilevel disease (i.e., same-side stenoses at both the iliac and femoral levels, requiring multiple revascularization procedures)<br>• Isolated tibial artery disease<br>• Lesions deemed unsuitable for revascularization (iliac or femoropopliteal TASC type D and some TASC type B and/or C lesions, such as a unilateral external iliac occlusion that involved the origins of the internal iliac and/or common femoral artery or single or multiple femoral popliteal lesions in the absence of continuous tibial vessels to improve inflow for a distal bypass procedure)<br>• Prior treatment for the lesion (including exercise training)			
Spronk S, et al. 2008(247)<br>18771879	**Aim**: Cost-effectiveness analysis of above study<br><br>**Study type**: RCT<br><br>**Size**: n=76 endo; n=75 hospital based supervised exercise	**Inclusion criteria**:<br>• IC<br>• Max PFWD <350 m<br>• ABI <0.9<br><br>**Exclusion criteria**:<br>• AAA<br>• Life incapacitating cardiac disease (≥NYHA class III)<br>• Multilevel disease (i.e., same-side stenoses at both the iliac and femoral levels, requiring multiple revascularization procedures)<br>• Isolated tibial artery disease<br>• Lesions deemed unsuitable for revascularization (iliac or femoropopliteal TASC type D	**Intervention**: PTA with provisional stent<br><br>**Comparator**: Hospital based supervised exercise training	**1° endpoint**: Mean improvement of health-related QoL and functional capacity over a 12 mo period, cumulative 12 mo costs, and incremental costs per QALY<br><br>**Safety endpoint**: Not reported	• Endo costs more than exercise program when adjusted for QALY however this study had no difference between QoL at 12 mo

© American Heart Association, Inc. and American College of Cardiology Foundation

		and some TASC type B and/or C lesions, such as a unilateral external iliac occlusion that involved the origins of the internal iliac and/or common femoral artery or single or multiple femoral popliteal lesions in the absence of continuous tibial vessels to improve inflow for a distal bypass procedure)<br>• Prior treatment for the lesion (including exercise training)			
Gelin J, et al. 2001(248)<br>11472042	**Aim:** Invasive vs. supervised exercise vs. control<br><br>**Study type**: RCT single center<br><br>**Size:** Invasive (n=87 pts; 17 were endo) vs. meds (n=89) vs. control (n=89)	**Inclusion criteria**: IC with ABI <0.6<br><br>**Exclusion criteria:** Pts with a medical Hx contraindicating surgery and/or with other disorders severely limiting walking evaluation on a treadmill	**Intervention:** Surgery or endo<br><br>**Comparator:** Supervised exercise (3 30 min sessions for 6 mo and then 2 sessions per wk)<br><br>**Control:** Advise on risk factor management and walking	**1° endpoint:** ABI (p<0.01) and max treadmill time (p<0.01) improved only in invasive group<br><br>**Safety endpoint:** No difference in 1 y mortality	• Only 59% of exercise pts competed training
Taft C, et al. 2001(249)<br>11472043	**Aim:** QoL analysis of above study<br><br>**Study type:** : RCT single center<br><br>**Size:** Invasive (n=87 pts; 17 were endo) vs. Meds (n=89) vs. Control (n=89)	**Inclusion criteria**: IC with ABI <0.6<br><br>**Exclusion criteria:** Pts with a medical Hx contraindicating surgery and/or with other disorders severely limiting walking evaluation on a treadmill	**Intervention:** Surgery or endo<br><br>**Comparator:** Supervised exercise (3 30 min sessions for 6 mo and then 2 sessions per wk)<br><br>**Control:** Advise on risk factor management and walking	**1° endpoint:** Invasive therapy improved disease specific symptoms (waling pain) but no difference in other aspect of QoL	N/A

© American Heart Association, Inc. and American College of Cardiology Foundation

**EXACT**<br>Hobbs, et al.<br>2006(250)<br>16414385	**Aim**: Endo vs. Meds<br><br>**Study type**: RCT<br><br>**Size**: Endovascular revascularization+best medical therapy (n=9)<br>Best medical therapy (n=7)	**Inclusion criteria**: PAD pts with IC<br><br>**Exclusion criteria**: N/A	**Intervention**: PTA+meds<br><br>**Comparator**: Optimal medical therapy	**1° endpoint**: At 6 mo PTA group has better ABI (p=0.013) and MWD (p=0.008)	N/A
**CLEVER**<br>Murphy TP, et al.<br>2012(187)<br>22090168	**Aim**: Supervised exercise vs. stent vs. meds<br><br>**Study type**: RCT<br><br>**Size**: Meds (n=22) vs. SE (n=42) vs. stent (N=46)	**Inclusion criteria**:<br>• Severe IC (defined as ability to walk ≥2 but <11 min on a graded treadmill test using the Gardner protocol)<br>• Objective evidence of a hemodynamically significant aortoiliac arterial stenosis<br><br>**Exclusion criteria**: CLI or comorbid conditions that limited walking ability	**Intervention**: Supervised exercise<br><br>**Comparator**: Stenting vs. medical therapy alone	**1° endpoint**: Change in peak walking time a 6 mo compared to baseline (meds 1.2±2.6 mins, SE 5.8±4.6, ST 3.7±4.9) meds vs. SE p<0.001<br>SE vs. ST p=0.022	• Both SE and ST experienced improvement in QoL; peak walking time increase was larger for SE
**CLEVER 18 mo F/U**<br>Murphy TP, et al.<br>2015(186)<br>25766947	**Aim**: Supervised exercise vs. stent vs. meds<br><br>**Study type**: RCT<br><br>**Size**: Meds (n=22) vs. SE (n=42) vs. stent (n=46)	**Inclusion criteria**: Severe IC (defined as ability to walk ≥2 but <11 min on a graded treadmill test using the Gardner protocol) and objective evidence of a hemodynamically significant aortoiliac arterial stenosis<br><br>**Exclusion criteria**: CLI or comorbid conditions that limited walking ability	**Intervention**: Supervised exercise<br><br>**Comparator**: Stenting vs. Medical therapy alone	**1° endpoint**: Change in peak walking time at 18 mo compared to baseline (meds 0.2±2.1mins, SE 5.0±5.4 min, ST 3.7±4.7) meds vs. SE p<0.001<br>meds vs. ST p=0.04<br>SE vs. ST p=0.16	N/A
**OBACT**<br>Nylaende M, et al.<br>2007(251)<br>17055756	**Aim**: Endo vs. OMT<br><br>**Study type**: RCT single center	**Inclusion criteria**:<br>• PAD with disabling IC<br>• ABI <0.9 and peak walking distance <400 m<br>• Both Aortoiliac and	**Intervention**: PTA<br><br>**Comparator**: Medical therapy	**1° endpoint**:<br>• PFWD, MWD at 3, 12, and 24 mo PFWD, MWD, and ABI were improved in PTA group compared to	• On QoL questionnaires pain was less in PTA group

© American Heart Association, Inc. and American College of Cardiology Foundation

	**Size**: Endovascular revascularization+optimal medical therapy (n=28)<br>Optimal medical therapy (n=28)	femoropopliteal diseased population was included.<br><br>**Exclusion criteria**:<br>• Subjective PFWD >400 m<br>• CLI<br>• Previous vascular or endovascular surgery<br>• DM ulcer<br>• Other physical disability abrogating organized exercise<br>• Use of warfarin<br>• Renal Insufficiency		Med Tx;<br>• 24 mo p values PFWD p=0.0001, MWD p=0.0009, ABI p=0.0013	
**MIMIC**<br>Greenhalgh RM, et al. 2008(252)<br>19022184	**Aim**: Endo vs. SE<br><br>**Study type**: RCT single center<br><br>**Size**: Endovascular revascularization (n=87) multiple types of procedures vs. Supervised exercise (n=88) Treadmill walking training 3 times per wk for 6 mo	**Inclusion criteria**:<br>• PAD pts with IC (ABI <0.9)<br>• 93 pts with femoropopliteal disease, 34 pts with aortoiliac disease<br><br>**Exclusion criteria**:<br>• Symptoms too mild to consider angioplasty or so severe that intervention was mandatory<br>• CLI (absolute Doppler BP <50 mm hg or presence of ulcers or gangrene with a Doppler pressure >50 mm hg)<br>• Concomitant disease (e.g., musculoskeletal or cardiac) which prohibits exercise.	**Intervention**: PTA±stent<br><br>**Comparator**: SE once a wk for 6 mo	**1° endpoint**:<br>• 24 mo average walking time and initial claudication distance<br>• Fem-pop disease AWD was 38% greater with PTA (p=0.04) and ICD was longer with PTA (p=0.004)<br>• Aorto-iliac disease AWD was 78% greater with PTA (p=0.05) and ICD was longer with PTA(p=0.05)	N/A
Kruidenier LM, et al. 2011(253)<br>21571547	**Aim**: Endo vs. Endo+SE<br><br>**Study type**: RCT single center<br><br>**Size**: Endovascular revascularization (n=35) Consisted of	**Inclusion criteria**: PAD pts with Rutherford 1–4<br><br>**Exclusion criteria**:<br>• Hx of or current participation in a SET program<br>• Serious cardiopulmonary comorbidity (NYHA III–IV)	**Intervention**: Endo+SE<br><br>**Comparator**: Endo	**1° endpoint**:<br>• 6 mo absolute walking distance<br>• Endo+SE superior to endo alone (p=0.011)	N/A

© American Heart Association, Inc. and American College of Cardiology Foundation

	iliac angioplasty with selective stent placement for iliac stenoses, angioplasty with primary stent placement for SFA stenoses, or recanalization with primary stent placement for iliac and femoral occlusions<br>Vs. Endovascular revascularization+supervised exercise (n=35)<br>Nonspecified exercise program 2x/wk for 6 mo	• Other serious comorbidity preventing physical activity<br>• Insufficient knowledge of the Dutch language<br>• No insurance for SET<br>• Major amputation or tissue loss.			
Mazari FA, et al. 2012(254) 22021102	**Aim**: Endo vs. SE vs. Endo+SE<br><br>**Study type**: RCT single center<br><br>**Size**: Endovascular revascularization (n=60), SE (n=60) Endovascular revascularization+supervised exercise (n=58)	**Inclusion criteria**: PAD with sx unilateral claudication suitable for angioplasty and femoropopliteal lesions<br><br>**Exclusion criteria:**<br>• Critical ischemia<br>• Incapacitating systemic disease<br>• Inability to tolerate treadmill testing<br>• Ischemic changes on ECG during treadmill testing<br>• Ipsilateral surgery/PTA in previous 6 mo	**Intervention**: Endo+SE<br><br>**Comparator**: Endo alone vs. SE alone<br><br>Endovascular therapy: Percutaneous transluminal angioplasty<br>Supervised exercise therapy: Circuit of exercises 3x/ wk for 12 wk<br><br>Concomitant therapy: All pts were prescribed antiplatelet therapy	**1° endpoint**: ICD, MWD, repeat revascular, peri-procedural complications	• No significant difference at 12 mo in ICD and MWD or QoL

© American Heart Association, Inc. and American College of Cardiology Foundation

			(ASA and/or clopidogrel), received smoking cessation advice and support (including nicotine replacement therapy and NHS smoking cessation program), and risk factor		
Mazari FA, et al. 2010(195) [19762206](19762206)	**Aim:** 3 mo data for above trial<br><br>**Study type:** RCT<br><br>**Size:** n=178 pts	**Inclusion criteria:** PAD with sx unilateral claudication suitable for angioplasty<br><br>**Exclusion criteria:**<br>• Critical ischemia<br>• Incapacitating systemic disease<br>• Inability to tolerate treadmill testing<br>• Ischemic changes on ECG during treadmill testing<br>• Ipsilateral surgery/PTA in previous 6 mo	**Intervention:** Endo+SE<br><br>**Comparator:**<br>• Endo alone vs. SE alone<br>• Endovascular therapy: Percutaneous transluminal angioplasty Supervised exercise therapy: Circuit of exercises 3 times per wk for 12 wk<br>• Concomitant therapy: All pts were prescribed antiplatelet therapy (ASA and/or clopidogrel), received smoking cessation advice and support (including nicotine replacement therapy and NHS smoking cessation program), and risk factor	**1° endpoint:** ICD, MWD, repeat revascular, peri-procedural complications<br><br>**Safety endpoint:** None reported	At 3 mo PTA + SEP provided greater improvement in claudication than SEP or PTA alone.<br>See above for 12 mo results

© American Heart Association, Inc. and American College of Cardiology Foundation

Nordanstig J, et al.<br>2011(255)<br>21397530	**Aim**: Invasive+OMT vs. optimal medical tx<br><br>**Study type**: RCT multicenter<br><br>**Size**: Inv (n=100) vs. OMT(n=101)	**Inclusion criteria**: IC >6 mo<br><br>**Exclusion criteria:**<br>• Age ≥85 y<br>• Incorrect Dx<br>• Other disorders limiting walking performance<br>• Pts with ≥2 previously occluded vascular reconstructions.	**Intervention**: Invasive+OMT<br><br>**Comparator**:<br>• OMT<br>• Revascularization: In general, aorto-iliac TASC A and B lesions were treated endovascularly and TASC C and D lesions with surgery. Femoropopliteal TASC A lesions were offered angioplasty, whereas TASC BeD lesions usually were treated surgically. For lesions in the common femoral artery, endarterectomy with or without patch angioplasty was used.<br>• Optimal medical therapy: ASA 75 mg daily (or ticlopidine if contraindication to ASA). Smokers were offered participation in a smoking cessation support program and received verbal and written information with smoking cessation advice. Hypertension, DM, and hyperlipidemia	**1° endpoint:** 2 y Mean Walking Performance and QoL<br><br>MWP was not significantly (p=0.104) improved in the INV vs. the NON group. 2 SF-36 physical subscales, Bodily Pain (p<0.01) and Role Physical (p<0.05) improved significantly more in the INV vs. the NON group. There were 7% crossovers against the study protocol in the INV group.	N/A

© American Heart Association, Inc. and American College of Cardiology Foundation

			were managed according to national guidelines.<br>Verbal training advice and a written training program for IC. Instructed to walk at least 1 H/d and to walk up to their maximal claudication distance as often as possible and to perform an additional exercise program at home several times per d.		
**IRONIC**<br>Nordanstig J, et al.<br>2014(256)<br>25095886	**Aim**: Invasive+OMT vs. optimal medical tx<br><br>**Study type**: RCT (single center)<br><br>**Size**: Invasive (n=79) vs. OMT (n=79)	**Inclusion criteria**: IC >6 mo<br><br>**Exclusion criteria:**<br>• Very mild symptoms<br>• Symptoms so severe that invasive treatment was considered mandatory (main criteria according to protocol: inability to work because of IC, subcritical ischemia with occasional rest pain, infrarenal aortic thrombosis)<br>• Weight >120 kg (maximum possible load on treadmill)<br>• ≥2 previously failed ipsilateral vascular interventions	**Intervention**: Endo except for TASC D<br><br>79 allocated to invasive Rx<br>70 received intervention:<br>• 52 pts Endovascular<br>• 16 pts open surgery.<br>• 2 pts hybrid<br><br>**Comparator**: OMT	**1° endpoint**: SF 36 (p<0.001) and VascularuQoL (p<0.01) at 12 mo better with Inv	• Distance to onset of claudication better with Inv. Invasive (+124 m) vs. the noninvasive (+50 m) group (p=0.003)<br>• No difference Inv vs. Meds for MWD change<br>• Invasive therapy group included 18 pts treated with surgical and hybrid approach to invasive Rx<br>• Outcomes not stratified by surgical vs. endovsacular procedures.<br>• Both aortoiliac and femoropoliteal disease pts were enrolled. Pragmatic design to include large IC population independent of whether surgical or endovascular approach was required
Malgor RD, et al 2015(257)<br>25721067	**Aim**: Endo vs. surgical vs. SE vs. Meds<br><br>**Study type**: Meta-analysis of RCTs	**Inclusion criteria**: RCTs of IC pts<br><br>**Exclusion criteria**: Trials exclusively enrolling pts with CLI, defined as rest pain or tissue loss	**Intervention**: Endo vs. surgical vs. SE vs. Meds	**1° endpoint**:<br>• Open surgery, endovascular therapy, and exercise therapy were superior to medical management in terms of walking distance and	• Minimal data on cost effectiveness.<br>• Efficacy of surgery, endovascular and exercise therapy seemed to be superior to medical mgmt for walking distance, pain and claudication<br>• Evidence is sparse supporting superiority of one of three approaches<br>• Isolated iliac or femorpopliteal disease pts. may

© American Heart Association, Inc. and American College of Cardiology Foundation

	**Size:** n=8 systematic reviews and 12 trials enrolling 1,548 pts			claudication<br><br>**Results:**<br>RCTs for Surgery (with physical training):<br>• Max. and symptom free walking distance improved vs. Medical management alone or exercise alone<br>• ABI improved vs. surgery alone but not exercise<br>• Endovascular approaches with medical mgmt. or exercise: Combination of both may be a better approach<br>• Endovascular vs. open surgery:<br>• Studies generally showed open bypass had significantly longer hospital stay, high complications and a high 30-d mortality.<br>• Some SRs had conflicting info about 30-d mortality but patency was generally better in surgical arm.<br>• Revasc with medical mgmt or exercise:<br>• Invasive revasc generally increased leg BP and flow parameters, better SF 36, overall QoL score and IC distance but not MWD<br><br>**Safety endpoint:** Not reported	do better than combined disease according to the limited data.

© American Heart Association, Inc. and American College of Cardiology Foundation

Vemulapalli S, et al 2015(258) 25963038	**Aim**: Endo vs. surgical vs. exercise vs. Meds<br><br>**Study type**: Meta-analysis of RCTs<br><br>**Size**: n=35 studies of 7,475 pts	**Inclusion criteria**: IC pts<br><br>**Exclusion criteria**: N/A	**Intervention**: Endo vs. surgical vs. exercise vs. Meds<br><br>**Comparator**: Medication alone	**1° endpoint**: Only exercise improved MWD p=0.01 SF-36 improved in all groups compared to meds (usual care)<br><br>**Safety endpoint**: Not reported	• Authors conclude current RCT data is inconclusive to determine superiority for walking distance or QoL for claudication
McPhail IR, et al. 2001(259) 11300450	**Aim**: Compare the standard LE vascular laboratory treatmill exercise with the office-based active pedal plantarflexion technique<br><br>**Study type**: Prospective, randomized crossover study<br><br>**Size**: n=50 pts (100 LE)	**Inclusion criteria**:<br>• Known or suspected IC<br>• Referred for LE treadmill exercise testing<br><br>**Exclusion criteria**:<br>• Ankle SBP >300 mmHg or >50 mmHg higher than brachial systolic BP<br>• CLI and inability to walk on a treatmill or perform active pedal plantarflexion	**Intervention**: Active pedal plantarflexion<br><br>**Comparator**: LE treadmill exercise testing	**1° endpoint**: Active pedal plantarflexion compared favorably with treadmill exercise for the noninvasive objective assessment of PAOD<br><br>**Safety endpoint**: Not reported	N/A
Schulte KL, et al. 2015(260) 26245919	**Aim**: Compare primary placement of a self-expanding nitinol stent to PTA with bailout stenting in infrapopliteal arteries of pts with severe intermittent claudication or CLI<br><br>**Study type**: RCT<br><br>**Size**: n=92 pts	**Inclusion criteria**:<br>• Pts undergoing treatment for infrapopliteal stenosis in 11 European centers<br><br>**Exclusion criteria**:<br>• N/A	**Intervention**: Primary placement of a self-expanding nitinol stent vs. PTA with bailout stenting	**1° endpoint**: Sustainable clinical improvement after 12 mo, defined as ≥1 category increase for Rutherford category 3 pts, a ≥2 category increase for CLI pts compared with baseline.<br><br>**Safety endpoint**: TLR, mortality, and amputation assessed after 12 mo.	• Sustained improvement at 1 y in 74.3% of the pts treated with primary stenting and in 68.6% of the pts treated with PTA and bailout stenting (p>0.05).<br>• Freedom from TLR (76.6% and 77.6%), mortality (7.4% vs 2.1%), and amputation [8.9% (major 6.7%) vs 13.2% (major 8.7%)] at 1 y were not significantly different.<br>• Primary self-expanding nitinol stenting did not show statistically different clinical outcomes compared to PTA with bailout stenting

AAA indicates abdominal aortic aneurysm; ABF, aorto-bifemoral bypass; ABI, ankle-brachial index; ABPI, ankle-brachia pressure index; AFB, aortobifemoral bypass; AIOD, aortoiliac occlusive disease; ALI, acute limb ischemia; ASA, American Society of Anesthesiologist; AUC, appropriate use criteria; AWD, absolute walking distance; BMS, bare metal stent; BP, blood pressure; CI, confidence interval; CLI, critical limb ischemia; CTA, computed tomography angiography; DCB, drug coated balloon; DEB, drug eluting balloon; DES, drug eluting stent; DS,

© American Heart Association, Inc. and American College of Cardiology Foundation

diameter stenosis; ECG, electrocardiogram; ELA, excimer laser antherectomy; HR, hazard ratio; IC, intermittent claudication; ICD, International Classification of Disease; Inv, intervention group; ISR, in stent restenosis; ITT, intention to treat; JACC, Journal of American College of Cardiology; LE, lower extremity; MACE, major adverse cardiac event; MWD, maximal walking distance; MWP, mean walking performance; N/A, not applicable; NEJM, New England Journal of Medicine; NHP, Nottingham Health Score; NYHA, New York Heart Association; OR, odds ratio; OMT, osteopathic manipulative treatment; PAD, periphery artery disease; PEB, paclitaxel eluting balloon; PFWD, pain free walking distance; PTA, percutaneous angioplasty; PTAS, percutaneous angioplasty stent; PVD, peripheral vascular disease; QALY, quality adjusted life year; QoL, quality of life; RCT, randomized controlled trail; R/PTAS, recanalization, percutaneous transluminal angioplasty, and stenting; RR, relative risk; SE, supervised exercise; SEP, supervised exercise; SES, self-expanding stents; SFA, superficial femoral artery, ST stent revascularization; TASC, transatlantic inter-society consensus; TL, target lesion; TLR, total lesion revascularization; TPP, treatment per-protocol; TVR, target vessel revascularization; and VIA, viabahn treatment.

Evidence Table 36. Nonrandomized Trials, Observational Studies, and/or Registries of Endovascular and Endovascular Versus Noninvasive Treatment of Claudication–Section 8.1.

Study Acronym; Author; Year Published	Study Type/Design; Study Size	Patient Population	Primary Endpoint and Results (include P value; OR or RR; & 95% CI)	Summary/Conclusion Comment(s)
Scheinert D, et al. 2005 (261) 15653033	**Study type**: Prospective series assessing SES fracture incidence<br><br>**Size**: n=93 pts	**Inclusion criteria**: PTAS for claudication or chronic ischemia<br><br>**Exclusion criteria**: None reported	**1° endpoint:**<br>• Stent fracture incidence<br>• Restenosis incidence<br><br>**Results**: The primary patency rate at 12 mo was significantly lower for pts with stent fractures (41.1% vs. 84.3%, p<0.0001).	• Stent fractures predict restenosis<br>• Overall, stent fractures were detected in 45 of 121 treated legs (37.2%). In a stent-based analysis, 64 of 261 stents (24.5%) showed fractures, which were classified as minor (single strut fracture) in 31 cases (48.4%), moderate (fracture of >1 strut) in 17 cases (26.6%), and severe (complete separation of stent segments) in 16 cases (25.0%). Fracture rates were 13.2% for stented length ≤8 cm, 42.4% for stented length >8–16 cm, and 52.0% for stented length >16 cm. In 21 cases (32.8%) there was a restenosis of >50% diameter reduction at the site of stent fracture. In 22 cases (34.4%) with stent fracture there was a total stent reocclusion. According to Kaplan Meier estimates, the primary patency rate at 12 mo was significantly lower for pts with stent fractures (41.1% vs. 84.3%; p<0.0001).
Sakamoto Y, et al. 2013(262) 23536429	**Study type**: Case series evaluating PTAS patency for SFA CTO<br><br>**Size**: n=352 pts	**Inclusion criteria**: SFA CTO undergoing PTAS<br><br>**Exclusion criteria**: None reported. Lack of CTO	**1° endpoint**: 5 y primary and secondary patency rates and the rates of freedom from bypass surgery, major or minor amputation, and all-cause death<br><br>**Results**: Female gender (OR: 1.95; p=0.0051) and mean stent diameter	• Stent diameter predicts restenosis<br>• Mean age was 72±9 y and 31% were female pts. In total, 58% of the pts had DM and 25% were pts with CLI. Occluded length was 194±89 mm, mean total stent length was 198±7 mm, and mean stent diameter was 7.1±0.9 mm. 5 y primary and secondary patency rates were 51.8% and 79.5%, respectively, and the rates of freedom from bypass surgery, major or minor

© American Heart Association, Inc. and American College of Cardiology Foundation

			(OR: 0.77; p=0.0324) were factors strongly associated with restenosis.	amputation, and all-cause death were 96.1%, 96.2%, and 78.4%, respectively. Female sex (OR: 1.95; p=0.0051) and mean stent diameter (OR: 0.77; p=0.0324) were factors strongly associated with restenosis.
Feinglass J, et al. 2000(263) 10642712	**Study type:** Observational multicenter<br><br>**Size:** n=526 pts<br>Majority received medical Tx<br>60 surgical bypass grafting and 44 angioplasty only	**Inclusion criteria:** IC and abnormal ABI<br><br>**Exclusion criteria:** Evidence of CLI	**1° endpoint:** Invasive group had better walking distance and less pain at 18 mo follow-up<br><br>**Results:** The mean ABI improved significantly for the pts who underwent bypass grafting surgery (0.20; p<0.001) and modestly for the pts who underwent angioplasty (0.09; p<0.05) compared to baseline	• Study exclusion criteria were poorly described or not appropriate<br>• Comparator(s) not well described<br>• Diagnostic or therapeutic advances have been made in routine practice since the study was conducted
Giuliano G, et al. 2013 (264) 22790191	**Study type:** Observational Single center<br><br>**Size:** Endovascular revascularization (n=264)<br>Conservative medical therapy (n=215)	**Inclusion criteria:** Fontaine 2 IC, ABI <0.9, >50% stenosis in at least 1 leg artery<br><br>**Exclusion criteria:**<br>• CLI<br>• Previous lower limb revascularization<br>• Recent acute coronary or cerebrovascular ischemic events (6 mo)<br>• Recent coronary or carotid revascularization procedures (6 mo)<br>• Abnormal myocardial ischemia stress test at enrollment<br>• Decompensated HF<br>• Malignant neoplasia or significant hepatic, renal, or inflammatory disease.	**1° endpoint:**<br>• Improved functional status at 21 mo in Endo group<br>• Lower MACE (6.4% vs. 16.3%; p=0.003) in endo group<br><br>**Results:**<br>During a median follow-up of 21 mo (12.0–29.0), the incidence of cardiovascular events was markedly lower in PTA compared to MT pts (6.4% vs. 16.3%; p=0.003)	• Comparators not well described
Koivunen K and Lukkarinen H 2008(265) 18221916	**Study type:** Observational single center<br><br>**Size:** Endovascular	**Inclusion criteria:** PAD and IC<br><br>**Exclusion criteria:** Pts not receiving endo Tx	**1° endpoint:** Nottingham Health Profile Score<br><br>**Results:** 12 mo QoL better in invasive arms	• Comparator not well described<br>• Study did not use a clinically relevant surrogate outcome

© American Heart Association, Inc. and American College of Cardiology Foundation

	revascularization (n=85)<br>Percutaneous transluminal angioplasty or surgery (n=31)<br>Comparator<br>Conservative treatment (N=64) No description provided			
Pell JP and Lee AJ<br>1997(266)<br>9507581	**Study type:**<br>Observational multicenter<br><br>**Size**: Endovascular revascularization (n=19)<br>Percutaneous transluminal angioplasty or surgery (n=19)<br>Comparator<br>Conservative treatment (n=157) No description provided	**Inclusion criteria**: IC<br><br>**Exclusion criteria**: N/A	**1° endpoint**: 6 mo QOL<br><br>**Results**: PTA or surgery provided improved QOL at 6 mo compared to conservative Tx	• Study did not report pts' baseline characteristics<br>• Study did not report pts' comorbid conditions<br>• Comparator(s) not well described
Kalbaugh CA, et al<br>2006(267)<br>16814976	**Study type**: Case series<br><br>**Size**: IC n=54<br>CLI n=30	**Inclusion criteria**: Endo treatment of IC or ALI<br><br>**Exclusion criteria**: None reported	**1° endpoint**: QoL at 1 y<br><br>**Results**: Improved QoL in both IC and ALI compared to baseline	• No comparative arm
Sachs T, et al.<br>2011(268)<br>21880457	**Aim**: Determine national estimates for the costs, utilization, and outcomes of angioplasty and bypass graft for the treatment of claudication<br><br>**Study type**: Retrospective analysis<br><br>**Size**: n=563,143 pts	**Inclusion criteria**: Pts who underwent endo or surgery for PAD based on ICD-9 codes<br><br>**Exclusion**: Atherosclerosis unspecified ICD-I code	**1° endpoint**: Costs and clinical outcomes<br><br>**Results**: Unclear cost analysis as more PTA procedures were performed compared to surgery; lower mortality with PTA	Study limited by methodology; ICD-9 code analysis
Shammas NW, et al.<br>2009(269)<br>19966364	**Aim**: Determine predictors of distal embolization in pts undergoing LE arterial peripheral endovascular	**Inclusion criteria**: Pts undergoing peripheral intervention enrolled in a single center registry	**1° endpoint**: Predictors of distal embolization<br><br>**Results**: Prior Hx of amputation;	Limitation is that this is a single center registry analysis

© American Heart Association, Inc. and American College of Cardiology Foundation

	revasc<br><br>**Study type**: Retrospective analysis; case-control study<br><br>**Size**: n=577 pts	**Exclusion**: None reported	presence of thrombus, and TASC-D lesions predicted distal embolization	
Matsi PJ and Manninen HI 1998(270)<br>9853140	**Aim**: To report complications and predictors of complications in a cohort of pts undergoing endo revasc for claudication or CLI<br><br>**Study type**: Retrospective analysis<br><br>**Size**: n=410 procedures in 295 pts	**Inclusion criteria**: Pts undergoing peripheral intervention at a single center<br><br>**Exclusion**: None reported	**1° endpoint**: Complications and predictors of complications<br><br>**Results**: More complications in pts with occluded arteries compared to stenosed arteries; more bleeding complications in women; pts with CLI had higher mortality compared to claudication; mortality was driven by CAD and cerebrovascular disease	Limitation is that this is a single center retrospective analysis

ABI indicates ankle-brachial index; ALI, acute limb ischemia; CAD, coronary artery disease; CI, confidence interval; CLI, critical limb ischemia; CTO, chronic total occlusion; HF, heart failure; HR, hazard ratio; IC, intermittent claudication; ICD, International Classification of Diseases; JACC, Journal of American College of Cardiology; LE, lower extremity; MACE, major adverse cardiac event; OR, odds ratio; PAD, periphery artery disease; PTA, percutaneous angioplasty; PTAS, percutaneous angioplasty stent; pt, patient; QoL, quality of life; RR, relative risk; SES, self-expanding stents; SFA, superficial femoral artery; and TASC, Trans-Atlantic Inter-Society Consensus.

Evidence Table 37. RCTs Evaluating Surgical Treatment for Claudication–Section 8.1.2.

Study Acronym; Author; Year Published	Aim of Study; Study Type; Study Size (N)	Patient Population	Study Intervention (# patients) / Study Comparator (# patients)	Endpoint Results (Absolute Event Rates, P value; OR or RR; & 95% CI)	Relevant 2° Endpoint (if any); Study Limitations; Adverse Events
**IRONIC**<br>Nordanstig, et al.<br>2014(256)<br>25095886	**Aim**: Compare invasive vs. noninvasive treatment strategies for IC<br><br>**Study type**: RCT (single center, open label)<br><br>**Size**: n=158 pts with stable IC (79 allocated to invasive Rx 79 to noninvasive Rx)	**Inclusion criteria**: Stable (>6 mo) IC symptoms<br><br>**Exclusion criteria**: Mild or severe symptoms	**Intervention**:<br>• Invasive treatment (Open surgical repair reserved for TASC D lesions)<br>• 79 allocated to invasive Rx<br>• 70 received intervention:<br>52 pts	**1° endpoint**: HRQL assessed by SF-36, VascuQol. Greater improvement in VascuQol improved significantly more in invasive group (p<0.01) including 3/5 domain scores; claudication distance improved more in invasive group (+124m vs. +50m); change in MWD not different between groups	• Exclusion criteria somewhat arbitrary<br>• Only 18/158 pts had surgical or hybrid procedures (Total procedures: 1 aortobifemoral bypass, 3 femoral-femoral bypass, 8 ccommon femoral endarterectomy/profundaplasty, 5 femoral-popliteal artery bypass, 1 distal to popliteal

© American Heart Association, Inc. and American College of Cardiology Foundation

			Endovascular<br>16 pts open surgery.<br>2 pts. hybrid<br><br>**Comparator:** Noninvasive treatment (N=79 pts allocated)		bypass)<br>• Outcomes not stratified by surgical vs. endovsacular procedures
Linni K, et al. 2014(271)<br>25101576	**Aim:** Compare clinical and hemodynamic outcome in pts undergoing treatment of CFA atherosclerotic lesions by bioabsorbable stent implantation (BASI group) or by common femoral artery endarterectomy (CFE group).<br><br>**Study type:** RCT (single center, open label)<br><br>**Size:** n=80 pts	**Inclusion criteria:**<br>• Claudication or CLI >2 wk in duration<br>• CFA stenosis or occlusion<br>• Atherosclerosis<br><br>**Exclusion criteria:**<br>• Urgent CLI<br>• Simultaneous aneurysm repair or bypass grafting<br>• Redo CFE<br>• Trauma<br>• Renal insufficiency<br>• Pregnancy	**Intervention:** 1:1 randomization<br><br>**Comparator:** BASI implantation	**1° endpoint:** Surgical site infection (7 for CFE vs. 0 for BASI, p=0.002)	• Technical success (100% CFE vs. 97.5% BASI)<br>• 30d primary patency (100% CFE vs. 92.5% BASI; p=0.038)<br>• 1 y primary patency (100% CFE vs. 80% BASI; p=0.007)<br>• 1 y secondary patency (100% CFE vs. 84% BASI; p=0.01)<br>• Limb salvage (p=0.51)
Gabrielli R, et al. 2012(272)<br>23044257	**Aim:** Evaluated outcomes of RE vs. ENDO interventions on (TASC)-II D femoropopliteal lesions and identified factors predictive of restenosis.<br><br>**Study type:** RCT<br><br>**Size:** n=95 pts	**Inclusion criteria:** TASC-II D lesions (not claudication-specific)<br><br>**Exclusion criteria:**<br>• Previous treatment (endovascular intervention or bypass)<br>• Chronic renal insufficiency (serum creatinine 1.5 mg/dL)<br>• Occlusion of iliac<br>• Common femoral<br>• Popliteal arteries (P2-3 segments)	**Intervention:** Remote endarterectomy with distal endpoint angioplasty and stenting (N=51)<br><br>**Comparator:** Subintimal angioplasty and stenting (N=44)	**1° endpoint:** Primary patency was 76.5% (39 of 51) in RE and 56.8% (25 of 44) in ENDO (HR: 2.6; 95% CI: 0.99–4.2; p=0.05) at 24 mo and was 62.7% (32 of 46) in RE and 47.7% (21 of 40) in ENDO (HR: 1.89; 95% CI: 0.94–3.78; p=0.07) at 36 mo	• 61% of RE and 52% of endo group had Rutherford 4–5 ischemia (<50% of pts had claudication)
**REVAS**<br>Gisbertz SS, et al.<br>2010(273)<br>21035693	**Aim:** Compare RSFAE or supragenicular bypass, for TASC C and D lesions of the SFA<br><br>**Study type:** RCT	**Inclusion criteria:** TASC C and D lesions of the SFA<br><br>**Exclusion criteria:**<br>• Previous surgery or PTA with	**Intervention:** RSFAE<br><br>**Comparator:** Supragenicular bypass	**1° endpoint:** 3 y primary patency after 3 y was 47% for RSFAE and 60% for bypass (p=0.107), assisted primary patency was 63 and 69% (p=0.406), and secondary	• For venous (n=25) and prosthetic grafts (n=30) at 3 y primary patency was 65% and 56 vs. 47% for RSFAE (p=0.143), assisted primary

© American Heart Association, Inc. and American College of Cardiology Foundation

	**Size:** n=116 pts (77 [66%] had IC)	additional stent placement of the target SFA<br>• An SFA diameter <4 mm. SFA occlusion had to start <4 cm from the proximal SFA		patency was 69 and 73% (p=0.541), respectively	patency was 84% and 56 vs. 63% for RSFAE (p=0.052), and secondary patency was 89% and 59 vs. 69% for RSFAE (p=0.046).<br>• Pts were randomized to RSFAE or bypass with the ipsilateral saphenous vein. When the saphenous vein was not available or not suitable, 23 pts received a PTFE bypass
van Det RJ, et al. 2009(274) 19231253	**Aim:** To compare ePTFE prosthesis and collagen-impregnated knitted polyester (Dacron) for AK femoro-popliteal bypass grafts.<br><br>**Study type:** RCT (multicenter)<br><br>**Size:** n=228 bypass grafts (176 [77%] for IC)	**Inclusion criteria:**<br>• Disabling claudication<br>• Rest pain<br>• Tissue loss for whom suprageniculate femoral-popliteal bypass was feasible<br><br>**Exclusion criteria:**<br>• Previous ipsilateral femoro-popliteal procedures<br>• Contraindication to long-term anticoagulant therapy<br>• Life expectancy >1 y and current treatment with chemotherapy or radiotherapy.	**Intervention:** AK femoro-popliteal bypass grafts were randomly allocated to either an ePTFE (n Z 114) or a Dacron (n Z 114) vascular graft<br><br>**Comparator:** N/A	**1° endpoint:** After 5 y, the primary, primary assisted and secondary patency rates were 36% (95% CI: 26%–46%), 46% (CI: 36%–56%) and 51% (95% CI: 41%–61%) for ePTFE and 52% (95% CI: 42%–62%; p=0.04), 66% (95% CI: 56%–76%; p=0.01) and 70% (95% CI: 60–80%; p=0.01) for Dacron, respectively. After 10 y these rates were respectively 28% (95% CI: 18%–38%), 31% (95% CI:19%–43%) and 35% (95% CI: 23%–47%) for ePTFE and 28% (95% CI: 18%–38%), 49% (95% CI: 37%–61%) and 49% (95% CI: 37%–61%) for Dacron.	N/A
**REVAS** Gisbertz SS, et al. 2009(275) 18990592	**Aim:** Compare RSFAE vs. supragenicular bypass grafting<br><br>**Study type:** RCT<br><br>**Size:** n=116 pts (77 [66%] had IC)	**Inclusion criteria:** TASC C and D lesions of the SFA<br><br>**Exclusion criteria:**<br>• Previous treatment (endovascular intervention or bypass)<br>• Chronic renal insufficiency (serum creatinine 1.5 mg/dL)<br>• Occlusion of iliac, common femoral, and popliteal arteries (P2-3 segments)	**Intervention:** RSFAE<br><br>**Comparator:** Supragenicular bypass	**1° endpoint:** Primary patency after 1 y follow-up was 61% for RSFAE and 73% for bypass (p=0.094). Secondary patency was 79% for both groups. Subdividing between venous (n=25) and prosthetic grafts (n=30) shows a primary patency of 89% and 63% respectively at 1 y follow-up (p=0.086).	N/A

© American Heart Association, Inc. and American College of Cardiology Foundation

Ricco JB and Probst H 2008(276) 17997269	**Aim**: Compare crossover vs. direct bypass for unilateral iliac occlusive disease in claudicants<br><br>**Study type**: RCT (multicenter)<br><br>**Size**: n=143 pts	**Inclusion criteria**: Unilateral iliac artery occlusive disease and disabling claudication<br><br>**Exclusion criteria**: N/A	**Intervention**: Crossover bypass (N=74)<br><br>**Comparator**: Direct bypass (N=69)	**1° endpoint**: Primary patency and assisted primary patency Primary patency at 5 y was higher in the direct bypass group than in the crossover bypass group (92.7 vs. 73.2, p=0.001). Assisted primary patency and secondary patency at 5 y were also higher after direct bypass than crossover bypass (92.7 vs. 84.3, p=0.04 and 97.0 vs. 89.8, p=0.03, respectively). Patency at 5 y after crossover bypass was significantly higher in pts presenting no or low-grade SFA stenosis than in pts presenting high-grade (>50%) stenosis or occlusion of the SFA (74.0% vs. 62.5%, p=0.04). In both treatment groups, patency was comparable using PTFE and polyester grafts. Overall survival was 59.5±12% at 10 y.	N/A
Jensen LP, et al. 2007(277) 17400486	**Aim**: Compare PTFE and polyester grafts for femoral to above-knee popliteal artery bypass<br><br>**Study type**: RCT (multi-center), Scandinavia<br><br>**Size**: n=427 pts (270 [65%] had IC)	**Inclusion criteria**:<br>• Consecutive pts with chronic lower limb ischemia<br>• Considered suitable for surgical revascularization using a supragenicular prosthetic bypass graft<br>• Provided the pts consented to take part<br><br>**Exclusion criteria**:<br>• Age <18 y<br>• Pregnant<br>• Previously enrolled in the study<br>• Considered impossible to follow<br>• Informed consent could not be obtained.	**Intervention**: 6 mm Dacron conduit<br><br>**Comparator**: 6 mm PTFE conduit	**1° endpoint**: 2 y primary patency rates for Dacron and PTFE were 70% and 57% (p=0.02), whereas the secondary patency rates were 76% and 65% (p=0.04), respectively. Primary patency at 2 y was significantly influenced by the number of patent crural vessels (2 or 3 67%, 1 50%, p=0.01). At 2 y, pts treated for CLI had a major amputation more often than pts operated on for IC, 10 and 3 respectively (p=0.003), and had higher mortality rates, 20% and 8% respectively (p=0.001).	• Medical therapy was not standardized<br>• Amputations at 2 y, (major in 4% and minor in 3%), 30 d mortality and complications (wound infections: 3% and other wound complications: 13%) occurred equally frequent in both groups.

© American Heart Association, Inc. and American College of Cardiology Foundation

AbuRahma AF, et al.<br>1999(278)<br>10520903	**Aim:** Compare patency of PTFE vs. saphenous vein grafts for above-knee bypass<br><br>**Study type:** Prospective, randomized<br><br>**Size:** n=43 pts (86 legs)	**Inclusion criteria:**<br>• Bilateral disabling claudication<br>• Failed medical therapy<br>• Long SFA occlusion with above-knee reconstitution.<br><br>**Exclusion criteria:** None mentioned	**Intervention:** Pts received above-knee PTFE graft in 1 leg and saphenous vein graft in the other; were randomized in terms of the order of staged interventions (either SV-PTFE or PTFE-SV)<br><br>**Comparator:** Contralateral leg in same pts; each pt served as their own control	**1° endpoint:** No statistically significant differences between primary and secondary patency rates for both grafts; however, the assisted primary patency rates were higher for SVG ($p<0.05$).	Standardized antiplatelet therapy (ASA 325 mg), but no mention of other components of medical therapy.<br>All PTFE were 8 mm grafts.
Green RM, et al.<br>2000(279)<br>10709052	**Aim:** Identify factors affecting patency of prosthetic above-knee femoropopliteal bypass grafts<br><br>**Study type:** RCT<br><br>**Size:** n=240 pts (59% had claudication)	**Inclusion criteria:**<br>• An angiographically demonstrated superficial femoral artery occlusion with reconstitution of a popliteal segment above the knee<br>• Not undergone any earlier infrainguinal vascular procedures.<br><br>**Exclusion criteria:** Adjunctive inflow procedures were not allowed at the time of the femoropopliteal bypass grafting procedure (previous aortofemoral, iliofemoral, or femoral-femoral bypass grafts were eligible, however).	**Intervention:** Above-knee femoral-popliteal bypass<br><br>**Comparator:** Gore-tex vs. Hemashield grafts	**1° endpoint:** No difference in primary or secondary patency rates at 5 yrs between the 2 grafts.	Primary patency 45% vs. 43%.<br>Secondary patency 68% vs. 68%.<br>Risk of graft occlusion increased for pts age <65 d (HR: 2.1; $p=0.001$) and for grafts with diameters <7mm (HR: 1.65; $p=0.0219$).
Johnson WC and Lee KK<br>1999(280)<br>10587392	**Aim:** To identify whether improved patency exists with different bypass graft materials for pts with femoral-popliteal above-knee bypass grafts.<br><br>**Study type:** RCT	**Inclusion criteria:** Pts scheduled for femoral-AK popliteal bypass grafting at 20 VA Medical Centers<br><br>**Exclusion criteria:**<br>• Noncompressible vessels<br>• ABI >0.9<br>• Prior ipsilateral prosthetic fem-pop AK or below-knee bypass graft	**Intervention:** above-knee femoral-popliteal bypass graft.<br><br>**Comparator:** externally supported PTFE (n=265), HUV (n=261), or SV (n =	**1° endpoint:** The cumulative assisted primary patency rates were similar among the different conduit types at 2 yrs (SV: 81%; HUV: 70%; PTFE: 69%). After 5 y, above-knee SV bypass grafts had a significantly (*p*≤0.01) better patency rate (73%) than HUV bypass grafts (53%), which had a	Possible bias against HUV and PTFE- pts with prior SV graft in ipsilateral leg were not excluded, but instead had randomization limited to either HUV or PTFE.

© American Heart Association, Inc. and American College of Cardiology Foundation

	**Size**: n=752 pts	emergency surgery<br>• <1 y life expectancy<br>• Oral anticoagulation,<br>• Popliteal aneurysmal disease<br>• Serum creatinine >2.0 mg/dL<br>• Polycythemia (red blood cell count higher than 7.5 × 106/mm^3)<br>• Platelet count >106/mm^2<br>• Prior ipsilateral SV bypass graft were not excluded, but randomization was limited to either HUV or PTFE	226)	significantly (p≤0.01) better patency rate than PTFE bypass grafts (39%).	
Klinkert P, et al. 2003(281)<br>12514593	**Aim:** To compare vein with polytetrafluoroethylene for femoropopliteal bypasses with the distal anastomosis above the knee<br><br>**Study type**: RCT<br><br>**Size**: n=151 bypasses (120 for claudication)	**Inclusion criteria**: Femoropopliteal bypass with the distal anastomosis to the popliteal artery above the knee<br><br>**Exclusion criteria**: Earlier arterial bypass graft procedure in the same leg or with the greater saphenous vein removed earlier.	**Intervention:** Femoral-AK popliteal bypass<br><br>**Comparator:** Venous vs. PTFE graft conduit	**1° endpoint:** Primary patency rates after 5 yrs were 75.6% for venous bypass grafts and 51.9% for PTFE grafts (p=0.035). Secondary patency rates were 79.7% for vein and 57.2% for PTFE bypasses (p=0 .036).	Reversed vein was used in 75 bypass grafts, and 6 mm stretched polytetrafluoroethylene prostheses were used 76 times.
Veith FJ, et al. 1986(282)<br>3510323	**Aim:** Compare patency of PTFE vs. saphenous vein for infra-inguinal arterial reconstructions<br><br>**Study type:** prospective, randomized, multicenter<br><br>**Size:** n=845 bypasses. <20% of pts had claudication.	**Inclusion criteria:** Bypass to the popliteal or an infrapopliteal artery to control ischemia caused by atherosclerosis<br><br>**Exclusion criteria:**<br>• Bypass for non-PAD diagnosis<br>• Ability to treat with endovascular approach or through deep femoral revascularizastion without bypass<br>• Sequential bypasses<br>• Composite grafts<br>• Inadequate vein	**Intervention:** PTFE<br><br>**Comparator:** Autogenus saphenous vein graft	**1° endpoint:**<br>• Patency and limb salvage by distal anastomotic site.<br>• No difference in 4 y patency for above-knee grafts. No difference in rates of limb salvage for CLI.<br>• 4 y primary patency for infrapopliteal bypasses were inferior for PTFE (49% vs. 12%, p<0.001).	Inadequate vein defined based on diameter <3.0 mm for graft to tibial artery or <4.0mm for graft to popliteal artery.

ABF indicates aortobifemoral bypass; ABI, ankle-brachial index; AK, above knee; BASI, bioabsorbable stent; CFA, common femoral artery; CFE, common femoral endarterectomy; CI indicates confidence interval; CFA, common femoral artery; CFE, common femoral artery endarectomy; CLI, critical limb ischemia; EIA-external iliac artery; ENDO, endovascular interventions; ePTFE, expanded polytetrafluoroethylene; HR, hazard ratio; HUV, human umbilical vein; IC, intermittent claudication; MWD, maximum walking distance; N/A, not applicable; OR, odds ratio; PTA, percutaneous transluminal angioplasty; PTAS, percutaneous transluminal angioplasty stent; PTFE, polytetrafluoroethylene; pt, patient; RCT, randomized controlled

© American Heart Association, Inc. and American College of Cardiology Foundation

trail; RE, remote endarterectomy; R/PTAS, percutaneous transluminal angioplasty, and stenting; RR-relative risk; RSFAE, remote superficial artery endarterectomy; SA-RIEA, stent assisted remote iliac endarterectomy; SFA, superficial femoral artery; SIA, subintimal angioplasty; SV, saphenous vein; TASC, transatlantic inter-society consensus; and TL, target lesion.

Evidence Table 38. Nonrandomized Trials, Observational Studies, and/or Registries of Surgical Treatment for Claudication–Section 8.1.2.

Study Acronym; Author; Year Published	Study Type/Design; Study Size	Patient Population	Primary Endpoint and Results (include P value; OR or RR; & 95% CI)	Summary/Conclusion Comment(s)
Nguyen BN, et al. 2015(283) 25702917	**Study type**: NR<br><br>**Size**: 1,843 procedures	**Inclusion criteria**: Common femoral endarterectomies in NSQIP database<br><br>**Exclusion criteria**: Other major procedures, hybrid procedures	**1° endpoint**: Operative mortality<br><br>**Results**: 3.4% mortality; mortality predictors included age, nonindependent functional status, preoperative dialysis, sepsis, emergency status, and ASA class 4 or 5	• Not claudication-specific
Lo RC, et al. 2014 24080134 (284)	**Study type**: NR<br><br>**Size**: n=1,797,885 pts	**Inclusion criteria**: Pts admitted with IC identified through NIS dataset based on ICD-9 primary and secondary Dx codes<br><br>**Exclusion criteria**: N/A	**1° endpoint**: In-hospital mortality stratified by gender<br><br>**Results**:<br>• Mortality lowest among pts undergoing endovascular procedures and highest among those undergoing open+endo procedures.<br>• Women had higher mortality rates than men for all procedures (open: 1.0% vs. .7%; OR: 1.37; 95% CI; 1.25–1.49; p<0.01; endovascular: 0.5% vs. 0.2%; OR; 1.99; 95% CI: 1.72–2.30; p<0.01; open+endo: 1.8% vs. .8%; OR: 2.13; 95% CI: 1.76–2.58; p<0.01).	• Claudication pts were a subgroup analysis, but reference provides claudication-specific mortality rates stratified by procedure type<br>• Hypothesis and models based on gender<br>• In-hospital mortality highest among pts who had hybrid (open+endo) procedures<br>• In-hospital mortality lowest among pts undergoing endovascular procedures
Siracuse JJ, et al. 2014(285) 24142958	**Study type**: NR<br><br>**Size**: n=1,513 pts from the ACS-NSQIP dataset (no stratification by IC/CLI/other)	**Inclusion criteria**: Elective CFE<br><br>**Exclusion criteria**: N/A	**1° endpoint**: 30 d mortality<br><br>**Results**: Partial- and total-dependent functional status (OR: 9.0; 95% CI: 2.8–28.4 and OR: 21.3; 95% CI: 3.3–139.4) and dyspnea at rest (OR: 8.2; 95% CI: 1.2–58.8) predicted mortality	• No claudication-specific results or ABI data<br>• Major morbidity (aggregate): Independent predictors of morbidity include steroid use (OR: 2.4; 95% CI: 1.4–4.1), DM (OR: 1.8; 95% CI: 1.3–2.4), and obesity (OR: 1.6; 95% CI: 1.1–2.4).<br>• Postoperative morbidities included cardiac (1.0%), pulmonary (1.9%), renal (0.4%), urinary tract infection (1.7%), thromboembolic (0.5%), neurologic (0.4%), sepsis (2.7%), superficial (6.3%), and deep surgical site complications (2.0%).<br>• At least 1 complication, including major and minor, was seen in 7.9% of the pts.
Aihara H, et al.	**Study type**: NR,	**Inclusion criteria**:	**1° endpoint**: Primary patency	• Overall complication rate was 14.4% in the

© American Heart Association, Inc. and American College of Cardiology Foundation

2014(286) 24292129	pooled data registry analysis (Japan)<br><br>**Size**: n=263 pts (313 limbs); endovascular: 177 pts (202 limbs); bypass: 86 pts (111 limbs)	Endovascular therapy or bypass surgery for claudication and TASC C/D femoropopliteal disease<br><br>**Exclusion criteria**:<br>• Hybrid procedures<br>• Acute ischemia<br>• CLI<br>• TASC A/B	**Results**: 1 and 5 y primary patency rates 82.1% and 69.4% in the bypass group and 67.8% and 45.2% in the endovascular treatment group (p<0.01, log-rank test)	bypass surgery group and 3.5% in the EVT group (p<0.01)
Boufi M, et al. 2013(287) 23835109	**Study type**: NR retrospective (France)<br><br>**Size**: n=150 limbs (82 bypass, 58 SIA/stent)	**Inclusion criteria**: Claudicants with femoropopliteal disease treated with above-knee femoropopliteal bypass or SIA + stenting<br><br>**Exclusion criteria**: N/A	**1° endpoint**: Patency<br><br>**Results**: 24 mo, primary, primary-assisted, and secondary patency for bypass vs. SIA+stent groups was, respectively, 66.6% vs. 70.1%; 76.5% vs. 90.1%; and 88.2% vs. 90.1%.	• No statistical test provided for patency difference between treatments
Sachwani GR, et al. 2013(288) 23177535	**Study type**: NR retrospective<br><br>**Size**: n=229 pts (66% of ABF and 71% of percutaneous iliac stent group were claudicants)	**Inclusion criteria**: Sx iliac artery occlusive disease undergoing iliac stenting or aortofemoral bypass<br><br>**Exclusion criteria**: N/A	**1° endpoint**:<br>• Patency<br>• Survival<br><br>**Results**: At 72 mo, the primary patency for ABF bypass was greater than for PCIS (91% vs. 73%; p=0.010). Secondary patency rates were equivalent in both groups (98% ABF vs. 85% PCIS). Survival in the ABF bypass group was significantly greater than in the PCIS group (76% vs. 68%; p=0.013).	• Includes pts with CLI<br>• Pts in the ABF grafting group were younger (age 60±0.9 y vs. age 65±1.2 y; p=0.002) and more commonly had a Hx of nicotine abuse (97% vs. 86%; p=0.002), COPD (85% vs. 70%; p=0.02), and a greater incidence of superficial femoral artery disease (45% vs. 24%; p=0.001).<br>• "Iliac stenting has lower morbidity, shorter hospital length of stay, and equivalent secondary patency but inferior primary patency compared with ABF."
Jones WS, et al. 2013(289) 23844447	**Study type**: Systematic review (AHRQ)<br><br>**Size**: n=83 studies contributed evidence; 35 were claudication specific, while 12 evaluated mixed cohorts of CLI and	**Inclusion criteria**: PubMed, Embase, and the Cochrane Database of Systematic Reviews for relevant English language studies published since January 1995<br><br>**Exclusion criteria**: N/A	**1° endpoint**: N/A<br><br>**Results**: For claudication, data were too sparse to definitively conclude which treatment is most effective. QoL showed significant improvement from cilostazol, exercise training, endovascular intervention, and surgical intervention compared with usual care. The potential additive effects of combined treatment strategies and the timing of these combined strategies are unknown.	Surgery is effective for claudication, but limited comparative evidence to support it over other treatments.

© American Heart Association, Inc. and American College of Cardiology Foundation

	claudication.			
Antoniou GA, et al. 2013(290) 23159476	**Study type**: Meta-analysis<br><br>**Size**: n=4 RCT and 6 observational studies (2,817 pts; 139=87 open, 1430 endovascular). 1 study was claudication only, while 4 included pts with either claudication or CLI.	**Inclusion criteria**: Studies comparing open surgical and percutaneous transluminal methods for the treatment of femoropopliteal arterial disease<br><br>**Exclusion criteria**: N/A	**1° endpoint**: N/A<br><br>**Results**:<br>• Endovascular treatment had lower 30 d morbidity (OR: 2.93; 95% CI: 1.34–6.41) and higher technical failure (OR: 0.10; 95% CI: 0.05–0.22) than bypass surgery, whereas no differences in 30 d mortality between the 2 groups were identified (OR: 0.92; 95% CI: 0.55–1.51).<br>• Higher primary patency in the surgical treatment arm was found at 1 (OR: 2.42; 95% CI: 1.37–4.28), 2 (OR: 2.03; 95% CI: 1.20–3.45), and 3 (OR: 1.48; 95% CI: 1.12–1.97) y of intervention.<br>• Progression to amputation was found to occur more commonly in the endovascular group at the end of the second (OR: 0.60; 95% CI: 0.42–0.86) and third (OR; 0.55; 95% CI: 0.39–0.77) y of intervention.<br>• Higher amputation free and overall survival rates were found in the bypass group at 4 y (OR: 1.31; 95% CI: 1.07–1.61 and OR: 1.29; 95% CI: 1.04–1.61, respectively).	High level evidence demonstrating the superiority of one method over the other is lacking. An endovascular first approach may be advisable in pts with significant comorbidity, whereas for fit pts with a longer term perspective a bypass procedure may be offered as a first line interventional treatment.
Malgor RD, et al. 2012(291) 22944568	**Study type**: NR retrospective, single center<br><br>**Size**: n=230 pts/262 procedures	**Inclusion criteria**: Consecutive CFE<br><br>**Exclusion criteria**:<br>• Hx of infrainguinal revascularization, including aorto-,axillo-, or iliofemoral bypass<br>• Cross-femoral bypass<br>• Common femoral interposition grafting	**1° endpoint**: Mortality, patency, reintervention, and limb salvage; analysis stratified by use of CFE alone (Group A) vs. CFE+distal revascularization (Group B)<br><br>**Results**:<br>• Cumulative 5 y primary patencies for groups A and group B were 96% and 92%, respectively.<br>• Secondary patency was 100% at both time points. Limb salvage was also lower in pts with RC 5 and 6 (p=0.01; p=0.02).<br>• Overall survival was 93% at 1 y and 77% at 5 y. There was no difference in survival between the 2 groups.	• Predictors for distal revascularization were RC 5 or 6 (p<0.001), TASC D lesions (p<0.0001), DM (p=0.04), and being on anticoagulation (p=0.003).<br>• 113 (67%) of group A and 37/85 (40%) of group B pts were claudicants
Simons JP, et al. 2012(292) 22608039	**Study type**: NR multicenter registry (Vascular Study Group of New England)	**Inclusion criteria**: Elective and urgent infrainguinal LEB for an indication of CLI (defined as tissue loss or ischemic rest pain) or IC	**1° endpoint**: Amputation-free survival<br><br>**Results**: Pts with IC experienced a lower rate of major amputation at 1 y than pts with CLI (2% vs. 12%; p<0.0001)	• Graft patency was also significantly better in the IC group when compared to the CLI group (IC: primary 79%, primary-assisted 87%, secondary 89%; CLI: primary 66%, primary-assisted 75%, secondary 77%)

© American Heart Association, Inc. and American College of Cardiology Foundation

	**Size:** n=2,907 pts (797 [28%] had IC)	**Exclusion criteria:**<br>• ALI<br>• Bypass for aneurysmal disease<br>• No specified indication		
Siracuse JJ, et al. 2012(293) [22301210](22301210)	**Study type:** NR (single center retrospective)<br><br>**Size:** n=218 pts (113 bypass, 105 PTAS)	**Inclusion criteria:** All LEB procedures at single center for claudication<br><br>**Exclusion criteria:**<br>• Limb salvage procedure<br>• Secondary procedures	**1° endpoint:**<br>• Complications,<br>• Restenosis<br>• Symptom recurrence<br>• Reinterventions<br>• Major amputation<br>• Mortality<br><br>**Results:**<br>• Bypass showed improved freedom from restenosis (73% vs. 42% at 3 y; HR: 0.4; 95% CI: 0.23–0.71), symptom recurrence (70% and 36% at 3 y; HR: 0.37; 95% CI: 0.2–0.56), and freedom from symptoms at last follow-up (83% vs. 49%; HR: 0.18; 95% CI: 0.08–0.40).<br>• Multivariable analysis of all pts showed that restenosis was predicted by PTA/S (HR: 2.5; 95% CI: 1.4–4.4) and TASC D (HR: 3.7; 95% CI: 3.5–9) lesions.<br>• Recurrence of symptoms was similarly predicted by PTA/S (HR: 3.0; 95% CI: 1.8–5) and TASC D lesions (HR: 3.1; 95% CI: 1.4–7).	• Claudication-specific retrospective study<br>• Bypass grafts were used less for TASC<br>• A (17% vs. 40%; p<0.01) and more for TASC C (36% vs. 11%; p<0.01) and TASC D (13% vs. 3%; p<0.01) lesions.<br>• There was no difference in freedom from reintervention (77% vs. 66% at 3 y; NS)<br>• Statin use postoperatively was predictive of patency (HR: 0.6; 95% CI: 0.35–0.97) and freedom from recurrent symptoms (HR: 0.6; 95% CI: 0.36–0.93).<br>• No differences in perioperative mortality (2% vs. 0%; NS) or 3 y mortality (9% vs. 8%; NS).
Kakkos SK, et al. 2011(294) [21865062](21865062)	**Study type:** NR (single center retrospective)<br><br>**Size:** n=269 pts (86 [32%] for IC)	**Inclusion criteria:** AFB<br><br>**Exclusion criteria:** N/A	**1° endpoint:** Long-term survival, complications<br><br>**Results:** 60% survival at 10 y (vs. 42% for pts with Dx other than IC; p=0.013)	• IC associated with improved long-term survival vs. CLI or aneurysm Dx, but not significant in multivariable model<br>• No other results were stratified by Dx
Simó G, et al. 2011(295) [21704539](21704539)	**Study type:** NR (single center retrospective)<br><br>**Size:** n=155 procedures (79	**Inclusion criteria:** SA-RIEA<br><br>**Exclusion criteria:** N/A<br>• Long chronic CIA occlusion<br>• stenotic aorta and/or	**1° endpoint:** Patency<br><br>**Results:** The 1, 3, and 5 y primary, primary-assisted and secondary patency rates were 80.2%, 74.7% and 69.3%; 84.8%,82.4% and 78.2%; and 86.8%, 84.2% and 79.6%,	• 10 pts required conversion to a conventional iliofemoral reconstructive procedure

© American Heart Association, Inc. and American College of Cardiology Foundation

	[51%] had IC as indication)	aneurysmal degeneration<br>• Heavily calcified EIAs or bilateral lesions	respectively	
Eugster T, et al. 2011(296)<br>21850598	**Study type**: NR (single center retrospective)<br><br>**Size**: n=124 pts	**Inclusion criteria**: Pts operated on for severe IC (walking distance\200 m) ≥y ago after failing nonoperative management<br><br>**Exclusion criteria**: N/A	**1° endpoint:**<br>• Survival<br>• Primary patency rate<br>• Assisted primary patency rate<br><br>**Results**:<br>• In-hospital and 30 d mortality of 0.8%; survival rate was 50.3% (SE±5.42%)<br>• Primary patency rate at 10 y was 63.5% (SE±7.50%)<br>• Assisted-primary patency rate was 87.3% (SE±5.19%)<br>• Patency rates of spliced and nonspliced vein bypasses were not different	• In-hospital and 30 d mortality of 0.8%
Sachs T, et al. 2011 (268)<br>21880457	**Study type**: NR (NIS database 1997–2009)<br><br>**Size**: n=264,231 pts (claudication subgroup)	**Inclusion criteria**: Pts with ICD-9 defined Dx atherosclerotic disease who underwent intervention of angioplasty stent, peripheral bypass) or aortofemoral bypass<br><br>**Exclusion criteria**: N/A	**1° endpoint**: Demographics, costs, and comorbidities, as well as multivariable adjusted in-hospital mortality and major amputation.<br><br>**Results**:<br>• In-hospital mortality was similar for PTA and BPG groups for claudication (0.1% vs. 0.2%; p=0.04)<br>• Average cost per procedure of PTA was higher than BPG for claudication ($13,903 vs. $12,681; p=0.02).<br>• Number of pts per y undergoing PTA for IC increased threefold (15,903 to 46,138)	N/A
Piazza M, et al. 2011(297)<br>21531527	**Study type**: NR (single center retrospective)<br><br>**Size**: n=162 pts (248 limbs) 74% of open repair and 60% of hybrid repair pts were claudicants	**Inclusion criteria**: Hybrid repair (combining iliac stenting and open CFE) or open aortoiliac and femoral reconstruction in pts with extensive iliac and common femoral occlusive disease<br><br>**Exclusion criteria**:<br>• Aortic thrombosis<br>• Abdominal aortic or iliac aneurysms	**1° endpoint**:<br>• 30 d mortality and morbidity<br>• ABI increase<br>• Long-term patency<br>• Procedurally related limb salvage<br>• Overall survival<br><br>**Results**:<br>• 30 d morbidity (3% vs. 5%, p=0.55) and mortality (1.1% vs. 1.4%, p=0.85) were equivalent between hybrid and open repair.	• "Procedurally related" limb salvage is likely biased endpoint<br>• Reported 100% limb salvage rate is atypical<br>• Multiple selective sub-group tests without<br>• Multiple stratified comparisons by dichotomized TASC classification

© American Heart Association, Inc. and American College of Cardiology Foundation

		• Concomitant visceral artery revascularization<br>• ALI<br>• Pts <40 y with traumatic etiology for their disease from high performance sport (competitive cyclists).	• Primary patency of hybrid vs. open repair at 3 y was similar (91% vs. 97%; p=0.29) and was maintained after stratification by TASC A/B (89% vs. 100%; p=0.38) and TASC C/D (95% vs. 97%; p=0.54).<br>• Multivariate analysis for patency indicated that major tissue loss (Rutherford class 6) at presentation in the hybrid group was predictive of decreased long-term patency (p=0.02).<br>• Limb salvage at 3 y was 100% in both groups.<br>• Overall survival was 74% for OR vs. 40% for HR (p=0.007).	
Derksen WJ, et al. 2010(298)<br>20167515	**Study type**: NR (prospective cohort)<br><br>**Size**: n=90 pts (72 [80%] had IC)	**Inclusion criteria**: RSFAE performed TASC C/D SFA obstruction with or without an additional open CFE<br><br>**Exclusion criteria**: N/A	**1° endpoint**: Restenosis following RSFAE<br><br>**Results**:<br>• 57 pts (63%), a restenotic lesion was diagnosed within 12 mo.<br>• In multivariate analysis, age, duration of ischemic walking complaints, and lumen diameter before RSFAE were associated with increased restenosis	• Complicated inclusion/exclusion criteria make generalization challenging
Koscielny A, et al. 2010(299)<br>20101647	**Study type**: NR (retrospective case-control)<br><br>**Size**: n=48 pts (24 matched pairs)	**Inclusion criteria**: Pts with peripheral arterial occlusive disease undergoing femoropopliteal supragenicular bypass or profundaplasty<br><br>**Exclusion criteria**: None mentioned	**1° endpoint**:<br>• Bypass occlusion<br>• Surgical revision<br>• Amputation<br>• Death<br><br>**Results**: No significant outcome differences between supragenicular bypass surgery orprofundaplasty in pts who had surgery for IC	• Mean length of follow-up was 36 mo
Ballotta E, et al. 2010(300)<br>19828166	**Study type**: NR (retrospective single center cohort)(Italy)<br><br>**Size**: n=117 pts (121 procedures [60% of procedures were for claudication])	**Inclusion criteria**:<br>• CFA occlusive disease (isolated or with additional infrainguinal lesions in the ipsilateral limb)<br>• Amenable to endarterectomy of the CFA (isolated or combined with a profundoplasty or with the endarterectomy of the superficial or deep femoral artery first tract, not >1 cm long)	**1° endpoint**: Patency<br><br>**Results**:<br>• 7 y PP, APP, and LS rates were 96%, 100%, and 100%, respectively<br>• The 7 y rates of freedom from further revascularization and survival were 79% and 80%, respectively.	• No comparison group

© American Heart Association, Inc. and American College of Cardiology Foundation

		**Exclusion criteria**: Major tissue loss for which a contemporary infrainguinal revascularization was performed		
Burke CR, et al. 2010(301) 20122461	**Study type**: NR (retrospective single center)<br><br>**Size**: n=118 AFB and 174 aortoiliac angioplasty and AS procedures	**Inclusion criteria**: All pts undergoing treatment AIOD at the University of Michigan Hospitals between 1997–2007<br><br>**Exclusion criteria**: None mentioned	**1° endpoint**:<br>• Mortality<br>• Adverse events<br><br>**Results**:<br>• Long-term mortality, freedom from amputation, and freedom from revision procedure of any type (endovascular or open) were not different between groups.<br>• AFB was associated with increased surgical complication rates including the need for emergency surgery (6.8% and 1.7%; p=0.029), infection/sepsis (16.1% and 2.3%; p<0.001), transfusion (16.1% and 5.7%; p=0.004), and lymph leak (8.5% and 0.6%; p=0.001).<br>• No difference between AFB and AS groups with respect to 30 d mortality (0.8% and 1.1%; p=0.64), MI (1.7% and 1.1%; p=0.53), cerebrovascular accident (0.0% and 1.1%; p=0.35), or renal failure requiring hemodialysis (3.4% and 1.2%; p=0.19).	• Large number of statistical comparisons without adjustment of significance level<br>• Not claudication specific (60 % of PTA and 41% of AFB pts had IC)
Twine CP and McLain AD 2010(302) 20464717	**Study type**: Cochrane systematic review<br><br>**Size**: n=13 RCT with 2,313 pts (1955 above knee, 358 below knee bypasses)	**Inclusion criteria**: Randomized trials comparing femoro-popliteal grafts.<br><br>**Exclusion criteria**: N/A	**1° endpoint**: N/A<br><br>**Results**: 7 graft types were compared (reversed and in situ autologous vein, PTFE with and without vein cuff, HUV, Dacron and HBD. Above the knee, there was a benefit in primary patency for autologous vein over PTFE (p=0.0001) and HUV (p=0.0003) by 60 mo. Dacron showed primary patency benefit over PTFE by 24 mo (p=0.02), continuing to 60 mo (p=0.02). HUV also showed benefit over PTFE by 24 mo (p=0.0003) in 1 trial. Below the knee, in the 1 trial there was a significant benefit in primary patency for PTFE with a vein cuff when compared to PTFE alone at all time intervals to 24 mo (p=0.03). Limited data were available for limb survival. Antiplatelet and anticoagulant protocols varied extensively between	There was a clear primary patency benefit for autologous vein when compared to synthetic materials for above knee bypasses. In the long term (5 y) Dacron confers a small primary patency benefit over PTFE for above knee bypass. PTFE with a vein cuff improved primary patency when compared to PTFE alone for below knee bypasses. Further randomized data is needed to ascertain whether this information translates into improvement in limb survival.

© American Heart Association, Inc. and American College of Cardiology Foundation

			trials, and in some cases within trials.	
Chiesa R, et al. 2009(303) [19540713](19540713)	**Study type**: NR (retrospective single center cohort)<br><br>**Size**: n=822 pts (777 [94%] had claudication as indication)	**Inclusion criteria**: Consecutive pts undergoing aortoiliac or aortofemoral reconstruction employing a bifurcated ePTFE stretch graft<br><br>**Exclusion criteria**:	**1° endpoint**:<br>• Survival<br>• Graft-patency survival<br>• Amputation-free survival<br><br>**Results**:<br>• 11 y primary graft-patency rate 90.6%<br>• The secondary rate patency rate was 97.9%	• Amputation-free survival only evaluated in subset of pts with CLI as indication<br>• Primary patency reported was for total 11 y duration of study period but mean follow-up of only 72 mo<br>• No survival analysis; descriptive analysis without models accounting time considerations
Al-Khoury G, et al. 2009(304) [19628359](19628359)	**Study type**: NR (retrospective single center cohort)<br><br>**Size**: n=95 pts (105 limbs); 65% of procedures done for IC	**Inclusion criteria**: Pts who underwent an isolated femoral endarterectomy<br><br>**Exclusion criteria**: N/A	**1° endpoint**:<br>• Change in ABI (based on cut-point of 15)<br>• Change in Rutherford class<br>• Repeat intervention<br>• Patency<br><br>**Results**:<br>• 83.8% of pts with marked initial clinical improvement remained symptom free at 2 y, whereas only 28.6% in the group with mild and moderate initial response maintained their clinical status.<br>• 2 y freedom from repeat intervention was 61.8%.<br>• Multivariate analysis revealed that TASC C/ D lesions (OR: 9.3; 95% CI: 2.43–35.63; p=0.001) and DM (OR: 3.64; 95% CI: 1.01–13.15; p=0.048) were predictive of recurrent symptoms while extensive endarterectomy and ≥2 vessel tibial runoff decreased the need for repeat intervention.<br>• Patency was 100% with a mean follow-up of 11 mo (1–72).<br>• Complete resolution of symptoms was noted in 73.4% with some clinical improvement noted in 91% of limbs.<br>• ABI increase achieved in 85.1% with a mean ABI increase of 0.27±0.20, and this correlated with ≥2 runoff vessels (OR: 0.20; 95% CI: 0.04–0.96; p=0.04).	N/A
Goodney PP, et al. 2009(305) [19497502](19497502)	**Study type**: NR (prospective registry) (Vascular	**Inclusion criteria**: LEB for arterial occlusive disease	**1° endpoint**: Predictors of ambulation status 1 y postoperatively	

© American Heart Association, Inc. and American College of Cardiology Foundation

	Study Group of New England)<br><br>**Size**: n=1,400 pts, 1561 bypasses (IC was indication for 25%)	**Exclusion criteria**: N/A	**Results**:<br>• Claudicant pts had higher primary (79% vs. 73%; p<0.001) and secondary (87% vs. 81%; p<0.001) graft patency rates and were more likely to be alive and ambulatory 1 y postoperatively (96% vs. 81%; p<0.001) than CLI pts.<br>• Amputation rates were 12% for CLI pts and 1% for claudicant pts (p<0.001).<br>• All claudicant pts walked before surgery, and the 95% who survived 1 y postoperatively remained ambulatory.<br>• The risk of dying or being nonambulatory 1 y postoperatively was increased in pts who were nonambulatory preoperatively (HR: 1.5; 95% CI: 1.3–1.6; p<0.0001), by increasing age of 70–79 y (HR: 1.8; 95% CI: 1.2–2.6; p<0.007) and 80-89 y (HR: 2.3; 95% CI: 1.5–3.7; p<0.0001), by CLI (HR: 2.0; 95% CI: 1.2–3.4; p<0.007), by postoperative MI (HR: 2.5; 95% CI: 1.6–4.1; p<0.001), and by major amputation (HR: 2.9; 95% CI: 2.1–4.1; p<0.001).<br>• Graft thrombosis during follow-up (HR: 1.6; 95% CI: 1.1–1.8; p<0.003) and living in a nursing home preoperatively (HR: 3.5; 95% CI: 1.5–7.8; p<0.003) were independently associated with a higher risk of being nonambulatory at 1 y.	
Chang RW, et al. 2008(306)<br>18572359	**Study type**: NR (single center retrospective cohort)<br><br>**Size**: n=171 pts, 193 procedures (46% had claudication as indication)	**Inclusion criteria**: CFE with patch angioplasty and primary stenting or stent grafting in a single combined hybrid open and endovascular procedure for treatment of TASC C and iliofemoral occlusive disease<br><br>**Exclusion criteria**: N/A	**1° endpoint**: Technical success, clinical success (based on AHA classification), ABI change, patency, adverse events, length of stay<br><br>**Results**:<br>• 30 d mortality was 2.3% and 5 y survival was 60%.<br>• 5 y primary, primary-assisted, and secondary patencies were 60%, 97%, and 98% respectively.<br>• Endovascular reintervention was required in 14% of pts; inflow surgical procedures were required in 10%.<br>• By logistic regression analysis, use of stent grafts compared with bare stents was associated with significantly higher primary patency (87% 5% vs. 53% 7%; p<0.01).<br>• Clinical improvement was seen in 92% of pts.	N/A

© American Heart Association, Inc. and American College of Cardiology Foundation

			• Mean ABI increased from 0.38 0.32 to 0.72 0.24.<br>• Median length of stay was 2 d (range, 1–51 d).	
KoivunenK and Lukkarinen H 2008(265)<br>18221916	**Study type:** NR, prospective<br><br>**Size:** n=180 pts (64 conservative, 85 endovascular, 31 surgery)	**Inclusion criteria:** IC (Fontaine II), surgery clinic pt at university hospital in Finland<br><br>**Exclusion criteria:** Nonatherosclerotic disease, lack of angiographic verification of Dx, previous surgery/endovascular treatment <5 y, CLI	**1° endpoint:** HRQoL (Nottingham Health Profile)<br><br>**Results:**<br>• Conservative group's clinical outcomes (ABI, asx walking distance) remained stable, while these measures improved significantly in the surgery group<br>• Conservative group had improved quality of sleep and emotional reactions<br>• Endo group had significant improvement in emotional reactions and energy + reduction in social isolation. No significant changes in pain or mobility<br>• Surgery group had improvements in sleep, pain, emotional reactions, social isolation, and physical mobility<br>• Large effect size for surgery vs. small for conservative, endo	• Pts treated with conservative approach exercised more often at baseline<br>• Surgery group had more baseline hypertension<br>• Smoking increased significantly in conservative management group
Jaquinandi V, et al. 2007(307)<br>17264010	**Study type:** NR, prospective<br><br>**Size:** n=105 pts	**Inclusion criteria:**<br>• Age ≥18 y<br>• Had a patent AFB for ≥4 mo before his or her visit<br>• Able to walk on treadmill<br><br>**Exclusion criteria:**<br>• Acute CLI<br>• Unstable angina pectoris<br>• Uncontrolled hypertension<br>• New York Heart Association (NYHA) cardiac insufficiency function class of III or IV<br>• MI ≤3 mo<br>• Arterial aneurysm or pseudoaneurysm<br>• Major respiratory limitation (resting dyspnea)<br>• Stroke or major neurologic disorders<br>• Lived too far from the	**1° endpoint:** Symptoms based on modified San Diego Claudication questionnaire, change in $TcPO_2$ before and after treadmill ambulation<br><br>**Results:** 30 pts reported proximal exercise-related pain consistent with vascular criteria by Hx before exercise. However, 59 pts (56%) reported symptoms compatible with proximal claudication, and $TcPO_2$ values were abnormal on one or both sides in 52. The persistence of at least one (prograde or retrograde) pathway to the hypogastric circulation did not decrease proportion of pts reporting proximal claudication by Hx (26%) or on treadmill (55%) compared with those with bilateral hypogastric occlusion (33% by Hx; p=0.51 compared with at least one prograde hypogastric pathway and 61% based on treadmill test, p=0.65 compared with at least one prograde hypogastric pathway).	N/A

© American Heart Association, Inc. and American College of Cardiology Foundation

		laboratory.		
Fowkes F and Leng GC<br>2008(308)<br>18425879	**Study type:** Systematic review (Cochrane)<br><br>**Size:** n=19 trials (2 claudication only, 4 with claudication and CLI)	**Inclusion criteria:** RCTs of bypass surgery for chronic lower limb ischemia vs. any other treatment<br><br>**Exclusion criteria:** N/A	**1° endpoint:** N/A<br><br>**Results:** Mortality and amputation rates did not differ significantly between bypass surgery and PTA; primary patency was significantly higher in the bypass group after 12 mo (OR: 1.6; 95% CI: 1.0–2.6) but not after 4 y (p=0.14). Blood flow restoration was significantly greater in bypass than in thromboendarterectomy pts (Peto OR: 9.2; 95% CI: 1.7–50.6); mortality and amputation rates did not differ. Bypass surgery outcomes did not differ significantly from exercise or spinal cord stimulation.	There is limited evidence for the effectiveness of bypass surgery compared with other treatments; no studies compared bypass to no treatment. Further large trials are required.
Periera CE, et al.<br>2006(309)<br>16950427	**Study type:** Meta-analysis<br><br>**Size:** n=73 articles included; analysis included claudication-specific subgroup	**Inclusion criteria:** graft patency included as outcome, follow up of 1 y for at least some grafts, minimum of 30 bypasses in at least 1 series when article descrbied 2 or more series, and publication after 1986<br><br>**Exclusion criteria:**<br>• Clinical symptoms not described<br>• Predominance of blind segments of popliteal artery<br>• Predominance of composite bypass grafts<br>• Predominance of bypasses to the infrapopliteal arteries<br>• Repeat inclusion of bypasses<br>• Unreliable or unattainable reconstruction of life tables from graphs or texts.	**1° endpoint:** Pooled primary graft patency<br><br>**Results:** For claudication-specific meta-analysis, pooled primary graft patency was 57.4% for above-knee polytetrafluoroethylene, 77.2% for above-knee vein, and 64.8% for below-knee vein at 5 y; there was a significant difference between above-knee grafts at 3, 4, and 5 y (p<0.05). The corresponding pooled secondary graft patency was 73.2%, 80.1%, and 79.7%, respectively (p>0.05).	The great saphenous vein performs better than polytetrafluoroethylene in femoropopliteal bypass grafting and should be used whenever possible.
Rosenthal D, et al.<br>2006(310)<br>16953157	**Study type:** NR (retrospective multicenter cohort)<br><br>**Size:** n=210 pts (158 [75%] were	**Inclusion criteria:** Remote superficial femoral endarterectomy and distal aSpire stenting for TASC D SFA lesion	**1° endpoint:** Primary cumulative patency<br><br>**Results:**<br>• Primary cumulative patency rate by means of life-table analysis was 60.6±4.8% (SE) at 33 mo, (mean 17.1 mo; range 133 mo).	• Did not stratify results by diagnostic indication<br>• 12 pts (5.7%) had wound complications

© American Heart Association, Inc. and American College of Cardiology Foundation

	claudicants)	**Exclusion criteria**: N/A	• During follow-up percutaneous transluminal balloon and/or stent angioplasty was necessary in 50 pts for a primary assisted patency of 70.2±4.8% at 33 mo.<br>• Mean ABI rose from 0.58–0.95	
Martin JD, et al. 2006(311) 16476609	**Study type**: NR (retrospective single center cohort)<br><br>**Size**: n=133 pts (57% had IC)	**Inclusion criteria**: Remote endarterectomy from an inguinal incision for vascular reconstruction of >10 cm length total occlusions of the external iliac and/or superficial femoral arteries.<br><br>**Exclusion criteria**: N/A	**1° endpoint**: Primary patency<br><br>**Results**: Mean follow-up was 19 mo, with a primary patency of 70% at 30 mo by life-table analysis. Limb salvage was 94%.	• 12% technical failure rate (bypass performed in these pts)
Mori E, et al. 2002(312) 11821823	**Study type**: NR (prospective, observational)<br><br>**Size**: n=427 pts [surgery=259 (362 legs) conservative=168]	**Inclusion criteria**: Admitted to the hospital for IC<br><br>**Exclusion criteria**: N/A	**1° endpoint**:<br><br>**Results**:<br>• Surgery group had significantly better QOL improvement than conservative<br>• Infrainguinal and conservative were not significantly different	• Inferior 3 and 5 y patency observed for below knee bypass<br>• Recommendation for surgical revascularization may be overinterpretation of results<br>• No defined pharmacotherapy<br>• No exercise comparator<br>• Does not report adverse events, amputation rates
Feinglass J, et al 2000(263) 10642712	**Study type**: NR (prospective, observational)<br><br>**Size**: n=526 pts (104 had revascularization, including 60 bypasses and 44 angioplasties)	**Inclusion criteria**: Abnormal ABI without prior LE revascularization or CLI symptoms<br><br>**Exclusion criteria**:<br>• Prior revascularization<br>• Rest pain<br>• Ulcers<br>• Gangrene	**1° endpoint**: SF-36 physical functioning score<br><br>**Results**:<br>• Bypass and angioplasty groups maintained highly significant improvements in mean physical function and walking distance scores, and reported greater leg symptom improvement<br>• Conditions of unmatched medical management pts declined on all outcome measures<br>• Mean ABI improved significantly for bypass, modestly for angioplasty	• Pts who underwent angioplasty and surgery were classified as surgical bypass (regardless if procedures were staged within a single admission or separate hospitalizations)<br>• Does not include adverse event rates<br>• No standardized medical management<br>• No mention of exercise therapy
Pell JP and Lee AJ 1997(266) 9507581	**Study type**: NR (prospective, observational)<br><br>**Size**: n=201 pts	**Inclusion criteria**: newly referred pts with IC<br><br>**Exclusion criteria**: N/A	**1° endpoint**: QoL (SF-36)<br><br>**Results**:<br>• All aspects of QoL deteriorated following conservative treatment<br>PTA and reconstruction had significant improvement in pain and physical function after adjustment for case	• F/U data available on 81% of 195 pts alive at final timepoint.<br>◦10% had PTA<br>◦10% had reconstruction<br>◦76% managed conservatively<br>• "Conservative management" was not defined beyond lack of procedural intervention

© American Heart Association, Inc. and American College of Cardiology Foundation

			mix	• No defined pharmacotherapy<br>• No exercise therapy comparison group
Archie JP Jr<br>1994(313)<br>7811585	**Study type:** NR (retrospective, single center)<br><br>**Size:** n=312 bypasses in 285 pts (39% had IC as indication)	**Inclusion criteria:** Femoropopliteal bypass using ipsilateral autologous reversed GSV when available and PTFE when not.<br><br>**Exclusion criteria:** N/A	**1° endpoint:** Patency<br><br>**Results:** GSV patency superior to PTFE at 3 and 5 yr; P<0.01.	• Patency for GSV vs. PTFE was 87% vs. 54% at 3 yr and 81% vs. 48% at 5 ys.<br>• Above-knee GSV primary patency >below-knee GSV >above-knee PTFE.<br>• Overall PTFE failure rate was 3–4 times higher than that of GSV.
Hunink MG, et al.<br>1994(314)<br>8152359	**Study type:** NR (meta-analysis)<br><br>**Size:** n=17 femoral-popliteal bypass studies were included in life table analysis of patency	**Inclusion criteria:** English language articles had to report original data, patency based on life table or Kaplan-Meier analysis with the number at risk or standard errors, define patency as hemodynamic improvement, report the distribution of covariates, and not duplicate other published material.<br><br>**Exclusion criteria:** See above	**1° endpoint:** Patency<br><br>**Results:** Unadjusted pooled 5 yr patency was 45% for angioplasty, 73% for bypass surgery using a vein graft, and 49% for bypass surgery using PTFE graft. Adjusted 5 yr primary patencies after surgery varied from 33%–80% with the best results being for saphenous vein bypass performed for claudication.	Pooled data included bypasses performed for CLI/limb salvage as well as claudication, but analysis was stratified based on indication.
Schweiger H, et al.<br>1993(315)<br>8230575	**Study type:** NR (retrospective single center)<br><br>**Size:** n=211 grafts in 184 pts, 195 legs (none had IC)	**Inclusion criteria:** Below-popliteal (tibial) PTFE grafts implanted for limb salvage<br><br>**Exclusion criteria:** N/A	**1° endpoint:** 5 yr cumulative limb salvage<br><br>**Results:** 5 yr cumulative limb salvage was 51%	• 2 yr primary/secondary patency 37% / 45%<br>• 5 yr primary/secondary patency 23% / 25%<br>• Primary bypass procedures had superior outcomes vs. secondary<br>• All pts had CLI<br>• 25 limbs had acute ischemia
Baldwin ZK, et al.<br>2004(316)<br>15111843	**Study type:** Retrospective single center<br><br>**Size:** n=631 infrainguinal bypass grafts in 578 legs; 85% were for CLI.	**Inclusion criteria:** N/A<br><br>**Exclusion criteria:** N/A	**1° endpoint:** Limb salvage<br><br>**Results:** Limb salvage rates following graft failure were 50% at 2 yr. Limb salvage was 100% among pts with IC as initial bypass indication. Early graft failure (<30 d) had worse prognosis.	"The overall prognosis for limb salvage in pts with failed infrainguinal bypass grafts is poor, particularly in pts with grafts placed because of tissue loss and those with early graft failure."
Leng GC, et al.<br>1996(317)<br>9027521	**Study type:** Prospective cohort study (Edinburgh Artery Study)<br><br>**Size:** n=1,592 pts	**Inclusion criteria:** Age 55–74 y selected randomly from the age-sex registers of 10 general practices in Edinburgh, Scotland<br><br>**Exclusion criteria:** N/A	**1° endpoint:** Incidence and natural hx of claudication; incidence of CV events in sx and asx PAD.<br><br>**Results:**116 new cases of claudication identified (incidence of 15.5 per 1,000 person-years)	Among those with baseline claudication, 28.8% still had pain after 5 yr, 8.2% underwent vascular surgery or amputation, and 1.4% developed leg ulcers.
Kannel WB et al.	**Study type:** NR	**Inclusion criteria:** General	**1° endpoint:** Incidence of claudication by age and sex	5,209 pts at the initial examination; of these 4,030

© American Heart Association, Inc. and American College of Cardiology Foundation

1970(318)<br>5444530	(prospective cohort)<br><br>**Size:** n=5,209 pts	population of adult men and women (Framingham; 14 y follow up)<br><br>**Exclusion criteria:** None stated	**Results:** 79 men and 46 women developed claudication. Overall annual incidence per 10,000 was 26 for men and 12 for women. No death was attributable to impaired limb circulation, and no amputation related to circulatory diseased occurred over 14 yr study period.	returned for the 8 examination covered in this analysis.
Kannel WB and Shurtleff D<br>1971(319)<br>5119838	**Study type:** NR (prospective cohort)<br><br>**Size:** n=5,209 pts	**Inclusion criteria:** General population of adult men and women (Framingham; 16 y follow up)<br><br>**Exclusion criteria:** None stated	**1° endpoint:** Adverse cardiovascular events, mortality<br><br>**Results:** No death in the study group was directly attributable to impaired leg circulation. A total of 6 amputations occurred. Among those followed for ≥4 y from onset of claudication symptoms, 45% had their symptoms disappear for at least 4 y	• Purpose of study was "to examine in a general population the manner in which IC arises, evolves, and becomes complicated by more serious cardiovascular impairments, and terminates fatally".<br>• Significant overlap with Kannel 1970 (making it challenging to identify distinct findings within this report).
Tillgren C<br>1965(320)<br>14317326	**Study type:** NR (retrospective)<br><br>**Size:** n=466 pts	**Inclusion criteria:** Pts treated at hospitals in Stockholm for complaints in the lower limbs causing a suspicion of arterial insufficiency<br><br>**Exclusion criteria:** Embolic ALI, peripheral arterial insufficiency that appeared in the final stage of a severe disease (e.g., heart failure or cancer).	**1° endpoint:** Survival, amputation, adverse CV events.<br><br>**Results:** 36/294 (1.5%) of pts whose symptoms were attributed to arteriosclerosis had an amputation during the observation period. Amputation rate among this subgroup was 2.24/1000 mo for men and 1.23/1000 mo for women.	• Study included pts suspected to have Beurger's disease.<br>• Classified pts with DM separate from those with atherosclerosis.<br>• Included pts with CLI but did not stratify results in a similar fashion.<br>• Authors concluded that "the course of the disease in the lower limbs does not affect life expectancy to any considerable extent."
Jelnes R, et al.<br>1986(321)<br>3094806	**Study type:** NR<br><br>**Size:** n=257 pts	**Inclusion criteria:** Pts referred consecutively for the first time for claudication during a 1 y period.<br><br>**Exclusion criteria:** Rest pain, ulcers, or foot gangrene.	**1° endpoint:** Rate of clinical progression (to rest pain or gangrene).<br><br>**Results:** 7.5% rate of progression in the worst affected leg during first yr after referral; 2.2% per yr thereafter.	• Unclear whether design was prospective or retrospective.<br>• Recruitment occurred from the department of clinical physiology at a single hospital over 1 y.<br>• At a mean follow up of 6.5 ± 0.5 yts, 44% of pts had died.
Bloor K<br>1961(322)<br>19310276	**Study type:** Topic overview<br><br>**Size:** N/A	**Inclusion criteria:** N/A<br><br>**Exclusion criteria:** N/A	**1° endpoint:** N/A<br><br>**Results:** N/A	N/A
Dormandy J, et al.<br>1989 (323)<br>2647761	**Study type:** NR (Review)<br><br>**Size:** n=52 studies published between 1958–1986	**Inclusion criteria:** English language published data<br><br>**Exclusion criteria:** Publications based on small numbers of pts or inconclusive data	**1° endpoint:** Fate of pts presenting with chronic leg ischemia<br><br>**Results:** Reported prevalence of claudication in general population ranges from 0.4%–6.9% in men and 0.2%–3% in women. 25% of pts with claudication had worsening of symptoms after presentation, and 1.5–5% had major amputation.	N/A

© American Heart Association, Inc. and American College of Cardiology Foundation

ABF indicates aortobifemoral; ABI, ankle-brachial index; ALI, acute limb ischemia; ACS NSQIP, American College of Surgeons National Surgical Quality Improvement Program; AFB, aortobifemoral bypass; AHRQ, Agency for Healthcare Research and Quality; AIOD, aortoiliac occlusive disease; APP, assisted primary patency; AS, aortoiliac stenting; ASA, American Society of Anesthesiologist; BPG, bypass graft; CFA, common femoral artery; CFE, common femoral endarterectomy; CIA, common iliac artery; CI, confidence interval; CLI, critical limb ischemia; COPD, chronic obstructive pulmonary disease; CV, cardiovascular; EIA, external iliac artery; ePTFE, expanded polytetrafluoroethylene; EVT, endovascular treatment; GSV, greater saphenous vein; HBD, heparain bonded Dacron; HR, hazard ratio; HRQoL, heath-related quality of life; HUV, human umbilical vein; ICD, International Classification of Disease; IC, intermittent claudication; LEB, lower extremity bypass; LE, lower extremity; LS, limb salvage; N/A, not applicable; NIS, National Impatient Sample; NR, nonrandomized; NSQIP, National Surgical Quality Improvement Program, NS, not significant; NYHA, New York Heart Association; OR, odds ratio; PAD, peripheral artery disease; PCIS, percutaneous iliac stent; PP, primary patency; PTAS, percutaneous angioplasty/stent; PTFE, polytetrafluoroethylene; pt, patient; QoL, quality of life; RC, routine care; RCT, randomized controlled trial; RR, relative risk; RSFAE, remote superficial artery endarterectomy; SA RIEA, Stent-assisted remote iliac endarterectomy; SE, supervised exercise; SFA, superficial femoral artery; SIA, subintimal angioplasty; TASC, transatlantic inter-society consensus; and $TcPO_2$, transcutaneous oxygen pressure.

Evidence Table 39. RCTs Comparing Endovascular Revascularization for Chronic CLI–Section 8.2.

Study Acronym; Author; Year Published	Aim of Study; Study Type; Study Size (N)	Patient Population	Study Intervention (# patients) / Study Comparator (# patients)	Endpoint Results (Absolute Event Rates, P value; OR or RR; & 95% CI)	Relevant 2° Endpoint (if any); Study Limitations; Adverse Events
Werk M, et al. 2012(232)<br>23192918	**Aim**: SFA DCB vs. PTA<br><br>**Study type**: RCT<br><br>**Size**: **n=**85 pts	**Inclusion criteria**: Sx femoro-popliteal atherosclerotic disease<br><br>**Exclusion criteria**:<br>• Acute thrombus or aneurysm in the target vessel<br>• Failure to cross the target lesion with a guidewire<br>• Inflow lesions that cannot be successfully pretreated<br>• Significant disease of all 3 infrapopliteal vessels<br>• Renal failure (serum creatinine >2.0 mg/dL)<br>• Known intolerance or allergy to study medication<br>• Life expectancy <2 y	**Intervention**: DCB<br><br>**Comparator**: PTA	**1° endpoint**: The primary endpoint was late lumen loss at 6 mo assessed by blinded angiographic corelab quantitative analyses	• DEB is superior to PTA<br>• Pts with sx femoro-popliteal atherosclerotic disease undergoing percutaneous transluminal angioplasty were randomized to paclitaxel-coated IN.PACT Pacific or uncoated Pacific balloons. The primary endpoint was late lumen loss at 6 mo assessed by blinded angiographic corelab quantitative analyses. Secondary endpoints were binary restenosis and Rutherford class change at 6 mo, and target lesion revascularization + major adverse clinical events (major adverse events=death, target limb amputation, or target lesion revascularization) at 6 and 12 mo. 85 pts (91 cases=interventional procedures) were randomized in 3 hospitals (44 to DEB and 47 to uncoated balloons). Average lesion length was 7.0±5.3 and 6.6±5.5 cm for DEB and control arm, respectively. Procedural success was obtained in all cases. 6 mo quantitative angiography showed that DEB were associated with significantly lower late lumen loss (-0.01 mm; 95% CI: -0.29–0.26 vs. 0.65 mm; 95% CI: 0.37–0.93; p=0.001) and fewer binary restenoses (3 [8.6%] vs. 11 [32.4%]; p=0.01). This translated into a clinically

© American Heart Association, Inc. and American College of Cardiology Foundation

					relevant benefit with significantly fewer major adverse events for DEB vs. uncoated balloons up to 12 mo (3 [7.1%] vs. 15 [34.9%]; p<0.01) as well as target lesion revascularizations (3 [7.1%] vs. 12 [27.9%]; p=0.02).
**IN.PACT**<br>Tepe G, et al.<br>2015(229)<br>25472980	**Aim**: SFA DCB vs. PTA<br><br>**Study type**: RCT<br><br>**Size**: n=331 pts	**Inclusion criteria**: IC or ischemic rest pain attributable to superficial femoral and popliteal PAD<br><br>**Exclusion criteria**:<br>• Lesion and/or occlusions located in or extending to the popliteal artery or below the ankle joint space<br>• Inflow lesion or occlusion in the ipsilateral iliac, SFA, or popliteal arteries with length ≥15 cm<br>• Significant (≥50% DS) inflow lesion or occlusion in the ipsilateral iliac, SFA, or popliteal arteries left untreated<br>• Previously implanted stent in the TL(s)<br>• Aneurysm in the target vessel. Acute thrombus in the TL	**Intervention**: DCB<br><br>**Comparator**: PTA	**1° endpoint**: 12 mo primary patency	• DCB superior to PTA<br>• The IN.PACT SFA Trial is a prospective, multicenter, single-blinded, randomized trial in which 331 pts with IC or ischemic rest pain attributable to superficial femoral and popliteal PAD were randomly assigned in a 2:1 ratio to treatment with DCB or PTA. The primary efficacy endpoint was primary patency, defined as freedom from restenosis or clinically driven target lesion revascularization at 12 mo. Baseline characteristics were similar between the 2 groups. Mean lesion length and the percentage of total occlusions for the DCB and PTA arms were 8.94±4.89 and 8.81±5.12 cm (p=0.82) and 25.8% and 19.5% (p=0.22), respectively. DCB resulted in higher primary patency vs. PTA (82.2% vs. 52.4%; p<0.001). The rate of clinically driven target lesion revascularization was 2.4% in the DCB arm in comparison with 20.6% in the PTA arm (p<0.001). There was a low rate of vessel thrombosis in both arms (1.4% after DCB and 3.7% after PTA [p=0.10]). There were no device- or procedure-related deaths and no major amputations
**ABSOLUTE**<br>Schillinger M, et al.<br>2007(225)<br>17502568	**Aim:** SFA PTAS vs. PTA<br><br>**Study type:** RCT<br><br>**Size**: n=104 pts	**Inclusion criteria**: Rutherford 3–5 and SFA stenosis<br><br>**Exclusion criteria**:<br>•ALI<br>• Previous bypass surgery, or stenting of the SFA<br>• Untreated inflow disease of the ipsilateral pelvic arteries (>50% stenosis or occlusions)	**Intervention**: PTAS<br><br>**Comparator**: PTA	**1° endpoint:** Restenosis by duplex at 2 y	• PTAS is superior to PTA for long lesions (lesion length 112 mm PTAS and 93 mm PTA)<br>• Of 104 pts with chronic limb ischemia and SFA obstructions, 98 (94%) could be followed up until 2 y after intervention for occurrence of restenosis (>50%) by duplex ultrasound and for clinical and hemodynamic outcome by treadmill walking distance and ABI. Restenosis rates at 2 y were 45.7% (21 of 46) vs. 69.2% (36 of 52) in favor of primary stenting compared with balloon angioplasty with optional secondary stenting by an ITT analysis (p=0.031). Consistently, stenting (whether primary or secondary; n=63) was superior to plain balloon angioplasty (n=35) with respect to the occurrence of restenosis (49.2% vs. 74.3%; p=0.028) by a treatment-received analysis. Clinically, pts in the primary stent group showed a trend toward better

© American Heart Association, Inc. and American College of Cardiology Foundation

					treadmill walking capacity (average, 302 vs. 196 m; p=0.12) and better ABI values (average, 0.88 vs. 0.78; p=0.09) at 2 y, respectively. Reintervention rates tended to be lower after primary stenting (17 of 46 [37.0%] vs. 28 of 52 [53.8%]; p=0.14)
**FAST**<br>Krankenberg H, et al.<br>2007(226)<br>17592075	**Aim**: SFA PTA vs. PTAS<br><br>**Study type**: RCT<br><br>**Size**: n=244 pts	**Inclusion criteria**: SFA stenosis & claudication or CLI<br><br>**Exclusion criteria**: Major exclusion criteria were:<br>• A TL that required pretreatment with adjunctive devices such as lasers or debulking catheters<br>• A TL that extended into the popliteal artery<br>• Previous stent implantation in the targeted SFA<br>• Multiple lesions exceeding a total length of 10 cm<br>• Acute or subacute (≤4 wk) thrombotic occlusion<br>• Untreated ipsilateral iliac artery stenosis<br>• Ongoing dialysis treatment<br>• Treatment with oral anticoagulants other than antiplatelet agents.	**Intervention**: PTAS<br><br>**Comparator**: PTA	**1° endpoint**: Technical success, 1 y duplex restenosis	• For short lesions mean length 45mm, no difference between PTAS and PTA<br>• Overall, stent fractures were detected in 45 of 121 treated legs (37.2%). In a stent-based analysis, 64 of 261 stents (24.5%) showed fractures, which were classified as minor (single strut fracture) in 31 cases (48.4%), moderate (fracture of >1 strut) in 17 cases (26.6%), and severe (complete separation of stent segments) in 16 cases (25.0%). Fracture rates were 13.2% for stented length ≤8 cm, 42.4% for stented length >8–16 cm, and 52.0% for stented length >16 cm. In 21 cases (32.8%) there was a restenosis of >50% diameter reduction at the site of stent fracture. In 22 cases (34.4%) with stent fracture there was a total stent reocclusion. According to Kaplan-Meier estimates, the primary patency rate at 12 mo was significantly lower for pts with stent fractures (41.1% vs. 84.3%, p<0.0001).
Gandini R, et al.<br>2013(324)<br>24325697	**Aim**: CLI & SFA ISR: DCB vs. laser+DCB<br><br>**Study type**: RCT<br><br>**Size**: n=448 pts	**Inclusion criteria**: CLI and chronic SFA in-stent occlusion<br><br>**Exclusion criteria**: Denovo stenosis without ISR	**Intervention**: Laser+DCB<br><br>**Comparator**: DCB	**1° endpoint**: 12 mo primary patency	• Laser+DEB superior to DEB alone<br>• In the Laser+DEB group, the patency rates at 6 and 12 mo (91.7% and 66.7%, respectively) were significantly higher (p=0.01) than in the DEB only pts (58.3% and 37.5%, respectively). TLR at 12 mo was 16.7% in the Laser+DEB group and 50% in the DEB only group (p=0.01). 2 (8%) pts needed major amputations in the Laser+DEB group vs. 11 (46%) in the DEB only group at 12 mo (p=0.003).
**DEBATE-SFA**<br>Liistro F, et al.<br>2013(230)<br>24239203	**Aim**: PEB+BMS vs. PTA+BMS<br><br>**Study type**: RCT	**Inclusion criteria**: Claudication or CLI and SFA stenosis<br><br>**Exclusion criteria**:<br>• Life expectancy <1 y	**Intervention**: PEB+BMS<br><br>**Comparator**: PTA+BMS	**1° endpoint**: 12 mo binary restenosis	• PEB+BMS is superior to PTA+BMS<br>• Mean lesion length was 94±60 vs. 96±69 mm in the PEB+BMS and PTA+BMS groups (p=0.8), respectively. The primary endpoint occurred in 9 (17%) vs. 26 (47.3%) of lesions in the PEB+BMS and PTA+BMS groups

© American Heart Association, Inc. and American College of Cardiology Foundation

	**Size**: n=104 pts	• Contraindication for combined antiplatelet therapy<br>• Known allergy to nickel or paclitaxel<br>• Need for major amputation at the time of enrollment<br>• Failure to recanalize intended below-the-knee arteries in CLI pts at risk of major amputation was also considered an exclusion criterion			(p=0.008), respectively. A near-significant (p=0.07) 1-y freedom from target lesion revascularization advantage was observed in the PEB+BMS group. No major amputation occurred. No significant difference was observed according to lesion characteristics or technical approach.
**IN.PACT DEEP**<br>Zeller T, et al.<br>2014 (325)<br>25301459	**Aim**: Infrapop: DCB vs. PTA<br><br>**Study type**: RCT<br><br>**Size**: n=358 pts	**Inclusion criteria**: CLI due to infrapop PAD<br><br>**Exclusion criteria**:<br>• Lesion and/or occlusions located in or extending to the popliteal artery or below the ankle joint space<br>• Inflow lesion or occlusion in the ipsilateral iliac, SFA, or popliteal arteries with length ≥15 cm<br>• Significant (≥50% DS) inflow lesion or occlusion in the ipsilateral iliac, SFA, or popliteal arteries left untreated<br>• Failure to obtain <30% residual stenosis in pre-existing, hemodynamically significant (≥50% DS and <15 cm length) inflow lesions in the ipsilateral iliac, SFA, or popliteal artery<br>• DES and/or DEB was not allowed for the treatment of inflow lesions GFR <30 mL/min except for pts with renal end-stage disease on chronic hemodialysis	**Intervention**: DCB<br><br>**Comparator**: PTA	**1° endpoint**: Clinically driven target lesion revascularization (CD-TLR) and late lumen loss (LLL).<br><br>**Safety endpoint**: The primary safety endpoint through 6 mo was a composite of all-cause mortality, major amputation, and CD-TLR.	• Increased amputation with DEB<br>• Clinical characteristics were similar between the 2 groups. Significant baseline differences between the IA-DEB and PTA arms included mean lesion length (10.2 cm vs. 12.9 cm; p=0.002), impaired inflow (40.7% vs. 28.8%; p=0.035), and previous target limb revascularization (32.2% vs. 21.8%; p=0.047). Primary efficacy results of IA-DEB vs. PTA were CD-TLR of 9.2% vs. 13.1% (p=0.291) and LLL of 0.61±0.78 mm vs. 0.62±0.78 mm (p=0.950). Primary safety endpoints were 17.7% vs. 15.8% (p=0.021) and met the noninferiority hypothesis. A safety signal driven by major amputations through 12 mo was observed in the IA-DEB arm vs. the PTA arm (8.8% vs. 3.6%; p=0.080).
**ACHILLES**<br>Scheinert D, et al.<br>2012(326)<br>23194941	**Aim**: Infrapop: DES vs. PTA<br><br>**Study type**: RCT<br><br>**Size**: n=200 pts	**Inclusion criteria**: CLI due to infrapop PAD<br><br>**Exclusion criteria**:<br>• Significant stenoses (>50%) distal to the TL that might require revascularization or impede runoff<br>• Angiographically evident thrombus or Hx of thrombolysis within 72 h	**Intervention**: DES<br><br>**Comparator**: PTA	**1° endpoint**: 1 y angiographic restenosis vessel patency death, repeat revascularization, index-limb amputation rates	• Infrapop DES superior to PTA for CLI<br>• 99 and 101 pts (mean age 73.4 y; 64% DM) were randomized to SES and PTA, respectively (8 crossover bailout cases to SES). At 1 y, there were lower angiographic restenosis rates (22.4% vs. 41.9%, p=0.019), greater vessel patency (75.0% vs. 57.1%, p=0.025), and similar death, repeat revascularization, index-limb amputation rates, and proportions of pts with improved Rutherford class for SES vs. PTA.

© American Heart Association, Inc. and American College of Cardiology Foundation

		• Untreated lesions (>75% stenosis) in the common or external iliac<br>• Common or superficial femoral and popliteal artery<br>• Infrapopliteal trifurcation lesions requiring 2- or 3-branch treatment<br>• Stent placement across or within 1 cm of the knee joint or in an artery subject to external compression<br>• Prior stenting within the target vessel(s) or aneurysm in the SFA or popliteal artery<br>• Hx of thrombophlebitis, deep venous thrombosis, or impaired renal function (Cr >2.5 mg/dl)<br>• Life expectancy <12 mo<br>• Known intolerance to antiplatelet medication.			
**ACHILLES**<br>Katsanos K, et al.<br>2016(327)<br>26777329	**Aim**: Infrapop: DES vs. PTA<br><br>**Study type**: RCT<br><br>**Size**: n=200 pts	**Inclusion criteria**: Refer to ACHILLES trial above<br><br>**Exclusion criteria**:<br>• Refer to ACHILLES trial above	**Intervention**: DES<br><br>**Comparator**: PTA	**1° endpoint**: 1 y angiographic restenosis vessel patency death, repeat revascularization, index-limb amputation rates	• Infrapop SES axcellerates wound healing and is ES superior to PTA for CLI<br>• There was a trend of more QALYs gained with SES compared with PTA up to 1 y after randomization. Relative QALY gain was 0.10 (95% CI: -0.01–0.21; p=0.08) in the whole study and 0.17 (95% CI: -0.03–0.35; p=0.09) in the wound subgroups comparison.
**BASIL**<br>Adam DJ, et al.<br>2005 (328)<br>16325694	**Aim**: Bypass vs. PTA for CLI<br><br>**Study type**: RCT<br><br>**Size**: n=452 pts	**Inclusion criteria**: CLI due to infrainguinal PAD<br><br>**Exclusion criteria**: Pt who could not be treated equally well with infrainguinal bypass or angioplasty in the opinion of a vascular surgeon and interventional radiologist	**Intervention**: PTA<br><br>**Comparator**: Bypass	**1° endpoint**: Amputation free survival	• Equal outcomes<br>• The trial ran for 5.5 y, and follow-up finished when pts reached an endpoint (amputation of trial leg above the ankle or death). 7 individuals were lost to follow-up after randomization (3 assigned angioplasty, 2 surgery); of these, 3 were lost (1 angioplasty, 2 surgery) during the first y of follow-up. 195 (86%) of 228 pts assigned to bypass surgery and 216 (96%) of 224 to balloon angioplasty underwent an attempt at their allocated intervention at a median (IQR) of 6 (3–16) and 6 (2–20) d after randomization, respectively. At the end of follow-up, 248 (55%) pts were alive without amputation (of trial leg), 38 (8%) alive with amputation, 36 (8%) dead after amputation, and 130 (29%) dead without amputation. After 6 mo, the 2 strategies did not differ significantly in

© American Heart Association, Inc. and American College of Cardiology Foundation

					amputation-free survival (48 vs. 60 pts; unadjusted HR: 1.07; 95% CI: 0.72–1.6; adjusted HR: 0.73; 95% CI: 0.49–1.07). We saw no difference in health-related quality of life between the 2 strategies, but for the first y the hospital costs associated with a surgery-first strategy were about 1/3 higher than those with an angioplasty-first strategy.
**BASIL**<br>Bradbury AW, et al.<br>2010 (329)<br>20307380	**Aim**: Bypass vs. PTA for CLI<br><br>**Study type**: RCT<br><br>**Size**: n=452 pts	**Inclusion criteria**: CLI due to infrainguinal PAD<br><br>**Exclusion criteria**: Pt who could not be treated equally well with infrainguinal bypass or angioplasty in the opinion of a vascular surgeon and interventional radiologist	**Intervention**: PTA<br><br>**Comparator**: Bypass	**1° endpoint**: AFS	N/A
**BASIL**<br>Bradbury AW, et al.<br>2014 (330)<br>20435259	**Aim**: Bypass vs. angiography for CLI<br><br>**Study type**: ITT analysis of a RCT<br><br>**Size**: n=452 pts	**Inclusion criteria**: CLI due to infrainguinal PAD<br><br>**Exclusion criteria**: Pt who could not be treated equally well with infrainguinal bypass or angioplasty in the opinion of a vascular surgeon and interventional radiologist	**Intervention**: PTA<br><br>**Comparator**: Bypass	**1° endpoint**: AFS and OS	Bypass was associated with improvements in OS and AFS of about 7 and 6 mo, but long term no significant difference between the treatments
**LEVANT 1**<br>Schienert D, et al.<br>2014 (231)<br>24456716	**Aim**: Assess efficacy of DEB vs. PTA with bailout stenting<br><br>**Study type**: RCT<br><br>**Size**: DEB=49 pts;<br>Standard PTA=52 pts	**Inclusion Criteria:** Rutherford 2–5 symptoms<br><br>**Exclusion criteria:**<br>• Listed in methods<br>• Notably highly calcified lesions	**Intervention**: DEB<br><br>**Comparator**: Standard PTA with bailout stenting	**1° endpoint:**<br>• Angiography lumen loss at 6 mo<br>• At 6 mo DEB had lower lumen loss than standard PTA ($p<0.016$)	Small study

© American Heart Association, Inc. and American College of Cardiology Foundation

**DEBELLUM**<br>Fanelli F, et al.<br>2012 (331)<br>23046320	**Aim**: Assess efficacy of DEB vs. PTA<br><br>**Study type**: RCT<br><br>**Size**: DEB=25 pts; Standard PTA=25 pts	**Inclusion criteria**: Fontaine 2b-4 symptoms<br><br>**Exclusion criteria**: Pts requiring provisional stenting after angioplasty secondary to flow-limiting dissection or residual stenosis >50%	**Intervention**: DEB<br><br>**Comparator**: Standard PTA	**1° endpoint**:<br>• Angiography lumen loss at 6 mo<br>• Late lumen loss was lower in the DEB group (p<0.01)	Small study
**LEVANT-2**<br>Rosenfield K, et al.<br>2015 (332)<br>26106946	**Aim**: Assess efficiency of DEB vs. PTA with bailout stenting<br><br>**Study type**: RCT<br><br>**Size**: n=476 pts	**Inclusion criteria**: Fontaine 2–4 symptoms<br><br>**Exclusion criteria**:<br>• Lesion length ≥15 cm<br>• Detailed in NEJM	**Intervention**: DEB<br><br>**Comparator**: Standard PTA	**1° endpoint**:<br>• Primary patency of target lesion at 12 mo<br>• DEB superior (p<0.02)<br>• DEB noninferior with regard to safety endpoints	N/A
**DESTINY**<br>Bosiers M, et al.<br>2012 (333)<br>22169682	**Aim**: Assess infrapopliteal PTAS with DES vs. BMS for CLI<br><br>**Study type**: RCT<br><br>**Size**: n=140 pts	**Inclusion criteria**: CLI and infrapop stenosis<br><br>**Exclusion criteria**: Lack of ≥1 vessel outflow to the foot	**Intervention**: DES<br><br>**Comparator**: BMS	**1° endpoint**:<br>• Binary restenosis of the target lesion at 12 mo<br>• DES was superior to BMS (p=0.001)	Reduced restenosis and the need for reintervention compared with bare metal stents
Rastan A, et al.<br>2011 (334)<br>21622669	**Aim**: Determine if SES improves primary patency rates after interventional therapy of focal lesions of infrapopliteal artery<br><br>**Study type**: Prospective,	**Inclusion criteria**:<br>• Age ≥21 y<br>• PAD with Rutherford-Becker class 3–5<br>• lifestyle-limiting claudication Rutherford-Becker classs 2 if successful intervention of TASC A femoropopliteal lesions to improve runoff status<br>• Presence of a single primary target lesion in a native infrapopliteal artery that was 2.5–3.5 mm in diameter, and ≤44 mm in length	**Intervention**: Polymer-free sirolimus-eluting stent<br><br>**Comparator**: Placebo-coated bare-metal stent	**1° endpoint**:<br>• 1-y primary patency rate<br><br>**2° endpoints**:<br>• 6-mo primary patency rate<br>• Secondary patency rate<br>• Changes in Rutherford-Becker classification after 1 y	SES improved mid-term patency rates compared to BMS

© American Heart Association, Inc. and American College of Cardiology Foundation

	randomized, multi-centre, double-blind trial<br><br>**Size**: n=161 pts	• Diameter stenosis of ≥70%<br><br>**Exclusion criteria**:<br>• Pregnant pts<br>• Visible thrombus within target lesion<br>• Known systemic coagulopathy<br>• Buerger's disease<br>• ALI<br>• Life expentency <1 y<br>• Intolerance of aspirin, clopidogrel, and heparin			
Siablis D, et al. 2014 (335) 25234679	**Aim**: To compare PCB vs. DES in long infrapopliteal lesions<br><br>**Study type**: Prospective PCT<br><br>**Size**: n=50 pts	**Inclusion criteria**:<br>• Rutherford classes 3–6<br>• Angiographically documented infrapopliteal disease ≥70 mm<br><br>**Exclusion criteria**: N/A	**Intervention**: Polymer-free sirolimus-eluting stent<br><br>**Comparator**: Placebo-coated bare-metal stent	**1° endpoint**:<br>• Target lesion restenosis >50% at 6 mo<br><br>**2° endpoints**:<br>• Immediate post-procedure stenosis<br>• Target lesion revascularization	• Significant lower residual immediate post-procedure stenosis in DES compared with PCB in long infrapopliteal lesion<br>• At 6 mo, significantly reduced vessel restenosis in DES compared with PCB
Tepe G, et al. 2015 (336) 25616822	**Aim**: Evaluate 5-y follow-up of PCB on the restenosis rate after peripheral arterial interventions.<br><br>**Study type**: multicenter RCT<br><br>**Size**: n=154 pts	**Inclusion criteria**:<br>• Included in the THUNDER study<br><br>**Exclusion criteria**: N/A	**Intervention**:<br>• PCB and standard nonionic contrast medium (PCB group)<br>• Plain old balloon angioplasty and paclicaxel added to standard nionic contrast medium (paclitaxel-in-CM Group)	**1° endpoint**:<br>• Angiographic LLL (difference between the postprocedural and 6-mo follow up minimal lumen diameter, evaluated by quantitative angiography)<br><br>**2° endpoints**:<br>• freedom from TL revascularization, binary restenosis rate, and amputation	• 5-y follow up period resulted in maintained reduced TL revascularizationrate following PCB treatment. No signs of drug-related local vessel abnormalities were detected.

© American Heart Association, Inc. and American College of Cardiology Foundation

			**Comparator:** Plain old balloon angioplastic and standard nonionic CM (Control group)		

ABI indicates ankle-brachial index; AFS, amputation-free surivival; ALI, acute limb ischemia; BMS indicates bare metal stent; CD-TLR, clinically driven target lesion revascularization; CI, confidence interval; CLI, critical limb ischemia; DCB, drug coated balloon; DEB, drug eluting balloon; DES, drug eluting stent; DM, diabetes mellitus; HR, hazard ratio; IA-DEB, apmhirion-drug eluting balloon; IC, intermittent claudication; ISR, in stent restenosis; IQR, interquartile range; JACC, Journal of American College of Cardiology; LLL, late lumen loss; N/A, not applicable; OR, odds ratio; OS, overall survival; PAD, periphery artery disease; PCB, paclitaxel-coated blaoon; PEB, paclitaxel eluting balloon; PTA, percutaneous angioplasty, PTAS, percutaneous angioplasty stent; pt, patient; RCT, randomized controlled trial; RR, relative risk; SES, self-expanding stents; and SFA, superficial femoral artery; and TL, target lesion.

Evidence Table 40. Nonrandomized Trials, Observational Studies, and/or Registries of Endovascular Revascularization for Chromic CLI–Section 8.2.1.

Study Acronym; Author; Year Published	Study Type/Design; Study Size	Patient Population	Primary Endpoint and Results (include P value; OR or RR; & 95% CI)	Summary/Conclusion Comment(s)
Kashyap VS, et al. 2008 (224) 18804943	**Study type:** Retrospective Endo vs. ABF<br><br>**Size:** n=189 pts	**Inclusion criteria:** Sx AIOD (claudication, 53%; rest pain, 28%; tissue loss, 12%; ALI, 7%)<br><br>**Exclusion criteria:**<br>• Pts undergoing endovascular treatment such as PTA or stenting for iliac stenoses<br>• Pts with iliac dissection, an associated AAA, or iliac recanalization before or during AAA endograft placement.	**1° endpoint:** Technical success, primary patency at 3 y<br><br>**Results:** 3 y primary patency was higher in ABF group but population was biased	• ABF superior<br>• Selection bias<br>• The ABF pts were younger than the R/PTAS pts (60 vs. 65 y; p=0.003) and had higher rates of hyperlipidemia (p=0.009) and smoking (p<0.001). All other clinical variables, including cardiac status, DM, symptoms at presentation, TransAtlantic Inter-Society Consensus stratification, and presence of poor outflow were similar between the 2 groups. Pts underwent ABF with general anesthesia (96%), often with concomitant treatment of femoral or infrainguinal disease (61% endarterectomy, profundaplasty, or distal bypass). Technical success was universal, with marked improvement in ABI (0.48–0.84; p<0.001). Pts underwent R/PTAS with local anesthesia/sedation (78%), with a 96% technical success rate and similar hemodynamic improvement (0.36–0.82; p<0.001). At the time of R/PTAS, 21% of pts underwent femoral endarterectomy/profundaplasty or bypass (n=5) for concomitant infrainguinal disease. Limb-based primary patency at 3 y was significantly higher for ABF than for R/PTAS (93% vs. 74%, p=0.002). Secondary patency rates (97% vs. 95%), limb salvage (98% vs. 98%), and long-term

© American Heart Association, Inc. and American College of Cardiology Foundation

				survival (80% vs. 80%) were similar. DM and the requirement of distal bypass were associated with decreased patency (p<0.001). CLI at presentation (tissue loss, HR: 8.1; p<0.001), poor outflow (HR: 2; p=0.023), and renal failure (HR: 2.5; p=0.02) were associated with decreased survival.
Ferraresi R, et al. 2009 (337) 19112033	**Study type:** Case series: infrapop PTA for CLI<br><br>**Size:** n=101 pts	**Inclusion criteria:** Pts with DM with CLI due to infrapop PAD<br><br>**Exclusion criteria:** Above the knee >70% stenosis	**1° endpoint:** Limb salvage<br><br>**Results:** 93% limb salvage rate; no comparator	• Proof of concept; poor quality<br>• The limb salvage rate was 93% after a mean follow-up of 1048±525 d (2.9±1.4 y). Transcutaneous oxygen tension significantly increased after 1 mo (18.1±11.2 vs. 39.6±15.1; p<0.05). After 1 y, target-vessel re-stenosis had occurred in 42% of the non-amputated limbs, 9 pts (9%) had died because of medical conditions unrelated to PTA and 3 pts had undergone repeat PTA for recurrent CLI.
Park, SW, et al. 2013 (338) 23975668	**Study type:** Case series<br><br>**Size:** n=64 pts	**Inclusion criteria:** CLI due to CTO in below the knee artery<br><br>**Exclusion criteria:** Pts with concomitant above-knee arterial steno-occlusive lesions including the aortoiliac and femoropopliteal arterial lesions, clinical or imaging signs of embolic disease, or who had undergone thrombolysis prior to endovascular or surgical procedures.	**1° endpoint:** Limb salvage<br><br>**Results:** 90.6% limb salvage rate and 59.1% primary patency rate at 1 y. No comparator group.	• Reasonable limb salvage<br>• Poor vessel patency at 1 y<br>• The BTK EVT was performed on 64 limbs. Technical success rate was 93.8% and limb salvage rate was 90.6%. 3 of 4 limbs with technical failure and 3 of 60 limbs with technical success underwent BTK amputation and the comparison of these rates were significantly different (75% vs. 5%; p=0.002). Primary patency rates for the limbs were 75% and 59.1% at 6 mo and 12 mo follow-up, respectively. Minor complications disappeared through the follow-up periods and there was no 30 d complication or systemic adverse events for the treated vessel.
Faglia E, et al. 2006 (339) 16730466	**Study type:** Case series<br><br>**Size:** n=564 total pts: 420 PTA, 117 bypass, 27 both	**Inclusion criteria:** Pts with DM with CLI<br><br>**Exclusion criteria:**<br>• Pts without DM<br>• No stenosis >50%	**1° endpoint:** Limb salvage<br><br>**Results:** Major amputation was associated with absence of revascularization (OR: 35.9; p<0.001; 95% CI: 12.9–99.7), occlusion of each of the 3 crural arteries (OR: 8.20; p=0.022; 95% CI: 1.35–49.6), wound infection (OR: 2.1; p=0.004; 95% CI: 1.3–3.6), dialysis (OR: 4.7; p=0.001; 95% CI: 1.9–11.7) increase in $TcPO_2$ after revascularization (OR: 0.80; p<0.001; 95% CI:	• PTA was carried out in 420 (74.5%), BPG in 117 (20.7%) pts. In 27 (4.8%) pts both PTA and BPG were not possible. 23 above-the-ankle amputations (4.1%) were performed at 30 d: 6 in PTA pts, 3 in BPG pts, 14 in nonrevascularized pts. In the follow-up of 558 pts (98.9%), 62 repeated PTAs and 9 new BPGs, 32 new major amputations (16 in PTA pts, 14 in BPG pts and 2 in nonrevascularized pts) were performed. Major amputation was associated with absence of revascularization (OR: 35.9; p<0.001; 95% CI: 12.9–99.7), occlusion of each of the 3 crural arteries (OR: 8.20; p=0.022; 95% CI: 1.35–49.6), wound infection (OR: 2.1; p=0.004; 95% CI: 1.3–3.6), dialysis (OR: 4.7; p=0.001; 95% CI: 1.9–11.7) increase in $TcPO_2$ after revascularization (OR: 0.80; p<0.001; 95% CI: 0.74–0.87). 173 pts died during follow-up and this

© American Heart Association, Inc. and American College of Cardiology Foundation

			0.74–0.87).	was associated with age (HR: 1.05; p<0.001; 95% CI: 1.03–1.07), Hx of cardiac disease (HR: 2.16; p<0.001; 95% CI: 1.53–3.06), dialysis (HR: 3.52; p<0.001; 95% CI: 2.08–5.97), absence of revascularization (HR: 1.68; p<0.001; 95% CI: 1.29–2.19) and impaired ejection fraction (HR: 1.08; p<0.001; 95% CI: 1.05–1.09).
Faglia E, et al. 2005. (340) 15878541	**Study type:** Case series<br><br>**Size:** n=993 pts	**Inclusion criteria:** CLI treated with endo<br><br>**Exclusion criteria:**<br>• Pts without DM<br>• No stenosis >50%	**1° endpoint:** Limb salvage<br><br>**Results:** 1.7% major amputation rate at variable follow-up of 26±15 mo. No comparator	• PTA effective<br>• PTA was successful performed in 993 pts. 17 (1.7%) major amputations were carried out. 1 death and 33 nonfatal complications were observed. Mean follow-up was 26±15 mo. Clinical restenosis was observed in 87 pts. The 5 y primary patency was 88%, 95% CI 86-91%. During follow-up 119 (12.0%) pts died at a rate of 6.7% per y.
Iida O, et al. 2012 (341) 22051875	**Study type:** Retrospective analysis of BTK PTA: angiosome vs. non-angiosome<br><br>**Size:** n=369 limbs from 329 consecutive pts	**Inclusion criteria:** CLI treated with endo<br><br>**Exclusion criteria:** Unsuccessful recanalization of ≥1 vessel to the pedal arch	**1° endpoint:** Limb salvage<br><br>**Results:** Freedom from major amputation at 18±16 mo was higher in the angiosome directed group 51%±8% vs. 28%±8%, p=0.008	• AFS higher in angiosome directed endo group<br>• During follow-up (mean, 18±16 mo), the overall limb salvage rate was 81% (300 of 369), death occurred in 36% (119 of 329), and the reintervention rate was 31% (114 of 369). After propensity score adjustment, the estimated (± standard error) rates for AFS (49%±8% vs. 29%±6%; p=0.0002), freedom from MALE (51%±8% vs. 28%±8%, p=0.008), and major amputation (82%±5% vs. 68%±5%, p=0.01) were significantly higher in the direct group than in the indirect group for up to 4 y after the index procedure. After multivariable Cox proportional analysis, the independent factors associated with major amputation were hemoglobin A(1c) level (HR: 1.4; 95% CI: 1.1–1.9; p=0.006) and cilostazol administration (HR: 0.28; 95% CI: 0.11–0.70; p=0.006) in the direct group, and C-reactive protein level (HR: 1.2; 95% CI: 1.1–1.4; p=0.002) in the indirect group
Feiring AJ, et al. 2010 (342) 20378075	**Study type:** Case series<br><br>**Size:** n=105 pts	**Inclusion criteria:** Infrapop DES for CLI<br><br>**Exclusion criteria:**<br>• Lack of CLI<br>• No exclusions for other comorbidities	**1° endpoint:** Major amputation and mortality<br><br>**Results:** The 3 y cumulative incidence of amputation was 6±2%, survival was 71±5%, and amputation-free-survival was 68±5%	• Infrapop DES for CLI appears effective<br>• The mean pt age was 74±9 y. There were 228 DES implanted (83% Cypher [Cordis, Johnson & Johnson, Warren, New Jersey], 17% Taxus [Boston Scientific, Maple Grove, Minnesota]). The number of stents per limb was 1.9±0.9, and 35% of limbs received overlapping DES (length of 60±13 mm). There were no procedural deaths, and 96% of pts were discharged within 24 h. The 3 y cumulative incidence of amputation was 6±2%, survival was 71±5%, and amputation-free-survival was 68±5%. Only 12% of pts who died had a preceding major amputation. Rutherford category, age,

© American Heart Association, Inc. and American College of Cardiology Foundation

				creatinine level, and dialysis (p≤0.001–0.04) were predictors of death but not amputation. Target limb revascularization occurred in 15% of pts, and repeat angiography in 35% of pts revealed a binary restenosis in 12%.
Siablis D, et al. 2009 (343) [19620014](19620014)	**Study type:** Registry: Infrapop DES vs. BMS<br><br>**Size:** n=103 pts	**Inclusion criteria:** CLI treated with infrapop DES or BMS<br><br>**Exclusion criteria:**<br>• Hx of severe contrast allergy/hypersensitivity<br>• Hypersensitivity to ASA and/or clopidogrel<br>• Systemic coagulopathy or hypercoagulation disorders<br>• ALI<br>• Buerger disease<br>• Deep vein thrombosis<br>• Bifurcation and/or trifurcation lesions<br>• Previous use of other DES (not SES)<br>• Stenting indications after suboptimal and/or complicated balloon angioplasty<br>• Elastic recoil<br>Flow-limiting dissection<br>• Residual stenosis >30%	**1° endpoint:** Primary clinical and angiographic endpoints included mortality, limb salvage, primary patency, binary angiographic restenosis, and clinically driven repeat intervention-free survival.<br><br>**Results:** At 3 y, SES-treated lesions were associated with significantly better primary patency (HR: 4.81; 95% CI: 2.91–7.94; p<0.001), reduced binary restenosis (HR: 0.38; 95% CI: 0.25–0.58; p<0.001), and better repeat intervention-free survival (HR: 2.56; 95% CI: 1.30–5.00; p=0.006) vs. BMS-treated ones. No significant differences were identified between SESs and BMSs with regard to overall 3 y pt mortality (29.3% vs. 32.0%; p=0.205) and limb salvage (80.3% vs. 82.0%; p=0.507).	• Infrapop DES for CLI appears effective<br>• In total, 103 pts were included in the analysis; 41 (75.6% with DM) were treated with a BMS (47 limbs; 77 lesions) and 62 (87.1% with DM) with an SES (75 limbs; 153 lesions). At 3 y, SES-treated lesions were associated with significantly better primary patency (HR: 4.81; 95% CI: 2.91–7.94; p<0.001), reduced binary restenosis (HR: 0.38; 95% CI: 0.25–0.58; p<0.001), and better repeat intervention-free survival (HR: 2.56; 95% CI: 1.30–5.00; p=0.006) vs. BMS-treated ones. No significant differences were identified between SESs and BMSs with regard to overall 3 y pt mortality (29.3% vs. 32.0%; p=0.205) and limb salvage (80.3% vs. 82.0%; p=0.507).
Werner M, et al. 2012 (344) [22313195](22313195)	**Study type:** Case series<br><br>**Size:** n=158 pts	**Inclusion criteria:** Infrapop DES for CLI<br><br>**Exclusion criteria:** Lack of infrapop stenosis	**1° endpoint:** Angiographic binary restenosis; freedom from death, amputation, and bypass<br><br>**Results:** Results in column to the right; no comparator group	• Proof of concept for infrapop DES<br>• Technical success was achieved in all cases. The primary patency rates were 97.0% after 6 mo, 87.0% after 12 mo, and 83.8% at 60 mo. In-stent stenosis was predominantly observed in the first y after stent placement. Female gender was associated with a higher rate of ISS. During clinical follow-up of 144 (91%) pts over a mean 31.1±20.3 mo, there were 27 (18.8%) deaths, 4 (2.8%) amputations, and no bypass surgery. Clinical status improved in 92% of the pts with CLI and 77% of the pts suffering from claudication (p=0.022).
Acin F, et al. 2014 (345)	**Study type:** Retrospective case	**Inclusion criteria:** Infrapop intervention for CLI in pts with	**1° endpoint:** Ischemic ulcer healing and limb salvage rates	N/A

© American Heart Association, Inc. and American College of Cardiology Foundation

[24527215](24527215)	series assessing CLI treatment with number of infrapop vessels and angiosome relationship<br><br>**Size**: n=101 procedures; 92 pts	DM	**Results:** No difference between 1 vessel run-off and multiple vessels; no difference is single vessel was in angiosome of wound	
Alexandrescu VA, et al.<br>2008 (346)<br>18840046	**Study type:** Retrospective case series assessing CLI treatment with angiosome relationship<br><br>**Size:** n=98 pts	**Inclusion criteria:** Infrapop intervention for CLI in pts with DM	**1° endpoint:** Ischemic ulcer healing and limb salvage rates<br><br>**Results:** Limb salving and healing rates typical of that described for endo for CLI	No comparator group
Fossacaca R, et al.<br>2013 (347)<br>23358605	**Study type:** Retrospective case series assessing CLI treatment with angiosome relationship<br><br>**Size:** n=201 pts	**Inclusion criteria:** Infrapop intervention for CLI in pts with DM	**1° endpoint:** Ischemic ulcer healing and limb salvage rates at 1,6, and 12 mo<br><br>**Results:** No difference in therapeutic efficacy with indirect revasc vs. angiosome directed revasc	Higher $TcPO_2$ in angiosome group but no clinical outcome difference
Kabra A, et al.<br>2013 (348)<br>23058724	**Study type:** Prospective case series assessing CLI treatment with angiosome relationship<br><br>**Size:** n=64 pts	**Inclusion criteria:** Infrapop intervention for CLI in pts	**1° endpoint:**<br>• Ischemic ulcer healing and limb salvage rates at 1,3, and 6 mo<br>• The difference in the rates of ulcer healing between the DR and IR groups was statistically significant (p=0.021). The limb salvage in the DR group (84%) and IR group (75%) was not statistically significant (p=0.06)	Small study
Kret MR, et al.<br>2014 (349)<br>23972526	**Study type:** Retrospective case series assessing CLI treatment with angiosome relationship	**Inclusion criteria:** Infrapop intervention for CLI in pts	**1° endpoint:**<br>• Complete wound healing and time to complete wound<br>• No difference between angiosome group and indirect revasc group	N/A

© American Heart Association, Inc. and American College of Cardiology Foundation

	**Size:** n=97 pts			
Lejay A, et al. 2014 (350) 24333196	**Study type:** Retrospective case series assessing CLI treatment with angiosome relationship<br><br>**Size:** n=54 pts	**Inclusion criteria:** Infrapop bypass for CLI in pts	**1° endpoint:**<br>• Median ulcer-healing time, survival, primary patency, and limb salvage rates between angiosome vs. indirect bypass group<br>• Angiosome directed bypass had higher limb salvage at 1, 3, and 5 y (p=0.03) compared to indirect revasc	Small study
Neville RF, et al. 2009 (351) 19179041	**Study type:** Retrospective case series assessing CLI treatment with angiosome relationship<br><br>**Size:** n=48 pts	**Inclusion criteria:** Infrapop bypass for CLI in pts	**1° endpoint:**<br>• Complete wound healing and time to complete wound<br>• Angiosome group had more complete wound healing ; among wounds that did heal there was no difference in time to healing between the 2 groups	Small study
Osawa S, et al. 2013 (352) 23822940	**Study type:** Retrospective case series assessing CLI with angiosome relationship<br><br>**Size:** n=111 pts (n=57 for endo therapy)	**Inclusion criteria:** CLI	**1° endpoint:**<br>• Time to complete wound in pts who had angiosome or indirect revasc<br>• Wound healing rate was faster for angiosome directed group	Small study
Abu Dabrh AM, et al. 2015 (353) 26391460	**Aim:** To investigate natural hx of untreated CLI or severe limb ischemia<br><br>**Study type:** SR/MA of observational studies<br><br>**Size:** n=13 studies (1,527)	**Inclusion criteria:**<br>• Studies with pts. reporting rest pain, tissue loss, ulcer, or gangrene<br>• Rutherford class 4–6<br>• Or ankle pressure <70 mm Hg, toe pressure <50 mm Hg<br>• Flat pulse volume recording<br>• transcutaneous O_2 pressure <40 mmHg for ≥1 y.<br>• No revasc treatment.	**1° endpoint:** Mortality, Major amputation, wound healing<br><br>**Results:**<br>• All-cause mortality: 22% (95% CI: 12%–33%)<br>• Major amputation rate: 22% (95% CI: 2%–42%)<br>• Worsened wound or ulcer: 35% (95% CI: 10%–62%)	Trend towards improvement in the current era probably due to improved medical care

© American Heart Association, Inc. and American College of Cardiology Foundation

		**Exclusion criteria**: Revascularization treated arms		

AAA indicates abdominal aortic aneurysm; ABF, aortobifemoral bypass; ABI, ankle-brachial index; AFS, amputation free survival; AIOD, aortoiliac occlusive disease; ALI, acute limb ischemia; ASA, aspirin; BMS, bare metal stent; BPG, bypass graft; BTK, below the knee; BPG, bypass graft; CI, confidence interval; CLI, critical limb ischemia; CTO, chronic total occlusion; DES, drug eluting stent; DM, diabetes mellitus; DR, direct revascularization; EVT, endovascular treatment; HR, hazard ratio; IR, indirect revascularization; MALE major adverse limb event; N/A, not applicable; OR, odds ratio; PTA, percutaneous angioplasty; pt, patient; R/PTAS, recanalization, percutaneous transluminal angioplasty, and stenting; RR, relative risk; SES, self-expanding stents; and $TcPO_2$, transcutaneous oxygen pressure.

Evidence Table 41. RCTs of Surgical Revascularization for Chronic CLI–Section 8.2.

Study Acronym; Author; Year Published	Aim of Study; Study Type; Study Size (N)	Patient Population	Study Intervention (# patients) / Study Comparator (# patients)	Endpoint Results (Absolute Event Rates, P value; OR or RR; & 95% CI)	Relevant 2° Endpoint (if any); Study Limitations; Adverse Events
Abidia A, et al. 2003 (354) 12787692	**Aim**: Evaluate hyperbaric oxygen in pts with DM with ischemic nonhealing ulcer.<br><br>**Study type**: Double blind RCT<br><br>**Size**: n=18 pts	**Inclusion criteria**:<br>• Ulcer >1 cm and <10 cm in maximum diameter which had not shown any signs of healing, despite optimum medical management for more than 6 wk since presenting.<br>• ABI <0.8 (or great TBI <0.7 if calf vessels were incompressible).<br>• Pts with DM, HgbA1c <8.5%.<br><br>**Exclusion criteria**: Pts for whom vascular surgery, angioplasty or thrombolysis was planned	**Intervention**: 100% oxygen (Tx at 2.4 Atmospheres of absolute pressure for 90 min daily (30 treatments).<br><br>**Comparator**: Air Tx at 2.4 Atmospheres of absolute pressure for 90 min daily (30 treatments).	**1° endpoint**:<br>• At 6 wk follow-up, complete healing was achieved in 5 of 8 ulcers in the Tx group compared with 1 of 8 ulcers in the control group.<br>• The respective results at 1 y follow-up were 5 of 8 and 0 of 8 (p=0.026)<br>• 6 wk follow-up the median decrease of the wound areas in the Tx group was 100% compared with 52% in the control group (p=0.027). However, values at 6 mo follow-up were 100% and 95% respectively.	N/A
**STILE**<br>Weaver FA, et al. 1996 (355) 8911400	**Aim**: LE lysis vs. surgical revascularization with and without prior endovascular	**Inclusion criteria**: LE ischemia<br><br>**Exclusion criteria**: N/A	**Intervention**: Thrombolysis<br><br>**Comparator**: Surgical revascularization	**1° endpoint**: At 1 y, the incidence of recurrent ischemia (64% vs. 35%; p<0.0001) and major amputation (10% vs. 0%; p=0.0024) was increased in pts who were randomized to lysis.	• Factors associated with a poor lytic outcome included FP occlusion, diabetes, and critical ischemia.<br>• No differences in mortality rates were observed at 1 y between the

© American Heart Association, Inc. and American College of Cardiology Foundation

	intervention<br><br>**Study type** : RCT<br><br>**Size:** n=237 pts				lysis and surgical groups.
**TOPAS**<br>Ouriel K, et al. 1998 (356)<br>9545358	**Aim:** LE lysis vs. surgical revascularization with and without prior endovascular intervention<br><br>**Study type** : RCT Multicenter<br><br>**Size:** n=544 pts	**Inclusion criteria:** Acute thrombotic or embolic occlusion of a leg (native artery or bypass graft) within 14 d before randomization that met the guidelines for reversible limb-threatening ischemia<br><br>**Exclusion criteria:** Women who were pregnant or in whom pregnancy was a possibility.	**Intervention:** Thrombolysis with urokinase<br><br>**Comparator:** Surgical revascularization	**1° endpoint:**<br>• Final angiograms, which were available for 246 pts treated with urokinase, revealed recanalization in 196 (79.7%) and complete dissolution of thrombus in 167 (67.9%).<br>• Both Tx groups had similar significant improvements in mean ABI.<br>• Amputation-free survival rates in the urokinase group were 71.8% at 6 mo and 65.0% at 1 y, as compared with respective rates of 74.8% and 69.9% in the surgery group;<br>• 6 mo differences 95% CI: 10.5%–4.5%; p=0.43.<br>• 1 y differences 95% CI: -12.9%–3.1%; p=0.23.<br>• At 6 mo the surgery group had undergone 551 open operative procedures (excluding amputations), as compared with 315 in the thrombolysis group.	Major hemorrhage occurred in 32 pts in the urokinase group (12.5%) as compared with 14 pts in the surgery group (5.5%) (p=0.005). There were 4 episodes of intracranial hemorrhage in the urokinase group (1.6%), 1 of which was fatal. By contrast, there were no episodes of intracranial hemorrhage in the surgery group.
Dutch Iliac Stent Trial Study Group<br>Tetteroo E, et al. 1998 (221)<br>9643685	**Aim:** To determine outcomes between direct stent vs. delayed stent placement after angioplasty<br><br>**Study type:** RCT<br><br>**Size:** n=279 pts	**Inclusion criteria:** IC on the basis of iliac-artery stenosis of more than 50%, proven by angiography<br><br>**Exclusion criteria:** Women who were pregnant or in whom pregnancy was a possibility were excluded.	**Intervention:** Primary angioplasty with subsequent stent placement in case of a residual mean pressure gradient >10 mm Hg across the treated site group II<br><br>**Comparator:** Direct stent placement, group I	**1° endpoint:**<br>• In group II, selective stent placement was done in 59 (43%) of the 136 pts. The mean follow-up was 9.3 mo (range 3–24). Initial hemodynamic success and complication rates were 119 (81%) of 149 limbs and 6 (4%) of 143 limbs (group I) vs. 103 (82%) of 126 limbs and 10 (7%) of 136 limbs (group II), respectively.<br>• Clinical success rates at 2 y were 29 (78%) of 37 pts and 26 (77%) of 34 pts in groups I and II, respectively (p=0.6); however, 43% and 35% of the pts, respectively, still had symptoms.<br>• QoL improved significantly after	N/A

© American Heart Association, Inc. and American College of Cardiology Foundation

				intervention (p<0.05) but we found no difference between the groups during follow-up. 2 y cumulative patency rates were similar at 71% vs. 70% (p=0.2), respectively, as were reintervention rates at 7% vs. 4%, respectively (95% CI -2% to 9%).	
**CRISP-US**<br>Ponec D, et el. 2004 (357)<br>15361558	**Aim**: Compare SMART stent vs. Wallstent after suboptimal PTA.<br><br>**Study type**: RCT multicenter<br><br>**Size**: n=203 pts	**Inclusion criteria**: Chronic limb ischemia<br><br>**Exclusion criteria**: N/A	**Intervention**: Smart Stent<br><br>**Comparator**: Wall stent	**1° endpoint**: 9 mo composite end point rate was equivalent for the SMART stent and Wallstent (6.9% vs. 5.9%), with low rates of restenosis (3.5% vs. 2.7%), death (2.0% vs. 0.0%), and revascularization (2.0% vs. 4.0%) in the 2 groups. Primary patency at 12 mo was 94.7% and 91.1% with the SMART stent and Wallstent, respectively. Functional and hemodynamic improvement was also comparable between the groups. The frequency of major adverse events was similar at 1 y (4.9% vs. 5.9%).	The acute procedural success rate was higher in the SMART stent group (98.2% vs. 87.5%; p=0.002).
**CRISP-US**<br>Schillinger M, et al.<br>2006 (358)<br>16672699	**Aim**: Primary Stent vs. Angioplasty<br><br>**Study type**: RCT multicenter<br><br>**Size**: n=104 pts	**Inclusion criteria**: Severe claudication or chronic limb ischemia due to stenosis or occlusion of the SFA<br><br>**Exclusion criteria**: N/A	**Intervention**: Self-expanding nitinol stent<br><br>**Comparator**: Angioplasty	1° endpoint: At 6 mo, the rate of restenosis on angiography was 24% in the stent group and 43% in the angioplasty group (p=0.05); at 12 mo the rates on duplex ultrasonography were 37% and 63%, respectively (p=0.01). Pts in the stent group were able to walk significantly farther on a treadmill at 6 and 12 mo than those in the angioplasty group.	Angiographic follow-up was not done in all pts, resulting in lack of quantitative data on lumen diameter, residual stenosis, etc.
BASIL<br>Adam DJ, et al.<br>2005 (328)<br>16325694	**Aim**: Infrainguinal surgical bypass vs. PTA for CLI<br><br>**Study type**: RCT<br><br>**Size**: n= 452 pts	**Inclusion criteria**: CLI due to infrainguinal PAD<br><br>**Exclusion criteria**: N/A	**Intervention**: PTA (N=224)<br><br>**Comparator**: Bypass (N=228)	**1° endpoint**: Amputation free survival<br><br>**Safety endpoint**: Mortality	• Equal outcomes<br>• The trial ran for 5.5 y, and follow-up finished when pts reached an endpoint (amputation of trial leg above the ankle or death). 7 pts were lost to follow-up after randomization (3 assigned angioplasty, 2 surgery); of these, 3 were lost (1 angioplasty, 2 surgery) during the first y of follow-up. 195 (86%) of 228 pts assigned to bypass surgery and 216 (96%) of 224 to

© American Heart Association, Inc. and American College of Cardiology Foundation

					balloon angioplasty underwent an attempt at their allocated intervention at a median (IQR) of 6 (3–16) and 6 (2–20) d after randomization, respectively. At the end of follow-up, 248 (55%) pts were alive without amputation (of trial leg), 38 (8%) alive with amputation, 36 (8%) dead after amputation, and 130 (29%) dead without amputation. After 6 mo, the 2 strategies did not differ significantly in amputation-free survival (48 vs. 60 pts; unadjusted HR: 1.07; 95% CI: 0.72–1.6; adjusted HR: 0.73; 95% CI: 0.49–1.07). No difference in health-related quality of life between the 2 strategies, but for the first y the hospital costs associated with a surgery-first strategy were about 1/3 higher than those with an angioplasty-first strategy.
**PREVENT III**<br>Conte MS, et al. 2006 (359)<br>16616230	**Aim**: Reduce stenosis in Surgical bypass for CLI using E2F decoy<br><br>**Study type**: Prospective, randomized, double blinded, phase III RCT<br><br>**Size**: n=1,404 pts	**Inclusion criteria**: Pts with CLI (R4-6) who had autologous vein graft randomized to placebo or E2F decoy<br><br>**Exclusion criteria**: IC, hypercoagulable state, revisions of infrainguinal bypass grafts	**Intervention**: PTA (N=517)<br><br>**Comparator**: Bypass (N=341)	**1° endpoint**: Nontechnical index graft failure resulting in revision or major amputation<br><br>**Safety endpoint**: All-cause graft failure, freedom from significant index graft stenosis, amputation, index graft failure survival, graft patency, and limb salvage	• 2.7% 30 d mortality<br>• 4.7% MI<br>• 5.2% early graft occlusion<br>• Primary patency at 1 y: 61%<br>• Primary assisted patency: 77%<br>• Secondary patency: 80%<br>• Limb salvage: 88%
**BEST-CLI**<br>Farber A, et al. 2014 (360)<br>25241324	**Aim**: To compare best endovascular vs. best surgical therapy in pts with CLI. Compare treatment efficacy,	**Inclusion criteria**: Pts with CLI (R4-6)<br><br>**Exclusion criteria**: N/A	**Intervention**: Endovascular Tx (n=1,050)<br><br>**Comparator**: Bypass (N=1,050)	**1° endpoint**: MALE-free survival<br><br>**Safety endpoint**:<br>• MALE-POD (i.e., death within 30 d of procedure)	N/A

© American Heart Association, Inc. and American College of Cardiology Foundation

	functional outcomes, and cost in pts with CLI undergoing best open surgical or best endovascular revascularization<br><br>**Study type:** A prospective, multicenter, RCT. CLI trial has a 2-cohort design. The first cohort (1,620 pts) evaluates outcomes in pts who have adequate single segment great saphenous vein. The second cohort (480 pts) will study pts who do not have adequate single segment great saphenous vein.<br><br>**Size:** n=2,100 pts			• Freedom from perioperative death<br>• Freedom from MI<br>• Freedom from stroke, freedom from reinterventions (major and minor) in index leg, number of reinterventions (major and minor) per limb salvaged<br>freedom from clinical failure<br>• Freedom from CLI<br>• Freedom from all-cause mortality<br>• Freedom from hemodynamic failure.	
Veves A, et al. 2002 (361)<br>12093340	**Aim:** To compare a collagen and oxidized cellulose dressing to moistened gauze with regards to wound healing.<br><br>**Study Type:** RCT<br><br>**Size:** n=276 pts	**Inclusion criteria:** ≥8 y of age with a diabetic foot ulcer ≥30 d duration, Wagner grade 1–2, and an area of ≥1 cm^2 (greatest length × greatest width). Pts had adequate circulation with an oscillometer reading of the limb that had the target wound of ≥1 U and a wound that was debrided of	**Intervention:** Promogran, a wound dressing consisting of collagen and oxidized regenerated cellulose for diabetic plantar ulcers.<br><br>**Comparator:** Moistened Gauze with secondary dressing.	**1° endpoint:**<br>• Complete healing of the study ulcer (wound)<br>• After 12 wk of treatment, 51 (37.0%) Promogran treated pts had complete wound closure compared with 39 (28.3%) control pts, but this difference was not statistically significant (p=0.12).<br>• The difference in healing between Tx groups achieved borderline significance in the subgroup of pts with wounds of <6 mo duration. In pts with ulcers <6 mo duration,	Limitations: Study did not standardize frequency of dressing changes.

© American Heart Association, Inc. and American College of Cardiology Foundation

		necrotic/nonviable tissue at enrollment.<br><br>**Exclusion criteria:**<br>• Clinical signs of infection<br>• A target wound that had exposed bone<br>• A concurrent illness or a condition that may have interfered with wound healing, known hypersensitivity to any of the dressing components<br>• Unwillingness or inability of an ambulatory pt to be fitted with appropriate shoe gear or an off-loading device<br>• Multiple diabetic ulcers on the same foot.		43 (45%) of 95 Promogran-treated pts healed compared with 29 (33%) of 89 controls (p=0.056). In the group with wounds <6 mo duration, similar numbers of pts healed in the Promogran (8/43 [19%]; p=0.83) groups. No differences were seen in the safety measurements between groups.	

ABI indicates ankle-brachial index; CI, confidence interval; CLI, critical limb ischemia; DM, diabetes mellitus; E2F, egifoligide; FP, femoral popliteal; HgbA1c, hemoglobin A1c; HR, hazard ratio; IC, intermittent claudication; IQR, interquartile range; LE, lower extremity; MALE, major adverse limb event; MALE-POD, major adverse limb event perioperative death; N/A, not applicable; PTA, percutaneous angioplasty; pt, patient; QoL, quality of life; RCT, randomized controlled trial; SFA, superficial femoral artery; TBI, toe-brachial index; and tx, treatment.

Evidence Table 42. Nonrandomized Trials, Observational Studies, and/or Registries for Surgical Revascularization for Chronic CLI–Section 8.2.

Study Acronym (if applicable) Author Year	Study Type/Design; Study Size	Patient Population	Primary Endpoint and Results (include P value; OR or RR; and 95% CI)	Summary/Conclusion Comment(s)
Biancari F and Juvonen T<br>2014 (60)<br>24491282	**Aim:** Compare direct vs. indirect revascularization for wound healing and limb salvage.	**Inclusion criteria:** Prospective and retrospective observational studies with surgical, endovascular, or hybrid revascularization.	**Intervention:** Indirect Revascularization<br><br>**Comparator:** Direct Revascularization<br><br>**1° endpoint:** The risk of unhealed wound was significantly lower after direct revascularization (HR:	• Pooled limb salvage rates after direct and indirect revascularization were at 1 y 86.2% vs. 77.8% and at 2 y 84.9% vs. 70.1%, respectively.<br>• The analysis of 3 studies reporting only on pts with DM confirmed the benefit of direct revascularization in terms of limb salvage (HR:

© American Heart Association, Inc. and American College of Cardiology Foundation

	**Study type**: 9 Study Meta-Analysis<br><br>**Size**: n=1,290 Legs	**Exclusion criteria**: Data in abstracts alone, trials not reporting 6 mo data.	0.64; 95% CI: 0.52–0.8; r^2: 0%; 4 studies included) compared with indirect revascularization.<br><br>Direct revascularization was also associated with significantly lower risk of major amputation (HR: 0.44; 95% CI: 0.26–0.75; r^2: 62%; 8 studies included).	0.48; 95% CI: 0.31–0.75; r^2: 0%; 4 studies included)
Fogle MA, et al. 1987 (362) 3795391	**Study type**: Retrospective observational study<br><br>**Size**: n=675 grafts, 582 pts	**Inclusion criteria**: Disabling claudication and/or limb salvage, defined by the presence ischemic rest pain or tissue necrosis or schemic rest pain or tissue necrosis.<br><br>**Exclusion criteria**: Pts undergoing intervention for indications other than atherosclerotic disease.	**Intervention**: In Situ Vein Graft<br><br>**Comparator**: Reversed vein graft<br><br>**1° endpoint**:<br>In situ cumulative patency<br>1 y 85%<br>3 y 85%<br><br>Reversed vein cumulative patency<br>1 y 81%<br>3 y 73%	• Reversed vein patency at 5 y 63%<br>• Infrapopliteal reversed vein cumulative patency 3 y 62%<br>• Infrapopliteal in situ cumulative patency 3 y 87%<br>• Limitation: Study only examined cumulative patency not primary patency, etc.
Rashid H, et al. 2013 (363) 23523278	**Aim**: The effect of pedal arch quality on the amputation-free survival and patency rates of distal bypass grafts and its direct impact on the rate of healing and time to healing of tissue loss after direct angiosome revascularization in pts with CLI.<br><br>**Study type**: Restrospective<br><br>**Size**: n=154 pts	**Inclusion criteria**: CLI Rutherford Class 4–6<br><br>**Exclusion criteria**: N/A	**Intervention**:<br>Pts with a CPA, IPA, and NPA, all underwent infrapopliteal bypass.<br><br>**1° endpoint**:<br>• The primary patency rates at 1 y in the CPA, IPA, and NPA groups were 58.4%, 54.6%, and 63.8%, respectively (p=0.5168)<br>• Secondary patency rates were 86.0%, 84.7%, and 88.8%, respectively (p=0.8940)<br>• Amputation-free survival at 48 mo was 67.2%, 69.7%, and 45.9%, respectively (p=0.3883)	• Tissue loss was present in 141 of the 167 bypasses. In the CPA group, 83% of tissue loss with DAR healed compared with 92% in the non-DAR (median time to healing, 66 vs. 74 d).<br>• Similarly, in the IPA group, 90% with DAR healed compared with 81% in the non-DAR (median time to healing, 96 vs. 86 d). In the NPA group, only 75% with DAR healed compared with 73% in the non-DAR (median time to healing, 90 vs. 135 d). There was a significant difference in healing and time to healing between the CPA/IPA and NPA groups (p=0.0264).<br>• Limitation: Study did not stratify pts with underlying renal disease. Wound care techniques were not completely standardized.
Nolan BW, et al. 2011 (364) 21802888	**Aim**: LE bypass with and without prior endovascular intervention	**Inclusion criteria**: CLI (rest pain or tissue loss)<br><br>**Exclusion criteria**: N/A	**Intervention**: LE bypass post endovascular intervention.<br><br>**Comparator**: Primary LE bypass	N/A

© American Heart Association, Inc. and American College of Cardiology Foundation

	**Study type**: Retrospective cohort analysis (10 Centers)<br><br>**Size**: n=1,880 LE bypasses		**1° endpoint**: Major amputation and graft occlusion at 1 y postoperatively. Secondary outcomes included in-hospital MAE, 1 y mortality, and composite 1 y MALE.<br><br>Prior PVI or bypass did not alter 30 d MAE and 1 y mortality after the index bypass.<br><br>1 y major amputation and 1 y graft occlusion rates were significantly higher in pts who had prior iPVI than those without (31% vs. 20%; p=0.046 and 28% vs. 18%; p=0.009), similar to pts who had a prior ipsilateral bypass (1 y major amputation, 29% vs. 20%; p=0.022; 1 y graft occlusion, 33% vs. 18%; p=0.001).	
Santo VJ, et al. 2014 (365) 24613692	**Aim**: LE bypass with and without prior endovascular intervention<br><br>**Study type**: Retrospective<br><br>**Size**: n=314 autologous vein LE bypasses	**Inclusion criteria**: CLI LEBs were performed for CLI, 71% for tissue loss. TASC II type D or type C lesions were present in 62% and 25%, respectively.<br><br>**Exclusion criteria**: N/A	**Intervention**: LE bypass post endovascular intervention. PEI<br><br>**Comparator**: Primary LE bypass NPEI<br><br>**1° endpoint**:<br>• The 30-day mortality rate was 3.5%.<br>• Overall, Primary patency rates at 1 y and 5 y were 61% and 45%.<br>• The 5 y limb salvage rate was 89%, and the 5 y amputation-free survival was 49%.<br>• The 1 y primary patency rate was 62% for NPEI pts vs. 59% for PEI pts (p=0.759).<br>The 3 y limb salvage rate was 89% for NPEI pts vs. 92% for PEI pts (p=0.445).<br>• The 3 y amputation-free survival was 59% for NPEI pts vs. 52% for PEI pts (p=0.399). Median follow-up time was 323 d for NPEI pts (IQR: 83–918) vs. 463 d for PEI pts (IQR: 145–946; p=0.275).	N/A
Uhl C, et al. 2014 (366) 24418639	**Aim**: Pedal bypass surgery with and without prior endovascular intervention<br><br>**Study type**:	**Inclusion criteria**: CLI with rest pain, ulcers, or gangrene (Rutherford 4–6), who then required pedal bypass either as primary therapy or after prior endovascular	**Intervention**: Pedal Bypass post intervention. PEI<br><br>**Comparator**: Primary pedal bypass. BSF<br><br>**1° endpoint**:<br>• Overall, primary patency at 1 y was 58.3%, and secondary patency was 61.3%.	N/A

© American Heart Association, Inc. and American College of Cardiology Foundation

	Retrospective<br><br>**Size**: n=75 pedal bypass operations in 71 pts	intervention.<br><br>**Exclusion criteria**: N/A	• Limb salvage was 76.8% and survival was 80.4%<br>• Graft occlusion within 30 d was 18.7%. Revision in those cases was futile and 78.6% of pts had to undergo major amputation.<br>• Primary patency at 1 y was 67.0% in PEI group vs. 48.3% in BSF group (p=0.409) and secondary patency was 73.5% vs. 48.6% (p=0.100).<br>• Prior endovascular intervention had no significant impact on either limb salvage (82.3% vs. 71.6% at 1 y; p=0.515) or graft occlusions within 30 d (19.4% vs. 17.9%; p=0.547).<br>• Survival rate at 1 y was 79.5% in PEI group and 81.3% in BSF group (p=0.765).	
Korhonen M, et al.<br>2011 (367)<br>21195637	**Aim**: Compare Fem-pop PTA vs. surgical bypass for CLI<br><br>**Study type**: Observational single center<br><br>**Size**: n=858 pts	**Inclusion criteria**: Consecutive pts enrolled<br><br>**Exclusion criteria**: N/A	**Intervention**: PTA (N=517)<br><br>**Comparator**: Bypass (N=341)<br><br>**1° endpoint**:<br>• Mortality, limb salvage, AFS, Freedom from repeat intervention<br>• Mortality: (30 d, 1 y, 3 y): Endo: 5.1%, 24.3%, 41.1%<br>• Surgery: 2.4%, 17.8%, 35%<br>• LIMB SALVAGE: (1 y, 3 y, 5 y): Endo: 87%, 77%, 75.3%<br>• Surgery: 95%, 77%, 75.3%<br>• No significant difference in AFS after propensity score adjustment	N/A
Kasemi H, et al.<br>2016 (368)<br>26370748	**Aim**: To evaluate endovascular treatment of AIOD<br><br>**Study Type**<br>Retrospective<br><br>**Size**: n=22 pts.	**Inclusion criteria**: Indication for treatment were long-segment (>10 cm) TASC type D aortoiliac occlusion (2 suprarenal, 4 juxtarenal, and 16 infrarenal), extending to the common or iliac arteries (EIAs). Clinical indication for endovascular therapy was severe claudication or CLI.	A total of 22 pts underwent total endovascular treatment of AIOD from<br>January 2008–September 2014. BMSs in kissing configuration were deployed<br>in 9 cases, covered stents in kissing configuration in 9 pts and the aortic bifurcation<br>Teconstruction with the Y-guidewire configuration technique was performed in the last 4 pts.<br><br>**1° endpoint**:<br>• Technical success was 100%. Perioperative mortality rate was 4.5%. ABI improved from 0.49 ± 0.19 to 0.96 ± 0.05 at the right side and from 0.53 ± 0.17	N/A

© American Heart Association, Inc. and American College of Cardiology Foundation

		**Exclusion criteria**: Pts with inflammatory occlusive vascular disease and aortoiliac thromboembolic occlusion were excluded from the study.	• 0.98 ± 0.04 at the left side (p<0.01). Mean follow-up was 39.5 mo (range, 5–80 mo).<br>• The primary patency rate was 95.2% at 1 y and 90.5% at 3 y	
Bredahl K, et al. 2015 (369) [26115920](26115920)	**Aim:** To identify the effect of growing endovascular repair on open aortic repair outcomes.<br><br>**Study Type:** Retrospective<br><br>**Size:** n=3,623 aortobifemoral and 144 aortobiiliac bypass procedures	**Inclusion criteria:** Bypass procedures performed in Denmark due to chronic IC or chronic CLI<br><br>**Exclusion criteria:** We excluded pts with acute limb ischemia, secondary renovascular hypertension, secondary mesenteric ischemia, secondary aneurysm, and pts who had previously undergone intra-abdominal vascular surgery.	**Intervention:** Open Bypass<br><br>**1° endpoint:**<br>• The annual caseload fell from 323 to 106 during the study period, but the 30 d mortality at 3.6% (95% CI: 3.0–4.1) and the 30 d major complication rate remained constant at 20% (95% CI: 18–21).<br>• Gangrene (OR: 3.3; 95% CI: 1.7–6.5; p=0.005) was the most significant risk factor for 30-day mortality, followed by renal insufficiency (OR, 2.5; 95% CI, 1.1-5.8; p=0.035) and cardiac disease (OR: 2.1; 95% CI: 1.4–3.1; p<0.001).<br>• Multiorgan failure, mesenteric ischemia, need for dialysis and cardiac complications were the most lethal complications, with mortality rates of 94%, 44%, 38%, and 34%, respectively.	N/A
Chew DK, et al. 2001 (370) [11174776](11174776)	**Aim:** To evaluate the long-term results of autogenous composite vein grafts used for infrainguinal arterial bypass grafting<br><br>**Study Type:** Retrospective<br><br>**Size:** n=154 pts	**Inclusion criteria:**<br>• 90% of the operations were performed for limb salvage (rest pain: 36%; ulcer: 33%; gangrene: 21%); the rest were for severe claudication.<br>• 48% of bypass grafts were performed after failed previous reconstructions.	**Intervention:** Infrainguinal bypasses using composite vein grafts were examined<br><br>**1° endpoint:**<br>• The 30 d operative mortality rate was 1.8%. Perioperative graft failure (<30 d) occurred in 18 bypass grafts (11%), resulting in early amputation (<30 d) in 1.2%.<br>• Overall, 5 y cumulative patency rates were 44% ± 5% for primary patency, 63% ± 5% for PAP, and 65% ± 5% for secondary patency SP.<br>• A high revision rate for stenosis or thrombosis was required during follow-up to maintain patency of the grafts (27%).<br>Limb salvage was 81% ± 5% at 5 y.	N/A

© American Heart Association, Inc. and American College of Cardiology Foundation

			• Primary reconstructions with composite vein fared significantly better than secondary reconstructions (SP 76% vs. 54% at 5 y; p<0.01).<br>• Arm vein composites showed superior patency compared with greater saphenous vein composites (SP 79% vs. 61% at 5 y, p<0.05).	

ABI indicates ankle-brachial index; AFS, amputation free survival; AIOD, aortoiliac occlusive disease; BMS, bare metal stent; BSF, bypass surgery as first-line treatment; CI, confidence interval; CLI, critical limb ischemia; CPA, complete pedal arch; DAR, direct angiosome revascularization; DM, diabetes mellitus; EIA, external iliac artery; HR, hazard ratio; IC, intermittent claudication; IPA, incomplete pedal arch; iPVI, ipsilateral peripheral endovascular intervention; IQR, interquartile range; LEB, lower extremity bypass; LE, lower extremity; MAE, major adverse event; MALE, major adverse limb event; N/A, not applicable; NPA, no pedal arch; NPEI, no prior endovascular intervention; PAP, primary assisted patency; PEI, prior endovascular intervention; PTA, percutaneous angioplasty; pt, patient; PVI, peripheral endovascular intervention; SP, secondary patency; and TASC, TransAtlantic Inter-Society Consensus.

Evidence Table 43. RCT Comparing Prostanoids for End-Stage Peripheral Artery Disease–Section 8.2.3.

Study Acronym; Author; Year Published	Aim of Study; Study Type; Study Size (N)	Patient Population	Study Intervention (# patients) / Study Comparator (# patients)	Endpoint Results (Absolute Event Rates, P value; OR or RR; & 95% CI)	Relevant 2° Endpoint (if any); Study Limitations; Adverse Events
Ruffolo AJ, et al. 2010 (371) 20091595	**Aim:** Evaluation of the "effectiveness and safety of prostanoids in pts with CLI"<br><br>**Study type:** Meta-analysis and systematic review of randomized trials<br><br>**Size:** n=2,724 pts from 20 randomized trials	**Inclusion criteria:** CLI "without chance of rescue or reconstructive intervention"<br><br>**Exclusion criteria:** Trials in which treatment assignment was not masked; withdrawal of ≥10% of study population; no ITT analysis.	**Intervention:** Prostanoid administration (including prostaglandin E1, prostacyclin, iloprost, betaprost, cisaprost)<br><br>**Comparator:** Placebo or other pharmacologic control	**1° endpoint:** Decrease in rest pain relief (RR: 1.32; 95% CI: 1.10–1.57) and ulcer healing (RR: 1.54; 95% CI: 1.22–1.96) but no class effect on amputations (24.8 vs. 26.7%; RR: 0.89; 95% CI: 0.76–1.04). Iloprost specifically associated with decreased amputation rate (RR: 0.69; 95% CI: 0.52–0.93)<br><br>**1° Safety endpoint:** No effect on mortality (RR: 1.07; 95% CI: 0.65–1.75); higher risk of adverse events (RR: 2.35; 95% CI: 1.99–2.78)	• Adverse events included headache, flushing, nausea, vomiting, diarrhea<br>• "Amputation" not specifically defined if major only or total) in 9 of the trials<br>• Amputation rate of placebo group notably higher in iloprost studies (147 of 383, 38.4%) than overall (201 of 753, 26.7%)<br><br>**Summary**: Review "did not find any conclusive evidence that prostanoids provided long-term benefit."

CI indicates confidence interval; CLI, critical limb ischemia; ITT, intent to treat; pt, patient; RCT, randomized controlled trial; and RR, relative risk.

Evidence Table 44. Nonrandomized Trials, Observational Studies, and/or Registries for Would Healing Therapies for CLI–Section 8.2.3.

Study Acronym; Author;	Aim of Study; Study Type;	Patient Population	Endpoint Results (Absolute Event Rates, P value; OR	Relevant 2° Endpoint (if any); Study Limitations;

© American Heart Association, Inc. and American College of Cardiology Foundation

Year Published	Study Size (N)		or RR; & 95% CI)	Adverse Events
Moran PS, et al. 2015 (372) 25270409	**Aim:** Evaluation of IPC and standard medical therapy for pts who were "ineligible for revascularization"<br><br>**Study type:** Meta-analysis and systematic review of studies<br><br>**Size:** n=409 limbs in 8 series; no randomized trials identified	**Inclusion criteria:** CLI "ineligible for revascularization"; see Table 1 of publication for details<br><br>**Exclusion criteria:** N/A	**1° endpoint:** Significant improvements in limb salvage and wound healing rates (58 vs. 17% at 18 mo for both) in 1 controlled study; significant improvement in SF-36 quality of life domains in another controlled study; 10–15 mm Hg average increase in toe pressures<br><br>**1° Safety endpoint:** Compression therapy not completed because of pain in 7% of pts	• No randomized trials available; only 2 case series made comparisons to controls (total n=32)<br><br>**Summary**: "Limited available results suggest that IPC may be associated with improved limb salvage, wound healing, and pain management".
Kobayashi N, et al. 2015 (373) 25542618	**Aim:** Determine if endovascular therapy improves tissue loss in CLI pts<br><br>**Study type:** Prospective<br><br>**Size:** n=187 CLI pts; 113 with complete wound healing	**Inclusion criteria:** CLI pts with tissue loss who achieved complete wound healing after endovascular revascularization<br><br>**Exclusion criteria:** N/A	**1° endpoint:** Survival rate at 3 y 74%	**2° endpoint:** Limb salvage rate and recurrence rate at 3 y 100%<br><br>Recurrance rate of CLI at 3 y 9%
Armstrong DG, et al. 2012 (205) 22431496	**Study type:** NR, retrospective cohort<br><br>**Size:** n=790 diabetic foot operations	**Inclusion criteria:** All diabetic foot operations 2006–2008 vs. 2008-2010	**1° endpoint:** Amputation level, case mix<br><br>**Results:** 37.5% reduction in transtibial amputations; 44% increase in vascular interventions	Interdisciplinary care as a "rapid and sustained impact in changing surgery type from reactive to proactive" and reduces major amputations
Chung J, et al. 2015 (206) 25073577	**Study type:** NR, retrospective cohort<br><br>**Size:** n=85 pts	**Inclusion criteria:** "All consecutive pts" with R5/6 CLI at a single hospital 8/2010–6/2012	**1° endpoint:** 1 y amputation-free survival<br><br>**Results:** 67 vs. 42% at 1 y; also higher mean limb salvage times. Multidisciplinary care remained significant on multivariate analysis	Multidisciplinary care improves amputation-free survival in pts with R5/6 CLI
Vartanian et al. 2015 (211) 25596408	**Study type:** NR, retrospective review	**Inclusion criteria:** Pts with neuroischemic wounds treated at a signle institutional	**1° endpoint:** Time to wound healing, reulceration rate, and ambulatory status.	Multidisciplinary care helps effectively heal wounds and maintain ambulatory status in pts

© American Heart Association, Inc. and American College of Cardiology Foundation

	**Size**: n=91 limbs from 89 pts	amputation prevention clinic from March 2012–July 2013. Pts at highest risk for limb loss, defined as ischemic wounds (ischemic ulcer or gangrene) or diabetic foot ulcers.<br><br>**Exclusion criteria**: New pts evaluated for benign conditions (e.g., arthritis, overuse injuries, simple infections in nondiabetics, venous ulcers, minor trauma, radiculopathy).	**Results**: 67% of wounds were present >6 wk before referral. A total of 151 podiatric and 86 vascular interventions were prformed, with an equal distribution of endovascular and open revascularizations. Complete wound healing observed in 59% of wounds, and average time to full healing was 12 wk. Hindfood wounds predictive of failure to heal (OR: 0.21; p<0.01; 95% CI: 0.06–0.68).	with limb threatening neuroischemic wounds. Hindfoot or ankle wounds can adversely influence the outcome. Healing can be prolonged and a substancial proportion of pts can be expected to have a recurrence, therefore surveillance is mandatory. A coordinated amputation prevention program may help to minimize hospital readmissions in the high-risk population.

CLI indicates critical limb ischemia; IPC, intermittent pneumatic compression; and N/A, not applicable.

Evidence Table 45. Nonrandomized Trials, Observational Studies, and/or Registries of Acute Limb Ischemia–Section 9.1.

Study Acronym; Author; Year Published	Study Type/Design; Study Size	Patient Population	Primary Endpoint and Results (include P value; OR or RR; & 95% CI)	Summary/Conclusion Comment(s)
Rutherford RB, et al. 1992 (374)<br>9308598	**Study type**: Consensus Document<br><br>**Size**: N/A	**Inclusion criteria**: N/A<br><br>**Exclusion criteria**: N/A	**1° endpoint**: Scoring Scheme for ALI<br><br>**Results**: N/A	N/A
Nypaver TJ, et al. 1998 (375)<br>9737621	**Study type**: Single institution retrospective cohort<br><br>**Size**: n=71 pts	**Inclusion criteria**: Acute arterial ischemia and required an urgent/emergent LE arterial bypass reconstruction<br><br>**Exclusion criteria**: N/A	**1° endpoint**: Outcome of arterial bypass reconstruction in the setting of acute arterial ischemia<br><br>**Results**: N/A<br>• Mean duration of symptoms was 43 h (median 24), and mean time from hospital presentation to the operating room was 36 h (median 12)<br>• Death, limb loss, or both, were associated with a paralytic limb (p=0.001) and congestive heart failure (p=0.03)	N/A
Fogarty TJ and Cranley JJ	**Study type**: Descriptive	**Inclusion criteria**: N/A	**1° endpoint**: N/A	• First description of embolectomy catheter

© American Heart Association, Inc. and American College of Cardiology Foundation

1965 (376)<br>14263952	**Size**: n=56 episodes of embolism occurring in 50 pts	**Exclusion criteria**: N/A	**Results**: N/A	
Shin HS, et al.<br>2013 (377)<br>24436594	**Study type**: Single institution<br><br>**Size**: n=18 acutely ischemic limbs in 14 consecutive pts	**Inclusion criteria**: All pts with ALI<br><br>**Exclusion criteria**: N/A	**1° endpoint**: Limb salvage via novel surgical approach<br><br>**Results**: Of 14 pts, 1 died and 1 underwent amputation. After 1 wk of anticoagulation therapy, ≥2 arterial pulses were detected at the ankles in all 15 limbs from the remaining 12 pts. All 15 limbs were salvaged successfully.	• CTA for Dx<br>• 71% heart disease:<br>57% atrial fibrillation<br>14% had a Hx of previous MI<br>• 86% of pts with mixed thromboembolic disease<br>• Below-knee exposure and 1 vessel runoff
de Donato G, et al.<br>2014 (378)<br>24342067	**Study type**: Single institution cohort<br><br>**Size**: n=322 pts	**Inclusion criteria**: All pts w ALI<br><br>**Exclusion criteria**: ALI from graft thrombosis	**1° endpoint:**<br>• In-hospital complications<br>• 30 d mortality<br>• Primary and secondary patency<br>• Reintervention rate<br>• Limb salvage<br>• Overall survival rates<br><br>**Results:** Reduction in complications when hybrid techniques utilized as opposed to just thromboembolectomy	• Thromboembolectomy alone in 35%<br>• 45.5% via CFA<br>• 30 d mortality 4.4%<br>• 15% in hospital complications<br>• 8 pts w complication from catheter
**VS.GNNE ALI**<br>Baril DT, et al.<br>2013 (379)<br>23714364	**Study type**: Registry review<br><br>**Size**: n=323 pts	**Inclusion criteria**: All pts undergoing infrainguinal lower extremity bypass between 2003 and 2011 (ALI vs. CLI)<br><br>**Exclusion criteria**: N/A	**1° endpoint**: Major amputation and mortality<br><br>**Results**: ALI predictor of both major amputation (HR: 2.16; CI: 1.38–3.40; p=0.001) and mortality (HR: 1.41; CI: 1.09–1.83; p=0.009) at 1 y	• Age and gender similar to CLI<br>• ALI less likely to be on ASA (63% vs. 75%; p<0.0001) or a statin (55% vs. 68%; p<0.0001)<br>• ALI more likely to be current smokers (49%<br>vs. 39%; p<0.0001), to have had a prior ipsilateral bypass (33% vs. 24%; p=0.004) or a prior ipsilateral percutaneous intervention (41% vs. 29%; p=0.001])
Manojlović V, et al.<br>2013 (380)<br>23534299	**Study type**:<br>Retrospective study<br><br>**Size**: n=95 pts	**Inclusion criteria**: Pts operated on ≤6 h after onset of symptoms of ALI.<br><br>**Exclusion criteria**:	**1° endpoint**: Preserved extremity, amputation, and fatal outcome<br><br>**Results**:<br>• More pts had embolism of blood vessel	• Majority of pts age ≥70 y<br>• Surgical procedures showed no difference when final outcome analyzed<br>• Mortality rate was 10.5% and 7/10 pts with this outcome had severe form of

© American Heart Association, Inc. and American College of Cardiology Foundation

		Previous reconstructive procedures on blood vessels and where acute ischemia had been induced by trauma or aneurysmal disease of the peripheral blood vessels	(73.7%) compared to a chronic lesion (26.3%); p<0.05<br>• 86.2% of pts achieved successful revascularization<br>• 3.2% of pts had mputating treatment ≤30 d.<br>• 10.5% of pts had a fatal outcome	chronic myocardiopathy and metabolic decompensation<br>• High success rate, with successful revascularization of LE achieved in 85%. This demonstrates benefits of early operative treatment in ALI, regardless of the clause of ischemia (thrombosis or embolism)
Duval S, et al. 2014 (381) 25262269	**Study type:** Registry<br><br>**Size:** n=200 pts	**Inclusion criteria:**<br>• Limb threatening ischemia<br>• Enrolled in the FRIENDS registry<br><br>**Exclusion criteria:**<br>• N/A	**1° endpoint:** Amputation and mortality<br><br>**Results:**<br>• Duration of limb ischemia in pts with ALI was associated with much higher rates of first amputation (p= o.0002) and worse amputation-free survival (p=0.037). No significant associations were observed in pts with CLI.<br>• Increased duration of limb ischemia in pts with ALI was associated with progressively increased 30-day ambputation (p=0.028 for trend)	• The longer lower extremity symptoms in ALI occur, the less likely the possibility of salvage<br>• Limb ALI episodes are extreamly deadly, even with limb revascularization

ALI indicates acute limb ischemia; CI, confidence interval; CFA, common femoral artery; CLI, critical limb; CTA, computed tomography angiography; HR, hazard ratio; LE, lower extremity; MI, myocardial infarction; N/A, not applicable; OR, odds ratio; and RR, relative risk.

Evidence Table 46. Nonrandomized Trials, Observational studies, and/or Registries Comparing Evaluating Noninvasive Testing and Angiography for ALI–Section 9.1.

Study Acronym; Author; Year Published	Study Type/Design; Study Size	Patient Population	Primary Endpoint and Results (include P value; OR or RR; & 95% CI)	Summary/Conclusion Comment(s)
Morris-Stiff G, et al. 2009 (382) 19785938	**Study type:** Retrospective review comparing pts with ALI from 2 time periods<br><br>**Size:** n=205 pts	**Inclusion criteria:** Pts presenting with ALI during specified time period<br><br>**Exclusion criteria:** N/A	**Results:** Despite increased pre-operative (15% vs. 47%; p<0.05) and on-table imaging (0% vs. 16%; p<0.05) technical success did not improve.	• Delay from symptom onset to surgery is a major determinant of outcome.
Londero LS, et al. 2014 (383) 25400690	**Study type:** Prospective cross-sectional cohort study including all pts suspected with ALI<br><br>**Size:** n=42 pts	**Inclusion criteria:** All<br><br>**Exclusion criteria:** N/A	**1° endpoint:** 30 pts needed immediate intervention. In the group of 14 pts who had immediate operation, the median time from vascular evaluation to revascularization was 324.5 (122–873) min and in the group of 8 pts that went through an imaging procedure	• If CT or MRA was used the intervention was delayed by 3 h<br>• No clear delay to angiography, but thrombolysis duration was longer than surgery

© American Heart Association, Inc. and American College of Cardiology Foundation

			before an operation the median delay was 822 (494–1185) min from specialist assessment to revascularization. The median time for revascularization among 4 pts, who were treated with arterial thrombolysis was 5621 (1686–8376) min.	

ALI indicates acute limb ischemia; CI, confidence interval; CLI, critical limb ischemia; CT, computed tomography; DSA, digital subtraction angiography; DUAM, duplex ultrasound arterial mapping; HR, hazard ratio; N/A, not applicable; MRA, magnetic resonance angiography; OR, odds ratio; pt, patient; and RR, relative risk.

Evidence Table 47. RCTs of Revascularization Strategy for ALI–Section 9.2.2.

Study Acronym; Author; Year Published	Aim of Study; Study Type; Study Size (N)	Patient Population	Study Intervention (# patients) / Study Comparator (# patients)	Endpoint Results (Absolute Event Rates, P value; OR or RR; & 95% CI)	Relevant 2° Endpoint (if any); Study Limitations; Adverse Events
Ouriel K, et al. 1994 (384) 8201703	**Aim**: Catheter directed Intra-arterial urokinase vs. surgery<br><br>**Study type**: RCT<br><br>**Size**: n=57 pts IAT vs. n=57 pts surgery	**Inclusion criteria**: ALI <7 d<br><br>**Exclusion criteria**: Pts were excluded from study if they manifested a contraindication to thrombolytic therapy, including one or more of the following: a major operative procedure within 14 d, active peptic ulcer disease, an intracranial neoplasm, or a Hx of a cerebrovascular accident. Pts were also excluded if they had a contraindication to operative revascularization; non-ambulatory prior to ALI or Cr>2.5	**Intervention**: Catheter directed urokinase<br><br>**Comparator**: Surgery	**1° endpoint**:<br>• Limb salvage 82% at 12 mo both groups<br>• Survival 84% IAT vs. 58% surgery at 12 mo, p=0.01	• Increased cardiopulmonary complications in surgery group 49% vs. 16%, p=0.001
**TOPAS**<br>Ouriel K, et al. 1998 (356) 9545358	**Aim**: Catheter directed Intra-arterial urokinase vs. surgery<br><br>**Study type**: RCT<br><br>**Size**: n=272 pts IAT vs. n=272 pts surgery	**Inclusion criteria**: ALI ≤14 d<br><br>**Exclusion criteria**: pts ineligible for thrombolytics	**Intervention**: Catheter directered urokinase<br><br>**Comparator**: Surgery	**1° endpoint**: 6 mo amputation free survival 71.68 IAT vs. 74.8 surgery p=0.43<br><br>**Safety endpoint**: Mortality at hospital discharge 8.8 IAT vs. 5.9 surgery p=0.19	N/A

© American Heart Association, Inc. and American College of Cardiology Foundation

**STILE**<br>Graor RA, et al.<br>1994 (385)<br>8092895	**Aim**: Catheter directed Intra-arterial tPA or urokinase vs. surgery<br><br>**Study type**: RCT<br><br>**Size**: n=137 pts tPA, n=112 pts UK, N= 144 pts surgery	**Inclusion criteria**:<br>• 18–90 y<br>• Signs or symptoms of worsening limb ischemia within the past 6 mo who required intervention<br>• Angiographically documented nonembolic arterial or bypass graft occlusion<br><br>**Exclusion criteria**: infected grafts or contraindications to lytics	**Intervention**: Catheter directed urokinase or tPA<br><br>**Comparator**: Surgery	**1° endpoint**: Composite clinical outcome (see page 255 of mansuscript) 22.6% surgery vs. 38.3% IAT, p=0.011	• Note: failure of catheter placement occurred in 28% of IAT group resulting in large failure rate<br>• Poor quality study
Comerota AJ, et al.<br>1996 (386)<br>8795509	**Aim**: Surgery vs. CDT for occluded bypass grafts<br><br>**Study type**: RCT<br><br>**Size**: Surgery (n=46 pts) or CDT (n=78 pts)	**Inclusion criteria**: ALI <14 d or chronic ischemia >14 d<br><br>**Exclusion criteria**: contra-indications to thrombolysis	**Intervention**: CDT<br><br>**Comparator**: Surgery	**1° endpoint**:<br>• A composite clinical outcome including death, amputation, ongoing/recurrent ischemia, and major morbidity was analyzed on an intent-to-treat basis at 30 d and 1 y.<br>• Acutely ischemic pts (0–14 d) randomized to lysis demonstrated a trend toward a lower major amputation rate at 30 d (p=0.074) and significantly at 1 y (p=0.026) compared with surgical pts, while those with >14 d ischemia showed no difference in limb salvage but higher ongoing/recurrent ischemia in lytic pts (p<0.001)	• For ALI <14 d CDT is similar to surgery
Diffin DC and Kandarpa K<br>1996 (387)<br>8773976	**Aim**: Review the risks and benefits of PIAT vs. SR as initial tx for ALLI<br><br>**Study type**: Analysis of 2 RCTs<br><br>**Size**: SR (n=1,051 pts) or PIAT (n=895 pts)	**Inclusion criteria**: Published RCTs that compared PIAT with SR as the initial treatment of ALLI<br><br>**Exclusion criteria**: Studied that included >1 disease category but did not specifically stratify results by category	**Intervention**: PIAT<br><br>**Comparator**: SR	**1° endpoint**: Limb salvage and mortality at 30 d and 6–12 mo	• Limb salvage rates at 30 d for PIAT vs. SR: 93%; vs. 89%<br>• Limb salvage rates at 6–12 mo for PIAT vs. SR: 89%; vs. 73%<br>• PIAT better limb-salvage rate and mortality than SR in the treatment of ALLI

© American Heart Association, Inc. and American College of Cardiology Foundation

Schrijver AM,, et al.<br>2011 (388)<br>PMC3033836	**Study type**: RCT<br><br>**Size**: n=60 pts	**Inclusion criteria**:<br>• Pts age >18 y and <85 y<br>• Pts with thrombosed femoropopliteal or femorocrural native arteries or femoropopliteal or femorocrural venous or prosthetic bypass grafts with ischemic complaints between 1–7 wks<br>• Pts with acute lower limb ischaemia class I and IIa according to Rutherford classification<br>• Pts understand the nature of the procedure and provide written informed consent<br><br>**Exclusion criteria**:<br>• Isolated common femoral artery thrombosis<br>• localized emboli (<5 cm) or occlusions in the native femoropopliteal arteries<br>• Clinical complaints of ALI due to thrombosis of the femoropopliteal or femorocrural native arteries, or femoropopliteal or femorocrural venous or prothetic bypass grafts <1 wk and >7 wk<br>• ALI class IIb and III Rutherford classification<br>Antiplatelet therapy, anticoagulants, or thrombolytic drugs are contraindicated<br>• <6 wk ischemic stroke or cerebral bleeding<br>• 6 wk surger<br>• DBP >110 mm HG, SBP >200 mm Hg<br>• Current malignancy<br>• Hx of life-threatening reaction to contrast medium<br>• Uncorrected bleeding disorders<br>• Women with child-bearing potential not on contraceptives or currently breastfeeding	**Intervention**: Standard thrombolysis<br><br>**Comparator**: US-accelerated thrombolysis	**1° endpoint**: Duration of catheter-directed thrombolysis needed for uninterrupted flow in the thrombosed infrainguinal native artery or bypass graft, with outflow through ≥1 crural artery	• RCT comparing this technique to standard catheter-based thrombolytic therapy failed to demonstrate a difference in outcomes including bleeding despite a lower total amount of lytic delivered

© American Heart Association, Inc. and American College of Cardiology Foundation

		• pregnancy<br>• Hemodynamically unstable at the onset of the procedure<br>• Pts who refuse treatment<br>• Currently participating in another study<br>• Life expectancy of <1 mo<br>• Contraindication for MRI			

ALI indicates acute limb ischemia; ALLI, acute lower-limb ischemia; CDT, catheter-directed thrombolysis; CI, confidence interval; DBP, diastolic blood pressure; HR, hazard ratio; hx, history; IAT, intra-arterial treatment; MRI, magnetic resonance imaging; N/A, not applicable; OR, odds ratio; PIAT, peripheral intraarterial thrombolysis; pt, patient; RCT, randomized controlled trail; RR, relative risk; SR, surgical revascularization; SBP, systolic blood pressure; STILE, Surgery Versus Thrombolysis for Ischemia of the Lower Extremity; TOPAS, Thrombolysis or Peripheral Arterial Surgery; and tPA, tissue plasminogen activator

Evidence Table 48. Nonrandomized Trials, Observational Studies, and/or Registries of Clinical Presentation of ALI–Section 9.2.2.

Study Acronym; Author; Year Published	Study Type/Design; Study Size	Patient Population	Primary Endpoint and Results (include P value; OR or RR; & 95% CI)	Summary/Conclusion Comment(s)
Fagundes C, et al. 2005 (389)<br>17315606	**Study type**: Single institution prospective cohort (observational)<br><br>**Size**: n=83 pts	**Inclusion criteria**: ALI, and etiology<br><br>**Exclusion criteria**: Stage I ischemia	**1° endpoint**: Mortality and amputation<br><br>**Results:**<br>• Male gender, smoking, and comorbidities were more frequent among pts with thrombosis, and atrial fibrillation was more common among pts with embolism<br>• Occlusion longer than 24 h (OR: 2.6; 95% CI: 1.1–7.6) was associated with death and amputation in the multivariate analysis<br>• Mortality 15 (18.1%)<br>• Amputation 24 (28.9%)	• Comorbidities were also more frequent among pts with thrombosis
Rutherford RB, et al.<br>1997 (46)<br>9308598	**Study type**: Consensus document<br><br>**Size**: N/A	**Inclusion criteria**: N/A<br><br>**Exclusion criteria**: N/A	**1° endpoint**: Scoring Scheme for ALI<br><br>**Results**: N/A	N/A
Nypaver TJ, et al. 1998 (375)<br>9737621	**Study type**: Single institution retrospective cohort	**Inclusion criteria**: Acute arterial ischemia and required an urgent/emergent lower-	**1° endpoint**: Outcome of arterial bypass reconstruction in the setting of acute arterial ischemia	N/A

© American Heart Association, Inc. and American College of Cardiology Foundation

	**Size**: n= 71	extremity arterial bypass reconstruction<br><br>**Exclusion criteria**: N/A	**Results:**<br>• Mean duration of symptoms was 43 h (median 24), and mean time from hospital presentation to the operating room was 36 h (median 12)<br>• Death, limb loss, or both, were associated with a paralytic limb (p=0.001) and congestive heart failure (p=0.03)	
Fogarty TJ, et al. 1963 (390) 13945714	**Study type**: Descriptive<br><br>**Size**: N/A	**Inclusion criteria**: N/A<br><br>**Exclusion criteria**: N/A	**1° endpoint**: N/A<br><br>**Results**: N/A	• First description of embolectomy catheter
Shin HS, et al. 2013 (377) 24436594	**Study type**: Single institution<br><br>**Size**: n=18 limbs in 14 consecutive pts	**Inclusion criteria**: All pts with ALI<br><br>**Exclusion criteria**: N/A	**1° endpoint**: Limb salvage via novel surgical approach<br><br>**Results**: N/A	• CTA for Dx<br>• 71% heart disease:<br>57% atrial fibrillation<br>14% had a Hx of previous MI<br>• 86% of pts with mixed thromboembolic disease<br>• Below knee exposure and 1 vessel runoff
Eliason JL and Wakefield TW 2009 (391) 19298933	**Study type**: Review article<br><br>**Size**: n=18 studies	**Inclusion criteria**: N/A<br><br>**Exclusion criteria**: N/A	**1° endpoint**: N/A<br><br>**Results**: N/A	• Compartment pressures are easily measured through multiple methods of pressure transduction<br>• The majority of the lethal events associated with IR injury occur with acute lung injury as a prominent component of the multiple organ dysfunction syndrome
de Donato G, et al. 2014 (378) 24342067	**Study type**: Single institution cohort<br><br>**Size**: n=322 pts	**Inclusion criteria**: All pts w ALI<br><br>**Exclusion criteria**: ALI from graft thrombosis	**1° endpoint:**<br>• In-hospital complications<br>• 30 d mortality<br>• Primary and secondary patency reintervention rate<br>• Limb salvage<br>• Overall survival rates<br><br>**Results**: Reduction in complications when hybrid techniques utilized as opposed to just thromboembolectomy	• Thromboembolectomy alone in 35%<br>• 45.5% via CFA<br>• 30 d mortality 4.4%<br>• 15% in hospital complications<br>8 pts with complication from catheter
Baril DT, et al. 2013 (379) 23714364	**Study type**: Registry review	**Inclusion criteria**: All pts undergoing infrainguinal lower	**1° endpoint**: Major amputation and mortality<br><br>**Results**: ALI predictor of both major amputation	• Age and gender similar to CLI<br>• ALI less likely to be on ASA (63% vs. 75%; p<0.0001) or a statin (55% vs. 68%;

© American Heart Association, Inc. and American College of Cardiology Foundation

	**Size**: n=323 bypass procedures	extremity bypass between 2003 and 2011 (ALI vs. CLI)<br><br>**Exclusion criteria**: N/A	(HR: 2.16; CI: 1.38–3.40; p=0.001) and mortality (HR: 1.41; CI: 1.09–1.83; p=0.009 at 1 y	p<0.0001)<br>• ALI more likely to be current smokers (49%<br>vs. 39%; p<0 .0001), to have had a prior ipsilateral bypass (33% vs. 24%; p=0.004) or a prior ipsilateral percutaneous intervention (41% vs. 29%; p=0.001)
Lurie F, et al.<br>2015 (392)<br>25154566	**Study type**: Multiple institution review<br><br>**Size**: n=1,074 pts	**Inclusion criteria**: Pts treated within 14 d of onset of their symptoms of nonembolic ALI<br><br>**Exclusion criteria**: Elective admission, no therapy	**1° endpoint**: Clinical and technical outcomes, number and type of reinterventions, complications, relief of ischemia, limb salvage, and AFS<br><br>**Results**:<br>• No association between the choice of initial treatment, pt characteristics, location of the occlusion, or the class of ischemia, individually or in combination<br>• Combined endpoint of readmission and AFS was significantly<br>lower in the CDT and CDTA groups	• The cause of ALI was an occluded native vessel in 115 pts (56.1%) and an occluded bypass graft in 90 (43.9%).<br>• Initial treatment resulted in an overall primary success of 67.3%. 60 pts (29.7%) required a second intervention, 11 (5.4%) required a third intervention, 5 (2.4%) required amputation, and 2 (1%) died

ALI indicates acute limb ischemia; AFS, amputation-free survival; ASA, acetylsalicylic Acid; CA, contrast arteriography; CDTA, catheter directed thrombolysis and angioplasty; CDT, catheter directed thrombolysis; CFA, common femoral artery; CI, confidence interval; CLI, critical limb ischemia; CTA, computed tomography angiography; DSA, digital subtraction angiography; DUAM, duplex ultrasound arterial mapping; HR, hazard ratio; MI, myocardial infarction; MRA, magnetic resonance angiography; N/A, not applicable; NEJM, New England Journal of Medicine; NIS, National Inpatient Sample; OR, odds ratio; pt, patient; and RR, relative risk.

Evidence Table 49. Nonrandomized Trials, Observational Studies, and/or Registries of Diagnostic Evaluation of the Cause of ALI–Section 9.2.2.

(There is no literature specifically addressing the diagnostic work up for the cause of ALI. This large single-center series does give etiologies. Echocardiography and telemetry seem reasonable for those without underlying PAD. Focused evaluation for hypercoagulable state seems reasonable in those with native artery thrombosis.)

Study Acronym;<br>Author;<br>Year Published	Study Type/Design;<br>Study Size	Patient Population	Primary Endpoint and Results<br>(include P value; OR or RR;<br>& 95% CI)	Summary/Conclusion<br>Comment(s)
Taha<br>2015 (393)<br>25080883	**Study type**: Single center retrospective review comparing open and endovascular repair in ALI<br><br>**Size**: n=473 pts	**Inclusion criteria**: ALI pts cared for my vascular surgeons. All with embolism or thrombosis as etiology.<br><br>**Exclusion criteria**: Trauma as etiology of ALI, blue toe syndrome	**1° endpoint**: Technical success, incidence of postoperative complications, length of hospital stay, loss of primary patency, loss of assisted primary patency, and loss of secondary patency as well as amputation and mortality rates at 30 d and 1 y	• Underlying cause of ALI retrieved from medical record, cause by percent: cardiac embolism 17.7; native artery thrombosis 26.2; failed stent 17.9; failed bypass graft 33.5; thrombosed peripheral aneurysm 4.7

© American Heart Association, Inc. and American College of Cardiology Foundation

			**Results**: N/A	

ALI indicates acute limb ischemia; N/A, not applicable; PAD, peripheral artery disease; and pt, patient.

Evidence Table 50. Nonrandomized Trials, Observational Studies, and/or Registries of Revascularization Strategy for ALI–Section 9.2.2.

Study Acronym; Author; Year Published	Study Type/Design; Study Size	Patient Population	Primary Endpoint and Results (include P value; OR or RR; & 95% CI)	Summary/Conclusion Comment(s)
Gupta R and Hennebry TA 2012 (394) 22511320	**Study type**: Case series<br><br>**Size**: n=24 pts	**Inclusion criteria**: ALI <14 d treated with Trellis device<br><br>**Exclusion criteria**: Vessel size less than 3 mm diameter or distal location or contrast intolerance, as assessed by the treating clinician's discretion	**1° endpoint**: Limb salvage=100%<br><br>**Results**: In hospital and 30 d mortality=4.16%	• Proof of concept<br>• Level C data
Ansel GM, et al. 2008(395) 18726955	**Study type**: Case series<br><br>**Size**: n=29 limbs treated in 119 pts	**Inclusion criteria**: ALI <14 d treated with pharmaco-mechanical thrombectomy±catheter directed lysis<br><br>**Exclusion criteria**: Pts felt to have possibly experienced a cardio embolic, and evaluated pts with only arterial thrombosis as the inciting event.	**1° endpoint**: Limb salvage<br><br>**Results**: In-hospital success with limb salvage was attained in 96.5% (n=55) with mortality of 3.5% (n=2). 30 d limb salvage and mortality were 94.7% (n=54) and 5.3% (n=3), respectively. At mean 5 y follow-up (mean=62 mo), 3 pts have been lost to follow-up. The results of 54/57 (94.7%) are available. Amputation free survival was 94.7% (n=36/38) with long-term mortality rate of 29.6% (n=16/54).	• Level C data
Byrne RM, et al. 2014 (396) 24360240	**Study type**: Case series<br><br>**Size**: n=154 limbs were treated in 147 pts	**Inclusion criteria**: ALI treated with PMT±CDT<br><br>**Exclusion criteria**: None reported	**1° endpoint**: Technical success was achieved in 83.8% of cases, with a 30 d mortality rate of 5.2%<br><br>**Results**: Overall rate of major amputation was 15.0% (18.1% for CDT only, 11.3% for PMT; p=NS)	• Level C data
Taha AG, et al. 2015 (393) 25080883	**Study type**: Retrospective comparison of endo vs. OR<br><br>**Size**: n=154 limbs were treated in 147 pts in the ER group, compared with 326	**Inclusion criteria**: ALI<br><br>**Exclusion criteria**: Blue toe syndrome and acute ischemia secondary to trauma or dissection were excluded	**1° endpoint**: Amputation and mortality at 1 y<br><br>**Results**:<br>• Overall amputation rates were 13.5% (OR) vs. 6.5% (ER) at 30 d (p=0.023) and 19.6% (OR) vs. 13.0% (ER) at 1 y (p=0.074)	• Equal amputation rates<br>• Endo had lower 30 d mortality<br>• Level C data

© American Heart Association, Inc. and American College of Cardiology Foundation

	limbs in 296 pts in the OR group		• 30 d mortality rate was 13.2% (OR) and 5.4% (ER) (p=0.012)	
Schernthaner MB, et al. 2014 (397) 24933285	**Study type**: Retrospective series; UAT and standard CDT in pts with acute and subacute limb ischemia.<br><br>**Size**: n=UAT was performed in 75 pts, and CDT was performed in 27 pts	**Inclusion criteria**: ALI or subacute limb ischemia<br><br>**Exclusion criteria**: None reported	**1° endpoint**: Limb salvage<br><br>**Results**:<br>• No difference in limb salvage<br>• Major and minor bleeding combined was lower: 6.7% (UAT) vs. 22.2% (CDT) (p=0.025) despite no difference in lytic dose	• Pilot data – level C
Silva JA, et al. 1998 (398) 9863742	**Study type**: Case series<br><br>**Size**: n=21 pts	**Inclusion criteria**: ALI ≤14 d treated with rheolytic thrombectomy<br><br>**Exclusion criteria**: None reported	**1° endpoint**: Limb salvage<br><br>**Results:** The overall 6 mo survival was 81% (17 pts), and limb salvage occurred in 16 of 18 limbs (89%) in the 17 pts	• Proof of concept<br>• Level C data
Kasirajan K, et al. 2001 (399) 11287526	**Study type**: Retrospective analysis<br><br>**Size**: n=86 pts (acute, n=65; subacute, n=21); acute <14 d; suacute 14 d–4 mo	**Inclusion criteria**: ALI (acute or subacute)<br><br>**Exclusion criteria**: None reported	**1° endpoint**: Angiographic success=61.4%<br><br>**Results**: 1 mo amputation and mortality rates were 11.6% and 9.3%	• Level C data<br>• Mixed population
Allie DE, et al. 2004 (400) 15558768	**Study type**: Case series<br><br>**Size**: n=49 pts	**Inclusion criteria**: ALI treated with rheolytic thrombectomy catheter with thrombolytic solution priming agent<br><br>**Exclusion criteria**: None reported	**1° endpoint**: 30 d limb salvage=91%<br><br>**Results**: No significant difference between power pulse with UK or TNK; however no comparator group using catheter directed lytic delivery	• Proof of concept<br>• Level C data
Elmahdy MG, et al. 2010 (401) 20934653	**Study type**: Prospective<br><br>**Size**: n=97 pts	**Inclusion criteria**: Non traumatic ALI<br><br>**Exclusion criteria**: Past Hx of peripheral arterial graft, traumatic limb ischemia, dissection, and thrombosis induced by vasospasm, arteritis, popliteal cyst, or entrapment.	**1° endpoint**: Agreement with surgical determination of embolic or thrombotic<br><br>**Results**:<br>• Clinical characteristics similar in embolic and thrombotic groups<br>• Greater difference in diameter of artery compared with contralateral artery diameter identified embolic etiology	• Duplex provided information on etiology that could guide treatment
Ascher et al. 1999 (402) 12712369	**Study type**: Retrospective, bypass for CLI performed using ultrasound alone or	**Inclusion criteria**: Need for infra inguinal arterial bypass	**1° endpoint**: Adequacy of ultrasound to diagnose stenosis	• Duplex took 100 min angiography required in 2 pts due to arterial

© American Heart Association, Inc. and American College of Cardiology Foundation

	ultrasound + angiography<br><br>**Size**: n=27 pts	**Exclusion criteria**: Contrast allergy	**Results**: Adequate map by ultrasound alone in the majority of pts	calcification<br>• Not clear if any pts had ALI
Lowery AJ, et al. 2007 (403) 17628263	**Study type**: Prospective evaluation of US, MRA, DSA<br><br>**Size**: n=465 pts	**Inclusion criteria**: All pts with CLI being considered for endovascular revascularization<br><br>**Exclusion criteria**: N/A	**1° endpoint**: Compared clinical pragmatism, hemodynamic outcomes, and cost-effectiveness when using DUAM alone compared to DSA or MRA as preoperative assessment<br><br>**Results**: In the DUAM group, 43 lesions were identified and marked at the time of preoperative DUAM, all of which were treated at angioplasty. In the DSA group, 53 lesions identified preoperatively were treated at angioplasty. In the MRA group, 58 lesions were identified as requiring treatment on the preoperative MRA. Only 50 of these required angioplasty.	• US and DSA are reasonable, MRA may have overestimated stenosis<br>• Not clear if any pts had ALI<br>• Similar results from Hingorani and Soule, different from Cambria
Leung DA, et al. 2015 (404) 26109628	**Study type**: Rheolytic thrombectomy registry study<br><br>**Size**: n=283 pts	**Inclusion criteria**: Pts with ALI undergoing treatment with the AngioJet System<br><br>**Exclusion criteria**: N/A	**1° endpoint**: Procedure success, 12-mo amputation free survival, 12-mo freedom from amputation<br><br>**Results**: 83% achieved procedure success. 52% of procedures completed without the need for adjunctive CDT. 12-mo follow-up, 81% amputation free survival and 91% freedom from mortality, 91% freedom from bleeding requiring transfusion, 95% freedom from renal failure. Significantly better outcomes in pts without infrapopliteal involvement and those who underwent PMT without CDT.<br>Higher rates of procedure success (p=0.021), 12-mo amputation free survival (p=0.028), and 12-mo freedom from amputation (p=0.01) in the PMT without CDT group	• PMT had more positive results as a first line treatment for ALI
Schrijver AM, et al. 2012 (405) 21534002	**Study type**: Prospective cohort<br><br>**Size**: n=21 consecutive pts	**Inclusion criteria**: Pts with aotrofemoral arterial thromboembolic obstructions<br><br>**Exclusion criteria**: N/A	**1° endpoint**: 30-d technical and clinical outcome of US-accelerated thrombolysis<br><br>**Results**: Complete thrombolysis (>95% lysis of thrombus) was achieved in 20 pts; in 9 pts within 24 hours. Median ankle-brachial index (ABI) increased from 0.28 (range, 0-0.85) to 0.91	• This feasibility study showed a high technical success rate of US-accelerated thrombolysis for aortofemoral arterial obstructions. US-accelerated thrombolysis

© American Heart Association, Inc. and American College of Cardiology Foundation

			(range, 0.58-1.35). One pt had a thromboembolic complication and needed surgical intervention. No hemorrhagic complications and no deaths occurred. At 30-day follow-up, 17 of 21 pts (81%) had a patent artery or bypass.	led to complete lysis within 24 h in almost half of pts, with a low 30-d major complication rate.
Schrijver A, et al. 2011 (406) 21792154	**Study type:** Retrospective cohort<br><br>**Size:** n=57 pts	**Inclusion criteria:** Pts undergoing US-accelerated thrombolysis for thromboembolic arterial occlusions of the lower extremities<br><br>**Exclusion criteria:** N/A	**1° endpoint:** 30-d and 6-mo follow-up<br><br>**Results:** The 30-day patency rate was 81%, without additional mortality. During a median 6-month (range, 2-14) follow-up, 9 reinterventions were performed. Two pts underwent major amputation and 3 pts died; because of malignancy (N=2) and stroke (N=1).	• Initial success rates of ultrasound-accelerated thrombolysis are high and complication rate is low. However, reintervention rate during short-term follow-up for recurrent ischemia is substantial.

ALI indicates acute limb ischemia; CI, confidence interval; CDT, catheter-directed thrombolysis; CLI, critical limb ischemia; CT, computed tomography; DUAM, duplex ultrasound arterial mapping; DSA, digital-subtraction angiography; ER, endovascular revascularization; HR, hazard ratio; MRA, magnetic resonance angiography; N/A, not applicable; OR, odds ratio; PMT, percutaneous mechanical thrombectomy; P-PS, power-pulse spray; pt, patient; RR, relative risk; RT, rheolytic thrombectomy; TNK, tenecteplase; UAT, ultrasound accelerated thrombolysis; UAT, ultrasound-accelerated thrombolysis; UK, urokinase; and US, ultrasound.

Evidence Table 51. RCTs for Longitudinal Follow-Up–Section 10.

Study Acronym; Author; Year Published	Aim of Study; Study Type; Study Size (N)	Patient Population	Study Intervention (# patients) / Study Comparator (# patients)	Endpoint Results (Absolute Event Rates, P value; OR or RR; & 95% CI)	Relevant 2° Endpoint (if any); Study Limitations; Adverse Events
Ihlberg L, et al. 1999 (407) 10610828	**Aim:** To evaluate benefits of duplex over clinical surveillance with ABI, in preventing vein-graft failure.<br><br>**Study type:** Randomized<br><br>**Size:** n=304 pts (362 infrainguinal bypasses)	**Inclusion criteria:** All primary infrainguinal bypass autogenous vein grafts between 1/91 and 12/95<br><br>**Exclusion criteria:** N/A	**Intervention:** ABI group (183)<br><br>**Comparator:**Duplex group (179)<br><br>Surveillance time points for groups at 1, 3, 6, 9 and 12 mo.	**1° endpoint:**<br>• Primary assisted patency, secondary patency and limb salvage rates were 67%, 74% and 85% for ABI group vs. 67%, 73% and 81% for the Duplex group, respectively. (NS difference)<br>• Similar outcomes at 1y.<br><br>**Safety endpoint:** N/A	Grafts were more often redone in the duplex group.<br><br>**Limitations:** Low power. A large multicenter trial is required
Lundell A, et al. 1995 (408) 7823359	**Aim:**<br>To investigate whether intensive surveillance (Duplex and ABI) improves	**Inclusion criteria:** Pts undergoing reconstruction surgery (CLI, popliteal aneurysm, IC diminishing QoL)	**Intervention:** Intensive surveillance (79)<br><br>**Comparator:** Routine follow up (77)	**1° endpoint:** Assisted primary cumulative vein graft patency rates in the intensive group vs. routine group (78% vs. 53%; p<0.05) and secondary patency rates (82% vs.	• Most of the failing grafts and graft occlusions found in first postop. y.<br>• More failing grafts identified if the intervals

© American Heart Association, Inc. and American College of Cardiology Foundation

	femoropopliteal/crural graft patency as compared to routine follow up.<br><br>**Study type**: Randomized<br><br>**Size**: n=156 pts	**Exclusion criteria**: N/A		56%; p<0.05)<br><br>Assisted primary cumulative ePTFE and composite graft patency in the intensive group vs. the routine group (57% vs. 50%; NS) and secondary patency results were also NS.<br><br>**Safety endpoint**: N/A	between visits was 6 wk for first 6mo

ABI indicates ankle brachial index; CLI, critical limb ischemia; ePTFE, Polytetrafluoroethylene; IC, intermittent claudication; N/A, not applicable; NS, not significant; pt, patient; QoL, quality of life; and RCT, randomized controlled trial;

Evidence Table 52. Nonrandomized Trials, Observational Studies, and/or Registries for Longitudinal Follow-Up–Section 10.

Study Acronym; Author; Year Published	Study Type/Design; Study Size	Patient Population	Primary Endpoint and Results (include P value; OR or RR; & 95% CI)	Summary/Conclusion Comment(s)
Jongsma H, et al. 2016 (409) 26482995	**Study type**: Retrospective cohort study<br><br>**Size**: n=69 pts	**Inclusion criteria:** Pts with primary PTA for autologous infrainguinal bypasses monitored with duplex u/s for 1y<br><br>**Exclusion criteria**: None reported	**1° endpoint**: Number of study interventions<br><br>**Results:**<br>• 43% free of major stenosis/ bypass occlusion<br>• 42% recurrent stenosis<br>• 14% occluded	• Secondary interventions are common however such frequent interventions result in patency rates >80% at 1y
Carter A, et al. 2007 (410) 17980793	**Study type**: Observational<br><br>**Size**: n=212 grafts (197 pts)	**Inclusion criteria**: Infrainguinal lower limb grafts with duplex u/s surveillance (0, 1, 3, 6, 12 and 18 mo)<br><br>**Exclusion criteria**: None reported	**1° endpoint**: Graft failures and time points<br><br>**Results:**<br>• Occlusions-21.6%<br>• Salvage procedure-16% (40.5% done at 6 mo)<br>• 56.6% occlusion preceded by stenosis<br>• Primary occlusions: 95.9% in the prosthetic group and 66.5% in the femorocrural group<br>• Twice as many stenosis in venous conduits than the prosthetic ones	• Surveillance effective for AKV and BKV groups (for detecting the presence of significant lesions at high risk of failure without intervention)<br>• Statins protective against graft failure
Westerband A, et al. 1997 (411) 9061138	**Study type**: Observational<br><br>**Size**: n=98 pts (101 infrainguinal vein grafts)	**Inclusion criteria**: CFDS and ABI every 3 mo for 1 y and every 6 mo thereafter for another y<br><br>**Exclusion criteria**: Lost to follow up pts	**1° endpoint**: No. of evaluations and interventions to prevent graft occlusion after the threshold criteria based on existent literature (HVC defined as PSV >300 cm/sec and Vr >3.5; LVC defined as PSV <45 cm/sec; an ABI decrease >0.15)	• Infrainguinal vein grafts with normal CFDS and ABI are at minimal risk for spontaneous occlusion prospectively validating the threshold criteria.<br>• High risk of bias being an

© American Heart Association, Inc. and American College of Cardiology Foundation

			**Results:**<br>-51 grafts didn't occlude and didn't require revision.<br>-43 had stenosis (20 underwent revision, 2 stenosed, 10 regressed spontaneously, 10 remained stable)	observational validation.
Mills JL, et al.<br>1990 (412)<br>2214034	**Study type:** Observational<br><br>**Size:** n=292 pts (379 reversed vein grafts)	**Inclusion criteria:** Infrainguinal reversed vein bypasses subjects undergoing prospective surveillance protocol<br><br>**Exclusion criteria:** None reported	**1° endpoint and results:**<br>• Mean of 3.2 surveillance exams/ graft with a mean follow up was 21.5 mo.<br>• -2.1% of 280 grafts with GFV >45cm/sec failed within 6 mo of surveillance exam. GFV <45 cm/sec in 99 grafts resulted in arteriography in 75 grafts, identifying 50 stenoses in 48 bypasses.<br>-29% of grafts diagnosed as failing by duplex scans were related to decrease in ABI >0.15.	• Duplex surveillance appeared to be more reliable in the failing grafts than ABI<br>• Dupex surveillance identified graft-threatening lesions in 13% of 379 grafts and repair was successful
Brumberg RS, et al.<br>2007 (413)<br>17920227	**Study type:** Observational<br><br>**Size:** n=121 pts (130 PTFE infrainguinal bypasses)	**Inclusion criteria:** Pts with no usable saphenous veins. Lower limb ischemia (rest pain, tissue loss, disabling claudication/and or popliteal aneurysm, pts requiring a repeat bypass). Duplex surveillance at 1, 4 and 7 mo. and twice yearly afterwards.<br><br>**Exclusion criteria:** Cadaveric vein	**1° endpoint and results:**<br>• 3y primary patency, assisted and secondary patency results were 39%, 43% and 59%, respectively.<br>• NS differences noted between above knee and below knee grafts.<br>• At 3 y, freedom from limb loss was 75% and pt. survival was 75%.<br>• Distal anastomotic adjunct with below knee bypasses reduced graft thrombosis (35% with vs. 60% without) but no patency advantage.<br>• Multivariate analysis: low graft flow (OR: 6.1; 95% CI: 1.9–19.2), use of warfarin (OR: 8.4; 95% CI: 2.1–34.5) and therapeutic warfarin (OR; 24.6%; CI: 5.7–106) to be independent predictors of patency.	• Low graft flow endangered graft patency more frequently than development of duplex scan detected stenoses.<br>• Early duplex scanning more important for diagnosing MGV and the thrombotic potential.
Calligaro KD, et al.<br>2001 (414)<br>11665434	**Study type:** Observational<br><br>**Size:** n= 66 pts (89 infrainguinal bypasses)	**Inclusion criteria:** Infringuinal prosthetic bypasses with Duplex surveillance and entered graft surveillance protocol<br><br>**Exclusion criteria:** No duplex surveillance, inadequate follow up (<3 mo)	**1° endpoint and results:**<br>-22 thrombosed and 25 failing grafts<br>-25 failing grafts were redone.<br>-Sensitivity of duplex correctly identifying failing graft:<br>88% for FT vs. 57% for FP (p = 0.04)<br>-PPV was 95% FT vs. 65% FP (p = 0.04)	• The surveillance and follow up management not shown to be correlated with improved outcomes<br>• Prosthetic grafts more prone to thrombosis.
Stone PA, et al.	**Study type:**	**Inclusion criteria:** Bypasses	**1° endpoint and results:**	Duplex surveillance with repair of

© American Heart Association, Inc. and American College of Cardiology Foundation

2006 (415)<br>16950423	Observational<br><br>**Size:**<br>n=108 pts.<br>(femorofemoral: 100; vein: 8 bypasses)	undergoing Duplex surveillance protocol<br><br>**Exclusion criteria:** None reported	• 29% bypasses were revised<br>• Primary patency at 1, 3 and 5y was 86%, 78% and 62%, respectively.<br>• Duplex assisted-primary patency was 95% at 1 y, 88% at 3 and 5 y (p<0.0001, log rank)<br>• Secondary graft patency was 98% at 1 y, 93% at 3 and 5 y.	lesions with PSVs >300 cm/s improved long term patency of femorofemoral grafts.
Back MR, et al.<br>2001 (416)<br>11797981	**Study type:**<br>Observational<br><br>**Size:** n=64 pts (84 iliac stents)	**Inclusion criteria:**<br>Iliac PTA and stents undergoing aortoiliac duplex surveillance protocol at <1 mo, 3 mo. and 6 mo. intervals for 36 mo.<br><br>**Exclusion criteria:** None reported	**1° endpoints and results:**<br>• 73 patent<br>• 3 occlusions<br>• 2 failing by duplex<br>• 6 re-stented	• Duplex surveillance with iliac stenting localized deteriorating inflow segments, enhanced assisted patency.<br>• Superior efficacy for multilevel occlusive disease and outflow reconstructions.
Baril DT and Marone LK<br>2012 (417)<br>22609972	**Study type:**<br>Observational<br><br>**Size:** n=330 limbs	**Inclusion criteria:** Femoropopliteal angioplasty and stenting pts. undergoing surveillance at 1, 3 and 6 mo. and then at 6 mo. intervals indefinitely after procedure.<br><br>**Exclusion criteria:** None reported	**1° endpoints and results:**<br>• Data pairs of duplex and angiographically measured stenosis within 30 d of each underwent analyses.<br>• Linear regression analyses were performed and ROC curves were used to ascertain optimal criteria associating to ≥50% and ≥80% in-stenosis. A linear regression model of PSV vs. degree of angiographic stenosis (R^2=0.60; p<0.001);<br>(R^2=0.55; p<0.001) for velocity ratio vs. degree of angiographic stenosis showing strong correlation, a moderate adjusted correlation<br>Co-efficient (R^2=0.31; p<0.02) for decrease in ABI vs. degree of angiographic stenosis.	• Applying duplex criteria for both ≥50% and ≥80% in-stent stenosis during follow up may help in preventing endovascular intervention failures.
Troutman DA, et al.<br>2014 (418)<br>25256612	**Study type:**<br>Observational (retrospective)<br><br>**Size:** n=142 stent grafts (92 arterial segments in 79 pts)	**Inclusion criteria:** DU protocol with at least 1 study documenting patent stent graft, at 1wk, every 3 mo for first y and every 6 mo thereafter.<br><br>**Exclusion criteria:** None reported	**1° endpoints and results:**<br>• 15 of 20 pts with ≥1 of abnormal DU findings underwent prophylactic treatment (8) or occluded without treatment (7), whereas only 2 of 72 with normal DU findings occluded (p=0.0001).<br>• Senstivity of DU for total cohort: 58%<br>• Specificity of DU: 97%<br>• NPV: 78%<br>• PPV: 93%	• DU surveillance can predict failure of stent grafts<br>• Statistically reliable markers for predicting stent graft thrombosis: Focal PSVs >300 cm/s, Vr >3.0, and uniform PSVs <50 cm/s throughout the stent graft
Connors G, et al.	**Study type:**	**Inclusion criteria:** Pts with IC	**1° endpoints and results:**	• Long-term primary patency with

© American Heart Association, Inc. and American College of Cardiology Foundation

2011 (419)<br>20853355	Observational<br><br>**Size:** n=142 limbs in 111 consecutive pts	(Rutherford category 3)<br><br>**Exclusion criteria:** Pts with revascularization for CLI	• Compared to lesions <100 mm, longer lesions had higher failed primary patency (100–200 mm; HR: 2.0; p=0.16 vs. >200 mm: HR=2.6; p=0.03)<br>• Short and intermediate lesions had similar failed secondary patency (<5% incidence)<br>• Lesions >200 mm had higher trend in failed secondary patency (HR=4.2; p=0.06)<br>• Compared to lesions >100 mm, higher gain in long-term patency with outpatient surveillance and reintervention for longer lesions and significantly so for intermediate lesions (100–200 mm=23% vs. <100 mm=8%; p=0.041)	percutaneous treatment of femoral artery lesions was lower for long lesions (>100mm).<br>• Outpatient surveillance for restenosis requiring repeat intervention had a greater effect on long-term patency in pts receiving initial treatment for longer femoral artery lesions (>100 mm length).

ABI indicates ankle-brachial index; AKV, above knee venous graft; BKV, below knee venous graft; CFDS, color flow duplex surveillance; CI, confidence interval; CLI, critical limb ischemia; DU, duplex ultrasound; FP, femoropopliteal graft; FT, femorotibial graft; GFV, graft flow velocity; HVC, high-velocity criteria; IC, intermittent claudication; LCV; MGV; NPV, negative predictive value; NS, not significant; OR, odds ratio; PPV, positive predictive value; PSV, peak systolic velocities; PTA, percutaneous transluminal angioplasty; PTFE, polytetrafluoroethylene; pt, patient; PSV; u/s, ultrasound; ROC, receiver operating characteristic;and Vr, velocity ratio.

© American Heart Association, Inc. and American College of Cardiology Foundation

References

1. ROSE GA. The diagnosis of ischaemic heart pain and intermittent claudication in field surveys. Bull World Health Organ. 1962;27:645-58.
2. Leng GC, Fowkes FG. The Edinburgh Claudication Questionnaire: an improved version of the WHO/Rose Questionnaire for use in epidemiological surveys. J Clin Epidemiol. 1992;45:1101-9.
3. Criqui MH, Denenberg JO, Bird CE, et al. The correlation between symptoms and non-invasive test results in patients referred for peripheral arterial disease testing. Vasc Med. 1996;1:65-71.
4. McDermott MM, Mehta S, Greenland P. Exertional leg symptoms other than intermittent claudication are common in peripheral arterial disease. Arch Intern Med. 1999;159:387-92.
5. McDermott MM, Greenland P, Liu K, et al. Leg symptoms in peripheral arterial disease: associated clinical characteristics and functional impairment. JAMA. 2001;286:1599-606.
6. Hirsch AT, Criqui MH, Treat-Jacobson D, et al. Peripheral arterial disease detection, awareness, and treatment in primary care. JAMA. 2001;286:1317-24.
7. Khan NA, Rahim SA, Anand SS, et al. Does the clinical examination predict lower extremity peripheral arterial disease? JAMA. 2006;295:536-46.
8. Grøndal N, Sogaard R, Lindholt JS. Baseline prevalence of abdominal aortic aneurysm, peripheral arterial disease and hypertension in men aged 65-74 years from a population screening study (VIVA trial). Br J Surg. 2015;102:902-6.
9. Wassel CL, Loomba R, Ix JH, et al. Family history of peripheral artery disease is associated with prevalence and severity of peripheral artery disease: the San Diego Population Study. J Am Coll Cardiol. 2011;58:1386-92.
10. Clark CE, Taylor RS, Shore AC, et al. Association of a difference in systolic blood pressure between arms with vascular disease and mortality: a systematic review and meta-analysis. Lancet. 2012;379:905-14.
11. Singh S, Sethi A, Singh M, et al. Simultaneously measured inter-arm and inter-leg systolic blood pressure differences and cardiovascular risk stratification: a systemic review and meta-analysis. J Am Soc Hypertens. 2015;9:640-50.e12.
12. Shadman R, Criqui MH, Bundens WP, et al. Subclavian artery stenosis: prevalence, risk factors, and association with cardiovascular diseases. J Am Coll Cardiol. 2004;44:618-23.
13. Cournot M, Boccalon H, Cambou JP, et al. Accuracy of the screening physical examination to identify subclinical atherosclerosis and peripheral arterial disease in asymptomatic subjects. J Vasc Surg. 2007;46:1215-21.
14. Armstrong DWJ, Tobin C, Matangi MF. The accuracy of the physical examination for the detection of lower extremity peripheral arterial disease. Can J Cardiol. 2010;26:e346-50.
15. Fowkes FG, Price JF, Stewart MC, et al. Aspirin for prevention of cardiovascular events in a general population screened for a low ankle brachial index: a randomized controlled trial. JAMA. 2010;303:841-8.
16. Belch J, MacCuish A, Campbell I, et al. The Prevention of Progression of Arterial Disease and Diabetes (POPADAD) trial: factorial randomised placebo controlled trial of aspirin and antioxidants in patients with diabetes and asymptomatic peripheral arterial disease. BMJ. 2008;337:a1840.
17. McDermott MM, Liu K, Guralnik JM, et al. Home-based walking exercise intervention in peripheral artery disease: a randomized clinical trial. JAMA. 2013;310:57-65.
18. Criqui MH, Vargas V, Denenberg JO, et al. Ethnicity and peripheral arterial disease: the San Diego Population Study. Circulation. 2005;112:2703-7.
19. Selvin E, Erlinger TP. Prevalence of and risk factors for peripheral arterial disease in the United States: results from the National Health and Nutrition Examination Survey, 1999-2000. Circulation. 2004;110:738-43.
20. Guo X, Li J, Pang W, et al. Sensitivity and specificity of ankle-brachial index for detecting angiographic stenosis of peripheral arteries. Circ J. 2008;72:605-10.

© American Heart Association, Inc. and American College of Cardiology Foundation

21. Aboyans V, Criqui MH, Abraham P, et al. Measurement and interpretation of the ankle-brachial index: a scientific statement from the American Heart Association. Circulation. 2012;126:2890-909.
22. Aboyans V, Ho E, Denenberg JO, et al. The association between elevated ankle systolic pressures and peripheral occlusive arterial disease in diabetic and nondiabetic subjects. J Vasc Surg. 2008;48:1197-203.
23. Schröder F, Diehm N, Kareem S, et al. A modified calculation of ankle-brachial pressure index is far more sensitive in the detection of peripheral arterial disease. J Vasc Surg. 2006;44:531-6.
24. Premalatha G, Ravikumar R, Sanjay R, et al. Comparison of colour duplex ultrasound and ankle-brachial pressure index measurements in peripheral vascular disease in type 2 diabetic patients with foot infections. J Assoc Physicians India. 2002;50:1240-4.
25. Allen J, Oates CP, Henderson J, et al. Comparison of lower limb arterial assessments using color-duplex ultrasound and ankle/brachial pressure index measurements. Angiology. 1996;47:225-32.
26. Lijmer JG, Hunink MG, van den Dungen JJ, et al. ROC analysis of noninvasive tests for peripheral arterial disease. Ultrasound Med Biol. 1996;22:391-8.
27. Fowkes FG, Murray GD, Butcher I, et al. Ankle brachial index combined with Framingham Risk Score to predict cardiovascular events and mortality: a meta-analysis. JAMA. 2008;300:197-208.
28. Fowkes FG, Murray GD, Butcher I, et al. Development and validation of an ankle brachial index risk model for the prediction of cardiovascular events. Eur J Prev Cardiol. 2014;21:310-20.
29. Diehm C, Allenberg JR, Pittrow D, et al. Mortality and vascular morbidity in older adults with asymptomatic versus symptomatic peripheral artery disease. Circulation. 2009;120:2053-61.
30. Lin JS, Olson CM, Johnson ES, et al. The ankle-brachial index for peripheral artery disease screening and cardiovascular disease prediction among asymptomatic adults: a systematic evidence review for the U.S. Preventive Services Task Force. Ann Intern med. 2013;333-41.
31. Alahdab F, Wang AT, Elraiyah TA, et al. A systematic review for the screening for peripheral arterial disease in asymptomatic patients. J Vasc Surg. 2015;61:42S-53S.
32. Hiramoto JS, Katz R, Ix JH, et al. Sex differences in the prevalence and clinical outcomes of subclinical peripheral artery disease in the Health, Aging, and Body Composition (Health ABC) study. Vascular. 2014;22:142-8.
33. Bundó M, Muñoz L, Pérez C, et al. Asymptomatic peripheral arterial disease in type 2 diabetes patients: a 10-year follow-up study of the utility of the ankle brachial index as a prognostic marker of cardiovascular disease. Ann Vasc Surg. 2010;24:985-93.
34. Tsivgoulis G, Bogiatzi C, Heliopoulos I, et al. Low ankle-brachial index predicts early risk of recurrent stroke in patients with acute cerebral ischemia. Atherosclerosis. 2012;220:407-12.
35. Bouisset F, Bongard V, Ruidavets JB, et al. Prognostic usefulness of clinical and subclinical peripheral arterial disease in men with stable coronary heart disease. Am J Cardiol. 2012;110:197-202.
36. Sen S, Lynch DR, Kaltsas E, et al. Association of asymptomatic peripheral arterial disease with vascular events in patients with stroke or transient ischemic attack. Stroke. 2009;40:3472-7.
37. Ratanakorn D, Keandoungchun J, Tegeler CH. Prevalence and association between risk factors, stroke subtypes, and abnormal ankle brachial index in acute ischemic stroke. J Stroke Cerebrovasc Dis. 2012;21:498-503.
38. Ramos R, García-Gil M, Comas-Cufí M, et al. Statins for prevention of cardiovascular events in a low-risk population with low ankle brachial index. J Am Coll Cardiol. 2016;67:630-40.
39. Jiménez M, Dorado L, Hernández-Pérez M, et al. Ankle-brachial index in screening for asymptomatic carotid and intracranial atherosclerosis. Atherosclerosis. 2014;233:72-5.
40. McDermott MM, Fried L, Simonsick E, et al. Asymptomatic peripheral arterial disease is independently associated with impaired lower extremity functioning: the Women's Health and Aging Study. Circulation. 2000;101:1007-12.
41. McDermott MM, Liu K, Greenland P, et al. Functional decline in peripheral arterial disease: associations with the ankle brachial index and leg symptoms. JAMA. 2004;292:453-61.

© American Heart Association, Inc. and American College of Cardiology Foundation

42. McDermott MM, Liu K, Ferrucci L, et al. Physical performance in peripheral arterial disease: a slower rate of decline in patients who walk more. Ann Intern med. 2006;144:10-20.
43. McDermott MM, Ferrucci L, Liu K, et al. Leg symptom categories and rates of mobility decline in peripheral arterial disease. J Am Geriatr Soc. 2010;58:1256-62.
44. McDermott MM, Applegate WB, Bonds DE, et al. Ankle brachial index values, leg symptoms, and functional performance among community-dwelling older men and women in the Lifestyle Interventions and Independence for Elders Study. J Am Heart Assoc. 2013;2:e000257.
45. Niazi K, Khan TH, Easley KA. Diagnostic utility of the two methods of ankle brachial index in the detection of peripheral arterial disease of lower extremities. Catheter Cardiovasc Interv. 2006;68:788-92.
46. Rutherford RB, Baker JD, Ernst C, et al. Recommended standards for reports dealing with lower extremity ischemia: revised version. J Vasc Surg. 1997;26:517-38.
47. Eslahpazir BA, Allemang MT, Lakin RO, et al. Pulse volume recording does not enhance segmental pressure readings for peripheral arterial disease stratification. Ann Vasc Surg. 2014;28:18-27.
48. Ouriel K, McDonnell AE, Metz CE, et al. Critical evaluation of stress testing in the diagnosis of peripheral vascular disease. Surgery. 1982;91:686-93.
49. Aerden D, Massaad D, von KK, et al. The ankle--brachial index and the diabetic foot: a troublesome marriage. Ann Vasc Surg. 2011;25:770-7.
50. Park SC, Choi CY, Ha YI, et al. Utility of toe-brachial index for diagnosis of peripheral artery disease. Arch Plast Surg. 2012;39:227-31.
51. Weinberg I, Giri J, Calfon MA, et al. Anatomic correlates of supra-normal ankle brachial indices. Catheter Cardiovasc Interv. 2013;81:1025-30.
52. Suominen V, Rantanen T, Venermo M, et al. Prevalence and risk factors of PAD among patients with elevated ABI. Eur J Vasc Endovasc Surg. 2008;35:709-14.
53. Wagener JS, Hendricker C. Intra-subject variability of noninvasive oxygen measurements. Chest. 1987;92:1047-9.
54. Tsai FW, Tulsyan N, Jones DN, et al. Skin perfusion pressure of the foot is a good substitute for toe pressure in the assessment of limb ischemia. J Vasc Surg. 2000;32:32-6.
55. Yamada T, Ohta T, Ishibashi H, et al. Clinical reliability and utility of skin perfusion pressure measurement in ischemic limbs—comparison with other noninvasive diagnostic methods. J Vasc Surg. 2008;47:318-23.
56. Bosanquet DC, Glasbey JC, Williams IM, et al. Systematic review and meta-analysis of direct versus indirect angiosomal revascularisation of infrapopliteal arteries. Eur J Vasc Endovasc Surg. 2014;48:88-97.
57. Carter SA. Clinical measurement of systolic pressures in limbs with arterial occlusive disease. JAMA. 1969;207:1869-74.
58. Carter SA, Tate RB. Value of toe pulse waves in addition to systolic pressures in the assessment of the severity of peripheral arterial disease and critical limb ischemia. J Vasc Surg. 1996;24:258-65.
59. Ramsey DE, Manke DA, Sumner DS. Toe blood pressure. A valuable adjunct to ankle pressure measurement for assessing peripheral arterial disease. J Cardiovasc Surg (Torino). 1983;24:43-8.
60. Biancari F, Juvonen T. Angiosome-targeted lower limb revascularization for ischemic foot wounds: systematic review and meta-analysis. Eur J Vasc Endovasc Surg. 2014;47:517-22.
61. Vincent DG, Salles-Cunha SX, Bernhard VM, et al. Noninvasive assessment of toe systolic pressures with special reference to diabetes mellitus. J Cardiovasc Surg (Torino). 1983;24:22-8.
62. Mahe G, Pollak AW, Liedl DA, et al. Discordant diagnosis of lower extremity peripheral artery disease using American Heart Association postexercise guidelines. Medicine (Baltimore). 2015;94:e1277.
63. Nicolaï SP, Viechtbauer W, Kruidenier LM, et al. Reliability of treadmill testing in peripheral arterial disease: a meta-regression analysis. J Vasc Surg. 2009;50:322-9.
64. Laing SP, Greenhalgh RM. Standard exercise test to assess peripheral arterial disease. Br Med J. 1980;280:13-6.
65. Raines JK, Darling RC, Buth J, et al. Vascular laboratory criteria for the management of peripheral vascular disease of the lower extremities. Surgery. 1976;79:21-9.

© American Heart Association, Inc. and American College of Cardiology Foundation

66. Sumner DS, Strandness DE. The relationship between calf blood flow and ankle blood pressure in patients with intermittent claudication. Surgery. 1969;65:763-71.
67. Castronuovo JJ, Adera HM, Smiell JM, et al. Skin perfusion pressure measurement is valuable in the diagnosis of critical limb ischemia. J Vasc Surg. 1997;26:629-37.
68. Biotteau E, Mahe G, Rousseau P, et al. Transcutaneous oxygen pressure measurements in diabetic and non-diabetic patients clinically suspected of severe limb ischemia: a matched paired retrospective analysis. Int Angiol. 2009;28:479-83.
69. Bunte MC, Jacob J, Nudelman B, et al. Validation of the relationship between ankle-brachial and toe-brachial indices and infragenicular arterial patency in critical limb ischemia. Vasc Med. 2015;20:23-9.
70. Stein R, Hriljac I, Halperin JL, et al. Limitation of the resting ankle-brachial index in symptomatic patients with peripheral arterial disease. Vasc Med. 2006;11:29-33.
71. Shishehbor MH, Hammad TA, Zeller T, et al. An analysis of IN.PACT DEEP randomized trial on the limitations of the societal guidelines-recommended hemodynamic parameters to diagnose critical limb ischemia. J Vasc Surg. 2016;63:1311-7.
72. Wikström J, Hansen T, Johansson L, et al. Ankle brachial index <0.9 underestimates the prevalence of peripheral artery occlusive disease assessed with whole-body magnetic resonance angiography in the elderly. Acta Radiol. 2008;49:143-9.
73. Wikström J, Hansen T, Johansson L, et al. Lower extremity artery stenosis distribution in an unselected elderly population and its relation to a reduced ankle-brachial index. J Vasc Surg. 2009;50:330-4.
74. Clairotte C, Retout S, Potier L, et al. Automated ankle-brachial pressure index measurement by clinical staff for peripheral arterial disease diagnosis in nondiabetic and diabetic patients. Diabetes Care. 2009;32:1231-6.
75. Burbelko M, Augsten M, Kalinowski MO, et al. Comparison of contrast-enhanced multi-station MR angiography and digital subtraction angiography of the lower extremity arterial disease. J Magn Reson Imaging. 2013;37:1427-35.
76. Shareghi S, Gopal A, Gul K, et al. Diagnostic accuracy of 64 multidetector computed tomographic angiography in peripheral vascular disease. Catheter Cardiovasc Interv. 2010;75:23-31.
77. de Vries SO, Hunink MG, Polak JF. Summary receiver operating characteristic curves as a technique for meta-analysis of the diagnostic performance of duplex ultrasonography in peripheral arterial disease. Acad Radiol. 1996;3:361-9.
78. Ota H, Takase K, Igarashi K, et al. MDCT compared with digital subtraction angiography for assessment of lower extremity arterial occlusive disease: importance of reviewing cross-sectional images. AJR Am J Roentgenol. 2004;182:201-9.
79. He C, Yang JG, Li YM, et al. Comparison of lower extremity atherosclerosis in diabetic and non-diabetic patients using multidetector computed tomography. BMC Cardiovasc Disord. 2014;14:125.
80. Philip F, Shishehbor MH, Desai MY, et al. Characterization of internal pudendal artery atherosclerosis using aortography and multi-detector computed angiography. Catheter Cardiovasc Interv. 2013;82:E516-E521.
81. Kayhan A, Palabiyik F, Serinsöz S, et al. Multidetector CT angiography versus arterial duplex USG in diagnosis of mild lower extremity peripheral arterial disease: is multidetector CT a valuable screening tool? Eur J Radiol. 2012;81:542-6.
82. Joshi SB, Mendoza DD, Steinberg DH, et al. Ultra-low-dose intra-arterial contrast injection for iliofemoral computed tomographic angiography. JACC Cardiovasc Imaging. 2009;2:1404-11.
83. Mesurolle B, Qanadli SD, El HM, et al. Occlusive arterial disease of abdominal aorta and lower extremities: comparison of helical CT angiography with transcatheter angiography. Clin Imaging. 2004;28:252-60.
84. Romano M, Mainenti PP, Imbriaco M, et al. Multidetector row CT angiography of the abdominal aorta and lower extremities in patients with peripheral arterial occlusive disease: diagnostic accuracy and interobserver agreement. Eur J Radiol. 2004;50:303-8.
85. Martin ML, Tay KH, Flak B, et al. Multidetector CT angiography of the aortoiliac system and lower extremities: a prospective comparison with digital subtraction angiography. AJR Am J Roentgenol. 2003;180:1085-91.

© American Heart Association, Inc. and American College of Cardiology Foundation

86. Andreucci M, Solomon R, Tasanarong A. Side effects of radiographic contrast media: pathogenesis, risk factors, and prevention. Biomed Res Int. 2014;2014:741018.
87. Stacul F, van der Molen AJ, Reimer P, et al. Contrast induced nephropathy: updated ESUR Contrast Media Safety Committee guidelines. Eur Radiol. 2011;21:2527-41.
88. Meyer BC, Klein S, Krix M, et al. Comparison of a standard and a high-concentration contrast medium protocol for MDCT angiography of the lower limb arteries. Rofo. 2012;184:527-34.
89. Fraioli F, Catalano C, Napoli A, et al. Low-dose multidetector-row CT angiography of the infra-renal aorta and lower extremity vessels: image quality and diagnostic accuracy in comparison with standard DSA. Eur Radiol. 2006;16:137-46.
90. Met R, Bipat S, Legemate DA, et al. Diagnostic performance of computed tomography angiography in peripheral arterial disease: a systematic review and meta-analysis. JAMA. 2009;301:415-24.
91. Favaretto E, Pili C, Amato A, et al. Analysis of agreement between Duplex ultrasound scanning and arteriography in patients with lower limb artery disease. J Cardiovasc Med (Hagerstown). 2007;8:337-41.
92. Kau T, Eicher W, Reiterer C, et al. Dual-energy CT angiography in peripheral arterial occlusive disease-accuracy of maximum intensity projections in clinical routine and subgroup analysis. Eur Radiol. 2011;21:1677-86.
93. McCullough PA, Capasso P. Patient discomfort associated with the use of intra-arterial iodinated contrast media: a meta-analysis of comparative randomized controlled trials. BMC Med Imaging. 2011;11:12.
94. Sultan S, Chua BY, Hamada N, et al. Preoperative vascular screening in the presence of aortic, carotid and peripheral pathology for patients undergoing their first arterial intervention: 18 month follow-up. Int Angiol. 2013;32:281-90.
95. Kurvers HA, van der Graaf Y, Blankensteijn JD, et al. Screening for asymptomatic internal carotid artery stenosis and aneurysm of the abdominal aorta: comparing the yield between patients with manifest atherosclerosis and patients with risk factors for atherosclerosis only. J Vasc Surg. 2003;37:1226-33.
96. Giugliano G, Laurenzano E, Rengo C, et al. Abdominal aortic aneurysm in patients affected by intermittent claudication: prevalence and clinical predictors. BMC Surg. 2012;12(suppl 1):S17.
97. Barba A, Estallo L, Rodríguez L, et al. Detection of abdominal aortic aneurysm in patients with peripheral artery disease. Eur J Vasc Endovasc Surg. 2005;30:504-8.
98. Lee JY, Lee SW, Lee WS, et al. Prevalence and clinical implications of newly revealed, asymptomatic abnormal ankle-brachial index in patients with significant coronary artery disease. JACC Cardiovasc Interv. 2013;6:1303-13.
99. Moyer VA. Screening for peripheral artery disease and cardiovascular disease risk assessment with the ankle-brachial index in adults: U.S. Preventive Services Task Force recommendation statement. Ann Intern Med. 2013;159:342-8.
100. McFalls EO, Ward HB, Moritz TE, et al. Coronary-artery revascularization before elective major vascular surgery. N Engl J Med. 2004;351:2795-804.
101. Olin JW, Melia M, Young JR, et al. Prevalence of atherosclerotic renal artery stenosis in patients with atherosclerosis elsewhere. Am J Med. 1990;88:46N-51N.
102. Leertouwer TC, Pattynama PM, van den Berg-Huysmans A. Incidental renal artery stenosis in peripheral vascular disease: a case for treatment? Kidney Int. 2001;59:1480-3.
103. Hansen KJ, Edwards MS, Craven TE, et al. Prevalence of renovascular disease in the elderly: a population-based study. J Vasc Surg. 2002;36:443-51.
104. Catalano M, Born G, Peto R. Prevention of serious vascular events by aspirin amongst patients with peripheral arterial disease: randomized, double-blind trial. J Intern Med. 2007;261:276-84.
105. Horrocks M, Horrocks EH, Murphy P, et al. The effects of platelet inhibitors on platelet uptake and restenosis after femoral angioplasty. Int Angiol. 1997;16:101-6.
106. Minar E, Ahmadi A, Koppensteiner R, et al. Comparison of effects of high-dose and low-dose aspirin on restenosis after femoropopliteal percutaneous transluminal angioplasty. Circulation. 1995;91:2167-73.
107. CAPRIE Steering Committee. A randomised, blinded, trial of clopidogrel versus aspirin in patients at risk of ischaemic events (CAPRIE). Lancet. 1996;348:1329-39.

© American Heart Association, Inc. and American College of Cardiology Foundation

108. Cacoub PP, Bhatt DL, Steg PG, et al. Patients with peripheral arterial disease in the CHARISMA trial. Eur Heart J. 2009;30:192-201.
109. Bhatt DL, Flather MD, Hacke W, et al. Patients with prior myocardial infarction, stroke, or symptomatic peripheral arterial disease in the CHARISMA trial. J Am Coll Cardiol. 2007;49:1982-8.
110. Berger PB, Bhatt DL, Fuster V, et al. Bleeding complications with dual antiplatelet therapy among patients with stable vascular disease or risk factors for vascular disease: results from the Clopidogrel for High Atherothrombotic Risk and Ischemic Stabilization, Management, and Avoidance (CHARISMA) trial. Circulation. 2010;121:2575-83.
111. Cassar K, Ford I, Greaves M, et al. Randomized clinical trial of the antiplatelet effects of aspirin-clopidogrel combination versus aspirin alone after lower limb angioplasty. Br J Surg. 2005;92:159-65.
112. Belch JJ, Dormandy J, CASPAR Writing CommitteeBiasi GM, et al. Results of the randomized, placebo-controlled Clopidogrel and Acetylsalicylic Acid in Bypass Surgery for Peripheral Arterial Disease (CASPAR) trial. J Vasc Surg. 2010;52:825-33.
113. Tepe G, Bantleon R, Brechtel K, et al. Management of peripheral arterial interventions with mono or dual antiplatelet therapy-the MIRROR study: a randomised and double-blinded clinical trial. Eur Radiol. 2012;22:1998-2006.
114. Bonaca MP, Scirica BM, Creager MA, et al. Vorapaxar in patients with peripheral artery disease: results from TRA2°P-TIMI 50. Circulation. 2013;127:1522-9.
115. Strobl FF, Brechtel K, Schmehl J, et al. Twelve-month results of a randomized trial comparing mono with dual antiplatelet therapy in endovascularly treated patients with peripheral artery disease. J Endovasc Ther. 2013;20:699-706.
116. Collaborative overview of randomised trials of antiplatelet therapy--II: Maintenance of vascular graft or arterial patency by antiplatelet therapy. Antiplatelet Trialists' Collaboration. BMJ. 1994;308:159-68.
117. Antithrombotic Trialists' Collaboration. Collaborative meta-analysis of randomised trials of antiplatelet therapy for prevention of death, myocardial infarction, and stroke in high risk patients. BMJ. 2002;324:71-86.
118. Morrow DA, Braunwald E, Bonaca MP, et al. Vorapaxar in the secondary prevention of atherothrombotic events. N Engl J Med. 2012;366:1404-13.
119. Bohula EA, Aylward PE, Bonaca MP, et al. Efficacy and safety of vorapaxar with and without a thienopyridine for secondary prevention in patients with previous myocardial infarction and no history of stroke or transient ischemic attack: results from TRA 2°P-TIMI 50. Circulation. 2015;132:1871-9.
120. Bonaca MP, Gutierrez JA, Creager MA, et al. Acute limb ischemia and outcomes with vorapaxar in patients with peripheral artery disease: results from the Trial to Assess the Effects of Vorapaxar in Preventing Heart Attack and Stroke in patients With Atherosclerosis-Thrombolysis in Myocardial Infarction 50 (TRA2°P-TIMI 50). Circulation. 2016;997-1005.
121. Jones WS, Dolor RJ, Hasselblad V, et al. Comparative effectiveness of endovascular and surgical revascularization for patients with peripheral artery disease and critical limb ischemia: systematic review of revascularization in critical limb ischemia. Am Heart J. 2014;167:489-98.e7.
122. Katsanos K, Spiliopoulos S, Saha P, et al. Comparative Efficacy and Safety of Different Antiplatelet Agents for Prevention of Major Cardiovascular Events and Leg Amputations in Patients with Peripheral Arterial Disease: A Systematic Review and Network Meta-Analysis. PLoS One. 2015;10:e0135692.
123. Magnani G, Bonaca MP, Braunwald E, et al. Efficacy and safety of vorapaxar as approved for clinical use in the United States. J Am Heart Assoc. 2015;4:e001505.
124. Berger JS, Krantz MJ, Kittelson JM, et al. Aspirin for the prevention of cardiovascular events in patients with peripheral artery disease: a meta-analysis of randomized trials. JAMA. 2009;301:1909-19.
125. Armstrong EJ, Anderson DR, Yeo KK, et al. Association of dual-antiplatelet therapy with reduced major adverse cardiovascular events in patients with symptomatic peripheral arterial disease. J Vasc Surg. 2015;62:157-65.
126. Heart Protection Study Collaborative Group. Randomized trial of the effects of cholesterol-lowering with simvastatin on peripheral vascular and other major vascular outcomes in 20,536 people with peripheral arterial disease and other high-risk conditions. J Vasc Surg. 2007;45:645-54.
127. Mohler ER3, Hiatt WR, Creager MA. Cholesterol reduction with atorvastatin improves walking distance in patients with peripheral arterial disease. Circulation. 2003;108:1481-6.
128. Hiatt WR, Hirsch AT, Creager MA, et al. Effect of niacin ER/lovastatin on claudication symptoms in patients with peripheral artery disease. Vasc Med. 2010;15:171-9.

© American Heart Association, Inc. and American College of Cardiology Foundation

129. Giri J, McDermott MM, Greenland P, et al. Statin use and functional decline in patients with and without peripheral arterial disease. J Am Coll Cardiol. 2006;47:998-1004.
130. West AM, Anderson JD, Meyer CH, et al. The effect of ezetimibe on peripheral arterial atherosclerosis depends upon statin use at baseline. Atherosclerosis. 2011;218:156-62.
131. Stoekenbroek RM, Boekholdt SM, Fayyad R, et al. High-dose atorvastatin is superior to moderate-dose simvastatin in preventing peripheral arterial disease. Heart. 2015;101:356-62.
132. Aung PP, Maxwell HG, Jepson RG, et al. Lipid-lowering for peripheral arterial disease of the lower limb. Cochrane Database Syst Rev. 2007;CD000123.
133. Kumbhani DJ, Steg PG, Cannon CP, et al. Statin therapy and long-term adverse limb outcomes in patients with peripheral artery disease: insights from the REACH registry. Eur Heart J. 2014;35:2864-72.
134. Vogel TR, Dombrovskiy VY, Galiñanes EL, et al. Preoperative statins and limb salvage after lower extremity revascularization in the Medicare population. Circ Cardiovasc Interv. 2013;6:694-700.
135. Westin GG, Armstrong EJ, Bang H, et al. Association between statin medications and mortality, major adverse cardiovascular event, and amputation-free survival in patients with critical limb ischemia. J Am Coll Cardiol. 2014;63:682-90.
136. Feringa HH, Karagiannis SE, van Waning VH, et al. The effect of intensified lipid-lowering therapy on long-term prognosis in patients with peripheral arterial disease. J Vasc Surg. 2007;45:936-43.
137. Ostergren J, Sleight P, Dagenais G, et al. Impact of ramipril in patients with evidence of clinical or subclinical peripheral arterial disease. Eur Heart J. 2004;25:17-24.
138. Yusuf S, Sleight P, Pogue J, et al. Effects of an angiotensin-converting-enzyme inhibitor, ramipril, on cardiovascular events in high-risk patients. The Heart Outcomes Prevention Evaluation Study Investigators. N Engl J Med. 2000;342:145-53.
139. Yusuf S, Teo KK, Pogue J, et al. Telmisartan, ramipril, or both in patients at high risk for vascular events. N Engl J Med. 2008;358:1547-59.
140. Bavry AA, Anderson RD, Gong Y, et al. Outcomes Among hypertensive patients with concomitant peripheral and coronary artery disease: findings from the INternational VErapamil-SR/Trandolapril STudy. Hypertension. 2010;55:48-53.
141. Zanchetti A, Julius S, Kjeldsen S, et al. Outcomes in subgroups of hypertensive patients treated with regimens based on valsartan and amlodipine: an analysis of findings from the VALUE trial. J Hypertens. 2006;24:2163-8.
142. Diehm C, Pittrow D, Lawall H. Effect of nebivolol vs. hydrochlorothiazide on the walking capacity in hypertensive patients with intermittent claudication. J Hypertens. 2011;29:1448-56.
143. Espinola-Klein C, Weisser G, Jagodzinski A, et al. b-Blockers in patients with intermittent claudication and arterial hypertension: results from the nebivolol or metoprolol in arterial occlusive disease trial. Hypertension. 2011;58:148-54.
144. Paravastu SC, Mendonca DA, Da Silva A. Beta blockers for peripheral arterial disease. Cochrane Database Syst Rev. 2013;CD005508.
145. ALLHAT Officers and Coordinators for the ALLHAT Collaborative Research Group. Major outcomes in high-risk hypertensive patients randomized to angiotensin-converting enzyme inhibitor or calcium channel blocker vs diuretic: The Antihypertensive and Lipid-Lowering Treatment to Prevent Heart Attack Trial (ALLHAT). JAMA. 2002;288:2981-97.
146. Feringa HH, van Waning VH, Bax JJ, et al. Cardioprotective medication is associated with improved survival in patients with peripheral arterial disease. J Am Coll Cardiol. 2006;47:1182-7.
147. Sleight P. The HOPE Study (Heart Outcomes Prevention Evaluation). J Renin Angiotensin Aldosterone Syst. 2000;1:18-20.
148. Rigotti NA, Regan S, Levy DE, et al. Sustained care intervention and postdischarge smoking cessation among hospitalized adults: a randomized clinical trial. JAMA. 2014;312:719-28.
149. Rigotti NA, Pipe AL, Benowitz NL, et al. Efficacy and safety of varenicline for smoking cessation in patients with cardiovascular disease: a randomized trial. Circulation. 2010;121:221-9.
150. Hennrikus D, Joseph AM, Lando HA, et al. Effectiveness of a smoking cessation program for peripheral artery disease patients: a randomized controlled trial. J Am Coll Cardiol. 2010;56:2105-12.

© American Heart Association, Inc. and American College of Cardiology Foundation

151. Tonstad S, Farsang C, Klaene G, et al. Bupropion SR for smoking cessation in smokers with cardiovascular disease: a multicentre, randomised study. Eur Heart J. 2003;24:946-55.
152. Stead LF, Buitrago D, Preciado N, et al. Physician advice for smoking cessation. Cochrane Database Syst Rev. 2013;CD000165.
153. Prochaska JJ, Hilton JF. Risk of cardiovascular serious adverse events associated with varenicline use for tobacco cessation: systematic review and meta-analysis. BMJ. 2012;344:e2856.
154. Mills EJ, Thorlund K, Eapen S, et al. Cardiovascular events associated with smoking cessation pharmacotherapies: a network meta-analysis. Circulation. 2014;129:28-41.
155. Clair C, Rigotti NA, Porneala B, et al. Association of smoking cessation and weight change with cardiovascular disease among adults with and without diabetes. JAMA. 2013;309:1014-21.
156. Hoel AW, Nolan BW, Goodney PP, et al. Variation in smoking cessation after vascular operations. J Vasc Surg. 2013;57:1338-44.
157. Selvarajah S, Black JH3, Malas MB, et al. Preoperative smoking is associated with early graft failure after infrainguinal bypass surgery. J Vasc Surg. 2014;59:1308-14.
158. Armstrong EJ, Wu J, Singh GD, et al. Smoking cessation is associated with decreased mortality and improved amputation-free survival among patients with symptomatic peripheral artery disease. J Vasc Surg. 2014;60:1565-71.
159. Lu L, Mackay DF, Pell JP. Association between level of exposure to secondhand smoke and peripheral arterial disease: cross-sectional study of 5,686 never smokers. Atherosclerosis. 2013;229:273-6.
160. Tan CE, Glantz SA. Association between smoke-free legislation and hospitalizations for cardiac, cerebrovascular, and respiratory diseases: a meta-analysis. Circulation. 2012;126:2177-83.
161. Dormandy JA, Charbonnel B, Eckland DJ, et al. Secondary prevention of macrovascular events in patients with type 2 diabetes in the PROactive Study (PROspective pioglitAzone Clinical Trial In macroVascular Events): a randomised controlled trial. Lancet. 2005;366:1279-89.
162. Singh S, Armstrong EJ, Sherif W, et al. Association of elevated fasting glucose with lower patency and increased major adverse limb events among patients with diabetes undergoing infrapopliteal balloon angioplasty. Vasc Med. 2014;19:307-14.
163. Takahara M, Kaneto H, Iida O, et al. The influence of glycemic control on the prognosis of Japanese patients undergoing percutaneous transluminal angioplasty for critical limb ischemia. Diabetes Care. 2010;33:2538-42.
164. Resnick HE, Lindsay RS, McDermott MM, et al. Relationship of high and low ankle brachial index to all-cause and cardiovascular disease mortality: the Strong Heart Study. Circulation. 2004;109:733-9.
165. Anand S, Yusuf S, Xie C, et al. Oral anticoagulant and antiplatelet therapy and peripheral arterial disease. N Engl J Med. 2007;357:217-27.
166. Efficacy of oral anticoagulants compared with aspirin after infrainguinal bypass surgery (The Dutch Bypass Oral Anticoagulants or Aspirin Study): a randomised trial. Lancet. 2000;355:346-51.
167. Johnson WC, Williford WO, Department of Veterans Affairs Cooporative Study #362. Benefits, morbidity, and mortality associated with long-term administration of oral anticoagulant therapy to patients with peripheral arterial bypass procedures: a prospective randomized study. J Vasc Surg. 2002;35:413-21.
168. Sarac TP, Huber TS, Back MR, et al. Warfarin improves the outcome of infrainguinal vein bypass grafting at high risk for failure. J Vasc Surg. 1998;28:446-57.
169. Antonicelli R, Sardina M, Scotti A, et al. Randomized trial of the effects of low-dose calcium-heparin in patients with peripheral arterial disease and claudication. Italian CAP Study Group. Am J Med. 1999;107:234-9.
170. Alonso-Coello P, Bellmunt S, McGorrian C, et al. Antithrombotic therapy in peripheral artery disease: Antithrombotic Therapy and Prevention of Thrombosis, 9th ed: American College of Chest Physicians Evidence-Based Clinical Practice Guidelines. Chest. 2012;141:e669S-90S.
171. Bedenis R, Lethaby A, Maxwell H, et al. Antiplatelet agents for preventing thrombosis after peripheral arterial bypass surgery. Cochrane Database Syst Rev. 2015;CD000535.

© American Heart Association, Inc. and American College of Cardiology Foundation

172. Cosmi B, Conti E, Coccheri S. Anticoagulants (heparin, low molecular weight heparin and oral anticoagulants) for intermittent claudication. Cochrane Database Syst Rev. 2001;CD001999.
173. Bedenis R, Stewart M, Cleanthis M, et al. Cilostazol for intermittent claudication. Cochrane Database Syst Rev. 2014;CD003748.
174. Dawson DL, Cutler BS, Hiatt WR, et al. A comparison of cilostazol and pentoxifylline for treating intermittent claudication. Am J Med. 2000;109:523-30.
175. Goldenberg NA, Krantz MJ, Hiatt WR. L-Carnitine plus cilostazol versus cilostazol alone for the treatment of claudication in patients with peripheral artery disease: a multicenter, randomized, double-blind, placebo-controlled trial. Vasc Med. 2012;17:145-54.
176. Warner CJ, Greaves SW, Larson RJ, et al. Cilostazol is associated with improved outcomes after peripheral endovascular interventions. J Vasc Surg. 2014;59:1607-14.
177. Iida O, Yokoi H, Soga Y, et al. Cilostazol reduces angiographic restenosis after endovascular therapy for femoropopliteal lesions in the Sufficient Treatment of Peripheral Intervention by Cilostazol study. Circulation. 2013;127:2307-15.
178. Salhiyyah K, Senanayake E, Abdel-Hadi M, et al. Pentoxifylline for intermittent claudication. Cochrane Database Syst Rev. 2012;1:CD005262.
179. Villarruz MV, Dans A, Tan F. Chelation therapy for atherosclerotic cardiovascular disease. Cochrane Database Syst Rev. 2002;CD002785.
180. Khandanpour N, Loke YK, Meyer FJ, et al. Homocysteine and peripheral arterial disease: systematic review and meta-analysis. Eur J Vasc Endovasc Surg. 2009;38:316-22.
181. Lonn E, Yusuf S, Arnold MJ, et al. Homocysteine lowering with folic acid and B vitamins in vascular disease. N Engl J Med. 2006;354:1567-77.
182. Lonn E, Held C, Arnold JM, et al. Rationale, design and baseline characteristics of a large, simple, randomized trial of combined folic acid and vitamins B6 and B12 in high-risk patients: the Heart Outcomes Prevention Evaluation (HOPE)-2 trial. Can J Cardiol. 2006;22:47-53.
183. Gurfinkel EP, Leo de la Fuente R, Mendiz O, et al. Flu vaccination in acute coronary syndromes and planned percutaneous coronary interventions (FLUVACS) Study. Eur Heart J. 2004;25:25-31.
184. Ciszewski A, Bilinska ZT, Brydak LB, et al. Influenza vaccination in secondary prevention from coronary ischaemic events in coronary artery disease: FLUCAD study. Eur Heart J. 2008;29:1350-8.
185. Davis MM, Taubert K, Benin AL, et al. Influenza vaccination as secondary prevention for cardiovascular disease: a science advisory from the American Heart Association/American College of Cardiology. J Am Coll Cardiol. 2006;48:1498-502.
186. Murphy TP, Cutlip DE, Regensteiner JG, et al. Supervised exercise, stent revascularization, or medical therapy for claudication due to aortoiliac peripheral artery disease: the CLEVER study. J Am Coll Cardiol. 2015;65:999-1009.
187. Murphy TP, Cutlip DE, Regensteiner JG, et al. Supervised exercise versus primary stenting for claudication resulting from aortoiliac peripheral artery disease: six-month outcomes from the claudication: exercise versus endoluminal revascularization (CLEVER) study. Circulation. 2012;125:130-9.
188. McDermott MM, Guralnik JM, Criqui MH, et al. Home-based walking exercise in peripheral artery disease: 12-month follow-up of the GOALS randomized trial. J Am Heart Assoc. 2014;3:e000711.
189. Collins TC, Lunos S, Carlson T, et al. Effects of a home-based walking intervention on mobility and quality of life in people with diabetes and peripheral arterial disease: a randomized controlled trial. Diabetes Care. 2011;34:2174-9.
190. Gardner AW, Parker DE, Montgomery PS, et al. Efficacy of quantified home-based exercise and supervised exercise in patients with intermittent claudication: a randomized controlled trial. Circulation. 2011;123:491-8.
191. Saxton JM, Zwierska I, Blagojevic M, et al. Upper- versus lower-limb aerobic exercise training on health-related quality of life in patients with symptomatic peripheral arterial disease. J Vasc Surg. 2011;53:1265-73.
192. Treat-Jacobson D, Bronas UG, Leon AS. Efficacy of arm-ergometry versus treadmill exercise training to improve walking distance in patients with claudication. Vasc Med. 2009;14:203-13.
193. Mika P, Konik A, Januszek R, et al. Comparison of two treadmill training programs on walking ability and endothelial function in intermittent claudication. Int J Cardiol. 2013;168:838-42.
194. Fakhry F, Rouwet EV, den Hoed PT, et al. Long-term clinical effectiveness of supervised exercise therapy versus endovascular revascularization for intermittent claudication from a randomized clinical trial. Br J Surg. 2013;100:1164-71.

© American Heart Association, Inc. and American College of Cardiology Foundation

195. Mazari FA, Gulati S, Rahman MN, et al. Early outcomes from a randomized, controlled trial of supervised exercise, angioplasty, and combined therapy in intermittent claudication. Ann Vasc Surg. 2010;24:69-79.
196. Fakhry F, Spronk S, van der Laan L, et al. Endovascular revascularization and supervised exercise for peripheral artery disease and intermittent claudication: a randomized clinical trial. JAMA. 2015;314:1936-44.
197. Guidon M, McGee H. One-year effect of a supervised exercise programme on functional capacity and quality of life in peripheral arterial disease. Disabil Rehabil. 2013;35:397-404.
198. Gardner AW, Parker DE, Montgomery PS, et al. Step-monitored home exercise improves ambulation, vascular function, and inflammation in symptomatic patients with peripheral artery disease: a randomized controlled trial. J Am Heart Assoc. 2014;3:e001107.
199. Langbein WE, Collins EG, Orebaugh C, et al. Increasing exercise tolerance of persons limited by claudication pain using polestriding. J Vasc Surg. 2002;35:887-93.
200. Walker RD, Nawaz S, Wilkinson CH, et al. Influence of upper- and lower-limb exercise training on cardiovascular function and walking distances in patients with intermittent claudication. J Vasc Surg. 2000;31:662-9.
201. Pilz M, Kandioler-Honetz E, Wenkstetten-Holub A, et al. Evaluation of 6- and 12-month supervised exercise training on strength and endurance parameters in patients with peripheral arterial disease. Wien Klin Wochenschr. 2014;126:383-9.
202. Mays RJ, Rogers RK, Hiatt WR, et al. Community walking programs for treatment of peripheral artery disease. J Vasc Surg. 2013;58:1678-87.
203. Crane M, Werber B. Critical pathway approach to diabetic pedal infections in a multidisciplinary setting. J Foot Ankle Surg. 1999;38:30-3.
204. Larsson J, Apelqvist J, Agardh CD, et al. Decreasing incidence of major amputation in diabetic patients: a consequence of a multidisciplinary foot care team approach? Diabet Med. 1995;12:770-6.
205. Armstrong DG, Bharara M, White M, et al. The impact and outcomes of establishing an integrated interdisciplinary surgical team to care for the diabetic foot. Diabetes Metab Res Rev. 2012;28:514-8.
206. Chung J, Modrall JG, Ahn C, et al. Multidisciplinary care improves amputation-free survival in patients with chronic critical limb ischemia. J Vasc Surg. 2015;61:162-9.
207. Canavan RJ, Unwin NC, Kelly WF, et al. Diabetes- and nondiabetes-related lower extremity amputation incidence before and after the introduction of better organized diabetes foot care: continuous longitudinal monitoring using a standard method. Diabetes Care. 2008;31:459-63.
208. Williams DT, Majeed MU, Shingler G, et al. A diabetic foot service established by a department of vascular surgery: an observational study. Ann Vasc Surg. 2012;26:700-6.
209. Driver VR, Madsen J, Goodman RA. Reducing amputation rates in patients with diabetes at a military medical center: the limb preservation service model. Diabetes Care. 2005;28:248-53.
210. Wrobel JS, Charns MP, Diehr P, et al. The relationship between provider coordination and diabetes-related foot outcomes. Diabetes Care. 2003;26:3042-7.
211. Vartanian SM, Robinson KD, Ofili K, et al. Outcomes of neuroischemic wounds treated by a multidisciplinary amputation prevention service. Ann Vasc Surg. 2015;29:534-42.
212. Gardner SE, Hillis SL, Frantz RA. Clinical signs of infection in diabetic foot ulcers with high microbial load. Biol Res Nurs. 2009;11:119-28.
213. Lipsky BA, Berendt AR, Cornia PB, et al. 2012 Infectious Diseases Society of America clinical practice guideline for the diagnosis and treatment of diabetic foot infections. Clin Infect Dis. 2012;54:e132-73.
214. Pickwell K, Siersma V, Kars M, et al. Predictors of lower-extremity amputation in patients with an infected diabetic foot ulcer. Diabetes Care. 2015;38:852-7.
215. Dinh MT, Abad CL, Safdar N. Diagnostic accuracy of the physical examination and imaging tests for osteomyelitis underlying diabetic foot ulcers: meta-analysis. Clin Infect Dis. 2008;47:519-27.
216. Prompers L, Schaper N, Apelqvist J, et al. Prediction of outcome in individuals with diabetic foot ulcers: focus on the differences between individuals with and without peripheral arterial disease. The EURODIALE Study. Diabetologia. 2008;51:747-55.
217. Rogers LC, Andros G, Caporusso J, et al. Toe and flow: essential components and structure of the amputation prevention team. J Vasc Surg. 2010;52:23S-7S.

© American Heart Association, Inc. and American College of Cardiology Foundation

218. Sumpio BE, Armstrong DG, Lavery LA, et al. The role of interdisciplinary team approach in the management of the diabetic foot: a joint statement from the Society for Vascular Surgery and the American Podiatric Medical Association. J Vasc Surg. 2010;51:1504-6.
219. Fitzgerald RH, Mills JL, Joseph W, et al. The diabetic rapid response acute foot team: 7 essential skills for targeted limb salvage. Eplasty. 2009;9:e15.
220. Wrobel JS, Robbins JM, Charns MP, et al. Diabetes-related foot care at 10 Veterans Affairs medical centers: must do's associated with successful microsystems. Jt Comm J Qual Patient Saf. 2006;32:206-13.
221. Tetteroo E, van der Graaf Y, Bosch JL, et al. Randomised comparison of primary stent placement versus primary angioplasty followed by selective stent placement in patients with iliac-artery occlusive disease. Dutch Iliac Stent Trial Study Group. Lancet. 1998;351:1153-9.
222. Klein WM, van der Graaf Y, Seegers J, et al. Long-term cardiovascular morbidity, mortality, and reintervention after endovascular treatment in patients with iliac artery disease: The Dutch Iliac Stent Trial Study. Radiology. 2004;232:491-8.
223. Bosch JL, Hunink MG. Meta-analysis of the results of percutaneous transluminal angioplasty and stent placement for aortoiliac occlusive disease. Radiology. 1997;204:87-96.
224. Kashyap VS, Pavkov ML, Bena JF, et al. The management of severe aortoiliac occlusive disease: endovascular therapy rivals open reconstruction. J Vasc Surg. 2008;48:1451-7, 1457.
225. Schillinger M, Sabeti S, Dick P, et al. Sustained benefit at 2 years of primary femoropopliteal stenting compared with balloon angioplasty with optional stenting. Circulation. 2007;115:2745-9.
226. Krankenberg H, Schlüter M, Steinkamp HJ, et al. Nitinol stent implantation versus percutaneous transluminal angioplasty in superficial femoral artery lesions up to 10 cm in length: the Femoral Artery Stenting Trial (FAST). Circulation. 2007;116:285-92.
227. Laird JR, Katzen BT, Scheinert D, et al. Nitinol stent implantation versus balloon angioplasty for lesions in the superficial femoral artery and proximal popliteal artery: twelve-month results from the RESILIENT randomized trial. Circ Cardiovasc Interv. 2010;3:267-76.
228. Dick P, Wallner H, Sabeti S, et al. Balloon angioplasty versus stenting with nitinol stents in intermediate length superficial femoral artery lesions. Catheter Cardiovasc Interv. 2009;74:1090-5.
229. Tepe G, Laird J, Schneider P, et al. Drug-coated balloon versus standard percutaneous transluminal angioplasty for the treatment of superficial femoral and popliteal peripheral artery disease: 12-month results from the IN.PACT SFA randomized trial. Circulation. 2015;131:495-502.
230. Liistro F, Grotti S, Porto I, et al. Drug-eluting balloon in peripheral intervention for the superficial femoral artery: the DEBATE-SFA randomized trial (drug eluting balloon in peripheral intervention for the superficial femoral artery). JACC Cardiovasc Interv. 2013;6:1295-302.
231. Scheinert D, Duda S, Zeller T, et al. The LEVANT I (Lutonix paclitaxel-coated balloon for the prevention of femoropopliteal restenosis) trial for femoropopliteal revascularization: first-in-human randomized trial of low-dose drug-coated balloon versus uncoated balloon angioplasty. JACC Cardiovasc Interv. 2014;7:10-9.
232. Werk M, Albrecht T, Meyer DR, et al. Paclitaxel-coated balloons reduce restenosis after femoro-popliteal angioplasty: evidence from the randomized PACIFIER trial. Circ Cardiovasc Interv. 2012;5:831-40.
233. Lammer J, Zeller T, Hausegger KA, et al. Heparin-bonded covered stents versus bare-metal stents for complex femoropopliteal artery lesions: the randomized VIASTAR trial (Viabahn endoprosthesis with PROPATEN bioactive surface [VIA] versus bare nitinol stent in the treatment of long lesions in superficial femoral artery occlusive disease). J Am Coll Cardiol. 2013;62:1320-7.
234. Geraghty PJ, Mewissen MW, Jaff MR, et al. Three-year results of the VIBRANT trial of VIABAHN endoprosthesis versus bare nitinol stent implantation for complex superficial femoral artery occlusive disease. J Vasc Surg. 2013;58:386-95.
235. Saxon RR, Dake MD, Volgelzang RL, et al. Randomized, multicenter study comparing expanded polytetrafluoroethylene-covered endoprosthesis placement with percutaneous transluminal angioplasty in the treatment of superficial femoral artery occlusive disease. J Vasc Interv Radiol. 2008;19:823-32.
236. Kedora J, Hohmann S, Garrett W, et al. Randomized comparison of percutaneous Viabahn stent grafts vs prosthetic femoral-popliteal bypass in the treatment of superficial femoral arterial occlusive disease. J Vasc Surg. 2007;45:10-6.
237. Dake MD, Ansel GM, Jaff MR, et al. Paclitaxel-eluting stents show superiority to balloon angioplasty and bare metal stents in femoropopliteal disease: twelve-month Zilver PTX randomized study results. Circ Cardiovasc Interv. 2011;4:495-504.

© American Heart Association, Inc. and American College of Cardiology Foundation

238. Dake MD, Ansel GM, Jaff MR, et al. Durable clinical effectiveness with paclitaxel-eluting stents in the femoropopliteal artery: 5-year results of the Zilver PTX Randomized Trial. Circulation. 2016;133:1472-83.
239. Duda SH, Bosiers M, Lammer J, et al. Drug-eluting and bare nitinol stents for the treatment of atherosclerotic lesions in the superficial femoral artery: long-term results from the SIROCCO trial. J Endovasc Ther. 2006;13:701-10.
240. Tepe G, Zeller T, Albrecht T, et al. Local delivery of paclitaxel to inhibit restenosis during angioplasty of the leg. N Engl J Med. 2008;358:689-99.
241. Dippel EJ, Makam P, Kovach R, et al. Randomized controlled study of excimer laser atherectomy for treatment of femoropopliteal in-stent restenosis: initial results from the EXCITE ISR trial (EXCImer Laser Randomized Controlled Study for Treatment of FemoropopliTEal In-Stent Restenosis). JACC Cardiovasc Interv. 2015;8:92-101.
242. Banerjee S, Das TS, Abu-Fadel MS, et al. Pilot trial of cryoplasty or conventional balloon post-dilation of nitinol stents for revascularization of peripheral arterial segments: the COBRA trial. J Am Coll Cardiol. 2012;60:1352-9.
243. Whyman MR, Fowkes FG, Kerracher EM, et al. Randomised controlled trial of percutaneous transluminal angioplasty for intermittent claudication. Eur J Vasc Endovasc Surg. 1996;12:167-72.
244. Whyman MR, Fowkes FG, Kerracher EM, et al. Is intermittent claudication improved by percutaneous transluminal angioplasty? A randomized controlled trial. J Vasc Surg. 1997;26:551-7.
245. Perkins JM, Collin J, Creasy TS, et al. Reprinted article "Exercise training versus angioplasty for stable claudication. Long and medium term results of a prospective, randomised trial". Eur J Vasc Endovasc Surg. 2011;42(suppl 1):S41-5.
246. Spronk S, Bosch JL, den Hoed PT, et al. Intermittent claudication: clinical effectiveness of endovascular revascularization versus supervised hospital-based exercise training-randomized controlled trial. Radiology. 2009;250:586-95.
247. Spronk S, Bosch JL, den Hoed PT, et al. Cost-effectiveness of endovascular revascularization compared to supervised hospital-based exercise training in patients with intermittent claudication: a randomized controlled trial. J Vasc Surg. 2008;48:1472-80.
248. Gelin J, Jivegård L, Taft C, et al. Treatment efficacy of intermittent claudication by surgical intervention, supervised physical exercise training compared to no treatment in unselected randomised patients, I: one year results of functional and physiological improvements. Eur J Vasc Endovasc Surg. 2001;22:107-13.
249. Taft C, Karlsson J, Gelin J, et al. Treatment efficacy of intermittent claudication by invasive therapy, supervised physical exercise training compared to no treatment in unselected randomised patients, II: one-year results of health-related quality of life. Eur J Vasc Endovasc Surg. 2001;22:114-23.
250. Hobbs SD, Marshall T, Fegan C, et al. The constitutive procoagulant and hypofibrinolytic state in patients with intermittent claudication due to infrainguinal disease significantly improves with percutaneous transluminal balloon angioplasty. J Vasc Surg. 2006;43:40-6.
251. Nylaende M, Abdelnoor M, Stranden E, et al. The Oslo Balloon Angioplasty versus Conservative Treatment study (OBACT)—the 2-years results of a single centre, prospective, randomised study in patients with intermittent claudication. Eur J Vasc Endovasc Surg. 2007;33:3-12.
252. Greenhalgh RM, Belch JJ, Brown LC, et al. The adjuvant benefit of angioplasty in patients with mild to moderate intermittent claudication (MIMIC) managed by supervised exercise, smoking cessation advice and best medical therapy: results from two randomised trials for stenotic femoropopliteal and aortoiliac arterial disease. Eur J Vasc Endovasc Surg. 2008;36:680-8.
253. Kruidenier LM, Nicolai SP, Rouwet EV, et al. Additional supervised exercise therapy after a percutaneous vascular intervention for peripheral arterial disease: a randomized clinical trial. J Vasc Interv Radiol. 2011;22:961-8.
254. Mazari FA, Khan JA, Carradice D, et al. Randomized clinical trial of percutaneous transluminal angioplasty, supervised exercise and combined treatment for intermittent claudication due to femoropopliteal arterial disease. Br J Surg. 2012;99:39-48.
255. Nordanstig J, Gelin J, Hensäter M, et al. Walking performance and health-related quality of life after surgical or endovascular invasive versus non-invasive treatment for intermittent claudication—a prospective randomised trial. Eur J Vasc Endovasc Surg. 2011;42:220-7.
256. Nordanstig J, Taft C, Hensäter M, et al. Improved quality of life after 1 year with an invasive versus a noninvasive treatment strategy in claudicants: one-year results of the Invasive Revascularization or Not in Intermittent Claudication (IRONIC) Trial. Circulation. 2014;130:939-47.
257. Malgor RD, Alahdab F, Elraiyah TA, et al. A systematic review of treatment of intermittent claudication in the lower extremities. J Vasc Surg. 2015;61:54S-73S.

© American Heart Association, Inc. and American College of Cardiology Foundation

258. Vemulapalli S, Dolor RJ, Hasselblad V, et al. Comparative effectiveness of medical therapy, supervised exercise, and revascularization for patients with intermittent claudication: a network meta-analysis. Clin Cardiol. 2015;38:378-86.
259. McPhail IR, Spittell PC, Weston SA, et al. Intermittent claudication: an objective office-based assessment. J Am Coll Cardiol. 2001;37:1381-5.
260. Schulte KL, Pilger E, Schellong S, et al. Primary self-expanding nitinol stenting vs balloon angioplasty with optional bailout stenting for the treatment of infrapopliteal artery disease in patients with severe intermittent claudication or critical limb ischemia (EXPAND Study). J Endovasc Ther. 2015;22:690-7.
261. Scheinert D, Scheinert S, Sax J, et al. Prevalence and clinical impact of stent fractures after femoropopliteal stenting. J Am Coll Cardiol. 2005;45:312-5.
262. Sakamoto Y, Hirano K, Iida O, et al. Five-year outcomes of self-expanding nitinol stent implantation for chronic total occlusion of the superficial femoral and proximal popliteal artery. Catheter Cardiovasc Interv. 2013;82:E251-E256.
263. Feinglass J, McCarthy WJ, Slavensky R, et al. Functional status and walking ability after lower extremity bypass grafting or angioplasty for intermittent claudication: results from a prospective outcomes study. J Vasc Surg. 2000;31:93-103.
264. Giugliano G, Di SL, Perrino C, et al. Effects of successful percutaneous lower extremity revascularization on cardiovascular outcome in patients with peripheral arterial disease. Int J Cardiol. 2013;167:2566-71.
265. Koivunen K, Lukkarinen H. One-year prospective health-related quality-of-life outcomes in patients treated with conservative method, endovascular treatment or open surgery for symptomatic lower limb atherosclerotic disease. Eur J Cardiovasc Nurs. 2008;7:247-56.
266. Pell JP, Lee AJ. Impact of angioplasty and arterial reconstructive surgery on the quality of life of claudicants. The Scottish Vascular Audit Group. Scott Med J. 1997;42:47-8.
267. Kalbaugh CA, Taylor SM, Blackhurst DW, et al. One-year prospective quality-of-life outcomes in patients treated with angioplasty for symptomatic peripheral arterial disease. J Vasc Surg. 2006;44:296-302.
268. Sachs T, Pomposelli F, Hamdan A, et al. Trends in the national outcomes and costs for claudication and limb threatening ischemia: angioplasty vs bypass graft. J Vasc Surg. 2011;54:1021-31.
269. Shammas NW, Shammas GA, Dippel EJ, et al. Predictors of distal embolization in peripheral percutaneous interventions: a report from a large peripheral vascular registry. J Invasive Cardiol. 2009;21:628-31.
270. Matsi PJ, Manninen HI. Complications of lower-limb percutaneous transluminal angioplasty: a prospective analysis of 410 procedures on 295 consecutive patients. Cardiovasc Intervent Radiol. 1998;21:361-6.
271. Linni K, Ugurluoglu A, Hitzl W, et al. Bioabsorbable stent implantation vs. common femoral artery endarterectomy: early results of a randomized trial. J Endovasc Ther. 2014;21:493-502.
272. Gabrielli R, Rosati MS, Vitale S, et al. Randomized controlled trial of remote endarterectomy versus endovascular intervention for TransAtlantic Inter-Society Consensus II D femoropopliteal lesions. J Vasc Surg. 2012;56:1598-605.
273. Gisbertz SS, Tutein Nolthenius RP, de Borst GJ, et al. Remote endarterectomy versus supragenicular bypass surgery for long occlusions of the superficial femoral artery: medium-term results of a randomized controlled trial (the REVAS trial). Ann Vasc Surg. 2010;24:1015-23.
274. van Det RJ, Vriens BH, van der Palen J, et al. Dacron or ePTFE for femoro-popliteal above-knee bypass grafting: short- and long-term results of a multicentre randomised trial. Eur J Vasc Endovasc Surg. 2009;37:457-63.
275. Gisbertz SS, Ramzan M, Tutein Nolthenius RP, et al. Short-term results of a randomized trial comparing remote endarterectomy and supragenicular bypass surgery for long occlusions of the superficial femoral artery [the REVAS trial]. Eur J Vasc Endovasc Surg. 2009;37:68-76.
276. Ricco JB, Probst H. Long-term results of a multicenter randomized study on direct versus crossover bypass for unilateral iliac artery occlusive disease. J Vasc Surg. 2008;47:45-53.
277. Jensen LP, Lepäntalo M, Fossdal JE, et al. Dacron or PTFE for above-knee femoropopliteal bypass. a multicenter randomised study. Eur J Vasc Endovasc Surg. 2007;34:44-9.
278. AbuRahma AF, Robinson PA, Holt SM. Prospective controlled study of polytetrafluoroethylene versus saphenous vein in claudicant patients with bilateral above knee femoropopliteal bypasses. Surgery. 1999;126:594-602.

© American Heart Association, Inc. and American College of Cardiology Foundation

279. Green RM, Abbott WM, Matsumoto T, et al. Prosthetic above-knee femoropopliteal bypass grafting: five-year results of a randomized trial. J Vasc Surg. 2000;31:417-25.
280. Johnson WC, Lee KK. Comparative evaluation of externally supported Dacron and polytetrafluoroethylene prosthetic bypasses for femorofemoral and axillofemoral arterial reconstructions. Veterans Affairs Cooperative Study #141. J Vasc Surg. 1999;30:1077-83.
281. Klinkert P, Schepers A, Burger DH, et al. Vein versus polytetrafluoroethylene in above-knee femoropopliteal bypass grafting: five-year results of a randomized controlled trial. J Vasc Surg. 2003;37:149-55.
282. Veith FJ, Gupta SK, Ascer E, et al. Six-year prospective multicenter randomized comparison of autologous saphenous vein and expanded polytetrafluoroethylene grafts in infrainguinal arterial reconstructions. J Vasc Surg. 1986;3:104-14.
283. Nguyen BN, Amdur RL, Abugideiri M, et al. Postoperative complications after common femoral endarterectomy. J Vasc Surg. 2015;61:1489-94.
284. Lo RC, Bensley RP, Dahlberg SE, et al. Presentation, treatment, and outcome differences between men and women undergoing revascularization or amputation for lower extremity peripheral arterial disease. J Vasc Surg. 2014;59:409-18.
285. Siracuse JJ, Gill HL, Schneider DB, et al. Assessing the perioperative safety of common femoral endarterectomy in the endovascular era. Vasc Endovascular Surg. 2014;48:27-33.
286. Aihara H, Soga Y, Mii S, et al. Comparison of long-term outcome after endovascular therapy versus bypass surgery in claudication patients with Trans-Atlantic Inter-Society Consensus-II C and D femoropopliteal disease. Circ J. 2014;78:457-64.
287. Boufi M, Azghari A, Belahda K, et al. Subintimal recanalization plus stenting or bypass for management of claudicants with femoro-popliteal occlusions. Eur J Vasc Endovasc Surg. 2013;46:347-52.
288. Sachwani GR, Hans SS, Khoury MD, et al. Results of iliac stenting and aortofemoral grafting for iliac artery occlusions. J Vasc Surg. 2013;57:1030-7.
289. Jones WS, Schmit KM, Vemulapalli S, et al. Treatment Strategies for Patients With Peripheral Artery Disease. Comparative Effectiveness Review No. 118. The Duke Evidence-based Practice Center under Contract No 290-2007-10066-I. 2013;Available at: http://www.effectivehealthcare.ahrq.gov/ehc/products/368/1415/Peripheral-Artery-Disease-Treatment-130301.pdf. Accessed September 25, 2016.
290. Antoniou GA, Chalmers N, Georgiadis GS, et al. A meta-analysis of endovascular versus surgical reconstruction of femoropopliteal arterial disease. J Vasc Surg. 2013;57:242-53.
291. Malgor RD, Ricotta JJ, Bower TC, et al. Common femoral artery endarterectomy for lower-extremity ischemia: evaluating the need for additional distal limb revascularization. Ann Vasc Surg. 2012;26:946-56.
292. Simons JP, Schanzer A, Nolan BW, et al. Outcomes and practice patterns in patients undergoing lower extremity bypass. J Vasc Surg. 2012;55:1629-36.
293. Siracuse JJ, Giles KA, Pomposelli FB, et al. Results for primary bypass versus primary angioplasty/stent for intermittent claudication due to superficial femoral artery occlusive disease. J Vasc Surg. 2012;55:1001-7.
294. Kakkos SK, Haurani MJ, Shepard AD, et al. Patterns and outcomes of aortofemoral bypass grafting in the era of endovascular interventions. Eur J Vasc Endovasc Surg. 2011;42:658-66.
295. Simó G, Banga P, Darabos G, et al. Stent-assisted remote iliac artery endarterectomy: an alternative approach to treating combined external iliac and common femoral artery disease. Eur J Vasc Endovasc Surg. 2011;42:648-55.
296. Eugster T, Marti R, Gurke L, et al. Ten years after arterial bypass surgery for claudication: venous bypass is the primary procedure for TASC C and D lesions. World J Surg. 2011;35:2328-31.
297. Piazza M, Ricotta JJ, Bower TC, et al. Iliac artery stenting combined with open femoral endarterectomy is as effective as open surgical reconstruction for severe iliac and common femoral occlusive disease. J Vasc Surg. 2011;54:402-11.
298. Derksen WJ, Gisbertz SS, Hellings WE, et al. Predictive risk factors for restenosis after remote superficial femoral artery endarterectomy. Eur J Vasc Endovasc Surg. 2010;39:597-603.
299. Koscielny A, Putz U, Willinek W, et al. Case-control comparison of profundaplasty and femoropopliteal supragenicular bypass for peripheral arterial disease. Br J Surg. 2010;97:344-8.

© American Heart Association, Inc. and American College of Cardiology Foundation

300. Ballotta E, Gruppo M, Mazzalai F, et al. Common femoral artery endarterectomy for occlusive disease: an 8-year single-center prospective study. Surgery. 2010;147:268-74.
301. Burke CR, Henke PK, Hernandez R, et al. A contemporary comparison of aortofemoral bypass and aortoiliac stenting in the treatment of aortoiliac occlusive disease. Ann Vasc Surg. 2010;24:4-13.
302. Twine CP, McLain AD. Graft type for femoro-popliteal bypass surgery. Cochrane Database Syst Rev. 2010;CD001487.
303. Chiesa R, Marone EM, Tshomba Y, et al. Aortobifemoral bypass grafting using expanded polytetrafluoroethylene stretch grafts in patients with occlusive atherosclerotic disease. Ann Vasc Surg. 2009;23:764-9.
304. Al-Khoury G, Marone L, Chaer R, et al. Isolated femoral endarterectomy: impact of SFA TASC classification on recurrence of symptoms and need for additional intervention. J Vasc Surg. 2009;50:784-9.
305. Goodney PP, Likosky DS, Cronenwett JL, et al. Predicting ambulation status one year after lower extremity bypass. J Vasc Surg. 2009;49:1431-9.
306. Chang RW, Goodney PP, Baek JH, et al. Long-term results of combined common femoral endarterectomy and iliac stenting/stent grafting for occlusive disease. J Vasc Surg. 2008;48:362-7.
307. Jaquinandi V, Picquet J, Bouye P, et al. High prevalence of proximal claudication among patients with patent aortobifemoral bypasses. J Vasc Surg. 2007;45:312-8.
308. Fowkes F, Leng GC. Bypass surgery for chronic lower limb ischaemia. Cochrane Database Syst Rev. 2008;CD002000.
309. Pereira CE, Albers M, Romiti M, et al. Meta-analysis of femoropopliteal bypass grafts for lower extremity arterial insufficiency. J Vasc Surg. 2006;44:510-7.
310. Rosenthal D, Martin JD, Smeets L, et al. Remote superficial femoral artery endarterectomy and distal aSpire stenting: results of a multinational study at three-year follow-up. J Cardiovasc Surg (Torino). 2006;47:385-91.
311. Martin JD, Hupp JA, Peeler MO, et al. Remote endarterectomy: lessons learned after more than 100 cases. J Vasc Surg. 2006;43:320-6.
312. Mori E, Komori K, Kume M, et al. Comparison of the long-term results between surgical and conservative treatment in patients with intermittent claudication. Surgery. 2002;131:S269-74.
313. Archie JP. Femoropopliteal bypass with either adequate ipsilateral reversed saphenous vein or obligatory polytetrafluoroethylene. Ann Vasc Surg. 1994;8:475-84.
314. Hunink MG, Wong JB, Donaldson MC, et al. Patency results of percutaneous and surgical revascularization for femoropopliteal arterial disease. Med Decis Making. 1994;14:71-81.
315. Schweiger H, Klein P, Lang W. Tibial bypass grafting for limb salvage with ringed polytetrafluoroethylene prostheses: results of primary and secondary procedures. J Vasc Surg. 1993;18:867-74.
316. Baldwin ZK, Pearce BJ, Curi MA, et al. Limb salvage after infrainguinal bypass graft failure. J Vasc Surg. 2004;39:951-7.
317. Leng GC, Lee AJ, Fowkes FG, et al. Incidence, natural history and cardiovascular events in symptomatic and asymptomatic peripheral arterial disease in the general population. Int J Epidemiol. 1996;25:1172-81.
318. Kannel WB, Skinner JJ, Schwartz MJ, et al. Intermittent claudication. Incidence in the Framingham Study. Circulation. 1970;41:875-83.
319. Kannel WB, Shurtleff D. The natural history of arteriosclerosis obliterans. Cardiovasc Clin. 1971;3:37-52.
320. TILLGREN C. Obliterative Arterial Disease of the Lower Limbs. II. A Study of the Course of the Disease. Acta Med Scand. 1965;178:103-19.
321. Jelnes R, Gaardsting O, Hougaard Jensen K, et al. Fate in intermittent claudication: outcome and risk factors. Br Med J (Clin Res Ed). 1986;293:1137-40.
322. Bloor K. Natural history of arteriosclerosis of the lower extremities: Hunterian lecture delivered at the Royal College of Surgeons of England on 22nd April 1960. Ann R Coll Surg Engl. 1961;28:36-52.
323. Dormandy J, Mahir M, Ascady G, et al. Fate of the patient with chronic leg ischaemia. A review article. J Cardiovasc Surg (Torino). 1989;30:50-7.
324. Gandini R, Del GC, Merolla S, et al. Treatment of chronic SFA in-stent occlusion with combined laser atherectomy and drug-eluting balloon angioplasty in patients with critical limb ischemia: a single-center, prospective, randomized study. J Endovasc Ther. 2013;20:805-14.
325. Zeller T, Baumgartner I, Scheinert D, et al. Drug-eluting balloon versus standard balloon angioplasty for infrapopliteal arterial revascularization in critical limb ischemia: 12-month results from the IN.PACT DEEP randomized trial. J Am Coll Cardiol. 2014;64:1568-76.

© American Heart Association, Inc. and American College of Cardiology Foundation

326. Scheinert D, Katsanos K, Zeller T, et al. A prospective randomized multicenter comparison of balloon angioplasty and infrapopliteal stenting with the sirolimus-eluting stent in patients with ischemic peripheral arterial disease: 1-year results from the ACHILLES trial. J Am Coll Cardiol. 2012;60:2290-5.
327. Katsanos K, Spiliopoulos S, Diamantopoulos A, et al. Wound healing outcomes and health-related quality-of-life changes in the ACHILLES trial: 1-year results from a prospective randomized controlled trial of infrapopliteal balloon angioplasty versus sirolimus-eluting stenting in patients with ischemic peripheral arterial disease. JACC Cardiovasc Interv. 2016;9:259-67.
328. Adam DJ, Beard JD, Cleveland T, et al. Bypass versus angioplasty in severe ischaemia of the leg (BASIL): multicentre, randomised controlled trial. Lancet. 2005;366:1925-34.
329. Bradbury AW, Adam DJ, Bell J, et al. Multicentre randomised controlled trial of the clinical and cost-effectiveness of a bypass-surgery-first versus a balloon-angioplasty-first revascularisation strategy for severe limb ischaemia due to infrainguinal disease. The Bypass versus Angioplasty in Severe Ischaemia of the Leg (BASIL) trial. Health Technol Assess. 2010;14:1-210, iii-iv.
330. Bradbury AW, Adam DJ, Bell J, et al. Bypass versus Angioplasty in Severe Ischaemia of the Leg (BASIL) trial: Analysis of amputation free and overall survival by treatment received. J Vasc Surg. 2010;51:18S-31S.
331. Fanelli F, Cannavale A, Boatta E, et al. Lower limb multilevel treatment with drug-eluting balloons: 6-month results from the DEBELLUM randomized trial. J Endovasc Ther. 2012;19:571-80.
332. Rosenfield K, Jaff MR, White CJ, et al. Trial of a paclitaxel-coated balloon for femoropopliteal artery disease. N Engl J Med. 2015;373:145-53.
333. Bosiers M, Scheinert D, Peeters P, et al. Randomized comparison of everolimus-eluting versus bare-metal stents in patients with critical limb ischemia and infrapopliteal arterial occlusive disease. J Vasc Surg. 2012;55:390-8.
334. Rastan A, Tepe G, Krankenberg H, et al. Sirolimus-eluting stents vs. bare-metal stents for treatment of focal lesions in infrapopliteal arteries: a double-blind, multi-centre, randomized clinical trial. Eur Heart J. 2011;32:2274-81.
335. Siablis D, Kitrou PM, Spiliopoulos S, et al. Paclitaxel-coated balloon angioplasty versus drug-eluting stenting for the treatment of infrapopliteal long-segment arterial occlusive disease: the IDEAS randomized controlled trial. JACC Cardiovasc Interv. 2014;7:1048-56.
336. Tepe G, Schnorr B, Albrecht T, et al. Angioplasty of femoral-popliteal arteries with drug-coated balloons: 5-year follow-up of the THUNDER trial. JACC Cardiovasc Interv. 2015;8:102-8.
337. Ferraresi R, Centola M, Ferlini M, et al. Long-term outcomes after angioplasty of isolated, below-the-knee arteries in diabetic patients with critical limb ischaemia. Eur J Vasc Endovasc Surg. 2009;37:336-42.
338. Park SW, Kim JS, Yun IJ, et al. Clinical outcomes of endovascular treatments for critical limb ischemia with chronic total occlusive lesions limited to below-the-knee arteries. Acta Radiol. 2013;54:785-9.
339. Faglia E, Clerici G, Clerissi J, et al. Early and five-year amputation and survival rate of diabetic patients with critical limb ischemia: data of a cohort study of 564 patients. Eur J Vasc Endovasc Surg. 2006;32:484-90.
340. Faglia E, Dalla PL, Clerici G, et al. Peripheral angioplasty as the first-choice revascularization procedure in diabetic patients with critical limb ischemia: prospective study of 993 consecutive patients hospitalized and followed between 1999 and 2003. Eur J Vasc Endovasc Surg. 2005;29:620-7.
341. Iida O, Soga Y, Hirano K, et al. Long-term results of direct and indirect endovascular revascularization based on the angiosome concept in patients with critical limb ischemia presenting with isolated below-the-knee lesions. J Vasc Surg. 2012;55:363-70.
342. Feiring AJ, Krahn M, Nelson L, et al. Preventing leg amputations in critical limb ischemia with below-the-knee drug-eluting stents: the PaRADISE (PReventing Amputations using Drug eluting StEnts) trial. J Am Coll Cardiol. 2010;55:1580-9.
343. Siablis D, Karnabatidis D, Katsanos K, et al. Infrapopliteal application of sirolimus-eluting versus bare metal stents for critical limb ischemia: analysis of long-term angiographic and clinical outcome. J Vasc Interv Radiol. 2009;20:1141-50.
344. Werner M, Schmidt A, Freyer M, et al. Sirolimus-eluting stents for the treatment of infrapopliteal arteries in chronic limb ischemia: long-term clinical and angiographic follow-up. J Endovasc Ther. 2012;19:12-9.
345. Acín F, Varela C, López de Maturana I, et al. Results of infrapopliteal endovascular procedures performed in diabetic patients with critical limb ischemia and tissue loss from the perspective of an angiosome-oriented revascularization strategy. Int J Vasc Med. 2014;2014:270539.

© American Heart Association, Inc. and American College of Cardiology Foundation

346. Alexandrescu VA, Hubermont G, Philips Y, et al. Selective primary angioplasty following an angiosome model of reperfusion in the treatment of Wagner 1-4 diabetic foot lesions: practice in a multidisciplinary diabetic limb service. J Endovasc Ther. 2008;15:580-93.
347. Fossaceca R, Guzzardi G, Cerini P, et al. Endovascular treatment of diabetic foot in a selected population of patients with below-the-knee disease: is the angiosome model effective? Cardiovasc Intervent Radiol. 2013;36:637-44.
348. Kabra A, Suresh KR, Vivekanand V, et al. Outcomes of angiosome and non-angiosome targeted revascularization in critical lower limb ischemia. J Vasc Surg. 2013;57:44-9.
349. Kret MR, Cheng D, Azarbal AF, et al. Utility of direct angiosome revascularization and runoff scores in predicting outcomes in patients undergoing revascularization for critical limb ischemia. J Vasc Surg. 2014;59:121-8.
350. Lejay A, Georg Y, Tartaglia E, et al. Long-term outcomes of direct and indirect below-the-knee open revascularization based on the angiosome concept in diabetic patients with critical limb ischemia. Ann Vasc Surg. 2014;28:983-9.
351. Neville RF, Attinger CE, Bulan EJ, et al. Revascularization of a specific angiosome for limb salvage: does the target artery matter? Ann Vasc Surg. 2009;23:367-73.
352. Osawa S, Terashi H, Tsuji Y, et al. Importance of the six angiosomes concept through arterial-arterial connections in CLI. Int Angiol. 2013;32:375-85.
353. Abu Dabrh AM, Steffen MW, Undavalli C, et al. The natural history of untreated severe or critical limb ischemia. J Vasc Surg. 2015;62:1642-51.
354. Abidia A, Laden G, Kuhan G, et al. The role of hyperbaric oxygen therapy in ischaemic diabetic lower extremity ulcers: a double-blind randomised-controlled trial. Eur J Vasc Endovasc Surg. 2003;25:513-8.
355. Weaver FA, Comerota AJ, Youngblood M, et al. Surgical revascularization versus thrombolysis for nonembolic lower extremity native artery occlusions: results of a prospective randomized trial. The STILE Investigators. Surgery versus Thrombolysis for Ischemia of the Lower Extremity. J Vasc Surg. 1996;24:513-21.
356. Ouriel K, Veith FJ, Sasahara AA. A comparison of recombinant urokinase with vascular surgery as initial treatment for acute arterial occlusion of the legs. Thrombolysis or Peripheral Arterial Surgery (TOPAS) Investigators. N Engl J Med. 1998;338:1105-11.
357. Ponec D, Jaff MR, Swischuk J, et al. The Nitinol SMART stent vs Wallstent for suboptimal iliac artery angioplasty: CRISP-US trial results. J Vasc Interv Radiol. 2004;15:911-8.
358. Schillinger M, Sabeti S, Loewe C, et al. Balloon angioplasty versus implantation of nitinol stents in the superficial femoral artery. N Engl J Med. 2006;354:1879-88.
359. Conte MS, Bandyk DF, Clowes AW, et al. Results of PREVENT III: a multicenter, randomized trial of edifoligide for the prevention of vein graft failure in lower extremity bypass surgery. J Vasc Surg. 2006;43:742-51.
360. Farber A, Rosenfield K, Menard M. The BEST-CLI trial: a multidisciplinary effort to assess which therapy is best for patients with critical limb ischemia. Tech Vasc Interv Radiol. 2014;17:221-4.
361. Veves A, Sheehan P, Pham HT. A randomized, controlled trial of Promogran (a collagen/oxidized regenerated cellulose dressing) vs standard treatment in the management of diabetic foot ulcers. Arch Surg. 2002;137:822-7.
362. Fogle MA, Whittemore AD, Couch NP, et al. A comparison of in situ and reversed saphenous vein grafts for infrainguinal reconstruction. J Vasc Surg. 1987;5:46-52.
363. Rashid H, Slim H, Zayed H, et al. The impact of arterial pedal arch quality and angiosome revascularization on foot tissue loss healing and infrapopliteal bypass outcome. J Vasc Surg. 2013;57:1219-26.
364. Nolan BW, De Martino RR, Stone DH, et al. Prior failed ipsilateral percutaneous endovascular intervention in patients with critical limb ischemia predicts poor outcome after lower extremity bypass. J Vasc Surg. 2011;54:730-5.
365. Santo VJ, Dargon P, Azarbal AF, et al. Lower extremity autologous vein bypass for critical limb ischemia is not adversely affected by prior endovascular procedure. J Vasc Surg. 2014;60:129-35.
366. Uhl C, Hock C, Betz T, et al. Pedal bypass surgery after crural endovascular intervention. J Vasc Surg. 2014;59:1583-7.

© American Heart Association, Inc. and American College of Cardiology Foundation

367. Korhonen M, Biancari F, Söderström M, et al. Femoropopliteal balloon angioplasty vs. bypass surgery for CLI: a propensity score analysis. Eur J Vasc Endovasc Surg. 2011;41:378-84.
368. Kasemi H, Marino M, Dionisi CP, et al. Seven-year approach evolution of the aortoiliac occlusive disease endovascular treatment. Ann Vasc Surg. 2016;30:277-85.
369. Bredahl K, Jensen LP, Schroeder TV, et al. Mortality and complications after aortic bifurcated bypass procedures for chronic aortoiliac occlusive disease. J Vasc Surg. 2015;62:75-82.
370. Chew DK, Conte MS, Donaldson MC, et al. Autogenous composite vein bypass graft for infrainguinal arterial reconstruction. J Vasc Surg. 2001;33:259-64.
371. Ruffolo AJ, Romano M, Ciapponi A. Prostanoids for critical limb ischaemia. Cochrane Database Syst Rev. 2010;CD006544.
372. Moran PS, Teljeur C, Harrington P, et al. A systematic review of intermittent pneumatic compression for critical limb ischaemia. Vasc Med. 2015;20:41-50.
373. Kobayashi N, Hirano K, Nakano M, et al. Prognosis of critical limb ischemia patients with tissue loss after achievement of complete wound healing by endovascular therapy. J Vasc Surg. 2015;61:951-9.
374. Rutherford RB. Acute limb ischemia: Clinical assessment and standards for reporting. Semin Vasc Surg. 1992;5:4-10.
375. Nypaver TJ, Whyte BR, Endean ED, et al. Nontraumatic lower-extremity acute arterial ischemia. Am J Surg. 1998;176:147-52.
376. FOGARTY TJ, CRANLEY JJ. Catheter Technic for Arterial Embolectomy. Ann Surg. 1965;161:325-30.
377. Shin HS, Kyoung KH, Suh BJ, et al. Acute limb ischemia: surgical thromboembolectomy and the clinical course of arterial revascularization at ankle. Int J Angiol. 2013;22:109-14.
378. de Donato G, Setacci F, Sirignano P, et al. The combination of surgical embolectomy and endovascular techniques may improve outcomes of patients with acute lower limb ischemia. J Vasc Surg. 2014;59:729-36.
379. Baril DT, Patel VI, Judelson DR, et al. Outcomes of lower extremity bypass performed for acute limb ischemia. J Vasc Surg. 2013;58:949-56.
380. Manojlovic V, Popovic V, Nikolic D, et al. Analysis of associated diseases in patients with acute critical lower limb ischemia. Med Pregl. 2013;66:41-5.
381. Duval S, Keo HH, Oldenburg NC, et al. The impact of prolonged lower limb ischemia on amputation, mortality, and functional status: the FRIENDS registry. Am Heart J. 2014;168:577-87.
382. Morris-Stiff G, D'Souza J, Raman S, et al. Update experience of surgery for acute limb ischaemia in a district general hospital-are we getting any better? Ann R Coll Surg Engl. 2009;91:637-40.
383. Londero LS, Nørgaard B, Houlind K. Patient delay is the main cause of treatment delay in acute limb ischemia: an investigation of pre- and in-hospital time delay. World J Emerg Surg. 2014;9:56.
384. Ouriel K, Shortell CK, DeWeese JA, et al. A comparison of thrombolytic therapy with operative revascularization in the initial treatment of acute peripheral arterial ischemia. J Vasc Surg. 1994;19:1021-30.
385. Results of a prospective randomized trial evaluating surgery versus thrombolysis for ischemia of the lower extremity. The STILE trial. Ann Surg. 1994;220:266-8.
386. Comerota AJ, Weaver FA, Hosking JD, et al. Results of a prospective, randomized trial of surgery versus thrombolysis for occluded lower extremity bypass grafts. Am J Surg. 1996;172:105-12.
387. Diffin DC, Kandarpa K. Assessment of peripheral intraarterial thrombolysis versus surgical revascularization in acute lower-limb ischemia: a review of limb-salvage and mortality statistics. J Vasc Interv Radiol. 1996;7:57-63.
388. Schrijver AM, Reijnen MM, van Oostayen JA, et al. Dutch randomized trial comparing standard catheter-directed thrombolysis versus ultrasound-accelerated thrombolysis for thromboembolic infrainguinal disease (DUET): design and rationale. Trials. 2011;12:20.
389. Fagundes C, Fuchs FD, Fagundes A, et al. Prognostic factors for amputation or death in patients submitted to vascular surgery for acute limb ischemia. Vasc Health Risk Manag. 2005;1:345-9.
390. FOGARTY TJ, CRANLEY JJ, KRAUSE RJ, et al. A method for extraction of arterial emboli and thrombi. Surg Gynecol Obstet. 1963;116:241-4.
391. Eliason JL, Wakefield TW. Metabolic consequences of acute limb ischemia and their clinical implications. Semin Vasc Surg. 2009;22:29-33.

© American Heart Association, Inc. and American College of Cardiology Foundation

392. Lurie F, Vaidya V, Comerota AJ. Clinical outcomes and cost-effectiveness of initial treatment strategies for nonembolic acute limb ischemia in real-life clinical settings. J Vasc Surg. 2015;61:138-46.
393. Taha AG, Byrne RM, Avgerinos ED, et al. Comparative effectiveness of endovascular versus surgical revascularization for acute lower extremity ischemia. J Vasc Surg. 2015;61:147-54.
394. Gupta R, Hennebry TA. Percutaneous isolated pharmaco-mechanical thrombolysis-thrombectomy system for the management of acute arterial limb ischemia: 30-day results from a single-center experience. Catheter Cardiovasc Interv. 2012;80:636-43.
395. Ansel GM, Botti CF, Silver MJ. Treatment of acute limb ischemia with a percutaneous mechanical thrombectomy-based endovascular approach: 5-year limb salvage and survival results from a single center series. Catheter Cardiovasc Interv. 2008;72:325-30.
396. Byrne RM, Taha AG, Avgerinos E, et al. Contemporary outcomes of endovascular interventions for acute limb ischemia. J Vasc Surg. 2014;59:988-95.
397. Schernthaner MB, Samuels S, Biegler P, et al. Ultrasound-accelerated versus standard catheter-directed thrombolysis in 102 patients with acute and subacute limb ischemia. J Vasc Interv Radiol. 2014;25:1149-56.
398. Silva JA, Ramee SR, Collins TJ, et al. Rheolytic thrombectomy in the treatment of acute limb-threatening ischemia: immediate results and six-month follow-up of the multicenter AngioJet registry. Possis Peripheral AngioJet Study AngioJet Investigators. Cathet Cardiovasc Diagn. 1998;45:386-93.
399. Kasirajan K, Gray B, Beavers FP, et al. Rheolytic thrombectomy in the management of acute and subacute limb-threatening ischemia. J Vasc Interv Radiol. 2001;12:413-21.
400. Allie DE, Hebert CJ, Lirtzman MD, et al. Novel simultaneous combination chemical thrombolysis/rheolytic thrombectomy therapy for acute critical limb ischemia: the power-pulse spray technique. Catheter Cardiovasc Interv. 2004;63:512-22.
401. Elmahdy MF, Ghareeb MS, Baligh EE, et al. Value of duplex scanning in differentiating embolic from thrombotic arterial occlusion in acute limb ischemia. Cardiovasc Revasc Med. 2010;11:223-6.
402. Ascher E, Hingorani A, Markevich N, et al. Acute lower limb ischemia: the value of duplex ultrasound arterial mapping (DUAM) as the sole preoperative imaging technique. Ann Vasc Surg. 2003;17:284-9.
403. Lowery AJ, Hynes N, Manning BJ, et al. A prospective feasibility study of duplex ultrasound arterial mapping, digital-subtraction angiography, and magnetic resonance angiography in management of critical lower limb ischemia by endovascular revascularization. Ann Vasc Surg. 2007;21:443-51.
404. Leung DA, Blitz LR, Nelson T, et al. Rheolytic pharmacomechanical thrombectomy for the management of acute limb ischemia: results from the PEARL Registry. J Endovasc Ther. 2015;22:546-57.
405. Schrijver AM, Reijnen MM, van Oostayen JA, et al. Initial results of catheter-directed ultrasound-accelerated thrombolysis for thromboembolic obstructions of the aortofemoral arteries: a feasibility study. Cardiovasc Intervent Radiol. 2012;35:279-85.
406. Schrijver A, Vos J, Hoksbergen AW, et al. Ultrasound-accelerated thrombolysis for lower extremity ischemia: multicenter experience and literature review. J Cardiovasc Surg (Torino). 2011;52:467-76.
407. Ihlberg L, Luther M, Albäck A, et al. Does a completely accomplished duplex-based surveillance prevent vein-graft failure? Eur J Vasc Endovasc Surg. 1999;18:395-400.
408. Lundell A, Lindblad B, Bergqvist D, et al. Femoropopliteal-crural graft patency is improved by an intensive surveillance program: a prospective randomized study. J Vasc Surg. 1995;21:26-33.
409. Jongsma H, Bekken JA, van Buchem F, et al. Secondary interventions in patients with autologous infrainguinal bypass grafts strongly improve patency rates. J Vasc Surg. 2016;63:385-90.
410. Carter A, Murphy MO, Halka AT, et al. The natural history of stenoses within lower limb arterial bypass grafts using a graft surveillance program. Ann Vasc Surg. 2007;21:695-703.
411. Westerband A, Mills JL, Kistler S, et al. Prospective validation of threshold criteria for intervention in infrainguinal vein grafts undergoing duplex surveillance. Ann Vasc Surg. 1997;11:44-8.
412. Mills JL, Harris EJ, Taylor LM, et al. The importance of routine surveillance of distal bypass grafts with duplex scanning: a study of 379 reversed vein grafts. J Vasc Surg. 1990;12:379-86.

© American Heart Association, Inc. and American College of Cardiology Foundation

413. Brumberg RS, Back MR, Armstrong PA, et al. The relative importance of graft surveillance and warfarin therapy in infrainguinal prosthetic bypass failure. J Vasc Surg. 2007;46:1160-6.
414. Calligaro KD, Doerr K, McAffee-Bennett S, et al. Should duplex ultrasonography be performed for surveillance of femoropopliteal and femorotibial arterial prosthetic bypasses? Ann Vasc Surg. 2001;15:520-4.
415. Stone PA, Armstrong PA, Bandyk DF, et al. Duplex ultrasound criteria for femorofemoral bypass revision. J Vasc Surg. 2006;44:496-502.
416. Back MR, Novotney M, Roth SM, et al. Utility of duplex surveillance following iliac artery angioplasty and primary stenting. J Endovasc Ther. 2001;8:629-37.
417. Baril DT, Marone LK. Duplex evaluation following femoropopliteal angioplasty and stenting: criteria and utility of surveillance. Vasc Endovascular Surg. 2012;46:353-7.
418. Troutman DA, Madden NJ, Dougherty MJ, et al. Duplex ultrasound diagnosis of failing stent grafts placed for occlusive disease. J Vasc Surg. 2014;60:1580-4.
419. Connors G, Todoran TM, Engelson BA, et al. Percutaneous revascularization of long femoral artery lesions for claudication: patency over 2.5 years and impact of systematic surveillance. Catheter Cardiovasc Interv. 2011;77:1055-62.

© American Heart Association, Inc. and American College of Cardiology Foundation
